# Interspecies evolutionary divergence in *Liriodendron*, evidence from the nucleotide variations of *LcDHN-like* gene

**DOI:** 10.1186/s12862-018-1318-7

**Published:** 2018-12-19

**Authors:** Yanli Cheng, Huogen Li

**Affiliations:** The Southern Modern Forestry Collaborative Innovation Center, College of Forestry, Nanjing Forestry University, Nanjing, 210037 China

**Keywords:** *Liriodendron*, Divergence, Dehydrin, Nucleotide variation, Adaptation

## Abstract

**Background:**

*Liriodendron* is a genus of Magnoliaceae, which consists of two relict species, *Liriodendron chinense* and *L. tulipifera*. Although the morphologies are highly similar, the two species exhibit different adaptive capacity. Dehydrins (DHNs) are abiotic stresses resistant proteins in *planta*, which are associated with adaptive evolution. To better understand the evolution divergence between *L. chinense* and *L. tulipifera* and how *DHN* genes are associated with adaptation evolution, we firstly investigated the DNA polymorphisms of the *LcDHN-like* gene in 21 *L. chinense* and 6 *L. tulipifera* populations.

**Results:**

A 707 bp *LcDHN-like* gene was cloned, which included a 477 bp open reading frame (ORF) and coding 158 amino acids. 311 *LcDHN-like* gDNA sequences were obtained from 70 *L. chinense* and 35 *L. tulipifera* individuals. The AMOVA and phylogenetic relationship analysis showed significant differences between the two species. A higher genetic diversity was observed in *L. tulipifera* compared to *L. chinense*, in consistent with the higher adaptive capacity of *L. tulipifera*. Our data also suggested that the *LcDHN-like* genes’ polymorphisms were under neutral mutation and purifying selection model in the *L. chinense* and *L. tulipifera* populations, respectively. The distinct expanding range and rate between the two species, haplotypes shared only in *L.chinense*’s nearby populations, and wide dispersals in *L. tulipifera* could contribute to the obscure east-west separation in *L. chinense* and entirely unordered phylogeny in *L. tulipifera*. The completely separated nonsynonymous substitution at position 875 and the higher range scope of aliphatic index in *L. tulipifera* populations may be related with its higher adaptive capacity. Taken together, our study suggests *LcDHN-like* gene is a potential mark gene responsible for adaptive evolution divergence in *Liriodendron*.

**Conclusions:**

Significant differences and completely distinct haplogroups between *L. chinense* and *L. tulipifera* showed that the two species have evolved into different directions. The more widely distribution, earlier haplogroups divergence events, and richer SNPs variations in *L. tulipifera* could imply its stronger adaptation in this species. And potential effect of the allelic variations in *LcDHN-like* gene may reflect the difference of water stress and chill tolerance between *L. chinense* and *L. tulipifera*, which could provide some information for further adaption evolution studies of *Liriodendron*.

**Electronic supplementary material:**

The online version of this article (10.1186/s12862-018-1318-7) contains supplementary material, which is available to authorized users.

## Background

*Liriodendron tulipifera* and *L. chinense* is an east Asian-east North American sister species pair [1]. They are well-known for rapid growth, valuable wood, attractive leaves and flowers, thus having great ecological and economic potential [1–3]. *L. chinense* distributes in southern China and northern Vietnam as a scattered population pattern [4]. Because of endangering factors including endangering habitat, degraded population structure, low seed germination rates and artificial interference, *L. chinense* was listed in the IUCN Red List of Threatened Species by the International Union for Conservation of Nature and Natural Resources [5–8]. On the contrary, *L. tulipifera* abundantly and widely distributed between27°and 42°north latitude and predominant in east of the Mississippi River [1, 9, 10].

As an excellent pioneer species, *L. tulipifera* possesses a stronger adaptive capacity than *L. chinense* [11]. Previous studies have shown that *L. chinense* is more sensitive to low-temperature and waterflooding [12–14]. The variations of adaptive genes at DNA level in other plants has been shown to be highly correlated with their adaptation difference, which also could provide a snapshot of evolution divergence. For example, nucleotide variations at several cold candidate genes were surveyed in Scots pine, which showed that significant differentiation in allelic frequency or haplotypes structure between north and south populations was detected at dhn1, dhn3, and abaH loci [15]. The difference of nucleotide diversity, haplotypes, and expression after dehydration of *Dhn1* between ‘African’ dry slope (AS) and ‘European’ humid slope (ES) showed that adaptive natural microclimatic selection is the major evolutionary divergent driving force of *Hordeum spontaneum* in ‘Evolution Canyon’ (ECI) at Lower Nahal Oren, Mount Carmel, Israel [16].

*Dehydrin (DHN)*, also called *LEA-D11* or *LEA II*, belongs to a small gene family of the late embryogenesis abundant (LEA) proteins [17]. *DHNs* are largely expressed during the later stage of seeds maturation or/and the cell dehydration process caused by environmental stresses, such as drought, cold, and high salinity [18–20]. DHN proteins contain three conserved segments, which are (V/T)D(E/Q)YGNP, (LHRSGS4–10(E/D)3), and EKKGIM(E/D)KIKEKLPG], named Y-, S-, and K-segment, respectively. Based on the appearance and order of these segments, DHN proteins can be classified into five subgroups K_n_, SK_n_, K_n_S, Y_n_SK_m_, and Y_n_K_m_ [21].Y-segment, normally found in the N terminus of DHN protein, is similar with partial amino acids of nucleotide binding sites of chaperones in plant and bacterial [3, 21–23].S-segment, a string of Serine residues, could be phosphorylated and promote DHNs interaction with specific signal peptides for the translocation of DHNs into the nucleus [21, 24, 25]. K-segment, a Lysine-rich15-residues, usually exist at the C terminal, could form an amphipathic “α- helix” like structure, which may play an important role in the effect of hydrophobicity /hydrophilicity [21, 23].

In this study, we firstly investigated the variations of *DHN* genes’ coding DNA sequence (CDS) among the *L. chinense* and *L. tulipifera* populations. Full-length of *LcDHN-like* gene was cloned by Rapid Amplification of cDNA Ends (RACE) and the corresponding coding protein was predicted. We then explored the evolution dynamics of *LcDHN-like* gene by analyzing the DNA polymorphisms. Second, we conducted population structure, phylogeny and demographic expansion analysis to explore the different evolutionary history in *L. chinense* and *L. tulipifera*, respectively. All together, this study would not only benefit the understanding of the evolutionary divergence interspecies, but also contribute to analyze the evolution of *DHN* genes and lay the foundation for seeking the difference of water stress, low-temperature adaption in different *Liriodendron* populations.

## Materials and methods

### Plant materials

The petals for gene cloning were gathered from a 21-year-old tree in April 2015, which located at a provenance trial plantation in Xiashu, Jurong County, Jiangsu Province (119°13′20″E, 32°7′8″N) [26]. Fresh petals were quick frozen and brought back to laboratory storing at − 80 °C in a freezer prior. Total RNA and DNA were extracted using RNAprep Pure Plant Kit and Plant Genomic DNA Kit, respectively (Tiangen Biotech, China).

27 *Liriodendron* populations, including 21 *L. chinense* and 6 *L. tulipifera*, were sampled from different geographical origins. Each population contains 2 to 5 individuals, altogether, 105 individuals were taken as plant materials for this study (Table 1).Total genomic DNA was isolated from their young leaves or winter buds according to the protocol provided by Plant Genomic DNA Kit (Tiangen Biotech, China).

**Table 1:** Plant materials and haplotypes of *Liriodendron* populations

Code	Site	Sample size	Sequences number	Haplotype type
ZJ-AJ	Anji, Zhejiang, CHN	4	15	Hap1- Hap6
ZJ-SY	Songyang, Zhejiang, CHN	4	12	Hap7- Hap9
AH-JX	Jixi, Anhui, CHN	3	8	Hap4, Hap5
AH-HS	Huangshan, Anhui, CHN	3	7	Hap5,Hap10,Hap11
JX-LS	Lushan, Jiangxi, CHN	3	9	Hap12, Hap13
FJ-WYS	Wuyishan, Fujian, CHN	3	9	Hap5-Hap7,Hap14- Hap17
HB-XN	Xianning, Hubei, CHN	4	13	Hap18- Hap23
HB-EX	Exi, Hubei, CHN	4	12	Hap20,Hap21,Hap24- Hap26
HN-SN	Suining, Hunan, CHN	3	9	Hap18,Hap27- Hap29
GX-LY	Leye, Guangxi, CHN	3	9	Hap30- Hap33
GX-MES	Maoershan, Guangxi, CHN	3	9	Hap34- Hap36
GX-HP	Huaping, Guangxi, CHN	3	8	Hap33,Hap37, Hap38
GZ-YJ	Yinjiang, Guizhou, CHN	4	11	Hap39- Hap43
GZ-ST	Songtao, Guizhou, CHN	3	9	Hap20,Hap44- Hap46
GZ-XS	Xishui, Guizhou, CHN	2	6	Hap47- Hap49
GZ-LP	Liping, Guizhou, CHN	4	10	Hap50- Hap52
SC-XY	Xuyong, Sichuan, CHN	3	9	Hap48,Hap53- Hap55
SC-YY	Youyang, Sichuan, CHN	3	9	Hap56- Hap58
YN-XC	Xichou, Yunnan, CHN	4	12	Hap34-Hap36, Hap43, Hap58- Hap62
YN-MG	Maguan, Yunnan, CHN	4	8	Hap60
YN-JP	Jinping, Yunnan, CHN	3	8	Hap63, Hap64
Hershey	Pennsyjvania, USA	5	14	Hap65- Hap75
BK	North Carolina, USA	6	17	Hap70,Hap72,Hap76- Hap79
MSL	Missouri, USA	6	20	Hap72, Hap80- Hap92
ZZY	Georgia, USA	5	15	Hap68,Hap70, Hap78, Hap93- Hap97
NK	South Carolina, USA	6	21	Hap68,Hap70,Hap76,Hap78,Hap95,Hap96,Hap98-Hap107
LYS	Louisiana, USA	7	22	Hap72,Hap78,Hap108-Hap123
Total	27 populations	105	311	

### Full-length cDNA cloning by RACE

The full-length cDNA was obtained by Rapid Amplification of cDNA Ends (RACE). 3’-RACE and 5’-RACE cDNA were synthesized using RACE kits (3’-Full RACE Core Set with PrimeScript RTase, 5’-Full RACE Kit, Takara, Japan). The RACE primers(Additional file 1: Table S1) were designed basing on EST sequence of *DHN*, which acquired by searching “Dehydrin” annotation in *L. chinense*’s transcriptome database [27]. All the PCR reactions were carried out in a 50 μL reaction mix according to the PCR protocol (Additional file 1: Table S1). The reaction mix consisted of 5 μL 10 × LA PCR Buffer II (Mg^2+^ Free), 5 μL MgCL_2_ (25 mmol·L^− 1^), 8 μL dNTP Mixture (2.5 mmol·L^− 1^ each), 2 μL of each primer (2.5 mmol·L^− 1^ each), 0.5 μL *TaKaRa LA Taq* (5 U·L^− 1^), 2 μL each cDNA, 25.5 μL ddH_2_O. PCR products were separated on a 1.5% agarose gel and purified using the DNA gel extraction kit (Transgen Biotech, China). The purified PCR products were cloned into pEASY®-T1 Cloning Vector and transferred into Trans5α Chemically Competent Cells for white-blue plaque selection (Transgen Biotech, China). Positive monoclonal was screened for sequencing (Genscript, China). Finally, the gene full-length was assembled according to the 3′ and 5’sequencing results.

### Open reading frame prediction, verification and analysis

The Open reading frame (ORF) and corresponding protein was forecasted using full-length sequence by ORF Finder (https://www.ncbi.nlm.nih.gov/orffinder/). ORF primers were designed in the untranslated regions (UTR) to ensure getting the whole ORF (Additional file 1: Table S1). cDNA of *L. chinense* was reversed using RevertAid strand cDNA Synthesis Kit (Thermo Scientific, USA). 25 μL 2 × TransStart FastPfu PCR SuperMix, 2 μL of each ORF primer (2.5 mmol·L^− 1^ each), 2 μL cDNA, 19 μL ddH_2_O were mixed for PCR. The PCR protocol of ORF was same with above, except that inserted PCR products into pEASY®-Blunt Cloning Vector (Transgen Biotech, China). ORF and the coding protein were blasted in NCBI to make sure that the cloning result is accurate. The conserved motifs were manually identified according to the conserved sequences (Y, S, K) of DHN. LcDHN-like protein and 13 homologous DHN proteins from other plants (obtaining from the NCBI database) were used to construct an UPGMA tree by MEGA version 6 [28]. Bootstrap values were estimated at 1000 replications.

### gDNA cloning of *LcDHN-like* gene among *Liriodendron* populations

The gDNA sequences of *LcDHN-like* gene of all the populations were obtained using the same primers, PCR procedure and cloning methods with ORF. Each individual selected 1–4 positive monoclonal for sequencing and DNA sequences were summarized.

### Sequence diversity and selection mode analyses

DNA sequences were aligned using Clustalx1.83 [29]. DnaSP v5 software was employed to achieve statistical estimates of polymorphic sites (S), InDels (Insertion-Deletion) sites, nucleotide diversity (π) and Theta (per site) from S (θ_W_) [30].

The departures from the standard neutral model of evolution was evaluated by three models of Tajima’s D, Fu and Li’s F* and Fu and Li’s D* statistics with DnaSP v5 [31–33]. Significantly negative value of Tajima’s D means existing excess of low frequency polymorphisms, which is consistent with positive directional selection, exhibiting mildly deleterious alleles or a recent population expansion [31, 34, 35]. Significantly positive value of Tajima’s D means existing excess of intermediate-frequency polymorphisms, which may be indicative of balancing selection or a population contraction [31, 34, 35]. If purifying or negative selection and advantageous alleles have recently become fixed in the population, the values of Fu and Li’s F* and Fu and Li’s D* are significantly negative [33]. If balancing selection has happened, the values of Fu and Li’s F* and Fu and Li’s D* are significantly positive [33].

The selection pressures were quantified by likelihood ratio test (LRT), which compares dN and dS by paml4.8 package [36]. dN/dS < 1 means purifying selection; dN/dS = 1 means neutral evolution; dN/dS > 1 means positive selection. Z-test of selection with modified Nei–Gojobori algorithm via the Jukes-Cantor was analyzed by MEGA version 6 to test the significance [37]. The population size changes of *Liriodendron* were inferred by DnaSP v5 [30].

### Population genetic structure analyses

The AMOVA program within the Arlequinver 3.5.2.2 software package was applied to evaluate the genetic variance between *L. chinense* and *L. tulipifera* populations and within the two species [38].The number of permutations was set as 1000 and showed significant difference when significance tests at *P* < 0.05 level.

We inferred the population structure of *Liriodendro*n by employing the Bayesian clustering algorithm with admixture Model and allele frequencies correlated models in STRUCTURE V2.3 [39, 40]. The K values were ranging from 2 to 27, the upper bound of which was the number of actual sampled populations. Each K value was run 10 times with 100,000 steps after a burn-in period of 10,000 steps. We estimated the most probable K value by StructureHarvester v0.6.93, which was eventually determined by the relationship between ΔK and K [41]. Replicate cluster analyses of the results about optimum K value were performed by CLUster Matching and Permutation Program version 1.1.2 and the final outputs from the Bayesian analyses were visualized clearly by DISTRUCT v1.1 [42].

### Phylogenetic relationship and geographical distribution of haplotypes

Haplotypes data and diversity of *LcDHN-like* gene in *Liriodendron* were generated by DnaSP version 5.0 [30].The original UPGMA tree and Time Trees of haplotypes were constructed by MEGA version 6 with 1000 bootstrap replications [37]. The calibration constraints was set according to the separating time of *L. chinense* and *L. tulipifera*, about 10–16 million years ago, by which MEGA could produce absolute divergence times for all branching points in the tree based on the RelTime method [28, 43]. And, representative divided haplogroups were summarized by sketchy phylogenetic tree.

The phylogenetic relationships among haplotypes were constructed by Median-Joining network in NETWORK version 4.2.0.1 [44, 45]. For a high quality final graphics, the Median-Joining network was enhanced by another Reduced-Median network to simplify the outcome (All parameters were designated as the default values) [45].

The geographical distribution of haplotypes from sampled sites was marked on the map of China and American. And different *LcDHN-like* haplotypes in each sample site were drawn as circle pie proportionally by Adobe Illustrator CS6 [46].

### Demographic history analysis

Pairwise mismatch distributions were carried out by Arlequinver 3.5.2.2 to infer the historical demography of *L. chinense* and *L. tulipifera*, with the expected frequency based on a constant population size model [30]. Two specific parameters, the sum of squared deviations (SSD) and Harpending’s raggedness index (HRag), were used to test the goodness of fit under a spatial expansion model. Their significances were tested using a parametric bootstrap approach with 1000 replications, and showed significant difference when significance tests at *P* < 0.05 level. τ = 2ut was used to calculate the recent expansion time, where u (u = μk) is the mutation rate for the whole haplotypes. μ, calculated by eq. 9.62 of Nei, is the mutation rate per nucleotide; k is the number of nucleotides in the sequence [47, 48].

The population history of *L. chinense* and *L. tulipifera* was also investigated using Bayesian Skyline Plot (BSP) in BEAST v.1.4, which employed a coalescent Bayesian Skyline tree prior and uncorrelated relaxed clock fixing prior substitutions rate at 2.0 × 10^− 8^ per year [49]. The GTR nucleotide substitution model for sequence evolution was estimated by MEGA version 6 [37]. Each MCMC sample was based on a run of 10,000,000 generations, sampled every 1000, with the first 1000,000 generations discarded as burn-in. Bayesian Skyline Plots were summarized by Tracer v1.7 [50].

### Protein structure prediction

The exons of *LcDHN-like* gene were selected and translated into proteins by MEGA 6. These proteins were aligned and then classified by pairwise distance in MEGA 6. Expasy ProtParam(http://web.expasy.org/protparam/)was applied to compute the various physical and chemical parameters of LcDHN-like proteins. The secondary structure of proteins was predicted using SOPMA (https://npsa-prabi.ibcp.fr/cgibin/npsa_automat.pl?page=npsa_sopma.html).

## Results

### Isolation and characterization of *LcDHN-like* gene

**Fig. 1:**
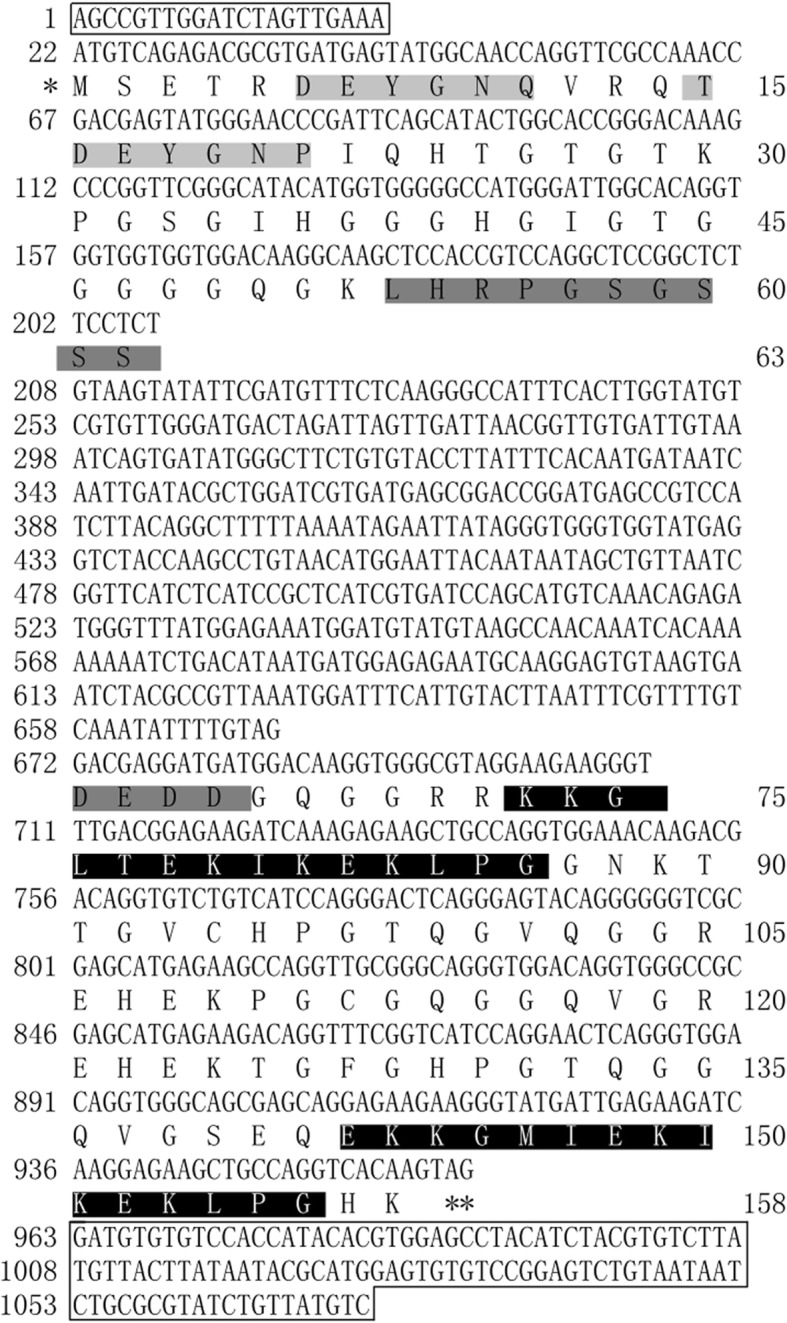
Sequences analysis of *LcDHN-like* gene. Note: 1. Nucleotide and amino acid numbers are counted on the left and right separately. 2. The ORF split into two exons (exon 1:22-207 bp; exon 2:672-962 bp, coding 158 amino acids) by one intron (209-672 bp). 3. The Y- segments are highlighted in *light grey*; The S-segment is highlighted in *grey;* The K-segments are highlighted in *black*. 4. Start and stop codons are indicated by asterisks and double asterisks, respectively. 5. Nucleotides are boxed represent 5’and 3′ untranslated regions

**Fig. 2:**
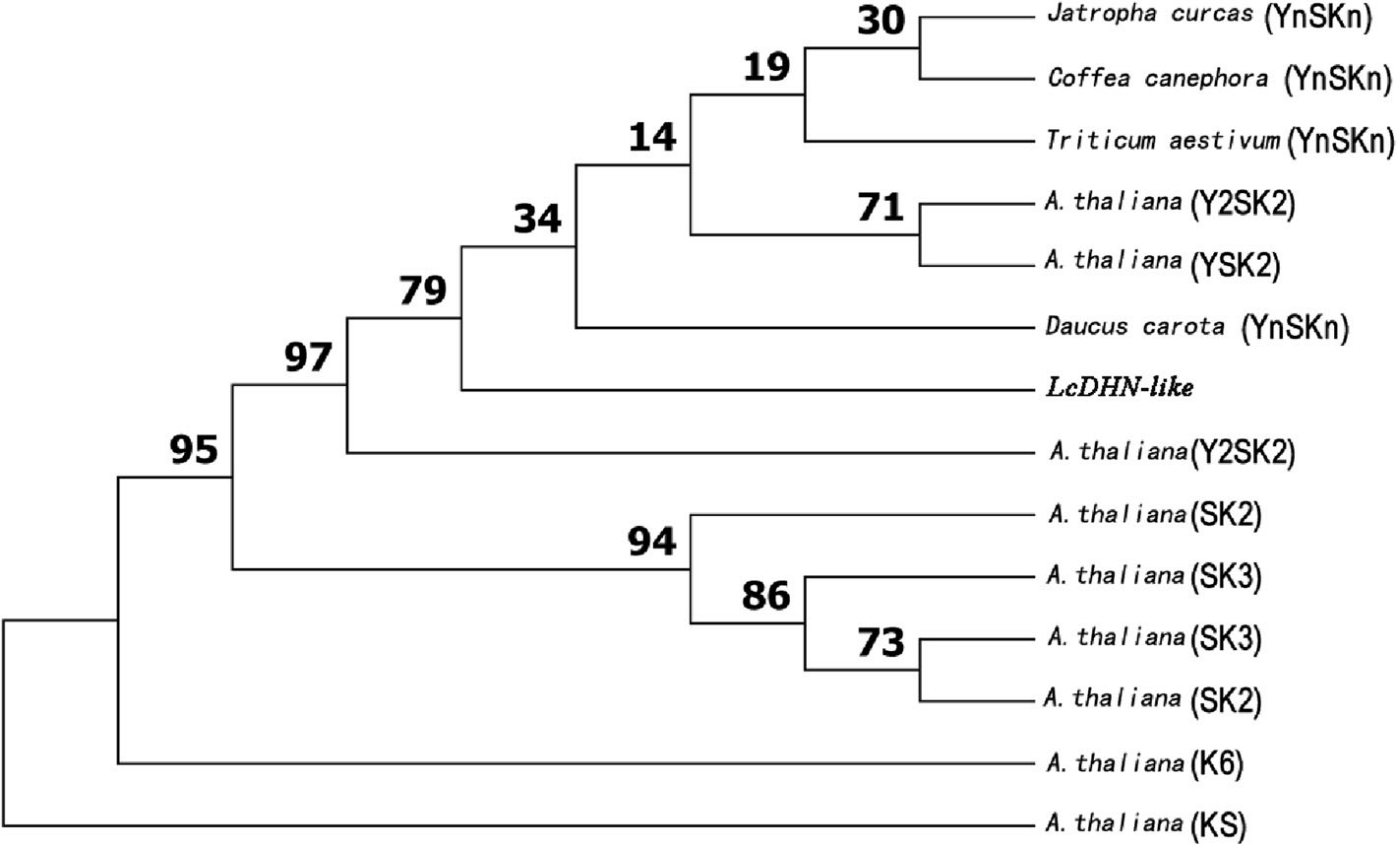
The UPGMA tree based on LcDHN-like protein and 13 homologous DHN proteins from other plants. Note: 1. The bootst rapping was set 1000 replicates and bootstrap values were labeled beside the branches. 2. *Jatropha curcas*: NP_001295638; *Coffea canephora*: ABC55671; *Daucus carota*: BAD86644; *Triticum aestivum*: AOM63238; *A. thaliana*: NP_201441; *A. thaliana*: NP_190667; *A. thaliana*: NP_179744; *A. thaliana*: NP_195554; *A. thaliana*: NP_177745; *A. thaliana*: NP_564114; *A. thaliana*: NP_173468; *A. thaliana*: NP_190666; *A. thaliana*: NP_175843

A 707 bp full-length cDNA was assembled with the 468 bp 5’sequence and 423 bp 3’sequence (Additional file 2). The prediction and experimental verification proved that *LcDHN-like* gene contained a 477 bp open reading frame (ORF), which encoded a 158 amino acids polypeptide (Fig. 1).The coding region was presented in the genomic sequence to analyze the gDNA structure. *LcDHN-like* gene was composed of two exons (186 bp and 291 bp), which were separated by one 464 bp intron (Fig. 1). Moreover, a 21 bp 5’Untranslated Region (UTR) and a 110 bp 3’ UTR were obtained by the ORF primers (Fig. 1).The intron of *LcDHN-like* gene falls to obey the GT -AG rule of exon - intron borders [51]. ORF and amino acids sequences were used to blast against Nucleotide collection (nr/nt) database from Genebank, which indicated it is a homolog of *DHN*. We then named it *LcDHN-like*. The deduced protein product possesses 2 Y-segments, 1S-segment, and 2 K-segments, suggesting it is Y_n_SK_n_ –type DHN protein (Fig. 1). This result was further confirmed by a phylogenetic analysis that showed LcDHN-like protein cluseted into the Y_n_SK_n_ subgroup, including homologs of Y_n_SK_n_ –type proteins from *Arabidopsis thaliana*, *Daucus carota*, *Triticum aestivum*, *Jatropha curcas*, and *Coffea canephora* (Fig. 2) [52–55].

### DNA diversity in *Liriodendron* populations

**Fig. 3:**
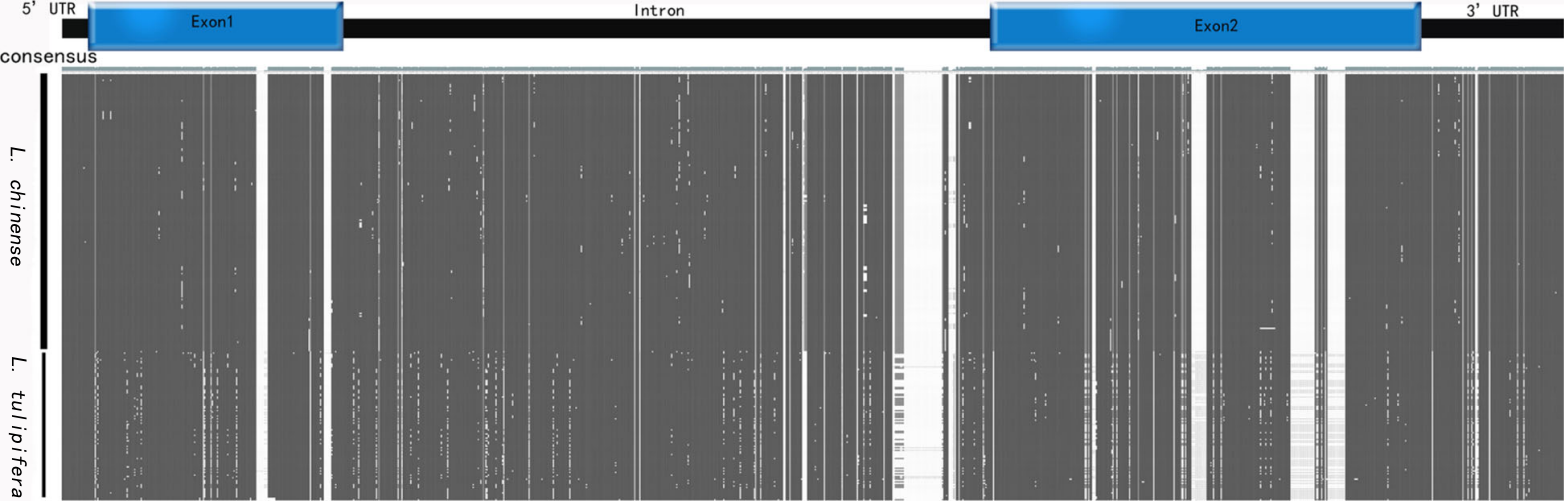
Overall SNPs and Indels of *LcDHN-like* gene in *Liriodendron* populations. Note: The white lines with higher consensus represent SNPs and the white lines with lower consensus represent Indels

311 *LcDHN-like* gDNA sequences were obtained from 70 *L. chinense* and 35 *L. tulipifera* individuals representing 27 populations (Additional file 3). After alignment, in total, 233 SNPs (19.73%) and 152 indels (12.87%) were found in 1181 bp nucleotide positions (Table 2). Of the 233 SNPs, 102,113, and 18 SNPs were located in exon, intron, and UTR region, respectively (Table 2, Fig. 3). At a whole population level, the nucleotide diversity, π was 0.02881 and θw was 0.03585. π was observed as 0.02890 and 0.03030, as well as θw was 0.03542 and 0.04021, in the coding and intron regions, respectively.

**Table 2:** The nucleotide diversity of *LcDHN-like* gene in *Liriodendron* populations

		Exon-1	Exon-2	Exon	5′UTR	Intron	3′UTR	Total
*Liriodendron*	Size	201	339	540	21	508	112	1181
	SNPs	42	60	102	2	113	16	233
	Indels	21	63	84	0	63	5	152
	π	0.02529	0.03125	0.02890	0.00061	0.03030	0.02776	0.02881
	θw	0.03695	0.03442	0.03542	0.01508	0.04021	0.02368	0.03585
*L. chinense*	Size	186	291	477	21	474	110	1082
	SNPs	12	18	30	2	55	5	92
	Indels	0	12	12	0	17	1	30
	π	0.00573	0.00640	0.00613	0.00094	0.01410	0.00964	0.00986
	θw	0.01097	0.01097	0.01097	0.01619	0.02046	0.00780	0.01487
*L. tulipifera*	Size	201	339	540	21	506	111	1178
	SNPs	32	44	76	0	75	10	161
	Indels	21	51	72	0	54	3	129
	π	0.03237	0.03757	0.03891	0	0.03473	0.02572	0.03348
	θw	0.03377	0.02902	0.03085	0	0.03152	0.01759	0.02916

*SNPs* single nucleotide polymorphism sites, *Indels* insertion or deletion sites, π nucleotide diversity, *θw* theta (per site) from S

Table 2 showed that 92SNPs (8.50%) and 30 indels (2.77%) were found in the populations of *L. chinense*. Of the 92 SNPs, 30 SNPs exist in exon (12 SNPs in exon1, 18 SNPs in exon2), 55 in intron, and 7 in UTR. The π for the whole sequence, exon and intron are 0.00986, 0.00613, and 0.01410, respectively. The θw for the whole sequence, exon and intron are 0.01487, 0.01097, and 0.02046 respectively. In general, the intron does not have clear function and evolving under neutral with higher polymorphism. Higher selection effect in exon region leads to its lower polymorphism and stronger conservation.

161SNPs (13.67%) and 129 indels (10.95%) were found in *L. tulipifera* populations, including76 in the exon (32 in exon1, 44 in exon2), 75 in intron (Table 2), and 10 in the 3’ UTR. No SNPs were discovered in the 5’ UTR. The π for the whole sequence, exon and intron are 0.03348, 0.03891, and 0.03473, respectively. The θw for the whole sequence, exon and intron are 0.02916, 0.03085 and 0.03152, respectively. These results indicate that *L. tulipifera* populations possess much higher level of DNA polymorphism compared to the *L. chinense.*

### Neutrality tests

We then evaluated the evolutionary selection dynamic of *LcDHN-like* gene using four neutrality tests (dN/dS, Tajima’s D, Fu and Li’s D*, and Fu and Li’s F*). In all studied populations, dN/dS ratio was found to be significantly less than 1.Tajima’s D, Fu and Li’s D* and Fu and Li’s F* were − 0.81939, − 2.64830, and − 2.01595, respectively. Only Fu and Li’s D* showed significant (Table 3). Of four neutrality tests, only dN/dS ratio showed significance within the *L. chinense* populations (Table 3). However, in the *L. tulipifera* populations, all four neutrality tests showed no significance, suggesting the *LcDHN-like* gene does not reject the neutral mutation hypothesis (Table 3).

**Table 3:** Neutrality tests values of *LcDHN-like* gene based on nucleotide variation

	dN/dS ratio	Tajima’s D	Fu & Li’s D*	Fu & Li’s F*	
*Liriodendron*	0.18166^*^	−0.81939	−2.64830*	−2.01595	
*L. chinense*	0.06065^*^	−1.12616	−0.42516	− 0.89998	
*L. tulipifera*	0.32699	0.24844	−1.88595	−1.14004	

^*^significant; *P* < 0.05

### Molecular variance analysis between *L. chinense* and *L. tulipifera* populations

To survive in continuously changes of environment, *Liriodendron* has adapted to a number of distinct environmental conditions. We then performed AMOVA analysis to evaluate more in detail about the differences of *LcDHN-like* gene interspecies (Table 4). The results indicate that most of the variation (61.82%) contributed by the difference between the two species with a highly significant F_SC_ value (0.17) (Table 4). Within species, the variation was contributed mainly by the difference among the individuals (31.78%) and less by populations (6.40%) (Table 4).

**Table 4:** Analysis of molecular variance of *LcDHN-like* gene of *Liriodendron* populations

Source of variation	Degrees of freedom	Sum of squares	Variance components	Percentage variation	Fixation Indices	*P*-Value
Among species	1	3862.12	26.87	61.82	F_SC_ = 0.17	< 0.001^*^
Within species among populations	25	1130.85	2.78	6.40	F_ST_ = 0.68	< 0.001^*^
Within populations	284	3921.82	13.81	31.78	F_CT_ = 0.62	< 0.001^*^

^*^indicates that Fixation Indices showed significant difference when significance tests (1023 permutations) were at *P* < 0.001 level

**Fig. 4:**
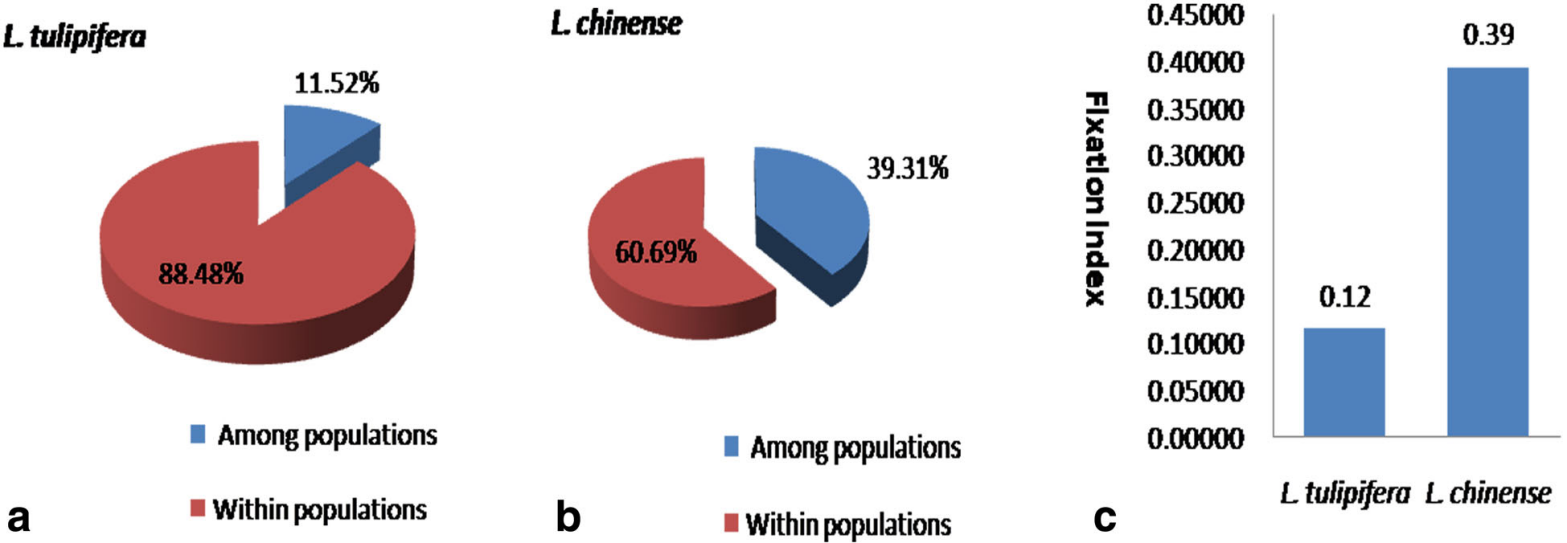
Molecular variance analysis of LcDHN-like gene in *L. chinense* and *L. tulipifera*. **a** The percentage variation among and within *L. chinense* populations. **b** The percentage variation among and within *L. tulipifera* populations. **c** The fixation indices of *L. chinense* and *L. tulipifera* populations

AMOVA analysis within the species showed that the variation stored at population level in *L.chinense* (39.31%) is bigger than *L. tulipifera* (11.52%) (Fig. 4a, Fig. 4b). However, at individual level, the total variation in *L. chinense* (60.69%) is lower than *L. tulipifera* (88.48%) (Fig. 4a, b). Consistently, the F_ST_ value in *L. chinense* populations (0.39) is bigger than *L. tulipifera* (0.12) (Fig. 4c). These results suggested that *L. tulipifera* possesses higher genetic diversities at an individual level but less genetic differentiation among the populations compared to that of *L. chinense.*

### Two distinct lineages in *Liriodendron*

**Fig. 5:**
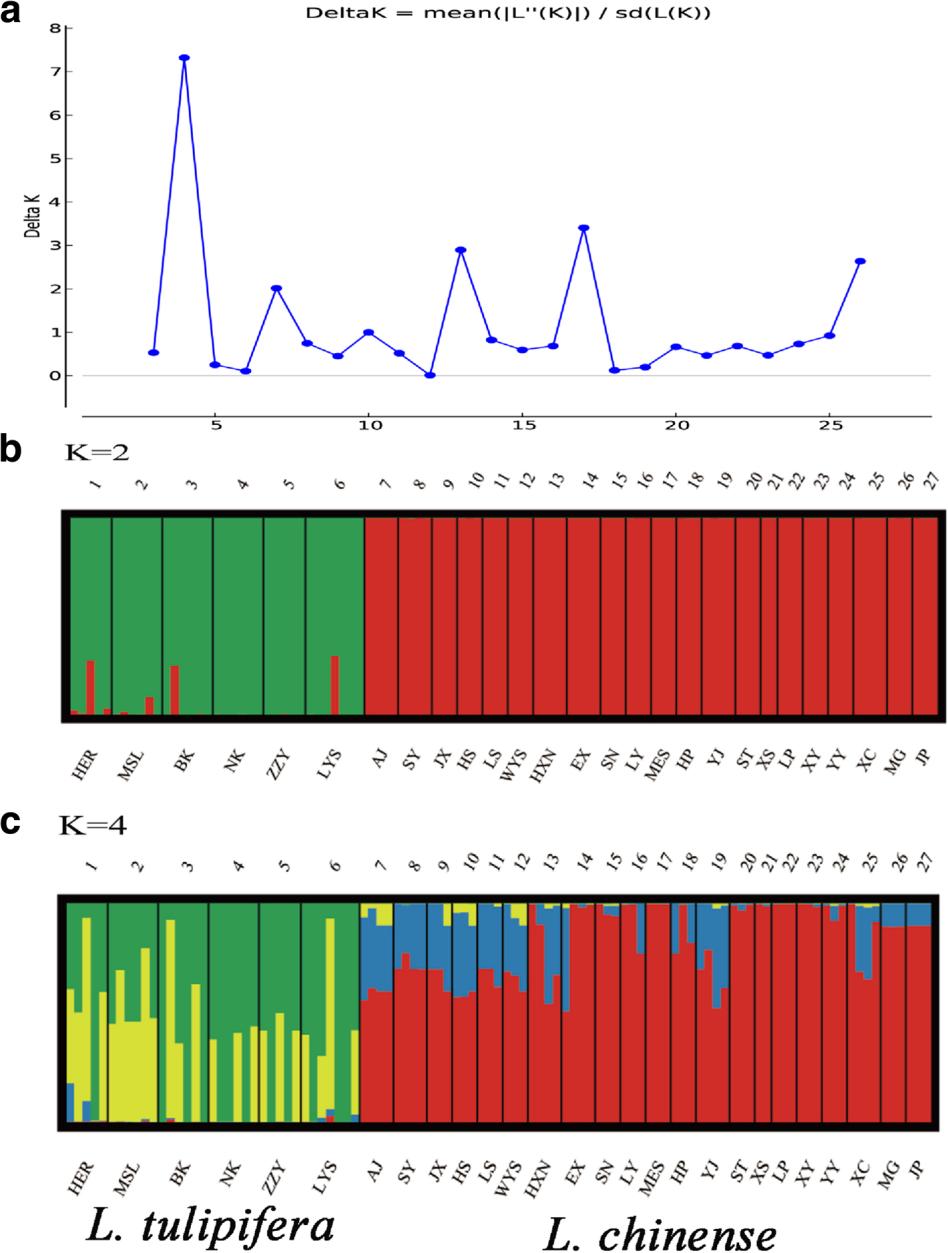
Population structure of *Liriodendron* populations. **a** The StructureHarvester result of relationships between ΔK and K. **b** the clusters result of K = 2. **c** the clusters result of K = 4

**Fig. 6:**
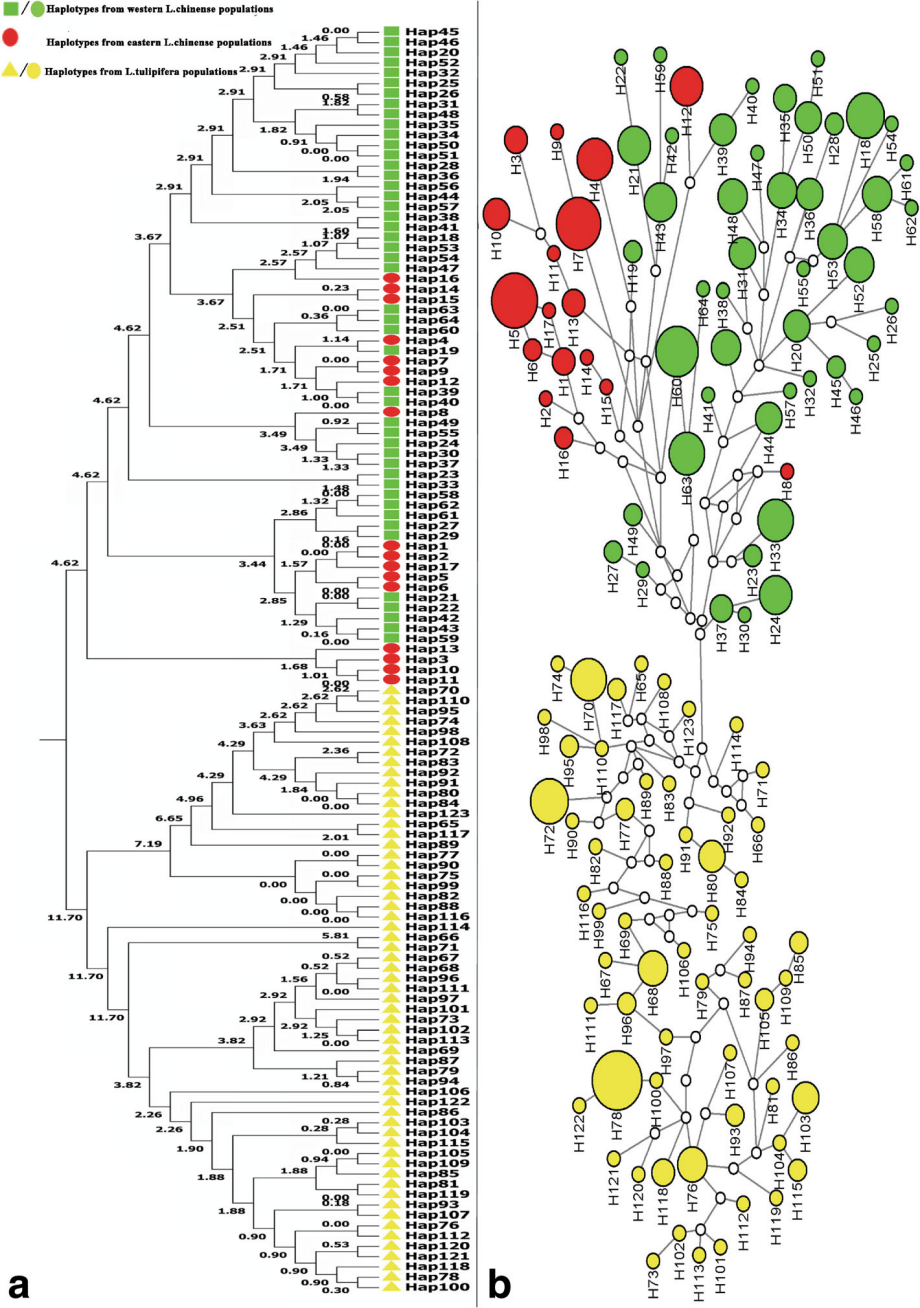
Phylogenetic relationship based on 123 haplotypes of *LcDHN-like* gene in *Liriodendron* populations. Green squares/ circles represent haplotypes from western *L. chinense* populations; Red circles represent haplotypes from eastern *L. chinense* populations;Yellow triangles/ circles represent haplotypes from *L. tulipifera* populations. **a** The RelTime UPGMA tree. Divergence times (Ma) for all branching points were labeled. **b** Phylogenetic network. Sizes of nodes are the number of haplotypes: *n* = 1 for small nodes, *n* ≥ 2 for otherwise; the white dots represent median vectors

A total of 123 haplotypes were defined by 233 SNPs, including 64 from *L. chinense* and 59 from *L.tulipifera*. The two species dependent were clustered into two separated clades in the UPGMA phylogenetic tree and network analysis (Fig. 6). Though K = 4 was considered as the most adequate number of clusters, all *Liriodendron* populations were divided into 2 major genetic clusters: *L. tulipifera* (green) and *L. chinense* (red) (Fig. 5). Six specific SNPs were identified for distinguished *L. chinense* and *L. tulipifera*, including 3 in exons, 2 in intron, and 1 in 5’UTR (Table 5).

**Table 5:** The completely separated positions between *L. chinense* and *L. tulipifera* populations

region	position	Variation	*L. chinense*	*L. tulipifera*	Codon	Amino acid	Effect
Exon-2	733	C → T	C	T	GAC → GAT	Asp→Asp	SC
	859	T → C	T	C	GGC → GGT	Gly → Gly	SC
	875	C → A	C	A	CCA/CAA → ACA	Pro/Gln → Thr	NSC
Intron	647	A → G	A	G			
	703	-/C → T/G	-/C	T/G			
5′UTR	1078	T → C	T	C			

*NSC* nonsynonymous change, *SC* synonymous change

For *L. chinense* populations, the most frequent frequencies of haplotypes were H5, H7, H60 and H18, which were observed 12, 11, 10 and 8 times with frequencies of 5.94, 5.45, 4.95 and 3.96%, respectively. While H (63, 33, 4), H (43, 24, 21, 12), H (58, 56, 53, 52, 48, 34), H (50, 44, 39, 36, 31, 20, 10), H (37, 35, 13, 3, 1) and H (49, 45, 28, 27, 23, 19, 16, H6) were happened 7, 6, 5, 4, 3, 2 times with frequencies of 3.47, 2.97, 2.48, 1.98, 1.49 and 0.99%, respectively. The other haplotypes were uniqe with a frequence of 0.50%. For *L. tulipifera* populations, the most frequent frequencies of haplotypes were H78, H72 and H70, which were observed 14, 8 and 7 times with frequencies of 12.84, 7.34 and 6.42% respectively. While H (76, 68), H (103, 80), H118 and H (117, 115, 105, 96, 95, 93, 85, 77) were happened 5, 4, 3 and 2 times with frequencies of 4.59, 3.67, 2.75 and 1.83%, respectively. The other haplotypes were uniqe with a frequence of 0.92%. The numerous haplotypes with relative lower frequencies were corresponding with the higher haplotypes diversity of studied *Liriodendron* individuals (0.9870). Both of the two species’ lineage networks contained multiple clades, indicating a complex relationship patterns between populations (Fig. 6). In *L. chinense*, the eastern populations, as a big clade, separated from western populations, while in *L. tulipifera*, no obvious intraspecies phylogeographic pattern was found (Fig. 6b).

### Haplotypes geographic distribution of *Liriodendron*

**Fig. 7:**
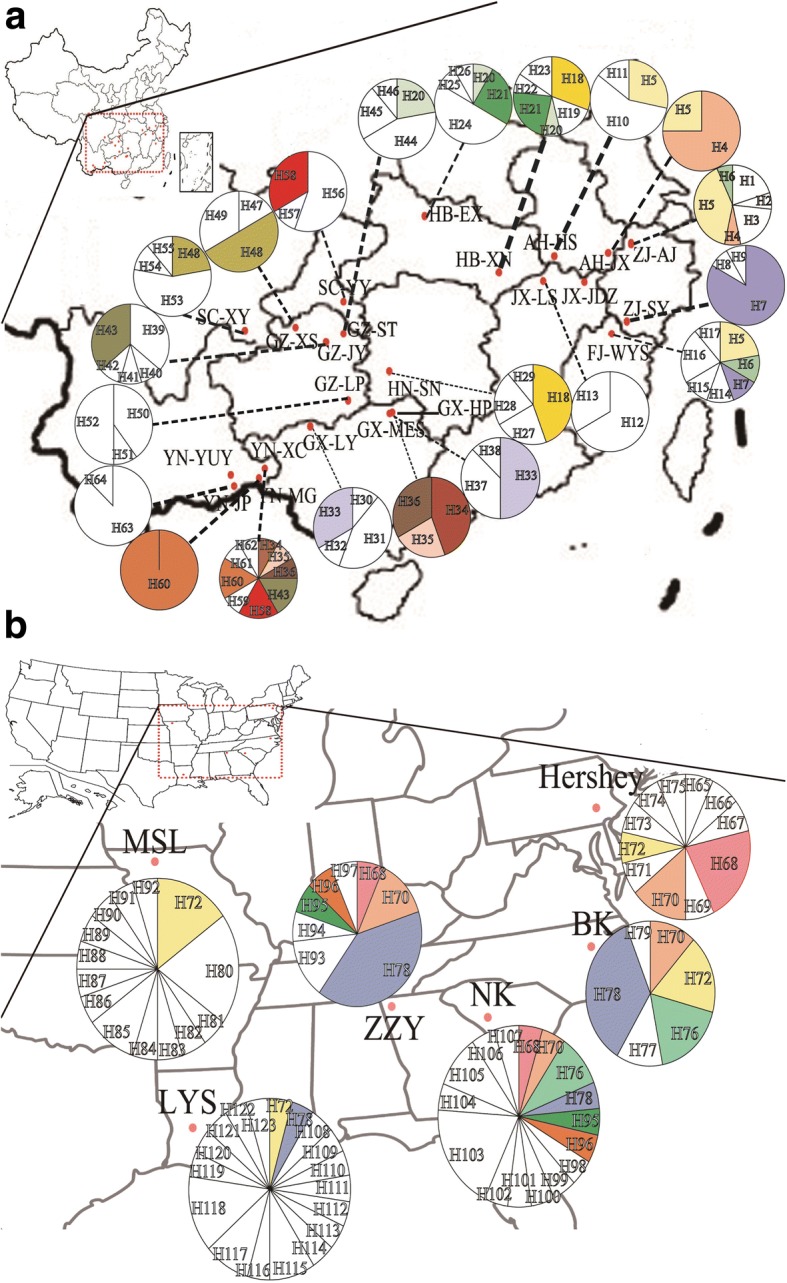
Geographic distribution of *LcDHN-like* haplotypes about *Liriodendron*. The white parts of circles represent the unshared haplotypes, the circles with same colour represent they share same haplotypes; the divided area of each circle corresponds to the frequency of each haplotype. The maps of CHN and USA were downloaded from https://commons.wikimedia.org/wiki/File:China-map.xcf and https://upload.wikimedia.org/wikipedia/commons/c/ca/Blank_US_map_borders.svg, separately. **a** The haplotypes distribution of *L. chinense* in China. **b** The haplotypes distribution of *L. tulipifera* in America

For *L. chinense*, haplotypes H (4, 6, 7, 18, 21, 33, 34, 35, 36, 43, 48, 58, 60), H20 and H5, as shared haplotypes, were only found in nearby 2, 3 and 4 populations respectively (Fig. 7). For *L.tulipifera*, H (76, 95, 96), H68 and H (70, 72, 78), as common and dispersed haplotypes, were presented in 2, 3 and 4 populations respectively (Fig. 7). The remaining haplotypes for both *L. chinense* and *L. tulipifera* were restricted in a single population (Fig. 7). Geographic distribution of haplotypes suggested that all populations contained their own particular haplotypes. Although existing shared haplotypes with higher frequencies, no prominent central and ancestral haplotypes were found in gene genealogies of *L. chinense* and *L. tulipifera* populations.

### The analysis of divergences times and demographic expansion in *Liriodendron*

As a Timetree, the UPGMA phylogenetic tree also showed the divergent time of haplotypes in *Liriodendron* populations, in which the earliest haplotypes divergence time were approximately4.62 and 11.7 Ma ago in *L. chinense* and *L. tulipifera*, respectively (Fig. 6a).The completely separated western haplotypes in *L. chinense* were happened about 3.67 Ma ago.

The values of SSD/H_Rag_ were 0.205067/0.480071 and 0.480071/ 0.436351 with nonsignificant *p*-value in both *L. chinense* and *L. tulipifera* populations, which supported a recently demographic expansion in these two species. In view of the tau value (τ) and the mutation rates of *LcDHN-like* gene (2.0 × 10^− 8^ per year), we deduced that 0.263 Ma and 2.02 Ma ago would be the time of recent population expansion to *L. chinense* and *L. tulipifera* populations respectively.

### The demographic history of *Liriodendron*

**Fig. 8:**
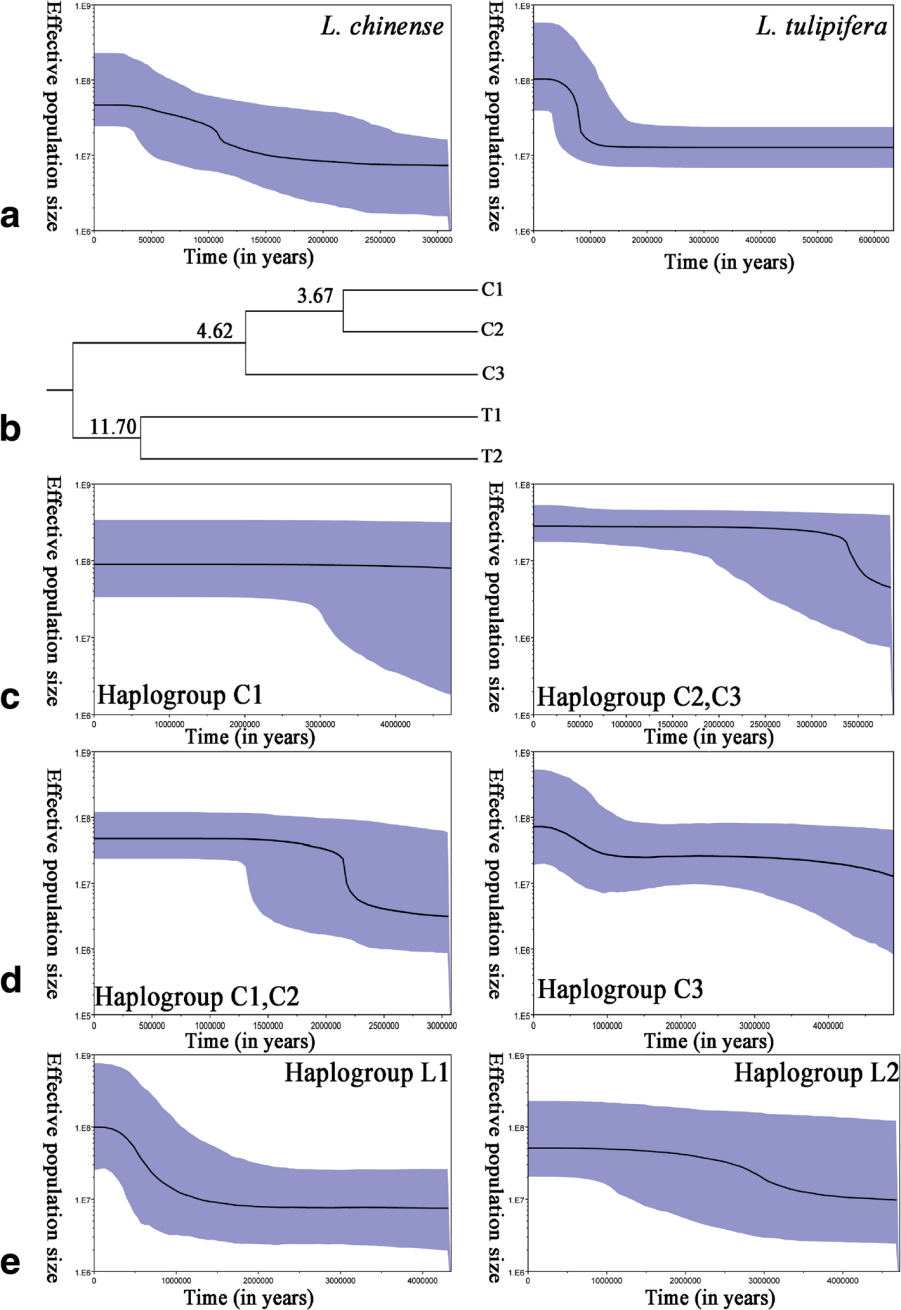
Bayesian Skyline plots. The Black lines represent median population estimates over time and blue lines represent 95% confidence intervals surrounding those medians. **a** BSP of *L. chinense* and *L. tulipifera* populations. **b** Sketchy phylogenetic tree. Divergence times (Ma) were labeled on the branches. **c** BSP of haplogroup C1; C(2, 3). **d** BSP of haplogroup C(1, 2) and C3. **e** BSP of haplogroup T1 and T2

As showed in the UPGMA phylogenetic tree (Fig. 8b), five main haplogroups, C1, C2, C3; T1, T2, were obviously grouped on account of separated time nodes. Haplogroup C1 was found in most western populations except Jingping and Maguang from Yunnan. Though haplogroup C2 contained populations from eastern and western China, it didn’t present at Songtao, Liping from Guizhou; Leye, Maoershan, Huaping from Guangxi, Sichuang-Youyang, Hubei-Exi and Anhui-Huangshan. The haplogroup C3, a widely distributed haplogroup across southern China from east to west, presented in most populations except four populations as Songtao, Liping, Maoershan, Jingping, and Maguan. Haplogroup T1 and T2 were both common in North America, while T2 included more numerous haplotypes. We performed BSPs for the total populations of *L. chinense* and *L. tulipifera* and haplogroup C1, C(2, 3); C(1, 2); C3; T1; T2 to detect their changes in effective population size over time.

The Bayesian skyline plot (Fig. 8a) indicated that both *L. chinense* and *L tulipifera* populations experienced demographic expansions around 0.5 to 1.5 Ma ago, Pleistocene, which were consistent with the results of pairwise mismatch distributions analysis. Figure 8a also showed that the effective population size of *L. chinense* was smaller than that of *L. tulipifera* in the beginning, followed by a slow and rapid growth in both species, and finally, the effective population size of *L. tulipifera* increased much higher than that of *L. chinense*. Thus, we deduced that the population expanding rate of *L. tulipifera* was rapider than that of *L.chinense*. The haplogroup C1 did not undergo large fluctuations in population size, whereas a marked increase of effective population size in haplogroup C(2, 3) was estimated about 3.3 Ma ago (Fig. 8c). Haplogroup C(1, 2) and C3 were severally experienced population expansion during 2–2.25 Ma and 0–1 Ma respectively, however, no obvious change of population size was found about 4 Ma ago in haplogroup C3 (Fig. 8d). Figure 8e showed that haplogroup T1 kept a long history of nearly constant population size, before a recent population expansion over the last 0.3–1.0 Ma. A slow population growth during 2.5–3.5 Ma was observed in haplogroup T2 (Fig. 8e).

### The variations of LcDHN-like proteins

Fifty-eight LcDHN-like protein isoforms were identified from the studied populations, which derived from non-synonymous SNPs or Indels. There are17 and 41 specific isoforms for *L. chinense* and *L. tulipifera*, repectively (Additional file 4).In the studied populations, the LcDHN-like proteins ranges from 154 to175 AA length, resulting molecular weight range between 15.94 and18.00 kDa (Additional file 1: Table S2).Generally, LcDHN-like proteins in *L. tulipifera* are slightly bigger than that of *L. chinense*. The hydrophilic index ranges from 32.66 to 39.63, in which *L. chinense* contributed more to the lower part of this range (32.66–36.33) and *L.tulipifera* more to the higher part (32.99–39.63).

## Discussion

### The polymorphism of *LcDHN-like* gene in *L. chinense* and *L. tulipifera* populations

In this study, we investigated the *LcDHN-like* gene polymorphisms from 21 *L. chinense* populations and 6 *L. tulipifera* populations. Interestingly, we observed much higher genetic diversity in *L. tulipifera* populations which was sampled from a region with a smaller scale of Appalachian uplands and the southeastern coastal plains, compared to *L. chinense* (Tables 1 and 2). These results are consistent with previous studies based on expressed sequence tag derived SSR (EST-SSRs) markers and RAPD markers [56, 57]. Moreover, this precise survey of diversity at the nucleotide level provided a snapshot of the *LcDHN-like* gene evolution and represented its reservoir of genetic diversity for short-term (ecological) and/or long-term (evolutionary) adaptation [58–61]. Therefore, our data suggest that *L. tulipifera* possesses a higher genetic diversity which might result in a stronger adaptation and resilience to the unforeseen environmental changes.

### The selection patterns of *LcDHN-like* gene in *L. chinense* and *L. tulipifera* populations

**Fig. 9:**
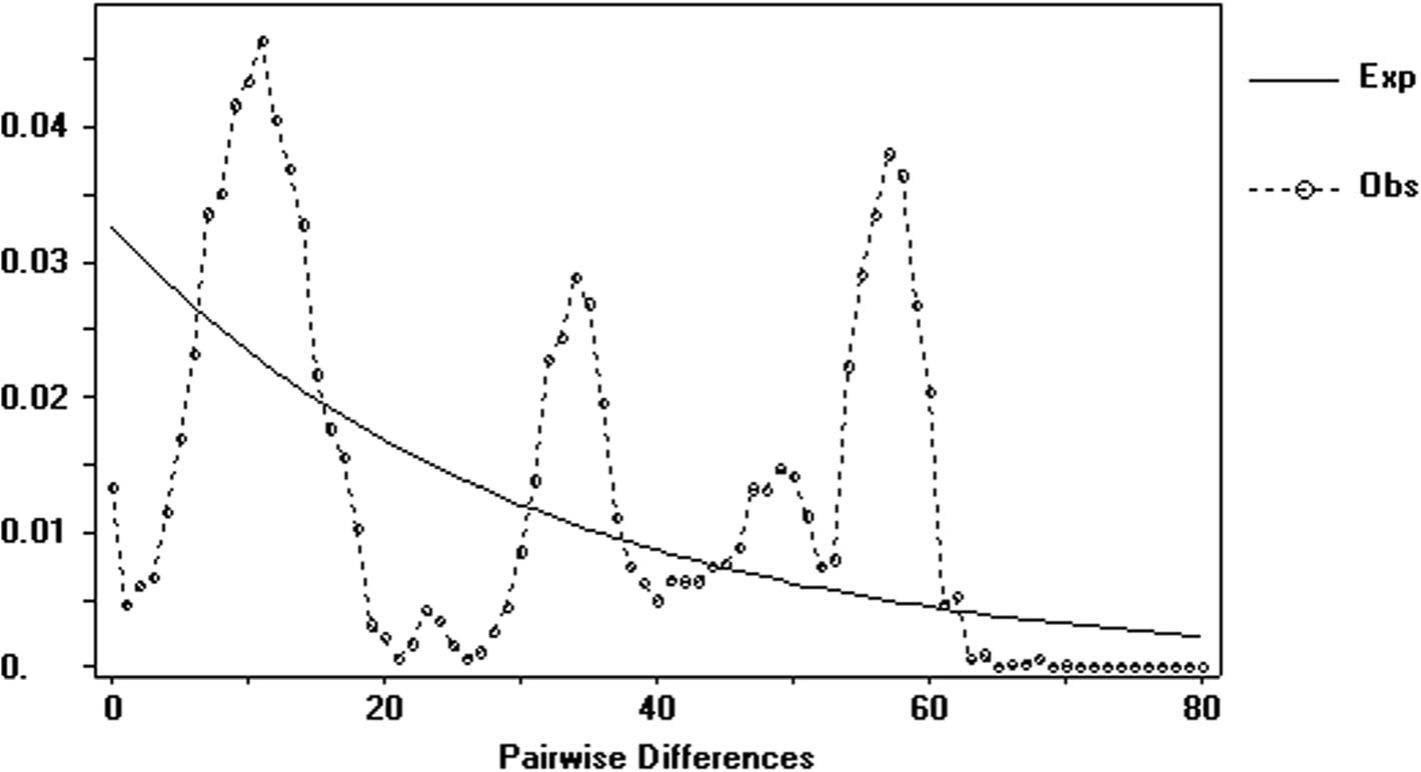
Mismatch distribution of the *Liriodendron* populations. Note: The line represents expected distributions of expanding population; the dotted line represents observed mismatch distribution from variation sites of *LcDHN-like* gene in *Liriodendron* populations

All the studied 27*Liriodendron* or 21 *L. chinense* populations showed dN/dS values that were significantly less than unity, suggesting that this gene was under purifying selection in these populations [32, 62]. The significant negative value of Fu and Li’s D* may indicate events of expansion or selection of the *Liriodendron* populations during the evolution [34]. The mismatches distribution normally exhibits a Poisson distribution in expanding populations, while a variety of geometric distributions were often observed for populations with constant size [63]. The distribution we observed for *LcDHN-like* gene mismatches confirmed that the studied *Liriodendron* populations are under purifying selection (Fig. 9). Taken together, these results indicated that a natural selection is occurring to suppress LcDHN protein mutations and reduce the nucleotide diversity [64].

Intriguingly, no significance was detected in the neutrality tests for *L. tulipifera* populations, indicating that the gene in this species was under the neutral mutation hypothesis. The neutral theory thought that most evolutionary changes at the molecular level are caused by natural drift of selectively neutral or nearly neutral mutations rather than by natural selection [65]. Therefore, these results suggested that *LcDHN-like* gene in *L. tulipifera* currently are not under a natural selection. On the contrary, *L. chinense* is under a purifying selection resulting in its lower genetic diversity [66].

### The genetic structure difference between *L. chinense* and *L. tulipifera* populations

The AMOVA analysis has shown that the greatest variation of *LcDHN-like* gene sequence in *Liriodendron* populations came from interspecies, which presumably caused by their long time geographic separation. *L. chinense* and *L. tulipifera* were separated about 10–16 Ma ago according to the molecular and paleobotanical evidences, which indicated restricted gene flow with isolation by distance [43]. Fossil floras data indicated that *Liriodendron* may not survived in Beringia after the late Miocene [43]. And the Atlantic Ocean broke the North American-European connection [67]. These two events resulted in a breakage of gene flow between North American and Asian. In summary, the significantly variation between *L. chinense* and *L. tulipifera* populations, which is consistent with completely distinct phylogenetic relationship, were the result of distance and evolution under different environment for a long time.

*L.chinense* showed greater variation than *L. tulipifera* at population level and lower variation at individual level. *L. tulipifera* populations distribute more widely, causing possibly higher gene flow, which could decrease variation among populations [1, 10].On the contrary, the small, scattered and isolated *L. chinense* populations are more likely facing barriers of gene flow, which could increase the variation among populations [1].

### The distinct expanding range between *L. chinense* and *L. tulipifera* populations

The same lineages of *L. chinense* and *L. tulipifera*’s populations could contribute to the ambiguous phylogeographic patterns detected in the two species. And the shared haplotypes related to demographic expansion in Pleistocene also facilitated the unordered phylogeographic patterns. Unlike the disordered haplotypes of *L. tulipifera*, the eastern populations of *L. chinense* separated from western population as a big clade in the haplotypes networks and coincided with the biogeographic districts described by Hao and He [68, 69]. The distinct expanding range, haplotypes shared only in nearby *L. chinense* populations while wide dispersals in *L. tulipifera* populations could contribute to the obscure east and west separation in *L. chinense* and entirely unordered phylogeny in *L. tulipifera*.

### The haplotypes separation and diverse demography history between *L. chinense* and *L. tulipifera*

The completely separated haplotypes and the distinguished mutation sites between species have been proposed to be the consequence of their apart evolution under different environments for a long time [43, 70]. Thus, climatic oscillations and past geology were important to understand their demography and evolution history [70, 71]. Hereinafter, we took the inferred haplogroups patterns, together with their divergence time in *L. chinense* and *L. tulipifera* and relevant paleo-climatic fluctuations for further discussing.

Our phylogenetic analysis suggested that the earliest divergence time among haplotypes in *L. tulipifera* was occurred about 11.7 Ma ago, during the late Miocene (Fig. 6a). This event might be the consequence of southward migrating Arctic air masses during the late Miocene, which led to the warm temperate mesophytic vegetation arise range restriction [43]. The separated haplogroups T1 and T2 expanded during 0.3–1.0 Ma and 2.5–3.5 Ma with different growth trend, which could contribute to the further haplotypes divergence of Quaternary period (Fig. 6a, Fig. 8e). In *L. chinense*, the earliest divergence time of haplotypes was approximately 4.62 Ma ago. Considering that no apparent population changes were found 4 Ma ago in haplogroup C3, we deduced that the divergence of haplotypes might be the result of continued cold environment during the late tertiary [72, 73]. The earlier haplotypes divergence event in *L. tulipifera* might reflect the earlier adaption to cold environment, which contributed to the stronger cold tolerance of *L. tulipifera* when compared to *L. chinense*.

The completely separated western haplotypes in *L. chinense* occurred about 3.67 Ma ago, which coincided with the rapid expansion signal in haplogroup C(2, 3) about 3.3 Ma ago (Fig. 8c). And the warm period in Northern Hemisphere during Middle Pliocene (~ 3 Ma) also could stimulate the expansion of haplogroup C(2, 3) [74]. The nearly identical time of separation and expansion might reflect the founder population of haplogroup C(2, 3) from which the lineages C1 arose [75].

### The LcDHN-like protein variation between *L. chinense* and *L. tulipifera* populations

LcDHN-like protein (15.94–18.00 kDa) could be classified as a stable DHN protein ranging from 9kD to 200kD [21]. The DHNs are part of the intrinsically disordered proteins expressed under conditions of water-related stress [23].Hydrophilic properties reinforce the flexibility and the disorder aspect that can be conferred to LcDHN-like protein [76].The lower hydrophilic indices observed in *L. chinense* suggested potential stronger abiotic stresses resistance of LcDHN-like proteins in *L. tulipifera* compared to *L. chinense.* The observed nonsynonymous nucleotides substitutions might affect LcDHN-like proteins’ function and result in adaption difference between *L. chinense* and *L. tulipifera*, which would be very interesting to investigate further in the future [77].

## Conclusions

In this paper, all of the investigated results from AMOVA, population structure and phylogenetic relationship provided a sharp phylogeographic break between *L. chinense* and *L.tulipifera* populations. We also provided comprehensive and important insights into the degree of interspecies evolutionary divergence by analyzing DNA polymorphism difference, evolution dynamics, molecular variation and haplotypes of *LcDHN-like* gene between the two species. The distinct expanding range and rate between the two species, haplotypes shared only in *L.chinense*’s nearby populations while widespread in *L. tulipifera*, could contribute to the unclear east-west separation detected in *L.chinense*’s network tree and unordered phylogeographic pattern in *L. tulipifera*. And the more widely distribution, earlier haplotypes divergence events and richer SNPs variations in *L. tulipifera* could imply their stronger adaptation.

## Additional files

Additional file 1: **Table S1.** Primers and PCR protocol for RACE amplification and ORF testing. **Table S2.** Characteristics of properties and structure about LcDHN-like proteins in *Liriodendron.* P1-P17, proteins from *L. chinense*; P18-P58, proteins from *L. tulipifera*. (DOCX 27 kb)

Additional file 2: 5’ RACE, 3’ RACE and cDNA sequences of *LcDHN-like* gene. (DOCX 15 kb)

Additional file 3: 311 *LcDHN-like* gDNA sequences in *Liriodendron*. (DOCX 152 kb)

Additional file 4: The list of 58 predicted proteins in *Liriodendron*. (DOCX 19 kb)

## Abbreviations

dN: Number of non-synonymous substitutions per non-synonymous site
dS: Number of synonymous substitutions per synonymous site
F_ST_: Fixation index
Indels: Insertion/Deletion sites
SNPs: Single nucleotide polymorphisms
θ_W_: Scaled measure of the number of polymorphic nucleotide sites per nucleotide
π: Nucleotide diversity
