# Supplementary material for: Interspecies evolutionary divergence in Liriodendron, evidence from the nucleotide variations of LcDHN-like gene
Source: BMC Evol Biol. 2018 Dec 19;18:195. doi: 10.1186/s12862-018-1318-7 (PMC6300021; doi:10.1186/s12862-018-1318-7)
Supplement: Supplementary file 1 — Table S1. Primers and PCR protocol for RACE amplification and ORF testing. Table S2. Characteristics of properties and structure about LcDHN-like proteins in Liriodendron. P1-P17, proteins from L. chinense; P18-P58, proteins from L. tulipifera. (DOCX 27 kb) [file 12862_2018_1318_MOESM1_ESM.docx]

**Additional file 1**

| **[Table S1](http://xueshu.baidu.com/s?wd=paperuri%3A%280c438fcb12f2e6193d3c8297d09c71b2%29&filter=sc_long_sign&tn=SE_xueshusource_2kduw22v&sc_vurl=http%3A%2F%2Fciteseerx.ist.psu.edu%2Fviewdoc%2Fdownload%3Fdoi%3D10.1.1.190.9540%26rep%3Drep1%26type%3Dpdf&ie=utf-8&sc_us=14537036281601669358): Primers and PCR protocol for RACE amplification and ORF testing** | | |
| --- | --- | --- |
| Primer name | Primer sequence（5′-3′） | The PCR protocol |
| 3’RACE outer primer F | CGTGATGAGTATGGCAACCAG | 94℃, 3min; 35cycles of 94℃ for 30s, 55℃ for 30s, 72℃ for 30s;  72℃ extending for10min |
| 3’RACE outer primer R | TACCGTCGTTCCACTAGTGATTT |  |
| 3’RACE inner primer F | CGTAGGAAGAAGGGTTTGAC | 94℃, 3min; 35cycles of 94℃ for 30s, 55℃ for 30s, 72℃ for 30s;  72℃ extending for10min |
| 3’RACE inner primer R | CGCGGATCCTCCACTAGTGATTTCACTATAGG |  |
| 5’RACE outer primer F | CATGGCTACATGCTGACAGCCTA | 94℃, 3min; 35cycles of 94℃ for 30s, 52℃ for 30s, 72℃ for 30s;  72℃ extending for10min |
| 5’RACE outer primer R | GGTGGACACACATCCTACTTG |  |
| 5’RACE inner primer F | CGCGGATCCACAGCCTACTGATGATCAGTCGATG | 94℃, 3min; 35cycles of 94℃ for 30s, 54℃ for 30s, 72℃ for 30s;  72℃ extending for10min |
| 5’RACE inner primer R | TCCCTGGATGACCGAAACC |  |
| ORF primer F | AGCCGTTGGATCTAGTTGAA | 94℃, 3min; 35cycles of 94℃ for 30s, 52℃ for 30s, 72℃ for 30s;  72℃ extending for10min |
| ORF primer R | GACATAACAGATACGCGCAGA |  |
|  |  |  |

| [**Table S2:**](http://xueshu.baidu.com/s?wd=paperuri%3A%280c438fcb12f2e6193d3c8297d09c71b2%29&filter=sc_long_sign&tn=SE_xueshusource_2kduw22v&sc_vurl=http%3A%2F%2Fciteseerx.ist.psu.edu%2Fviewdoc%2Fdownload%3Fdoi%3D10.1.1.190.9540%26rep%3Drep1%26type%3Dpdf&ie=utf-8&sc_us=14537036281601669358) **Characteristics of properties and structure about *LcDHN-like* proteins in *Liriodendron*** | | | | | | | | | | |
| --- | --- | --- | --- | --- | --- | --- | --- | --- | --- | --- |
| **Taxa** | **Number of amino acids** | **Molecular weight** | **PI** | **instability index** | **Aliphatic index** | **Gravy** | **Alpha helix** | **Extended strand** | **Beta turn** | **Random coil** |
| **p1** | 158 | 16281.75 | 9.08 | 25.49 | 33.86 | -1.351 | 0.2215 | 8.86% | 17.72% | 51.27% |
| **p2** | 158 | 16325.78 | 9.19 | 25.49 | 33.86 | -1.349 | 0.2215 | 8.86% | 17.72% | 51.27% |
| **p3** | 158 | 16295.77 | 9.08 | 27.56 | 33.86 | -1.351 | 0.2532 | 8.86% | 16.46% | 49.37% |
| **p4** | 158 | 16269.73 | 9.08 | 24.11 | 35.13 | -1.316 | 0.2215 | 8.86% | 17.09% | 51.90% |
| **p5** | 158 | 16353.81 | 8.85 | 26.09 | 33.86 | -1.371 | 0.2089 | 8.86% | 16.46% | 53.80% |
| **p6** | 158 | 16267.72 | 9.08 | 26.51 | 33.86 | -1.352 | 0.1962 | 8.86% | 17.09% | 54.43% |
| **p7** | 154 | 15944.41 | 9.25 | 26.91 | 34.74 | -1.373 | 0.2013 | 9.09% | 14.29% | 56.49% |
| **p8** | 158 | 16312.76 | 9.08 | 27.47 | 33.86 | -1.363 | 0.2215 | 8.86% | 18.35% | 50.63% |
| **p9** | 158 | 16281.75 | 9.08 | 24.65 | 34.49 | -1.338 | 0.1962 | 8.86% | 17.09% | 54.43% |
| **p10** | 158 | 16271.71 | 9.08 | 27.06 | 33.86 | -1.346 | 0.2215 | 9.49% | 18.35% | 50.00% |
| **p11** | 158 | 16298.73 | 9.08 | 26.69 | 33.86 | -1.364 | 0.1962 | 8.86% | 19.62% | 51.90% |
| **p12** | 158 | 16253.69 | 9.08 | 27.74 | 32.66 | -1.366 | 0.2215 | 4.43% | 17.72% | 55.70% |
| **p13** | 158 | 16247.77 | 9.23 | 25.43 | 36.33 | -1.347 | 0.2215 | 9.49% | 15.82% | 52.53% |
| **p14** | 158 | 16247.73 | 9.08 | 25.49 | 36.33 | -1.345 | 0.2215 | 8.86% | 15.82% | 53.16% |
| **p15** | 158 | 16353.81 | 8.85 | 24.23 | 33.86 | -1.371 | 0.2342 | 8.86% | 13.92% | 53.80% |
| **p16** | 158 | 16253.69 | 9.08 | 24.96 | 32.66 | -1.366 | 0.2215 | 7.59% | 17.72% | 52.53% |
| **p17** | 158 | 16230.65 | 9.08 | 26.9 | 32.66 | -1.368 | 0.2215 | 3.80% | 17.72% | 56.33% |
| **p18** | 162 | 16588.02 | 9.1 | 28.04 | 37.22 | -1.254 | 0.1728 | 14.81% | 14.81% | 53.09% |
| **p19** | 162 | 16561.99 | 9.1 | 27.03 | 37.84 | -1.233 | 0.179 | 16.05% | 16.67% | 49.38% |
| **p20** | 173 | 17870.39 | 8.93 | 21.07 | 34.34 | -1.345 | 0.1965 | 8.09% | 13.87% | 58.38% |
| **p21** | 174 | 17971.49 | 8.93 | 21.01 | 34.14 | -1.341 | 0.1954 | 8.05% | 16.09% | 56.32% |
| **p22** | 161 | 16504.89 | 8.87 | 27.8 | 36.27 | -1.283 | 0.2298 | 12.42% | 15.53% | 49.07% |
| **p23** | 161 | 16530.93 | 8.87 | 28.82 | 35.65 | -1.304 | 0.2236 | 11.18% | 13.66% | 52.80% |
| **p24** | 159 | 16444.94 | 9.12 | 29.85 | 38.55 | -1.257 | 0.1887 | 14.47% | 14.47% | 52.20% |
| **p25** | 174 | 17899.43 | 9.14 | 20.73 | 34.66 | -1.322 | 0.1494 | 11.49% | 15.52% | 58.05% |
| **p26** | 175 | 18000.53 | 9.14 | 20.66 | 34.46 | -1.319 | 0.1486 | 11.43% | 17.71% | 56.00% |
| **p27** | 173 | 17773.22 | 8.6 | 21.41 | 33.18 | -1.349 | 0.1618 | 8.09% | 14.45% | 61.27% |
| **p28** | 162 | 16562.03 | 9.25 | 25.32 | 37.84 | -1.235 | 0.179 | 16.05% | 16.67% | 49.38% |
| **p29** | 174 | 17901.44 | 8.93 | 22.21 | 34.66 | -1.306 | 0.2126 | 8.05% | 14.94% | 55.75% |
| **p30** | 174 | 17957.46 | 8.93 | 21.12 | 33.56 | -1.353 | 0.1954 | 8.05% | 16.67% | 55.75% |
| **p31** | 173 | 17869.36 | 8.93 | 21.19 | 33.76 | -1.373 | 0.1965 | 9.25% | 13.87% | 57.23% |
| **p32** | 173 | 17842.33 | 8.93 | 21.41 | 33.18 | -1.37 | 0.1965 | 8.09% | 14.45% | 57.80% |
| **p33** | 173 | 17854.39 | 8.93 | 21.41 | 35.43 | -1.34 | 0.1965 | 10.40% | 13.29% | 56.65% |
| **p34** | 174 | 17943.44 | 8.93 | 21.34 | 32.99 | -1.366 | 0.1954 | 8.05% | 16.67% | 55.75% |
| **p35** | 173 | 17856.32 | 8.6 | 22.28 | 33.18 | -1.386 | 0.2139 | 10.40% | 14.45% | 53.76% |
| **p36** | 173 | 17843.32 | 8.6 | 22.28 | 33.18 | -1.37 | 0.2139 | 9.25% | 15.03% | 54.34% |
| **p37** | 174 | 17944.42 | 8.6 | 22.21 | 32.99 | -1.366 | 0.2126 | 9.20% | 17.24% | 52.30% |
| **p38** | 173 | 17855.37 | 8.6 | 22.28 | 35.43 | -1.34 | 0.2139 | 11.56% | 15.61% | 51.45% |
| **p39** | 161 | 16490.73 | 7.87 | 39.6 | 33.85 | -1.309 | 0.1429 | 12.42% | 18.01% | 55.28% |
| **p40** | 173 | 17798.32 | 9.14 | 21.02 | 34.34 | -1.327 | 0.1965 | 8.67% | 13.29% | 58.38% |
| **p41** | 162 | 16732.18 | 8.97 | 25.35 | 39.07 | -1.253 | 0.2284 | 11.11% | 15.43% | 50.62% |
| **p42** | 159 | 16276.56 | 8.57 | 30.07 | 34.91 | -1.275 | 0.1698 | 11.95% | 15.72% | 55.35% |
| **p43** | 161 | 16461.86 | 8.85 | 27.1 | 35.65 | -1.284 | 0.1863 | 11.18% | 13.66% | 56.52% |
| **p44** | 162 | 16614.1 | 9.1 | 28.04 | 39.63 | -1.225 | 0.1728 | 16.05% | 14.20% | 52.47% |
| **p45** | 159 | 16502.98 | 8.89 | 30.39 | 38.55 | -1.277 | 0.195 | 14.47% | 13.84% | 52.20% |
| **p46** | 173 | 17812.35 | 9.14 | 19.81 | 34.86 | -1.325 | 0.1503 | 10.98% | 15.03% | 58.96% |
| **p47** | 162 | 16658.11 | 8.87 | 28.82 | 37.84 | -1.273 | 0.2222 | 11.11% | 11.73% | 54.94% |
| **p48** | 161 | 16461.82 | 8.53 | 28.82 | 35.65 | -1.281 | 0.1863 | 11.18% | 13.66% | 56.52% |
| **p49** | 159 | 16388.77 | 9.12 | 31.06 | 36.1 | -1.303 | 0.1761 | 13.84% | 17.61% | 50.94% |
| **p50** | 163 | 16763.23 | 8.97 | 26.54 | 39.39 | -1.212 | 0.2454 | 11.04% | 16.56% | 47.85% |
| **p51** | 174 | 17861.33 | 8.6 | 21.34 | 32.99 | -1.329 | 0.1609 | 8.05% | 15.52% | 60.34% |
| **p52** | 164 | 16732.15 | 9.1 | 28.57 | 36.77 | -1.246 | 0.1707 | 13.41% | 13.41% | 56.10% |
| **p53** | 173 | 17801.28 | 8.6 | 21.07 | 34.34 | -1.323 | 0.1561 | 8.09% | 12.72% | 63.58% |
| **p54** | 174 | 17914.44 | 8.93 | 22.21 | 34.66 | -1.322 | 0.2126 | 8.05% | 16.09% | 54.60% |
| **p55** | 158 | 16170.52 | 8.89 | 29.77 | 35.13 | -1.276 | 0.1456 | 13.29% | 16.46% | 55.70% |
| **p56** | 161 | 16530.97 | 9.08 | 28.11 | 35.65 | -1.307 | 0.2236 | 11.18% | 13.66% | 52.80% |
| **p57** | 171 | 17799.26 | 8.62 | 17.93 | 35.26 | -1.371 | 0.152 | 9.94% | 12.87% | 61.99% |
| **p58** | 159 | 16474.86 | 8.54 | 25.17 | 37.92 | -1.287 | 0.1761 | 11.95% | 12.58% | 57.86% |
|  |  |  |  |  |  |  |  |  |  |  |
| **P1-P17:** Proteins from *L. chinense*  **P18-P58:** Proteins from *L. tulipifera* | | | | | | | | | | |
