# Supplementary material for: Interspecies evolutionary divergence in Liriodendron, evidence from the nucleotide variations of LcDHN-like gene
Source: BMC Evol Biol. 2018 Dec 19;18:195. doi: 10.1186/s12862-018-1318-7 (PMC6300021; doi:10.1186/s12862-018-1318-7)
Supplement: Supplementary file 2 — 5’ RACE, 3’ RACE and cDNA sequences of LcDHN-like gene. (DOCX 15 kb) [file 12862_2018_1318_MOESM2_ESM.docx]

**Additional file 2: 5’ RACE, 3’ RACE and cDNA sequences of *LcDHN-like* gene**

5’ RACE sequence (468bp)

GAAAATCTGATTTCAAGTGAACTTCTCTATCTGATTTGAAGTAGTTGATTTTGAGCCGTTGGATCTAGTTGAAAATGTCAGAGACGCGTGATGAGTATGGCAACCAGGTTCGCCAAACCGACGAGTATGGGAACCCGATTCAGCATAGTGGCACCGGGACAAAGCCCGGTTCGGGCATACATGGTGGGGGCCATGGGATTGGCACAGGTGGTGGTGGTGGACAAGGCAAGCTCCACCGTCCAGGCTCCGGCTCTTCCTCTGACGAGGATGATGGACAAGGTGGGCGTAGGAAGAAGGGTTTGACGGAGAAGATCAAAGAGAAGCTGCCAGGTGGAAACAAGACGACAGGTGTCTGTCATCCAGGGACTCAGGGAGTACAGGGGGGTCGTGAGCATGAGAAGCCAGGTTGCGGTCAGGGTGGACAGGTGGGCCGCGAGCATGAGAAGACAGGTTTCGGTCATCCAGGGA

3’ RACE sequence (423bp)

CGTAGGAAGAAGGGTTTGACGGAGAAGATCAAAGAGAAGCTGCCAGGTGGAAACAAGACGACAGGTGTCTGTCATCCAGGGACTCAGGGAGTACAGGGGGGTCGTGAGCATGAGAAGCCAGGTTGCGGTCAGGGTGGACAGGTGGGCCGCGAGCATGAGAAGACAGGTTTCGGTCATCCAGGGACTCAGGGTGGACAGGTGGGCAGCGAGCAGGAGAAGAAGGGTATGATTGAGAAGATCAAGGAGAAGCTGCCAGGTCACAAGTAGGATGTGTGTCCACCATACACGTGGAGTCTACACCTACGTGTCTTATGTTACTTATAATACGCATGGAGTGTGTCCGGAGTCTGTAATAATCTGCGCGTATCTGTTATGTCTTATTATGTGGTAAAAAACTTCTTATTGTGTGGTAAAAAAAAAAAA

cDNA sequence (707bp)

GAAAATCTGATTTCAAGTGAACTTCTCTATCTGATTTGAAGTAGTTGATTTTGAGCCGTTGGATCTAGTTGAAAATGTCAGAGACGCGTGATGAGTATGGCAACCAGGTTCGCCAAACCGACGAGTATGGGAACCCGATTCAGCATAGTGGCACCGGGACAAAGCCCGGTTCGGGCATACATGGTGGGGGCCATGGGATTGGCACAGGTGGTGGTGGTGGACAAGGCAAGCTCCACCGTCCAGGCTCCGGCTCTTCCTCTGACGAGGATGATGGACAAGGTGGGCGTAGGAAGAAGGGTTTGACGGAGAAGATCAAAGAGAAGCTGCCAGGTGGAAACAAGACGACAGGTGTCTGTCATCCAGGGACTCAGGGAGTACAGGGGGGTCGTGAGCATGAGAAGCCAGGTTGCGGTCAGGGTGGACAGGTGGGCCGCGAGCATGAGAAGACAGGTTTCGGTCATCCAGGGACTCAGGGTGGACAGGTGGGCAGCGAGCAGGAGAAGAAGGGTATGATTGAGAAGATCAAGGAGAAGCTGCCAGGTCACAAGTAGGATGTGTGTCCACCATACACGTGGAGTCTACACCTACGTGTCTTATGTTACTTATAATACGCATGGAGTGTGTCCGGAGTCTGTAATAATCTGCGCGTATCTGTTATGTCTTATTATGTGGTAAAAAACTTCTTATTGTGTGGTAAAAAAAAAAAA
