# Supplementary material for: Interspecies evolutionary divergence in Liriodendron, evidence from the nucleotide variations of LcDHN-like gene
Source: BMC Evol Biol. 2018 Dec 19;18:195. doi: 10.1186/s12862-018-1318-7 (PMC6300021; doi:10.1186/s12862-018-1318-7)
Supplement: Supplementary file 3 — 311 LcDHN-like gDNA sequences in Liriodendron. (DOCX 152 kb) [file 12862_2018_1318_MOESM3_ESM.docx]

**Additional file 3**:311 *LcDHN-like* gDNA sequences in *Liriodendron*

>ZJAJ51

AGCCGTTGGATCTAGTTGAAAATGTCAGAGACGCGTGATGAGTATGGCAACCAGGTTCGC

CAAACCGACGAGTATGGGAACCCGATTCAGCATACTGGCACCGGGACAAAGCCCGGTTCG

GGCATACATGGTGGGGGCCATGGGATTGGCACAGGTGGTGGTGGTGGACAAGGCAAGCTC

CACCGTCCAGGCTCCGGCTCTTCCTCTGTAAGTATATTCGATGTTTCTCAAGGGTCATTT

CACTTGGTATATAGTGTTGGGATGACTAGATTAGTTGATTAACGGTTGTGATTGTAAATC

AGTGATATGGGCTTCTGTGTACCTTATTTCACAATGATAATCAATTGATACGCTGGGTCG

TGATGAGCGGACCGGATGAGCCGTCCATCTTACAGGCTTTTTAAAATAGAATTATAGGGT

GGGTGGTATGAGGTCCACCAAGCCTGTAACATGGAATTACAATCATAGCCGTTAATTGGT

TCATCTCATCCGCTCATCGTGATCCAGCATGTCAAACAGAGATGGGTTTCTGGAGAAATG

GATGTATGTAAGCCCACAAATCACAAAAAAAATCTGACATAATGATGGAGAGAATGCAAG

GAGTGTAAGTGAATCTACGCCGTTAAATGGATTTCATTGTACTTAACTTCGTTTTGTCAA

ATATTTTGTAGGACGAGGATGATGGACAAGGTGGGCGTAGGAAGAAGGGTTTGACGGAGA

AGATCAAAGAGAAGCTGCCAGGTGGAAACAAGACGACAGGTGTCTGTCATCCAGGGACTC

AGGGAGTACAGGGGGGTCGCGAGCATGAGAAGCCAGGTTGCGGGCAGGGTGGACAGGTGG

GCCGCGAGCATGAGAAGACAGGTTTCGGTCATCCAGGAACTCAGGGTGGACAGGTGGGCA

GCGAGCAGGAGAAGAAGGGTATGATTGAGAAGATCAAGGAGAAGCTGCCAGGTCACAAGT

AGGATGTGTGTCCACCATACACGTGGAGTCTACACCTACGTGTCTTATGTTACTTATAAT

ACGCATGGAGTGTGTCCGGAGTCTGTAATAATCTGCGCGTATCTGTTATGTC

>ZJAJ53

AGCCGTTGGATCTAGTTGAAAATGTCAGAGACGCGTGATGAGTATGGCAACCAGGTTCGC

CAAACCGACGAGTATGGGAACCCGATTCAGCATACTGGCACCGGGACAAAGCCCGGTTCG

GGCATACATGGTGGGGGCCATGGGATTGGCACAGGTGGTGGTGGTGGACAAGGCAAGCTC

CACCGTCCAGGCTCCGGCTCTTCCTCTGTAAGTATATTCGATGTTTCTCAAGGGCCATTT

CACTTGGTATATCGTGTTGGGATGACTAGATTAGTTGATTAACGGTTGTGATTGTAAATC

AGTGATATGGGCTTCTGTGTACCTTATTTCACAATGATAATCAATTGATACGCTGGGTCG

TGATGAGCGGACCGGATGAGCCGTCCATCTTACAGGCTTTTTAAAATAGAATTATAGGGT

GGGTGGTATGAGGTCCACCAAGCCTGTAACATGGAATTACAATCATAGCCGTTAATTGGT

TCATCTCATCCGCTCATCGTGATCCAGCATGTCAAACAGAGATGGGTTTCTGGAGAAATG

GATGTATGTAAGCCCACAAATCACAAAAAAAATCTGACATAATGATGGAGAGAATGCAAG

GAGTGTAAGTGAATCTACGCCGTTAAATGGATTTCATTGTACTTAACTTCGTTTTGTCAA

ATATTTTGTAGGACGAGGATGATGGACAAGGTGGGCGTAGGAAGAAGGGTTTGACGGAGA

AGATCAAAGAGAAGCTGCCAGGTGGAAACAAGACGACAGGTGTCTGTCATCCAGGGACTC

AGGGAGTACAGGGGGGTCGCGAGCATGAGAAGCCAGGTTGCGGGCAGGGTGGACAGGTGG

GCCGCGAGCATGAGAAGACAGGTTTCGGTCATCCAGGAACTCAGGGTGGACAGGTGGGCA

GCGAGCAGGAGAAGAAGGGTATGATTGAGAAGATCAAGGAGAAGCTGCCAGGTCACAAGT

AGGATGTGTGTCCACCATACACGTGGAGTCTACACCTACGTGTCTTATGTTACTTATAAT

ACGCATGGAGTGTGTCCGGAGTCTGTAATAATCTGCGCGTATCTGTTATGTC

>ZJAJ54

AGCCGTTGGATCTAGTTGAAAATGTCAGAGACGCGTGATGAGTATGGCAACCAGGTTCGC

CAAACCGACGAGTATGGGAACCCGATTCAGCATACTGGCACCGGGACAAAGCCCGGTTCG

GGCATACATGGTGGGGGCCATGGGATTGGCACAGGTGGTGGTGGTGGACAAGGCAAGCTC

CACCGTCCAGGCTCCGGCTCTTCCTCTGTAAGTATATTCGATGTTTCTCAAGGGCCATTT

CACTTGGTATATCGTGTTGGGATGACTAGATTAGTTGATTAACGGTTGTGATTGTAAATC

AGTGATATGGGCTTCTGTGTACCTTATTTCATAATGATAATCAATTGATACGCTGGATCG

TGATGAGCGGACCGGATGAGCCGTCCATCTTACAGGCTTTTTAAAATAGAATTATAGGGT

GGGTGGTATGAGGTCTACCAAGCCTGTAACATGGAATTACAATCATAGCTGTTAATTGGT

TCATCTCATCCGCTCATCGTGATCCAGCATGTCAAACAGAGATGGGTTTCTGGAGAAATG

GATGTATGTAAGCCAACAAATCACAAAAAAAATCTGACATAATGATGGAGAGAATGCAAG

GAGTGTAAGTGAATCTACGCCGTTAAATGGATTTCATTGTACTTAATTTCGTTTCCTCAA

ATATTTTGTAGGACGAGGATGATGGACAAGGTGGGCGTAGGAAGAAGGGTTTGACGGAGA

AGATCAAAGAGAAGCTGCCAGGTGGAAACAAGACGACAGGTGTCTGTCATCCAGGGACTC

AGGGAGTACAGGGGGGTCGCGAGCATGAGAAGCCAGGTTGCGGGCAGGGTGGACAGGTGG

GCCGCGAGCATGAGAAGACAGGTTTCGGTCATCCAGGAACTCAGGGTGGACAGGTGGGCA

GCGAGCAGGAGAAGAAGGGTATGATTGAGAAGATCAAGGAGAAGCTGCCAGGTCACAAGT

AGGATGTGTGTCCACCATACACGTGGAGCCTACATCTACGTGTCTTATGTTACTTATAAT

ACGCATGGAGTGTGTCCGGAGTCTGTAATAATCTGCGCGTATCTGTTATGTC

>ZJAJ251

AGCCGTTGGATCTAGTTGAAAATGTCAGAGACGCGTGATGAGTATGGCAACCAGGTTCGC

CAAACCGACGAGTATGGGAACCCGATTCAGCATACTGGCACCGGGACAAAGCCCGGTTCG

GGCATACATGGTGGGGGCCATGGGATTGGCACAGGTGGTGGTGGTGGACAAGGCAAGCTC

CACCGTCCAGGCTCCGGCTCTTCCTCTGTAAGTATATTCGATGTTTCTCAAGGGCCATTT

CACTTGGTATATCGTGTTGGGATGACTAGATTAGTTGATTAACGGTTGTGATTGTAAATC

AGTGATATGGGCTTCTGTGTACCTTATTTCATAATGATAATCAATTGATACGCTGGATCG

TGATGAGCGGACCGGATGAGCCGTCCATCTTACAGGCTTTTTAAAATAGAATTATAGGGT

GGGTGGTATGAGGTCTACCAAGCCTGTAACATGGAATTACAATCATAGCTGTTAATTGGT

TCATCTCATCCGCTCATCGTGATCCAGCATGTCAAACAGAGATGGGTTTCTGGAGAAATG

GATGTATGTAAGCCAACAAATCACAAAAAAAATCTGACATAATGATGGAGAGAATGCAAG

GAGTGTAAGTGAATCTACGCCGTTAAATGGATTTCATTGTACTTAATTTCGTTTCCTCAA

ATATTTTGTAGGACGAGGATGATGGACAAGGTGGGCGTAGGAAGAAGGGTTTGACGGAGA

AGATCAAAGAGAAGCTGCCAGGTGGAAACAAGACGACAGGTGTCTGTCATCCAGGGACTC

AGGGAGTACAGGGGGGTCGCGAGCATGAGAAGCCAGGTTGCGGGCAGGGTGGACAGGTGG

GCCGCGAGCATGAGAAGACAGGTTTCGGTCATCCAGGAACTCAGGGTGGACAGGTGGGCA

GCGAGCAGGAGAAGAAGGGTATGATTGAGAAGATCAAGGAGAAGCTGCCAGGTCACAAGT

AGGATGTGTGTCCACCATACACGTGGAGCCTACATCTACGTGTCTTATGTTACTTATAAT

ACGCATGGAGTGTGTCCGGAGTCTGTAATAATCTGCGCGTATCTGTTATGTC

>ZJAJ253

AGCCGTTGGATCTAGTTGAAAATGTCAGAGACCCGTGAGGAGTATGGCAACCAGGTTCGC

CAAACCGACGAGTATGGGAACCCGATTCAGCATACTGGCACCGGGACAAAGCCCGGTTCG

GGCATACATGGTGGGGGCCATGGGATTGGCACAGGTGGTGGTGGTGGACAAGGCAAGCTC

CACCGTCCAGGCTCCGGCTCTTCCTCTGTAAGTATATTCGATGTTTCTCAAGGGCCATTT

CACTTGGTATGTCGTGTTGGGATGACTAGATTAGTTGATTAACGGTTGTGATTGTAAATC

AGTGATATGGGCTTCTGTGTACCTTATTTCACAATGATAATCAATTGATACGCTGGATCG

TGATGAGCGGACCGGATGAGCCGTCCATCTTACAGGCTTTTTAAAATAGAATTATAGGGT

GGGTGGTATGAGGTCTACCAAGCCTGTAACATGGAATTACAATCATAGCTGTTAATCGGT

TCATCTCATCCGCTCATCGTGATCCAGCATGTCAAACAGAGATGGGTTTATGGAGAAATG

GATGTATGTAAGCCAACAAATCACAAAAAAATCTGACATAATGATGGAGAGAATGCAAGG

AGTGTAAGTGAATCTACGCCGTTAAATGGATTTCATTGTACTTAATTTCGTTTTGTCAAA

TATTTTGTAGGACGAGGATGATGGACAAGGTGGGCGTAGGAAGAAGGGTTTGACGGAGAA

GATCAAAGAGAAGCTGCCAGGTGGAAACAAGACGACAGGTGTCTGTCATCCAGGGACTCA

GGGAGTACAGGGGGGTCGCGAGCATGAGAAGCCAGGTTGCGGTCAGGGTGGACAGGTGGG

CCGCGAGCATGAGAAGACAGGTTTCGGTCATCCAGGGACTCAGGGTGGACAGGTGGGCAG

CGAGCAGGAGAAGAAGGGTATGATTGAGAAGATCAAGGAGAAGCTGCCAGGTCACAAGTA

GGATGTGTGTCCACCATACACGTGGAGTCTACACCTACGTGTCTTATGTTACTTATAATA

CGCATGGAGTGTGTCCGGAGTCTGTAATAATCTGCGCGTATCTGTTATGTC

>ZJAJ255

AGCCGTTGGATCTAGTTGAAAATGTCAGAGACGCGTGATGAGTATGGCAACCAGGTTCGC

CAAACCGACGAGTATGGGAACCCGATTCAGCATACTGGCACCGGGACAAAGCCCGGTTCG

GGCATACATGGTGGGGGCCATGGGATTGGCACAGGTGGTGGTGGTGGACAAGGCAAGCTC

CACCGTCCAGGCTCCGGCTCTTCCTCTGTAAGTATATTCGATGTTTCTCAAGGGCCATTT

CACTTGGTATATCGTGTTGGGATGACTAGATTAGTTGATTAACGGTTGTGATTGTAAATC

AGTGATATGGGCTTCTGTGTACCTTATTTCATAATGATAATCAATTGATACGCTGGATCG

TGATGAGCGGACCGGATGAGCCGTCCATCTTACAGGCTTTTTAAAATAGAATTATAGGGT

GGGTGGTATGAGGTCTACCAAGCCTGTAACATGGAATTACAATCATAGCTGTTAATTGGT

TCATCTCATCCGCTCATCGTGATCCAGCATGTCAAACAGAGATGGGTTTCTGGAGAAATG

GATGTATGTAAGCCAACAAATCACAAAAAAAATCTGACATAATGATGGAGAGAATGCAAG

GAGTGTAAGTGAATCTACGCCGTTAAATGGATTTCATTGTACTTAATTTCGTTTCCTCAA

ATATTTTGTAGGACGAGGATGATGGACAAGGTGGGCGTAGGAAGAAGGGTTTGACGGAGA

AGATCAAAGAGAAGCTGCCAGGTGGAAACAAGACGACAGGTGTCTGTCATCCAGGGACTC

AGGGAGTACAGGGGGGTCGCGAGCATGAGAAGCCAGGTTGCGGGCAGGGTGGACAGGTGG

GCCGCGAGCATGAGAAGACAGGTTTCGGTCATCCAGGAACTCAGGGTGGACAGGTGGGCA

GCGAGCAGGAGAAGAAGGGTATGATTGAGAAGATCAAGGAGAAGCTGCCAGGTCACAAGT

AGGATGTGTGTCCACCATACACGTGGAGCCTACATCTACGTGTCTTATGTTACTTATAAT

ACGCATGGAGTGTGTCCGGAGTCTGTAATAATCTGCGCGTATCTGTTATGTC

>ZJAJ341

AGCCGTTGGATCTAGTTGAAAATGTCAGAGACGCGTGATGAGTATGGCAACCAGGTTCGC

CAAACCGACGAGTATGGGAACCCGATTCAGCATACTGGCACCGGGACAAAGCCCGGTTCG

GGCATACATGGTGGGGGCCATGGGATTGGCACAGGTGGTGGTGGTGGACAAGGCAAGCTC

CACCGTCCAGGCTCCGGCTCTTCCTCTGTAAGTATATTCGATGTTTCTCAAGGGTCATTT

CACTTGGTATATAGTGTTGGGATGACTAGATTAGTTGATTAACGGTTGTGATTGTAAATC

AGTGATATGGGCTTCTGTGTACCTTATTTCACAATGATAATCAATTGATACGCTGGGTCG

TGATGAGCGGACCGGATGAGCCGTCCATCTTACAGGCTTTTTAAAATAGAATTATAGGGT

GGGTGGTATGAGGTCCACCAAGCCTGTAACATGGAATTACAATCATAGCCGTTAATTGGT

TCATCTCATCCGCTCATCGTGATCCAGCATGTCAAACAGAGATGGGTTTCTGGAGAAATG

GATGTATGTAAGCCCACAAATCACAAAAAAAATCTGACATAATGATGGAGAGAATGCAAG

GAGTGTAAGTGAATCTACGCCGTTAAATGGATTTCATTGTACTTAACTTCGTTTTGTCAA

ATATTTTGTAGGACGAGGATGATGGACAAGGTGGGCGTAGGAAGAAGGGTTTGACGGAGA

AGATCAAAGAGAAGCTGCCAGGTGGAAACAAGACGACAGGTGTCTGTCATCCAGGGACTC

AGGGAGTACAGGGGGGTCGCGAGCATGAGAAGCCAGGTTGCGGGCAGGGTGGACAGGTGG

GCCGCGAGCATGAGAAGACAGGTTTCGGTCATCCAGGAACTCAGGGTGGACAGGTGGGCA

GCGAGCAGGAGAAGAAGGGTATGATTGAGAAGATCAAGGAGAAGCTGCCAGGTCACAAGT

AGGATGTGTGTCCACCATACACGTGGAGTCTACACCTACGTGTCTTATGTTACTTATAAT

ACGCATGGAGTGTGTCCGGAGTCTGTAATAATCTGCGCGTATCTGTTATGTC

>ZJAJ342

AGCCGTTGGATCTAGTTGAAAATGTCAGAGACGCGTGATGAGTATGGCAACCAGGTTCGC

CAAACCGACGAGTATGGGAACCCGATTCAGCATACTGGCACCGGGACAAAGCCCGGTTCG

GGCATACATGGTGGGGGCCATGGGATTGGCACAGGTGGTGGTGGTGGACAAGGCAAGCTC

CACCGTCCAGGCTCCGGCTCTTCCTCTGTAAGTATATTCGATGTTTCTCAAGGGTCATTT

CACTTGGTATATAGTGTTGGGATGACTAGATTAGTTGATTAACGGTTGTGGTTGTAAATC

AGTGATATGGGCTTCTATGTACCTTATTTCACAATGATAATCAATTGATACGCTGGATCG

TGATGAGCGGACCGGATGAGCCGTCCATCTTACAGGCTTTTTAAAATAGAATTATAGGGT

GGGTGGTATGAGGTCCACCAAGCCTGTAACATGGAATTACAATCATAGCCGTTAATTGGT

TCATCTCATCCGCTCATCGTGATCCAGCATGTCAAACAGAGATGGGTTTCTGGAGAAGTG

GATGTATGTAAGCCCACAAATCACAAAAAAAATCTGACATAATGATGGAGAGAATGCAAG

GAGTGTAAGTGAATCTACGCCGTTAAATGGATTTCATTGTACTTAACTTCGTTTTGTCAA

ATATTTTGTAGGACGAGGATGATGGACAAGGTGGGCGTAGGAAGAAGGGTTTGACGGAGA

AGATCAAAGAGAAGCTGCCAGGTGGAAACAAGACGACAGGTGTCTGTCATCCAGGGACTC

AGGGAGTACAGGGGGGTCGCGAGCATGAGAAGCCAGGTTTCGGTCAGGGTGGACAGGTGG

GCCGCGAGCATGAGAAGACAGGTTTCGGTCATCCAGGGACTCAGGGTGGACAGGTGGGCA

GCGAGCAGGAGAAGAAGGGTATGATTGAGAAGATCAAGGAGAAGCTGCCAGGTCACAAGT

AGGATGTGTGTCCACTATACACGTGGAGTCTGCATCTACGTGTCTTATGTTACTTATAAT

ACGCATGGAGTGTGTCCGGAGTCTGTAATAATCTGCGCGTATCTGTTATGTC

>ZJAJ343

AGCCGTTGGATCTAGTTGAAAATGTCAGAGACGCGTGATGAGTATGGCAACCAGGTTCGC

CAAACCGACGAGTATGGGAACCCGATTCAGCATACTGGCACCGGGACAAAGCCCGGTTCG

GGCATACATGGTGGGGGCCATGGGATTGGCACAGGTGGTGGTGGTGGACAAGGCAAGCTC

CACCGTCCAGGCTCCGGCTCTTCCTCTGTAAGTATATTCGATGTTTCTCAAGGGTCATTT

CACTTGGTATATAGTGTTGGGATGACTAGATTAGTTGATTAACGGTTGTGGTTGTAAATC

AGTGATATGGGCTTCTATGTACCTTATTTCACAATGATAATCAATTGATACGCTGGATCG

TGATGAGCGGACCGGATGAGCCGTCCATCTTACAGGCTTTTTAAAATAGAATTATAGGGT

GGGTGGTATGAGGTCCACCAAGCCTGTAACATGGAATTACAATCATAGCCGTTAATTGGT

TCATCTCATCCGCTCATCGTGATCCAGCATGTCAAACAGAGATGGGTTTCTGGAGAAGTG

GATGTATGTAAGCCCACAAATCACAAAAAAAATCTGACATAATGATGGAGAGAATGCAAG

GAGTGTAAGTGAATCTACGCCGTTAAATGGATTTCATTGTACTTAACTTCGTTTTGTCAA

ATATTTTGTAGGACGAGGATGATGGACAAGGTGGGCGTAGGAAGAAGGGTTTGACGGAGA

AGATCAAAGAGAAGCTGCCAGGTGGAAACAAGACGACAGGTGTCTGTCATCCAGGGACTC

AGGGAGTACAGGGGGGTCGCGAGCATGAGAAGCCAGGTTTCGGTCAGGGTGGACAGGTGG

GCCGCGAGCATGAGAAGACAGGTTTCGGTCATCCAGGGACTCAGGGTGGACAGGTGGGCA

GCGAGCAGGAGAAGAAGGGTATGATTGAGAAGATCAAGGAGAAGCTGCCAGGTCACAAGT

AGGATGTGTGTCCACTATACACGTGGAGTCTGCATCTACGTGTCTTATGTTACTTATAAT

ACGCATGGAGTGTGTCCGGAGTCTGTAATAATCTGCGCGTATCTGTTATGTC

>ZJAJ349

AGCCGTTGGATCTAGTTGAAAATGTCAGAGACGCGTGATGAGTATGGCAACCAGGTTCGC

CAAACCGACGAGTATGGGAACCCGATTCAGCATACTGGCACCGGGACAAAGCCCGGTTCG

GGCATACATGGTGGGGGCCATGGGATTGGCACAGGTGGTGGTGGTGGACAAGGCAAGCTC

CACCGTCCAGGCTCCGGCTCTTCCTCTGTAAGTATATTCGATGTTTCTCAAGGGTCATTT

CACTTGGTATATAGTGTTGGGATGACTAGATTAGTTGATTAACGGTTGTGGTTGTAAATC

AGTGATATGGGCTTCTATGTACCTTATTTCACAATGATAATCAATTGATACGCTGGATCG

TGATGAGCGGACCGGATGAGCCGTCCATCTTACAGGCTTTTTAAAATAGAATTATAGGGT

GGGTGGTATGAGGTCCACCAAGCCTGTAACATGGAATTACAATCATAGCCGTTAATTGGT

TCATCTCATCCGCTCATCGTGATCCAGCATGTCAAACAGAGATGGGTTTCTGGAGAAGTG

GATGTATGTAAGCCCACAAATCACAAAAAAAATCTGACATAATGATGGAGAGAATGCAAG

GAGTGTAAGTGAATCTACGCCGTTAAATGGATTTCATTGTACTTAACTTCGTTTTGTCAA

ATATTTTGTAGGACGAGGATGATGGACAAGGTGGGCGTAGGAAGAAGGGTTTGACGGAGA

AGATCAAAGAGAAGCTGCCAGGTGGAAACAAGACGACAGGTGTCTGTCATCCAGGGACTC

AGGGAGTACAGGGGGGTCGCGAGCATGAGAAGCCAGGTTTCGGTCAGGGTGGACAGGTGG

GCCGCGAGCATGAGAAGACAGGTTTCGGTCATCCAGGGACTCAGGGTGGACAGGTGGGCA

GCGAGCAGGAGAAGAAGGGTATGATTGAGAAGATCAAGGAGAAGCTGCCAGGTCACAAGT

AGGATGTGTGTCCACTATACACGTGGAGTCTGCATCTACGTGTCTTATGTTACTTATAAT

ACGCATGGAGTGTGTCCGGAGTCTGTAATAATCTGCGCGTATCTGTTATGTC

>ZJAJ3411

AGCCGTTGGATCTAGTTGAAAATGTCAGAGACGCGTGATGAGTATGGCAACCAGGTTCGC

CAAACCGACGAGTATGGGAACCCGATTCAGCATACTGGCACCGGGACAAAGCCCGGTTCG

GGCATACATGGTGGGGGCCATGGGATTGGCACAGGTGGTGGTGGTGGACAAGGCAAGCTC

CACCGTCCAGGCTCCGGCTCTTCCTCTGTAAGTATATTCGATGTTTCTCAAGGGTCATTT

CACTTGGTATATAGTGTTGGGATGACTAGATTAGTTGATTAACGGTTGTGATTGTAAATC

AGTGATATGGGCTTCTGTGTACCTTATTTCACAATGATAATCAATTGATACGCTGGGTCG

TGATGAGCGGACCGGATGAGCCGTCCATCTTACAGGCTTTTTAAAATAGAATTATAGGGT

GGGTGGTATGAGGTCCACCAAGCCTGTAACATGGAATTACAATCATAGCCGTTAATTGGT

TCATCTCATCCGCTCATCGTGATCCAGCATGTCAAACAGAGATGGGTTTCTGGAGAAGTG

GATGTATGTAAGCCCACAAATCACAAAAAAAATCTGACATAATGATGGAGAGAATGCAAG

GAGTGTAAGTGAATCTACGCCGTTAAATGGATTTCATTGTACTTAACTTCGTTTTGTCAA

ATATTTTGTAGGACGAGGATGATGGACAAGGTGGGCGTAGGAAGAAGGGTTTGACGGAGA

AGATCAAAGAGAAGCTGCCAGGTGGAAACAAGACGACAGGTGTCTGTCATCCAGGGACTC

AGGGAGTACAGGGGGGTCGCGAGCATGAGAAGCCAGGTTTCGGTCAGGGTGGACAGGTGG

GCCGCGAGCATGAGAAGACAGGTTTCGGTCATCCAGGGACTCAGGGTGGACAGGTGGGCA

GCGAGCAGGAGAAGAAGGGTATGATTGAGAAGATCAAGGAGAAGCTGCCAGGTCACAAGT

AGGATGTGTGTCCACTATACACGTGGAGTCTGCATCTACGTGTCTTATGTTACTTATAAT

ACGCATGGAGTGTGTCCGGAGTCTGTAATAATCTGCGCGTATCTGTTATGTC

>ZJAJ3412

AGCCGTTGGATCTAGTTGAAAATGTCAGAGACGCGTGATGAGTATGGCAACCAGGTTCGC

CAAACCGACGAGTATGGGAACCCGATTCAGCATACTGGCACCGGGACAAAGCCCGGTTCG

GGCATACATGGTGGGGGCCATGGGATTGGCACAGGTGGTGGTGGTGGACAAGGCAAGCTC

CACCGTCCAGGCTCCGGCTCTTCCTCTGTAAGTATATTCGATGTTTCTCAAGGGTCATTT

CACTTGGTATATAGTGTTGGGATGACTAGATTAGTTGATTAACGGTTGTGGTTGTAAATC

AGTGATATGGGCTTCTATGTACCTTATTTCACAATGATAATCAATTGATACGCTGGATCG

TGATGAGCGGACCGGATGAGCCGTCCATCTTACAGGCTTTTTAAAATAGAATTATAGGGT

GGGTGGTATGAGGTCCACCAAGCCTGTAACATGGAATTACAATCATAGCCGTTAATTGGT

TCATCTCATCCGCTCATCGTGATCCAGCATGTCAAACAGAGATGGGTTTCTGGAGAAGTG

GATGTATGTAAGCCCACAAATCACAAAAAAAATCTGACATAATGATGGAGAGAATGCAAG

GAGTGTAAGTGAATCTACGCCGTTAAATGGATTTCATTGTACTTAACTTCGTTTTGTCAA

ATATTTTGTAGGACGAGGATGATGGACAAGGTGGGCGTAGGAAGAAGGGTTTGACGGAGA

AGATCAAAGAGAAGCTGCCAGGTGGAAACAAGACGACAGGTGTCTGTCATCCAGGGACTC

AGGGAGTACAGGGGGGTCGCGAGCATGAGAAGCCAGGTTTCGGTCAGGGTGGACAGGTGG

GCCGCGAGCATGAGAAGACAGGTTTCGGTCATCCAGGGACTCAGGGTGGACAGGTGGGCA

GCGAGCAGGAGAAGAAGGGTATGATTGAGAAGATCAAGGAGAAGCTGCCAGGTCACAAGT

AGGATGTGTGTCCACTATACACGTGGAGTCTGCATCTACGTGTCTTATGTTACTTATAAT

ACGCATGGAGTGTGTCCGGAGTCTGTAATAATCTGCGCGTATCTGTTATGTC

>ZJAJ561

AGCCGTTGGATCTAGTTGAAAATGTCAGAGACGCGTGATGAGTATGGCAACCAGGTTCGC

CAAACCGACGAGTATGGGAACCCGATTCAGCATACTGGCACCGGGACAAAGCCCGGTTCG

GGCATACATGGTGGGGGCCATGGGATTGGCACAGGTGGTGGTGGTGGACAAGGCAAGCTC

CACCGTCCAGGCTCCGGCTCTTCCTCTGTAAGTATATTCGATGTTTCTCAAGGGTCATTT

CACTTGGTATATAGTGTTGGGATGACTAGATTAGTTGATTAACGGTTGTGGTTGTAAATC

AGTGATATGGGCTTCTATGTACCTTATTTCACAATGATAATCAATTGATACGCTGGATCG

TGATGAGCGGACCGGATGAGCCGTCCATCTTACAGGCTTTTTAAAATAGAATTATAGGGT

GGGTGGTATGAGGTCCACCAAGCCTGTAACATGGAATTACAATCATAGCCGTTAATTGGT

TCATCTCATCCGCTCATCGTGATCCAGCATGTCAAACAGAGATGGGTTTCTGGAGAAGTG

GATGTATGTAAGCCCACAAATCACAAAAAAAATCTGACATAATGATGGAGAGAATGCAAG

GAGTGTAAGTGAATCTACGCCGTTAAATGGATTTCATTGTACTTAACTTCGTTTTGTCAA

ATATTTTGTAGGACGAGGATGATGGACAAGGTGGGCGTAGGAAGAAGGGTTTGACGGAGA

AGATCAAAGAGAAGCTGCCAGGTGGAAACAAGACGACAGGTGTCTGTCATCCAGGGACTC

AGGGAGTACAGGGGGGTCGCGAGCATGAGAAGCCAGGTTTCGGTCAGGGTGGACAGGTGG

GCCGCGAGCATGAGAAGACAGGTTTCGGTCATCCAGGGACTCAGGGTGGACAGGTGGGCA

GCGAGCAGGAGAAGAAGGGTATGATTGAGAAGATCAAGGAGAAGCTGCCAGGTCACAAGT

AGGATGTGTGTCCACTATACACGTGGAGTCTGCATCTACGTGTCTTATGTTACTTATAAT

ACGCATGGAGTGTGTCCGGAGTCTGTAATAATCTGCGCGTATCTGTTATGTC

>ZJAJ562

AGCCGTTGGATCTAGTTGAAAATGTCAGAGACGCGTGATGAGTATGGCAACCAGGTTCGC

CAAACCGACGAGTATGGGAACCCGATTCAGCATACTGGCACCGGGACAAAGCCCGGTTCG

GGCATACATGGTGGGGGCCATGGGATTGGCACAGGTGGTGGTGGTGGACAAGGCAAGCTC

CACCGTCCAGGCTCCGGCTCTTCCTCTGTAAGTATATTCGATGTTTCTCAAGGGTCATTT

CACTTGGTATATAGTGTTGGGATGACTAGATTAGTTGATTAACGGTTGTGATTGTAAATC

AGTGATATGGGCTTCTGTGTACCTTATTTCACAATGATAATCAATTGATACGCTGGGTCG

TGATGAGCGGACCGGATGAGCCGTCCATCTTACAGGCTTTTTAAAATAGAATTATAGGGT

GGGTGGTATGAGGTCCACCAAGCCTGTAACATGGAATTACAATCATAGCCGTTAATTGGT

TCATCTCATCCGCTCATCGTGATCCAGCATGTCAAACAGAGATGGGTTTCTGGAGAAATG

GATGTATGTAAGCCCACAAATCACAAAAAAAATCTGACATAATGATGGAGAGAATGCAAG

GAGTGTAAGTGAATCTACGCCGTTAAATGGATTTCATTGTACTTAACTTCGTTTTGTCAA

ATATTTTGTAGGACGAGGATGATGGACAAGGTGGGCGTAGGAAGAAGGGTTTGACGGAGA

AGATCAAAGAGAAGCTGCCAGGTGGAAACAAGACGACAGGTGTCTGTCATCCAGGGACTC

AGGGAGTACAGGGGGGTCGCGAGCATGAGAAGCCAGGTTGCGGGCAGGGTGGACAGGTGG

GCCGCGAGCATGAGAAGACAGGTTTCGGTCATCCAGGAACTCAGGGTGGACAGGTGGGCA

GCGAGCAGGAGAAGAAGGGTATGATTGAGAAGATCAAGGAGAAGCTGCCAGGTCACAAGT

AGGATGTGTGTCCACCATACACGTGGAGTCTACACCTACGTGTCTTATGTTACTTATAAT

ACGCATGGAGTGTGTCCGGAGTCTGTAATAATCTGCGCGTATCTGTTATGTC

>ZJAJ564

AGCCGTTGGATCTAGTTGAAAATGTCAGAGACGCGTGATGAGTATGGCAACCAGGTTCGC

CAAACCGACGAGTATGGGAACCCGATTCAGCATACTGGCACCGGGACAAAGCCCGGTTCG

GGCATACATGGTGGGGGCCATGGGATTGGCACAGGTGGTGGTGGTGGACAAGGCAAGCTC

CACCGTCCAGGCTCCGGCTCTTCCTCTGTAAGTATATTCGATGTTTCTCAAGGGTCATTT

CACTTGGTATATAGTGTTGGGATGACTAGATTAGTTGATTAACGGTTGTGGTTGTAAATC

AGTGATATGGGCTTCTATGTACCTTATTTCACAATGATAATCAATTGATACGCTGGATCG

TGATGAGCGGACCGGATGAGCCGTCCATCTTACAGGCTTTTTAAAATAGAATTATAGGGT

GGGTGGTATGAGGTCCACCAAGCCTGTAACATGGAATTACAATCATAGCCGTTAATTGGT

TCATCTCATCCGCTCATCGTGATCCAGCATGTCAAACAGAGATGGGTTTCTGGAGAAGTG

GATGTATGTAAGCCCACAAATCACAAAAAAAATCTGACATAATGATGGAGAGAATGCAAG

GAGTGTAAGTGAATCTACGCCGTTAAATGGATTTCATTGTACTTAACTTCGTTTTGTCAA

ATATTTTGTAGGACGAGGATGATGGACAAGGTGGGCGTAGGAAGAAGGGTTTGACGGAGA

AGATCAAAGAGAAGCTGCCAGGTGGAAACAAGACGACAGGTGTCTGTCATCCAGGGACTC

AGGGAGTACAGGGGGGTCGCGAGCATGAGAAGCCAGGTTTCGGTCAGGGTGGACAGGTGG

GCCGCGAGCATGAGAAGACAGGTTTCGGTCATCCAGGGACTCAGGGTGGACAGGTGGGCA

GCGAGCAGGAGAAGAAGGGTATGATTGAGAAGATCAAGGAGAAGCTGCCAGGTCACAAGT

AGGATGTGTGTCCACTATACACGTGGAGTCTGCATCTACGTGTCTTATGTTACTTATAAT

ACGCATGGAGTGTGTCCGGAGTCTGTAATAATCTGCGCGTATCTGTTATGTC

>ZJSY42

AGCCGTTGGATCTAGTTGAAAATGTCAGAGACGCGTGATGAGTATGGCAACCAGGTTCGC

CAAACCGACGAGTATGGGAACCCGATTCAGCATACTGGCACCGGGACAAAGCCCGGTTCG

GGCATACATGGTGGGGGCCATGGGATTGGCACAGGTGGTGGTGGTGGACAAGGCAAGCTC

CACCGTCCAGGCTCCGGCTCTTCCTCTGTAAGTATATTCGATGTTTCTCAAGGGCCATTT

CACTTGGTATATCGTGTTGGGATGACTAGATTAGTTGATTAACGGTTGTGATTGTAAATC

AGTGATATGAGCTTCTGTGTACCTTATTTCACAATGATAATCAATTGATACGCTGGATCG

TGATGAGCGGACCGGATGAGCCGTCCATCTTACAGGCTTTTTAAAATAGAATTATAGGGT

GGGTGGTATGAGGTCTACCAAGCCTGTAACATGGAATTACAATCATAGCTGTTAATCGGT

TCATCTCATCCGCTCATCGTGATCCAGCATGTCAAACAGAGATGGGTTTATGGAGAAATG

GATGTATGTAAGCCAACAAATCACAAAAAAATCTGACATAATGATGGAGAGAATGCAAGG

AGTGTAAGTGAATCTACGCCGTTAAATGGATTTCATTGTACTTAATTTCGTTTTGTCAAA

TATTTTGTAGGACGAGGATGATGGACAAGGTGGGCGTAGGAAGAAGGGTTTGACGGAGAA

GATCAAAGAGAAGCTGCCAGGTGGAAACAAGACGACAGGTGTCTGTCATCCAGGGACTCA

GGGAGTACAGGGGGGTCGCGAGCATGAGAAGCCAGGTTGCGGTCAGGGTGGACAGGTGGG

CCGCGAGCATGAGAAGACAGGTTTCGGTCATCCAGGGACTCAGGGTGGACAGGTGGGCAG

CGAGCAGGAGAAGAAGGGTATGATTGAGAAGATCAAGGAGAAGCTGCCAGGTCACAAGTA

GGATGTGTGTCCACCATACACGTGGAGTCTACACCTACGTGTCTTATGTTACTTATAATA

CGCATGGAGTGTGTCCGGAGTCTGTAATAATCTGCGCGTATCTGTTATGTC

>ZJSY45

AGCCGTTGGATCTAGTTGAAAATGTCAGAGACGCGTGATGAGTATGGCAACCAGGTTCGC

CAAACCGACGAGTATGGGAACCCGATTCAGCATACTGGCACCGGGACAAAGCCCGGTTCG

GGCATACATGGTGGGGGCCATGGGATTGGCACAGGTGGTGGTGGTGGACAAGGCAAGCTC

CACCGTCCAGGCTCCGGCTCTTCCTCTGTAAGTATATTCGATGTTTCTCAAGGGCCATTT

CACTTGGTATATCGTGTTGGGATGACTAGATTAGTTGATTAACGGTTGTGATTGTAAATC

AGTGATATGAGCTTCTGTGTACCTTATTTCACAATGATAATCAATTGATACGCTGGATCG

TGATGAGCGGACCGGATGAGCCGTCCATCTTACAGGCTTTTTAAAATAGAATTATAGGGT

GGGTGGTATGAGGTCTACCAAGCCTGTAACATGGAATTACAATCATAGCTGTTAATCGGT

TCATCTCATCCGCTCATCGTGATCCAGCATGTCAAACAGAGATGGGTTTATGGAGAAATG

GATGTATGTAAGCCAACAAATCACAAAAAAATCTGACATAATGATGGAGAGAATGCAAGG

AGTGTAAGTGAATCTACGCCGTTAAATGGATTTCATTGTACTTAATTTCGTTTTGTCAAA

TATTTTGTAGGACGAGGATGATGGACAAGGTGGGCGTAGGAAGAAGGGTTTGACGGAGAA

GATCAAAGAGAAGCTGCCAGGTGGAAACAAGACGACAGGTGTCTGTCATCCAGGGACTCA

GGGAGTACAGGGGGGTCGCGAGCATGAGAAGCCAGGTTGCGGTCAGGGTGGACAGGTGGG

CCGCGAGCATGAGAAGACAGGTTTCGGTCATCCAGGGACTCAGGGTGGACAGGTGGGCAG

CGAGCAGGAGAAGAAGGGTATGATTGAGAAGATCAAGGAGAAGCTGCCAGGTCACAAGTA

GGATGTGTGTCCACCATACACGTGGAGTCTACACCTACGTGTCTTATGTTACTTATAATA

CGCATGGAGTGTGTCCGGAGTCTGTAATAATCTGCGCGTATCTGTTATGTC

>ZJSY49

AGCCGTTGGATCTAGTTGAAAATGTCAGAGACGCGTGATGAGTATGGCAACCAGGTTCGC

CAAACCGACGAGTATGGGAACCCGATTCAGCATACTGGCACCGGGACAAAGCCCGGTTCG

GGCATACATGGTGGGGGCCATGGGATTGGCACAGGTGGTGGTGGTGGACAAGGCAAGCTC

CACCGTCCAGGCTCCGGCTCTTCCTCTGTAAGTATATTCGATGTTTCTCAAGGGCCATTT

CACTTGGTATATCGTGTTGGGATGACTAGATTAGTTGATTAACGGTTGTGATTGTAAATC

AGTGATATGAGCTTCTGTGTACCTTATTTCACAATGATAATCAATTGATACGCTGGATCG

TGATGAGCGGACCGGATGAGCCGTCCATCTTACAGGCTTTTTAAAATAGAATTATAGGGT

GGGTGGTATGAGGTCTACCAAGCCTGTAACATGGAATTACAATCATAGCTGTTAATCGGT

TCATCTCATCCGCTCATCGTGATCCAGCATGTCAAACAGAGATGGGTTTATGGAGAAATG

GATGTATGTAAGCCAACAAATCACAAAAAAATCTGACATAATGATGGAGAGAATGCAAGG

AGTGTAAGTGAATCTACGCCGTTAAATGGATTTCATTGTACTTAATTTCGTTTTGTCAAA

TATTTTGTAGGACGAGGATGATGGACAAGGTGGGCGTAGGAAGAAGGGTTTGACGGAGAA

GATCAAAGAGAAGCTGCCAGGTGGAAACAAGACGACAGGTGTCTGTCATCCAGGGACTCA

GGGAGTACAGGGGGGTCGCGAGCATGAGAAGCCAGGTTGCGGTCAGGGTGGACAGGTGGG

CCGCGAGCATGAGAAGACAGGTTTCGGTCATCCAGGGACTCAGGGTGGACAGGTGGGCAG

CGAGCAGGAGAAGAAGGGTATGATTGAGAAGATCAAGGAGAAGCTGCCAGGTCACAAGTA

GGATGTGTGTCCACCATACACGTGGAGTCTACACCTACGTGTCTTATGTTACTTATAATA

CGCATGGAGTGTGTCCGGAGTCTGTAATAATCTGCGCGTATCTGTTATGTC

>ZJSY64

AGCCGTTGGATCTAGTTGAAAATGTCAGAGACGCGTGATGAGTATGGCAACCAGGTTCGC

CAAACCGACGAGTATGGGAACCCGATTCAGCATACTGGCACCGGGACAAAGCCCGGTTCG

GGCATACATGGTGGGGGCCATGGGATTGGCACAGGTGGTGGTGGTGGACAAGGCAAGCTC

CACCGTCCAGGCTCCGGCTCTTCCTCTGTAAGTATATTCGATGTTTCTCAAGGGCCATTT

CACTTGGTATATCGTGTTGGGATGACTAGATTAGTTGATTAACGGTTGTGATTGTAAATC

AGTGATATGAGCTTCTGTGTACCTTATTTCACAATGATAATCAATTGATACGCTGGATCG

TGATGAGCGGACCGGATGAGCCGTCCATCTTACAGGCTTTTTAAAATAGAATTATAGGGT

GGGTGGTATGAGGTCTACCAAGCCTGTAACATGGAATTACAATCATAGCTGTTAATCGGT

TCATCTCATCCGCTCATCGTGATCCAGCATGTCAAACAGAGATGGGTTTATGGAGAAATG

GATGTATGTAAGCCAACAAATCACAAAAAAATCTGACATAATGATGGAGAGAATGCAAGG

AGTGTAAGTGAATCTACGCCGTTAAATGGATTTCATTGTACTTAATTTCGTTTTGTCAAA

TATTTTGTAGGACGAGGATGATGGACAAGGTGGGCGTAGGAAGAAGGGTTTGACGGAGAA

GATCAAAGAGAAGCTGCCAGGTGGAAACAAGACGACAGGTGTCTGTCATCCAGGGACTCA

GGGAGTACAGGGGGGTCGCGAGCATGAGAAGCCAGGTTGCGGTCAGGGTGGACAGGTGGG

CCGCGAGCATGAGAAGACAGGTTTCGGTCATCCAGGGACTCAGGGTGGACAGGTGGGCAG

CGAGCAGGAGAAGAAGGGTATGATTGAGAAGATCAAGGAGAAGCTGCCAGGTCACAAGTA

GGATGTGTGTCCACCATACACGTGGAGTCTACACCTACGTGTCTTATGTTACTTATAATA

CGCATGGAGTGTGTCCGGAGTCTGTAATAATCTGCGCGTATCTGTTATGTC

>ZJSY66

AGCCGTTGGATCTAGTTGAAAATGTCAGAGACGCGTGATGAGTATGGCAACCAGGTTCGC

CAAACCGACGAGTATGGGAACCCGATTCAGCATAGTGGCACCGGGACAAAGCCCGGTTCG

GGCATACATGGTGGGGGCCATGGGATTGGCACAGGTGGTGGTGGTGGACAAGGCAAGCTC

CACCGTCCAGGCTCCGGCTCTTCCTCTGTAAGTATATTCGATGTTTCTCAAGGGTCATTT

CACTTGGTATATTGTGTTGGGATGACTCGATTAGTTGATTAACGGTTGTGATTGTAAATC

AGTGATATGGGCTTCTGTGTACCTTATTTCACAATGATAATCAATTGATACGCTGGATCG

TGATGAGCGGACCGGATGAGCCGTCCATCTTACAGGCTTTTTAAAATAGAATTATAGGGT

GGGTGGTATGAGGTCCACCAAAGCCTGTAACATGGAATTACAATCATAACCGTTAATCGG

TTCATCTCATCCGCTCATCGTGATCCAGCATGTCAAACAGAGATGGGTTTATGGAGAAAT

GGATGTATGTAAGCGCCAACAAATCACAAAAAAAATCTGACATAATGATGGAGAGAATGC

AAGGAGTGTAAGTGAATCTACGCCGTTAAATGGATTTCATTGTACTTAATTTCGTTTTGT

CAAATATTTTGTAGGACGAGGATGATGGACAAGGTGGGCGTAGGAAGAAGGGTTTGACGG

AGAAGATCAAAGAGAAGCTGCCAGGTGGAAACAAGACTACAGGTGTCTGTCATCCAGGGA

CTCAGGGAGTACAGGGGGGTCGCGAGCATGAGAAGCCAGGTTGCGGTCAGGGTGGACAGG

TGGGCCGCGAGCATGAGAAGACAGGTTTCGGTCATCCAGGGACTCAGGGTGGACAGGTGG

GCAGCGAGCAGGAGAAGAAGGGTATGATTGAGAAGATCAAGGAGAAGCTACCAGGTCACA

AGTAGGATGTGTGTCCACCATACACGTGGAGTCTGCATCTACGTGTCTTATGTTACTTAT

AATACGCATGGAGTGTGTCCGGAGTCTGTAATAATCTGCGCGTATCTGTTATGTC

>ZJSY613

AGCCGTTGGATCTAGTTGAAAATGTCAGAGACGCGTGATGAGTATGGCAACCAGGTTCGC

CAAACCGACGAGTATGGGAACCCGATTCAGCATACTGGCACCGGGACAAAGCCCGGTTCG

GGCATACATGGTGGGGGCCATGGGATTGGCACAGGTGGTGGTGGTGGACAAGGCAAGCTC

CACCGTCCAGGCTCCGGCTCTTCCTCTGTAAGTATATTCGATGTTTCTCAAGGGCCATTT

CACTTGGTATATCGTGTTGGGATGACTAGATTAGTTGATTAACGGTTGTGATTGTAAATC

AGTGATATGAGCTTCTGTGTACCTTATTTCACAATGATAATCAATTGATACGCTGGATCG

TGATGAGCGGACCGGATGAGCCGTCCATCTTACAGGCTTTTTAAAATAGAATTATAGGGT

GGGTGGTATGAGGTCTACCAAGCCTGTAACATGGAATTACAATCATAGCTGTTAATCGGT

TCATCTCATCCGCTCATCGTGATCCAGCATGTCAAACAGAGATGGGTTTATGGAGAAATG

GATGTATGTAAGCCAACAAATCACAAAAAAATCTGACATAATGATGGAGAGAATGCAAGG

AGTGTAAGTGAATCTACGCCGTTAAATGGATTTCATTGTACTTAATTTCGTTTTGTCAAA

TATTTTGTAGGACGAGGATGATGGACAAGGTGGGCGTAGGAAGAAGGGTTTGACGGAGAA

GATCAAAGAGAAGCTGCCAGGTGGAAACAAGACGACAGGTGTCTGTCATCCAGGGACTCA

GGGAGTACAGGGGGGTCGCGAGCATGAGAAGCCAGGTTGCGGTCAGGGTGGACAGGTGGG

CCGCGAGCATGAGAAGACAGGTTTCGGTCATCCAGGGACTCAGGGTGGACAGGTGGGCAG

CGAGCAGGAGAAGAAGGGTATGATTGAGAAGATCAAGGAGAAGCTGCCAGGTCACAAGTA

GGATGTGTGTCCACCATACACGTGGAGTCTACACCTACGTGTCTTATGTTACTTATAATA

CGCATGGAGTGTGTCCGGAGTCTGTAATAATCTGCGCGTATCTGTTATGTC

>ZJSY72

AGCCGTTGGATCTAGTTGAAAATGTCAGAGACGCGTGATGAGTATGGCAACCAGGTTCGC

CAAACCGACGAGTATGGGAACCCGATTCAGCATACTGGCACCGGGACAAAGCCCGGTTCG

GGCATACATGGTGGGGGCCATGGGATTGGCACAGGTGGTGGTGGTGGACAAGGCAAGCTC

CACCGTCCAGGCTCCGGCTCTTCCTCTGTAAGTATATTCGATGTTTCTCAAGGGCCATTT

CACTTGGTATATCGTGTTGGGATGACTAGATTAGTTGATTAACGGTTGTGATTGTAAATC

AGTGATATGAGCTTCTGTGTACCTTATTTCACAATGATAATCAATTGATACGCTGGATCG

TGATGAGCGGACCGGATGAGCCGTCCATCTTACAGGCTTTTTAAAATAGAATTATAGGGT

GGGTGGTATGAGGTCTACCAAGCCTGTAACATGGAATTACAATCATAGCTGTTAATCGGT

TCATCTCATCCGCTCATCGTGATCCAGCATGTCAAACAGAGATGGGTTTATGGAGAAATG

GATGTATGTAAGCCAACAAATCACAAAAAAATCTGACATAATGATGGAGAGAATGCAAGG

AGTGTAAGTGAATCTACGCCGTTAAATGGATTTCATTGTACTTAATTTCGTTTTGTCAAA

TATTTTGTAGGACGAGGATGATGGACAAGGTGGGCGTAGGAAGAAGGGTTTGACGGAGAA

GATCAAAGAGAAGCTGCCAGGTGGAAACAAGACGACAGGTGTCTGTCATCCAGGGACTCA

GGGAGTACAGGGGGGTCGCGAGCATGAGAAGCCAGGTTGCGGTCAGGGTGGACAGGTGGG

CCGCGAGCATGAGAAGACAGGTTTCGGTCATCCAGGGACTCAGGGTGGACAGGTGGGCAG

CGAGCAGGAGAAGAAGGGTATGATTGAGAAGATCAAGGAGAAGCTGCCAGGTCACAAGTA

GGATGTGTGTCCACCATACACGTGGAGTCTACACCTACGTGTCTTATGTTACTTATAATA

CGCATGGAGTGTGTCCGGAGTCTGTAATAATCTGCGCGTATCTGTTATGTC

>ZJSY73

AGCCGTTGGATCTAGTTGAAAATGTCAGAGACGCGTGATGAGTATGGCAACCAGGTTCGC

CAAACCGACGAGTATGGGAACCCGATTCAGCATACTGGCACCGGGACAAAGCCCGGTTCG

GGCATACATGGTGGGGGCCATGGGATTGGCACAGGTGGTGGTGGTGGACAAGGCAAGCTC

CACCGTCCAGGCTCCGGCTCTTCCTCTGTAAGTATATTCGATGTTTCTCAAGGGCCATTT

CACTTGGTATATCGTGTTGGGATGACTAGATTAGTTGATTAACGGTTGTGATTGTAAATC

AGTGATATGAGCTTCTGTGTACCTTATTTCACAATGATAATCAATTGATACGCTGGATCG

TGATGAGCGGACCGGATGAGCCGTCCATCTTACAGGCTTTTTAAAATAGAATTATAGGGT

GGGTGGTATGAGGTCTACCAAGCCTGTAACATGGAATTACAATCATAGCTGTTAATCGGT

TCATCTCATCCGCTCATCGTGATCCAGCATGTCAAACAGAGATGGGTTTATGGAGAAATG

GATGTATGTAAGCCAACAAATCACAAAAAAATCTGACATAATGATGGAGAGAATGCAAGG

AGTGTAAGTGAATCTACGCCGTTAAATGGATTTCATTGTACTTAATTTCGTTTTGTCAAA

TATTTTGTAGGACGAGGATGATGGACAAGGTGGGCGTAGGAAGAAGGGTTTGACGGAGAA

GATCAAAGAGAAGCTGCCAGGTGGAAACAAGACGACAGGTGTCTGTCATCCAGGGACTCA

GGGAGTACAGGGGGGTCGCGAGCATGAGAAGCCAGGTTGCGGTCAGGGTGGACAGGTGGG

CCGCGAGCATGAGAAGACAGGTTTCGGTCATCCAGGGACTCAGGGTGGACAGGTGGGCAG

CGAGCAGGAGAAGAAGGGTATGATTGAGAAGATCAAGGAGAAGCTGCCAGGTCACAAGTA

GGATGTGTGTCCACCATACACGTGGAGTCTACACCTACGTGTCTTATGTTACTTATAATA

CGCATGGAGTGTGTCCGGAGTCTGTAATAATCTGCGCGTATCTGTTATGTC

>ZJSY75

AGCCGTTGGATCTAGTTGAAAATGTCAGAGACGCGTGATGAGTATGGCAACCAGGTTCGC

CAAACCGACGAGTATGGGAACCCGATTCAGCATACTGGCACCGGGACAAAGCCCGGTTCG

GGCATACATGGTGGGGGCCATGGGATTGGCACAGGTGGTGGTGGTGGACAAGGCAAGCTC

CACCGTCCAGGCTCCGGCTCTTCCTCTGTAAGTATATTCGATGTTTCTCAAGGGCCATTT

CACTTGGTATATCGTGTTGGGATGACTAGATTAGTTGATTAACGGTTGTGATTGTAAATC

AGTGATATGAGCTTCTGTGTACCTTATTTCACAATGATAATCAATTGATACGCTGGATCG

TGATGAGCGGACCGGATGAGCCGTCCATCTTACAGGCTTTTTAAAATAGAATTATAGGGT

GGGTGGTATGAGGTCTACCAAGCCTGTAACATGGAATTACAATCATAGCTGTTAATCGGT

TCATCTCATCCGCTCATCGTGATCCAGCATGTCAAACAGAGATGGGTTTATGGAGAAATG

GATGTATGTAAGCCAACAAATCACAAAAAAATCTGACATAATGATGGAGAGAATGCAAGG

AGTGTAAGTGAATCTACGCCGTTAAATGGATTTCATTGTACTTAATTTCGTTTTGTCAAA

TATTTTGTAGGACGAGGATGATGGACAAGGTGGGCGTAGGAAGAAGGGTTTGACGGAGAA

GATCAAAGAGAAGCTGCCAGGTGGAAACAAGACGACAGGTGTCTGTCATCCAGGGACTCA

GGGAGTACAGGGGGGTCGCGAGCATGAGAAGCCAGGTTGCGGTCAGGGTGGACAGGTGGG

CCGCGAGCATGAGAAGACAGGTTTCGGTCATCCAGGGACTCAGGGTGGACAGGTGGGCAG

CGAGCAGGAGAAGAAGGGTATGATTGAGAAGATCAAGGAGAAGCTGCCAGGTCACAAGTA

GGATGTGTGTCCACCATACACGTGGAGTCTACACCTACGTGTCTTATGTTACTTATAATA

CGCATGGAGTGTGTCCGGAGTCTGTAATAATCTGCGCGTATCTGTTATGTC

>ZJSY914

AGCCGTTGGATCTAGTTGAAAATGTCAGAGACGCGTGATGAGTATGGCAACCAGGTTCGC

CAAACCGACGAGTATGGGAACCCGATTCAGCATACTGGCACCGGGACAAAGCCCGGTTCG

GGCATACATGGTGGGGGCCATGGGATTGGCACAGGTGGTGGTGGTGGACAAGGCAAGCTC

CACCGTCCAGGCTCCGGCTCTTCCTCTGTAAGTATATTCGATGTTTCTCAAGGGCCATTT

CACTTGGTATATCGTGTTGGGATGACTAGATTAGTTGATTAACGGTTGTGATTGTAAATC

AGTGATATGAGCTTCTGTGTACCTTATTTCACAATGATAATCAATTGATACGCTGGATCG

TGATGAGCGGACCGGATGAGCCGTCCATCTTACAGGCTTTTTAAAATAGAATTATAGGGT

GGGTGGTATGAGGTCTACCAAGCCTGTAACATGGAATTACAATCATAGCTGTTAATCGGT

TCATCTCATCCGCTCATCGTGATCCAGCATGTCAAACAGAGATGGGTTTATGGAGAAATG

GATGTATGTAAGCCAACAAATCACAAAAAAATCTGACATAATGATGGAGAGAATGCAAGG

AGTGTAAGTGAATCTACGCCGTTAAATGGATTTCATTGTACTTAATTTCGTTTTGTCAAA

TATTTTGTAGGACGAGGATGATGGACAAGGTGGGCGTAGGAAGAAGGGTTTGACGGAGAA

GATCAAAGAGAAGCTGCCAGGTGGAAACAAGACGACAGGTGTCTGTCATCCAGGGACTCA

GGGAGTACAGGGGGGTCGCGAGCATGAGAAGCCAGGTTGCGGTCAGGGTGGACAGGTGGG

CCGCGAGCATGAGAAGACAGGTTTCGGTCATCCAGGGACTCAGGGTGGACAGGTGGGCAG

CGAGCAGGAGAAGAAGGGTATGATTGAGAAGATCAAGGAGAAGCTGCCAGGTCACAAGTA

GGATGTGTGTCCACCATACACGTGGAGTCTACACCTACGTGTCTTATGTTACTTATAATA

CGCATGGAGTGTGTCCGGAGTCTGTAATAATCTGCGCGTATCTGTTATGTC

>ZJSY916

AGCCGTTGGATCTAGTTGAAAATGTCAGAGACGCGTGATGAGTATGGCAACCAGGTTCGC

CAAACCGACGAGTATGGGAACCCGATTCAGCATACTGGCACCGGGACAAAGCCCGGTTCG

GGCATACATGGTGGGGGCCATGGGATTGGCACAGGTGGTGGTGGTGGACAAGGCAAGCTC

CACCGTCCAGGCTCCGGCTCTTCCTCTGTAAGTATATTCGATGTTTCTCAAGGGCCATTT

CACTTGGTATATCGTGTTGGGATGACTAGATTAGTTGATTAACGGTTGTGATTGTAAATC

AGTGATATGAGCTTCTGTGTACCTTATTTCACAATGATAATCAATTGATACGCTGGATCG

TGATGAGCGGACCGGATGAGCCGTCCATCTTACAGGCTTTTTAAAATAGAATTATAGGGT

GGGTGGTATGAGGTCTACCAAGCCTGTAACATGGAATTACAATCATAGCTGTTAATCGGT

TCATCTCATCCGCTCATCGTGATCCAGCATGTCAAACAGAGATGGGTTTATGGAGAAATG

GATGTATGTAAGCCAACAAATCACAAAAAAATCTGACATAATGATGGAGAGAATGCAAGG

AGTGTAAGTGAATCTACGCCGTTAAATGGATTTCATTGTACTTAATTTCGTTTTGTCAAA

TATTTTGTAGGACGAGGATGATGGACAAGGTGGGCGTAGGAAGAAGGGTTTGACGGAGAA

GATCAAAGAGAAGCTGCCAGGTGGAAACAAGACGACAGGTGTCTGTCATCCAGGGACTCA

GGGAGTACAGGGGGGTCGCGAGCATGAGAAGCCAGGTTGCGGTCAGGGTGGACAGGTGGG

CCGCGAGCATGAGAAGACAGGTTTCGGTCATCCAGGGACTCAGGGTGGACAGGTGGGCAG

CGAGCAGGAGAAGAAGGGTATGATTGAGAAGATCAAGGAGAAGCTGCCAGGTCACAAGTA

GGATGTGTGTCCACCATACACGTGGAGTCTACACCTACGTGTCTTATGTTACTTATAATA

CGCATGGAGTGTGTCCGGAGTCTGTAATAATCTGCGCGTATCTGTTATGTC

>ZJSY917

AGCCGTTGGATCTAGTTGAAAATGTCAGAGACGCGTGATGAGTATGGCAACCAGGTTCGC

CAAACCGACGAGTATGGGAACCCGATTCAGCATACTGGCACCGGGACAAAGCCCGGTTCG

GGCATACATGGTGGGGGCCATGGGATTGGCACGGGTGGTGGTGGTGGACAAGGCAAGCTC

CACCGTCCAGGCTCCGGCTCTTCCTCTGTAAGTATATTCGATGTTTCTCAAGGGCCATTT

CACTTGGTATATCGTGTTGGGATGACTAGATTAGTTGATTAACGGTTGTGATTGTAAATC

AGTGATATGAGCTTCTGTGTACCTTATTTCACAATGATAATCAATTGATACGCTGGATCG

TGATGAGCGGACCGGATGAGCCGTCCATCTTACAGGCTTTTTAAAATAGAATTATAGGGT

GGGTGGTATGAGGTCTACCAAGCCTGTAACATGGAATTACAATCATAGCTGTTAATCGGT

TCATCTCATCCGCTCATCGTGATCCAGCATGTCAAACAGAGATGGGTTTATGGAGAAATG

GATGTATGTAAGCCAACAAATCACAAAAAAATCTGACATAATGATGGAGAGAATGCAAGG

AGTGTAAGTGAATCTACGCCGTTAAATGGATTTCATTGTACTTAATTTCGTTTTGTCAAA

TATTTTGTAGGACGAGGATGATGGACAAGGTGGGCGTAGGAAGAAGGGTTTGACGGAGAA

GATCAAAGAGAAGCTGCCAGGTGGAAACAAGACGACAGGTGTCTGTCATCCAGGGACTCA

GGGAGTACAGGGGGGTCGCGAGCATGAGAAGCCAGGTTGCGGTCAGGGTGGACAGGTGGG

CCGCGAGCATGAGAAGACAGGTTTCGGTCATCCAGGGACTCAGGGTGGACAGGTGGGCAG

CGAGCAGGAGAAGAAGGGTATGATTGAGAAGATCAAGGAGAAGCTGCCAGGTCACAAGTA

GGATGTGTGTCCACCATACACGTGGAGTCTACACCTACGTGTCTTATGTTACTTATAATA

CGCATGGAGTGTGTCCGGAGTCTGTAATAATCTGCGCGTATCTGTTATGTC

>AHJXQ41

AGCCGTTGGATCTAGTTGAAAATGTCAGAGACCCGTGAGGAGTATGGCAACCAGGTTCGC

CAAACCGACGAGTATGGGAACCCGATTCAGCATACTGGCACCGGGACAAAGCCCGGTTCG

GGCATACATGGTGGGGGCCATGGGATTGGCACAGGTGGTGGTGGTGGACAAGGCAAGCTC

CACCGTCCAGGCTCCGGCTCTTCCTCTGTAAGTATATTCGATGTTTCTCAAGGGCCATTT

CACTTGGTATGTCGTGTTGGGATGACTAGATTAGTTGATTAACGGTTGTGATTGTAAATC

AGTGATATGGGCTTCTGTGTACCTTATTTCACAATGATAATCAATTGATACGCTGGATCG

TGATGAGCGGACCGGATGAGCCGTCCATCTTACAGGCTTTTTAAAATAGAATTATAGGGT

GGGTGGTATGAGGTCTACCAAGCCTGTAACATGGAATTACAATCATAGCTGTTAATCGGT

TCATCTCATCCGCTCATCGTGATCCAGCATGTCAAACAGAGATGGGTTTATGGAGAAATG

GATGTATGTAAGCCAACAAATCACAAAAAAATCTGACATAATGATGGAGAGAATGCAAGG

AGTGTAAGTGAATCTACGCCGTTAAATGGATTTCATTGTACTTAATTTCGTTTTGTCAAA

TATTTTGTAGGACGAGGATGATGGACAAGGTGGGCGTAGGAAGAAGGGTTTGACGGAGAA

GATCAAAGAGAAGCTGCCAGGTGGAAACAAGACGACAGGTGTCTGTCATCCAGGGACTCA

GGGAGTACAGGGGGGTCGCGAGCATGAGAAGCCAGGTTGCGGTCAGGGTGGACAGGTGGG

CCGCGAGCATGAGAAGACAGGTTTCGGTCATCCAGGGACTCAGGGTGGACAGGTGGGCAG

CGAGCAGGAGAAGAAGGGTATGATTGAGAAGATCAAGGAGAAGCTGCCAGGTCACAAGTA

GGATGTGTGTCCACCATACACGTGGAGTCTACACCTACGTGTCTTATGTTACTTATAATA

CGCATGGAGTGTGTCCGGAGTCTGTAATAATCTGCGCGTATCTGTTATGTC

>AHJXQ43

AGCCGTTGGATCTAGTTGAAAATGTCAGAGACCCGTGAGGAGTATGGCAACCAGGTTCGC

CAAACCGACGAGTATGGGAACCCGATTCAGCATACTGGCACCGGGACAAAGCCCGGTTCG

GGCATACATGGTGGGGGCCATGGGATTGGCACAGGTGGTGGTGGTGGACAAGGCAAGCTC

CACCGTCCAGGCTCCGGCTCTTCCTCTGTAAGTATATTCGATGTTTCTCAAGGGCCATTT

CACTTGGTATGTCGTGTTGGGATGACTAGATTAGTTGATTAACGGTTGTGATTGTAAATC

AGTGATATGGGCTTCTGTGTACCTTATTTCACAATGATAATCAATTGATACGCTGGATCG

TGATGAGCGGACCGGATGAGCCGTCCATCTTACAGGCTTTTTAAAATAGAATTATAGGGT

GGGTGGTATGAGGTCTACCAAGCCTGTAACATGGAATTACAATCATAGCTGTTAATCGGT

TCATCTCATCCGCTCATCGTGATCCAGCATGTCAAACAGAGATGGGTTTATGGAGAAATG

GATGTATGTAAGCCAACAAATCACAAAAAAATCTGACATAATGATGGAGAGAATGCAAGG

AGTGTAAGTGAATCTACGCCGTTAAATGGATTTCATTGTACTTAATTTCGTTTTGTCAAA

TATTTTGTAGGACGAGGATGATGGACAAGGTGGGCGTAGGAAGAAGGGTTTGACGGAGAA

GATCAAAGAGAAGCTGCCAGGTGGAAACAAGACGACAGGTGTCTGTCATCCAGGGACTCA

GGGAGTACAGGGGGGTCGCGAGCATGAGAAGCCAGGTTGCGGTCAGGGTGGACAGGTGGG

CCGCGAGCATGAGAAGACAGGTTTCGGTCATCCAGGGACTCAGGGTGGACAGGTGGGCAG

CGAGCAGGAGAAGAAGGGTATGATTGAGAAGATCAAGGAGAAGCTGCCAGGTCACAAGTA

GGATGTGTGTCCACCATACACGTGGAGTCTACACCTACGTGTCTTATGTTACTTATAATA

CGCATGGAGTGTGTCCGGAGTCTGTAATAATCTGCGCGTATCTGTTATGTC

>AHJX1215

AGCCGTTGGATCTAGTTGAAAATGTCAGAGACCCGTGAGGAGTATGGCAACCAGGTTCGC

CAAACCGACGAGTATGGGAACCCGATTCAGCATACTGGCACCGGGACAAAGCCCGGTTCG

GGCATACATGGTGGGGGCCATGGGATTGGCACAGGTGGTGGTGGTGGACAAGGCAAGCTC

CACCGTCCAGGCTCCGGCTCTTCCTCTGTAAGTATATTCGATGTTTCTCAAGGGCCATTT

CACTTGGTATGTCGTGTTGGGATGACTAGATTAGTTGATTAACGGTTGTGATTGTAAATC

AGTGATATGGGCTTCTGTGTACCTTATTTCACAATGATAATCAATTGATACGCTGGATCG

TGATGAGCGGACCGGATGAGCCGTCCATCTTACAGGCTTTTTAAAATAGAATTATAGGGT

GGGTGGTATGAGGTCTACCAAGCCTGTAACATGGAATTACAATCATAGCTGTTAATCGGT

TCATCTCATCCGCTCATCGTGATCCAGCATGTCAAACAGAGATGGGTTTATGGAGAAATG

GATGTATGTAAGCCAACAAATCACAAAAAAATCTGACATAATGATGGAGAGAATGCAAGG

AGTGTAAGTGAATCTACGCCGTTAAATGGATTTCATTGTACTTAATTTCGTTTTGTCAAA

TATTTTGTAGGACGAGGATGATGGACAAGGTGGGCGTAGGAAGAAGGGTTTGACGGAGAA

GATCAAAGAGAAGCTGCCAGGTGGAAACAAGACGACAGGTGTCTGTCATCCAGGGACTCA

GGGAGTACAGGGGGGTCGCGAGCATGAGAAGCCAGGTTGCGGTCAGGGTGGACAGGTGGG

CCGCGAGCATGAGAAGACAGGTTTCGGTCATCCAGGGACTCAGGGTGGACAGGTGGGCAG

CGAGCAGGAGAAGAAGGGTATGATTGAGAAGATCAAGGAGAAGCTGCCAGGTCACAAGTA

GGATGTGTGTCCACCATACACGTGGAGTCTACACCTACGTGTCTTATGTTACTTATAATA

CGCATGGAGTGTGTCCGGAGTCTGTAATAATCTGCGCGTATCTGTTATGTC

>AHJX125

AGCCGTTGGATCTAGTTGAAAATGTCAGAGACCCGTGAGGAGTATGGCAACCAGGTTCGC

CAAACCGACGAGTATGGGAACCCGATTCAGCATACTGGCACCGGGACAAAGCCCGGTTCG

GGCATACATGGTGGGGGCCATGGGATTGGCACAGGTGGTGGTGGTGGACAAGGCAAGCTC

CACCGTCCAGGCTCCGGCTCTTCCTCTGTAAGTATATTCGATGTTTCTCAAGGGCCATTT

CACTTGGTATGTCGTGTTGGGATGACTAGATTAGTTGATTAACGGTTGTGATTGTAAATC

AGTGATATGGGCTTCTGTGTACCTTATTTCACAATGATAATCAATTGATACGCTGGATCG

TGATGAGCGGACCGGATGAGCCGTCCATCTTACAGGCTTTTTAAAATAGAATTATAGGGT

GGGTGGTATGAGGTCTACCAAGCCTGTAACATGGAATTACAATCATAGCTGTTAATCGGT

TCATCTCATCCGCTCATCGTGATCCAGCATGTCAAACAGAGATGGGTTTATGGAGAAATG

GATGTATGTAAGCCAACAAATCACAAAAAAATCTGACATAATGATGGAGAGAATGCAAGG

AGTGTAAGTGAATCTACGCCGTTAAATGGATTTCATTGTACTTAATTTCGTTTTGTCAAA

TATTTTGTAGGACGAGGATGATGGACAAGGTGGGCGTAGGAAGAAGGGTTTGACGGAGAA

GATCAAAGAGAAGCTGCCAGGTGGAAACAAGACGACAGGTGTCTGTCATCCAGGGACTCA

GGGAGTACAGGGGGGTCGCGAGCATGAGAAGCCAGGTTGCGGTCAGGGTGGACAGGTGGG

CCGCGAGCATGAGAAGACAGGTTTCGGTCATCCAGGGACTCAGGGTGGACAGGTGGGCAG

CGAGCAGGAGAAGAAGGGTATGATTGAGAAGATCAAGGAGAAGCTGCCAGGTCACAAGTA

GGATGTGTGTCCACCATACACGTGGAGTCTACACCTACGTGTCTTATGTTACTTATAATA

CGCATGGAGTGTGTCCGGAGTCTGTAATAATCTGCGCGTATCTGTTATGTC

>AHJX126

AGCCGTTGGATCTAGTTGAAAATGTCAGAGACCCGTGAGGAGTATGGCAACCAGGTTCGC

CAAACCGACGAGTATGGGAACCCGATTCAGCATACTGGCACCGGGACAAAGCCCGGTTCG

GGCATACATGGTGGGGGCCATGGGATTGGCACAGGTGGTGGTGGTGGACAAGGCAAGCTC

CACCGTCCAGGCTCCGGCTCTTCCTCTGTAAGTATATTCGATGTTTCTCAAGGGCCATTT

CACTTGGTATGTCGTGTTGGGATGACTAGATTAGTTGATTAACGGTTGTGATTGTAAATC

AGTGATATGGGCTTCTGTGTACCTTATTTCACAATGATAATCAATTGATACGCTGGATCG

TGATGAGCGGACCGGATGAGCCGTCCATCTTACAGGCTTTTTAAAATAGAATTATAGGGT

GGGTGGTATGAGGTCTACCAAGCCTGTAACATGGAATTACAATCATAGCTGTTAATCGGT

TCATCTCATCCGCTCATCGTGATCCAGCATGTCAAACAGAGATGGGTTTATGGAGAAATG

GATGTATGTAAGCCAACAAATCACAAAAAAATCTGACATAATGATGGAGAGAATGCAAGG

AGTGTAAGTGAATCTACGCCGTTAAATGGATTTCATTGTACTTAATTTCGTTTTGTCAAA

TATTTTGTAGGACGAGGATGATGGACAAGGTGGGCGTAGGAAGAAGGGTTTGACGGAGAA

GATCAAAGAGAAGCTGCCAGGTGGAAACAAGACGACAGGTGTCTGTCATCCAGGGACTCA

GGGAGTACAGGGGGGTCGCGAGCATGAGAAGCCAGGTTGCGGTCAGGGTGGACAGGTGGG

CCGCGAGCATGAGAAGACAGGTTTCGGTCATCCAGGGACTCAGGGTGGACAGGTGGGCAG

CGAGCAGGAGAAGAAGGGTATGATTGAGAAGATCAAGGAGAAGCTGCCAGGTCACAAGTA

GGATGTGTGTCCACCATACACGTGGAGTCTACACCTACGTGTCTTATGTTACTTATAATA

CGCATGGAGTGTGTCCGGAGTCTGTAATAATCTGCGCGTATCTGTTATGTC

>AHJX1220

AGCCGTTGGATCTAGTTGAAAATGTCAGAGACCCGTGAGGAGTATGGCAACCAGGTTCGC

CAAACCGACGAGTATGGGAACCCGATTCAGCATACTGGCACCGGGACAAAGCCCGGTTCG

GGCATACATGGTGGGGGCCATGGGATTGGCACAGGTGGTGGTGGTGGACAAGGCAAGCTC

CACCGTCCAGGCTCCGGCTCTTCCTCTGTAAGTATATTCGATGTTTCTCAAGGGCCATTT

CACTTGGTATGTCGTGTTGGGATGACTAGATTAGTTGATTAACGGTTGTGATTGTAAATC

AGTGATATGGGCTTCTGTGTACCTTATTTCACAATGATAATCAATTGATACGCTGGATCG

TGATGAGCGGACCGGATGAGCCGTCCATCTTACAGGCTTTTTAAAATAGAATTATAGGGT

GGGTGGTATGAGGTCTACCAAGCCTGTAACATGGAATTACAATCATAGCTGTTAATCGGT

TCATCTCATCCGCTCATCGTGATCCAGCATGTCAAACAGAGATGGGTTTATGGAGAAATG

GATGTATGTAAGCCAACAAATCACAAAAAAATCTGACATAATGATGGAGAGAATGCAAGG

AGTGTAAGTGAATCTACGCCGTTAAATGGATTTCATTGTACTTAATTTCGTTTTGTCAAA

TATTTTGTAGGACGAGGATGATGGACAAGGTGGGCGTAGGAAGAAGGGTTTGACGGAGAA

GATCAAAGAGAAGCTGCCAGGTGGAAACAAGACGACAGGTGTCTGTCATCCAGGGACTCA

GGGAGTACAGGGGGGTCGCGAGCATGAGAAGCCAGGTTGCGGTCAGGGTGGACAGGTGGG

CCGCGAGCATGAGAAGACAGGTTTCGGTCATCCAGGGACTCAGGGTGGACAGGTGGGCAG

CGAGCAGGAGAAGAAGGGTATGATTGAGAAGATCAAGGAGAAGCTGCCAGGTCACAAGTA

GGATGTGTGTCCACCATACACGTGGAGTCTACACCTACGTGTCTTATGTTACTTATAATA

CGCATGGAGTGTGTCCGGAGTCTGTAATAATCTGCGCGTATCTGTTATGTC

>AHJXQ231

AGCCGTTGGATCTAGTTGAAAATGTCAGAGACGCGTGATGAGTATGGCAACCAGGTTCGC

CAAACCGACGAGTATGGGAACCCGATTCAGCATACTGGCACCGGGACAAAGCCCGGTTCG

GGCATACATGGTGGGGGCCATGGGATTGGCACAGGTGGTGGTGGTGGACAAGGCAAGCTC

CACCGTCCAGGCTCCGGCTCTTCCTCTGTAAGTATATTCGATGTTTCTCAAGGGTCATTT

CACTTGGTATATAGTGTTGGGATGACTAGATTAGTTGATTAACGGTTGTGGTTGTAAATC

AGTGATATGGGCTTCTATGTACCTTATTTCACAATGATAATCAATTGATACGCTGGATCG

TGATGAGCGGACCGGATGAGCCGTCCATCTTACAGGCTTTTTAAAATAGAATTATAGGGT

GGGTGGTATGAGGTCCACCAAGCCTGTAACATGGAATTACAATCATAGCCGTTAATTGGT

TCATCTCATCCGCTCATCGTGATCCAGCATGTCAAACAGAGATGGGTTTCTGGAGAAGTG

GATGTATGTAAGCCCACAAATCACAAAAAAAATCTGACATAATGATGGAGAGAATGCAAG

GAGTGTAAGTGAATCTACGCCGTTAAATGGATTTCATTGTACTTAACTTCGTTTTGTCAA

ATATTTTGTAGGACGAGGATGATGGACAAGGTGGGCGTAGGAAGAAGGGTTTGACGGAGA

AGATCAAAGAGAAGCTGCCAGGTGGAAACAAGACGACAGGTGTCTGTCATCCAGGGACTC

AGGGAGTACAGGGGGGTCGCGAGCATGAGAAGCCAGGTTTCGGTCAGGGTGGACAGGTGG

GCCGCGAGCATGAGAAGACAGGTTTCGGTCATCCAGGGACTCAGGGTGGACAGGTGGGCA

GCGAGCAGGAGAAGAAGGGTATGATTGAGAAGATCAAGGAGAAGCTGCCAGGTCACAAGT

AGGATGTGTGTCCACTATACACGTGGAGTCTGCATCTACGTGTCTTATGTTACTTATAAT

ACGCATGGAGTGTGTCCGGAGTCTGTAATAATCTGCGCGTATCTGTTATGTC

>AHJXQ234

AGCCGTTGGATCTAGTTGAAAATGTCAGAGACGCGTGATGAGTATGGCAACCAGGTTCGC

CAAACCGACGAGTATGGGAACCCGATTCAGCATACTGGCACCGGGACAAAGCCCGGTTCG

GGCATACATGGTGGGGGCCATGGGATTGGCACAGGTGGTGGTGGTGGACAAGGCAAGCTC

CACCGTCCAGGCTCCGGCTCTTCCTCTGTAAGTATATTCGATGTTTCTCAAGGGTCATTT

CACTTGGTATATAGTGTTGGGATGACTAGATTAGTTGATTAACGGTTGTGGTTGTAAATC

AGTGATATGGGCTTCTATGTACCTTATTTCACAATGATAATCAATTGATACGCTGGATCG

TGATGAGCGGACCGGATGAGCCGTCCATCTTACAGGCTTTTTAAAATAGAATTATAGGGT

GGGTGGTATGAGGTCCACCAAGCCTGTAACATGGAATTACAATCATAGCCGTTAATTGGT

TCATCTCATCCGCTCATCGTGATCCAGCATGTCAAACAGAGATGGGTTTCTGGAGAAGTG

GATGTATGTAAGCCCACAAATCACAAAAAAAATCTGACATAATGATGGAGAGAATGCAAG

GAGTGTAAGTGAATCTACGCCGTTAAATGGATTTCATTGTACTTAACTTCGTTTTGTCAA

ATATTTTGTAGGACGAGGATGATGGACAAGGTGGGCGTAGGAAGAAGGGTTTGACGGAGA

AGATCAAAGAGAAGCTGCCAGGTGGAAACAAGACGACAGGTGTCTGTCATCCAGGGACTC

AGGGAGTACAGGGGGGTCGCGAGCATGAGAAGCCAGGTTTCGGTCAGGGTGGACAGGTGG

GCCGCGAGCATGAGAAGACAGGTTTCGGTCATCCAGGGACTCAGGGTGGACAGGTGGGCA

GCGAGCAGGAGAAGAAGGGTATGATTGAGAAGATCAAGGAGAAGCTGCCAGGTCACAAGT

AGGATGTGTGTCCACTATACACGTGGAGTCTGCATCTACGTGTCTTATGTTACTTATAAT

ACGCATGGAGTGTGTCCGGAGTCTGTAATAATCTGCGCGTATCTGTTATGTC

>AHHS27

AGCCGTTGGATCTAGTTGAAAATGTCAGAGACGCGTGATGAGTATGGCAACCAGGTTCGC

CAAACCGACGAGTATGGGAACCCGATTCAGCATAGTGGCACCGGGACAAAGCCCGGTTCG

GGCATACATGGTGGGGGCCATGGGATTGGCACAGGTGGTGGTGGTGGACAAGGCAAGCTC

CACCGTCCAGGCTCCGGCTCTTCCTCTGTAAGTATATTCGATGTTTCTCAAGGGTCATTT

CACTTGGTATATCGTGTTGGTATGACTAGATTAGTTGATTAACGGTTGTGATTGTAAATC

AGTGATATGGGCTTCTGTGTACCTTATTTCACAATGATAATCAATTGATACGCTGGGTCG

TGATGAGCGGACCGGATGAGCCGTCCATCTTACAGGCTTTTTAAAATAGAATTATAGGGT

GGGTGGTATGAGGTCTACCAAGCCTGTAACATGGAATTACAATCATAGCTGTTAATCGGT

TCATCTCATCCGCTCATCGTGATCCAGCATGTCAAACAGAGATGGGTTTATGGAGAAATG

GATGTATGTAAGCCAACAAATCACAAAAAAAATCTGACATAATGATGGAGAGAATGCAAG

GAGTGTAAGTGAATCTACGCCGTTAAATGGATTTCATTGTACTTAATTTCGTTTCCTCAA

ATATTTTGTAGGACGAGGATGATGGACAAGGTGGGCGTAGGAAGAAGGGTTTGACGGAGA

AGATCAAAGAGAAGCTGCCAGGTGGAAACAAGACGACAGGTGTCTGTCATCCAGGGACTC

AGGGAGTACAGGGGGGTCGCGAGCATGAGAAGCCAGGTTGCGGGCAGGGTGGACAGGTGG

GCCGCGAGCATGAGAAGACAGGTTTCGGTCATCCAGGAACTCAGGGTGGACAGGTGGGCA

GCGAGCAGGAGAAGAAGGGTATGATTGAGAAGATCAAGGAGAAGCTGCCAGGTCACAAGT

AGGATGTGTGTCCACCATACACGTGGAGCCTACATCTACGTGTCTTATGTTACTTATAAT

ACGCATGGAGTGTGTCCGGAGTCTGTAATAATCTGCGCGTATCTGTTATGTC

>AHHS29

AGCCGTTGGATCTAGTTGAAAATGTCAGAGACGCGTGATGAGTATGGCAACCAGGTTCGC

CAAACCGACGAGTATGGGAACCCGATTCAGCATAGTGGCACCGGGACAAAGCCCGGTTCG

GGCATACATGGTGGGGGCCATGGGATTGGCACAGGTGGTGGTGGTGGACAAGGCAAGCTC

CACCGTCCAGGCTCCGGCTCTTCCTCTGTAAGTATATTCGATGTTTCTCAAGGGTCATTT

CACTTGGTATATCGTGTTGGTATGACTAGATTAGTTGATTAACGGTTGTGATTGTAAATC

AGTGATATGGGCTTCTGTGTACCTTATTTCACAATGATAATCAATTGATACGCTGGGTCG

TGATGAGCGGACCGGATGAGCCGTCCATCTTACAGGCTTTTTAAAATAGAATTATAGGGT

GGGTGGTATGAGGTCTACCAAGCCTGTAACATGGAATTACAATCATAGCTGTTAATCGGT

TCATCTCATCCGCTCATCGTGATCCAGCATGTCAAACAGAGATGGGTTTATGGAGAAATG

GATGTATGTAAGCCAACAAATCACAAAAAAAATCTGACATAATGATGGAGAGAATGCAAG

GAGTGTAAGTGAATCTACGCCGTTAAATGGATTTCATTGTACTTAATTTCGTTTCCTCAA

ATATTTTGTAGGACGAGGATGATGGACAAGGTGGGCGTAGGAAGAAGGGTTTGACGGAGA

AGATCAAAGAGAAGCTGCCAGGTGGAAACAAGACGACAGGTGTCTGTCATCCAGGGACTC

AGGGAGTACAGGGGGGTCGCGAGCATGAGAAGCCAGGTTGCGGGCAGGGTGGACAGGTGG

GCCGCGAGCATGAGAAGACAGGTTTCGGTCATCCAGGAACTCAGGGTGGACAGGTGGGCA

GCGAGCAGGAGAAGAAGGGTATGATTGAGAAGATCAAGGAGAAGCTGCCAGGTCACAAGT

AGGATGTGTGTCCACCATACACGTGGAGCCTACATCTACGTGTCTTATGTTACTTATAAT

ACGCATGGAGTGTGTCCGGAGTCTGTAATAATCTGCGCGTATCTGTTATGTC

>AHHS210

AGCCGTTGGATCTAGTTGAAAATGTCAGAGACGCGTGATGAGTATGGCAACCAGGTTCGC

CAAACCGACGAGTATGGGAACCCGATTCAGCATAGTGGCACCGGGACAAAGCCCGGTTCG

GGCATACATGGTGGGGGCCATGGGATTGGCACAGGTGGTGGTGGTGGACAAGGCAAGCTC

CACCGTCCAGGCTCCGGCTCTTCCTCTGTAAGTATATTCGATGTTTCTCAAGGGTCATTT

CACTTGGTATATCGTGTTGGTATGACTAGATTAGTTGATTAACGGTTGTGATTGTAAATC

AGTGATATGGGCTTCTGTGTACCTTATTTCACAATGATAATCAATTGATACGCTGGGTCG

TGATGAGCGGACCGGATGAGCCGTCCATCTTACAGGCTTTTTAAAATAGAATTATAGGGT

GGGTGGTATGAGGTCTACCAAGCCTGTAACATGGAATTACAATCATAGCTGTTAATCGGT

TCATCTCATCCGCTCATCGTGATCCAGCATGTCAAACAGAGATGGGTTTATGGAGAAATG

GATGTATGTAAGCCAACAAATCACAAAAAAAATCTGACATAATGATGGAGAGAATGCAAG

GAGTGTAAGTGAATCTACGCCGTTAAATGGATTTCATTGTACTTAATTTCGTTTCCTCAA

ATATTTTGTAGGACGAGGATGATGGACAAGGTGGGCGTAGGAAGAAGGGTTTGACGGAGA

AGATCAAAGAGAAGCTGCCAGGTGGAAACAAGACGACAGGTGTCTGTCATCCAGGGACTC

AGGGAGTACAGGGGGGTCGCGAGCATGAGAAGCCAGGTTGCGGGCAGGGTGGACAGGTGG

GCCGCGAGCATGAGAAGACAGGTTTCGGTCATCCAGGAACTCAGGGTGGACAGGTGGGCA

GCGAGCAGGAGAAGAAGGGTATGATTGAGAAGATCAAGGAGAAGCTGCCAGGTCACAAGT

AGGATGTGTGTCCACCATACACGTGGAGCCTACATCTACGTGTCTTATGTTACTTATAAT

ACGCATGGAGTGTGTCCGGAGTCTGTAATAATCTGCGCGTATCTGTTATGTC

>AHHS63

AGCCGTTGGATCTAGTTGAAAATGTCAGAGACGCGTGATGAGTATGGCAACCAGGTTCGC

CAAACCGACGAGTATGGGAACCCGATTCAGCATAGTGGCACCGGGACAAAGCCCGGTTCG

GGCATACATGGTGGGGGCCATGGGATTGGCACAGGTGGTGGTGGTGGACAAGGCAAGCTC

CACCGTCCAGGCTCCGGCTCTTCCTCTGTAAGTATATTCGATGTTTCTCAAGGGTCATTT

CACTTGGTATATCGTGTTGGTATGACTAGATTAGTTGATTAACGGTTGTGATTGTAAATC

AGTGATATGGGCTTCTGTGTACCTTATTTCACAATGATAATCAATTGATACGCTGGGTCG

TGATGAGCGGACCGGATGAGCCGTCCATCTTACAGGCTTTTTAAAATAGAATTATAGGGT

GGGTGGTATGAGGTCTACCAAGCCTGTAACATGGAATTACAATCATAGCTGTTAATCGGT

TCATCTCATCCGCTCATCGTGATCCAGCATGTCAAACAGAGATGGGTTTATGGAGAAATG

GATGTATGTAAGCCAACAAATCACAAAAAAAATCTGACATAATGATGGAGAGAATGCAAG

GAGTGTAAGTGAATCTACGCCGTTAAATGGATTTCATTGTACTTAATTTCGTTTCCTCAA

ATATTTTGTAGGACGAGGATGATGGACAAGGTGGGCGTAGGAAGAAGGGTTTGACGGAGA

AGATCAAAGAGAAGCTGCCAGGTGGAAACAAGACGACAGGTGTCTGTCATCCAGGGACTC

AGGGAGTACAGGGGGGTCGCGAGCATGAGAAGCCAGGTTGCGGGCAGGGTGGACAGGTGG

GCCGCGAGCATGAGAAGACAGGTTTCGGTCATCCAGGAACTCAGGGTGGACAGGTGGGCA

GCGAGCAGGAGAAGAAGGGTATGATTGAGAAGATCAAGGAGAAGCTGCCAGGTCACAAGT

AGGATGTGTGTCCACCATACACGTGGAGCCTACATCTACGTGTCTTATGTTACTTATAAT

ACGCATGGAGTGTGTCCGGAGTCTGTAATAATCTGCGCGTATCTGTTATGTC

>AHHS66

AGCCGTTGGATCTAGTTGAAAATGTCAGAGACGCGTGATGAGTATGGCAACCAGGTTCGC

CAAACCGACGAGTATGGGAACCCGATTCAGCATAGTGGCACCGGGACAAAGCCCGGTTCG

GGCATACATGGTGGGGGCCATGGGATTGGCACAGGTGGTGGTGGTGGACAAGGCAAGCTC

CACCGTCCAGGCTCCGGCTCTTCCTCTGTAAGTATATTCGATGTTTCTCAAGGGTCATTT

CACTTGGTATATCGTGTTGGTATGACTAGATTAGTTGATTAACGGTTGTGATTGTAAATC

AGTGATATGGGCTTCTGTGTACCTTATTTCACAATGATAATCAATTGATACGCTGGATCG

TGATGAGCGGACCGGATGAGCCGTCCATCTTACAGGCTTTTTAAAATAGAATTATAGGGT

GGGTGGTATGAGGTCTACCAAGCCTGTAACATGGAATTACAATAATAGCTGTTAATCGGT

TCATCTCATCCGCTCATCGTGATCCAGCATGTCAAACAGAGATGGGTTTATGGAGAAATG

GATGTATGTAAGCCAACAAATCACAAAAAAAATCTGACATAATGATGGAGAGAATGCAAG

GAGTGTAAGTGAATCTACGCCGTTAAATGGATTTCATTGTACTTAATTTCGTTTCCTCAA

ATATTTTGTAGGACGAGGATGATGGACAAGGTGGGCGTAGGAAGAAGGGTTTGACGGAGA

AGATCAAAGAGAAGCTGCCAGGTGGAAACAAGACGACAGGTGTCTGTCATCCAGGGACTC

AGGGAGTACAGGGGGGTCGCGAGCATGAGAAGCCAGGTTGCGGGCAGGGTGGACAGGTGG

GCCGCGAGCATGAGAAGACAGGTTTCGGTCATCCAGGAACTCAGGGTGGACAGGTGGGCA

GCGAGCAGGAGAAGAAGGGTATGATTGAGAAGATCAAGGAGAAGCTGCCAGGTCACAAGT

AGGATGTGTGTCCACCATACACGTGGAGCCTACATCTACGTGTCTTATGTTACTTATAAT

ACGCATGGAGTGTGTCCGGAGTCTGTAATAATCTGCGCGTATCTGTTATGTC

>AHHS78

AGCCGTTGGATCTAGTTGAAAATGTCAGAGACGCGTGATGAGTATGGCAACCAGGTTCGC

CAAACCGACGAGTATGGGAACCCGATTCAGCATACTGGCACCGGGACAAAGCCCGGTTCG

GGCATACATGGTGGGGGCCATGGGATTGGCACAGGTGGTGGTGGTGGACAAGGCAAGCTC

CACCGTCCAGGCTCCGGCTCTTCCTCTGTAAGTATATTCGATGTTTCTCAAGGGTCATTT

CACTTGGTATATAGTGTTGGGATGACTAGATTAGTTGATTAACGGTTGTGGTTGTAAATC

AGTGATATGGGCTTCTATGTACCTTATTTCACAATGATAATCAATTGATACGCTGGATCG

TGATGAGCGGACCGGATGAGCCGTCCATCTTACAGGCTTTTTAAAATAGAATTATAGGGT

GGGTGGTATGAGGTCCACCAAGCCTGTAACATGGAATTACAATCATAGCCGTTAATTGGT

TCATCTCATCCGCTCATCGTGATCCAGCATGTCAAACAGAGATGGGTTTCTGGAGAAGTG

GATGTATGTAAGCCCACAAATCACAAAAAAAATCTGACATAATGATGGAGAGAATGCAAG

GAGTGTAAGTGAATCTACGCCGTTAAATGGATTTCATTGTACTTAACTTCGTTTTGTCAA

ATATTTTGTAGGACGAGGATGATGGACAAGGTGGGCGTAGGAAGAAGGGTTTGACGGAGA

AGATCAAAGAGAAGCTGCCAGGTGGAAACAAGACGACAGGTGTCTGTCATCCAGGGACTC

AGGGAGTACAGGGGGGTCGCGAGCATGAGAAGCCAGGTTTCGGTCAGGGTGGACAGGTGG

GCCGCGAGCATGAGAAGACAGGTTTCGGTCATCCAGGGACTCAGGGTGGACAGGTGGGCA

GCGAGCAGGAGAAGAAGGGTATGATTGAGAAGATCAAGGAGAAGCTGCCAGGTCACAAGT

AGGATGTGTGTCCACTATACACGTGGAGTCTGCATCTACGTGTCTTATGTTACTTATAAT

ACGCATGGAGTGTGTCCGGAGTCTGTAATAATCTGCGCGTATCTGTTATGTC

>AHHS710

AGCCGTTGGATCTAGTTGAAAATGTCAGAGACGCGTGATGAGTATGGCAACCAGGTTCGC

CAAACCGACGAGTATGGGAACCCGATTCAGCATACTGGCACCGGGACAAAGCCCGGTTCG

GGCATACATGGTGGGGGCCATGGGATTGGCACAGGTGGTGGTGGTGGACAAGGCAAGCTC

CACCGTCCAGGCTCCGGCTCTTCCTCTGTAAGTATATTCGATGTTTCTCAAGGGTCATTT

CACTTGGTATATAGTGTTGGGATGACTAGATTAGTTGATTAACGGTTGTGGTTGTAAATC

AGTGATATGGGCTTCTATGTACCTTATTTCACAATGATAATCAATTGATACGCTGGATCG

TGATGAGCGGACCGGATGAGCCGTCCATCTTACAGGCTTTTTAAAATAGAATTATAGGGT

GGGTGGTATGAGGTCCACCAAGCCTGTAACATGGAATTACAATCATAGCCGTTAATTGGT

TCATCTCATCCGCTCATCGTGATCCAGCATGTCAAACAGAGATGGGTTTCTGGAGAAGTG

GATGTATGTAAGCCCACAAATCACAAAAAAAATCTGACATAATGATGGAGAGAATGCAAG

GAGTGTAAGTGAATCTACGCCGTTAAATGGATTTCATTGTACTTAACTTCGTTTTGTCAA

ATATTTTGTAGGACGAGGATGATGGACAAGGTGGGCGTAGGAAGAAGGGTTTGACGGAGA

AGATCAAAGAGAAGCTGCCAGGTGGAAACAAGACGACAGGTGTCTGTCATCCAGGGACTC

AGGGAGTACAGGGGGGTCGCGAGCATGAGAAGCCAGGTTTCGGTCAGGGTGGACAGGTGG

GCCGCGAGCATGAGAAGACAGGTTTCGGTCATCCAGGGACTCAGGGTGGACAGGTGGGCA

GCGAGCAGGAGAAGAAGGGTATGATTGAGAAGATCAAGGAGAAGCTGCCAGGTCACAAGT

AGGATGTGTGTCCACTATACACGTGGAGTCTGCATCTACGTGTCTTATGTTACTTATAAT

ACGCATGGAGTGTGTCCGGAGTCTGTAATAATCTGCGCGTATCTGTTATGTC

>JXLS23

AGCCGTTGGATCTAGTTGAAAATGTCAGAGACGCGTGATGAGTATGGCAACCAGGTTCGC

CAAACCGACGAGTATGGGAACCCGATTCAGCATAGTGGCACCGGGACAAAGCCCGGTTCG

GGCATACATGGTGGGGGCCATGGGATTGGCACAGGTGGTGGTGGTGGACAAGGCAAGCTC

CACCGTCCAGGCTCCGGCTCTTCCTCTGTAAGTATATTCGATGTTTCTCAAGGGCCATTT

CACTTGGTATATCGTGTTGGGATGACTAGATTAGTTGATTAACGGTTGTGATTGTAAATC

AGTGATATGGGCTTCTGTGTACCTTATTTCACAATGATAATCAATTGATACGCTGGATCG

TGATGAGCGGACCGGATGAGCCGTCCATCTTACAGGCTTTTTAAAATAGAATTATAGGGT

GGGTGGTATGAGGTCTACCAAGCCTGTAACATGGAATTACAATCATAGCTGTTAATCGGT

TCATCTCATCCGCTCATCGTGATCCAGCATGTCAAACAGAGATGGGTTTATGGAGAAATG

GATGTATGTAAGCCAAAAAATCACAAAAAAATCTGACATAATGATGGAGAGAATGCAAGG

AGTGTAAGTGAATCTACGCCGTTAAATGGATTTCATTGTACTTAATTTCGTTTTGTCAAA

TATTTTGTAGGACGAGGATGATGGACAAGGTGGGCGTAGGAAGAAGGGTTTGACGGAGAA

GATCAAAGAGAAGCTGCCAGGTGGAAACAAGACGACAGGTGTCTGTCATCCAGGGACTCA

GGGAGTACAGGGGGGTCGTGAGCATGAGAAGCCAGGTTGCGGTCAGGGTGGACAGGTGGG

CCGCGAGCATGAGAAGACAGGTTTCGGTCATCCAGGGACTCAGGGTGGACAGGTGGGCAG

CGAGCAGGAGAAGAAGGGTATGATTGAGAAGATCAAGGAGAAGCTGCCAGGTCACAAGTA

GGATGTGTGTCCACCATACACGTGGAGTCTACACCTACGTGTCTTATGTTACTTATAATA

CGCATGGAGTGTGTCCGGAGTCTGTAATAATCTGCGCGTATCTGTTATGTC

>JXLS25

AGCCGTTGGATCTAGTTGAAAATGTCAGAGACGCGTGATGAGTATGGCAACCAGGTTCGC

CAAACCGACGAGTATGGGAACCCGATTCAGCATAGTGGCACCGGGACAAAGCCCGGTTCG

GGCATACATGGTGGGGGCCATGGGATTGGCACAGGTGGTGGTGGTGGACAAGGCAAGCTC

CACCGTCCAGGCTCCGGCTCTTCCTCTGTAAGTATATTCGATGTTTCTCAAGGGCCATTT

CACTTGGTATATCGTGTTGGGATGACTAGATTAGTTGATTAACGGTTGTGATTGTAAATC

AGTGATATGGGCTTCTGTGTACCTTATTTCACAATGATAATCAATTGATACGCTGGATCG

TGATGAGCGGACCGGATGAGCCGTCCATCTTACAGGCTTTTTAAAATAGAATTATAGGGT

GGGTGGTATGAGGTCTACCAAGCCTGTAACATGGAATTACAATCATAGCTGTTAATCGGT

TCATCTCATCCGCTCATCGTGATCCAGCATGTCAAACAGAGATGGGTTTATGGAGAAATG

GATGTATGTAAGCCAAAAAATCACAAAAAAATCTGACATAATGATGGAGAGAATGCAAGG

AGTGTAAGTGAATCTACGCCGTTAAATGGATTTCATTGTACTTAATTTCGTTTTGTCAAA

TATTTTGTAGGACGAGGATGATGGACAAGGTGGGCGTAGGAAGAAGGGTTTGACGGAGAA

GATCAAAGAGAAGCTGCCAGGTGGAAACAAGACGACAGGTGTCTGTCATCCAGGGACTCA

GGGAGTACAGGGGGGTCGTGAGCATGAGAAGCCAGGTTGCGGTCAGGGTGGACAGGTGGG

CCGCGAGCATGAGAAGACAGGTTTCGGTCATCCAGGGACTCAGGGTGGACAGGTGGGCAG

CGAGCAGGAGAAGAAGGGTATGATTGAGAAGATCAAGGAGAAGCTGCCAGGTCACAAGTA

GGATGTGTGTCCACCATACACGTGGAGTCTACACCTACGTGTCTTATGTTACTTATAATA

CGCATGGAGTGTGTCCGGAGTCTGTAATAATCTGCGCGTATCTGTTATGTC

>JXLS27

AGCCGTTGGATCTAGTTGAAAATGTCAGAGACGCGTGATGAGTATGGCAACCAGGTTCGC

CAAACCGACGAGTATGGGAACCCGATTCAGCATAGTGGCACCGGGACAAAGCCCGGTTCG

GGCATACATGGTGGGGGCCATGGGATTGGCACAGGTGGTGGTGGTGGACAAGGCAAGCTC

CACCGTCCAGGCTCCGGCTCTTCCTCTGTAAGTATATTCGATGTTTCTCAAGGGCCATTT

CACTTGGTATATCGTGTTGGGATGACTAGATTAGTTGATTAACGGTTGTGATTGTAAATC

AGTGATATGGGCTTCTGTGTACCTTATTTCACAATGATAATCAATTGATACGCTGGATCG

TGATGAGCGGACCGGATGAGCCGTCCATCTTACAGGCTTTTTAAAATAGAATTATAGGGT

GGGTGGTATGAGGTCTACCAAGCCTGTAACATGGAATTACAATCATAGCTGTTAATCGGT

TCATCTCATCCGCTCATCGTGATCCAGCATGTCAAACAGAGATGGGTTTATGGAGAAATG

GATGTATGTAAGCCAAAAAATCACAAAAAAATCTGACATAATGATGGAGAGAATGCAAGG

AGTGTAAGTGAATCTACGCCGTTAAATGGATTTCATTGTACTTAATTTCGTTTTGTCAAA

TATTTTGTAGGACGAGGATGATGGACAAGGTGGGCGTAGGAAGAAGGGTTTGACGGAGAA

GATCAAAGAGAAGCTGCCAGGTGGAAACAAGACGACAGGTGTCTGTCATCCAGGGACTCA

GGGAGTACAGGGGGGTCGTGAGCATGAGAAGCCAGGTTGCGGTCAGGGTGGACAGGTGGG

CCGCGAGCATGAGAAGACAGGTTTCGGTCATCCAGGGACTCAGGGTGGACAGGTGGGCAG

CGAGCAGGAGAAGAAGGGTATGATTGAGAAGATCAAGGAGAAGCTGCCAGGTCACAAGTA

GGATGTGTGTCCACCATACACGTGGAGTCTACACCTACGTGTCTTATGTTACTTATAATA

CGCATGGAGTGTGTCCGGAGTCTGTAATAATCTGCGCGTATCTGTTATGTC

>JXLS31

AGCCGTTGGATCTAGTTGAAAATGTCAGAGACGCGTGATGAGTATGGCAACCAGGTTCGC

CAAACCGACGAGTATGGGAACCCGATTCAGCATAGTGGCACCGGGACAAAGCCCGGTTCG

GGCATACATGGTGGGGGCCATGGGATTGGCACAGGTGGTGGTGGTGGACAAGGCAAGCTC

CACCGTCCAGGCTCCGGCTCTTCCTCTGTAAGTATATTCGATGTTTCTCAAGGGCCATTT

CACTTGGTATATCGTGTTGGGATGACTAGATTAGTTGATTAACGGTTGTGATTGTAAATC

AGTGATATGGGCTTCTGTGTACCTTATTTCACAATGATAATCAATTGATACGCTGGATCG

TGATGAGCGGACCGGATGAGCCGTCCATCTTACAGGCTTTTTAAAATAGAATTATAGGGT

GGGTGGTATGAGGTCTACCAAGCCTGTAACATGGAATTACAATCATAGCTGTTAATCGGT

TCATCTCATCCGCTCATCGTGATCCAGCATGTCAAACAGAGATGGGTTTATGGAGAAATG

GATGTATGTAAGCCAAAAAATCACAAAAAAATCTGACATAATGATGGAGAGAATGCAAGG

AGTGTAAGTGAATCTACGCCGTTAAATGGATTTCATTGTACTTAATTTCGTTTTGTCAAA

TATTTTGTAGGACGAGGATGATGGACAAGGTGGGCGTAGGAAGAAGGGTTTGACGGAGAA

GATCAAAGAGAAGCTGCCAGGTGGAAACAAGACGACAGGTGTCTGTCATCCAGGGACTCA

GGGAGTACAGGGGGGTCGTGAGCATGAGAAGCCAGGTTGCGGTCAGGGTGGACAGGTGGG

CCGCGAGCATGAGAAGACAGGTTTCGGTCATCCAGGGACTCAGGGTGGACAGGTGGGCAG

CGAGCAGGAGAAGAAGGGTATGATTGAGAAGATCAAGGAGAAGCTGCCAGGTCACAAGTA

GGATGTGTGTCCACCATACACGTGGAGTCTACACCTACGTGTCTTATGTTACTTATAATA

CGCATGGAGTGTGTCCGGAGTCTGTAATAATCTGCGCGTATCTGTTATGTC

>JXLS34

AGCCGTTGGATCTAGTTGAAAATGTCAGAGACGCGTGATGAGTATGGCAACCAGGTTCGC

CAAACCGACGAGTATGGGAACCCGATTCAGCATAGTGGCACCGGGACAAAGCCCGGTTCG

GGCATACATGGTGGGGGCCATGGGATTGGCACAGGTGGTGGTGGTGGACAAGGCAAGCTC

CACCGTCCAGGCTCCGGCTCTTCCTCTGTAAGTATATTCGATGTTTCTCAAGGGCCATTT

CACTTGGTATATCGTGTTGGGATGACTAGATTAGTTGATTAACGGTTGTGATTGTAAATC

AGTGATATGGGCTTCTGTGTACCTTATTTCACAATGATAATCAATTGATACGCTGGATCG

TGATGAGCGGACCGGATGAGCCGTCCATCTTACAGGCTTTTTAAAATAGAATTATAGGGT

GGGTGGTATGAGGTCTACCAAGCCTGTAACATGGAATTACAATCATAGCTGTTAATCGGT

TCATCTCATCCGCTCATCGTGATCCAGCATGTCAAACAGAGATGGGTTTATGGAGAAATG

GATGTATGTAAGCCAAAAAATCACAAAAAAATCTGACATAATGATGGAGAGAATGCAAGG

AGTGTAAGTGAATCTACGCCGTTAAATGGATTTCATTGTACTTAATTTCGTTTTGTCAAA

TATTTTGTAGGACGAGGATGATGGACAAGGTGGGCGTAGGAAGAAGGGTTTGACGGAGAA

GATCAAAGAGAAGCTGCCAGGTGGAAACAAGACGACAGGTGTCTGTCATCCAGGGACTCA

GGGAGTACAGGGGGGTCGTGAGCATGAGAAGCCAGGTTGCGGTCAGGGTGGACAGGTGGG

CCGCGAGCATGAGAAGACAGGTTTCGGTCATCCAGGGACTCAGGGTGGACAGGTGGGCAG

CGAGCAGGAGAAGAAGGGTATGATTGAGAAGATCAAGGAGAAGCTGCCAGGTCACAAGTA

GGATGTGTGTCCACCATACACGTGGAGTCTACACCTACGTGTCTTATGTTACTTATAATA

CGCATGGAGTGTGTCCGGAGTCTGTAATAATCTGCGCGTATCTGTTATGTC

>JXLS38

AGCCGTTGGATCTAGTTGAAAATGTCAGAGACGCGTGATGAGTATGGCAACCAGGTTCGC

CAAACCGACGAGTATGGGAACCCGATTCAGCATAGTGGCACCGGGACAAAGCCCGGTTCG

GGCATACATGGTGGGGGCCATGGGATTGGCACAGGTGGTGGTGGTGGACAAGGCAAGCTC

CACCGTCCAGGCTCCGGCTCTTCCTCTGTAAGTATATTCGATGTTTCTCAAGGGCCATTT

CACTTGGTATATCGTGTTGGGATGACTAGATTAGTTGATTAACGGTTGTGATTGTAAATC

AGTGATATGGGCTTCTGTGTACCTTATTTCACAATGATAATCAATTGATACGCTGGATCG

TGATGAGCGGACCGGATGAGCCGTCCATCTTACAGGCTTTTTAAAATAGAATTATAGGGT

GGGTGGTATGAGGTCTACCAAGCCTGTAACATGGAATTACAATCATAGCTGTTAATCGGT

TCATCTCATCCGCTCATCGTGATCCAGCATGTCAAACAGAGATGGGTTTATGGAGAAATG

GATGTATGTAAGCCAAAAAATCACAAAAAAATCTGACATAATGATGGAGAGAATGCAAGG

AGTGTAAGTGAATCTACGCCGTTAAATGGATTTCATTGTACTTAATTTCGTTTTGTCAAA

TATTTTGTAGGACGAGGATGATGGACAAGGTGGGCGTAGGAAGAAGGGTTTGACGGAGAA

GATCAAAGAGAAGCTGCCAGGTGGAAACAAGACGACAGGTGTCTGTCATCCAGGGACTCA

GGGAGTACAGGGGGGTCGTGAGCATGAGAAGCCAGGTTGCGGTCAGGGTGGACAGGTGGG

CCGCGAGCATGAGAAGACAGGTTTCGGTCATCCAGGGACTCAGGGTGGACAGGTGGGCAG

CGAGCAGGAGAAGAAGGGTATGATTGAGAAGATCAAGGAGAAGCTGCCAGGTCACAAGTA

GGATGTGTGTCCACCATACACGTGGAGTCTACACCTACGTGTCTTATGTTACTTATAATA

CGCATGGAGTGTGTCCGGAGTCTGTAATAATCTGCGCGTATCTGTTATGTC

>JXLS55

AGCCGTTGGATCTAGTTGAAAATGTCAGAGACGCGTGATGAGTATGGCAACCAGGTTCGC

CAAACCGACGAGTATGGGAACCCGATTCAGCATACTGGCACCGGGACAAAGCCCGGTTCG

GGCATACATGGTGGGGGCCATGGGATTGGCACAGGTGGTGGTGGTGGACAAGGCAAGCTC

CACCGTCCAGGCTCCGGCTCTTCCTCTGTAAGTATATTCGATGTTTCTCAAGGGCCATTT

CACTTGGTATGTCGTGTTGGGATGACTAGATTAGTTGATTAACGGTTGTGATTGTAAATC

AGTGATATGGGCTTCTGTGTACCTTATTTCACAATGATAATCAATTGATACGCTGGATCG

TGATGAGCGGACCGGATGAGCCGTCCATCTTACAGGCTTTTTAAAATAGAATTATAGGGT

GGGTGGTATGAGGTCTACCAAGCCTGTAACATGGAATTACAATAATAGCTGTTAATCGGT

TCATCTCATCCGCTCATCGTGATCCAGCATGTCAAACAGAGATGGGTTTATGGAGAAATG

GATGTATGTAAGCCAACAAATCACAAAAAAAATCTGACATAATGATGGAGAGAATGCAAG

GAGTGTAAGTGAATCTACGCCGTTAAATGGATTTCATTGTACTTAATTTCGTTTTGTCAA

ATATTTTGTAGGACGAGGATGATGGACAAGGTGGGCGTAGGAAGAAGGGTTTGACGGAGA

AGATCAAAGAGAAGCTGCCAGGTGGAAACAAGACGACAGGTGTCTGTCATCCAGGGACTC

AGGGAGTACAGGGGGGTCGCGAGCATGAGAAGCCAGGTTGCGGGCAGGGTGGACAGGTGG

GCCGCGAGCATGAGAAGACAGGTTTCGGTCATCCAGGAACTCAGGGTGGACAGGTGGGCA

GCGAGCAGGAGAAGAAGGGTATGATTGAGAAGATCAAGGAGAAGCTGCCAGGTCACAAGT

AGGATGTGTGTCCACCATACACGTGGAGCCTACATCTACGTGTCTTATGTTACTTATAAT

ACGCATGGAGTGTGTCCGGAGTCTGTAATAATCTGCGCGTATCTGTTATGTC

>JXLS510

AGCCGTTGGATCTAGTTGAAAATGTCAGAGACGCGTGATGAGTATGGCAACCAGGTTCGC

CAAACCGACGAGTATGGGAACCCGATTCAGCATACTGGCACCGGGACAAAGCCCGGTTCG

GGCATACATGGTGGGGGCCATGGGATTGGCACAGGTGGTGGTGGTGGACAAGGCAAGCTC

CACCGTCCAGGCTCCGGCTCTTCCTCTGTAAGTATATTCGATGTTTCTCAAGGGCCATTT

CACTTGGTATGTCGTGTTGGGATGACTAGATTAGTTGATTAACGGTTGTGATTGTAAATC

AGTGATATGGGCTTCTGTGTACCTTATTTCACAATGATAATCAATTGATACGCTGGATCG

TGATGAGCGGACCGGATGAGCCGTCCATCTTACAGGCTTTTTAAAATAGAATTATAGGGT

GGGTGGTATGAGGTCTACCAAGCCTGTAACATGGAATTACAATAATAGCTGTTAATCGGT

TCATCTCATCCGCTCATCGTGATCCAGCATGTCAAACAGAGATGGGTTTATGGAGAAATG

GATGTATGTAAGCCAACAAATCACAAAAAAAATCTGACATAATGATGGAGAGAATGCAAG

GAGTGTAAGTGAATCTACGCCGTTAAATGGATTTCATTGTACTTAATTTCGTTTTGTCAA

ATATTTTGTAGGACGAGGATGATGGACAAGGTGGGCGTAGGAAGAAGGGTTTGACGGAGA

AGATCAAAGAGAAGCTGCCAGGTGGAAACAAGACGACAGGTGTCTGTCATCCAGGGACTC

AGGGAGTACAGGGGGGTCGCGAGCATGAGAAGCCAGGTTGCGGGCAGGGTGGACAGGTGG

GCCGCGAGCATGAGAAGACAGGTTTCGGTCATCCAGGAACTCAGGGTGGACAGGTGGGCA

GCGAGCAGGAGAAGAAGGGTATGATTGAGAAGATCAAGGAGAAGCTGCCAGGTCACAAGT

AGGATGTGTGTCCACCATACACGTGGAGCCTACATCTACGTGTCTTATGTTACTTATAAT

ACGCATGGAGTGTGTCCGGAGTCTGTAATAATCTGCGCGTATCTGTTATGTC

>JXLS513

AGCCGTTGGATCTAGTTGAAAATGTCAGAGACGCGTGATGAGTATGGCAACCAGGTTCGC

CAAACCGACGAGTATGGGAACCCGATTCAGCATACTGGCACCGGGACAAAGCCCGGTTCG

GGCATACATGGTGGGGGCCATGGGATTGGCACAGGTGGTGGTGGTGGACAAGGCAAGCTC

CACCGTCCAGGCTCCGGCTCTTCCTCTGTAAGTATATTCGATGTTTCTCAAGGGCCATTT

CACTTGGTATGTCGTGTTGGGATGACTAGATTAGTTGATTAACGGTTGTGATTGTAAATC

AGTGATATGGGCTTCTGTGTACCTTATTTCACAATGATAATCAATTGATACGCTGGATCG

TGATGAGCGGACCGGATGAGCCGTCCATCTTACAGGCTTTTTAAAATAGAATTATAGGGT

GGGTGGTATGAGGTCTACCAAGCCTGTAACATGGAATTACAATAATAGCTGTTAATCGGT

TCATCTCATCCGCTCATCGTGATCCAGCATGTCAAACAGAGATGGGTTTATGGAGAAATG

GATGTATGTAAGCCAACAAATCACAAAAAAAATCTGACATAATGATGGAGAGAATGCAAG

GAGTGTAAGTGAATCTACGCCGTTAAATGGATTTCATTGTACTTAATTTCGTTTTGTCAA

ATATTTTGTAGGACGAGGATGATGGACAAGGTGGGCGTAGGAAGAAGGGTTTGACGGAGA

AGATCAAAGAGAAGCTGCCAGGTGGAAACAAGACGACAGGTGTCTGTCATCCAGGGACTC

AGGGAGTACAGGGGGGTCGCGAGCATGAGAAGCCAGGTTGCGGGCAGGGTGGACAGGTGG

GCCGCGAGCATGAGAAGACAGGTTTCGGTCATCCAGGAACTCAGGGTGGACAGGTGGGCA

GCGAGCAGGAGAAGAAGGGTATGATTGAGAAGATCAAGGAGAAGCTGCCAGGTCACAAGT

AGGATGTGTGTCCACCATACACGTGGAGCCTACATCTACGTGTCTTATGTTACTTATAAT

ACGCATGGAGTGTGTCCGGAGTCTGTAATAATCTGCGCGTATCTGTTATGTC

>FJWYS5122

AGCCGTTGGATCTAGTTGAAAATGTCAGAGACGCGTGATGAGTATGGCAACCAGGTTCGC

CAAACCGACGAGTATGGGAACCCGATTCAGCATACTGGCACCGGGACAAAGCCCGGTTCG

GGCATACATGGTGGGGGCCATGGGATTGGCACAGGTGGTGGTGGTGGACAAGGCAAGCTC

CACCGTCCAGGCTCCGGCTCTTCCTCTGTAAGTATATTCGATGTTTCTCAAGGGCCATTT

CACTTGGTATGTCGTGTTGGGATGACTAGATTAGTTGATTAACGGTTGTGATTGTAAATC

AGTGATATGGGCTTCTGTGTACCTTATTTCACAATGATAATCAATTGATACGCTGGATCG

TGATGAGCGGACCGGATGAGCCGTCCATCTTACAGGCTTTTTAAAATAGAATTATAGGGT

GGGTGGTATGAGGTCTACCAAGCCTGTAACATGGAATTACAATCATAGCCGTTAATCGGT

TCATCTCATCCGCTCATCGTGATCCAGCATGTCAAACAGAGATGGGTTTATGGAGAAATG

GATGTATGTAAGCCAACAAACCACAAAAAAAATCTGACATAATGATGGAGAGAATGCAAG

GAGTGTAAGTGAATCTACGCCGTTAAATGGATTTCATTGTACTTAATTTCGTTTTGTCAA

ATATTTTGTAGGACGAGGATGATGGACAAGGTGGGCGTAGGAAGAAGGGTTTGACGGAGA

AGATCAAAGAGAAGCTGCCAGGTGGAAACAAGACGACAGGTGTCTGTCATCCAGGGACTA

AGGGAGTACAGGGGGGTCGCGAGCATGAGAAGCCAGGTTGCGGTCAGGGTGGACAGGTGG

GCCGCGAGCATGAGAAGACAGGTTTGGGTCATCCAGGGACTCAGGGTGGACAGGTGGGCA

GCGAGCAGGAGAAGAAGGGTATGATTGAGAAGATCAAGGAGAAGCTGCCAGGTCACAAGT

AGGATGTGTGTCCACCATACACGTGGAGTCTGCATCTACGTGTCTTATGTTACTTATAAT

ACGCATGGAGTGTGTCCGGAGTCTGTAATAATCTGCGCGTATCTGTTATGTC

>FJWYS5123

AGCCGTTGGATCTAGTTGAAAATGTCAGAGACGCGTGATGAGTATGGCAACCAGGTTCGC

CAAACCGACGAGTATGGGAACCCGATTCAGCATACTGGCACCGGGACAAAGCCCGGTTCG

GGCATACATGGTGGGGGCCATGGGATTGGCACAGGTGGTGGTGGTGGACAAGGCAAGCTC

CACCGTCCAGGCTCCGGCTCTTCCTCTGTAAGTATATTCGATGTTTCTCAAGGGCCATTT

CACTTGGTATATCGTGTTGGGATGACTAGATTAGTTGATTAACGGTTGTGATTGTAAATC

AGTGATATGAGCTTCTGTGTACCTTATTTCACAATGATAATCAATTGATACGCTGGATCG

TGATGAGCGGACCGGATGAGCCGTCCATCTTACAGGCTTTTTAAAATAGAATTATAGGGT

GGGTGGTATGAGGTCTACCAAGCCTGTAACATGGAATTACAATCATAGCTGTTAATCGGT

TCATCTCATCCGCTCATCGTGATCCAGCATGTCAAACAGAGATGGGTTTATGGAGAAATG

GATGTATGTAAGCCAACAAATCACAAAAAAATCTGACATAATGATGGAGAGAATGCAAGG

AGTGTAAGTGAATCTACGCCGTTAAATGGATTTCATTGTACTTAATTTCGTTTTGTCAAA

TATTTTGTAGGACGAGGATGATGGACAAGGTGGGCGTAGGAAGAAGGGTTTGACGGAGAA

GATCAAAGAGAAGCTGCCAGGTGGAAACAAGACGACAGGTGTCTGTCATCCAGGGACTCA

GGGAGTACAGGGGGGTCGCGAGCATGAGAAGCCAGGTTGCGGTCAGGGTGGACAGGTGGG

CCGCGAGCATGAGAAGACAGGTTTCGGTCATCCAGGGACTCAGGGTGGACAGGTGGGCAG

CGAGCAGGAGAAGAAGGGTATGATTGAGAAGATCAAGGAGAAGCTGCCAGGTCACAAGTA

GGATGTGTGTCCACCATACACGTGGAGTCTACACCTACGTGTCTTATGTTACTTATAATA

CGCATGGAGTGTGTCCGGAGTCTGTAATAATCTGCGCGTATCTGTTATGTC

>FJWYS5128

AGCCGTTGGATCTAGTTGAAAATGTCAGAGACGCGTGATGAGTATGGCAACCAGGTTCGC

CAAACCGACGAGTATGGGAACCCGATTCAGCATACTGGCACCGGGACAAAGCCCGGTTCG

GGCATACATGGTGGGGGCCATGGGATTGGCACAGGTGGTGGTGGTGGACAAGGCAAGCTC

CACCGTCCAGGCTCCGGCTCTTCCTCTGTAAGTATATTCGATGTTTCTCAAGGGCCATTT

CACTTGGTATGTCGTGTTGGGATGACTAGATTAGTTGATTAACGGTTGTGATTGTAAATC

AGTGATATGGGCTTCTGTGTACCTTATTTCACAATGATAATCAATTGATACGCTGGATCG

TGATGAGCGGACCGGATGAGCCGTCCATCTTACAGGCTTTTTAAAATAGAATTATAGGGT

GGGTGGTATGAGGTCTACCAAGCCTGTAACATGGAATTACAATCATAGCCGTTAATCGGT

TCATCTCATCCGCTCATCGTGATCCAGCATGTCAAACAGAGATGGGTTTATGGAGAAATG

GATGTATGTAAGCCAACAAACCACAAAAAAAATCTGACATAATGATGGAGAGAATGCAAG

GAGTGTAAGTGAATCTACGCCGTTAAATGGATTTCATTGTACTTAATTTCGTTTTGTCAA

ATATTTTGTAGGACGAGGATGATGGACAAGGTGGGCGTAGGAAGAAGGGTTTGACGGAGA

AGATCAAAGAGAAGCTGCCAGGTGGAAACAAGACGACAGGTGTCTGTCATCCAGGGACTC

AGGGAGTACAGGGGGGTCGCGAGCATGAGAAGCCAGGTTGCGGTCAGGGTGGACAGGTGG

GCCGCGAGCATGAGAAGACAGGTTTGGGTCATCCAGGGACTCAGGGTGGACAGGTGGGCA

GCGAGCAGGAGAAGAAGGGTATGATTGAGAAGATCAAGGAGAAGCTGCCAGGTCACAAGT

AGGATGTGTGTCCACCATACACGTGGAGTCTGCATCTACGTGTCTTATGTTACTTATAAT

ACGCATGGAGTGTGTCCGGAGTCTGTAATAATCTGCGCGTATCTGTTATGTC

>FJWYS24

AGCCGTTGGATCTAGTTGAAAATGTCAGAGACGCGTGATGAGTATGGCAACCAGGTTCGC

CAAACCGACGAGTATGGGAACCCGATTCAGCATAGTGGCACCGGGACAAAGCCCGGTTCG

GGCATACATGGTGGGGGCCATGGGATTGGCACAGGTGGTGGTGGTGGACAAGGCAAGCTC

CACCGTCCAGGCTCCGGCTCTTCCTCTGTAAGTATATTCGATGTTTCTCAAGGGTCATTT

CACTTGGTATATCGTGTTGGGATGACTAGATTAGTTGATTAACGGTTGTGATTGTAAATC

AGTGATATGGGCTTCTGTGTACCTTATTTCACAATGATAATCAATTGATACGCTGGATCG

TGATGAGCGGACCGGATGAGCCGTCCATCTTACAGGCTTTTTAAAATAGAATTATAGGGT

GGGTGGTATGAGGTCTACCAAGCCTGTAACATGGAATTACAATCATAGCCGTTAATCGGT

TCATCTCATCCGCTCATCGTGATCCAGCATGTCAAACAGAGATGGGTTTATGGAGAAATG

GATGTATGTAAGCCAACAAATCACAAAAAAAATCTGACATAATGATGGAGAGAATGCAAG

GAGTGTAAGTGAATCTACGCCGTTAAATGGATTTCATTGTACTTAACTTCGTTTTGTCAA

ATATTTTGTAGGACGAGGATGATGGACAAGGTGGGCGTAGGAAGAAGGGTTTGACGGAGA

AGATCAAAGAGAAGCTGCCAGGTGGAAACAAGACGACAGGTGTCTGTCATCCAGGGACTC

AGGGAGTACAGGGGGGTCGCGAGCATGAGAAGCCAGGTTGCGGGCAGGGTGGACAGGTGG

GCCGCGAGCATGAGAAGACAGGTTTCGGTCATCCAGGAACTCAGGGTGGACAGGTGGGCA

GCGAGCAGGAGAAGAAGGGTATGATTGAGAAGATCAAGGAGAAGCTGCCAGGTCACAAGT

AGGATGTGTGTCCACCATACACGTGGAGTCTACACCTACGTGTCTTATGTTACTTATAAT

ACGCATGGAGTGTGTCCGGAGTCTGTAATAATCTGCGCGTATCTGTTATGTC

>FJWYS27

AGCCGTTGGATCTAGTTGAAAATGTCAGAGACGCGTGATGAGTATGGCAACCAGGTTCGC

CAAACCGACGAGTATGGGAACCCGATTCAGCATAGTGGCACCGGGACAAAGCCCGGTTCG

GGCATACATGGTGGGGGCCATGGGATTGGCACAGGTGGTGGTGGTGGACAAGGCAAGCTC

CACCGTCCAGGCTCCGGCTCTTCCTCTGTAAGTATATTCGATGTTTCTCAAGGGTCATTT

CACTTGGTATATCGTGTTGGGATGACTAGATTAGTTGATTAACGGTTGTGATTGTAAATC

AGTGATATGGGCTTCTGTGTACCTTATTTCACAATGATAATCAATTGATACGCTGGATCG

TGATGAGCGGACCGGATGAGCCGTCCATCTTACAGGCTTTTTAAAATAGAATTATAGGGT

GGGTGGTATGAGGTCTACCAAGCCTGTAACATGGAATTACAATCATAGCCGTTAATCGGT

TCATCTCATCCGCTCATCGTGATCCAGCATGTCAAACAGAGATGGGTTTATGGAGAAATG

GATGTATGTAAGCCAACAAATCACAAAAAAAATCTGACATAATGATGGAGAGAATGCAAG

GAGTGTAAGTGAATCTACGCCGTTAAATGGATTTCATTGTACTTAACTTCGTTTTGTCAA

ATATTTTGTAGGACGAGGATGATGGACAAGGTGGGCGTAGGAAGAAGGGTTTGACGGAGA

AGATCAAAGAGAAGCTGCCAGGTGGAAACAAGACGACAGGTGTCTGTCATCCAGGGACTC

AGGGAGTACAGGGGGGTCGCGAGCATGAGAAGCCAGGTTGCGGGCAGGGTGGACAGGTGG

GCCGCGAGCATGAGAAGACAGGTTTCGGTCATCCAGGAACTCAGGGTGGACAGGTGGGCA

GCGAGCAGGAGAAGAAGGGTATGATTGAGAAGATCAAGGAGAAGCTGCCAGGTCACAAGT

AGGATGTGTGTCCACCATACACGTGGAGTCTACACCTACGTGTCTTATGTTACTTATAAT

ACGCATGGAGTGTGTCCGGAGTCTGTAATAATCTGCGCGTATCTGTTATGTC

>FJWYS212

AGCCGTTGGATCTAGTTGAAAATGTCAGAGACGCGTGATGAGTATGGCAACCAGGTTCGC

CAAACCGACGAGTATGGGAACCCGATTCAGCATACTGGCACCGGGACAAAGCCCGGTTCG

GGCATACATGGTGGGGGCCATGGGATTGGCACAGGTGGTGGTGGTGGACAAGGCAAGCTC

CACCGTCCAGGCTCCGGCTCTTCCTCTGTAAGTATATTCGATGTTTCTCAAGGGTCATTT

CACTTGGTATATAGTGTTGGGATGACTAGATTAGTTGATTAACGGTTGTGGTTGTAAATC

AGTGATATGGGCTTCTATGTACCTTATTTCACAATGATAATCAATTGATACGCTGGATCG

TGATGAGCGGACCGGATGAGCCGTCCATCTTACAGGCTTTTTAAAATAGAATTATAGGGT

GGGTGGTATGAGGTCCACCAAGCCTGTAACATGGAATTACAATCATAGCCGTTAATTGGT

TCATCTCATCCGCTCATCGTGATCCAGCATGTCAAACAGAGATGGGTTTCTGGAGAAGTG

GATGTATGTAAGCCCACAAATCACAAAAAAAATCTGACATAATGATGGAGAGAATGCAAG

GAGTGTAAGTGAATCTACGCCGTTAAATGGATTTCATTGTACTTAACTTCGTTTTGTCAA

ATATTTTGTAGGACGAGGATGATGGACAAGGTGGGCGTAGGAAGAAGGGTTTGACGGAGA

AGATCAAAGAGAAGCTGCCAGGTGGAAACAAGACGACAGGTGTCTGTCATCCAGGGACTC

AGGGAGTACAGGGGGGTCGCGAGCATGAGAAGCCAGGTTTCGGTCAGGGTGGACAGGTGG

GCCGCGAGCATGAGAAGACAGGTTTCGGTCATCCAGGGACTCAGGGTGGACAGGTGGGCA

GCGAGCAGGAGAAGAAGGGTATGATTGAGAAGATCAAGGAGAAGCTGCCAGGTCACAAGT

AGGATGTGTGTCCACTATACACGTGGAGTCTGCATCTACGTGTCTTATGTTACTTATAAT

ACGCATGGAGTGTGTCCGGAGTCTGTAATAATCTGCGCGTATCTGTTATGTC

>FJWYS43

AGCCGTTGGATCTAGTTGAAAATGTCAGAGACGCGTGATGAGTATGGCAACCAGGTTCGC

CAAACCGACGAGTATGGGAACCCGATTCAGCATACTGGCACCGGGACAAAGCCCGGTTCG

GGCATACATGGTGGGGGCCATGGGATTGGCACAGGTGGTGGTGGTGGACAAGGCAAGCTC

CACCGTCCAGGCTCCGGCTCTTCCTCTGTAAGTATATTCGATGTTTCTCAAGGGTCATTT

CACTTGGTATATAGTGTTGGGATGACTAGATTAGTTGATTAACGGTTGTGGTTGTAAATC

AGTGATATGGGCTTCTATGTACCTTATTTCACAATGATAATCAATTGATACGCTGGATCG

TGATGAGCGGACCGGATGAGCCGTCCATCTTACAGGCTTTTTAAAATAGAATTATAGGGT

GGGTGGTATGAGGTCCACCAAGCCTGTAACATGGAATTACAATCATAGCCGTTAATTGGT

TCATCTCATCCGCTCATCGTGATCCAGCATGTCAAACAGAGATGGGTTTCTGGAGAAATG

GATGTATGTAAGCCCACAAATCACAAAAAAAATCTGACATAATGATGGAGAGAATGCAAG

GAGTGTAAGTGAATCTACGCCGTTAAATGGATTTCATTGTACTTAACTTCGTTTTGTCAA

ATATTTTGTAGGACGAGGATGATGGACAAGGTGGGCGTAGGAAGAAGGGTTTGACGGAGA

AGATCAAAGAGAAGCTGCCAGGTGGAAACAAGACGACAGGTGTCTGTCATCCAGGGACTC

AGGGAGTACAGGGGGGTCGCGAGCATGAGAAGCCAGGTTGCGGGCAGGGTGGACAGGTGG

GCCGCGAGCATGAGAAGACAGGTTTCGGTCATCCAGGAACTCAGGGTGGACAGGTGGGCA

GCGAGCAGGAGAAGAAGGGTATGATTGAGAAGATCAAGGAGAAGCTGCCAGGTCACAAGT

AGGATGTGTGTCCACCATACACGTGGAGTCTACACCTACGTGTCTTATGTTACTTATAAT

ACGCATGGAGTGTGTCCGGAGTCTGTAATAATCTGCGCGTATCTGTTATGTC

>FJWYS45

AGCCGTTGGATCTAGTTGAAAATGTCAGAGACGCGTGATGAGTATGGCAACCAGGTTCGC

CAAACCGACGAGTATGGGAACCCGATTCAGCATACTGGCACCGGGACAAAGCCCGGTTCG

GGCATACATGGTGGGGGCCATGGGATTGGCACAGGTGGTGGTGGTGGACAAGGCAAGCTC

CACCGTCCAGGCTCCGGCTCTTCCTCTGTAAGTATATTCGATGTTTCTCAAGGGTCATTT

CACTTGGTATATAGTGTTGGGATGACTAGATTAGTTGATTAACGGTTGTGGTTGTAAATC

AGTGATATGGGCTTCTATGTACCTTATTTCACAATGATAATCAATTGATACGCTGGATCG

TGATGAGCGGACCGGATGAGCCGTCCATCTTACAGGCTTTTTAAAATAGAATTATAGGGT

GGGTGGTATGAGGTCCACCAAGCCTGTAACATGGAATTACAATCATAGCCGTTAATTGGT

TCATCTCATCCGCTCATCGTGATCCAGCATGTCAAACAGAGATGGGTTTCTGGAGAAGTG

GATGTATGTAAGCCCACAAATCACAAAAAAAATCTGACATAATGATGGAGAGAATGCAAG

GAGTGTAAGTGAATCTACGCCGTTAAATGGATTTCATTGTACTTAACTTCGTTTTGTCAA

ATATTTTGTAGGACGAGGATGATGGACAAGGTGGGCGTAGGAAGAAGGGTTTGACGGAGA

AGATCAAAGAGAAGCTGCCAGGTGGAAACAAGACGACAGGTGTCTGTCATCCAGGGACTC

AGGGAGTACAGGGGGGTCGCGAGCATGAGAAGCCAGGTTTCGGTCAGGGTGGACAGGTGG

GCCGCGAGCATGAGAAGACAGGTTTCGGTCATCCAGGGACTCAGGGTGGACAGGTGGGCA

GCGAGCAGGAGAAGAAGGGTATGATTGAGAAGATCAAGGAGAAGCTGCCAGGTCACAAGT

AGGATGTGTGTCCACTATACACGTGGAGTCTGCATCTACGTGTCTTATGTTACTTATAAT

ACGCATGGAGTGTGTCCGGAGTCTGTAATAATCTGCGCGTATCTGTTATGTC

>FJWYS47

AGCCGTTGGATCTAGTTGAAAATGTCAGAGACGCGTGATGAGTATGGCAACCAGGTTCGC

CAAACCGACGAGTATGGGAACCCGATTCAGCATACTGGCACCGGGACAAAGCCCGGTTCG

GGCATACATGGTGGGGGCCATGGGATTGGCACAGGTGGTGGTGGTGGACAAGGCAAGCTC

CACCGTCCAGGCTCCGGCTCTTCCTCTGTAAGTATATTCGATGTTTCTCAAGGGTCATTT

CACTTGGTATATAGTGTTGGGATGACTAGATTAGTTGATTAACGGTTGTGATTGTAAATC

AGTGATATGGGCTTCTGTGTACCTTATTTCACAATGATAATCAATTGATACGCTGGGTCG

TGATGAGCGGACCGGATGAGCCGTCCATCTTACAGGCTTTTTAAAATAGAATTATAGGGT

GGGTGGTATGAGGTCCACCAAGCCTGTAACATGGAATTACAATCATAGCCGTTAATTGGT

TCATCTCATCCGCTCATCGTGATCCAGCATGTCAAACAGAGATGGGTTTCTGGAGAAGTG

GATGTATGTAAGCCCACAAATCACAAAAAAAATCTGACATAATGATGGAGAGAATGCAAG

GAGTGTAAGTGAATCTACGCCGTTAAATGGATTTCATTGTACTTAACTTCGTTTTGTCAA

ATATTTTGTAGGACGAGGATGATGGACAAGGTGGGCGTAGGAAGAAGGGTTTGACGGAGA

AGATCAAAGAGAAGCTGCCAGGTGGAAACAAGACGACAGGTGTCTGTCATCCAGGGACTC

AGGGAGTACAGGGGGGTCGCGAGCATGAGAAGCCAGGTTTCGGTCAGGGTGGACAGGTGG

GCCGCGAGCATGAGAAGACAGGTTTCGGTCATCCAGGGACTCAGGGTGGACAGGTGGGCA

GCGAGCAGGAGAAGAAGGGTATGATTGAGAAGATCAAGGAGAAGCTGCCAGGTCACAAGT

AGGATGTGTGTCCACTATACACGTGGAGTCTGCATCTACGTGTCTTATGTTACTTATAAT

ACGCATGGAGTGTGTCCGGAGTCTGTAATAATCTGCGCGTATCTGTTATGTC

>HBXN11

AGCCGTTGGATCTAGTTGAAAATGTCAGAGACGCGTGATGAGTATGGCAACCAGGTTCGC

CAAACCGACGAGTATGGGAACCCGATTCAGCATAGTGGCACCGGGACAAAGCCCGGTTCG

GGCATACATGGTGGGGGCCATGGGATTGGCACAGGTGGTGGTGGTGGACAAGGCAAGCTC

CACCGTCCAGGCTCCGGCTCTTCCTCTGTAAGTATATTCGATGTTTCTCAAGGGTCATTT

CACTTGGTATATCGTGTTGGGATGACTAGATTAGTTGATTAACGGTTGTGATTGTAAATC

AGTGATATGGGTTTCTGTGTACCTTATTTCACAATGATAATCAATTGATACGCTGGATCG

TGATGAGCGGACCGGATGAGCCGTCCATCTTACAGGCTTTTTAAAATAGAATTATAGGGT

GGGTGGTATGAGGTCTACCAAGCCTGTAACATGGAATTACAATCATAGCCGTTAATCGGT

TCATCTCATCCGCTCATCGTGATCCAGCATGTCAAACAGAGATGGGTTTATGGAGAAATG

GATGTATGTAAGCCAACAAATCACAAAAAAAATCTGACATAATGATGGAGAGAATGCAAG

GAGTGTAAGTGAATCTACGCCGTTAAATGGATTTCATTGTACTTAATTTCAATTTCGTTT

TGTCAAATATTTTGTAGGACGAGGATGATGGACAAGGTGGGCGGAGGAAGAAGGGTTTGA

CGGAGAAGATCAAAGAGAAGCTGCCAGGTGGAAACAAGACGACAGGTGTCTGTCATCCAG

GGACTCAGGGTGTACAGGGGGGTCGCGAGCATGAGAAGCCAGGTTGCGGTCAGGGTGGAC

AGGTGGGCCGCGAGCATGAGAAGACAGGTTTCGGTCATCCAGGGACTCAGGGTGGACAGG

TGGGCAGCGAGCAGGAGAAGAAGGGTATGATTGAGAAGATCAAGGAGAAGCTGCCAGGTC

ACAAGTAGGATGTGTGTCCACCATACACGTGGAGTCTACACCTACGTGTCTTATGTTACT

TATAATACGCATGGAGTGTGTCCGGAGTCTGTAATAATCTGCGCGTATCTGTTATGTC

>HBXN15

AGCCGTTGGATCTAGTTGAAAATGTCAGAGACGCGTGATGAGTATGGCAACCAGGTTCGC

CAAACCGACGAGTATGGGAACCCGATTCAGCATAGTGGCACCGGGACAAAGCCCGGTTCG

GGCATACATGGTGGGGGCCATGGGATTGGCACAGGTGGTGGTGGTGGACAAGGCAAGCTC

CACCGTCCAGGCTCCGGCTCTTCCTCTGTAAGTATATTCGATGTTTCTCAAGGGTCATTT

CACTTGGTATATCGTGTTGGGATGACTAGATTAGTTGATTAACGGTTGTGATTGTAAATC

AGTGATATGGGTTTCTGTGTACCTTATTTCACAATGATAATCAATTGATACGCTGGATCG

TGATGAGCGGACCGGATGAGCCGTCCATCTTACAGGCTTTTTAAAATAGAATTATAGGGT

GGGTGGTATGAGGTCTACCAAGCCTGTAACATGGAATTACAATCATAGCCGTTAATCGGT

TCATCTCATCCGCTCATCGTGATCCAGCATGTCAAACAGAGATGGGTTTATGGAGAAATG

GATGTATGTAAGCCAACAAATCACAAAAAAAATCTGACATAATGATGGAGAGAATGCAAG

GAGTGTAAGTGAATCTACGCCGTTAAATGGATTTCATTGTACTTAATTTCAATTTCGTTT

TGTCAAATATTTTGTAGGACGAGGATGATGGACAAGGTGGGCGGAGGAAGAAGGGTTTGA

CGGAGAAGATCAAAGAGAAGCTGCCAGGTGGAAACAAGACGACAGGTGTCTGTCATCCAG

GGACTCAGGGTGTACAGGGGGGTCGCGAGCATGAGAAGCCAGGTTGCGGTCAGGGTGGAC

AGGTGGGCCGCGAGCATGAGAAGACAGGTTTCGGTCATCCAGGGACTCAGGGTGGACAGG

TGGGCAGCGAGCAGGAGAAGAAGGGTATGATTGAGAAGATCAAGGAGAAGCTGCCAGGTC

ACAAGTAGGATGTGTGTCCACCATACACGTGGAGTCTACACCTACGTGTCTTATGTTACT

TATAATACGCATGGAGTGTGTCCGGAGTCTGTAATAATCTGCGCGTATCTGTTATGTC

>HBXN112

AGCCGTTGGATCTAGTTGAAAATGTCAGAGACGCGTGATGAGTATGGCAACCAGGTTCGC

CAAACCGACGAGTATGGGAACCCGATTCAGCATAGTGGCACCGGGACAAAGCCCGGTTCG

GGCATACATGGTGGGGGCCATGGGATTGGCACAGGTGGTGGTGGTGGACAAGGCAAGCTC

CACCGTCCAGGCTCCGGCTCTTCCTCTGTAAGTATATTCGATGTTTCTCAAGGGTCATTT

CACTTGGTATATCGTGTTGGGATGACTAGATTAGTTGATTAACGGTTGTGATTGTAAATC

AGTGATATGGGTTTCTGTGTACCTTATTTCACAATGATAATCAATTGATACGCTGGATCG

TGATGAGCGGACCGGATGAGCCGTCCATCTTACAGGCTTTTTAAAATAGAATTATAGGGT

GGGTGGTATGAGGTCTACCAAGCCTGTAACATGGAATTACAATCATAGCCGTTAATCGGT

TCATCTCATCCGCTCATCGTGATCCAGCATGTCAAACAGAGATGGGTTTATGGAGAAATG

GATGTATGTAAGCCAACAAATCACAAAAAAAATCTGACATAATGATGGAGAGAATGCAAG

GAGTGTAAGTGAATCTACGCCGTTAAATGGATTTCATTGTACTTAATTTCAATTTCGTTT

TGTCAAATATTTTGTAGGACGAGGATGATGGACAAGGTGGGCGGAGGAAGAAGGGTTTGA

CGGAGAAGATCAAAGAGAAGCTGCCAGGTGGAAACAAGACGACAGGTGTCTGTCATCCAG

GGACTCAGGGTGTACAGGGGGGTCGCGAGCATGAGAAGCCAGGTTGCGGTCAGGGTGGAC

AGGTGGGCCGCGAGCATGAGAAGACAGGTTTCGGTCATCCAGGGACTCAGGGTGGACAGG

TGGGCAGCGAGCAGGAGAAGAAGGGTATGATTGAGAAGATCAAGGAGAAGCTGCCAGGTC

ACAAGTAGGATGTGTGTCCACCATACACGTGGAGTCTACACCTACGTGTCTTATGTTACT

TATAATACGCATGGAGTGTGTCCGGAGTCTGTAATAATCTGCGCGTATCTGTTATGTC

>HBXN116

AGCCGTTGGATCTAGTTGAAAATGTCAGAGACGCGTGATGAGTATGGCAACCAGGTTCGC

CAAACCGACGAGTATGGGAACCCGATTCAGCATAGTGGCACCGGGACAAAGCCCGGTTCG

GGCATACATGGTGGGGGCCATGGGATTGGCACAGGTGGTGGTGGTGGACAAGGCAAGCTC

CACCGTCCAGGCTCCGGCTCTTCCTCTGTAAGTATATTCGATGTTTCTCAAGGGTCATTT

CACTTGGTATATCGTGTTGGGATGACTAGATTAGTTGATTAACGGTTGTGATTGTAAATC

AGTGATATGGGTTTCTGTGTACCTTATTTCACAATGATAATCAATTGATACGCTGGATCG

TGATGAGCGGACCGGATGAGCCGTCCATCTTACAGGCTTTTTAAAATAGAATTATAGGGT

GGGTGGTATGAGGTCTACCAAGCCTGTAACATGGAATTACAATCATAGCCGTTAATCGGT

TCATCTCATCCGCTCATCGTGATCCAGCATGTCAAACAGAGATGGGTTTATGGAGAAATG

GATGTATGTAAGCCAACAAATCACAAAAAAAATCTGACATAATGATGGAGAGAATGCAAG

GAGTGTAAGTGAATCTACGCCGTTAAATGGATTTCATTGTACTTAATTTCAATTTCGTTT

TGTCAAATATTTTGTAGGACGAGGATGATGGACAAGGTGGGCGGAGGAAGAAGGGTTTGA

CGGAGAAGATCAAAGAGAAGCTGCCAGGTGGAAACAAGACGACAGGTGTCTGTCATCCAG

GGACTCAGGGTGTACAGGGGGGTCGCGAGCATGAGAAGCCAGGTTGCGGTCAGGGTGGAC

AGGTGGGCCGCGAGCATGAGAAGACAGGTTTCGGTCATCCAGGGACTCAGGGTGGACAGG

TGGGCAGCGAGCAGGAGAAGAAGGGTATGATTGAGAAGATCAAGGAGAAGCTGCCAGGTC

ACAAGTAGGATGTGTGTCCACCATACACGTGGAGTCTACACCTACGTGTCTTATGTTACT

TATAATACGCATGGAGTGTGTCCGGAGTCTGTAATAATCTGCGCGTATCTGTTATGTC

>HXN21

AGCCGTTGGATCTAGTTGAAAATGTCAGAGACGCGTGATGAGTATGGCAACCAGGTTCGC

CAAACCGACGAGTATGGGAACCCGATTCAGCATACTGGCACCGGGACAAAGGCCGGTTCG

GGCATACATGGTGGGGCCCATGGGATTGGCACAGGTGGTGGTGGTGGACAAGGCAAGCTC

CACCGTCCAGGCTCCGGCTCTTCCTCTGTAAGTATATTCGATGTTTCTCAAGGGCCATTT

CACTTGGTATGTCGTGTTGGGATGACTAGATTAGTTGATTAACGGTTGTGATTGTAAATC

AGTGATATGGGCTTCTGTGTACCTTATTTCACAATGATAATCAATTGATACGCTGGATCG

TGATGAGCGGACCGGATGAGCCGTCCATCTTACAGGCTTTTTAAAATAGAATTATAGGGT

GGGTGGTATGAGGTCTACCAAGCCTGTAACATGGAATTACAATCATAGCTGTTAATCGGT

TCATCTCATCCGCTCATCGTGATCCAGCATGTCAAACAGAGATGGGTTTATGGAGAAATG

GATGTATGTAAGCCAACAAATCACAAAAAAATCTGACATAATGATGGAGAGAATGCAAGG

AGTGTAAGTGAATCTACGCCGTTAAATGGATTTCATTGTACTTAATTTCGTTTTGTCAAA

TATTTTGTAGGACGAGGATGATGGACAAGGTGGGCGTAGGAAGAAGGGTTTGACGGAGAA

GATCAAAGAGAAGCTGCCAGGTGGAAACAAGACGACAGGTGTCTGTCATCCAGGGACTCA

GGGAGTACAGGGGGGTCGCGAGCATGAGAAGCCAGGTTGCGGTCAGGGTGGACAGGTGGG

CCGCGAGCATGAGAAGACAGGTTTCGGTCATCCAGGGACTCAGGGTGGACAGGTGGGCAG

CGAGCAGGAGAAGAAGGGTATGATTGAGAAGATCAAGGAGAAGCTGCCAGGTCACAAGTA

GGATGTGTGTCCACCATACACGTGGAGTCTACACCTACGTGTCTTATGTTACTTATAATA

CGCATGGAGTGTGTCCGGAGTCTGTAATAATCTGCGCGTATCTGTTATGTC

>HXN24

AGCCGTTGGATCTAGTTGAAAATGTCAGAGACGCGTGATGAGTATGGCAACCAGGTTCGC

CAAACCGACGAGTATGGGAACCCGATTCAGCATACTGGCACCGGGACAAAGGCCGGTTCG

GGCATACATGGTGGGGCCCATGGGATTGGCACAGGTGGTGGTGGTGGACAAGGCAAGCTC

CACCGTCCAGGCTCCGGCTCTTCCTCTGTAAGTATATTCGATGTTTCTCAAGGGCCATTT

CACTTGGTATGTCGTGTTGGGATGACTAGATTAGTTGATTAACGGTTGTGATTGTAAATC

AGTGATATGGGCTTCTGTGTACCTTATTTCACAATGATAATCAATTGATACGCTGGATCG

TGATGAGCGGACCGGATGAGCCGTCCATCTTACAGGCTTTTTAAAATAGAATTATAGGGT

GGGTGGTATGAGGTCTACCAAGCCTGTAACATGGAATTACAATCATAGCTGTTAATCGGT

TCATCTCATCCGCTCATCGTGATCCAGCATGTCAAACAGAGATGGGTTTATGGAGAAATG

GATGTATGTAAGCCAACAAATCACAAAAAAATCTGACATAATGATGGAGAGAATGCAAGG

AGTGTAAGTGAATCTACGCCGTTAAATGGATTTCATTGTACTTAATTTCGTTTTGTCAAA

TATTTTGTAGGACGAGGATGATGGACAAGGTGGGCGTAGGAAGAAGGGTTTGACGGAGAA

GATCAAAGAGAAGCTGCCAGGTGGAAACAAGACGACAGGTGTCTGTCATCCAGGGACTCA

GGGAGTACAGGGGGGTCGCGAGCATGAGAAGCCAGGTTGCGGTCAGGGTGGACAGGTGGG

CCGCGAGCATGAGAAGACAGGTTTCGGTCATCCAGGGACTCAGGGTGGACAGGTGGGCAG

CGAGCAGGAGAAGAAGGGTATGATTGAGAAGATCAAGGAGAAGCTGCCAGGTCACAAGTA

GGATGTGTGTCCACCATACACGTGGAGTCTACACCTACGTGTCTTATGTTACTTATAATA

CGCATGGAGTGTGTCCGGAGTCTGTAATAATCTGCGCGTATCTGTTATGTC

>HXN29

AGCCGTTGGATCTAGTTGAAAATGTCAGAGACGCGTGATGAGTATGGCAACCAGGTTCGC

CAAACCGACGAGTATGGGAACCCGATTCAGCATACTGGCACCGGGACAAAGCCCGGTTCG

GGCATACATGGTGGGGGCCATGGGATTGGCACAGGTGGTGGTGGTGGACAAGGCAAGCTC

CACCGTCCAGGCTCCGGCTCTTCCTCTGTAAGTATATTCGATGTTTCTCAAGGGTCATTT

CACTTGGTATATAGTGTTGGGATGACTAGATTAGTTGATTAACGGTTGTGATTGTAAATC

AGTGATATGGGCTTCTATGTACCTTATTTCACAATGATAATCAATTGATACGCTGGATCG

TGATGAGCGGACCGGATGAGCCGTCCATCTTACAGGCTTTTTAAAATAGAATTATAGGGT

GGGTGGTATGAGGTCTACCAAGCCTGTAACATGGAATTACAATCATAGCCGTTAATCCGT

TCATCTCATCCGCTCATCGTGATCCAGCATGTCAAACAGAGATGGGTTTATGGAGAAATG

GATGTATGTAAGCCAACAAATCACAAAAAAAATCTGACATAATGATGGAGAGAATGCAAG

GAGTGTAAGTGAATCTACGCCGTTAAATGGATTTCATTGTACTTAATTTCGTTTTGTCAA

ATATTTTGTAGGACGAGGATGATGGACAAGGTGGGCGTAGGAAGAAGGGTTTGACGGAGA

AGATCAAAGAGAAGCTGCCAGGTGGAAACAAGACGACAGGTGTCTGTCATCCAGGGACTC

AGGGTGTACAGGGGGGTCGCGAGCATGAGAAGCCAGGTTGCGGTCAGGGTGGACAGGTGG

GCCGCGAGCATGAGAAGACAGGTTTCGGTCATCCAGGGACTCAGGGTGGACAGGTGGGCA

GCGAGCAGGAGAAGAAGGGTATGATTGAGAAGATCAAGGAGAAGCTGCCAGGTCACAAGT

AGGATGTGTGTCCACCATACACGTGGAGTCTACACCTACGTGTCTTATGTTACTTATAAT

ACGCATGGAGTGTGTCCGGAGTCTGTAATAATCTGCGCGTATCTGTTATGTC

>HBXN31

AGCCGTTGGATCTAGTTGAAAATGTCAGAGACGCGTGATGAGTATGGCAACCAGGTTCGC

CAAACCGACGAGTATGGGAACCCGATTCAGCATACTGGCACCGGGACAAAGCCCGGTTCG

GGCATACATGGTGGGGGCCATGGGATTGGCACAGGTGGTGGTGGTGGACAAGGCAAGCTC

CACCGTCCAGGCTCCGGCTCTTCCTCTGTAAGTATATTCGATGTTTCTCAAGGGCCATTT

CACTTGGTATATCGTGTTGGGATGACTAGATTAGTTGATTAACGGTTGTGATTGTAAATC

AGTGATATGGGCTTCTGTGTACCTTATTTCACAATGATAATCAATTGATACGCTGGATCG

TGATGAGCGGACCGGATGAGCCGTCCATCTTACTGGCTTTTTAAAATAGAATTATAGGGT

GGGTGGTATGAGGTCTACCAAGCCTGTAACATGGAATTACAATCATAGCTGTTAATCGGT

TCATCTCATCCGCTCATCGTGATCCAGCATGTCCAACAGAGATGGGTTTCTGGAGAAATG

GATGTATGTAAGCCCACAAATCACAAAAAAAAATCTGACATAATGATGGAGAGAATGCAA

GGAGTGTAAGTGAATCTACGCCGTTAAATGGATTTCATTGTACTTAACTTCGTTTTGTCA

AATATTTTGTAGGACGAGGATGATGGACAAGGTGGGCGTAGGAAGAAGGGTTTGACGGAG

AAGATCAAAGAGAAGCTGCCAGGTGGAAACAAGACGACAGGTGTCTGTCATCCAGGGACT

CAGGGAGTACAGGGGGGTCGCGAGCATGAGAAGCCAGGTTGCGGTCAGGGTGGACAGGTG

GGCCGCGAGCATGAGAAGACAGGTTTCGGCCATCCAGGGACTCAGGGTGGACAGGTGGGC

AGCGAGCAGGAGAAGAAGGGTATGATTGAGAAGATCAAGGAGAAGCTGCCAGGTCACAAG

TAGGATGTGTGTCCACCATACACGTGGAGTCTACATCTACGTGTCTTATGTTACTTATAA

TACGCATGGAGTGTGTCCGGAGTCTGTAATAATCTGCGCGTATCTGTTATGTC

>HBXN320

AGCCGTTGGATCTAGTTTAAAATGTCAGAGACGCGTGATGAGTATGGCAACCAGGTTCGC

CAAACCGACGAGTATGAGAACCCGATTCAGCATACTGGCACCGGGACAAAGCCCGGTTCG

GGCATACATGGTGGGGGCCATGGGATTGGCACAGGTGGTGGTGGTGGACAAGGCAAGCTC

CACCGTCCAGGCTCCGGCTCTTCCTCTGTAAGTATATTCGATGTTTCTCAAGGGTCATTT

CACTTGGTATATCGTGTTGGGATGACTAGATTAGTTGATTAACGGTTGTGATTGTAAATC

AGTGATATGGGCTTGTGTGTACCTTATTTCACAATGATAATCAATTGATACGCTGGATCG

TGATGAGCGGACCGGATGAGCCGTCCATCTTACAGGCTTTTTAAAATAGAATTATAGGGT

GGGTGGTATGAGGTCTACCAAGCCTGTAACATGGAATTACAATCATAGCTGTTAATCGGT

TCATCTCATCCGCTCATCGTGATCCAGCATGTCCAACAGAGATGGGTTTCTGGAGAAATG

GATGTATGTAAGCCCACAAATCACAAAAAAAAATCTGACATAATGATGGAGAGAATGCAA

GGAGTGTAAGTGAATCTACGCCGTTAAATGGATTTCATTGTACTTAACTTCGTTTTGTCA

AATATTTTGTAGGACGAGGATGATGGACAAGGTGGGCGTAGGAAGAAGGGTTTGACGGAG

AAGATCAAAGAGAAGCTGCCAGGTGGAAACAAGACGACAGGTGTCTGTCATCCAGGGACT

CAGGGAGTACAGGGGGGTCGCGAGCATGAGAAGCCAGGTTGCGGTCAGGGTGGACAGGTG

GGCCGCGAGCATGAGAAGACAGGTTTCGGCCATCCAGGGACTCAGGGTGGACAGGTGGGC

AGCGAGCAGGAGAAGAAGGGTATGATTGAGAAGATCAAGGAGAAGCTGCCAGGTCACAAG

TAGGATGTGTGTCCACCATACACGTGGAGTCTACATCTACGTGTCTTATGTTACTTATAA

TACGCATGGAGTGTGTCCGGAGTCTGTAATAATCTGCGCGTATCTGTTATGTC

>HBXN329

AGCCGTTGGATCTAGTTGAAAATGTCAGAGACGCGTGATGAGTATGGCAACCAGGTTCGC

CAAACCGACGAGTATGGGAACCCGATTCAGCATACTGGCACCGGGACAAAGCCCGGTTCG

GGCATACATGGTGGGGGCCATGGGATTGGCACAGGTGGTGGTGGTGGACAAGGCAAGCTC

CACCGTCCAGGCTCCGGCTCTTCCTCTGTAAGTATATTCGATGTTTCTCAAGGGCCATTT

CACTTGGTATATCGTGTTGGGATGACTAGATTAGTTGATTAACGGTTGTGATTGTAAATC

AGTGATATGGGCTTCTGTGTACCTTATTTCACAATGATAATCAATTGATACGCTGGATCG

TGATGAGCGGACCGGATGAGCCGTCCATCTTACTGGCTTTTTAAAATAGAATTATAGGGT

GGGTGGTATGAGGTCTACCAAGCCTGTAACATGGAATTACAATCATAGCTGTTAATCGGT

TCATCTCATCCGCTCATCGTGATCCAGCATGTCCAACAGAGATGGGTTTCTGGAGAAATG

GATGTATGTAAGCCCACAAATCACAAAAAAAAATCTGACATAATGATGGAGAGAATGCAA

GGAGTGTAAGTGAATCTACGCCGTTAAATGGATTTCATTGTACTTAACTTCGTTTTGTCA

AATATTTTGTAGGACGAGGATGATGGACAAGGTGGGCGTAGGAAGAAGGGTTTGACGGAG

AAGATCAAAGAGAAGCTGCCAGGTGGAAACAAGACGACAGGTGTCTGTCATCCAGGGACT

CAGGGAGTACAGGGGGGTCGCGAGCATGAGAAGCCAGGTTGCGGTCAGGGTGGACAGGTG

GGCCGCGAGCATGAGAAGACAGGTTTCGGCCATCCAGGGACTCAGGGTGGACAGGTGGGC

AGCGAGCAGGAGAAGAAGGGTATGATTGAGAAGATCAAGGAGAAGCTGCCAGGTCACAAG

TAGGATGTGTGTCCACCATACACGTGGAGTCTACATCTACGTGTCTTATGTTACTTATAA

TACGCATGGAGTGTGTCCGGAGTCTGTAATAATCTGCGCGTATCTGTTATGTC

>HBXN62

AGCCGTTGGATCTAGTTGAAAATGTCAGAGACGCGTGATGAGTATGGCAACCAGGTTCGC

CAAACCGACGAGTATGGGAACCCGATTCAGCATACTGGCACCGGGACAAAGCCCGGTTCG

GGCATACATGGTGGGGGCCATGGGATTGGCACAGGTGGTGGTGGTGGACAAGGCAAGCTC

CACCGTCCAGGCTCCGGCTCTTCCTCTGTAAGTATATTCGATGTTTCTCAAGGGCCATTT

CACTTGGTATATCGTGTTGGGATGACTAGATTAGTTGATTAACGGTTGTGATTGTAAATC

AGTGATATGGGCTTCTGTGTACCTTATTTCACAATGATAATCAATTGATACGCTGGATCG

TGATGAGCGGACCGGATGAGCCGTCCATCTTACTGGCTTTTTAAAATAGAATTATAGGGT

GGGTGGTATGAGGTCTACCAAGCCTGTAACATGGAATTACAATCATAGCTGTTAATCGGT

TCATCTCATCCGCTCATCGTGATCCAGCATGTCCAACAGAGATGGGTTTCTGGAGAAATG

GATGTATGTAAGCCCACAAATCACAAAAAAAAATCTGACATAATGATGGAGAGAATGCAA

GGAGTGTAAGTGAATCTACGCCGTTAAATGGATTTCATTGTACTTAACTTCGTTTTGTCA

AATATTTTGTAGGACGAGGATGATGGACAAGGTGGGCGTAGGAAGAAGGGTTTGACGGAG

AAGATCAAAGAGAAGCTGCCAGGTGGAAACAAGACGACAGGTGTCTGTCATCCAGGGACT

CAGGGAGTACAGGGGGGTCGCGAGCATGAGAAGCCAGGTTGCGGTCAGGGTGGACAGGTG

GGCCGCGAGCATGAGAAGACAGGTTTCGGCCATCCAGGGACTCAGGGTGGACAGGTGGGC

AGCGAGCAGGAGAAGAAGGGTATGATTGAGAAGATCAAGGAGAAGCTGCCAGGTCACAAG

TAGGATGTGTGTCCACCATACACGTGGAGTCTACATCTACGTGTCTTATGTTACTTATAA

TACGCATGGAGTGTGTCCGGAGTCTGTAATAATCTGCGCGTATCTGTTATGTC

>HBXN69

AGCCGTTGGATCTAGTTGAAAATGTCAGAGACGCGTGATGAGTATGGCAACCAGGTTCGC

CAAACCGACGAGTATGAGAACCCGATTCAGCATACTGGCACCGGGACAAAGCCCGGTTCG

GGCATACATGGTGGGGGCCATGGGATTGGCACAGGTGGTGGTGGTGGACAAGGCAAGCTC

CACCGTCCAGGCTCCGGCTCTTCCTCTGTAAGTATATTCGATGTTTCTCAAGGGTCATTT

CACTTGGTATATCGTGTTGGGATGACTAGATTAGTTGATTAACGGTTGTGATTGTAAATC

AGTGATATGGGCTTGTGTGTACCTTATTTCACAATGATAATCAATTGATACGCTGGATCG

TGATGAGCGGACCGGATGAGCCGTCCATCTTACAGGCTTTTTAAAATAGAATTATAGGGT

GGGTGGTATGAAGTCTACCAAGCCTGTAACATGGAATTACAATCATAGCCGTTAATCGGT

TCATCTCATTCGCTCATCGTGATCCAGCATGTCAAACAGAGATGGGTTTATGGAGAAATG

GATGTATGTAAGCCAACAAATCACAAAAAAAATCTGACATAATGATGGAGAGAATGCAAG

GAGTGTAAGTGAATCTACGCCGTTAAATGGATTTCATTGTAATTAATTTCGTTTTGTCAA

ATATTTTGTAGGACGAGGATGATGGACAAGGTGGGCGTAGGAAGAAGGGTTTGACGGAGA

AGATCAAAGAGAAGCTGCCAGGTGGAAACAAGACGACAGGTGTCTGTCATCCAGGGACTC

AGGGTGTACAGGGGGGTCGCGAGCATGAGAAGCCAGGTTGCGGTCAGGGTGGACAGGTGG

GCCGCGAGCATGAGAAGACAGGTTTCGGTCATCCAGGAACTCAGGGTGGACAGGTGGGCA

GCGAGCAGGAGAAGAAGGGTATGATTGAGAAGATCAAGGAGAAGCTGCCAGGTCACAAGT

AGGATGTGTGTCCACCATACACGTGGAGTCTACATCTACGTGTCTTATGTTACTTATAAT

ACGCATGGAGTGTGTCCGGAGTCTGTAATAATCTGCGCGTATCTGTTATGTC

>HBXN616

AGCCGTTGGATCTAGTTGAAAATGTCAGAGACGCGTGATGAGTATGGCAACCAGGTTCGC

CAAACCGACGAGTATGAGAACCCGATTCAGCATACTGGCACCGGGACAAAGCCCGGTTCG

GGCATACATGGTGGGGGCCATGGGATTGGCACAGGTGGTGGTGGTGGACAAGGCAAGCTC

CACCGTCCAGGCTCCGGCTCTTCCTCTGTAAGTATATTCGATGTTTCTCAAGGGTCATTT

CACTTGGTATATCGTGTTGGGATGACTAGATTAGTTGATTAACGGTTGTGATTGTAAATC

AGTGATATGGGCTTGTGTGTACCTTATTTCACAATGATAATCAATTGATACGCTGGATCG

TGATGAGCGGACCGGATGAGCCGTCCATCTTACAGGCTTTTTAAAATAGAATTATAGGGT

GGGTGGTATGAAGTCTACCAAGCCTGTAACATGGAATTACAATCATAGCCGTTAATCGGT

TCATCTCATTCGCTCATCGTGATCCAGCATGTCAAACAGAGATGGGTTTATGGAGAAATG

GATGTATGTAAGCCAACAAATCACAAAAAAAATCTGACATAATGATGGAGAGAATGCAAG

GAGTGTAAGTGAATCTACGCCGTTAAATGGATTTCATTGTAATTAATTTCGTTTTGTCAA

ATATTTTGTAGGACGAGGATGATGGACAAGGTGGGCGTAGGAAGAAGGGTTTGACGGAGA

AGATCAAAGAGAAGCTGCCAGGTGGAAACAAGACGACAGGTGTCTGTCATCCAGGGACTC

AGGGTGTACAGGGGGGTCGCGAGCATGAGAAGCCAGGTTGCGGTCAGGGTGGACAGGTGG

GCCGCGAGCATGAGAAGACAGGTTTCGGTCATCCAGGAACTCAGGGTGGACAGGTGGGCA

GCGAGCAGGAGAAGAAGGGTATGATTGAGAAGATCAAGGAGAAGCTGCCAGGTCACAAGT

AGGATGTGTGTCCACCATACACGTGGAGTCTACATCTACGTGTCTTATGTTACTTATAAT

ACGCATGGAGTGTGTCCGGAGTCTGTAATAATCTGCGCGTATCTGTTATGTC

>HBEX818

AGCCGTTGGATCTAGTTGAAAATGTCAGAGACGCGTGATGAGTATGGCAACCAGGTTCGC

CAAACCGACGAGTATGGGAACCCGATTCAGCATACTGGCACCGGGACAAAGCCCGGTTCG

GGCATACATGGTGGGGGCCATGGGATTGGCACAGGTGGTGGTGGTGGACAAGGCAAGCTC

CACCGTCCAGGCTCCGGCTCTTCCTCTGTAAGTATATTCGATGTTTCTCAAGGGCCATTT

CACTTGGTATATCGTGTTGGGATGACTAGATTAGTTGATTAACGGTTGTGATTGTAAATC

AGTGATATGGGCTTCTGTGTACCTTATTTCACAATGATAATCAATTGATACGCTGGATCG

TGATGAGCGGACCGGATGAGCCGTCCATCTTACTGGCTTTTTAAAATAGAATTATAGGGT

GGGTGGTATGAGGTCTACCAAGCCTGTAACATGGAATTACAATCATAGCTGTTAATCGGT

TCATCTCATCCGCTCATCGTGATCCAGCATGTCCAACAGAGATGGGTTTCTGGAGAAATG

GATGTATGTAAGCCCACAAATCACAAAAAAAAATCTGACATAATGATGGAGAGAATGCAA

GGAGTGTAAGTGAATCTACGCCGTTAAATGGATTTCATTGTACTTAACTTCGTTTTGTCA

AATATTTTGTAGGACGAGGATGATGGACAAGGTGGGCGTAGGAAGAAGGGTTTGACGGAG

AAGATCAAAGAGAAGCTGCCAGGTGGAAACAAGACGACAGGTGTCTGTCATCCAGGGACT

CAGGGAGTACAGGGGGGTCGCGAGCATGAGAAGCCAGGTTGCGGTCAGGGTGGACAGGTG

GGCCGCGAGCATGAGAAGACAGGTTTCGGCCATCCAGGGACTCAGGGTGGACAGGTGGGC

AGCGAGCAGGAGAAGAAGGGTATGATTGAGAAGATCAAGGAGAAGCTGCCAGGTCACAAG

TAGGATGTGTGTCCACCATACACGTGGAGTCTACATCTACGTGTCTTATGTTACTTATAA

TACGCATGGAGTGTGTCCGGAGTCTGTAATAATCTGCGCGTATCTGTTATGTC

>HBEX83

AGCCGTTGGATCTAGTTGAAAATGTCAGAGACGCGTGATGAGTATGGCAACCAGGTTCGC

CAAACCGACGAGTATGGGAACCCGATTCAGCATACTGGCACCGGGACAAAGCCCGGTTCG

GGCATACATGGTGGGGGCCATGGGATTGGCACAGGTGGTGGTGGTGGACAAGGCAAGCTC

CACCGTCCAGGCTCCGGCTCTTCCTCTGTAAGTATATTCGATGTTTCTCAAGGGCCATTT

CACTTGGTATATCGTGTTGGGATGACTAGATTAGTTGATTAACGGTTGTGATTGTAAATC

AGTGATATGGGCTTCTGTGTACCTTATTTCACAATGATAATCAATTGATACGCTGGATCG

TGATGAGCGGACCGGATGAGCCGTCCATCTTACTGGCTTTTTAAAATAGAATTATAGGGT

GGGTGGTATGAGGTCTACCAAGCCTGTAACATGGAATTACAATCATAGCTGTTAATCGGT

TCATCTCATCCGCTCATCGTGATCCAGCATGTCCAACAGAGATGGGTTTCTGGAGAAATG

GATGTATGTAAGCCCACAAATCACAAAAAAAAATCTGACATAATGATGGAGAGAATGCAA

GGAGTGTAAGTGAATCTACGCCGTTAAATGGATTTCATTGTACTTAACTTCGTTTTGTCA

AATATTTTGTAGGACGAGGATGATGGACAAGGTGGGCGTAGGAAGAAGGGTTTGACGGAG

AAGATCAAAGAGAAGCTGCCAGGTGGAAACAAGACGACAGGTGTCTGTCATCCAGGGACT

CAGGGAGTACAGGGGGGTCGCGAGCATGAGAAGCCAGGTTGCGGTCAGGGTGGACAGGTG

GGCCGCGAGCATGAGAAGACAGGTTTCGGCCATCCAGGGACTCAGGGTGGACAGGTGGGC

AGCGAGCAGGAGAAGAAGGGTATGATTGAGAAGATCAAGGAGAAGCTGCCAGGTCACAAG

TAGGATGTGTGTCCACCATACACGTGGAGTCTACATCTACGTGTCTTATGTTACTTATAA

TACGCATGGAGTGTGTCCGGAGTCTGTAATAATCTGCGCGTATCTGTTATGTC

>HBEX84

AGCCGTTGGATCTAGTTGAAAATGTCAGAGACGCGTGATGAGTATGGCAACCAGGTTCGC

CAAACCGACGAGTATGGGAACCCGATTCAGCATACTGGCACCGGGACAAAGCCCGGTTCG

GGCATACATGGTGGGGGCCATGGGATTGGCACAGGTGGTGGTGGTGGACAAGGCAAGCTC

CACCGTCCAGGCTCCGGCTCTTCCTCTGTAAGTATATTCGATGTTTCTCAAGGGCCATTT

CACTTGGTATATCGTGTTGGGATGACTAGATTAGTTGATTAACGGTTGTGATTGTAAATC

AGTGATATGGGCTTCTGTGTACCTTATTTCACAATGATAATCAATTGATACGCTGGATCG

TGATGAGCGGACCGGATGAGCCGTCCATCTTACTGGCTTTTTAAAATAGAATTATAGGGT

GGGTGGTATGAGGTCTACCAAGCCTGTAACATGGAATTACAATCATAGCTGTTAATCGGT

TCATCTCATCCGCTCATCGTGATCCAGCATGTCCAACAGAGATGGGTTTCTGGAGAAATG

GATGTATGTAAGCCCACAAATCACAAAAAAAAATCTGACATAATGATGGAGAGAATGCAA

GGAGTGTAAGTGAATCTACGCCGTTAAATGGATTTCATTGTACTTAACTTCGTTTTGTCA

AATATTTTGTAGGACGAGGATGATGGACAAGGTGGGCGTAGGAAGAAGGGTTTGACGGAG

AAGATCAAAGAGAAGCTGCCAGGTGGAAACAAGACGACAGGTGTCTGTCATCCAGGGACT

CAGGGAGTACAGGGGGGTCGCGAGCATGAGAAGCCAGGTTGCGGTCAGGGTGGACAGGTG

GGCCGCGAGCATGAGAAGACAGGTTTCGGCCATCCAGGGACTCAGGGTGGACAGGTGGGC

AGCGAGCAGGAGAAGAAGGGTATGATTGAGAAGATCAAGGAGAAGCTGCCAGGTCACAAG

TAGGATGTGTGTCCACCATACACGTGGAGTCTACATCTACGTGTCTTATGTTACTTATAA

TACGCATGGAGTGTGTCCGGAGTCTGTAATAATCTGCGCGTATCTGTTATGTC

>HBEX12

AGCCGTTGGATCTAGTTGAAAATGTCAGAGACGCGTGATGAGTATGGCAACCAGGTTCGC

CAAACCGACGAGTATGGGAACCCGATTCAGCATACTGGCACCGGGACAAAGGCCGGTTCG

GGCATACATGGTGGGGCCCATGGGATTGGCACAGGTGGTGGTGGTGGACAAGGCAAGCTC

CACCGTCCAGGCTCCGGCTCTTCCTCTGTAAGTATATTCGATGTTTCTCAAGGGTCATTT

CACTTGGTATATAGTGTTGGGATGACTAGATTAGTTGATTAACGGTTGTATTGTAAATCA

GTGATATGGGCTTCTATGTACCTTATTTCACAATGATAATCAATTGATACGCTGGATCGT

GATGAGCGGACCGGATGAGCCGTCCATCTTACAGGCTTTTTAAAATAGAATTATAGGGTG

GGTGGTATGAGGTCCACCAAAGCCTGTAACATGGAATTACAATCATAACCGTTAATCGGT

TCATCTCATCCGCTCATCGTGATCCAGCATGTCAAACAGAGATGGGTTTATGGAGAAATG

GATGTATGTAAGCGCCAACAAATCACAAAAAAATCTGACATAATGATGGAGAGAATGCAA

GGAGTGTAAGTGAATCTACGCCGTTAAATGGATTTCATTGTAATTAATTTCGTTTTGTCA

AATATTTTGTAGGACGAGGATGATGGACAAGGTGGGCGTAGGAAGAAGGGTTTGACGGAG

AAGATCAAAGAGAAGCTGCCAGGTGGAAACAAGACGACAGGTGTCTGTCATCCAGGGACT

CAGGGTGTACAGGGGGGTCGCGAGCATGAGAAGCCAGGTTGCGGTCAGGGTGGACAGGTG

GGCCGCGAGCATGAGAAGACAGGTTTCGGTCATCCAGGAACTCAGGGTGGACAGGTGGGC

AGCGAGCAGGAGAAGAAGGGTATGATTGAGAAGATCAAGGAGAAGCTGCCAGGTCACAAG

TAGGATGTGTGTCCACCATACACGTGGAGTCTACATCTACGTGTCTTATGTTACTTATAA

TACGCATGGAGTGTGTCCGGAGTCTGTAATAATCTGCGCGTATCTGTTATGTC

>HBEX14

AGCCGTTGGATCTAGTTGAAAATGTCAGAGACGCGTGATGAGTATGGCAACCAGGTTCGC

CAAACCGACGAGTATGGGAACCCGATTCAGCATACTGGCACCGGGACAAAGGCCGGTTCG

GGCATACATGGTGGGGCCCATGGGATTGGCACAGGTGGTGGTGGTGGACAAGGCAAGCTC

CACCGTCCAGGCTCCGGCTCTTCCTCTGTAAGTATATTCGATGTTTCTCAAGGGTCATTT

CACTTGGTATATAGTGTTGGGATGACTAGATTAGTTGATTAACGGTTGTATTGTAAATCA

GTGATATGGGCTTCTATGTACCTTATTTCACAATGATAATCAATTGATACGCTGGATCGT

GATGAGCGGACCGGATGAGCCGTCCATCTTACAGGCTTTTTAAAATAGAATTATAGGGTG

GGTGGTATGAGGTCCACCAAAGCCTGTAACATGGAATTACAATCATAACCGTTAATCGGT

TCATCTCATCCGCTCATCGTGATCCAGCATGTCAAACAGAGATGGGTTTATGGAGAAATG

GATGTATGTAAGCGCCAACAAATCACAAAAAAATCTGACATAATGATGGAGAGAATGCAA

GGAGTGTAAGTGAATCTACGCCGTTAAATGGATTTCATTGTAATTAATTTCGTTTTGTCA

AATATTTTGTAGGACGAGGATGATGGACAAGGTGGGCGTAGGAAGAAGGGTTTGACGGAG

AAGATCAAAGAGAAGCTGCCAGGTGGAAACAAGACGACAGGTGTCTGTCATCCAGGGACT

CAGGGTGTACAGGGGGGTCGCGAGCATGAGAAGCCAGGTTGCGGTCAGGGTGGACAGGTG

GGCCGCGAGCATGAGAAGACAGGTTTCGGTCATCCAGGAACTCAGGGTGGACAGGTGGGC

AGCGAGCAGGAGAAGAAGGGTATGATTGAGAAGATCAAGGAGAAGCTGCCAGGTCACAAG

TAGGATGTGTGTCCACCATACACGTGGAGTCTACATCTACGTGTCTTATGTTACTTATAA

TACGCATGGAGTGTGTCCGGAGTCTGTAATAATCTGCGCGTATCTGTTATGTC

>HBEX15

AGCCGTTGGATCTAGTTGAAAATGTCAGAGACGCGTGATGAGTATGGCAACCAGGTTCGC

CAAACCGACGAGTATGGGAACCCGATTCAGCATACTGGCACCGGGACAAAGCCCGGTTCG

GGCATACATGGTGGGGGCCATGGGATTGGCACAGGTGGTGGTGGTGGACAAGGCAAGCTC

CACCGTCCAGGCTCCGGCTCTTCCTCTGTAAGTATATTCGATGTTTCTCAAGGGTCATTT

CACTTGGTATATAGTGTTGGGATGACTAGATTAGTTGATTAACGGTTGTGATTGTAAATC

AGTGATATGGGCTTCTATGTACCTTATTTCACAATGATAATCAATTGATACGCTGGATCG

TGATGAGCGGACCGGATGAGCCGTCCATCTTACAGGCTTTTTAAAATAGAATTATAGGGT

GGGTGGTATGAGGTCTACCAAGCCTGTAACATGGAATTACAATCATAGCCGTTAATCCGT

TCATCTCATCCGCTCATCGTGATCCAGCATGTCAAACAGAGATGGGTTTATGGAGAAATG

GATGTATGTAAGCCAACAAATCACAAAAAAAATCTGACATAATGATGGAGAGAATGCAAG

GAGTGTAAGTGAATCTACGCCGTTAAATGGATTTCATTGTACTTAATTTCGTTTTGTCAA

ATATTTTGTAGGACGAGGATGATGGACAAGGTGGGCGTAGGAAGAAGGGTTTGACGGAGA

AGATCAAAGAGAAGCTGCCAGGTGGAAACAAGACGACAGGTGTCTGTCATCCAGGGACTC

AGGGTGTACAGGGGGGTCGCGAGCATGAGAAGCCAGGTTGCGGTCAGGGTGGACAGGTGG

GCCGCGAGCATGAGAAGACAGGTTTCGGTCATCCAGGGACTCAGGGTGGACAGGTGGGCA

GCGAGCAGGAGAAGAAGGGTATGATTGAGAAGATCAAGGAGAAGCTGCCAGGTCACAAGT

AGGATGTGTGTCCACCATACACGTGGAGTCTACACCTACGTGTCTTATGTTACTTATAAT

ACGCATGGAGTGTGTCCGGAGTCTGTAATAATCTGCGCGTATCTGTTATGTC

>HBEX22

AGCCGTTGGATCTAGTTGAAAATGTCAGAGACGCGTGATGAGTATGGCAACCAGGTTCGC

CAAACCGACGAGTATGGGAACCCGATTCAGCATACTGGCACCGGGACAAAGCCCGGTTCG

GGCATACATGGTGGGGGCCATGGGATTGGTACAGGTGGTGGTGGTGGACAAGGCAAGCTC

CACCGTCCAGGCTCCGGCTCTTCCTCTGTAAGTATATTCGGTGTTTCTCAAGGGTCATTT

CACTTGGTATATAGTGTCGGGATGACTAGATTAGTTGATTAACGGTTGTGATTGTAAATC

AGTGATATGGGCTTCTATGTACCTTATTTCACAATGATAATCAATTGATACGCTGGATCG

TGATGAGCGGACCGGATGAGCCGTCCATCTTACAGGCTTTTTAAAATAGAATTATAGGGT

GGGTGGTATGAGGTCTACCAAGCCTGTAACATGGAATTACAATCATAGCCGTTAATCCGT

TCATCTCATCCGCTCATCGTGATCCAGCATGTCAAACAGAGATGGGTTTATGGAGAAATG

GATGTATGTAAGCCAACAAATCACAAAAAAAATCTGACATAATGATGGAGAGAATGCAAG

GAGTGTAAGTGAATCTACGCCGTTAAATGGATTTCATTGTACTTAATTTCGTTTTGTCAA

ATATTTTGTAGGACGAGGATGATGGACAAGGTGGGCGTAGGAAGAAGGGTTTGACGGAGA

AGATCAAAGAGAAGCTGCCAGGTGGAAACAAGACGACAGGTGTCTGTCATCCAGGGACTC

AGGGTGTACAGGGGGGTCGCGAGCATGAGAAGCCAGGTTGCGGTCAGGGTGGACAGGTGG

GCCGCGAGCATGAGAAGACAGGTTTCGGTCATCCAGGGACTCAGGGTGGACAGGTGGGCA

GCGAGCAGGAGAAGAAGGGTATGATTGAGAAGATCAAGGAGAAGCTACCAGGTCACAAGT

AGGATGTGTGTCCACCATACACGTGGAGTCTACACCTACGTGTCTTATGTTACTTATAAT

ACGCATGGAGTGTGTCCGGAGTCTGTAATAATCTGCGCGTATCTGTTATGTC

>HBEX24

AGCCGTTGGATCTAGTTGAAAATGTCAGAGACGCGTGATGAGTATGGCAACCAGGTTCGC

CAAACCGACGAGTATGGGAACCCGATTCAGCATACTGGCACCGGGACAAAGCCCGGTTCG

GGCATACATGGTGGGGGCCATGGGATTGGTACAGGTGGTGGTGGTGGACAAGGCAAGCTC

CACCGTCCAGGCTCCGGCTCTTCCTCTGTAAGTATATTCGGTGTTTCTCAAGGGTCATTT

CACTTGGTATATAGTGTCGGGATGACTAGATTAGTTGATTAACGGTTGTGATTGTAAATC

AGTGATATGGGCTTCTATGTACCTTATTTCACAATGATAATCAATTGATACGCTGGATCG

TGATGAGCGGACCGGATGAGCCGTCCATCTTACAGGCTTTTTAAAATAGAATTATAGGGT

GGGTGGTATGAGGTCTACCAAGCCTGTAACATGGAATTACAATCATAGCCGTTAATCCGT

TCATCTCATCCGCTCATCGTGATCCAGCATGTCAAACAGAGATGGGTTTATGGAGAAATG

GATGTATGTAAGCCAACAAATCACAAAAAAAATCTGACATAATGATGGAGAGAATGCAAG

GAGTGTAAGTGAATCTACGCCGTTAAATGGATTTCATTGTACTTAATTTCATTTTGTCAA

ATATTTTGTAGGACGAGGATGATGGACAAGGTGGGCGTAGGAAGAAGGGTTTGACGGAGA

AGATCAAAGAGAAGCTGCCAGGTGGAAACAAGACGACAGGTGTCTGTCATCCAGGGACTC

AGGGTGTACAGGGGGGTCGCGAGCATGAGAAGCCAGGTTGCGGTCAGGGTGGACAGGTGG

GCCGCGAGCATGAGAAGACAGGTTTCGGTCATCCAGGGACTCAGGGTGGACAGGTGGGCA

GCGAGCAGGAGAAGAAGGGTATGATTGAGAAGATCAAGGAGAAGCTGCCAGGTCACAAGT

AGGATGTGTGTCCACCATACACGTGGAGTCTACACCTACGTGTCTTATGTTACTTATAAT

ACGCATGGAGTGTGTCCGGAGTCTGTAATAATCTGCGCGTATCTGTTATGTC

>HBEX27

AGCCGTTGGATCTAGTTGAAAATGTCAGAGACGCGTGATGAGTATGGCAACCAGGTTCGC

CAAACCGACGAGTATGGGAACCCGATTCAGCATACTGGCACCGGGACAAAGGCCGGTTCG

GGCATACATGGTGGGGCCCATGGGATTGGCACAGGTGGTGGTGGTGGACAAGGCAAGCTC

CACCGTCCAGGCTCCGGCTCTTCCTCTGTAAGTATATTCGATGTTTCTCAAGGGTCATTT

CACTTGGTATATAGTGTTGGGATGACTAGATTAGTTGATTAACGGTTGTATTGTAAATCA

GTGATATGGGCTTCTATGTACCTTATTTCACAATGATAATCAATTGATACGCTGGATCGT

GATGAGCGGACCGGATGAGCCGTCCATCTTACAGGCTTTTTAAAATAGAATTATAGGGTG

GGTGGTATGAGGTCCACCAAAGCCTGTAACATGGAATTACAATCATAACCGTTAATCGGT

TCATCTCATCCGCTCATCGTGATCCAGCATGTCAAACAGAGATGGGTTTATGGAGAAATG

GATGTATGTAAGCGCCAACAAATCACAAAAAAATCTGACATAATGATGGAGAGAATGCAA

GGAGTGTAAGTGAATCTACGCCGTTAAATGGATTTCATTGTAATTAATTTCGTTTTGTCA

AATATTTTGTAGGACGAGGATGATGGACAAGGTGGGCGTAGGAAGAAGGGTTTGACGGAG

AAGATCAAAGAGAAGCTGCCAGGTGGAAACAAGACGACAGGTGTCTGTCATCCAGGGACT

CAGGGTGTACAGGGGGGTCGCGAGCATGAGAAGCCAGGTTGCGGTCAGGGTGGACAGGTG

GGCCGCGAGCATGAGAAGACAGGTTTCGGTCATCCAGGAACTCAGGGTGGACAGGTGGGC

AGCGAGCAGGAGAAGAAGGGTATGATTGAGAAGATCAAGGAGAAGCTGCCAGGTCACAAG

TAGGATGTGTGTCCACCATACACGTGGAGTCTACATCTACGTGTCTTATGTTACTTATAA

TACGCATGGAGTGTGTCCGGAGTCTGTAATAATCTGCGCGTATCTGTTATGTC

>HBEX66

AGCCGTTGGATCTAGTTGAAAATGTCAGAGACGCGTGATGAGTATGGCAACCAGGTTCGC

CAAACCGACGAGTATGGGAACCCGATTCAGCATACTGGCACCGGGACAAAGGCCGGTTCG

GGCATACATGGTGGGGCCCATGGGATTGGCACAGGTGGTGGTGGTGGACAAGGCAAGCTC

CACCGTCCAGGCTCCGGCTCTTCCTCTGTAAGTATATTCGATGTTTCTCAAGGGTCATTT

CACTTGGTATATAGTGTTGGGATGACTAGATTAGTTGATTAACGGTTGTATTGTAAATCA

GTGATATGGGCTTCTATGTACCTTATTTCACAATGATAATCAATTGATACGCTGGATCGT

GATGAGCGGACCGGATGAGCCGTCCATCTTACAGGCTTTTTAAAATAGAATTATAGGGTG

GGTGGTATGAGGTCCACCAAAGCCTGTAACATGGAATTACAATCATAACCGTTAATCGGT

TCATCTCATCCGCTCATCGTGATCCAGCATGTCAAACAGAGATGGGTTTATGGAGAAATG

GATGTATGTAAGCGCCAACAAATCACAAAAAAATCTGACATAATGATGGAGAGAATGCAA

GGAGTGTAAGTGAATCTACGCCGTTAAATGGATTTCATTGTAATTAATTTCGTTTTGTCA

AATATTTTGTAGGACGAGGATGATGGACAAGGTGGGCGTAGGAAGAAGGGTTTGACGGAG

AAGATCAAAGAGAAGCTGCCAGGTGGAAACAAGACGACAGGTGTCTGTCATCCAGGGACT

CAGGGTGTACAGGGGGGTCGCGAGCATGAGAAGCCAGGTTGCGGTCAGGGTGGACAGGTG

GGCCGCGAGCATGAGAAGACAGGTTTCGGTCATCCAGGAACTCAGGGTGGACAGGTGGGC

AGCGAGCAGGAGAAGAAGGGTATGATTGAGAAGATCAAGGAGAAGCTGCCAGGTCACAAG

TAGGATGTGTGTCCACCATACACGTGGAGTCTACATCTACGTGTCTTATGTTACTTATAA

TACGCATGGAGTGTGTCCGGAGTCTGTAATAATCTGCGCGTATCTGTTATGTC

>HBEX69

AGCCGTTGGATCTAGTTGAAAATGTCAGAGACGCGTGATGAGTATGGCAACCAGGTTCGC

CAAACCGACGAGTATGGGAACCCGATTCAGCATACTGGCACCGGGACAAAGGCCGGTTCG

GGCATACATGGTGGGGCCCATGGGATTGGCACAGGTGGTGGTGGTGGACAAGGCAAGCTC

CACCGTCCAGGCTCCGGCTCTTCCTCTGTAAGTATATTCGATGTTTCTCAAGGGTCATTT

CACTTGGTATATAGTGTTGGGATGACTAGATTAGTTGATTAACGGTTGTATTGTAAATCA

GTGATATGGGCTTCTATGTACCTTATTTCACAATGATAATCAATTGATACGCTGGATCGT

GATGAGCGGACCGGATGAGCCGTCCATCTTACAGGCTTTTTAAAATAGAATTATAGGGTG

GGTGGTATGAGGTCCACCAAAGCCTGTAACATGGAATTACAATCATAACCGTTAATCGGT

TCATCTCATCCGCTCATCGTGATCCAGCATGTCAAACAGAGATGGGTTTATGGAGAAATG

GATGTATGTAAGCGCCAACAAATCACAAAAAAATCTGACATAATGATGGAGAGAATGCAA

GGAGTGTAAGTGAATCTACGCCGTTAAATGGATTTCATTGTAATTAATTTCGTTTTGTCA

AATATTTTGTAGGACGAGGATGATGGACAAGGTGGGCGTAGGAAGAAGGGTTTGACGGAG

AAGATCAAAGAGAAGCTGCCAGGTGGAAACAAGACGACAGGTGTCTGTCATCCAGGGACT

CAGGGTGTACAGGGGGGTCGCGAGCATGAGAAGCCAGGTTGCGGTCAGGGTGGACAGGTG

GGCCGCGAGCATGAGAAGACAGGTTTCGGTCATCCAGGAACTCAGGGTGGACAGGTGGGC

AGCGAGCAGGAGAAGAAGGGTATGATTGAGAAGATCAAGGAGAAGCTGCCAGGTCACAAG

TAGGATGTGTGTCCACCATACACGTGGAGTCTACATCTACGTGTCTTATGTTACTTATAA

TACGCATGGAGTGTGTCCGGAGTCTGTAATAATCTGCGCGTATCTGTTATGTC

>HBEX610

AGCCGTTGGATCTAGTTGAAAATGTCAGAGACGCGTGATGAGTATGGCAACCAGGTTCGC

CAAACCGACGAGTATGGGAACCCGATTCAGCATACTGGCACCGGGACAAAGGCCGGTTCG

GGCATACATGGTGGGGCCCATGGGATTGGCACAGGTGGTGGTGGTGGACAAGGCAAGCTC

CACCGTCCAGGCTCCGGCTCTTCCTCTGTAAGTATATTCGATGTTTCTCAAGGGTCATTT

CACTTGGTATATAGTGTTGGGATGACTAGATTAGTTGATTAACGGTTGTATTGTAAATCA

GTGATATGGGCTTCTATGTACCTTATTTCACAATGATAATCAATTGATACGCTGGATCGT

GATGAGCGGACCGGATGAGCCGTCCATCTTACAGGCTTTTTAAAATAGAATTATAGGGTG

GGTGGTATGAGGTCCACCAAAGCCTGTAACATGGAATTACAATCATAACCGTTAATCGGT

TCATCTCATCCGCTCATCGTGATCCAGCATGTCAAACAGAGATGGGTTTATGGAGAAATG

GATGTATGTAAGCGCCAACAAATCACAAAAAAATCTGACATAATGATGGAGAGAATGCAA

GGAGTGTAAGTGAATCTACGCCGTTAAATGGATTTCATTGTAATTAATTTCGTTTTGTCA

AATATTTTGTAGGACGAGGATGATGGACAAGGTGGGCGTAGGAAGAAGGGTTTGACGGAG

AAGATCAAAGAGAAGCTGCCAGGTGGAAACAAGACGACAGGTGTCTGTCATCCAGGGACT

CAGGGTGTACAGGGGGGTCGCGAGCATGAGAAGCCAGGTTGCGGTCAGGGTGGACAGGTG

GGCCGCGAGCATGAGAAGACAGGTTTCGGTCATCCAGGAACTCAGGGTGGACAGGTGGGC

AGCGAGCAGGAGAAGAAGGGTATGATTGAGAAGATCAAGGAGAAGCTGCCAGGTCACAAG

TAGGATGTGTGTCCACCATACACGTGGAGTCTACATCTACGTGTCTTATGTTACTTATAA

TACGCATGGAGTGTGTCCGGAGTCTGTAATAATCTGCGCGTATCTGTTATGTC

>HNSN11

AGCCGTTGGATCTAGTTGAAAATGTCAGAGACGCGTGATGAGTATGGCAACCAGGTTCGC

CAAACCGACGAGTATGGGAACCCGATTCAGCATAGTGGCACCGGGACAAAGCCCGGTTCG

GGCATACATGGTGGGGGCCATGGGATTGGCACAGGTGGTGGTGGTGGACAAGGCAAGCTC

CACCGTCCAGGCTCCGGCTCTTCCTCTGTAAGTATATTCGATGTTTCTCAAGGGTCATTT

CACTTGGTATATCGTGTTGGGATGACTAGATTAGTTGATTAACGGTTGTGATTGTAAATC

AGTGATATGGGTTTCTGTGTACCTTATTTCACAATGATAATCAATTGATACGCTGGATCG

TGATGAGCGGACCGGATGAGCCGTCCATCTTACAGGCTTTTTAAAATAGAATTATAGGGT

GGGTGGTATGAGGTCTACCAAGCCTGTAACATGGAATTACAATCATAGCCGTTAATCGGT

TCATCTCATCCGCTCATCGTGATCCAGCATGTCAAACAGAGATGGGTTTATGGAGAAATG

GATGTATGTAAGCCAACAAATCACAAAAAAAATCTGACATAATGATGGAGAGAATGCAAG

GAGTGTAAGTGAATCTACGCCGTTAAATGGATTTCATTGTACTTAATTTCAATTTCGTTT

TGTCAAATATTTTGTAGGACGAGGATGATGGACAAGGTGGGCGGAGGAAGAAGGGTTTGA

CGGAGAAGATCAAAGAGAAGCTGCCAGGTGGAAACAAGACGACAGGTGTCTGTCATCCAG

GGACTCAGGGTGTACAGGGGGGTCGCGAGCATGAGAAGCCAGGTTGCGGTCAGGGTGGAC

AGGTGGGCCGCGAGCATGAGAAGACAGGTTTCGGTCATCCAGGGACTCAGGGTGGACAGG

TGGGCAGCGAGCAGGAGAAGAAGGGTATGATTGAGAAGATCAAGGAGAAGCTGCCAGGTC

ACAAGTAGGATGTGTGTCCACCATACACGTGGAGTCTACACCTACGTGTCTTATGTTACT

TATAATACGCATGGAGTGTGTCCGGAGTCTGTAATAATCTGCGCGTATCTGTTATGTC

>HNSN14

AGCCGTTGGATCTAGTTGAAAATGTCAGAGACGCGTGATGAGTATGGCAACCAGGTTCGC

CAAACCGACGAGTATGGGAACCCGATTCAGCATAGTGGCACCGGGACAAAGCCCGGTTCG

GGCATACATGGTGGGGGCCATGGGATTGGCACAGGTGGTGGTGGTGGACAAGGCAAGCTC

CACCGTCCAGGCTCCGGCTCTTCCTCTGTAAGTATATTCGATGTTTCTCAAGGGTCATTT

CACTTGGTATATCGTGTTGGGATGACTAGATTAGTTGATTAACGGTTGTGATTGTAAATC

AGTGATATGGGTTTCTGTGTACCTTATTTCACAATGATAATCAATTGATACGCTGGATCG

TGATGAGCGGACCGGATGAGCCGTCCATCTTACAGGCTTTTTAAAATAGAATTATAGGGT

GGGTGGTATGAGGTCTACCAAGCCTGTAACATGGAATTACAATCATAGCCGTTAATCGGT

TCATCTCATCCGCTCATCGTGATCCAGCATGTCAAACAGAGATGGGTTTATGGAGAAATG

GATGTATGTAAGCCAACAAATCACAAAAAAAATCTGACATAATGATGGAGAGAATGCAAG

GAGTGTAAGTGAATCTACGCCGTTAAATGGATTTCATTGTACTTAATTTCAATTTCGTTT

TGTCAAATATTTTGTAGGACGAGGATGATGGACAAGGTGGGCGGAGGAAGAAGGGTTTGA

CGGAGAAGATCAAAGAGAAGCTGCCAGGTGGAAACAAGACGACAGGTGTCTGTCATCCAG

GGACTCAGGGTGTACAGGGGGGTCGCGAGCATGAGAAGCCAGGTTGCGGTCAGGGTGGAC

AGGTGGGCCGCGAGCATGAGAAGACAGGTTTCGGTCATCCAGGGACTCAGGGTGGACAGG

TGGGCAGCGAGCAGGAGAAGAAGGGTATGATTGAGAAGATCAAGGAGAAGCTGCCAGGTC

ACAAGTAGGATGTGTGTCCACCATACACGTGGAGTCTACACCTACGTGTCTTATGTTACT

TATAATACGCATGGAGTGTGTCCGGAGTCTGTAATAATCTGCGCGTATCTGTTATGTC

>HNSN16

AGCCGTTGGATCTAGTTGAAAATGTCAGAGACGCGTGATGAGTATGGCAACCAGGTTCGC

CAAACCGACGAGTATGGGAACCCGATTCAGCATAGTGGCACCGGGACAAAGCCCGGTTCG

GGCATACATGGTGGGGGCCATGGGATTGGCACAGGTGGTGGTGGTGGACAAGGCAAGCTC

CACCGTCCAGGCTCCGGCTCTTCCTCTGTAAGTATATTCGATGTTTCTCAAGGGTCATTT

CACTTGGTATATCGTGTTGGGATGACTAGATTAGTTGATTAACGGTTGTGATTGTAAATC

AGTGATATGGGTTTCTGTGTACCTTATTTCACAATGATAATCAATTGATACGCTGGATCG

TGATGAGCGGACCGGATGAGCCGTCCATCTTACAGGCTTTTTAAAATAGAATTATAGGGT

GGGTGGTATGAGGTCTACCAAGCCTGTAACATGGAATTACAATCATAGCCGTTAATCGGT

TCATCTCATCCGCTCATCGTGATCCAGCATGTCAAACAGAGATGGGTTTATGGAGAAATG

GATGTATGTAAGCCAACAAATCACAAAAAAAATCTGACATAATGATGGAGAGAATGCAAG

GAGTGTAAGTGAATCTACGCCGTTAAATGGATTTCATTGTACTTAATTTCAATTTCGTTT

TGTCAAATATTTTGTAGGACGAGGATGATGGACAAGGTGGGCGGAGGAAGAAGGGTTTGA

CGGAGAAGATCAAAGAGAAGCTGCCAGGTGGAAACAAGACGACAGGTGTCTGTCATCCAG

GGACTCAGGGTGTACAGGGGGGTCGCGAGCATGAGAAGCCAGGTTGCGGTCAGGGTGGAC

AGGTGGGCCGCGAGCATGAGAAGACAGGTTTCGGTCATCCAGGGACTCAGGGTGGACAGG

TGGGCAGCGAGCAGGAGAAGAAGGGTATGATTGAGAAGATCAAGGAGAAGCTGCCAGGTC

ACAAGTAGGATGTGTGTCCACCATACACGTGGAGTCTACACCTACGTGTCTTATGTTACT

TATAATACGCATGGAGTGTGTCCGGAGTCTGTAATAATCTGCGCGTATCTGTTATGTC

>HNSN41

AGCCGTTGGATCTAGTTGAAAATGTCAGAGACGCGTGATGAGTATGGCAACCAGGTTCGC

CAAACCGACGAGTATGGGAACCCGATTCAGCATAGTGGCACCGGGACAAAGCCCGGTTCG

GGCATACATGGTGGGGGCCATGGGATTGGCACAGGTGGTGGTGGTGGACAAGGCAAGCTC

CACCGTCCAGGCTCCGGCTCTTCCTCTGTAAGTATATTCGATGTTTCTCAAGGGACATTT

CACTTGGTATATCGTGTTGGGATGACTACATTAGTTGATTAACGGTTGTGATTGTAAATC

AGTGATATGGGCTTCTGTGTACCTTATTTCACAATGATAATCAATTGATAAGCTGGATCG

TGATGAGCGGACCGGATGAGCCGTCCATCTTACAGGCTTTTTAAAATAGAATTATAGAGT

GGGTGGTATGAGGTCCACCAAAACCTGTAACATGGAATTACAATCATAGCCGTTAATCGG

TTCATCTCATCCCCTCATCGTGATCCAGCATGTCAAACAGAGATGGGTTTCTGGAGAAAT

GGATGTATGTAAGCCCACAAATCACAAAAATCTGACATTATGATGGAGAGAATGCAAGGA

GTGTAAGTGAATCTACGCCGTTAAATGGATTTCATTGTACTTAATTTCATTTTGTCAAAT

ATTTTGTAGGACGAGGATGATGGACAAGGTGGGCGTAGGAAGAAGGGTTTGACGGAGAAG

ATCAAAGAGAAGCTGCCAGGTGGAAACAAGACGACAGGTGTCTGTCATCCAGGGACTCAG

GGTGTACAGGGGGGTCGCGAGCATGAGAAGCCAGGTTGCGGTCAGGGTGGACAGGTGGGC

CGCGAGCATGAGAAGACAGGTTTCGGTCATCCAGGGACTCAGGGTGGACAGGTGGGCAGC

GAGCAGGAGAAGAAGGGTATGATTGAGAAGATCAAGGAGAAGCTGCCAGGTCACAAGTAG

GATGTGTGTCCACCATACACGTGGAGTCTACACCTACGTGTCTTATGTTACTTATAATAC

GCATGGAGTGTGTCCGGAGTCTGTAATAATCTGCGCGTATCTGTTATGTC

>HNSN43

AGCCGTTGGATCTAGTTGAAAATGTCAGAGACGCGTGATGAGTATGGCAACCAGGTTCGC

CAAACCGACGAGTATGGGAACCCGATTCAGCATAGTGGCACCGGGACAAAGCCCGGTTCG

GGCATACATGGTGGGGGCCATGGGATTGGCACAGGTGGTGGTGGTGGACAAGGCAAGCTC

CACCGTCCAGGCTCCGGCTCTTCCTCTGTAAGTATATTCGATGTTTCTCAAGGGACATTT

CACTTGGTATATCGTGTTGGGATGACTACATTAGTTGATTAACGGTTGTGATTGTAAATC

AGTGATATGGGCTTCTGTGTACCTTATTTCACAATGATAATCAATTGATAAGCTGGATCG

TGATGAGCGGACCGGATGAGCCGTCCATCTTACAGGCTTTTTAAAATAGAATTATAGAGT

GGGTGGTATGAGGTCCACCAAAACCTGTAACATGGAATTACAATCATAGCCGTTAATCGG

TTCATCTCATCCCCTCATCGTGATCCAGCATGTCAAACAGAGATGGGTTTCTGGAGAAAT

GGATGTATGTAAGCCCACAAATCACAAAAATCTGACATTATGATGGAGAGAATGCAAGGA

GTGTAAGTGAATCTACGCCGTTAAATGGATTTCATTGTACTTAATTTCATTTTGTCAAAT

ATTTTGTAGGACGAGGATGATGGACAAGGTGGGCGTAGGAAGAAGGGTTTGACGGAGAAG

ATCAAAGAGAAGCTGCCAGGTGGAAACAAGACGACAGGTGTCTGTCATCCAGGGACTCAG

GGTGTACAGGGGGGTCGCGAGCATGAGAAGCCAGGTTGCGGTCAGGGTGGACAGGTGGGC

CGCGAGCATGAGAAGACAGGTTTCGGTCATCCAGGGACTCAGGGTGGACAGGTGGGCAGC

GAGCAGGAGAAGAAGGGTATGATTGAGAAGATCAAGGAGAAGCTGCCAGGTCACAAGTAG

GATGTGTGTCCACCATACACGTGGAGTCTACACCTACGTGTCTTATGTTACTTATAATAC

GCATGGAGTGTGTCCGGAGTCTGTAATAATCTGCGCGTATCTGTTATGTC

>HNSN44

AGCCGTTGGATCTAGTTGAAAATGTCAGAGACGCGTGATGAGTATGGCAACCAGGTTCGC

CAAACCGACGAGTATGGGAACCCGATTCAGCATAGTGGCACCGGGACAAAGCCCGGTTCG

GGCATACATGGTGGGGGCCATGGGATTGGCACAGGTGGTGGTGGTGGACAAGGCAAGCTC

CACCGTCCAGGCTCCGGCTCTTCCTCTGTAAGTATATTCGATGTTTCTCAAGGGTCATTT

CACTTGGTATATCGTGTTGGGATGACTAGATTAGTTGATTAACGGTTGTGATTGTAAATC

AGTGATATGGGTTTCTGTGTACCTTATTTCACAATGATAATCAATTGATACGCTGGATCG

TGATGAGCGGACCGGATGAGCCGTCCATCTTACAGGCTTTTTAAAATAGAATTATAGGGT

GGGTGGTATGAGGTCTACCAAGCCTGTAACATGGAATTACAATCATAGCCGTTAATCGGT

TCATCTCATCCGCTCATCGTGATCCAGCATGTCAAACAGAGATGGGTTTATGGAGAAATG

GATGTATGTAAGCCAACAAATCACAAAAAAAATCTGACATAATGATGGAGAGAATGCAAG

GAGTGTAAGTGAATCTACGCCGTTAAATGGATTTCATTGTACTTAATTTCAATTTCGTTT

TGTCAAATATTTTGTAGGACGAGGATGATGGACAAGGTGGGCGGAGGAAGAAGGGTTTGA

CGGAGAAGATCAAAGAGAAGCTGCCAGGTGGAAACAAGACGACAGGTGTCTGTCATCCAG

GGACTCAGGGTGTACAGGGGGGTCGCGAGCATGAGAAGCCAGGTTGCGGTCAGGGTGGAC

AGGTGGGCCGCGAGCATGAGAAGACAGGTTTCGGTCATCCAGGGACTCAGGGTGGACAGG

TGGGCAGCGAGCAGGAGAAGAAGGGTATGATTGAGAAGATCAAGGAGAAGCTGCCAGGTC

ACAAGTAGGATGTGTGTCCACCATACACGTGGAGTCTACACCTACGTGTCTTATGTTACT

TATAATACGCATGGAGTGTGTCCGGAGTCTGTAATAATCTGCGCGTATCTGTTATGTC

>HNSN54

AGCCGTTGGATCTAGTTGAAAATGTCAGAGACGCGTGATGAGTATGGCAACCAGGTTCGC

CAAACCGACGAGTATGGGAACCCGATTCAGCATACTGGCACCGGGACAAAGCCCGGTTCG

GGCATACATGGTGGGGGCCATGGGATTGGCACAGGTGGTGGTGGTGGACAAGGCAAGCTC

CACCGTCCAGGCTCCGGCTCTTCCTCTGTAAGTATATTCGATGTTTCTCAAGGATCATTT

CACTTGGTATATAGTGTTGGGATGACTAGATTAGTTGATTAACGGTTGTGATTGTAAATC

AGTGATATGGGCTTCTATGTACCTTATTTCACAATGATAATCAATTGATACGCTGGATCG

TGATGAGCGGACCGGATGAGCCGTCCATCTTACAGGCTTTTTAAAATAGAATTATAGGGT

GGGTGGTATGAGGTCTACCAAGCCTGTAACATGGAATTACAATCATAGCCGTTAATCGGT

TCATCTCATCCGCTCATCGTGATCCAGCATGTCAAACAGAGATGGGTTTATGGAGAAATG

GATGTATGTAAGCCAACAAATCACAAAAAAAATCTGACATAATGATGGAGAGAATGCAAG

GAGTGTAAGTGAATCTACGCCGTTAAATGGATTTCATTGTACTTAATTTCAATTTCGTTT

TGTCAAATATTTTGTAGGACGAGGATGATGGACAAGGTGGGCGGAGGAAGAAGGGTTTGA

CGGAGAAGATCAAAGAGAAGCTGCCAGGTGGAAACAAGACGACAGGTGTCTGTCATCCAG

GGACTCAGGGTGTACAGGGGGGTCGCGAGCATGAGAAGCCAGGTTGCGGTCAGGGTGGAC

AGGTGGGCCGCGAGCATGAGAAGACAGGTTTCGGTCATCCAGGGACTCAGGGTGGACAGG

TGGGCAGCGAGCAGGAGAAGAAGGGTATGATTGAGAAGATCAAGGAGAAGCTGCCAGGTC

ACAAGTAGGATGTGTGTCCACCATACACGTGGAGTCTACACCTACGTGTCTTATGTTACT

TATAATACGCATGGAGTGTGTCCGGAGTCTGTAATAATCTGCGCGTATCTGTTATGTC

>HNSN55

AGCCGTTGGATCTAGTTGAAAATGTCAGAGACGCGTGATGAGTATGGCAACCAGGTTCGC

CAAACCGACGAGTATGGGAACCCGATTCAGCATACTGGCACCGGGACAAAGCCCGGTTCG

GGCATACATGGTGGGGGCCATGGGATTGGCACAGGTGGTGGTGGTGGACAAGGCAAGCTC

CACCGTCCAGGCTCCGGCTCTTCCTCTGTAAGTATATTCGATGTTTCTCAAGGGACATTT

CACTTGGTATATCGTGTTGGGATGACTACATTAGTTGATTAACGGTTGTGATTGTAAATC

AGTGATATGGGCTTCTGTGTACCTTATTTCACAATGATAATCAATTGATAAGCTGGATCG

TGATGAGCGGACCGGATGAGCCGTCCATCTTACAGGCTTTTTAAAATAGAATTATAGAGT

GGGTGGTATGAGGTCCACCAAAACCTGTAACATGGAATTACAATCATAGCCGTTAATCGG

TTCATCTCATCCCCTCATCGTGATCCAGCATGTCAAACAGAGATGGGTTTCTGGAGAAAT

GGATGTATGTAAGCCCACAAATCACAAAAATCTGACATTATGATGGAGAGAATGCAAGGA

GTGTAAGTGAATCTACGCCGTTAAATGGATTTCATTGTACTTAATTTCATTTTGTCAAAT

ATTTTGTAGGACGAGGATGATGGACAAGGTGGGCGTAGGAAGAAGGGTTTGACGGAGAAG

ATCAAAGAGAAGCTGCCAGGTGGAAACAAGACGACAGGTGTCTGTCATCCAGGGACTCAG

GGTGTACAGGGGGGTCGCGAGCATGAGAAGCCAGGTTGCGGTCAGGGTGGACAGGTGGGC

CGCGAGCATGAGAAGACAGGTTTCGGTCATCCAGGGACTCAGGGTGGACAGGTGGGCAGC

GAGCAGGAGAAGAAGGGTATGATTGAGAAGATCAAGGAGAAGCTGCCAGGTCACAAGTAG

GATGTGTGTCCACCATACACGTGGAGTCTACACCTACGTGTCTTATGTTACTTATAATAC

GCATGGAGTGTGTCCGGAGTCTGTAATAATCTGCGCGTATCTGTTATGTC

>HNSN56

AGCCGTTGGATCTAGTTGAAAATGTCAGAGACGCGTGATGAGTATGGCAACCAGGTTCGC

CAAACCGACGAGTATGGGAACCCGATTCAGCATACTGGCACCGGGACAAAGCCCGGTTCG

GGCATACATGGTGGGGGCCATGGGATTGGCACAGGTGGTGGTGGTGGACAAGGCAAGCTC

CACCGTCCAGGCTCCGGCTCTTCCTCTGTAAGTATATTCGATGTTTCTCAAGGATCATTT

CACTTGGTATATAGTGTTGGGATGACTAGATTAGTTGATTAACGGTTGTGATTGTAAATC

AGTGATATGGGCTTCTATGTACCTTATTTCACAATGATAATCAATTGATACGCTGGATCG

TGATGAGCGGACCGGATGAGCCGTCCATCTTACAGGCTTTTTAAAATAGAATTATAGGGT

GGGTGGTATGAGGTCTACCAAGCCTGTAACATGGAATTACAATCATAGCCGTTAATCGGT

TCATCTCATCCGCTCATCGTGATCCAGCATGTCAAACAGAGATGGGTTTATGGAGAAATG

GATGTATGTAAGCCAACAAATCACAAAAAAAATCTGACATAATGATGGAGAGAATGCAAG

GAGTGTAAGTGAATCTACGCCGTTAAATGGATTTCATTGTACTTAATTTCAATTTCGTTT

TGTCAAATATTTTGTAGGACGAGGATGATGGACAAGGTGGGCGGAGGAAGAAGGGTTTGA

CGGAGAAGATCAAAGAGAAGCTGCCAGGTGGAAACAAGACGACAGGTGTCTGTCATCCAG

GGACTCAGGGTGTACAGGGGGGTCGCGAGCATGAGAAGCCAGGTTGCGGTCAGGGTGGAC

AGGTGGGCCGCGAGCATGAGAAGACAGGTTTCGGTCATCCAGGGACTCAGGGTGGACAGG

TGGGCAGCGAGCAGGAGAAGAAGGGTATGATTGAGAAGATCAAGGAGAAGCTGCCAGGTC

ACAAGTAGGATGTGTGTCCACCATACACGTGGAGTCTACACCTACGTGTCTTATGTTACT

TATAATACGCATGGAGTGTGTCCGGAGTCTGTAATAATCTGCGCGTATCTGTTATGTC

>GXLY3132

AGCCGTTGGATCTAGTTGAAAATGTCAGAGACGCGTGATGAGTATGGCAACCAGGTTCGC

CAAACCGACGAGTATGGGAACCCGATTCAGCATACTGGCACCGGGACAAAGCCCGGTTCG

GGCATACATGGTGGGGGCCATGGGATTGGCACAGGTGGTGGTGGTGGACAAGGCAAGCTC

CACCGTCCAGGCTCCGGCTCTTCCTCTGTAAGTATATTCGATGTTTCTCAAGGGTCATTT

CACTTGGTATATCGTGTTGGGATGACTAGATTAGTTGATTAACGGTTGTGATTGTAAATC

AGTGATATGGGCTTCTGTGTACCTTATTTCACAATGATAATCAATTGATACGCTGGATCG

TGATGAGCGGACCGGATGAGCCGTCCATCTTACAGGCTTTTTAAAATAGAATTATAGGGT

GGGTGGTATGAGGTCCACCAAGCCTGTAACATGGAATTACAATCATAACCGTTAATCGGT

TCATCTCATCCGCTCATCGTGATCCAGCATGTCAAACAGAGATGGGTTTATGGAGAAATG

GATGTATGTAAGCGCCAACAAATCACAAAAAAATCTGACATAATGATGGAGAGAATGCAA

GGAGTGTAAGTGAATCTACGCCGTTAAATGGATTTCATTGTAATTAATTTCGTTTTGTCA

AATATTTTGTAGGACGAGGATGATGGACAAGGTGAGCGTAGGAAGAAGGGTTTGACGGAG

AAGATCAAAGAGAAGCTGCCAGGTGGAAACAAGACGACAGGTGTCTGTCATCCAGGGACT

CAGGGTGTACAGGGGGGTCGCGAGCATGAGAAGCCAGGTTGCGGTCAGGGTGGACAGGTG

GGCCGCGAGCATGAGAAGACAGGTTTCGGTCATCCAGGAACTCAGGGTGGACAGGTGGGC

AGCGAGCAGGAGAAGAAGGGTATGATTGAGAAGATCAAGGAGAAGCTGCCAGGTCACAAG

TAGGATGTGTGTCCACCATACACGTGGAGTCTACATCTACGTGTCTTATGTTACTTATAA

TACGCATGGAGTGTGTCCGGAGTCTGTAATAATCTGCGCGTATCTGTTATGTC

>GXLY3133

AGCCGTTGGATCTAGTTGAAAATGTCAGAGACGCGTGATGAGTATGGCAACCAGGTTCGC

CAAACCGACGAGTATGGGAACCCGATTCAGCATACTGGCACCGGGACAAAGCCCGGTTCG

GGCATACATGGTGGGGGCCATGGGATTGGCACAGGTGGTGGTGGTGGACAAGGCAAGCTC

CACCGTCCAGGCTCCGGCTCTTCCTCTGTAAGTATATTCGATGTTTCTCAAGGGTCATTT

CACTTGGTATATAGTGTTGGGATGACTAGATTAGTTGATTAACGGTTGTGATTGTAAATC

AGTGATATGGGCTTCTATGTACCTTATTTCACAATGATAATCAATTGATACGCTGGATCG

TGATGAGCGGACCGGATGAGCCGTCCATCTTACAGGCTTTTTAAAATAGAATTATAGGGT

GGGTGGTATGAGGTCTACCAAGCCTGTAACATGGAATTACAATCATAGCCGTTAATCGGT

TCATCTCATCCGCTCATCGTGATCCAGCATGTCAAACAGAGATGGGTTTATGGAGAAATG

GATGTATGTAAGCCAACAAATCACAAAAAAAATCTGACATAATGATGGAGAGAATGCAAG

GAGTGTAAGATCTACGCCGTTAAATGGATTTCATTGTACTTAATTTCATTTTGTCAAATA

TTTTGTAGGACGAGGATGATGGACAAGGTGGGCGTAGGAAGAAGGGTTTGACGGAGAAGA

TCAAAGAGAAGCTGCCAGGTGGAAACAAGACGACAGGTGTCTGTCATCCAGGGACTCAGG

GAGTACAGGGGGGTCGCGAGCATGAGAAGCCAGGTTGCGGTCAGGGTGGACAGGTGGGCC

GCGAGCATGAGAAGACAGGTTTCGGTCATCCAGGGACTCAGGGTGGACAGGTGGGCAGCG

AGCAGGAGAAGAAGGGTATGATTGAGAAGATCAAGGAGAAGCTGCCAGGTCACAAGTAGG

ATGTGTGTCCACCATACACGTGGAGTCTACACCTACGTGTCTTATGTTACTTATAATACG

CATGGAGTGTGTCCGGAGTCTGTAATAATCTGCGCGTATCTGTTATGTC

>GXLY3136

AGCCGTTGGATCTAGTTGAAAATGTCAGAGACGCGTGATGAGTATGGCAACCAGGTTCGC

CAAACCGACGAGTATGGGAACCCGATTCAGCATACTGGCACCGGGACAAAGCCCGGTTCG

GGCATACATGGTGGGGGCCATGGGATTGGCACAGGTGGTGGTGGTGGACAAGGCAAGCTC

CACCGTCCAGGCTCCGGCTCTTCCTCTGTAAGTATATTCGATGTTTCTCAAGGGTCATTT

CACTTGGTATATAGTGTTGGGATGACTAGATTAGTTGATTAACGGTTGTGATTGTAAATC

AGTGATATGGGCTTCTATGTACCTTATTTCACAATGATAATCAATTGATACGCTGGATCG

TGATGAGCGGACCGGATGAGCCGTCCATCTTACAGGCTTTTTAAAATAGAATTATAGGGT

GGGTGGTATGAGGTCTACCAAGCCTGTAACATGGAATTACAATCATAGCCGTTAATCGGT

TCATCTCATCCGCTCATCGTGATCCAGCATGTCAAACAGAGATGGGTTTATGGAGAAATG

GATGTATGTAAGCCAACAAATCACAAAAAAAATCTGACATAATGATGGAGAGAATGCAAG

GAGTGTAAGATCTACGCCGTTAAATGGATTTCATTGTACTTAATTTCATTTTGTCAAATA

TTTTGTAGGACGAGGATGATGGACAAGGTGGGCGTAGGAAGAAGGGTTTGACGGAGAAGA

TCAAAGAGAAGCTGCCAGGTGGAAACAAGACGACAGGTGTCTGTCATCCAGGGACTCAGG

GAGTACAGGGGGGTCGCGAGCATGAGAAGCCAGGTTGCGGTCAGGGTGGACAGGTGGGCC

GCGAGCATGAGAAGACAGGTTTCGGTCATCCAGGGACTCAGGGTGGACAGGTGGGCAGCG

AGCAGGAGAAGAAGGGTATGATTGAGAAGATCAAGGAGAAGCTGCCAGGTCACAAGTAGG

ATGTGTGTCCACCATACACGTGGAGTCTACACCTACGTGTCTTATGTTACTTATAATACG

CATGGAGTGTGTCCGGAGTCTGTAATAATCTGCGCGTATCTGTTATGTC

>GXLY111

AGCCGTTGGATCTAGTTGAAAATGTCAGAGACGCGTGATGAGTATGGCAACCAGGTTCGC

CAAACCGACGAGTATGGGAACCCGATTCAGCATACTGGCACCGGGACAAAGCCCGGTTCG

GGCATACATGGTGGGGGCCATGGGATTGGCACAGGTGGTGGTGGTGGACAAGGCAAGCTC

CACCGTCCAGGCTCCGGCTCTTCCTCTGTAAGTATATTCGATGTTTCTCAAGGGTCATTT

CACTTGGTATATAGTGTTGGGATGACTAGATTAGTTGATTAACGGTTGTGATTGTAAATC

AGTGATATGGGCTTCTATGTACCTTATTTCACAATGATAATCAATTGATACGCTGGATCG

TGATGAGCGGACCGGATGAGCCGTCCATCTTACAGGCTTTTTAAAATAGAATTATAGGGT

GGGTGGTATGAGGTCTACCAAGCCTGTAACATGGAATTACAATCATAGCCGTTAATCGGT

TCATCTCATCCGCTCATCGTGATCCAGCATGTCAAACAGAGATGGGTTTATGGAGAAATG

GATGTATGTAAGCCAACAAATCACAAAAAAAATCTGACATAATGATGGAGAGAATGCAAG

GAGTGTAAGTGAATCTACGCCGTTAAATGGATTTCATTGTACTTAATTTCGTTTTGTCAA

ATATTTTGTAGGACGAGGATGATGGACAAGGTGGGCGTAGGAAGAAGGGTTTGACGGAGA

AGATCAAAGAGAAGCTGCCAGGTGGAAACAAGACGACAGGTGTCTGTCATCCAGGGACTC

AGGGTGTACAGGGGGGTCGCGAGCATGAGAAGCCAGGTTGCGGTCAGGGTGGACAGGTGG

GCCGCGAGCATGAGAAGACAGGTTTCGGTCATCCAGGGACTCAGGGTGGACAGGTGGGCA

GCGAGCAGGAGAAGAAGGGTATGATTGAGAAGATAAAGGAGAAGCTGCCAGGTCACAAGT

AGGATGTGTGTCCACCATACACGTGGAGTCTACACCTACGTGTCTTATGTTACTTATAAT

ACGCATGGAGTGTGTCCGGAGTCTGTAATAATCTGCGCGTATCTGTTATGTC

>GXLY112

AGCCGTTGGATCTAGTTGAAAATGTCAGAGACGCGTGATGAGTATGGCAACCAGGTTCGC

CAAACCGACGAGTATGGGAACCCGATTCAGCATACTGGCACCGGGACAAAGCCCGGTTCG

GGCATACATGGTGGGGGCCATGGGATTGGCACAGGTGGTGGTGGTGGACAAGGCAAGCTC

CACCGTCCAGGCTCCGGCTCTTCCTCTGTAAGTATATTCGATGTTTCTCAAGGGTCATTT

CACTTGGTATATAGTGTTGGGATGACTAGATTAGTTGATTAACGGTTGTGATTGTAAATC

AGTGATATGGGCTTCTATGTACCTTATTTCACAATGATAATCAATTGATACGCTGGATCG

TGATGAGCGGACCGGATGAGCCGTCCATCTTACAGGCTTTTTAAAATAGAATTATAGGGT

GGGTGGTATGAGGTCTACCAAGCCTGTAACATGGAATTACAATCATAGCCGTTAATCGGT

TCATCTCATCCGCTCATCGTGATCCAGCATGTCAAACAGAGATGGGTTTATGGAGAAATG

GATGTATGTAAGCCAACAAATCACAAAAAAAATCTGACATAATGATGGAGAGAATGCAAG

GAGTGTAAGATCTACGCCGTTAAATGGATTTCATTGTACTTAATTTCATTTTGTCAAATA

TTTTGTAGGACGAGGATGATGGACAAGGTGGGCGTAGGAAGAAGGGTTTGACGGAGAAGA

TCAAAGAGAAGCTGCCAGGTGGAAACAAGACGACAGGTGTCTGTCATCCAGGGACTCAGG

GAGTACAGGGGGGTCGCGAGCATGAGAAGCCAGGTTGCGGTCAGGGTGGACAGGTGGGCC

GCGAGCATGAGAAGACAGGTTTCGGTCATCCAGGGACTCAGGGTGGACAGGTGGGCAGCG

AGCAGGAGAAGAAGGGTATGATTGAGAAGATCAAGGAGAAGCTGCCAGGTCACAAGTAGG

ATGTGTGTCCACCATACACGTGGAGTCTACACCTACGTGTCTTATGTTACTTATAATACG

CATGGAGTGTGTCCGGAGTCTGTAATAATCTGCGCGTATCTGTTATGTC

>GXLY19

AGCCGTTGGATCTAGTTGAAAATGTCAGAGACGCGTGATGAGTATGGCAACCAGGTTCGC

CAAACCGACGAGTATGGGAACCCGATTCAGCATACTGGCACCGGGACAAAGCCCGGTTCG

GGCATACATGGTGGGGGCCATGGGATTGGCACAGGTGGTGGTGGTGGACAAGGCAAGCTC

CACCGTCCAGGCTCCGGCTCTTCCTCTGTAAGTATATTCGATGTTTCTCAAGGGTCATTT

CACTTGGTATATAGTGTTGGGATGACTAGATTAGTTGATTAACGGTTGTGATTGTAAATC

AGTGATATGGGCTTCTATGTACCTTATTTCACAATGATAATCAATTGATACGCTGGATCG

TGATGAGCGGACCGGATGAGCCGTCCATCTTACAGGCTTTTTAAAATAGAATTATAGGGT

GGGTGGTATGAGGTCTACCAAGCCTGTAACATGGAATTACAATCATAGCCGTTAATCGGT

TCATCTCATCCGCTCATCGTGATCCAGCATGTCAAACAGAGATGGGTTTATGGAGAAATG

GATGTATGTAAGCCAACAAATCACAAAAAAAATCTGACATAATGATGGAGAGAATGCAAG

GAGTGTAAGATCTACGCCGTTAAATGGATTTCATTGTACTTAATTTCATTTTGTCAAATA

TTTTGTAGGACGAGGATGATGGACAAGGTGGGCGTAGGAAGAAGGGTTTGACGGAGAAGA

TCAAAGAGAAGCTGCCAGGTGGAAACAAGACGACAGGTGTCTGTCATCCAGGGACTCAGG

GAGTACAGGGGGGTCGCGAGCATGAGAAGCCAGGTTGCGGTCAGGGTGGACAGGTGGGCC

GCGAGCATGAGAAGACAGGTTTCGGTCATCCAGGGACTCAGGGTGGACAGGTGGGCAGCG

AGCAGGAGAAGAAGGGTATGATTGAGAAGATCAAGGAGAAGCTGCCAGGTCACAAGTAGG

ATGTGTGTCCACCATACACGTGGAGTCTACACCTACGTGTCTTATGTTACTTATAATACG

CATGGAGTGTGTCCGGAGTCTGTAATAATCTGCGCGTATCTGTTATGTC

>GXLY41

AGCCGTTGGATCTAGTTGAAAATGTCAGAGACGCGTGATGAGTATGGCAACCAGGTTCGC

CAAACCGACGAGTATGAGAACCCGATTCAGCATACTGGCACCGGGACAAAGCCCGGTTCG

GGCATACATGGTGGGGGCCATGGGATTGGCACAGGTGGTGGTGGTGGACAAGGCAAGCTC

CACCGTCCAGGCTCCGGCTCTTCCTCTGTAAGTATATTCGATGTTTCTCGAGGGTCATTT

CACTTGGTATATCGTGTTGGGATGACTAGATTAGTTGATTAACGGTTGTGATTGTAAATC

AGTGATATGGGCTTGTGTGTACCTTATTTCACAATGATAATCAATTGATACGCTGGATCG

TGATGAGCGGACCGGATGAGCCGTCCATCTTACAGGCTTTTTAAAATAGAATTATAGGGT

GGGTGGTATGAAGTCTACCAAGCCTGTAACATGGAATTACAATCATAGCCGTTAATCGGT

TCATCTCATTCGCTCATCGTGATCCAGCATGTCAAACAGAGATGGGTTTATGGAGAAATG

GATGTATGTAAGCCAACAAATCACAAAAAAAATCTGACATAATGATGGAGAGAATGCAAG

GAGTGTAAGTGAATCTACGCCGTTAAATGGATTTCATTGTACTTAATTTCGTTTTGTCAA

ATATTTTGTAGGACGAGGATGATGGACAAGGTGGGCGTAGGAAGAAGGGTTTGACGGAGA

AGATCAAAGAGAAGCTGCCAGGTGGAAACAAGACGACAGGTGTCTGTCATCCAGGGACTC

AGGGTGTACAGGGGGGTCGCGAGCATGAGAAGCCAGGTTGCGGTCAGGGTGGACAGGTGG

GCCGCGAGCATGAGAAGACAGGTTTCGGTCATCCAGGGACTCAGGGTGGACAGGTGGGCA

GCGAGCAGGAGAAGAAGGGTATGATTGAGAAGATCAAGGAGAAGCTGCCAGGTCACAAGT

AGGATGTGTGTCCACCATACACGTGGAGTCTGCATCTACGTGTCTTATGTTACTTATAAT

ACGCATGGAGTGTGTCCGGAGTCTGTAATAATCTGCGCGTATCTGTTATGTC

>GXLY44

AGCCGTTGGATCTAGTTGAAAATGTCAGAGACGCGTGATGAGTATGGCAACCAGGTTCGC

CAAACCGACGAGTATGAGAACCCGATTCAGCATACTGGCACCGGGACAAAGCCCGGTTCG

GGCATACATGGTGGGGGCCATGGGATTGGCACAGGTGGTGGTGGTGGACAAGGCAAGCTC

CACCGTCCAGGCTCCGGCTCTTCCTCTGTAAGTATATTCGATGTTTCTCGAGGGTCATTT

CACTTGGTATATCGTGTTGGGATGACTAGATTAGTTGATTAACGGTTGTGATTGTAAATC

AGTGATATGGGCTTGTGTGTACCTTATTTCACAATGATAATCAATTGATACGCTGGATCG

TGATGAGCGGACCGGATGAGCCGTCCATCTTACAGGCTTTTTAAAATAGAATTATAGGGT

GGGTGGTATGAAGTCTACCAAGCCTGTAACATGGAATTACAATCATAGCCGTTAATCGGT

TCATCTCATTCGCTCATCGTGATCCAGCATGTCAAACAGAGATGGGTTTATGGAGAAATG

GATGTATGTAAGCCAACAAATCACAAAAAAAATCTGACATAATGATGGAGAGAATGCAAG

GAGTGTAAGTGAATCTACGCCGTTAAATGGATTTCATTGTACTTAATTTCGTTTTGTCAA

ATATTTTGTAGGACGAGGATGATGGACAAGGTGGGCGTAGGAAGAAGGGTTTGACGGAGA

AGATCAAAGAGAAGCTGCCAGGTGGAAACAAGACGACAGGTGTCTGTCATCCAGGGACTC

AGGGTGTACAGGGGGGTCGCGAGCATGAGAAGCCAGGTTGCGGTCAGGGTGGACAGGTGG

GCCGCGAGCATGAGAAGACAGGTTTCGGTCATCCAGGGACTCAGGGTGGACAGGTGGGCA

GCGAGCAGGAGAAGAAGGGTATGATTGAGAAGATCAAGGAGAAGCTGCCAGGTCACAAGT

AGGATGTGTGTCCACCATACACGTGGAGTCTGCATCTACGTGTCTTATGTTACTTATAAT

ACGCATGGAGTGTGTCCGGAGTCTGTAATAATCTGCGCGTATCTGTTATGTC

>GXLY47

AGCCGTTGGATCTAGTTGAAAATGTCAGAGACGCGTGATGAGTATGGCAACCAGGTTCGC

CAAACCGACGAGTATGAGAACCCGATTCAGCATACTGGCACCGGGACAAAGCCCGGTTCG

GGCATACATGGTGGGGGCCATGGGATTGGCACAGGTGGTGGTGGTGGACAAGGCAAGCTC

CACCGTCCAGGCTCCGGCTCTTCCTCTGTAAGTATATTCGATGTTTCTCGAGGGTCATTT

CACTTGGTATATCGTGTTGGGATGACTAGATTAGTTGATTAACGGTTGTGATTGTAAATC

AGTGATATGGGCTTGTGTGTACCTTATTTCACAATGATAATCAATTGATACGCTGGATCG

TGATGAGCGGACCGGATGAGCCGTCCATCTTACAGGCTTTTTAAAATAGAATTATAGGGT

GGGTGGTATGAAGTCTACCAAGCCTGTAACATGGAATTACAATCATAGCCGTTAATCGGT

TCATCTCATTCGCTCATCGTGATCCAGCATGTCAAACAGAGATGGGTTTATGGAGAAATG

GATGTATGTAAGCCAACAAATCACAAAAAAAATCTGACATAATGATGGAGAGAATGCAAG

GAGTGTAAGTGAATCTACGCCGTTAAATGGATTTCATTGTACTTAATTTCGTTTTGTCAA

ATATTTTGTAGGACGAGGATGATGGACAAGGTGGGCGTAGGAAGAAGGGTTTGACGGAGA

AGATCAAAGAGAAGCTGCCAGGTGGAAACAAGACGACAGGTGTCTGTCATCCAGGGACTC

AGGGTGTACAGGGGGGTCGCGAGCATGAGAAGCCAGGTTGCGGTCAGGGTGGACAGGTGG

GCCGCGAGCATGAGAAGACAGGTTTCGGTCATCCAGGGACTCAGGGTGGACAGGTGGGCA

GCGAGCAGGAGAAGAAGGGTATGATTGAGAAGATCAAGGAGAAGCTGCCAGGTCACAAGT

AGGATGTGTGTCCACCATACACGTGGAGTCTGCATCTACGTGTCTTATGTTACTTATAAT

ACGCATGGAGTGTGTCCGGAGTCTGTAATAATCTGCGCGTATCTGTTATGTC

>GXMES131

AGCCGTTGGATCTAGTTGAAAATGTCAGAGACGCGTGATGAGTATGGCAACCAGGTTCGC

CAAACCGACGAGTATGGGAACCCGATTCAGCATACTGGCACCGGGACAAAGCCCGGTTCG

GGCATACATGGTGGGGGCCATGGGATTGGCACAGGTGGTGGTGGTGGACAAGGCAAGCTC

CACCGTCCAGGCTCCGGCTCTTCCTCTGTAAGTATATTCGATGTTTCTCAAGGGTCATTT

CACTTGGTATATAGTGTTGGGATGACTAGATTAGTTGATTAACGGTTGTGATTGTAAATC

AGTGATATGGGCTTCTATGTACCTTATTTCACAATGATAATCAATTGATACGCTGGATCG

TGATGAGCGGACCGGATGAGCCGTCCATCTTACAGGCTTTTTAAAATAGAATTATAGGGT

GGGTGGTATGAGGTCTACCAAGCCTGTAACATGGAATTACAATCATAGCCGTTAATCGGT

TCATCTCATCCGCTCATCGTGATCCAGCATGTCAAACAGAGATGGGTTTATGGAGAAATG

GATGTATGTAAGCCAACAAATCACAAAAAAAATCTGACATAATGATGGAGAGAATGCAAG

GAGTGTAAGATCTACGCCGTTAAATGGATTTCATTGTACTTAATTTCATTTTGTCAAATA

TTTTGTAGGACGAGGATGATGGACAAGGTGGGCGTAGGAAGAAGGGTTTGACGGAGAAGA

TCAAAGAGAAGCTGCCAGGTGGAAACAAGACGACAGGTGTCTGTCATCCAGGGACTCAGG

GTGTACAGGGGGGTCGCGAGCATGAGAAGCAAGGTTGCGGTCAGGGTGGACAGGTGGGCC

GCGAGCATGAGAAGACAGGTTTCGGTCATCCAGGGACTCAGGGTGGACAGGTGGGCAGCG

AGCAGGAGAAGAAGGGTATGATTGAGAAGATCAAGGAGAAGCTGCCAGGTCACAAGTAGG

ATGTGTGTCCACCATACACGTGGAGTCTACACCTACGTGTCTTATGTTACTTATAATACG

CATGGAGTGTGTCCGGAGTCTGTAATAATCTGCGCGTATCTGTTATGTC

>GXMES132

AGCCGTTGGATCTAGTTGAAAATGTCAGAGACGCGTGATGAGTATGGCAACCAGGTTCGC

CAAACCGACGAGTATGGGAACCCGATTCAGCATACTGGCACCGGGACAAAGCCCGGTTCG

GGCATACATGGTGGGGGCCATGGGATTGGCACAGGTGGTGGTGGTGGACAAGGCAAGCTC

CACCGTCCAGGCTCCGGCTCTTCCTCTGTAAGTATATTCGATGTTTCTCAAGGGTCATTT

CACTTGGTATATAGTGTTGGGATGACTAGATTAGTTGATTAACGGTTGTGATTGTAAATC

AGTGATATGGGCTTCTATGTACCTTATTTCACAATGATAATCAATTGATACGCTGGATCG

TGATGAGCGGACCGGATGAGCCGTCCATCTTACAGGCTTTTTAAAATAGAATTATAGGGT

GGGTGGTATGAGGTCTACCAAGCCTGTAACATGGAATTACAATCATAGCCGTTAATCGGT

TCATCTCATCCGCTCATCGTGATCCAGCATGTCAAACAGAGATGGGTTTATGGAGAAATG

GATGTATGTAAGCCAACAAATCACAAAAAAAATCTGACATAATGATGGAGAGAATGCAAG

GAGTGTAAGATCTACGCCGTTAAATGGATTTCATTGTACTTAATTTCATTTTGTCAAATA

TTTTGTAGGACGAGGATGATGGACAAGGTGGGCGTAGGAAGAAGGGTTTGACGGAGAAGA

TCAAAGAGAAGCTGCCAGGTGGAAACAAGACGACAGGTGTCTGTCATCCAGGGACTCAGG

GTGTACAGGGGGGTCGCGAGCATGAGAAGCAAGGTTGCGGTCAGGGTGGACAGGTGGGCC

GCGAGCATGAGAAGACAGGTTTCGGTCATCCAGGGACTCAGGGTGGACAGGTGGGCAGCG

AGCAGGAGAAGAAGGGTATGATTGAGAAGATCAAGGAGAAGCTGCCAGGTCACAAGTAGG

ATGTGTGTCCACCATACACGTGGAGTCTACACCTACGTGTCTTATGTTACTTATAATACG

CATGGAGTGTGTCCGGAGTCTGTAATAATCTGCGCGTATCTGTTATGTC

>GXMES133

AGCCGTTGGATCTAGTTGAAAATGTCAGAGACGCGTGATGAGTATGGCAACCAGGTTCGC

CAAACCGACGAGTATGGGAACCCGATTCAGCATACTGGCACCGGGACAAAGCCCGGTTCG

GGCATACATGGTGGGGGCCATGGGATTGGCACAGGTGGTGGTGGTGGACAAGGCAAGCTC

CACCGTCCAGGCTCCGGCTCTTCCTCTGTAAGTATATTCGATGTTTCTCAAGGGTCATTT

CACTTGGTATATAGTGTTGGGATGACTAGATTAGTTGATTAACGGTTGTGATTGTAAATC

AGTGATATGGGCTTCTATGTACCTTATTTCACAATGATAATCAATTGATACGCTGGATCG

TGATGAGCGGACCGGATGAGCCGTCCATCTTACAGGCTTTTTAAAATAGAATTATAGGGT

GGGTGGTATGAGGTCTACCAAGCCTGTAACATGGAATTACAATCATAGCCGTTAATCGGT

TCATCTCATCCGCTCATCGTGATCCAGCATGTCAAACAGAGATGGGTTTATGGAGAAATG

GATGTATGTAAGCCAACAAATCACAAAAAAAATCTGACATAATGATGGAGAGAATGCAAG

GAGTGTAAGATCTACGCCGTTAAATGGATTTCATTGTACTTAATTTCATTTTGTCAAATA

TTTTGTAGGACGAGGATGATGGACAAGGTGGGCGTAGGAAGAAGGGTTTGACGGAGAAGA

TCAAAGAGAAGCTGCCAGGTGGAAACAAGACGACAGGTGTCTGTCATCCAGGGACTCAGG

GTGTACAGGGGGGTCGCGAGCATGAGAAGCAAGGTTGCGGTCAGGGTGGACAGGTGGGCC

GCGAGCATGAGAAGACAGGTTTCGGTCATCCAGGGACTCAGGGTGGACAGGTGGGCAGCG

AGCAGGAGAAGAAGGGTATGATTGAGAAGATCAAGGAGAAGCTGCCAGGTCACAAGTAGG

ATGTGTGTCCACCATACACGTGGAGTCTACACCTACGTGTCTTATGTTACTTATAATACG

CATGGAGTGTGTCCGGAGTCTGTAATAATCTGCGCGTATCTGTTATGTC

>GXMES2491

AGCCGTTGGATCTAGTTGAAAATGTCAGAGACGCGTGATGAGTATGGCAACCAGGTTCGC

CAAACCGACGAGTATGGGAACCCGATTCAGCATACTGGCACCGGGACAAAGCCCGGTTCG

GGCATACATGGTGGGGGCCATGGGATTGGCACAGGTGGTGGTGGTGGACAAGGCAAGCTC

CACCGTCCAGGCTCCGGCTCTTCCTCTGTAAGTATATTCCCTGTTTCTCAAGGGTCATTT

CACTTGGTATATAGTGTTGGGATGACTAGATTAGTTGATTAACGGTTGTGATTGTAAATC

AGTGATATGGGCTTCTATGTACCTTATTTCACAATGATAATCAATTGATACGCTGGATCG

TGATGAGCGGACCGGATGAGCCGTCCATCTTACAGGCTTTTTAAAATAGAATTATAGGGT

GGGTGGTATGAGGTCTACCAAGCCTGTAACATGGAATTACAATCATAGCCGTTAATCGGT

TCATCTCATCCGCTCATCGTGATCCAGCATGTCAAACAGAGATGGGTTTATGGAGAAATG

GATGTATGTAAGCCAACAAATCACAAAAAAAATCTGACATAATGATGGAGAGAATGCAAG

GAGTGTAAGATCTACGCCGTTAAATGGATTTCATTGTACTTAATTTCATTTTGTCAAATA

TTTTGTAGGACGAGGATGATGGACAAGGTGGGCGTAGGAAGAAGGGTTTGACGGAGAAGA

TCAAAGAGAAGCTGCCAGGTGGAAACAAGACGACAGGTGTCTGTCATCCAGGGACTCAGG

GTGTACAGGGGGGTCGCGAGCATGAGAAGCAAGGTTGCGGTCAGGGTGGACAGGTGGGCC

GCGAGCATGAGAAGACAGGTTTCGGTCATCCAGGGACTCAGGGTGGACAGGTGGGCAGCG

AGCAGGAGAAGAAGGGTATGATTGAGAAGATCAAGGAGAAGCTGCCAGGTCACAAGTAGG

ATGTGTGTCCACCATACACGTGGAGTCTACACCTACGTGTCTTATGTTACTTATAATACG

CATGGAGTGTGTCCGGAGTCTGTAATAATCTGCGCGTATCTGTTATGTC

>GXMES2492

AGCCGTTGGATCTAGTTGAAAATGTCAGAGACGCGTGATGAGTATGGCAACCAGGTTCGC

CAAACCGACGAGTATGGGAACCCGATTCAGCATACTGGCACCGGGACAAAGCCCGGTTCG

GGCATACATGGTGGGGGCCATGGGATTGGCACAGGTGGTGGTGGTGGACAAGGCAAGCTC

CACCGTCCAGGCTCCGGCTCTTCCTCTGTAAGTATATTCGATGTTTCTCAAGGGTCATTT

CACTTGGTATATAGTGTTGGGATGACTAGATTAGTTGATTAACGGTTGTGATTGTAAATC

AGTGATATGGGCTTCTATGTACCTTATTTCACAATGATAATCAATTGATACGCTGGATCG

TGATGAGCGGACCGGATGAGCCGTCCATCTTACAGGCTTTTTAAAATAGAATTATAGGGT

GGGTGGTATGAGGTCTACCAAGCCTGTAACATGGAATTACAATCATAGCCGTTAATCGGT

TCATCTCATCCGCTCATCGTGATCCAGCATGTCAAACAGAGATGGGTTTATGGAGAAATG

GATGTATGTAAGCCAACAAATCACAAAAAAAATCTGACATAATGATGGAGAGAATGCAAG

GAGTGTAAGATCTACGCCGTTAAATGGATTTCATTGTACTTAATTTCATTTTGTCAAATA

TTTTGTAGGACGAGGATGATGGACAAGGTGGGCGTAGGAAGAAGGGTTTGACGGAGAAGA

TCAAAGAGAAGCTGCCAGGTGGAAACAAGACGACAGGTGTCTGTCATCCAGGGACTCAGG

GTGTACAGGGGGGTCGCGAGCATGAGAAGCAAGGTTGCGGTCAGGGTGGACAGGTGGGCC

GCGAGCATGAGAAGACAGGTTTCGGTCATCCAGGGACTCAGGGTGGACAGGTGGGCAGCG

AGCAGGAGAAGAAGGGTATGATTGAGAAGATCAAGGAGAAGCTGCCAGGTCACAAGTAGG

ATGTGTGTCCACCATACACGTGGAGTCTACACCTACGTGTCTTATGTTACTTATAATACG

CATGGAGTGTGTCCGGAGTCTGTAATAATCTGCGCGTATCTGTTATGTC

>GXMES2496

AGCCGTTGGATCTAGTTGAAAATGTCAGAGACGCGTGATGAGTATGGCAACCAGGTTCGC

CAAACCGACGAGTATGGGAACCCGATTCAGCATACTGGCACCGGGACAAAGCCCGGTTCG

GGCATACATGGTGGGGGCCATGGGATTGGCACAGGTGGTGGTGGTGGACAAGGCAAGCTC

CACCGTCCAGGCTCCGGCTCTTCCTCTGTAAGTATATTCCCTGTTTCTCAAGGGTCATTT

CACTTGGTATATAGTGTTGGGATGACTAGATTAGTTGATTAACGGTTGTGATTGTAAATC

AGTGATATGGGCTTCTATGTACCTTATTTCACAATGATAATCAATTGATACGCTGGATCG

TGATGAGCGGACCGGATGAGCCGTCCATCTTACAGGCTTTTTAAAATAGAATTATAGGGT

GGGTGGTATGAGGTCTACCAAGCCTGTAACATGGAATTACAATCATAGCCGTTAATCGGT

TCATCTCATCCGCTCATCGTGATCCAGCATGTCAAACAGAGATGGGTTTATGGAGAAATG

GATGTATGTAAGCCAACAAATCACAAAAAAAATCTGACATAATGATGGAGAGAATGCAAG

GAGTGTAAGATCTACGCCGTTAAATGGATTTCATTGTACTTAATTTCATTTTGTCAAATA

TTTTGTAGGACGAGGATGATGGACAAGGTGGGCGTAGGAAGAAGGGTTTGACGGAGAAGA

TCAAAGAGAAGCTGCCAGGTGGAAACAAGACGACAGGTGTCTGTCATCCAGGGACTCAGG

GTGTACAGGGGGGTCGCGAGCATGAGAAGCAAGGTTGCGGTCAGGGTGGACAGGTGGGCC

GCGAGCATGAGAAGACAGGTTTCGGTCATCCAGGGACTCAGGGTGGACAGGTGGGCAGCG

AGCAGGAGAAGAAGGGTATGATTGAGAAGATCAAGGAGAAGCTGCCAGGTCACAAGTAGG

ATGTGTGTCCACCATACACGTGGAGTCTACACCTACGTGTCTTATGTTACTTATAATACG

CATGGAGTGTGTCCGGAGTCTGTAATAATCTGCGCGTATCTGTTATGTC

>GXMES2364

AGCCGTTGGATCTAGTTGAAAATGTCAGAGACGCGTGATGAGTATGGCAACCAGGTTCGC

CAAACCGACGAGTATGGGAACCCGATTCAGCATACTGGCACCGGGACAAAGCCCGGTTCG

GGCATACATGGTGGGGGCCATGGGATTGGCACAGGTGGTGGTGGTGGACAAGGCAAGCTC

CACCGTCCAGGCTCCGGCTCTTCCTCTGTAAGTATATTCCCTGTTTCTCAAGGGTCATTT

CACTTGGTATATAGTGTTGGGATGACTAGATTAGTTGATTAACGGTTGTGATTGTAAATC

AGTGATATGGGCTTCTATGTACCTTATTTCACAATGATAATCAATTGATACGCTGGATCG

TGATGAGCGGACCGGATGAGCCGTCCATCTTACAGGCTTTTTAAAATAGAATTATAGGGT

GGGTGGTATGAGGTCTACCAAGCCTGTAACATGGAATTACAATCATAGCCGTTAATCGGT

TCATCTCATCCGCTCATCGTGATCCAGCATGTCAAACAGAGATGGGTTTATGGAGAAATG

GATGTATGTAAGCCAACAAATCACAAAAAAAATCTGACATAATGATGGAGAGAATGCAAG

GAGTGTAAGTGAATCTACGCCGTTAAATGGATTTCATTGTACTTAATTTCAATTTCGTCT

TGTCAAATATTTTGTAGGACGAGGATGATGGACAAGGTGGGCGGAGGAAGAAGGGTTTGA

CGGAGAAGATCAAAGAGAAGCTGCCAGGTGGAAACAAGACGACAGGTGTCTGTCATCCAG

GGACTCAGGGAGTACAGGGGGGTCGCGAGCATGAGAAGCCAGGTTGCGGTCAGGGTGGAC

AGGTGGGCCGCGAGCATGAGAAGACAGGTTTCGGTCATCCAGGGACTCAGGGTGGACAGG

TGGGCAGCGAGCAGGAGAAGAAGGGTATGATTGAGAAGATCAAGGAGAAGCTGCCAGGTC

ACAAGTAGGATGTGTGTCCACCATACACGTGGAGTCTACACCTACGTGTCTTATGTTACT

TATAATACGCATGGAGTGTGTCCGGAGTCTGTAATAATCTGCGCGTATCTGTTATGTC

>GXMES2366

AGCCGTTGGATCTAGTTGAAAATGTCAGAGACGCGTGATGAGTATGGCAACCAGGTTCGC

CAAACCGACGAGTATGGGAACCCGATTCAGCATACTGGCACCGGGACAAAGCCCGGTTCG

GGCATACATGGTGGGGGCCATGGGATTGGCACAGGTGGTGGTGGTGGACAAGGCAAGCTC

CACCGTCCAGGCTCCGGCTCTTCCTCTGTAAGTATATTCCCTGTTTCTCAAGGGTCATTT

CACTTGGTATATAGTGTTGGGATGACTAGATTAGTTGATTAACGGTTGTGATTGTAAATC

AGTGATATGGGCTTCTATGTACCTTATTTCACAATGATAATCAATTGATACGCTGGATCG

TGATGAGCGGACCGGATGAGCCGTCCATCTTACAGGCTTTTTAAAATAGAATTATAGGGT

GGGTGGTATGAGGTCTACCAAGCCTGTAACATGGAATTACAATCATAGCCGTTAATCGGT

TCATCTCATCCGCTCATCGTGATCCAGCATGTCAAACAGAGATGGGTTTATGGAGAAATG

GATGTATGTAAGCCAACAAATCACAAAAAAAATCTGACATAATGATGGAGAGAATGCAAG

GAGTGTAAGTGAATCTACGCCGTTAAATGGATTTCATTGTACTTAATTTCAATTTCGTCT

TGTCAAATATTTTGTAGGACGAGGATGATGGACAAGGTGGGCGGAGGAAGAAGGGTTTGA

CGGAGAAGATCAAAGAGAAGCTGCCAGGTGGAAACAAGACGACAGGTGTCTGTCATCCAG

GGACTCAGGGAGTACAGGGGGGTCGCGAGCATGAGAAGCCAGGTTGCGGTCAGGGTGGAC

AGGTGGGCCGCGAGCATGAGAAGACAGGTTTCGGTCATCCAGGGACTCAGGGTGGACAGG

TGGGCAGCGAGCAGGAGAAGAAGGGTATGATTGAGAAGATCAAGGAGAAGCTGCCAGGTC

ACAAGTAGGATGTGTGTCCACCATACACGTGGAGTCTACACCTACGTGTCTTATGTTACT

TATAATACGCATGGAGTGTGTCCGGAGTCTGTAATAATCTGCGCGTATCTGTTATGTC

>GXMES2368

AGCCGTTGGATCTAGTTGAAAATGTCAGAGACGCGTGATGAGTATGGCAACCAGGTTCGC

CAAACCGACGAGTATGGGAACCCGATTCAGCATACTGGCACCGGGACAAAGCCCGGTTCG

GGCATACATGGTGGGGGCCATGGGATTGGCACAGGTGGTGGTGGTGGACAAGGCAAGCTC

CACCGTCCAGGCTCCGGCTCTTCCTCTGTAAGTATATTCCCTGTTTCTCAAGGGTCATTT

CACTTGGTATATAGTGTTGGGATGACTAGATTAGTTGATTAACGGTTGTGATTGTAAATC

AGTGATATGGGCTTCTATGTACCTTATTTCACAATGATAATCAATTGATACGCTGGATCG

TGATGAGCGGACCGGATGAGCCGTCCATCTTACAGGCTTTTTAAAATAGAATTATAGGGT

GGGTGGTATGAGGTCTACCAAGCCTGTAACATGGAATTACAATCATAGCCGTTAATCGGT

TCATCTCATCCGCTCATCGTGATCCAGCATGTCAAACAGAGATGGGTTTATGGAGAAATG

GATGTATGTAAGCCAACAAATCACAAAAAAAATCTGACATAATGATGGAGAGAATGCAAG

GAGTGTAAGTGAATCTACGCCGTTAAATGGATTTCATTGTACTTAATTTCAATTTCGTCT

TGTCAAATATTTTGTAGGACGAGGATGATGGACAAGGTGGGCGGAGGAAGAAGGGTTTGA

CGGAGAAGATCAAAGAGAAGCTGCCAGGTGGAAACAAGACGACAGGTGTCTGTCATCCAG

GGACTCAGGGAGTACAGGGGGGTCGCGAGCATGAGAAGCCAGGTTGCGGTCAGGGTGGAC

AGGTGGGCCGCGAGCATGAGAAGACAGGTTTCGGTCATCCAGGGACTCAGGGTGGACAGG

TGGGCAGCGAGCAGGAGAAGAAGGGTATGATTGAGAAGATCAAGGAGAAGCTGCCAGGTC

ACAAGTAGGATGTGTGTCCACCATACACGTGGAGTCTACACCTACGTGTCTTATGTTACT

TATAATACGCATGGAGTGTGTCCGGAGTCTGTAATAATCTGCGCGTATCTGTTATGTC

>GXHP4104

AGCCGTTGGATCTAGTTGAAAATGTCAGAGACGCGTGATGAGTATGGCAACCAGGTTCGC

CAAACCGACGAGTATGAGAACCCGATTCAGCATACTGGCACCGGGACAAAGCCCGGTTCG

GGCATACATGGTGGGGGCCATGGGATTGGCACAGGTGGTGGTGGTGGACAAGGCAAGCTC

CACCGTCCAGGCTCCGGCTCTTCCTCTGTAAGTATATTCGATGTTTCTCGAGGGTCATTT

CACTTGGTATATCGTGTTGGGATGACTAGATTAGTTGATTAACGGTTGTGATTGTAAATC

AGTGATATGGGCTTGTGTGTACCTTATTTCACAATGATAATCAATTGATACGCTGGATCG

TGATGAGCGGACCGGATGAGCCGTCCATCTTACAGGCTTTTTAAAATAGAATTATAGGGT

GGGTGGTATGAAGTCTACCAAGCCTGTAACATGGAATTACAATCATAGCCGTTAATCGGT

TCATCTCATTCGCTCATCGTGATCCAGCATGTCAAACAGAGATGGGTTTATGGAGAAATG

GATGTATGTAAGCCAACAAATCACAAAAAAAATCTGACATAATGATGGAGAGAATGCAAG

GAGTGTAAGTGAATCTACGCCGTTAAATGGATTTCATTGTACTTAATTTCGTTTTGTCAA

ATATTTTGTAGGACGAGGATGATGGACAAGGTGGGCGTAGGAAGAAGGGTTTGACGGAGA

AGATCAAAGAGAAGCTGCCAGGTGGAAACAAGACGACAGGTGTCTGTCATCCAGGGACTC

AGGGTGTACAGGGGGGTCGCGAGCATGAGAAGCCAGGTTGCGGTCAGGGTGGACAGGTGG

GCCGCGAGCATGAGAAGACAGGTTTCGGTCATCCAGGGACTCAGGGTGGACAGGTGGGCA

GCGAGCAGGAGAAGAAGGGTATGATTGAGAAGATCAAGGAGAAGCTGCCAGGTCACAAGT

AGGATGTGTGTCCACCATACACGTGGAGTCTGCATCTACGTGTCTTATGTTACTTATAAT

ACGCATGGAGTGTGTCCGGAGTCTGTAATAATCTGCGCGTATCTGTTATGTC

>GXHP41018

AGCCGTTGGATCTAGTTGAAAATGTCAGAGACGCGTGATGAGTATGGCAACCAGGTTCGC

CAAACCGACGAGTATGAGAACCCGATTCAGCATACTGGCACCGGGACAAAGCCCGGTTCG

GGCATACATGGTGGGGGCCATGGGATTGGCACAGGTGGTGGTGGTGGACAAGGCAAGCTC

CACCGTCCAGGCTCCGGCTCTTCCTCTGTAAGTATATTCGATGTTTCTCGAGGGTCATTT

CACTTGGTATATCGTGTTGGGATGACTAGATTAGTTGATTAACGGTTGTGATTGTAAATC

AGTGATATGGGCTTGTGTGTACCTTATTTCACAATGATAATCAATTGATACGCTGGATCG

TGATGAGCGGACCGGATGAGCCGTCCATCTTACAGGCTTTTTAAAATAGAATTATAGGGT

GGGTGGTATGAAGTCTACCAAGCCTGTAACATGGAATTACAATCATAGCCGTTAATCGGT

TCATCTCATTCGCTCATCGTGATCCAGCATGTCAAACAGAGATGGGTTTATGGAGAAATG

GATGTATGTAAGCCAACAAATCACAAAAAAAATCTGACATAATGATGGAGAGAATGCAAG

GAGTGTAAGTGAATCTACGCCGTTAAATGGATTTCATTGTACTTAATTTCGTTTTGTCAA

ATATTTTGTAGGACGAGGATGATGGACAAGGTGGGCGTAGGAAGAAGGGTTTGACGGAGA

AGATCAAAGAGAAGCTGCCAGGTGGAAACAAGACGACAGGTGTCTGTCATCCAGGGACTC

AGGGTGTACAGGGGGGTCGCGAGCATGAGAAGCCAGGTTGCGGTCAGGGTGGACAGGTGG

GCCGCGAGCATGAGAAGACAGGTTTCGGTCATCCAGGGACTCAGGGTGGACAGGTGGGCA

GCGAGCAGGAGAAGAAGGGTATGATTGAGAAGATCAAGGAGAAGCTGCCAGGTCACAAGT

AGGATGTGTGTCCACCATACACGTGGAGTCTGCATCTACGTGTCTTATGTTACTTATAAT

ACGCATGGAGTGTGTCCGGAGTCTGTAATAATCTGCGCGTATCTGTTATGTC

>GXHP114

AGCCGTTGGATCTAGTTGAAAATGTCAGAGACGCGTGATGAGTATGGCAACCAGGTTCGC

CAAACCGACGAGTATGGGAACCCGATTCAGCATACTGGCACCGGGACAAAGCCCGGTTCG

GGCATACATGGTGGGGGCCATGGGATTGGCACAGGTGGTGGTGGTGGACAAGGCAAGCTC

CACCGTCCAGGCTCCGGCTCTTCCTCTGTAAGTATATTCGATGTTTCTCAAGGGTCATTT

CACTTGGTATATCGTGTTGGGATGACTAGATTAGTTGATTAACGGTTGTGATTGTAAATC

AGTGATATGGGCTTCTGTGTACCTTATTTCACAATGATAATCAATTGATACGCTGGATCG

TGATGAGCGGACCGGATGAGCCGTCCATCTTACAGGCTTTTTAAAATAGAATTATAGGGT

GGGTGGTATGAGGTCCACCAAGCCTGTAACATGGAATTACAATCATAACCGTTAATCGGT

TCATCTCATCCGCTCATCGTGATCCAGCATGTCAAACAGAGATGGGTTTATGGAGAAATG

GATGTATGTAAGCGCCAACAAATCACAAAAAAATCTGACATAATGATGGAGAGAATGCAA

GGAGTGTAAGTGAATCTACGCCGTTAAATGGATTTCATTGTAATTAATTTCGTTTTGTCA

AATATTTTGTAGGACGAGGATGATGGACAAGGTGGGCGTAGGAAGAAGGGTTTGACGGAG

AAGATCAAAGAGAAGCTGCCAGGTGGAAACAAGACGACAGGTGTCTGTCATCCAGGGACT

CAGGGTGTACAGGGGGGTCGCGAGCATGAGAAGCCAGGTTGCGGTCAGGGTGGACAGGTG

GGCCGCGAGCATGAGAAGACAGGTTTCGGTCATCCAGGAACTCAGGGTGGACAGGTGGGC

AGCGAGCAGGAGAAGAAGGGTATGATTGAGAAGATCAAGGAGAAGCTGCCAGGTCACAAG

TAGGATGTGTGTCCACCATACACGTGGAGTCTACATCTACGTGTCTTATGTTACTTATAA

TACGCATGGAGTGTGTCCGGAGTCTGTAATAATCTGCGCGTATCTGTTATGTC

>GXHP115

AGCCGTTGGATCTAGTTGAAAATGTCAGAGACGCGTGATGAGTATGGCAACCAGGTTCGC

CAAACCGACGAGTATGGGAACCCGATTCAGCATACTGGCACCGGGACAAAGCCCGGTTCG

GGCATACATGGTGGGGGCCATGGGATTGGCACAGGTGGTGGTGGTGGACAAGGCAAGCTC

CACCGTCCAGGCTCCGGCTCTTCCTCTGTAAGTATATTCGATGTTTCTCAAGGGTCATTT

CACTTGGTATATCGTGTTGGGATGACTAGATTAGTTGATTAACGGTTGTGATTGTAAATC

AGTGATATGGGCTTCTGTGTACCTTATTTCACAATGATAATCAATTGATACGCTGGATCG

TGATGAGCGGACCGGATGAGCCGTCCATCTTACAGGCTTTTTAAAATAGAATTATAGGGT

GGGTGGTATGAGGTCCACCAAGCCTGTAACATGGAATTACAATCATAACCGTTAATCGGT

TCATCTCATCCGCTCATCGTGATCCAGCATGTCAAACAGAGATGGGTTTATGGAGAAATG

GATGTATGTAAGCGCCAACAAATCACAAAAAAATCTGACATAATGATGGAGAGAATGCAA

GGAGTGTAAGTGAATCTACGCCGTTAAATGGATTTCATTGTAATTAATTTCGTTTTGTCA

AATATTTTGTAGGACGAGGATGATGGACAAGGTGGGCGTAGGAAGAAGGGTTTGACGGAG

AAGATCAAAGAGAAGCTGCCAGGTGGAAACAAGACGACAGGTGTCTGTCATCCAGGGACT

CAGGGTGTACAGGGGGGTCGCGAGCATGAGAAGCCAGGTTGCGGTCAGGGTGGACAGGTG

GGCCGCGAGCATGAGAAGACAGGTTTCGGTCATCCAGGAACTCAGGGTGGACAGGTGGGC

AGCGAGCAGGAGAAGAAGGGTATGATTGAGAAGATCAAGGAGAAGCTGCCAGGTCACAAG

TAGGATGTGTGTCCACCATACACGTGGAGTCTACATCTACGTGTCTTATGTTACTTATAA

TACGCATGGAGTGTGTCCGGAGTCTGTAATAATCTGCGCGTATCTGTTATGTC

>GXHP111

AGCCGTTGGATCTAGTTGAAAATGTCAGAGACGCGTGATGAGTATGGCAACCAGGTTCGC

CAAACCGACGAGTATGGGAACCCGATTCAGCATACTGGCACCGGGACAAAGCCCGGTTCG

GGCATACATGGTGGGGGCCATGGGATTGGCACAGGTGGTGGTGGTGGACAAGGCAAGCTC

CACCGTCCAGGCTCCGGCTCTTCCTCTGTAAGTATATTCGATGTTTCTCAAGGGTCATTT

CACTTGGTATATCGTGTTGGGATGACTAGATTAGTTGATTAACGGTTGTGATTGTAAATC

AGTGATATGGGCTTCTGTGTACCTTATTTCACAATGATAATCAATTGATACGCTGGATCG

TGATGAGCGGACCGGATGAGCCGTCCATCTTACAGGCTTTTTAAAATAGAATTATAGGGT

GGGTGGTATGAGGTCCACCAAGCCTGTAACATGGAATTACAATCATAACCGTTAATCGGT

TCATCTCATCCGCTCATCGTGATCCAGCATGTCAAACAGAGATGGGTTTATGGAGAAATG

GATGTATGTAAGCGCCAACAAATCACAAAAAAATCTGACATAATGATGGAGAGAATGCAA

GGAGTGTAAGTGAATCTACGCCGTTAAATGGATTTCATTGTAATTAATTTCGTTTTGTCA

AATATTTTGTAGGACGAGGATGATGGACAAGGTGGGCGTAGGAAGAAGGGTTTGACGGAG

AAGATCAAAGAGAAGCTGCCAGGTGGAAACAAGACGACAGGTGTCTGTCATCCAGGGACT

CAGGGTGTACAGGGGGGTCGCGAGCATGAGAAGCCAGGTTGCGGTCAGGGTGGACAGGTG

GGCCGCGAGCATGAGAAGACAGGTTTCGGTCATCCAGGAACTCAGGGTGGACAGGTGGGC

AGCGAGCAGGAGAAGAAGGGTATGATTGAGAAGATCAAGGAGAAGCTGCCAGGTCACAAG

TAGGATGTGTGTCCACCATACACGTGGAGTCTACATCTACGTGTCTTATGTTACTTATAA

TACGCATGGAGTGTGTCCGGAGTCTGTAATAATCTGCGCGTATCTGTTATGTC

>GXHP510

AGCCGTTGGATCTAGTTGAAAATGTCAGAGACGCGTGATGAGTATGGCAACCAGGTTCGC

CAAACCGACGAGTATGAGAACCCGATTCAGCATACTGGCACCGGGACAAAGCCCGGTTCG

GGCATACATGGTGGGGGCCATGGGATTGGCACAGGTGGTGGTGGTGGACAAGGCAAGCTC

CACCGTCCAGGCTCCGGCTCTTCCTCTGTAAGTATATTCGATGTTTCTCGAGGGTCATTT

CACTTGGTATATCGTGTTGGGATGACTAGATTAGTTGATTAACGGTTGTGATTGTAAATC

AGTGATATGGGCTTGTGTGTACCTTATTTCACAATGATAATCAATTGATACGCTGGATCG

TGATGAGCGGACCGGATGAGCCGTCCATCTTACAGGCTTTTTAAAATAGAATTATAGGGT

GGGTGGTATGAAGTCTACCAAGCCTGTAACATGGAATTACAATCATAGCCGTTAATCGGT

TCATCTCATTCGCTCATCGTGATCCAGCATGTCAAACAGAGATGGGTTTATGGAGAAATG

GATGTATGTAAGCCAACAAATCACAAAAAAAATCTGACATAATGATGGAGAGAATGCAAG

GAGTGTAAGTGAATCTACGCCGTTAAATGGATTTCATTGTACTTAATTTCGTTTTGTCAA

ATATTTTGTAGGACGAGGATGATGGACAAGGTGGGCGTAGGAAGAAGGGTTTGACGGAGA

AGATCAAAGAGAAGCTGCCAGGTGGAAACAAGACGACAGGTGTCTGTCATCCAGGGACTC

AGGGTGTACAGGGGGGTCGCGAGCATGAGAAGCCAGGTTGCGGTCAGGGTGGACAGGTGG

GCCGCGAGCATGAGAAGACAGGTTTCGGTCATCCAGGGACTCAGGGTGGACAGGTGGGCA

GCGAGCAGGAGAAGAAGGGTATGATTGAGAAGATCAAGGAGAAGCTGCCAGGTCACAAGT

AGGATGTGTGTCCACCATACACGTGGAGTCTGCATCTACGTGTCTTATGTTACTTATAAT

ACGCATGGAGTGTGTCCGGAGTCTGTAATAATCTGCGCGTATCTGTTATGTC

>GXHP513

AGCCGTTGGATCTAGTTGAAAATGTCAGAGACGCGTGATGAGTATGGCAACCAGGTTCGC

CAAACCGACGAGTATGGGAACCCGATTCAGCATACTGGCACCGGGACAAAGCCCGGTTCG

GGCATACATGGTGGGGGCCATGGGATTGGCACAGGTGGTGGTGGTGGACAAGGCAAGCTC

CACCGTCCAGGCTCCGGCTCTTCCTCTGTAAGTATATTCGATGTTTCTCAAGGGTCATTT

CACTTGGTATATAGTGTTGGGATGACTAGATTAGTTGATTAACGGTTGTGATTGTAAATC

AGTGATATGGGCTTCTATGTACCTTATTTCACAATGATAATCAATTGATACGCTGGATCG

TGATGAGCGGACCGGATGAGCCGTCCATCTTACAGGCTTTTTAAAATAGAATTATAGGGT

GGGTGGTATGAGGTCTACCAAGCCTGTAATATGGAATGACAATCATAGCCGTTAATCGGT

TCATCTCATCCGCTCATCGTGATCCAGCATGTCAAACAGAGATGGGTTTATGGAGAAATG

GATGTATGTAAGCCAACAAATCACAAAAAAAATCTGACATAATGATGGAGAGAATGCAAG

GAGTGTAAGTGAATCTACGCCGTTAAATGGATTTCATTGTACTTAATTTCGTTTTGTCAA

ATATTTTGTAGGACGAGGATGATGGACAAGGTGGGCGTAGGAAGAAGGGTTTGACGGAGA

AGATCAAAGAGAAGCTGCCAGGTGGAAACAAGACGACAGGTGTCTGTCATCCAGGGACTC

AGGGAGCACAGGGGGGTCGCGAGCATGAGAAGCCAGGTTGCGGTCAGGGTGGACAGGTGG

GCCGCGAGCATGAGAAGACAGGTTTCGGTCATCCAGGGACTCAGGGTGGACAGGTGGGCA

GCGAGCAGGAGAAGAAGGGTATGATTGAGAAGATCAAGGAGAAGCTGCCAGGTCACAAGT

AGGATGTGTGTCCACCATACACGTGGAGTCTACACCTACGTGTCTTATGTTACTTATAAT

ACGCATGGAGTGTGTCCGGAGTCTGTAATAATCTGCGCGTATCTGTTATGTC

>GXHP515

AGCCGTTGGATCTAGTTGAAAATGTCAGAGACGCGTGATGAGTATGGCAACCAGGTTCGC

CAAACCGACGAGTATGAGAACCCGATTCAGCATACTGGCACCGGGACAAAGCCCGGTTCG

GGCATACATGGTGGGGGCCATGGGATTGGCACAGGTGGTGGTGGTGGACAAGGCAAGCTC

CACCGTCCAGGCTCCGGCTCTTCCTCTGTAAGTATATTCGATGTTTCTCGAGGGTCATTT

CACTTGGTATATCGTGTTGGGATGACTAGATTAGTTGATTAACGGTTGTGATTGTAAATC

AGTGATATGGGCTTGTGTGTACCTTATTTCACAATGATAATCAATTGATACGCTGGATCG

TGATGAGCGGACCGGATGAGCCGTCCATCTTACAGGCTTTTTAAAATAGAATTATAGGGT

GGGTGGTATGAAGTCTACCAAGCCTGTAACATGGAATTACAATCATAGCCGTTAATCGGT

TCATCTCATTCGCTCATCGTGATCCAGCATGTCAAACAGAGATGGGTTTATGGAGAAATG

GATGTATGTAAGCCAACAAATCACAAAAAAAATCTGACATAATGATGGAGAGAATGCAAG

GAGTGTAAGTGAATCTACGCCGTTAAATGGATTTCATTGTACTTAATTTCGTTTTGTCAA

ATATTTTGTAGGACGAGGATGATGGACAAGGTGGGCGTAGGAAGAAGGGTTTGACGGAGA

AGATCAAAGAGAAGCTGCCAGGTGGAAACAAGACGACAGGTGTCTGTCATCCAGGGACTC

AGGGTGTACAGGGGGGTCGCGAGCATGAGAAGCCAGGTTGCGGTCAGGGTGGACAGGTGG

GCCGCGAGCATGAGAAGACAGGTTTCGGTCATCCAGGGACTCAGGGTGGACAGGTGGGCA

GCGAGCAGGAGAAGAAGGGTATGATTGAGAAGATCAAGGAGAAGCTGCCAGGTCACAAGT

AGGATGTGTGTCCACCATACACGTGGAGTCTGCATCTACGTGTCTTATGTTACTTATAAT

ACGCATGGAGTGTGTCCGGAGTCTGTAATAATCTGCGCGTATCTGTTATGTC

>GZYJ17322

AGCCGTTGGATCTAGTTGAAAATGTCAGAGACGCGTGATGAGTATGGCAACCAGGTTCGC

CAAACCGACGAGTATGGGAACCCGATTCAGCATACTGGCACCGGGACAAAGCCCGGTTCG

GGCATACATGGTGGGGGCCATGGGATTGGCACAGGTGGTGGTGGTGGACAAGGCAAGCTC

CACCGTCCAGGCTCCGGCTCTTCCTCTGTAAGTATATTCGATGTTTCTCAAGGGCCATTT

CACTTGGTATATCGTGTTGGGATGACTAGATTAGTTGATTAACGGTTGTGATTGTAAATC

AGTGATATGGGCTTCTGTGTACCTTATTTCACAATGATAATCAATTGATACGCTGGATCG

TGATGAGCGGACCGGATGAGCCGTCCATCTTACAGGCTTTTTAAAATAGAATTATAGGGT

GGGTGTTATGAGGTCTACCAAGCCTGTAACATGGAATTACAATCATAGCTGTTAATCGGT

TCATCTCATCCGCTCATCGTGATCCAGCATGTCAAACAGAGATGGGTTTATGGAGAAATG

GATGTATGTAAGCCAAAAAATCACAAAAAAATCTGACATAATGATGGAGAGAATGCAAGG

AGTGTAAGTGAATCTACGCCGTTAAATGGATTTCATTGTACTTAATTTCGTTTTGTCAAA

TATTTTGTAGGACGAGGATGATGGACAAGGTGGGCGTAGGAAGAAGGGTTTGACGGAGAA

GATCAAAGAGAAGCTGCCAGGTGGAAACAAGACGACAGGTGTCTGTCATCCAGGGACTCA

GGGAGTACAGGGGGGTCGCGAGCATGAGAAGCCAGGTTGCGGTCAGGGTGGACAGGTGGG

CCGCGAGCATGAGAAGACAGGTTTCGGTCATCCAGGGACTCAGGGTGGACAGGTGGGCAG

CGAGCAGGAGAAGAAGGGTATGATTGAGAAGATCAAGGAGAAGCTGCCAGGTCACAAGTA

GGATGTGTGTCCACCATACACGTGGAGTCTACACCTACGTGTCTTATGTTACTTATAATA

CGCATGGAGTGTGTCCGGAGTCTGTAATAATCTGCGCGTATCTGTTATGTC

>GZYJ17323

AGCCGTTGGATCTAGTTGAAAATGTCAGAGACGCGTGATGAGTATGGCAACCAGGTTCGC

CAAACCGACGAGTATGGGAACCCGATTCAGCATACTGGCACCGGGACAAAGCCCGGTTCG

GGCATACATGGTGGGGGCCATGGGATTGGCACAGGTGGTGGTGGTGGACAAGGCAAGCTC

CACCGTCCAGGCTCCGGCTCTTCCTCTGTAAGTATATTCGATGTTTCTCAAGGGCCATTT

CACTTGGTATATCGTGTTGGGATGACTAGATTAGTTGATTAACGGTTGTGATTGTAAATC

AGTGATATGGGCTTCTGTGTACCTTATTTCACAATGATAATCAATTGATACGCTGGATCG

TGATGAGCGGACCGGATGAGCCGTCCATCTTACAGGCTTTTTAAAATAGAATTATAGGGT

GGGTGTTATGAGGTCTACCAAGCCTGTAACATGGAATTACAATCATAGCTGTTAATCGGT

TCATCTCATCCGCTCATCGTGATCCAGCATGTCAAACAGAGATGGGTTTATGGAGAAATG

GATGTATGTAAGCCAAAAAATCACAAAAAAATCTGACATAATGATGGAGAGAATGCAAGG

AGTGTAAGTGAATCTACGCCGTTAAATGGATTTCATTGTACTTAATTTCGTTTTGTCAAA

TATTTTGTAGGACGAGGATGATGGACAAGGTGGGCGTAGGAAGAAGGGTTTGACGGAGAA

GATCAAAGAGAAGCTGCCAGGTGGAAACAAGACGACAGGTGTCTGTCATCCAGGGACTCA

GGGAGTACAGGGGGGTCGCGAGCATGAGAAGCCAGGTTGCGGTCAGGGTGGACAGGTGGG

CCGCGAGCATGAGAAGACAGGTTTCGGTCATCCAGGGACTCAGGGTGGACAGGTGGGCAG

CGAGCAGGAGAAGAAGGGTATGATTGAGAAGATCAAGGAGAAGCTGCCAGGTCACAAGTA

GGATGTGTGTCCACCATACACGTGGAGTCTACACCTACGTGTCTTATGTTACTTATAATA

CGCATGGAGTGTGTCCGGAGTCTGTAATAATCTGCGCGTATCTGTTATGTC

>GZYJ17324

AGCCGTTGGATCTAGTTGGAAATGTCAGAGACGCGTGATGAGTATGGCAACCAGGTTCGC

CAAACCGACGAGTATGGGAACCCGATTCAGCATACTGGCACCGGGACAAAGCCCGGTTCG

GGCATACATGGTGGGGGCCATGGGATTGGCACAGGTGGTGGTGGTGGACAAGGCAAGCTC

CACCGTCCAGGCTCCGGCTCTTCCTCTGTAAGTATATTCGATGTTTCTCAAGGGCCATTT

CACTTGGTATATCGTGTTGGGATGACTAGATTAGTTGATTAACGGTTGTGATTGTAAATC

AGTGATATGGGCTTCTGTGTACCTTATTTCACAATGATAATCAATTGATACGCTGGATCG

TGATGAGCGGACCGGATGAGCCGTCCATCTTACAGGCTTTTTAAAATAGAATTATAGGGT

GGGTGTTATGAGGTCTACCAAGCCTGTAACATGGAATTACAATCATAGCTGTTAATCGGT

TCATCTCATCCGCTCATCGTGATCCAGCATGTCAAACAGAGATGGGTTTATGGAGAAATG

GATGTATGTAAGCCAAAAAATCACAAAAAAATCTGACATAATGATGGAGAGAATGCAAGG

AGTGTAAGTGAATCTACGCCGTTAAATGGATTTCATTGTACTTAATTTCGTTTTGTCAAA

TATTTTGTAGGACGAGGATGATGGACAAGGTGGGCGTAGGAAGAAGGGTTTGACGGAGAA

GATCAAAGAGAAGCTGCCAGGTGGAAACAAGACGACAGGTGTCTGTCATCCAGGGACTCA

GGGAGTACAGGGGGGTCGCGAGCATGAGAAGCCAGGTTGCGGTCAGGGTGGACAGGTGGG

CCGCGAGCATGAGAAGACAGGTTTCGGTCATCCAGGGACTCAGGGTGGACAGGTGGGCAG

CGAGCAGGAGAAGAAGGGTATGATTGAGAAGATCAAGGAGAAGCTGCCAGGTCACAAGTA

GGATGTGTGTCCACCATACACGTGGAGTCTACACCTACGTGTCTTATGTTACTTATAATA

CGCATGGAGTGTGTCCGGAGTCTGTAATAATCTGCGCGTATCTGTTATGTC

>GZYJ4454

AGCCGTTGGATCTAGTTGAAAATGTCAGAGACGCGTGATGAGTATGGCAACCAGGTTCGC

CAAACCGACGAGTATGGGAACCCGATTCAGCATACTGGCACCGGGACAAAGCCCGGTTCG

GGCATACATGGTGGGGGCCATGGGATTGGCACAGGTGGTGGTGGTGGACAAGGCAAGCTC

CACCGTCCAGGCTCCGGCTCTTCCTCTGTAAGTATATTCGATGTTTCTCAAGGGTCATTT

CACTTGGTATATAGTGTTGGGATGACTAGATTAGTTGATTAACGGTTGTGATTGTAAATC

AGTGATATGGGCTTCTATGTACCTTATTTCACAATGATAATCAATTGATACGCTGGATCG

TGATGAGCGGACCGGATGAGCCGTCCATCTTACAGGCTTTTTAAAATAGAATTATAGGGT

GGGTGGTATGAGGTCTACCAAGCCTGTAATATGGAATGACAATCATAGCCGTTAATCGGT

TCATCTCATCCGCTCATCGTGATCCAGCATGTCAAACAGAGATGGGTTTATGGAGAAATG

GATGTATGTAAGCCAACAAATCACAAAAAAAATCTGACATAATGATGGAGAGAATGCAAG

GAGTGTAAGTGAATCTACGCCGTTAAATGGATTTCATTGTACTTAATTTCGTTTTGTCAA

ATATTTTGTAGGACGAGGATGATGGACAAGGTGGGCGTAGGAAGAAGGGTTTGACGGAGA

AGATCAAAGAGAAGCTGCCAGGTGGAAACAAGACGACAGGTGTCTGTCATCCAGGGACTC

AGGGTGTACAGGGGGGTCGCGAGCATGAGAAGCCAGGTTGCGGTCAGGGTGGACAGGTGG

GCCGCGAGCATGAGAAGACAGGTTTCGGTCATCCAGGAACTCAGGGTGGACAGGTGGGCA

GCGAGCAGGAGAAGAAGGGTATGATTGAGAAGATCAAGGAGAAGCTGCCAGGTCACAAGT

AGGATGTGTGTCCACCATACACGTGGAGTCTGCATCTACGTGTCTTATGTTACTTATAAT

ACGCATGGAGTGTGTCCGGAGTCTGTAATAATCTGCGCGTATCTGTTATGTC

>GZYJ4455

AGCCGTTGGATCTAGTTGAAAATGTCAGAGACGCGTGATGAGTATGGCAACCAGGTTCGC

CAAACCGACGAGTATGGGAACCCGATTCAGCATACTGGCACCGGGACAAAGCCCGGTTCG

GGCATACATGGTGGGGGCCATGGGATTGGCACAGGTGGTGGTGGTGGACAAGGCAAGCTC

CACCGTCCAGGCTCCGGCTCTTCCTCTGTAAGTATATTCGATGTTTCTCAAGGGCCATTT

CACTTGGTATATCGTGTTGGGATGACTAGATTAGTTGATTAACGGTTGTGATTGTAAATC

AGTGATATGGGCTTCTGTGTACCTTATTTCACAATGATAATCAATTGATACGCTGGATCG

TGATGAGCGGACCGGATGAGCCGTCCATCTTACAGGCTTTTTAAAATAGAATTATAGGGT

GGGTGTTATGAGGTCTACCAAGCCTGTAACATGGAATTACAATCATAGCTGTTAATCGGT

TCATCTCATCCGCTCATCGTGATCCAGCATGTCAAACAGAGATGGGTTTATGGAGAAATG

GATGTATGTAAGCCAAAAAATCACAAAAAAATCTGACATAATGATGGAGAGAATGCAAGG

AGTGTAAGTGAATCTACGCCGTTAAATGGATTTCATTGTACTTAATTTCGTTTTGTCAAA

TATTTTGTAGGACGAGGATGATGGACAAGGTGGGCGTAGGAAGAAGGGTTTGACGGAGAA

GATCAAAGAGAAGCTGCCAGGTGGAAACAAGACGACAGGTGTCTGTCATCCAGGGACTCA

GGGAGTACAGGGGGGTCGCGAGCATGAGAAGCCAGGTTGCGGTCAGGGTGGACAGGTGGG

CCGCGAGCATGAGAAGACAGGTTTCGGTCATCCAGGGACTCAGGGTGGACAGGTGGGCAG

CGAGCAGGAGAAGAAGGGTATGATTGAGAAGATCAAGGAGAAGCTGCCAGGTCACAAGTA

GGATGTGTGTCCACCATACACGTGGAGTCTACACCTACGTGTCTTATGTTACTTATAATA

CGCATGGAGTGTGTCCGGAGTCTGTAATAATCTGCGCGTATCTGTTATGTC

>GZYJ53163

AGCCGTTGGATCTAGTTGAAAATGTCAGAGACGCGTGATGAGTATGGCAACCAGGTTCGC

CAAACCGACGAGTATGGGAACCCGATTCAGCATACTGGCACCGGGACAAAGCCCGGTTCG

GGCATACATGGTGGGGGCCATGGGATTGGCACAGGTGGTGGTGGTGGACAAGGCAAGCTC

CACCGTCCAGGCTCCGGCTCTTCCTCTGTAAGTATATTCGATGTTTCTCAAGGGCCATTT

CACTTGGTATATCGTGTTGGGATGACTAGATTAGTTGATTAACGGTTGTGATTGTAAATC

AGTGATATGGGCTTCTGTGTACCTTATTTCACAATGATAATCAATTGATACGCTGGATCG

TGATGAGCGGACCGGATGAGCCGTCCATCTTACTGGCTTTTTAAAATAGAATTATAGGGT

GGGTGGTATGAGGTCTACCAAGCCCGTAACATGGAATTACAATCATAGCTGTTAATCGGT

TCATCTCATCCGCTCATCGTGATCCAGCATGTCAAACAGAGATGGGTTTCTGGAGAAATG

GATGTATGTAAGCCCACAAATCACAAAAAAAAATCTGACATAATGATGGAGAGAATGCAA

GGAGTGTAAGTGAATCTACGCCGTTAAATGGATTTCATTGTACTTAACTTCGTTTTGTCA

AATATTTTGTAGGACGAGGATGATGGACAAGGTGGGCGTAGGAAGAAGGGTTTGACGGAG

AAGATAAAAGAGAAGCTGCCAGGTGGAAACAAGACGACAGGTGTCTGTCATCCAGGGACT

CAGGGTGTACAGGGGGGTCGCGAGCATGAGAAGCCAGGTTGCGGTCAGGGTGGACAGGTG

GGCCGCGAGCATGAGAAGACAGGTTTCGGTCATCCAGGGACTCAGGGTGGACAGGTGGGC

AGCGAGCAGGAGAAGAAGGGTATGATTGAGAAGATCAAGGAGAAGCTACCAGGTCACAAG

TAGGATGTGTGTCCACCATACACGTGGAGTCTGCATCTACGTGTCTTATGTTACTTATAA

TACGCATGGAGTGTGTCCGGAGTCTGTAATAATCTGCGCGTATCTGTTATGTC

>GZYJ53164

AGCCGTTGGATCTAGTTGAAAATGTCAGAGACGCGTGATGAGTATGGCAACCAGGTTCGC

CAAACCGACGAGTATGGGAACCCGATTCAGCATACTGGCACCGGGACAAAGCCCGGTTCG

GGCATACATGGTGGGGGCCATGGGATTGGCACAGGTGGTGGTGGTGGACAAGGCAAGCTC

CACCGTCCAGGCTCCGGCTCTTCCTCTGTAAGTATATTCGATGTTTCTCAAGGGCCATTT

CACTTGGTATATCGTGTTGGGATGACTAGATTAGTTGATTAACGGTTGTGATTGTAAATC

AGTGATATGGGCTTCTGTGTACCTTATTTCACAATGATAATCAATTGATACGCTGGATCG

TGATGAGCGGACCGGATGAGCCGTCCATCTTACTGGCTTTTTAAAATAGAATTATAGGGT

GGGTGGTATGAGGTCTACCAAGCCTGTAACATGGAATTACAATCATAGCTGTTAATCGGT

TCATCTCATCCGCTCATCGTGATCCAGCATGTCAAACAGAGATGGGTTTCTGGAGAAATG

GATGTATGTAAGCCCACAAATCACAAAAAAAAATCTGACATAATGATGGAGAGAATGCAA

GGAGTGTAAGTGAATCTACGCCGTTAAATGGATTTCATTGTACTTAACTTCGTTTTGTCA

AATATTTTGTAGGACGAGGATGATGGACAAGGTGGGCGTAGGAAGAAGGGTTTGACGGAG

AAGATAAAAGAGAAGCTGCCAGGTGGAAACAAGACGACAGGTGTCTGTCATCCAGGGACT

CAGGGTGTACAGGGGGGTCGCGAGCATGAGAAGCCAGGTTGCGGTCAGGGTGGACAGGTG

GGCCGCGAGCATGAGAAGACAGGTTTCGGTCATCCAGGGACTCAGGGTGGACAGGTGGGC

AGCGAGCAGGAGAAGAAGGGTATGATTGAGAAGATCAAGGAGAAGCTACCAGGTCACAAG

TAGGATGTGTGTCCACCATACACGTGGAGTCTGCATCTACGTGTCTTATGTTACTTATAA

TACGCATGGAGTGTGTCCGGAGTCTGTAATAATCTGCGCGTATCTGTTATGTC

>GZYJ53165

AGCCGTTGGATCTAGTTGAAAATGTCAGAGACGCGTGATGAGTATGGCAACCAGGTTCGC

CAAACCGACGAGTATGGGAACCCGATTCAGCATACTGGCACCGGGACAAAGCCCGGTTCG

GGCATACATGGTGGGGGCCATGGGATTGGCACAGGTGGTGGTGGTGGACAAGGCAAGCTC

CACCGTCCAGGCTCCGGCTCTTCCTCTGTAAGTATATTCGATGTTTCTCAAGGGCCATTT

CACTTGGTATATCGTGTTGGGATGACTAGATTAGTTGATTAACGGTTGTGATTGTAAATC

AGTGATATGGGCTTCTGTGTACCTTATTTCACAATGATAATCAATTGATACGCTGGATCG

TGATGAGCGGACCGGATGAGCCGTCCATCTTACTGGCTTTTTAAAATAGAATTATAGGGT

GGGTGGTATGAGGTCTACCAAGCCTGTAACATGGAATTACAATCATAGCTGTTAATCGGT

TCATCTCATCCGCTCATCGTGATCCAGCATGTCAAACAGAGATGGGTTTCTGGAGAAATG

GATGTATGTAAGCCCACAAATCACAAAAAAAAATCTGACATAATGATGGAGAGAATGCAA

GGAGTGTAAGTGAATCTACGCCGTTAAATGGATTTCATTGTACTTAACTTCGTTTTGTCA

AATATTTTGTAGGACGAGGATGATGGACAAGGTGGGCGTAGGAAGAAGGGTTTGACGGAG

AAGATAAAAGAGAAGCTGCCAGGTGGAAACAAGACGACAGGTGTCTGTCATCCAGGGACT

CAGGGTGTACAGGGGGGTCGCGAGCATGAGAAGCCAGGTTGCGGTCAGGGTGGACAGGTG

GGCCGCGAGCATGAGAAGACAGGTTTCGGTCATCCAGGGACTCAGGGTGGACAGGTGGGC

AGCGAGCAGGAGAAGAAGGGTATGATTGAGAAGATCAAGGAGAAGCTACCAGGTCACAAG

TAGGATGTGTGTCCACCATACACGTGGAGTCTGCATCTACGTGTCTTATGTTACTTATAA

TACGCATGGAGTGTGTCCGGAGTCTGTAATAATCTGCGCGTATCTGTTATGTC

>GZYJ325412

AGCCGTTGGATCTAGTTGAAAATGTCAGAGACGCGTGATGAGTATGGCAACCAGGTTCGC

CAAACCGACGAGTATGGGAACCCGATTCAGCATACTGGCACCGGGACAAAGCCCGGTTCG

GGCATACATGGTGGGGGCCATGGGATTGGCACAGGTGGTGGTGGTGGACAAGGCAAGCTC

CACCGTCCAGGCTCCGGCTCTTCCTCTGTAAGTATATTCGATGTTTCTCAAGGGCCATTT

CACTTGGTATATCGTGTTGGGATGACTAGATTAGTTGATTAACGGTTGTGATTGTAAATC

AGTGATATGGGCTTCTGTGTACCTTATTTCACAATGATAATCAATTGATACGCTGGATCG

TGATGAGCGGACCGGATGAGCCGTCCATCTTACTGGCTTTTTAAAATAGAATTATAGGGT

GGGTGGTATGAGGTCTACCAAGCCTGTAACATGGAATTACAATCATAGCTGTTAATCGGT

TCATCTCATCCGCTCATCGTGATCCAGCATGTCAAACAGAGATGGGTTTCTGGAGAAATG

GATGTATGTAAGCCCACAAATCACAAAAAAAAATCTGACATAATGATGGAGAGAATGCAA

GGAGTGTAAGTGAATCTACGCCGTTAAATGGATTTCATTGTACTTAACTTCGTTTTGTCA

AATATTTTGTAGGACGAGGATGATGGACAAGGTGGGCGTAGGAAGAAGGGTTTGACGGAG

AAGATAAAAGAGAAGCTGCCAGGTGGAAACAAGACGACAGGTGTCTGTCATCCAGGGACT

CAGGGTGTACAGGGGGGTCGCGAGCATGAGAAGCCAGGTTGCGGTCAGGGTGGACAGGTG

GGCCGCGAGCATGAGAAGACAGGTTTCGGTCATCCAGGGACTCAGGGTGGACAGGTGGGC

AGCGAGCAGGAGAAGAAGGGTATGATTGAGAAGATCAAGGAGAAGCTACCAGGTCACAAG

TAGGATGTGTGTCCACCATACACGTGGAGTCTGCATCTACGTGTCTTATGTTACTTATAA

TACGCATGGAGTGTGTCCGGAGTCTGTAATAATCTGCGCGTATCTGTTATGTC

>GZYJ325413

AGCCGTTGGATCTAGTTGAAAATGTCAGAGACGCGTGATGAGTATGGCAACCAGGTTCGC

CAAACCGACGAGTATGGGAACCCGATTCAGCATACTGGCACCGGGACAAAGCCCGGTTCG

GGCATACATGGTGGGGGCCATGGGATTGGCACAGGTGGTGGTGGTGGACAAGGCAAGCTC

CACCGTCCAGGCTCCGGCTCTTCCTCTGTAAGTATATTCGATGTTTCTCAAGGGCCATTT

CACTTGGTATATCGTGTTGGGATGACTAGATTAGTTGATTAACGGTTGTGATTGTAAATC

AGTGATATGGGCTTCTGTGTACCTTATTTCACAATGATAATCAATTGATACGCTGGATCG

TGATGAGCGGACCGGATGAGCCGTCCATCTTACTGGCTTTTTAAAATAGAATTATAGGGT

GGGTGGTATGAGGTCTACCAAGCCTGTAACATGGAATTACAATCATAGCTGTTAATCGGT

TCATCTCATCCGCTCATCGTGATCCAGCATGTCAAACAGAGATGGGTTTCTGGAGAAATG

GATGTATGTAAGCCCACAAATCACAAAAAAAAATCTGACATAATGATGGAGAGAATGCAA

GGAGTGTAAGTGAATCTACGCCGTTAAATGGATTTCATTGTACTTAACTTCGTTTTGTCA

AATATTTTGTAGGACGAGGATGATGGACAAGGTGGGCGTAGGAAGAAGGGTTTGACGGAG

AAGATAAAAGAGAAGCTGCCAGGTGGAAACAAGACGACAGGTGTCTGTCATCCAGGGACT

CAGGGTGTACAGGGGGGTCGCGAGCATGAGAAGCCAGGTTGCGGTCAGGGTGGACAGGTG

GGCCGCGAGCATGAGAAGACAGGTTTCGGTCATCCAGGGACTCAGGGTGGACAGGTGGGC

AGCGAGCAGGAGAAGAAGGGTATGATTGAGAAGATCAAGGAGAAGCTACCAGGTCACAAG

TAGGATGTGTGTCCACCATACACGTGGAGTCTGCATCTACGTGTCTTATGTTACTTATAA

TACGCATGGAGTGTGTCCGGAGTCTGTAATAATCTGCGCGTATCTGTTATGTC

>GZYJ325415

AGCCGTTGGATCTAGTTGAAAATGTCAGAGACGCGTGATGAGTATGGCAACCAGGTTCGC

CAAACCGACGAGTATGGGAACCCGATTCAGCATACTGGCACCGGGACAAAGCCCGGTTCG

GGCATACATGGTGGGGGCCATGGGATTGGCACAGGTGGTGGTGGTGGACAAGGCAAGCTC

CACCGTCCAGGCTCCGGCTCTTCCTCTGTAAGTATATTCGATGTTTCTCAAGGGCCATTT

CACTTGGTATATCGTGTTGGGATGACTAGATTAGTTGATTAACGGTTGTGATTGTAAATC

AGTGATATGGGCTTCTGTGTACCTTATTTCACAATGATAATCAATTGATACGCTGGATCG

TGATGAGCGGACCGGATGAGCCGTCCATCTTACAGGCTTTTTAAAATAGAATTATAGGGT

GGGTGTTATGAGGTCTACCAAGCCTGTAACATGGAATTACAATCATAGCTGTTAATCGGT

TCATCTCATCCGCTCATCGTGATCCAGCATGTCAAACAGAGATGGGTTTATGGAGAAATG

GATGTATGTAAGCCAAAAAATCACAAAAAAATCTGACATAATGATGGAGAGAATGCAAGG

AGTGTAAGTGAATCTACGCCGTTAAATGGATTTCATTGTACTTAATTTCGTTTTGTCAAA

TATTTTGTAGGACGAGGATGATGGACAAGGTGGGCGTAGGAAGAAGGGTTTGACGGAGAA

GATCAAAGAGAAGCTGCCAGGTGGAAACAAGACGACAGGTGTCTGTCATCCAGGGACTCA

GGGAGTACAGGGGGGTCGCGAGCATGAGAAGCCAGGTTGCGGTCAGGGTGGACAGGTGGG

CCGCGAGCATGAGAAGACAGGTTTCGGTCATCCAGGGACTCAGGGTGGACAGGTGGGCAG

CGAGCAGGAGAAGAAGGGTATGATTGAGAAGATCAAGGAGAAGCTGCCAGGTCACAAGTA

GGATGTGTGTCCACCATACACGTGGAGTCTACACCTACGTGTCTTATGTTACTTATAATA

CGCATGGAGTGTGTCCGGAGTCTGTAATAATCTGCGCGTATCTGTTATGTC

>GZST64

AGCCGTTGGATCTAGTTGAAAATGTCAGAGACGCGTGATGAGTATGGCAACCAGGTTCGC

CAAACCGACGAGTATGGGAACCCGATTCAGCATACTGGCACCGGGACAAAGCCCGGTTCG

GGCATACATGGTGGGGGCCATGGGATTGGCACAGGTGGTGGTGGTGGACAAGGCAAGCTC

CACCGTCCAGGCTCCGGCTCTTCCTCTGTAAGTATATTCGATGTTTCTCAAGGGTCATTT

CACTTGGTATATAGTGTTGGGATGACTAGATTAGTTGATTAACGGTTGTGATTGTAAATC

AGTGATATGGGCTTCTATGTACCTTATTTCACAATGATAATCAATTGATACGCTGGATCG

TGATGAGCGGACCGGATGAGCCGTCCATCTTACAGGCTTTTTAAAATAGAATTATAGGGT

GGGTGGTATGAGGTCTACCAAGCCTGTAACATGGAATTACAATCATAGCCGTTAATCCGT

TCATCTCATCCGCTCATCGTGATCCAGCATGTCAAACAGAGATGGGTTTATGGAGAAATG

GATGTATGTAAGCCAACAAATCACAAAAAAAATCTGACATAATGATGGAGAGAATGCAAG

GAGTGTAAGTGAATCTACGCCGTTAAATGGATTTCATTGTACTTAATTTCGTTTTGTCAA

ATATTTTGTAGGACGAGGATGATGGACAAGGTGGGCGTAGGAAGAAGGGTTTGACGGAGA

AGATCAAAGAGAAGCTGCCAGGTGGAAACAAGACGACAGGTGTCTGTCATCCAGGGACTC

AGGGTGTACAGGGGGGTCGCGAGCATGAGAAGCCAGGTTGCGGTCAGGGTGGACAGGTGG

GCCGCGAGCATGAGAAGACAGGTTTCGGTCATCCAGGGACTCAGGGTGGACAGGTGGGCA

GCGAGCAGGAGAAGAAGGGTATGATTGAGAAGATCAAGGAGAAGCTGCCAGGTCACAAGT

AGGATGTGTGTCCACCATACACGTGGAGTCTACACCTACGTGTCTTATGTTACTTATAAT

ACGCATGGAGTGTGTCCGGAGTCTGTAATAATCTGCGCGTATCTGTTATGTC

>GZST66

AGCCGTTGGATCTAGTTGAAAATGTCAGAGACGCGTGATGAGTATGGCAACCAGGTTCGC

CAAACCGACGAGTATGGGAACCCGATTCAGCATACTGGCACCGGGACAAAGCCCGGTTCG

GGCATACATGGTGGGGGCCATGGGATTGGCACAGGTGGTGGTGGTGGACAAGGCAAGCTC

CACCGTCCAGGCTCCGGCTCTTCCTCTGTAAGTATATTCGATGTTTCTCAAGGGTCATTT

CACTTGGTATATAGTGTTGGGATGACTAGATTAGTTGATTAACGGTTGTGATTGTAAATC

AGTGATATGGGCTTCTATGTACCTTATTTCACAATGATAATCAATTGATACGCTGGATCG

TGATGAGCGGACCGGATGAGCCGTCCATCTTACAGGCTTTTTAAAATAGAATTATAGGGT

GGGTGGTATGAGGTCTACCAAGCCTGTAACATGGAATTACAATCATAGCCGTTAATCGGT

TCATCTCATCCGCTCATCGTGATCCAGCATGTCAAACAGAGATGGGTTTATGGAGAAATG

GATGTATGTAAGCCAACAAATCACAAAAAAAATCTGACATAATGATGGAGAGAATGCAAG

GAGTGTAAGTGAATCTACGCCGTTAAATGGATTTCATTGTACTTAATTTCGTTTTGTCAA

ATATTTTGTAGGACGAGGATGATGGACAAGGTGGGCGTAGGAAGAAGGGTTTGACGGAGA

AGATCAAAGAGAAGCTGCCAGGTGGAAACAAGACGACAGGTGTCTGTCATCCAGGGACTC

AGGGTGTACAGGGGGGTCGCGAGCATGAGAAGCCAGGTTGCGGTCAGGGTGGACAGGTGG

GCCGCGAGCATGAGAAGACAGGTTTCGGTCATCCAGGGACTCAGGGTGGACAGGTGGGCA

GCGAGCAGGAGAAGAAGGGTATGATTGAGAAGATCAAGGAGAAGCTACCAGGTCACAAGT

AGGATGTGTGTCCACCATACACGTGGAGTCTGCATCTACGTGTCTTATGTTACTTATAAT

ACGCATGGAGTGTGTCCGGAGTCTGTAATAATCTGCGCGTATCTGTTATGTC

>GZST624

AGCCGTTGGATCTAGTTGAAAATGTCAGAGACGCGTGATGAGTATGGCAACCAGGTTCGC

CAAACCGACGAGTATGGGAACCCGATTCAGCATACTGGCACCGGGACAAAGCCCGGTTCG

GGCATACATGGTGGGGGCCATGGGATTGGCACAGGTGGTGGTGGTGGACAAGGCAAGCTC

CACCGTCCAGGCTCCGGCTCTTCCTCTGTAAGTATATTCGATGTTTCTCAAGGGTCATTT

CACTTGGTATATAGTGTTGGGATGACTAGATTAGTTGATTAACGGTTGTGATTGTAAATC

AGTGATATGGGCTTCTATGTACCTTATTTCACAATGATAATCAATTGATACGCTGGATCG

TGATGAGCGGACCGGATGAGCCGTCCATCTTACAGGCTTTTTAAAATAGAATTATAGGGT

GGGTGGTATGAGGTCTACCAAGCCTGTAACATGGAATTACAATCATAGCCGTTAATCCGT

TCATCTCATCCGCTCATCGTGATCCAGCATGTCAAACAGAGATGGGTTTATGGAGAAATG

GATGTATGTAAGCCAACAAATCACAAAAAAAATCTGACATAATGATGGAGAGAATGCAAG

GAGTGTAAGTGAATCTACGCCGTTAAATGGATTTCATTGTACTTAATTTCGTTTTGTCAA

ATATTTTGTAGGACGAGGATGATGGACAAGGTGGGCGTAGGAAGAAGGGTTTGACGGAGA

AGATCAAAGAGAAGCTGCCAGGTGGAAACAAGACGACAGGTGTCTGTCATCCAGGGACTC

AGGGTGTACAGGGGGGTCGCGAGCATGAGAAGCCAGGTTGCGGTCAGGGTGGACAGGTGG

GCCGCGAGCATGAGAAGACAGGTTTCGGTCATCCAGGGACTCAGGGTGGACAGGTGGGCA

GCGAGCAGGAGAAGAAGGGTATGATTGAGAAGATCAAGGAGAAGCTGCCAGGTCACAAGT

AGGATGTGTGTCCACCATACACGTGGAGTCTACACCTACGTGTCTTATGTTACTTATAAT

ACGCATGGAGTGTGTCCGGAGTCTGTAATAATCTGCGCGTATCTGTTATGTC

>GZST910

AGCCGTTGGATCTAGTTGAAAATGTCAGAGACGCGTGATGAGTATGGCAACCAGGTTCGC

CAAACCGACGAGTATGGGAACCCGATTCAGCATACTGGCACCGGGACAAAGCCCGGTTCG

GGCATACATGGTGGGGGCCATGGGATTGGCACAGGTGGTGGTGGTGGACAAGGCAAGCTC

CACCGTCCAGGCTCCGGCTCTTCCTCTGTAAGTATATTCGATGTTTCTCAAGGGTCATTT

CACTTGGTATATAGTGTTGGGATGACTAGATTAGTTGATTAACGGTTGTGATTGTAAATC

AGTGATATGGGCTTCTATGTACCTTATTTCACAATGATAATCAATTGATACGCTGGATCG

TGATGAGCGGACCGGATGAGCCGTCCATCTTACAGGCTTTTTAAAATAGAATTATAGGGT

GGGTGGTATGAGGTCTACCAAGCCTGTAACATGGAATTACAATCATAGCCGTTAATCGGT

TCATCTCATCCGCTCATCGTGATCCAGCATGTCAAACAGAGATGGGTTTATGGAGAAATG

GATGTATGTAAGCCAACAAATCACAAAAAAAATCTGACATAATGATGGAGAGAATGCAAG

GAGTGTAAGTGAATCTACGCCGTTAAATGGATTTCATTGTACTTAATTTCGTTTTGTCAA

ATATTTTGTAGGACGAGGATGATGGACAAGGTGGGCGTAGGAAGAAGGGTTTGACGGAGA

AGATCAAAGAGAAGCTGCCAGGTGGAAACAAGACGACAGGTGTCTGTCATCCAGGGACTC

AGGGTGTACAGGGGGGTCGCGAGCATGAGAAGCCAGGTTGCGGTCAGGGTGGACAGGTGG

GCCGCGAGCATGAGAAGACAGGTTTCGGTCATCCAGGGACTCAGGGTGGACAGGTGGGCA

GCGAGCAGGAGAAGAAGGGTATGATTGAGAAGATCAAGGAGAAGCTACCAGGTCACAAGT

AGGATGTGTGTCCACCATACACGTGGAGTCTGCATCTACGTGTCTTATGTTACTTATAAT

ACGCATGGAGTGTGTCCGGAGTCTGTAATAATCTGCGCGTATCTGTTATGTC

>GZST911

AGCCGTTGGATCTAGTTGAAAATGTCAGAGACGCGTGATGAGTATGGCAACCAGGTTCGC

CAAACCGACGAGTATGGGAACCCGATTCAGCATACTGGCACCGGGACAAAGCCCGGTTCG

GGCATACATGGTGGGGGCCATGGGATTGGCACAGGTGGTGGTGGTGGACAAGGCAAGCTC

CACCGTCCAGGCTCCGGCTCTTCCTCTGTAAGTATATTCGATGTTTCTCAAGGGTCATTT

CACTTGGTATATAGTGTTGGGATGACTAGATTAGTTGATTAACGGTTGTGATTGTAAATC

AGTGATATGGGCTTCTATGTACCTTATTTCACAATGATAATCAATTGATACGCTGGATCG

TGATGAGCGGACCGGATGAGCCGTCCATCTTACAGGCTTTTTAAAATAGAATTATAGGGT

GGGTGGTATGAGGTCTACCAAGCCTGTAACATGGAATTACAATCATAGCCGTTAATCGGT

TCATCTCATCCGCTCATCGTGATCCAGCATGTCAAACAGAGATGGGTTTATGGAGAAATG

GATGTATGTAAGCCAACAAATCACAAAAAAAATCTGACATAATGATGGAGAGAATGCAAG

GAGTGTAAGTGAATCTACGCCGTTAAATGGATTTCATTGTACTTAATTTCGTTTTGTCAA

ATATTTTGTAGGACGAGGATGATGGACAAGGTGGGCGTAGGAAGAAGGGTTTGACGGAGA

AGATCAAAGAGAAGCTGCCAGGTGGAAACAAGACGACAGGTGTCTGTCATCCAGGGACTC

AGGGTGTACAGGGGGGTCGCGAGCATGAGAAGCCAGGTTGCGGTCAGGGTGGACAGGTGG

GCCGCGAGCATGAGAAGACAGGTTTCGGTCATCCAGGGACTCAGGGTGGACAGGTGGGCA

GCGAGCAGGAGAAGAAGGGTATGATTGAGAAGATCAAGGAGAAGCTACCAGGTCACAAGT

AGGATGTGTGTCCACCATACACGTGGAGTCTGCATCTACGTGTCTTATGTTACTTATAAT

ACGCATGGAGTGTGTCCGGAGTCTGTAATAATCTGCGCGTATCTGTTATGTC

>GZST916

AGCCGTTGGATCTAGTTGAAAATGTCAGAGACGCGTGATGAGTATGGCAACCAGGTTCGC

CAAACCGACGAGTATGGGAACCCGATTCAGCATACTGGCACCGGGACAAAGCCCGGTTCG

GGCATACATGGTGGGGGCCATGGGATTGGCACAGGTGGTGGTGGTGGACAAGGCAAGCTC

CACCGTCCAGGCTCCGGCTCTTCCTCTGTAAGTATATTCGATGTTTCTCAAGGGTCATTT

CACTTGGTATATAGTGTTGGGATGACTAGATTAGTTGATTAACGGTTGTGATTGTAAATC

AGTGATATGGGCTTCTATGTACCTTATTTCACAATGATAATCAATTGATACGCTGGATCG

TGATGAGCGGACCGGATGAGCCGTCCATCTTACAGGCTTTTTAAAATAGAATTATAGGGT

GGGTGGTATGAGGTCTACCAAGCCTGTAACATGGAATTACAATCATAGCCGTTAATCGGT

TCATCTCATCCGCTCATCGTGATCCAGCATGTCAAACAGAGATGGGTTTATGGAGAAATG

GATGTATGTAAGCCAACAAATCACAAAAAAAATCTGACATAATGATGGAGAGAATGCAAG

GAGTGTAAGTGAATCTACGCCGTTAAATGGATTTCATTGTACTTAATTTCGTTTTGTCAA

ATATTTTGTAGGACGAGGATGATGGACAAGGTGGGCGTAGGAAGAAGGGTTTGACGGAGA

AGATCAAAGAGAAGCTGCCAGGTGGAAACAAGACGACAGGTGTCTGTCATCCAGGGACTC

AGGGTGTACAGGGGGGTCGCGAGCATGAGAAGCCAGGTTGCGGTCAGGGTGGACAGGTGG

GCCGCGAGCATGAGAAGACAGGTTTCGGTCATCCAGGGACTCAGGGTGGACAGGTGGGCA

GCGAGCAGGAGAAGAAGGGTATGATTGAGAAGATCAAGGAGAAGCTACCAGGTCACAAGT

AGGATGTGTGTCCACCATACACGTGGAGTCTGCATCTACGTGTCTTATGTTACTTATAAT

ACGCATGGAGTGTGTCCGGAGTCTGTAATAATCTGCGCGTATCTGTTATGTC

>GZST138

AGCCGTTGGATCTAGTTGAAAATGTCAGAGACGCGTGATGAGTATGGCAACCAGGTTCGC

CAAACCGACGAGTATGGGAACCCGATTCAGCATACTGGCACCGGGACAAAGCCCGGTTCG

GGCATACATGGTGGGGGCCATGGGATTGGCACAGGTGGTGGTGGTGGACAAGGCAAGCTC

CACCGTCCAGGCTCCGGCTCTTCCTCTGTAAGTATATTCGATGTTTCTCAAGGGTCATTT

CACTTGGTATATATTGTTGGGATGACTAGATTAGTTGATTAACGGTTGTGATTGTAAATC

AGTGATATGGGCTTCTATGTACCTTATTTCACAATGATAATCAATTGATACGCTGGATCG

TGATGAGCGGACCGGATGAGCCGTCCATCTTACAGGCTTTTTAAAATAGAATTATAGGGT

GGGTGGTATGAGGTCTACCAAGCCTGTAACATGGAATTACAATCATAGCCGTTAATCCGT

TCATCTCATCCGCTCATCGTGATCCAGCATGTCAAACAGAGATGGGTTTATGGAGAAATG

GATGTATGTAAGCCAACAAATCACAAAAAAAATCTGACATAATGATGGAGAGAATGCAAG

GAGTGTAAGTGAATCTACGCCGTTAAATGGATTTCATTGTACTTAATTTCGTTTTGTCAA

ATATTTTGTAGGACGAGGATGATGGACAAGGTGGGCGTAGGAAGAAGGGTTTGACGGAGA

AGATCAAAGAGAAGCTGCCAGGTGGAAACAAGACGACAGGTGTCTGTCATCCAGGGACTC

AGGGTGTACAGGGGGGTCGCGAGCATGAGAAGCCAGGTTGCGGTCAGGGTGGACAGGTGG

GCCGCGAGCATGAGAAGACAGGTTTCGGTCATCCAGGGACTCAGGGTGGACAGGTGGGCA

GCGAGCAGGAGAAGAAGGGTATGATTGAGAAGATCAAGGAGAAGCTGCCAGGTCACAAGT

AGGATGTGTGTCCACCATACACGTGGAGTCTACACCTACGTGTCTTATGTTACTTATAAT

ACGCATGGAGTGTGTCCGGAGTCTGTAATAATCTGCGCGTATCTGTTATGTC

>GZST136

AGCCGTTGGATCTAGTTGAAAATGTCAGAGACGCGTGATGAGTATGGCAACCAGGTTCGC

CAAACCGACGAGTATGGGAACCCGATTCAGCATACTGGCACCGGGACAAAGCCCGGTTCG

GGCATACATGGTGGGGGCCATGGGATTGGCACAGGTGGTGGTGGTGGACAAGGCAAGCTC

CACCGTCCAGGCTCCGGCTCTTCCTCTGTAAGTATATTCGATGTTTCTCAAGGGTCATTT

CACTTGGTATATATTGTTGGGATGACTAGATTAGTTGATTAACGGTTGTGATTGTAAATC

AGTGATATGGGCTTCTATGTACCTTATTTCACAATGATAATCAATTGATACGCTGGATCG

TGATGAGCGGACCGGATGAGCCGTCCATCTTACAGGCTTTTTAAAATAGAATTATAGGGT

GGGTGGTATGAGGTCTACCAAGCCTGTAACATGGAATTACAATCATAGCCGTTAATCCGT

TCATCTCATCCGCTCATCGTGATCCAGCATGTCAAACAGAGATGGGTTTATGGAGAAATG

GATGTATGTAAGCCAACAAATCACAAAAAAAATCTGACATAATGATGGAGAGAATGCAAG

GAGTGTAAGTGAATCTACGCCGTTAAATGGATTTCATTGTACTTAATTTCGTTTTGTCAA

ATATTTTGTAGGACGAGGATGATGGACAAGGTGGGCGTAGGAAGAAGGGTTTGACGGAGA

AGATCAAAGAGAAGCTGCCAGGTGGAAACAAGACGACAGGTGTCTGTCATCCAGGGACTC

AGGGTGTACAGGGGGGTCGCGAGCATGAGAAGCCAGGTTGCGGTCAGGGTGGACAGGTGG

GCCGCGAGCATGAGAAGACAGGTTTCGGTCATCCAGGGACTCAGGGTGGACAGGTGGGCA

GCGAGCAGGAGAAGAAGGGTATGATTGAGAAGATCAAGGAGAAGCTGCCAGGTCACAAGT

AGGATGTGTGTCCACCATACACGTGGAGTCTACACCTACGTGTCTTATGTTACTTATAAT

ACGCATGGAGTGTGTCTGGAGTCTGTAATAATCTGCGCGTATCTGTTATGTC

>GZST139

AGCCGTTGGATCTAGTTGAAAATGTCAGAGACGCGTGATGAGTATGGCAACCAGGTTCGC

CAAACCGACGAGTATGGGAACCCGATTCAGCATACTGGCACCGGGACAAAGCCCGGTTCG

GGCATACATGGTGGGGGCCATGGGATTGGCACAGGTGGTGGTGGTGGACAAGGCAAGCTC

CACCGTCCAGGCTCCGGCTCTTCCTCTGTAAGTATATTCGATGTTTCTCAAGGGTCATTT

CACTTGGTATATATTGTTGGGATGACTAGATTAGTTGATTAACGGTTGTGATTGTAAATC

AGTGATATGGGCTTCTATGTACCTTATTTCACAATGATAATCAATTGATACGCTGGATCG

TGATGAGCGGACCGGATGAGCCGTCCATCTTACAGGCTTTTTAAAATAGAATTATAGGGT

GGGTGGTATGAGGTCTACCAAGCCTGTAACATGGAATTACAATCATAGCCGTTAATCCGT

TCATCTCATCCGCTCATCGTGATCCAGCATGTCAAACAGAGATGGGTTTATGGAGAAATG

GATGTATGTAAGCCAACAAATCACAAAAAAAATCTGACATAATGATGGAGAGAATGCAAG

GAGTGTAAGTGAATCTACGCCGTTAAATGGATTTCATTGTACTTAATTTCGTTTTGTCAA

ATATTTTGTAGGACGAGGATGATGGACAAGGTGGGCGTAGGAAGAAGGGTTTGACGGAGA

AGATCAAAGAGAAGCTGCCAGGTGGAAACAAGACGACAGGTGTCTGTCATCCAGGGACTC

AGGGTGTACAGGGGGGTCGCGAGCATGAGAAGCCAGGTTGCGGTCAGGGTGGACAGGTGG

GCCGCGAGCATGAGAAGACAGGTTTCGGTCATCCAGGGACTCAGGGTGGACAGGTGGGCA

GCGAGCAGGAGAAGAAGGGTATGATTGAGAAGATCAAGGAGAAGCTGCCAGGTCACAAGT

AGGATGTGTGTCCACCATACACGTGGAGTCTACACCTACGTGTCTTATGTTACTTATAAT

ACGCATGGAGTGTGTCCGGAGTCTGTAATAATCTGCGCGTATCTGTTATGTC

>GZXS23

AGCCGTTGGATCTAGTTGAAAATGTCAGAGACGCGTGATGAGTATGGCAACCAGGTTCGC

CAAACCGACGAGTATGGGAACCCGATTCAGCATAGTGGCACCGGGACAAAGCCCGGTTCG

GGCATACATGGTGGGGGCCATGGGATTGGCACAGGTGGTGGTGGTGGACAAGGCAAGCTC

CACCGTCCAGGCTCCGGCTCTTCCTCTGTAAGTATATTCGATGTTTCTCAAGGGTCATTT

CACTTGGTATATCGTGTTGGGATGACTAGATTAGTTGATTAACGGTTGTGATTGTAAATC

AGTGATATGGGCTTCTGTGTACCTTATTTCACAATGATAATCAATTGATACGCTGGATCG

TGATGAGCGGACCGGATGAGCCGTCCATCTTACAGGCTTTTTAAAATAGAATTATAGGGT

GGGTGGTATGAGGTCTACCAAGCCTGTAACATGGAATTACAATCATAGCCGTTAATCGGT

TCATCTCATCCGCTCATCGTGATCCAGCATGTCAAACAGAGATGGGTTTATGGAGAAATG

GATGTATGTAAGCCAACAAATCACAAAAAAAATCTGACATAATGATGGAGAGAATGCAAG

GAGTGTAAGATCTACGCCGTTAAATGGATTTCATTGTACTTAATTTCATTTTGTCAAATA

TTTTGTAGGACGAGGATGATGGACAAGGTGGGCGTAGGAAGAAGGGTTTGACGGAGAAGA

TCAAAGAGAAGCTGCCAGGTGGAAACAAGACGACAGGTGTCTGTCATCCAGGGACTCAGG

GTGTACAGGGGGGTCGCGAGCATGAGAAGCCAGGTTGCGGTCAGGGTGGACAGGTGGGCC

GCGAGCATGAGAAGACAGGTTTCGGTCATCCAGGGACTCAGGGTGGACAGGTGGGCAGCG

AGCAGGAGAAGAAGGGTATGATTGAGAAGATCAAGGAGAAGCTGCCAGGTCACAAGTAGG

ATGTGTGTCCACCATACACGTGGAGTCTACACCTACGTGTCTTATGTTACTTATAATACG

CATGGAGTGTGTCCGGAGTCTGTAATAATCTGCGCGTATCTGTTATGTC

>GZXS24

AGCCGTTGGATCTAGTTGAAAATGTCAGAGACGCGTGATGAGTATGGCAACCAGGTTCGC

CAAACCGACGAGTATGGGAACCCGATTCAGCATAGTGGCACCGGGACAAAGCCCGGTTCG

GGCATACATGGTGGGGCCCATGGGATTGGCACAGGTGGTGGTGGTGGACAAGGCAAGCTC

CACCGTCCAGGCTCCGGCTCTTCCTCTGTAAGTATATTCGATGTTTCTCAAGGGTCATTT

CACTTGGTATATAGTGTTGGGATGACTAGATTAGTTGATTAACGGTTGTGATTGTAAATC

AGTGATATGGGCTTCTATGTACCTTATTTCACAATGATAATCAATTGATACGCTGGATCG

TGATGAGCGGACCGGATGAGCCGTCCATCTTACAGGCTTTTTAAAATAGAATTATAGGGT

GGGTGGTATGAGGTCTACCAAGCCTGTAACATGGAATTACAATCATAGCCGTTAATCGGT

TCATCTCATCCGCTCATCGTGATCCAGCATGTCAAACAGAGATGGGTTTATGGAGAAATG

GATGTATGTAAGCCAACAAATCACAAAAAAAATCTGACATAATGATGGAGAGAATGCAAG

GAGTGTAAGATCTACGCCGTTAAATGGATTTCATTGTACTTAATTTCATTTTGTCAAATA

TTTTGTAGGACGAGGATGATGGACAAGGTGGGCGTAGGAAGAAGGGTTTGACGGAGAAGA

TCAAAGAGAAGCTGCCAGGTGGAAACAAGACGACAGGTGTCTGTCATCCAGGGACTCAGG

GTGTACAGGGGGGTCGCGAGCATGAGAAGCCAGGTTGCGGTCAGGGTGGACAGGTGGGCC

GCGAGCATGAGAAGACAGGTTTCGGTCATCCAGGGACTCAGGGTGGACAGGTGGGCAGCG

AGCAGGAGAAGAAGGGTATGATTGAGAAGATCAAGGAGAAGCTGCCAGGTCACAAGTAGG

ATGTGTGTCCACCATACACGTGGAGTCTACACCTACGTGTCTTATGTTACTTATAATACG

CATGGAGTGTGTCCGGAGTCTGTAATAATCTGCGCGTATCTGTTATGTC

>GZXS22

AGCCGTTGGATCTAGTTGAAAATGTCAGAGACGCGTGATGAGTATGGCAACCAGGTTCGC

CAAACCGACGAGTATGGGAACCCGATTCAGCATAGTGGCACCGGGACAAAGCCCGGTTCG

GGCATACATGGTGGGGCCCATGGGATTGGCACAGGTGGTGGTGGTGGACAAGGCAAGCTC

CACCGTCCAGGCTCCGGCTCTTCCTCTGTAAGTATATTCGATGTTTCTCAAGGGTCATTT

CACTTGGTATATAGTGTTGGGATGACTAGATTAGTTGATTAACGGTTGTGATTGTAAATC

AGTGATATGGGCTTCTATGTACCTTATTTCACAATGATAATCAATTGATACGCTGGATCG

TGATGAGCGGACCGGATGAGCCGTCCATCTTACAGGCTTTTTAAAATAGAATTATAGGGT

GGGTGGTATGAGGTCTACCAAGCCTGTAACATGGAATTACAATCATAGCCGTTAATCGGT

TCATCTCATCCGCTCATCGTGATCCAGCATGTCAAACAGAGATGGGTTTATGGAGAAATG

GATGTATGTAAGCCAACAAATCACAAAAAAAATCTGACATAATGATGGAGAGAATGCAAG

GAGTGTAAGATCTACGCCGTTAAATGGATTTCATTGTACTTAATTTCATTTTGTCAAATA

TTTTGTAGGACGAGGATGATGGACAAGGTGGGCGTAGGAAGAAGGGTTTGACGGAGAAGA

TCAAAGAGAAGCTGCCAGGTGGAAACAAGACGACAGGTGTCTGTCATCCAGGGACTCAGG

GTGTACAGGGGGGTCGCGAGCATGAGAAGCCAGGTTGCGGTCAGGGTGGACAGGTGGGCC

GCGAGCATGAGAAGACAGGTTTCGGTCATCCAGGGACTCAGGGTGGACAGGTGGGCAGCG

AGCAGGAGAAGAAGGGTATGATTGAGAAGATCAAGGAGAAGCTGCCAGGTCACAAGTAGG

ATGTGTGTCCACCATACACGTGGAGTCTACACCTACGTGTCTTATGTTACTTATAATACG

CATGGAGTGTGTCCGGAGTCTGTAATAATCTGCGCGTATCTGTTATGTC

>GZXS56

AGCCGTTGGATCTAGTTGAAAATGTCAGAGACGCGTGATGAGTATGGCAACCAGGTTCGC

CAAACCGACGAGTATGGGAACCCGATTCAGCATACTGGCACCGGGACAAAGCCCGGTTCG

GGCATACATGGTGGGGGCCATGGGATTGGCACAGGTGGTGGTGGTGGACAAGGCAAGCTC

CACCGTTCAGGCTCCGGCTCTTCCTCTGTAAGTATATTCGATGTTTCTCAAGGGCCATTT

CACTTGGTATATCGTGTTGGGATGACTAGATTAGTTGATTAACGGTTGTGACTGTAAATC

AGTGATATGGGCTTCTATGTACCTTATTTCACAATGATAATCAATTGATACGCTGGATCG

TGATGAGCGGACCGGATGAGCCGTCCATCTTACAGGCTTTTTAAAATAGAATTATAGGGT

GGGTGGTATGAGGTCCACCAAAGCCTGTAACATGGAATTACAATCATAACCGTTAATCGG

TTCATCTCATCCGCTCATCGTGATCCAGCATGTCAAACAGAGATGGGTTTATGGAGAAAT

GGATGTATGTAAGCGCCAACAAATCACAAAAAAATCTGACATAATGATGGAGAGAATGCA

AGGAGTGTAAGTGAATCTACGCCGTTAAATGGATTTCATTGTACTTAATTTCGTTTTGTC

AAATATTTTGTAGGACGAGGATGATGGACAAGGTGGGCGTAGGAAGAAGGGTTTGACGGA

GAAGATCAAAGAGAAGCTGCCAGGTGGAAACAAGACGACAGGTGTCTGTCATCCAGGGAC

TCAGGGAGTACAGGGGGGTCGCGAGCATGAGAAGCCAGGTTGCGGTCAGGGTGGACAGGT

GGGCCGCGAGCATGAGAAGACAGGTTTCGGTCATCCAGGGACTCAGGGTGGACAGGTGGG

CAGCGAGCAGGAGAAGAAGGGTATGATTGAGAAGATCAAGGAGAAGCTGCCAGGTCACAA

GTAGGATGTGTGTCCACCATACACGTGGAGTCTACACCTACGTGTCTTATGTTACTTATA

ATACGCATGGAGTGTGTCCGGAGTCTGTAATAATCTGCGCGTATCTGTTATGTC

>GZXS520

AGCCGTTGGATCTAGTTGAAAATGTCAGAGACGCGTGATGAGTATGGCAACCAGGTTCGC

CAAACCGACGAGTATGGGAACCCGATTCAGCATACTGGCACCGGGACAAAGCCCGGTTCG

GGCATACATGGTGGGGGCCATGGGATTGGCACAGGTGGTGGTGGTGGACAAGGCAAGCTC

CACCGTTCAGGCTCCGGCTCTTCCTCTGTAAGTATATTCGATGTTTCTCAAGGGCCATTT

CACTTGGTATATCGTGTTGGGATGACTAGATTAGTTGATTAACGGTTGTGACTGTAAATC

AGTGATATGGGCTTCTATGTACCTTATTTCACAATGATAATCAATTGATACGCTGGATCG

TGATGAGCGGACCGGATGAGCCGTCCATCTTACAGGCTTTTTAAAATAGAATTATAGGGT

GGGTGGTATGAGGTCCACCAAAGCCTGTAACATGGAATTACAATCATAACCGTTAATCGG

TTCATCTCATCCGCTCATCGTGATCCAGCATGTCAAACAGAGATGGGTTTATGGAGAAAT

GGATGTATGTAAGCGCCAACAAATCACAAAAAAATCTGACATAATGATGGAGAGAATGCA

AGGAGTGTAAGTGAATCTACGCCGTTAAATGGATTTCATTGTACTTAATTTCGTTTTGTC

AAATATTTTGTAGGACGAGGATGATGGACAAGGTGGGCGTAGGAAGAAGGGTTTGACGGA

GAAGATCAAAGAGAAGCTGCCAGGTGGAAACAAGACGACAGGTGTCTGTCATCCAGGGAC

TCAGGGAGTACAGGGGGGTCGCGAGCATGAGAAGCCAGGTTGCGGTCAGGGTGGACAGGT

GGGCCGCGAGCATGAGAAGACAGGTTTCGGTCATCCAGGGACTCAGGGTGGACAGGTGGG

CAGCGAGCAGGAGAAGAAGGGTATGATTGAGAAGATCAAGGAGAAGCTGCCAGGTCACAA

GTAGGATGTGTGTCCACCATACACGTGGAGTCTACACCTACGTGTCTTATGTTACTTATA

ATACGCATGGAGTGTGTCCGGAGTCTGTAATAATCTGCGCGTATCTGTTATGTC

>GZXS523

AGCCGTTGGATCTAGTTGAAAATGTCAGAGACGCGTGATGAGTATGGCAACCAGGTTCGC

CAAACCGACGAGTATGGGAACCCGATTCAGCATAGTGGCACCGGGACAAAGCCCGGTTCG

GGCATACATGGTGGGGCCCATGGGATTGGCACAGGTGGTGGTGGTGGACAAGGCAAGCTC

CACCGTCCAGGCTCCGGCTCTTCCTCTGTAAGTATATTCGATGTTTCTCAAGGGTCATTT

CACTTGGTATATAGTGTTGGGATGACTAGATTAGTTGATTAACGGTTGTGATTGTAAATC

AGTGATATGGGCTTCTATGTACCTTATTTCACAATGATAATCAATTGATACGCTGGATCG

TGATGAGCGGACCGGATGAGCCGTCCATCTTACAGGCTTTTTAAAATAGAATTATAGGGT

GGGTGGTATGAGGTCTACCAAGCCTGTAACATGGAATTACAATCATAGCCGTTAATCGGT

TCATCTCATCCGCTCATCGTGATCCAGCATGTCAAACAGAGATGGGTTTATGGAGAAATG

GATGTATGTAAGCCAACAAATCACAAAAAAAATCTGACATAATGATGGAGAGAATGCAAG

GAGTGTAAGATCTACGCCGTTAAATGGATTTCATTGTACTTAATTTCATTTTGTCAAATA

TTTTGTAGGACGAGGATGATGGACAAGGTGGGCGTAGGAAGAAGGGTTTGACGGAGAAGA

TCAAAGAGAAGCTGCCAGGTGGAAACAAGACGACAGGTGTCTGTCATCCAGGGACTCAGG

GTGTACAGGGGGGTCGCGAGCATGAGAAGCCAGGTTGCGGTCAGGGTGGACAGGTGGGCC

GCGAGCATGAGAAGACAGGTTTCGGTCATCCAGGGACTCAGGGTGGACAGGTGGGCAGCG

AGCAGGAGAAGAAGGGTATGATTGAGAAGATCAAGGAGAAGCTGCCAGGTCACAAGTAGG

ATGTGTGTCCACCATACACGTGGAGTCTACACCTACGTGTCTTATGTTACTTATAATACG

CATGGAGTGTGTCCGGAGTCTGTAATAATCTGCGCGTATCTGTTATGTC

>GZLP241

AGCCGTTGGATCTAGTTGAAAATGTCAGAGACGCGTGATGAGTATGGCAACCAGGTTCGC

CAAACCGACGAGTATGGGAACCCGATTCAGCATACTGGCACCGGGACAAAGCCCGGTTCG

GGCATACATGGTGGGGGCCATGGGATTGGCACAGGTGGTGGTGGTGGACAAGGCAAGCTC

CACCGTCCAGGCTCCGGCTCTTCCTCTGTAAGTATATTCGATGTTTCTCAAGGGTCATTT

CACTTGGTATATAGTGTTGGGATGACTAGATTAGTTGATTAACGGTTGTGATTGTAAATC

AGTGATATGGGCTTCTATGTACCTTATTTCACAATGATAATCAATTGATACGCTGGATCG

TGATGAGCGGACCGGATGAGCCGTCCATCTTACAGGCTTTTTAAAATAGAATTATAGGGT

GGGTGGTATGAGGTCTACCAAGCCTGTAACATGGAATTACAATCATAGCCGTTAATCCGT

TCATCTCATCCGCTCATCGTGATCCAGCATGTCAAACAGAGATGGGTTTATGGAGAAATG

GATGTATGTAAGCCAACAAATCACAAAAAAAATCTGACATAATGATGGAGAGAATGCAAG

GAGTGTAAGATCTACGCCGTTAAATGGATTTCATTGTACTTAATTTCATTTTGTCAAATA

TTTTGTAGGACGAGGATGATGGACAAGGTGGGCGTAGGAAGAAGGGTTTGACGGAGAAGA

TCAAAGAGAAGCTGCCAGGTGGAAACAAGACGACAGGTGTCTGTCATCCAGGGACTCAGG

GTGTACAGGGGGGTCGCGAGCATGAGAAGCAAGGTTGCGGTCAGGGTGGACAGGTGGGCC

GCGAGCATGAGAAGACAGGTTTCGGTCATCCAGGGACTCAGGGTGGACAGGTGGGCAGCG

AGCAGGAGAAGAAGGGTATGATTGAGAAGATCAAGGAGAAGCTGCCAGGTCACAAGTAGG

ATGTGTGTCCACCATACACGTGGAGTCTACACCTACGTGTCTTATGTTACTTATAATACG

CATGGAGTGTGTCCGGAGTCTGTAATAATCTGCGCGTATCTGTTATGTC

>GZLP242

AGCCGTTGGATCTAGTTGAAAATGTCAGAGACGCGTGATGAGTATGGCAACCAGGTTCGC

CAAACCGACGAGTATGGGAACCCGATTCAGCATACTGGCACCGGGACAAAGCCCGGTTCG

GGCATACATGGTGGGGGCCATGGGATTGGCACAGGTGGTGGTGGTGGACAAGGCAAGCTC

CACCGTCCAGGCTCCGGCTCTTCCTCTGTAAGTATATTCGATGTTTCTCAAGGGTCATTT

CACTTGGTATATAGTGTTGGGATGACTAGATTAGTTGATTAACGGTTGTGATTGTAAATC

AGTGATATGGGCTTCTATGTACCTTATTTCACAATGATAATCAATTGATACGCTGGATCG

TGATGAGCGGACCGGATGAGCCGTCCATCTTACAGGCTTTTTAAAATAGAATTATAGGGT

GGGTGGTATGAGGTCTACCAAGCCTGTAACATGGAATTATAATCATAGCCGTTAATCCGT

TCATCTCATCCGCTCATCGTGATCCAGCATGTCAAACAGAGATGGGTTTATGGAGAAATG

GATGTATGTAAGCCAACAAATCACAAAAAAAATCTGACATAATGATGGAGAGAATGCAAG

GAGTGTAAGATCTACGCCGTTAAATGGATTTCATTGTACTTAATTTCATTTTGTCAAATA

TTTTGTAGGACGAGGATGATGGACAAGGTGGGCGTAGGAAGAAGGGTTTGACGGAGAAGA

TCAAAGAGAAGCTGCCAGGTGGAAACAAGACGACAGGTGTCTGTCATCCAGGGACTCAGG

GTGTACAGGGGGGTCGCGAGCATGAGAAGCAAGGTTGCGGTCAGGGTGGACAGGTGGGCC

GCGAGCATGAGAAGACAGGTTTCGGTCATCCAGGGACTCAGGGTGGACAGGTGGGCAGCG

AGCAGGAGAAGAAGGGTATGATTGAGAAGATCAAGGAGAAGCTGCCAGGTCACAAGTAGG

ATGTGTGTCCACCATACACGTGGAGTCTACACCTACGTGTCTTATGTTACTTATAATACG

CATGGAGTGTGTCCGGAGTCTGTAATAATCTGCGCGTATCTGTTATGTC

>GZLP244

AGCCGTTGGATCTAGTTGAAAATGTCAGAGACGCGTGATGAGTATGGCAACCAGGTTCGC

CAAACCGACGAGTATGGGAACCCGATTCAGCATACTGGCACCGGGACAAAGCCCGGTTCG

GGCATACATGGTGGGGGCCATGGGATTGGCACAGGTGGTGGTGGTGGACAAGGCAAGCTC

CACCGTCCAGGCTCCGGCTCTTCCTCTGTAAGTATATTCGATGTTTCTCAAGGGTCATTT

CACTTGGTATATAGTGTTGGGATGACTAGATTAGTTGATTAACGGTTGTGATTGTAAATC

AGTGATATGGGCTTCTATGTACCTTATTTCACAATGATAATCAATTGATACGCTGGATCG

TGATGAGCGGACCGGATGAGCCGTCCATCTTACAGGCTTTTTAAAATAGAATTATAGGGT

GGGTGGTATGAGGTCTACCAAGCCTGTAACATGGAATTACAATCATAGCCGTTAATCCGT

TCATCTCATCCGCTCATCGTGATCCAGCATGTCAAACAGAGATGGGTTTATGGAGAAATG

GATGTATGTAAGCCAACAAATCACAAAAAAAATCTGACATAATGATGGAGAGAATGCAAG

GAGTGTAAGATCTACGCCGTTAAATGGATTTCATTGTACTTAATTTCATTTTGTCAAATA

TTTTGTAGGACGAGGATGATGGACAAGGTGGGCGTAGGAAGAAGGGTTTGACGGAGAAGA

TCAAAGAGAAGCTGCCAGGTGGAAACAAGACGACAGGTGTCTGTCATCCAGGGACTCAGG

GTGTACAGGGGGGTCGCGAGCATGAGAAGCAAGGTTGCGGTCAGGGTGGACAGGTGGGCC

GCGAGCATGAGAAGACAGGTTTCGGTCATCCAGGGACTCAGGGTGGACAGGTGGGCAGCG

AGCAGGAGAAGAAGGGTATGATTGAGAAGATCAAGGAGAAGCTGCCAGGTCACAAGTAGG

ATGTGTGTCCACCATACACGTGGAGTCTACACCTACGTGTCTTATGTTACTTATAATACG

CATGGAGTGTGTCCGGAGTCTGTAATAATCTGCGCGTATCTGTTATGTC

>GZLP261

AGCCGTTGGATCTAGTTGAAAATGTCAGAGACGCGTGATGAGTATGGCAACCAGGTTCGC

CAAACCGACGAGTATGGGAACCCGATTCAGCATACTGGCACCGGGACAAAGCCCGGTTCG

GGCATACATGGTGGGGGCCATGGGATTGGCACAGGTGGTGGTGGTGGACAAGGCAAGCTC

CACCGTCCAGGCTCCGGCTCTTCCTCTGTAAGTATATTCGATGTTTCTCAAGGGTCATTT

CACTTGGTATATAGTGTTGGGATGACTAGATTAGTTGATTAACGGTTGTGATTGTAAATC

AGTGATATGGGCTTCTATGTACCTTATTTCACAATGATAATCAATTGATACGCTGGATCG

TGATGAGCGGACCGGATGAGCCGTCCATCTTACAGGCTTTTTAAAATAGAATTATAGGGT

GGGTGGTATGAGGTCTACCAAGCCTGTAACATGGAATTACAATCATAGCCGTTAATCCGT

TCATCTCATCCGCTCATCGTGATCCAGCATGTCAAACAGAGATGGGTTTATGGAGAAATG

GATGTATGTAAGCCAACAAATCACAAAAAAAATCTGACATAATGATGGAGAGAATGCAAG

GAGTGTAAGATCTACGCCGTTAAATGGATTTCATTGTACTTAATTTCATTTTGTCAAATA

TTTTGTAGGACGAGGATGATGGACAAGGTGGGCGTAGGAAGAAGGGTTTGACGGAGAAGA

TCAAAGAGAAGCTGCCAGGTGGAAACAAGACGACAGGTGTCTGTCATCCAGGGACTCAGG

GTGTACAGGGGGGTCGCGAGCATGAGAAGCAAGGTTGCGGTCAGGGTGGACAGGTGGGCC

GCGAGCATGAGAAGACAGGTTTCGGTCATCCAGGGACTCAGGGTGGACAGGTGGGCAGCG

AGCAGGAGAAGAAGGGTATGATTGAGAAGATCAAGGAGAAGCTGCCAGGTCACAAGTAGG

ATGTGTGTCCACCATACACGTGGAGTCTACACCTACGTGTCTTATGTTACTTATAATACG

CATGGAGTGTGTCCGGAGTCTGTAATAATCTGCGCGTATCTGTTATGTC

>GZLP262

AGCCGTTGGATCTAGTTGAAAATGTCAGAGACGCGTGATGAGTATGGCAACCAGGTTCGC

CAAACCGACGAGTATGGGAACCCGATTCAGCATACTGGCACCGGGACAAAGCCCGGTTCG

GGCATACATGGTGGGGGCCATGGGATTGGCACAGGTGGTGGTGGTGGACAAGGCAAGCTC

CACCGTCCAGGCTCCGGCTCTTCCTCTGTAAGTATATTCGATGTTTCTCAAGGGTCATTT

CACTTGGTATATAGTGTTGGGATGACTAGATTAGTTGATTAACGGTTGTGATTGTAAATC

AGTGATATGGGCTTCTATGTACCTTATTTCACAATGATAATCAATTGATACGCTGGATCG

TGATGAGCGGACCGGATGAGCCGTCCATCTTACAGGCTTTTTAAAATAGAATTATAGGGT

GGGTGGTATGAGGTCTACCAAGCCTGTAACATGGAATTACAATCATAGCCGTTAATCCGT

TCATCTCATCCGCTCATCGTGATCCAGCATGTCAAACAGAGATGGGTTTATGGAGAAATG

GATGTATGTAAGCCAACAAATCACAAAAAAAATCTGACATAATGATGGAGAGAATGCAAG

GAGTGTAAGATCTACGCCGTTAAATGGATTTCATTGTACTTAATTTCATTTTGTCAAATA

TTTTGTAGGACGAGGATGATGGACAAGGTGGGCGTAGGAAGAAGGGTTTGACGGAGAAGA

TCAAAGAGAAGCTGCCAGGTGGAAACAAGACGACAGGTGTCTGTCATCCAGGGACTCAGG

GTGTACAGGGGGGTCGCGAGCATGAGAAGCAAGGTTGCGGTCAGGGTGGACAGGTGGGCC

GCGAGCATGAGAAGACAGGTTTCGGTCATCCAGGGACTCAGGGTGGACAGGTGGGCAGCG

AGCAGGAGAAGAAGGGTATGATTGAGAAGATCAAGGAGAAGCTGCCAGGTCACAAGTAGG

ATGTGTGTCCACCATACACGTGGAGTCTACACCTACGTGTCTTATGTTACTTATAATACG

CATGGAGTGTGTCCGGAGTCTGTAATAATCTGCGCGTATCTGTTATGTC

>GZLP264

AGCCGTTGGATCTAGTTGAAAATGTCAGAGACGCGTGATGAGTATGGCAACCAGGTTCGC

CAAACCGACGAGTATGGGAACCCGATTCAGCATAGTGGCACCGGGACAAAGCCCGGTTCG

GGCATACATGGTGGGGGCCATGGGATTGGCACAGGTGGTGGTGGTGGACAAGGCAAGCTC

CACCGTCCAGGCTCCGGCTCTTCCTCTGTAAGTATATTCGATGTTTCTCAAGGGTCATTT

CACTTGGTATATAGTGTTGGGATGACTAGATTAGTTGATTAACGGTTGTGATTGTAAATC

AGTGATATGGGCTTCTATGTACCTTATTTCACAATGATAATCAATTGATACGCTGGATCG

TGATGAGCGGACCGGATGAGCCGTCCATCTTACAGGCTTTTTAAAATAGAATTATAGGGT

GGGTGGTATGAGGTCTACCAAGCCTGTAACATGGAATTACAATCATAGCCGTTAATCCGT

TCATCTCATCCGCTCATCGTGATCCAGCATGTCAAACAGAGATGGGTTTATGGAGAAATG

GATGTATGTAAGCCAACAAATCACAAAAAAAATCTGACATAATGATGGAGAGAATGCAAG

GAGTGTAAGTGAATCTACGCCGTTAAATGGATTTCATTGTACTTAATTTCGTTTTGTCAA

ATATTTTGTAGGACGAGGATGATGGACAAGGTGGGCGTAGGAAGAAGGGTTTGACGGAGA

AGATCAAAGAGAAGCTGCCAGGTGGAAACAAGACGACAGGTGTCTGTCATCAAGGGACTC

AGGGTGTACAGGGGGGTCGCGAGCATGAGAAGCCAGGTTGCGGTCAGGGTGGACAGGTGG

GCCGCGAGCATGAGAAGACAGGTTTCGGTCATCCAGGGACTCAGGGTGGACAGGTGGGCA

GCGAGCAGGAGAAGAAGGGTATGATTGAGAAGATCAAGGAGAAGCTGCCAGGTCACAAGT

AGGATGTGTGTCCACCATACACGTGGAGTCTACACCTACGTGTCTTATGTTACTTATAAT

ACGCATGGAGTGTGTCCGGAGTCTGTAATAATCTGCGCGTATCTGTTATGTC

>GZLPD1014

AGCCGTTGGATCTAGTTGAAAATGTCAGAGACGCGTGATGAGTATGGCAACCAGGTTCGC

CAAACCGACGAGTATGGGAACCCGATTCAGCATAGTGGCACCGGGACAAAGCCCGGTTCG

GGCATACATGGTGGGGGCCATGGGATTGGCACAGGTGGTGGTGGTGGACAAGGCAAGCTC

CACCGTCCAGGCTCCGGCTCTTCCTCTGTAAGTATATTCGATGTTTCTCAAGGGTCATTT

CACTTGGTATATAGTGTTGGGATGACTAGATTAGTTGATTAACGGTTGTGATTGTAAATC

AGTGATATGGGCTTCTATGTACCTTATTTCACAATGATAATCAATTGATACGCTGGATCG

TGATGAGCGGACCGGATGAGCCGTCCATCTTACAGGCTTTTTAAAATAGAATTATAGGGT

GGGTGGTATGAGGTCTACCAAGCCTGTAACATGGAATTACAATCATAGCCGTTAATCCGT

TCATCTCATCCGCTCATCGTGATCCAGCATGTCAAACAGAGATGGGTTTATGGAGAAATG

GATGTATGTAAGCCAACAAATCACAAAAAAAATCTGACATAATGATGGAGAGAATGCAAG

GAGTGTAAGTGAATCTACGCCGTTAAATGGATTTCATTGTACTTAATTTCGTTTTGTCAA

ATATTTTGTAGGACGAGGATGATGGACAAGGTGGGCGTAGGAAGAAGGGTTTGACGGAGA

AGATCAAAGAGAAGCTGCCAGGTGGAAACAAGACGACAGGTGTCTGTCATCAAGGGACTC

AGGGTGTACAGGGGGGTCGCGAGCATGAGAAGCCAGGTTGCGGTCAGGGTGGACAGGTGG

GCCGCGAGCATGAGAAGACAGGTTTCGGTCATCCAGGGACTCAGGGTGGACAGGTGGGCA

GCGAGCAGGAGAAGAAGGGTATGATTGAGAAGATCAAGGAGAAGCTGCCAGGTCACAAGT

AGGATGTGTGTCCACCATACACGTGGAGTCTACACCTACGTGTCTTATGTTACTTATAAT

ACGCATGGAGTGTGTCCGGAGTCTGTAATAATCTGCGCGTATCTGTTATGTC

>GZLPD710

AGCCGTTGGATCTAGTTGAAAATGTCAGAGACGCGTGATGAGTATGGCAACCAGGTTCGC

CAAACCGACGAGTATGGGAACCCGATTCAGCATAGTGGCACCGGGACAAAGCCCGGTTCG

GGCATACATGGTGGGGGCCATGGGATTGGCACAGGTGGTGGTGGTGGACAAGGCAAGCTC

CACCGTCCAGGCTCCGGCTCTTCCTCTGTAAGTATATTCGATGTTTCTCAAGGGTCATTT

CACTTGGTATATAGTGTTGGGATGACTAGATTAGTTGATTAACGGTTGTGATTGTAAATC

AGTGATATGGGCTTCTATGTACCTTATTTCACAATGATAATCAATTGATACGCTGGATCG

TGATGAGCGGACCGGATGAGCCGTCCATCTTACAGGCTTTTTAAAATAGAATTATAGGGT

GGGTGGTATGAGGTCTACCAAGCCTGTAACATGGAATTACAATCATAGCCGTTAATCCGT

TCATCTCATCCGCTCATCGTGATCCAGCATGTCAAACAGAGATGGGTTTATGGAGAAATG

GATGTATGTAAGCCAACAAATCACAAAAAAAATCTGACATAATGATGGAGAGAATGCAAG

GAGTGTAAGTGAATCTACGCCGTTAAATGGATTTCATTGTACTTAATTTCGTTTTGTCAA

ATATTTTGTAGGACGAGGATGATGGACAAGGTGGGCGTAGGAAGAAGGGTTTGACGGAGA

AGATCAAAGAGAAGCTGCCAGGTGGAAACAAGACGACAGGTGTCTGTCATCAAGGGACTC

AGGGTGTACAGGGGGGTCGCGAGCATGAGAAGCCAGGTTGCGGTCAGGGTGGACAGGTGG

GCCGCGAGCATGAGAAGACAGGTTTCGGTCATCCAGGGACTCAGGGTGGACAGGTGGGCA

GCGAGCAGGAGAAGAAGGGTATGATTGAGAAGATCAAGGAGAAGCTGCCAGGTCACAAGT

AGGATGTGTGTCCACCATACACGTGGAGTCTACACCTACGTGTCTTATGTTACTTATAAT

ACGCATGGAGTGTGTCCGGAGTCTGTAATAATCTGCGCGTATCTGTTATGTC

>GZLPD714

AGCCGTTGGATCTAGTTGAAAATGTCAGAGACGCGTGATGAGTATGGCAACCAGGTTCGC

CAAACCGACGAGTATGGGAACCCGATTCAGCATAGTGGCACCGGGACAAAGCCCGGTTCG

GGCATACATGGTGGGGGCCATGGGATTGGCACAGGTGGTGGTGGTGGACAAGGCAAGCTC

CACCGTCCAGGCTCCGGCTCTTCCTCTGTAAGTATATTCGATGTTTCTCAAGGGTCATTT

CACTTGGTATATAGTGTTGGGATGACTAGATTAGTTGATTAACGGTTGTGATTGTAAATC

AGTGATATGGGCTTCTATGTACCTTATTTCACAATGATAATCAATTGATACGCTGGATCG

TGATGAGCGGACCGGATGAGCCGTCCATCTTACAGGCTTTTTAAAATAGAATTATAGGGT

GGGTGGTATGAGGTCTACCAAGCCTGTAACATGGAATTACAATCATAGCCGTTAATCCGT

TCATCTCATCCGCTCATCGTGATCCAGCATGTCAAACAGAGATGGGTTTATGGAGAAATG

GATGTATGTAAGCCAACAAATCACAAAAAAAATCTGACATAATGATGGAGAGAATGCAAG

GAGTGTAAGTGAATCTACGCCGTTAAATGGATTTCATTGTACTTAATTTCGTTTTGTCAA

ATATTTTGTAGGACGAGGATGATGGACAAGGTGGGCGTAGGAAGAAGGGTTTGACGGAGA

AGATCAAAGAGAAGCTGCCAGGTGGAAACAAGACGACAGGTGTCTGTCATCAAGGGACTC

AGGGTGTACAGGGGGGTCGCGAGCATGAGAAGCCAGGTTGCGGTCAGGGTGGACAGGTGG

GCCGCGAGCATGAGAAGACAGGTTTCGGTCATCCAGGGACTCAGGGTGGACAGGTGGGCA

GCGAGCAGGAGAAGAAGGGTATGATTGAGAAGATCAAGGAGAAGCTGCCAGGTCACAAGT

AGGATGTGTGTCCACCATACACGTGGAGTCTACACCTACGTGTCTTATGTTACTTATAAT

ACGCATGGAGTGTGTCCGGAGTCTGTAATAATCTGCGCGTATCTGTTATGTC

>GZLPD716

AGCCGTTGGATCTAGTTGAAAATGTCAGAGACGCGTGATGAGTATGGCAACCAGGTTCGC

CAAACCGACGAGTATGGGAACCCGATTCAGCATAGTGGCACCGGGACAAAGCCCGGTTCG

GGCATACATGGTGGGGGCCATGGGATTGGCACAGGTGGTGGTGGTGGACAAGGCAAGCTC

CACCGTCCAGGCTCCGGCTCTTCCTCTGTAAGTATATTCGATGTTTCTCAAGGGTCATTT

CACTTGGTATATAGTGTTGGGATGACTAGATTAGTTGATTAACGGTTGTGATTGTAAATC

AGTGATATGGGCTTCTATGTACCTTATTTCACAATGATAATCAATTGATACGCTGGATCG

TGATGAGCGGACCGGATGAGCCGTCCATCTTACAGGCTTTTTAAAATAGAATTATAGGGT

GGGTGGTATGAGGTCTACCAAGCCTGTAACATGGAATTACAATCATAGCCGTTAATCCGT

TCATCTCATCCGCTCATCGTGATCCAGCATGTCAAACAGAGATGGGTTTATGGAGAAATG

GATGTATGTAAGCCAACAAATCACAAAAAAAATCTGACATAATGATGGAGAGAATGCAAG

GAGTGTAAGTGAATCTACGCCGTTAAATGGATTTCATTGTACTTAATTTCGTTTTGTCAA

ATATTTTGTAGGACGAGGATGATGGACAAGGTGGGCGTAGGAAGAAGGGTTTGACGGAGA

AGATCAAAGAGAAGCTGCCAGGTGGAAACAAGACGACAGGTGTCTGTCATCAAGGGACTC

AGGGTGTACAGGGGGGTCGCGAGCATGAGAAGCCAGGTTGCGGTCAGGGTGGACAGGTGG

GCCGCGAGCATGAGAAGACAGGTTTCGGTCATCCAGGGACTCAGGGTGGACAGGTGGGCA

GCGAGCAGGAGAAGAAGGGTATGATTGAGAAGATCAAGGAGAAGCTGCCAGGTCACAAGT

AGGATGTGTGTCCACCATACACGTGGAGTCTACACCTACGTGTCTTATGTTACTTATAAT

ACGCATGGAGTGTGTCCGGAGTCTGTAATAATCTGCGCGTATCTGTTATGTC

>SCXY71

AGCCGTTGGATCTAGTTGAAAATGTCAGAGACGCGTGATGAGTATGGCAACCAGGTTCGC

CAAACCGACGAGTATGGGAACCCGATTCAGCATAGTGGCACCGGGACAAAGCCCGGTTCG

GGCATACATGGTGGGGGCCATGGGATTGGCACAGGTGGTGGTGGTGGACAAGGCAAGCTC

CACCGTCCAGGCTCCGGCTCTTCCTCTGTAAGTATATTCGATGTTTCTCAAGGGTCATTT

CACTTGGTATATCGTGTTGGGATGACTAGATTAGTTGATTAACGGTTGTGATTGTAAATC

AGTGATATGGGCTTCTGTGTACCTTATTTCACAATGATAATCAATTGATACGCTGGATCG

TGATGAGCGGACCGGATGAGCCGTCCATCTTACAGGCTTTTTAAAATAGAATTATAGGGT

GGGTGGTATGAGGTCTACCAAGCCTGTAACATGGAATTACAATCATAGCCGTTAATCGGT

TCATCTCATCCGCTCATCGTGATCCAGCATGTCAAACAGAGATGGGTTTATGGAGAAATG

GATGTATGTAAGCCAACAAATCACAAAAAAAATCTGACATAATGATGGAGAGAATGCAAG

GAGTGTAAGTGAATCTACGCCGTTAAATGGATTTCATTGTACTTAATTTCAATTTCGTTT

TGTCAAATATTTTGTAGGACGAGGATGATGGACAAGGTGGGCGGAGGAAGAAGGGTTTGA

CGGAGAAGATCAAAGAGAAGCTGCCAGGTGGAAACAAGACGACAGGTGTCTGTCATCCAG

GGACTCAGGGTGTACAGGGGGGTCGCGAGCATGAGAAGCCAGGTTGCGGTCAGGGTGGAC

AGGTGGGCCGCGAGCATGAGAAGACAGGTTTCGGTCATCCAGGGACTCAGGGTGGACAGG

TGGGCAGCGAGCAGGAGAAGAAGGGTATGATTGAGAAGATCAAGGAGAAGCTGCCAGGTC

ACAAGTAGGATGTGTGTCCACCATACACGTGGAGTCTACACCTACGTGTCTTATGTTACT

TATAATACGCATGGAGTGTGTCCGGAGTCTGTAATAATCTGCGCGTATCTGTTATGTC

>SCXY72

AGCCGTTGGATCTAGTTGAAAATGTCAGAGACGCGTGATGAGTATGGCAACCAGGTTCGC

CAAACCGACGAGTATGGGAACCCGATTCAGCATAGTGGCACCGGGACAAAGCCCGGTTCG

GGCATACATGGTGGGGCCCATGGGATTGGCACAGGTGGTGGTGGTGGACAAGGCAAGCTC

CACCGTCCAGGCTCCGGCTCTTCCTCTGTAAGTATATTCGATGTTTCTCAAGGGTCATTT

CACTTGGTATATAGTGTTGGGATGACTAGATTAGTTGATTAACGGTTGTGATTGTAAATC

AGTGATATGGGCTTCTATGTACCTTATTTCACAATGATAATCAATTGATACGCTGGATCG

TGATGAGCGGACCGGATGAGCCGTCCATCTTACAGGCTTTTTAAAATAGAATTATAGGGT

GGGTGGTATGAGGTCTACCAAGCCTGTAACATGGAATTACAATCATAGCCGTTAATCGGT

TCATCTCATCCGCTCATCGTGATCCAGCATGTCAAACAGAGATGGGTTTATGGAGAAATG

GATGTATGTAAGCCAACAAATCACAAAAAAAATCTGACATAATGATGGAGAGAATGCAAG

GAGTGTAAGATCTACGCCGTTAAATGGATTTCATTGTACTTAATTTCATTTTGTCAAATA

TTTTGTAGGACGAGGATGATGGACAAGGTGGGCGTAGGAAGAAGGGTTTGACGGAGAAGA

TCAAAGAGAAGCTGCCAGGTGGAAACAAGACGACAGGTGTCTGTCATCCAGGGACTCAGG

GTGTACAGGGGGGTCGCGAGCATGAGAAGCCAGGTTGCGGTCAGGGTGGACAGGTGGGCC

GCGAGCATGAGAAGACAGGTTTCGGTCATCCAGGGACTCAGGGTGGACAGGTGGGCAGCG

AGCAGGAGAAGAAGGGTATGATTGAGAAGATCAAGGAGAAGCTGCCAGGTCACAAGTAGG

ATGTGTGTCCACCATACACGTGGAGTCTACACCTACGTGTCTTATGTTACTTATAATACG

CATGGAGTGTGTCCGGAGTCTGTAATAATCTGCGCGTATCTGTTATGTC

>SCXY73

AGCCGTTGGATCTAGTTGAAAATGTCAGAGACGCGTGATGAGTATGGCAACCAGGTTCGC

CAAACCGACGAGTATGGGAACCCGATTCAGCATAGTGGCACCGGGACAAAGCCCGGTTCG

GGCATACATGGTGGGGCCCATGGGATTGGCACAGGTGGTGGTGGTGGACAAGGCAAGCTC

CACCGTCCAGGCTCCGGCTCTTCCTCTGTAAGTATATTCGATGTTTCTCAAGGGTCATTT

CACTTGGTATATAGTGTTGGGATGACTAGATTAGTTGATTAACGGTTGTGATTGTAAATC

AGTGATATGGGCTTCTATGTACCTTATTTCACAATGATAATCAATTGATACGCTGGATCG

TGATGAGCGGACCGGATGAGCCGTCCATCTTACAGGCTTTTTAAAATAGAATTATAGGGT

GGGTGGTATGAGGTCTACCAAGCCTGTAACATGGAATTACAATCATAGCCGTTAATCGGT

TCATCTCATCCGCTCATCGTGATCCAGCATGTCAAACAGAGATGGGTTTATGGAGAAATG

GATGTATGTAAGCCAACAAATCACAAAAAAAATCTGACATAATGATGGAGAGAATGCAAG

GAGTGTAAGATCTACGCCGTTAAATGGATTTCATTGTACTTAATTTCATTTTGTCAAATA

TTTTGTAGGACGAGGATGATGGACAAGGTGGGCGTAGGAAGAAGGGTTTGACGGAGAAGA

TCAAAGAGAAGCTGCCAGGTGGAAACAAGACGACAGGTGTCTGTCATCCAGGGACTCAGG

GTGTACAGGGGGGTCGCGAGCATGAGAAGCCAGGTTGCGGTCAGGGTGGACAGGTGGGCC

GCGAGCATGAGAAGACAGGTTTCGGTCATCCAGGGACTCAGGGTGGACAGGTGGGCAGCG

AGCAGGAGAAGAAGGGTATGATTGAGAAGATCAAGGAGAAGCTGCCAGGTCACAAGTAGG

ATGTGTGTCCACCATACACGTGGAGTCTACACCTACGTGTCTTATGTTACTTATAATACG

CATGGAGTGTGTCCGGAGTCTGTAATAATCTGCGCGTATCTGTTATGTC

>SCXY257

AGCCGTTGGATCTAGTTGAAAATGTCAGAGACGCGTGATGAGTATGGCAACCAGGTTCGC

CAAACCGACGAGTATGGGAACCCGATTCAGCATAGTGGCACCGGGACAAAGCCCGGTTCG

GGCATACATGGTGGGGGCCATGGGATTGGCACAGGTGGTGGTGGTGGACAAGGCAAGCTC

CACCGTCCAGGCTCCGGCTCTTCCTCTGTAAGTATATTCGATGTTTCTCAAGGGTCATTT

CACTTGGTATATCGTGTTGGGATGACTAGATTAGTTGATTAACGGTTGTGATTGTAAATC

AGTGATATGGGCTTCTGTGTACCTTATTTCACAATGATAATCAATTGATACGCTGGATCG

TGATGAGCGGACCGGATGAGCCGTCCATCTTACAGGCTTTTTAAAATAGAATTATAGGGT

GGGTGGTATGATGTCTACCAAGCCTGTAACATGGAATTACAATCATAGCCGTTAATCGGT

TCATCTCATCCGCTCATCGTGATCCAGCATGTCAAACAGAGATGGGTTTATGGAGAAATG

GATGTATGTAAGCCAACAAATCACAAAAAAAATCTGACATAATGATGGAGAGAATGCAAG

GAGTGTAAGTGAATCTACGCCGTTAAATGGATTTCATTGTACTTAATTTCAATTTCGTTT

TGTCAAATATTTTGTAGGACGAGGATGATGGACAAGGTGGGCGGAGGAAGAAGGGTTTGA

CGGAGAAGATCAAAGAGAAGCTGCCAGGTGGAAACAAGACGACAGGTGTCTGTCATCCAG

GGACTCAGGGTGTACAGGGGGGTCGCGAGCATGAGAAGCCAGGTTGCGGTCAGGGTGGAC

AGGTGGGCCGCGAGCATGAGAAGACAGGTTTCGGTCATCCAGGGACTCAGGGTGGACAGG

TGGGCAGCGAGCAGGAGAAGAAGGGTATGATTGAGAAGATCAAGGAGAAGCTGCCAGGTC

ACAAGTAGGATGTGTGTCCACCATACACGTGGAGTCTACACCTACGTGTCTTATGTTACT

TATAATACGCATGGAGTGTGTCCGGAGTCTGTAATAATCTGCGCGTATCTGTATGTC

>SCXY259

AGCCGTTGGATCTAGTTGAAAATGTCAGAGACGCGTGATGAGTATGGCAACCAGGTTCGC

CAAACCGACGAGTATGGGAACCCGATTCAGCATAGTGGCACCGGGACAAAGCCCGGTTCG

GGCATACATGGTGGGGGCCATGGGATTGGCACAGGTGGTGGTGGTGGACAAGGCAAGCTC

CACCGTCCAGGCTCCGGCTCTTCCTCTGTAAGTATATTCGATGTTTCTCAAGGGTCATTT

CACTTGGTATATCGTGTTGGGATGACTAGATTAGTTGATTAACGGTTGTGATTGTAAATC

AGTGATATGGGCTTCTGTGTACCTTATTTCACAATGATAATCAATTGATACGCTGGATCG

TGATGAGCGGACCGGATGAGCCGTCCATCTTACAGGCTTTTTAAAATAGAATTATAGGGT

GGGTGGTATGAGGTCTACCAAGCCTGTAACATGGAATTACAATCATAGCCGTTAATCGGT

TCATCTCATCCGCTCATCGTGATCCAGCATGTCAAACAGAGATGGGTTTATGGAGAAATG

GATGTATGTAAGCCAACAAATCACAAAAAAAATCTGACATAATGATGGAGAGAATGCAAG

GAGTGTAAGTGAATCTACGCCGTTAAATGGATTTCATTGTACTTAATTTCAATTTCGTTT

TGTCAAATATTTTGTAGGACGAGGATGATGGACAAGGTGGGCGGAGGAAGAAGGGTTTGA

CGGAGAAGATCAAAGAGAAGCTGCCAGGTGGAAACAAGACGACAGGTGTCTGTCATCCAG

GGACTCAGGGTGTACAGGGGGGTCGCGAGCATGAGAAGCCAGGTTGCGGTCAGGGTGGAC

AGGTGGGCCGCGAGCATGAGAAGACAGGTTTCGGTCATCCAGGGACTCAGGGTGGACAGG

TGGGCAGCGAGCAGGAGAAGAAGGGTATGATTGAGAAGATCAAGGAGAAGCTGCCAGGTC

ACAAGTAGGATGTGTGTCCACCATACACGTGGAGTCTACACCTACGTGTCTTATGTTACT

TATAATACGCATGGAGTGTGTCCGGAGTCTGTAATAATCTGCGCGTATCTGTTATGTC

>SCXY2512

AGCCGTTGGATCTAGTTGAAAATGTCAGAGACGCGTGATGAGTATGGCAACCAGGTTCGC

CAAACCGACGAGTATGGGAACCCGATTCAGCATAGTGGCACCGGGACAAAGCCCGGTTCG

GGCATACATGGTGGGGGCCATGGGATTGGCACAGGTGGTGGTGGTGGACAAGGCAAGCTC

CACCGTCCAGGCTCCGGCTCTTCCTCTGTAAGTATATTCGATGTTTCTCAAGGGTCATTT

CACTTGGTATATCGTGTTGGGATGACTAGATTAGTTGATTAACGGTTGTGATTGTAAATC

AGTGATATGGGCTTCTGTGTACCTTATTTCACAATGATAATCAATTGATACGCTGGATCG

TGATGAGCGGACCGGATGAGCCGTCCATCTTACAGGCTTTTTAAAATAGAATTATAGGGT

GGGTGGTATGAGGTCTACCAAGCCTGTAACATGGAATTACAATCATAGCCGTTAATCGGT

TCATCTCATCCGCTCATCGTGATCCAGCATGTCAAACAGAGATGGGTTTATGGAGAAATG

GATGTATGTAAGCCAACAAATCACAAAAAAAATCTGACATAATGATGGAGAGAATGCAAG

GAGTGTAAGTGAATCTACGCCGTTAAATGGATTTCATTGTACTTAATTTCAATTTCGTTT

TGTCAAATATTTTGTAGGACGAGGATGATGGACAAGGTGGGCGGAGGAAGAAGGGTTTGA

CGGAGAAGATCAAAGAGAAGCTGCCAGGTGGAAACAAGACGACAGGTGTCTGTCATCCAG

GGACTCAGGGTGTACAGGGGGGTCGCGAGCATGAGAAGCCAGGTTGCGGTCAGGGTGGAC

AGGTGGGCCGCGAGCATGAGAAGACAGGTTTCGGTCATCCAGGGACTCAGGGTGGACAGG

TGGGCAGCGAGCAGGAGAAGAAGGGTATGATTGAGAAGATCAAGGAGAAGCTGCCAGGTC

ACAAGTAGGATGTGTGTCCACCATACACGTGGAGTCTACACCTACGTGTCTTATGTTACT

TATAATACGCATGGAGTGTGTCCGGAGTCTGTAATAATCTGCGCGTATCTGTTATGTC

>SCXY272

AGCCGTTGGATCTAGTTGAAAATGTCAGAGACGCGTGATGAGTATGGCAACCAGGTTCGC

CAAACCGACGAGTATGGGAACCCGATTCAGCATAGTGGCACCGGGACAAAGCCCGGTTCG

GGCATACATGGTGGGGGCCATGGGATTGGCACAGGTGGTGGTGGTGGACAAGGCAAGCTC

CACCGTCCAGGCTCCGGCTCTTCCTCTGTAAGTATATTCGATGTTTCTCAAGGGTCATTT

CACTTGGTATATCGTGTTGGGATGACTAGATTAGTTGATTAACGGTTGTGATTGTAAATC

AGTGATATGGGCTTCTGTGTACCTTATTTCACAATGATAATCAATTGATACGCTGGATCG

TGATGAGCGGACCGGATGAGCCGTCCATCTTACAGGCTTTTTAAAATAGAATTATAGGGT

GGGTGGTATGAGGTCTACCAAGCCTGTAACATGGAATTACAATCATAGCCGTTAATCGGT

TCATCTCATCCGCTCATCGTGATCCAGCATGTCAAACAGAGATGGGTTTATGGAGAAATG

GATGTATGTAAGCCAACAAATCACAAAAAAAATCTGACATAATGATGGAGAGAATGCAAG

GAGTGTAAGTGAATCTACGCCGTTAAATGGATTTCATTGTACTTAATTTCAATTTCGTTT

TGTCAAATATTTTGTAGGACGAGGATGATGGACAAGGTGGGCGGAGGAAGAAGGGTTTGA

CGGAGAAGATCAAAGAGAAGCTGCCAGGTGGAAACAAGACGACAGGTGTCTGTCATCCAG

GGACTCAGGGTGTACAGGGGGGTCGCGAGCATGAGAAGCCAGGTTGCGGTCAGGGTGGAC

AGGTGGGCCGCGAGCATGAGAAGACAGGTTTCGGTCATCCAGGGACTCAGGGTGGACAGG

TGGGCAGCGAGCAGGAGAAGAAGGGTATGATTGAGAAGATCAAGGAGAAGCTGCCAGGTC

ACAAGTAGGATGTGTGTCCACCATACACGTGGAGTCTACACCTACGTGTCTTATGTTACT

TATAATACGCATGGAGTGTGTCCGGAGTCTGTAATAATCTGCGCGTATCTGTTATGTC

>SCXY274

AGCCGTTGGATCTAGTTGAAAATGTCAGAGACGCGTGATGAGTATGGCAACCAGGTTCGC

CAAACCGACGAGTATGGGAACCCGATTCAGCATACTGGCACCGGGACAAAGCCCGGTTCG

GGCATACATGGTGGGGGCCATGGGATTGGCACAGGTGGTGGTGGTGGACAAGGCAAGCTC

CACCGTTCAGGCTCCGGCTCTTCCTCTGTAAGTATATTCGATGTTTCTCAAGGGCCATTT

CACTTGGTATATCGTGTTGGGATGACTAGATTAGTTGATTAACGGTTGTGACTGTAAATC

AGTGATATGGGCTTCTATGTACCTTATTTCACAATGATAATCAATTGATACGCTGGATCG

TGATGAGCGGACCGGATGAGCCGTCCATCTTACAGGCTTTTTAAAATAGAATTATAGGGT

GGGTGGTATGAGGTCCACCAAAGCCTGTAACATGGAATTACAATCATAACCGTTAATCGG

TTCATCTCATCCGCTCATCGTGATCCAGCATGTCAAACAGAGATGGGTTTATGGAGAAAT

GGATGTATGTAAGCCAACAAATCACAAAAAAAATCTGACATAATGATGGAGAGAATGCAA

GGAGTGTAAGTGAATCTACGCCGTTAAATGGATTTCATTGTACTTAATTTCAATTTCGTT

TTGTCAAATATTTTGTAGGACGAGGATGATGGACAAGGTGGGCGGAGGAAGAAGGGTTTG

ACGGAGAAGATCAAAGAGAAGCTGCCAGGTGGAAACAAGACGACAGGTGTCTGTCATCCA

GGGACTCAGGGTGTACAGGGGGGTCGCGAGCATGAGAAGCCAGGTTGCGGTCAGGGTGGA

CAGGTGGGCCGCGAGCATGAGAAGACAGGTTTCGGTCATCCAGGGACTCAGGGTGGACAG

GTGGGCAGCGAGTAGGAGAAGAAGGGTATGATTGAGAAGATCAAGGAGAAGCTGCCAGGT

CACAAGTAGGATGTGTGTCCACCATACACGTGGAGTCTACACCTACGTGTCTTATGTTAC

TTATAATACGCATGGAGTGTGTCCGGAGTCTGTAATAATCTGCGCGTATCTGTTATGTC

>SCXY276

AGCCGTTGGATCTAGTTGAAAATGTCAGAGACGCGTGATGAGTATGGCAACCAGGTTCGC

CAAACCGACGAGTATGGGAACCCGATTCAGCATAGTGGCACCGGGACAAAGCCCGGTTCG

GGCATACATGGTGGGGGCCATGGGATTGGCACAGGTGGTGGTGGTGGACAAGGCAAGCTC

CACCGTCCAGGCTCCGGCTCTTCCTCTGTAAGTATATTCGATGTTTCTCAAGGGTCATTT

CACTTGGTATATCGTGTTGGGATGACTAGATTAGTTGATTAACGGTTGTGATTGTAAATC

AGTGATATGGGCTTCTGTGTACCTTATTTCACAATGATAATCAATTGATACGCTGGATCG

TGATGAGCGGACCGGATGAGCCGTCCATCTTACAGGCTTTTTAAAATAGAATTATAGGGT

GGGTGGTATGAGGTCTACCAAGCCTGTAACATGGAATTACAATCATAGCCGTTAATCGGT

TCATCTCATCCGCTCATCGTGATCCAGCATGTCAAACAGAGATGGGTTTATGGAGAAATG

GATGTATGTAAGCCAACAAATCACAAAAAAAATCTGACATAATGATGGAGAGAATGCAAG

GAGTGTAAGTGAATCTACGCCGTTAAATGGATTTCATTGTACTTAATTTCAATTTCGTTT

TGTCAAATATTTTGTAGGACGAGGATGATGGACAAGGTGGGCGGAGGAAGAAGGGTTTGA

CGGAGAAGATCAAAGAGAAGCTGCCAGGTGGAAACAAGACGACAGGTGTCTGTCATCCAG

GGACTCAGGGTGTACAGGGGGGTCGCGAGCATGAGAAGCCAGGTTGCGGTCAGGGTGGAC

AGGTGGGCCGCGAGCATGAGAAGACAGGTTTCGGTCATCCAGGGACTCAGGGTGGACAGG

TGGGCAGCGAGCAGGAGAAGAAGGGTATGATTGAGAAGATCAAGGAGAAGCTGCCAGGTC

ACAAGTAGGATGTGTGTCCACCATACACGTGGAGTCTACACCTACGTGTCTTATGTTACT

TATAATACGCATGGAGTGTGTCCGGAGTCTGTAATAATCTGCGCGTATCTGTTATGTC

>SCYY91

AGCCGTTGGATCTAGTTGAAAATGTCAGAGACGCGTGATGAGTATGGCAACCAGGTTCGC

CAAACCGACGAGTATGGGAACCCGATTCAGCATACTGGCACCGGGACAAAGCCCGGTTCG

GGCATACATGGTGGGGGCCATGGGATTGGCACAGGTGGTGGTGGTGGACAAGGCAAGCTC

CACCGTCCAGGCTCCGGTTCTTCCTCTGTAAGTATATTCGATGTTTCTCAAGGGTCATTT

CACTTGGTATATAGTGTTGGGATGACTAGATTAGTTGATTAACGGTTGTGATTGTAAATC

AGTGATATGGGCTTCTATGTACCTTATTTCACAATGATAATCAATTGATACGCTGGATCG

TGATGAGCGGACCGGATGAGCCGTCCATCTTACAGGCTTTTTAAAATAGAATTATAGGGT

GGGTGGTATGAGGTCTACCAAGCCTGTAACATGGAATTACAATCATAGCCGTTAATCGGT

TCATCTCATCCGCTCATCGTGATGCAGCATGTCAAACAGAGATGGGTTTATGGAGAAATG

GATGTATGTAAGCCAACAAATCACAAAAAAAATCTGACATAATGATGGAGAGAATGCAAG

GAGTGTAAGTGAATCTACGCCGTTAAATGGATTTCATTGTACTTAATTTCGTTTTGTCAA

ATATTTTGTAGGACGAGGATGATGGACAAGGTGGGCGTAGGAAGAAGGGTTTGACGGAGA

AGATCAAAGAGAAGCTGCCAGGTGGAAACAAGACGACAGGTGTCTGTCATCCAGGGACTC

AGGGTGTACAGGGGGGTCGCGAGCATGAGAAGCCAGGTTGCGGTCAGGGTGGACAGGTGG

GCCGCGAGCATGAGAAGACAGGTTTCGGTCATCCAGGAACTCAGGGTGGACAGGTGGGCA

GCGAGCAGGAGAAGAAGGGTATGATTGAGAAGATCAAGGAGAAGCTGCCAGGTCACAAGT

AGGATGTGTGTCCACCATACACGTGGAGCCTACATCTACGTGTCTTATGTTACTTATAAT

ACGCATGGAGTGTGTCCGGAGTCTGTAATAATCTGCGCGTATCTGTTATGTC

>SCYY95

AGCCGTTGGATCTAGTTGAAAATGTCAGAGACGCGTGATGAGTATGGCAACCAGGTTCGC

CAAACCGACGAGTATGGGAACCCGATTCAGCATACTGGCACCGGGACAAAGCCCGGTTCG

GGCATACATGGTGGGGGCCATGGGATTGGCACAGGTGGTGGTGGTGGACAAGGCAAGCTC

CACCGTCCAGGCTCCGGTTCTTCCTCTGTAAGTATATTCGATGTTTCTCAAGGGTCATTT

CACTTGGTATATAGTGTTGGGATGACTAGATTAGTTGATTAACGGTTGTGATTGTAAATC

AGTGATATGGGCTTCTATGTACCTTATTTCACAATGATAATCAATTGATACGCTGGATCG

TGATGAGCGGACCGGATGAGCCGTCCATCTTACAGGCTTTTTAAAATAGAATTATAGGGT

GGGTGGTATGAGGTCTACCAAGCCTGTAACATGGAATTACAATCATAGCCGTTAATCGGT

TCATCTCATCCGCTCATCGTGATGCAGCATGTCAAACAGAGATGGGTTTATGGAGAAATG

GATGTATGTAAGCCAACAAATCACAAAAAAAATCTGACATAATGATGGAGAGAATGCAAG

GAGTGTAAGTGAATCTACGCCGTTAAATGGATTTCATTGTACTTAATTTCGTTTTGTCAA

ATATTTTGTAGGACGAGGATGATGGACAAGGTGGGCGTAGGAAGAAGGGTTTGACGGAGA

AGATCAAAGAGAAGCTGCCAGGTGGAAACAAGACGACAGGTGTCTGTCATCCAGGGACTC

AGGGTGTACAGGGGGGTCGCGAGCATGAGAAGCCAGGTTGCGGTCAGGGTGGACAGGTGG

GCCGCGAGCATGAGAAGACAGGTTTCGGTCATCCAGGAACTCAGGGTGGACAGGTGGGCA

GCGAGCAGGAGAAGAAGGGTATGATTGAGAAGATCAAGGAGAAGCTGCCAGGTCACAAGT

AGGATGTGTGTCCACCATACACGTGGAGCCTACATCTACGTGTCTTATGTTACTTATAAT

ACGCATGGAGTGTGTCCGGAGTCTGTAATAATCTGCGCGTATCTGTTATGTC

>SCYY911

AGCCGTTGGATCTAGTTGAAAATGTCAGAGACGCGTGATGAGTATGGCAACCAGGTTCGC

CAAACCGACGAGTATGGGAACCCGATTCAGCATACTGGCACCGGGACAAAGCCCGGTTCG

GGCATACATGGTGGGGGCCATGGGATTGGCACAGGTGGTGGTGGTGGACAAGGCAAGCTC

CACCGTCCAGGCTCCGGCTCTTCCTCTGTAAGTATATTCGATGTTTCTCAAGGGTCATTT

CACTTGGTATATAGTGTTGGGATGACTAGATTAGTTGATTAACGGTTGTGATTGTAAATC

AGTGATATGGGCTTCTATGTACCTTATTTCACAATGATAATCAATTGATACGCTGGATCG

TGATGAGCGGACCGGATGAGCCGTCCATCTTACAGGCTTTCTAAAATAGAATTATAGGGT

GGGTGGTATGAGGTCTACCAAGCCTGTAACATGGAATTACAATCATAGCCGTTAATCGGT

TCATCTCATCCGCTCATCGTGATCCAGCATGTCAAACAGAGATGGGTTTATGGAGAAATG

GATGTATGTAAGCCAACAAATCACAAAAAAAATCTGACATAATGATGGAGAGAATGCAAG

GAGTGTAAGTGAATCTACGCCGTTAAATGGATTTCATTGTACTTAATTTCGTTTTGTCAA

ATATTTTGTAGGACGAGGATGATGGACAAGGTGGGCGTAGGAAGAAGGGTTTGACGGAGA

AGATCAAAGAGAAGCTGCCAGGTGGAAACAAGACGACAGGTGTCTGTCATCCAGGGACTC

AGGGTGTACAGGGGGGTCGCGAGCATGAGAAGCCAGGTTGCGGTCAGGGTGGACAGGTGG

GCCGCGAGCATGAGAAGACAGGTTTCGGTCATCCAGGGACTCAGGGTGGACAGGTGGGCA

GCGAGCAGGAGAAGAAGGGTATGATTGAGAAGATCAAGGAGAAGCTGCCAGGTCACAAGT

AGGATGTGTGTCCACCATACACGTGGAGTCTACATCTACGTGTCTTATGTTACTTATAAT

ACGCATGGAGTGTGTCCGGAGTCTGTAATAATCTGCGCGTATCTGTTATGTC

>SCYY81

AGCCGTTGGATCTAGTTGAAAATGTCAGAGACGCGTGATGAGTATGGCAACCAGGTTCGC

CAAACCGACGAGTATGGGAACCCGATTCAGCATAGTGGCACCGGGACAAAGCCCGGTTCG

GGCATACATGGTGGGGGCCATGGGATTGGCACAGGTGGTGGTGGTGGACAAGGCAAGCTC

CACCGTCCAGGCTCCGGCTCTTCCTCTGTAAGTATATTCGATGTTTCTCAAGGGTCATTT

CACTTGGTATATCGTGTTGGGATGACTAGATTAGTTGATTAACGGTTGTGATTGTAAATC

AGTGATATGGGCTTCTGTGTACCTTATTTCACAATGATAATCAATTGATACGCTGGATCG

TGATGAGCGGACCGGATGAGCCGTCCATCTTACAGGCTTTTTAAAATAGAATTATAGGGT

GGGTGGTATGAGGTCCACCAAGCCTGTAACATGGAATTACAATCATAGCCGTTAATCGGT

TCATCTCATCCGCTCATCGTGATCCAGCATGTCAAACAGAGATGGGTTTCTGGAGAAATG

GATGTATGTAAGCCCACAAATCACAAAAAAAATCTGACATAATGATGGAGAGAATGCAAG

GAGTGTAAGTGAATCTACGCCGTTAAATGGATTTCATTGTACTTAATTTCAATTTCGTTT

TGTCAAATATTTTGTAGGACGAGGATGATGGACAAGGTGGGCGGAGGAAGAAGGGTTTGA

CGGAGAAGATCAAAGAGAAGCTGCCAGGTGGAAACAAGACGACAGGTGTCTGTCATCCAG

GGACTCAGGGTGTACAGGGGGGTCGCGAGCATGAGAAGCCAGGTTGCGGTCAGGGTGGAC

AGGTGGGCCGCGAGCATGAGAAGACAGGTTTCGGTCATCCAGGGACTCAGGGTGGACAGG

TGGGCAGCGAGCAGGAGAAGAAGGGTATGATTGAGAAGATAAAGGAGAAGCTGCCAGGTC

ACAAGTAGGATGTGTGTCCACCATACACGTGGAGTCTACACCTACGTGTCTTATGTTACT

TATAATACGCATGGAGTGTGTCCGGAGTCTGTAATAATCTGCGCGTATCTGTTATGTC

>SCYY83

AGCCGTTGGATCTAGTTGAAAATGTCAGAGACGCGTGATGAGTATGGCAACCAGGTTCGC

CAAACCGACGAGTATGGGAACCCGATTCAGCATAGTGGCACCGGGACAAAGCCCGGTTCG

GGCATACATGGTGGGGGCCATGGGATTGGCACAGGTGGTGGTGGTGGACAAGGCAAGCTC

CACCGTCCAGGCTCCGGCTCTTCCTCTGTAAGTATATTCGATGTTTCTCAAGGGTCATTT

CACTTGGTATATCGTGTTGGGATGACTAGATTAGTTGATTAACGGTTGTGATTGTAAATC

AGTGATATGGGCTTCTGTGTACCTTATTTCACAATGATAATCAATTGATACGCTGGATCG

TGATGAGCGGACCGGATGAGCCGTCCATCTTACAGGCTTTTTAAAATAGAATTATAGGGT

GGGTGGTATGAGGTCCACCAAGCCTGTAACATGGAATTACAATCATAGCCGTTAATCGGT

TCATCTCATCCGCTCATCGTGATCCAGCATGTCAAACAGAGATGGGTTTCTGGAGAAATG

GATGTATGTAAGCCCACAAATCACAAAAAAAATCTGACATAATGATGGAGAGAATGCAAG

GAGTGTAAGTGAATCTACGCCGTTAAATGGATTTCATTGTACTTAATTTCAATTTCGTTT

TGTCAAATATTTTGTAGGACGAGGATGATGGACAAGGTGGGCGGAGGAAGAAGGGTTTGA

CGGAGAAGATCAAAGAGAAGCTGCCAGGTGGAAACAAGACGACAGGTGTCTGTCATCCAG

GGACTCAGGGTGTACAGGGGGGTCGCGAGCATGAGAAGCCAGGTTGCGGTCAGGGTGGAC

AGGTGGGCCGCGAGCATGAGAAGACAGGTTTCGGTCATCCAGGGACTCAGGGTGGACAGG

TGGGCAGCGAGCAGGAGAAGAAGGGTATGATTGAGAAGATAAAGGAGAAGCTGCCAGGTC

ACAAGTAGGATGTGTGTCCACCATACACGTGGAGTCTACACCTACGTGTCTTATGTTACT

TATAATACGCATGGAGTGTGTCCGGAGTCTGTAATAATCTGCGCGTATCTGTTATGTC

>SCYY86

AGCCGTTGGATCTAGTTGAAAATGTCAGAGACGCGTGATGAGTATGGCAACCAGGTTCGC

CAAACCGACGAGTATGGGAACCCGATTCAGCATAGTGGCACCGGGACAAAGCCCGGTTCG

GGCATACATGGTGGGGGCCATGGGATTGGCACAGGTGGTGGTGGTGGACAAGGCAAGCTC

CACCGTCCAGGCTCCGGCTCTTCCTCTGTAAGTATATTCGATGTTTCTCAAGGGTCATTT

CACTTGGTATATCGTGTTGGGATGACTAGATTAGTTGATTAACGGTTGTGATTGTAAATC

AGTGATATGGGCTTCTGTGTACCTTATTTCACAATGATAATCAATTGATACGCTGGATCG

TGATGAGCGGACCGGATGAGCCGTCCATCTTACAGGCTTTTTAAAATAGAATTATAGGGT

GGGTGGTATGAGGTCCACCAAGCCTGTAACATGGAATTACAATCATAGCCGTTAATCGGT

TCATCTCATCCGCTCATCGTGATCCAGCATGTCAAACAGAGATGGGTTTCTGGAGAAATG

GATGTATGTAAGCCCACAAATCACAAAAAAAATCTGACATAATGATGGAGAGAATGCAAG

GAGTGTAAGTGAATCTACGCCGTTAAATGGATTTCATTGTACTTAATTTCAATTTCGTTT

TGTCAAATATTTTGTAGGACGAGGATGATGGACAAGGTGGGCGGAGGAAGAAGGGTTTGA

CGGAGAAGATCAAAGAGAAGCTGCCAGGTGGAAACAAGACGACAGGTGTCTGTCATCCAG

GGACTCAGGGTGTACAGGGGGGTCGCGAGCATGAGAAGCCAGGTTGCGGTCAGGGTGGAC

AGGTGGGCCGCGAGCATGAGAAGACAGGTTTCGGTCATCCAGGGACTCAGGGTGGACAGG

TGGGCAGCGAGCAGGAGAAGAAGGGTATGATTGAGAAGATAAAGGAGAAGCTGCCAGGTC

ACAAGTAGGATGTGTGTCCACCATACACGTGGAGTCTACACCTACGTGTCTTATGTTACT

TATAATACGCATGGAGTGTGTCCGGAGTCTGTAATAATCTGCGCGTATCTGTTATGTC

>SCYY518

AGCCGTTGGATCTAGTTGAAAATGTCAGAGACGCGTGATGAGTATGGCAACCAGGTTCGC

CAAACCGACGAGTATGGGAACCCGATTCAGCATACTGGCACCGGGACAAAGCCCGGTTCG

GGCATACATGGTGGGGGCCATGGGATTGGCACAGGTGGTGGTGGTGGACAAGGCAAGCTC

CACCGTCCAGGCTCCGGTTCTTCCTCTGTAAGTATATTCGATGTTTCTCAAGGGTCATTT

CACTTGGTATATAGTGTTGGGATGACTAGATTAGTTGATTAACGGTTGTGATTGTAAATC

AGTGATATGGGCTTCTATGTACCTTATTTCACAATGATAATCAATTGATACGCTGGATCG

TGATGAGCGGACCGGATGAGCCGTCCATCTTACAGGCTTTTTAAAATAGAATTATAGGGT

GGGTGGTATGAGGTCTACCAAGCCTGTAACATGGAATTACAATCATAGCCGTTAATCGGT

TCATCTCATCCGCTCATCGTGATGCAGCATGTCAAACAGAGATGGGTTTATGGAGAAATG

GATGTATGTAAGCCAACAAATCACAAAAAAAATCTGACATAATGATGGAGAGAATGCAAG

GAGTGTAAGTGAATCTACGCCGTTAAATGGATTTCATTGTACTTAATTTCGTTTTGTCAA

ATATTTTGTAGGACGAGGATGATGGACAAGGTGGGCGTAGGAAGAAGGGTTTGACGGAGA

AGATCAAAGAGAAGCTGCCAGGTGGAAACAAGACGACAGGTGTCTGTCATCCAGGGACTC

AGGGTGTACAGGGGGGTCGCGAGCATGAGAAGCCAGGTTGCGGTCAGGGTGGACAGGTGG

GCCGCGAGCATGAGAAGACAGGTTTCGGTCATCCAGGAACTCAGGGTGGACAGGTGGGCA

GCGAGCAGGAGAAGAAGGGTATGATTGAGAAGATCAAGGAGAAGCTGCCAGGTCACAAGT

AGGATGTGTGTCCACCATACACGTGGAGCCTACATCTACGTGTCTTATGTTACTTATAAT

ACGCATGGAGTGTGTCCGGAGTCTGTAATAATCTGCGCGTATCTGTTATGTC

>SCYY528

AGCCGTTGGATCTAGTTGAAAATGTCAGAGACGCGTGATGAGTATGGCAACCAGGTTCGC

CAAACCGACGAGTATGGGAACCCGATTCAGCATACTGGCACCGGGACAAAGCCCGGTTCG

GGCATACATGGTGGGGGCCATGGGATTGGCACAGGTGGTGGTGGTGGACAAGGCAAGCTC

CACCGTCCAGGCTCCGGTTCTTCCTCTGTAAGTATATTCGATGTTTCTCAAGGGTCATTT

CACTTGGTATATAGTGTTGGGATGACTAGATTAGTTGATTAACGGTTGTGATTGTAAATC

AGTGATATGGGCTTCTATGTACCTTATTTCACAATGATAATCAATTGATACGCTGGATCG

TGATGAGCGGACCGGATGAGCCGTCCATCTTACAGGCTTTTTAAAATAGAATTATAGGGT

GGGTGGTATGAGGTCTACCAAGCCTGTAACATGGAATTACAATCATAGCCGTTAATCGGT

TCATCTCATCCGCTCATCGTGATGCAGCATGTCAAACAGAGATGGGTTTATGGAGAAATG

GATGTATGTAAGCCAACAAATCACAAAAAAAATCTGACATAATGATGGAGAGAATGCAAG

GAGTGTAAGTGAATCTACGCCGTTAAATGGATTTCATTGTACTTAATTTCGTTTTGTCAA

ATATTTTGTAGGACGAGGATGATGGACAAGGTGGGCGTAGGAAGAAGGGTTTGACGGAGA

AGATCAAAGAGAAGCTGCCAGGTGGAAACAAGACGACAGGTGTCTGTCATCCAGGGACTC

AGGGTGTACAGGGGGGTCGCGAGCATGAGAAGCCAGGTTGCGGTCAGGGTGGACAGGTGG

GCCGCGAGCATGAGAAGACAGGTTTCGGTCATCCAGGAACTCAGGGTGGACAGGTGGGCA

GCGAGCAGGAGAAGAAGGGTATGATTGAGAAGATCAAGGAGAAGCTGCCAGGTCACAAGT

AGGATGTGTGTCCACCATACACGTGGAGCCTACATCTACGTGTCTTATGTTACTTATAAT

ACGCATGGAGTGTGTCCGGAGTCTGTAATAATCTGCGCGTATCTGTTATGTC

>SCYY531

AGCCGTTGGATCTAGTTGAAAATGTCAGAGACGCGTGATGAGTATGGCAACCAGGTTCGC

CAAACCGACGAGTATGGGAACCCGATTCAGCATACTGGCACCGGGACAAAGCCCGGTTCG

GGCATACATGGTGGGGGCCATGGGATTGGCACAGGTGGTGGTGGTGGACAAGGCAAGCTC

CACCGTCCAGGCTCCGGTTCTTCCTCTGTAAGTATATTCGATGTTTCTCAAGGGTCATTT

CACTTGGTATATAGTGTTGGGATGACTAGATTAGTTGATTAACGGTTGTGATTGTAAATC

AGTGATATGGGCTTCTATGTACCTTATTTCACAATGATAATCAATTGATACGCTGGATCG

TGATGAGCGGACCGGATGAGCCGTCCATCTTACAGGCTTTTTAAAATAGAATTATAGGGT

GGGTGGTATGAGGTCTACCAAGCCTGTAACATGGAATTACAATCATAGCCGTTAATCGGT

TCATCTCATCCGCTCATCGTGATGCAGCATGTCAAACAGAGATGGGTTTATGGAGAAATG

GATGTATGTAAGCCAACAAATCACAAAAAAAATCTGACATAATGATGGAGAGAATGCAAG

GAGTGTAAGTGAATCTACGCCGTTAAATGGATTTCATTGTACTTAATTTCGTTTTGTCAA

ATATTTTGTAGGACGAGGATGATGGACAAGGTGGGCGTAGGAAGAAGGGTTTGACGGAGA

AGATCAAAGAGAAGCTGCCAGGTGGAAACAAGACGACAGGTGTCTGTCATCCAGGGACTC

AGGGTGTACAGGGGGGTCGCGAGCATGAGAAGCCAGGTTGCGGTCAGGGTGGACAGGTGG

GCCGCGAGCATGAGAAGACAGGTTTCGGTCATCCAGGAACTCAGGGTGGACAGGTGGGCA

GCGAGCAGGAGAAGAAGGGTATGATTGAGAAGATCAAGGAGAAGCTGCCAGGTCACAAGT

AGGATGTGTGTCCACCATACACGTGGAGCCTACATCTACGTGTCTTATGTTACTTATAAT

ACGCATGGAGTGTGTCCGGAGTCTGTAATAATCTGCGCGTATCTGTTATGTC

>YNXC475

AGCCGTTGGATCTAGTTGAAAATGTCAGAGACGCGTGATGAGTATGGCAACCAGGTTCGC

CAAACCGACGAGTATGGGAACCCGATTCAGCATACTGGCACCGGGACAAAGCCCGGTTCG

GGCATACATGGTGGGGGCCATGGGATTGGCACAGGTGGTGGTGGTGGACAAGGCAAGCTC

CACCGTCCAGGCTCCGGCTCTTCCTCTGTAAGTATATTCCCTGTTTCTCAAGGGTCATTT

CACTTGGTATATAGTGTTGGGATGACTAGATTAGTTGATTAACGGTTGTGATTGTAAATC

AGTGATATGGGCTTCTATGTACCTTATTTCACAATGATAATCAATTGATACGCTGGATCG

TGATGAGCGGACCGGATGAGCCGTCCATCTTACAGGCTTTTTAAAATAGAATTATAGGGT

GGGTGGTATGAGGTCTACCAAGCCTGTAACATGGAATTACAATCATAGCCGTTAATCGGT

TCATCTCATCCGCTCATCGTGATCCAGCATGTCAAACAGAGATGGGTTTATGGAGAAATG

GATGTATGTAAGCCAACAAATCACAAAAAAAATCTGACATAATGATGGAGAGAATGCAAG

GAGTGTAAGTGAATCTACGCCGTTAAATGGATTTCATTGTACTTAATTTCAATTTCGTCT

TGTCAAATATTTTGTAGGACGAGGATGATGGACAAGGTGGGCGGAGGAAGAAGGGTTTGA

CGGAGAAGATCAAAGAGAAGCTGCCAGGTGGAAACAAGACGACAGGTGTCTGTCATCCAG

GGACTCAGGGAGTACAGGGGGGTCGCGAGCATGAGAAGCCAGGTTGCGGTCAGGGTGGAC

AGGTGGGCCGCGAGCATGAGAAGACAGGTTTCGGTCATCCAGGGACTCAGGGTGGACAGG

TGGGCAGCGAGCAGGAGAAGAAGGGTATGATTGAGAAGATCAAGGAGAAGCTGCCAGGTC

ACAAGTAGGATGTGTGTCCACCATACACGTGGAGTCTACACCTACGTGTCTTATGTTACT

TATAATACGCATGGAGTGTGTCCGGAGTCTGTAATAATCTGCGCGTATCTGTTATGTC

>YNXC478

AGCCGTTGGATCTAGTTGAAAATGTCAGAGACGCGTGATGAGTATGGCAACCAGGTTCGC

CAAACCGACGAGTATGGGAACCCGATTCAGCATACTGGCACCGGGACAAAGCCCGGTTCG

GGCATACATGGTGGGGGCCATGGGATTGGCACAGGTGGTGGTGGTGGACAAGGCAAGCTC

CACCGTCCAGGCTCCGGCTCTTCCTCTGTAAGTATATTCGATGTTTCTCAAGGGTCATTT

CACTTGGTATATAGTGTTGGGATGACTAGATTAGTTGATTAACGGTTGTGATTGTAAATC

AGTGATATGGGCTTCTATGTACCTTATTTCACAATGATAATCAATTGATACGCTGGATCG

TGATGAGCGGACCGGATGAGCCGTCCATCTTACAGGCTTTTTAAAATAGAATTATAGGGT

GGGTGGTATGAGGTCTACCAAGCCTGTAACATGGAATTACAATCATAGCCGTTAATCGGT

TCATCTCATCCGCTCATCGTGATCCAGCATGTCAAACAGAGATGGGTTTATGGAGAAATG

GATGTATGTAAGCCAACAAATCACAAAAAAAATCTGACATAATGATGGAGAGAATGCAAG

GAGTGTAAGATCTACGCCGTTAAATGGATTTCATTGTACTTAATTTCATTTTGTCAAATA

TTTTGTAGGACGAGGATGATGGACAAGGTGGGCGTAGGAAGAAGGGTTTGACGGAGAAGA

TCAAAGAGAAGCTGCCAGGTGGAAACAAGACGACAGGTGTCTGTCATCCAGGGACTCAGG

GTGTACAGGGGGGTCGCGAGCATGAGAAGCAAGGTTGCGGTCAGGGTGGACAGGTGGGCC

GCGAGCATGAGAAGACAGGTTTCGGTCATCCAGGGACTCAGGGTGGACAGGTGGGCAGCG

AGCAGGAGAAGAAGGGTATGATTGAGAAGATCAAGGAGAAGCTGCCAGGTCACAAGTAGG

ATGTGTGTCCACCATACACGTGGAGTCTACACCTACGTGTCTTATGTTACTTATAATACG

CATGGAGTGTGTCCGGAGTCTGTAATAATCTGCGCGTATCTGTTATGTC

>YNXC4722

AGCCGTTGGATCTAGTTGAAAATGTCAGAGACGCGTGATGAGTATGGCAACCAGGTTCGC

CAAACCGACGAGTATGGGAACCCGATTCAGCATACTGGCACCGGGACAAAGCCCGGTTCG

GGCATACATGGTGGGGGCCATGGGATTGGCACAGGTGGTGGTGGTGGACAAGGCAAGCTC

CACCGTCCAGGCTCCGGCTCTTCCTCTGTAAGTATATTCCCTGTTTCTCAAGGGTCATTT

CACTTGGTATATAGTGTTGGGATGACTAGATTAGTTGATTAACGGTTGTGATTGTAAATC

AGTGATATGGGCTTCTATGTACCTTATTTCACAATGATAATCAATTGATACGCTGGATCG

TGATGAGCGGACCGGATGAGCCGTCCATCTTACAGGCTTTTTAAAATAGAATTATAGGGT

GGGTGGTATGAGGTCTACCAAGCCTGTAACATGGAATTACAATCATAGCCGTTAATCGGT

TCATCTCATCCGCTCATCGTGATCCAGCATGTCAAACAGAGATGGGTTTATGGAGAAATG

GATGTATGTAAGCCAACAAATCACAAAAAAAATCTGACATAATGATGGAGAGAATGCAAG

GAGTGTAAGATCTACGCCGTTAAATGGATTTCATTGTACTTAATTTCATTTTGTCAAATA

TTTTGTAGGACGAGGATGATGGACAAGGTGGGCGTAGGAAGAAGGGTTTGACGGAGAAGA

TCAAAGAGAAGCTGCCAGGTGGAAACAAGACGACAGGTGTCTGTCATCCAGGGACTCAGG

GTGTACAGGGGGGTCGCGAGCATGAGAAGCAAGGTTGCGGTCAGGGTGGACAGGTGGGCC

GCGAGCATGAGAAGACAGGTTTCGGTCATCCAGGGACTCAGGGTGGACAGGTGGGCAGCG

AGCAGGAGAAGAAGGGTATGATTGAGAAGATCAAGGAGAAGCTGCCAGGTCACAAGTAGG

ATGTGTGTCCACCATACACGTGGAGTCTACACCTACGTGTCTTATGTTACTTATAATACG

CATGGAGTGTGTCCGGAGTCTGTAATAATCTGCGCGTATCTGTTATGTC

>YNXC93

AGCCGTTGGATCTAGTTGAAAATGTCAGAGACGCGTGATGAGTATGGCAACCAGGTTCGC

CAAACCGACGAGTATGGGAACCCGATTCAGCATACTGGCACCGGGACAAAGCCCGGTTCG

GGCATACATGGTGGGGGCCATGGGATTGGCACAGGTGGTGGTGGTGGACAAGGCAAGCTC

CACCGTCCAGGCTCCGGCTCTTCCTCTGTAAGTATATTCGATGTTTCTCAAGGGCCATTT

CACTTGGTATATCGTGTTGGGATGACTAGATTAGTTGATTAACGGTTGTGATTGTAAATC

AGTGATATGGGCTTCTGTGTACCTTATTTCACAATGATAATCAATTGATACGCTGGATCG

TGATGAGCGGACCGGATGAGCCGTCCATCTTACTGGCTTTTTAAAATAGAATTATAGGGT

GGGTGGTATGAGGTCTACCAAGCCTGTAACATGGAATTACAATCATAGCTGTTAATCGGT

TCATCTCATCCGCTCATCGTGATCCAGCATGTCAAACAGAGATGGGTTTCTGGAGAAATG

GATGTATGTAAGCCCACAAATCACAAAAAAAAATCTGACATAATGATGGAGAGAATGCAA

GGAGTGTAAGTGAATCTACGTCGTTAAATGGATTTCATTGTACTTAACTTCGTTTTGTCA

AATATTTTGTAGGACGAGGATGATGGACAAGGTGGGCGTAGGAAGAAGGGTTTGACGGAG

AAGATAAAAGAGAAGCTGCCAGGTGGAAACAAGACGACAGGTGTCTGTCATCCAGGGACT

CAGGGTGTACAGGGGGGTCGCGAGCATGAGAAGCCAGGTTGCGGTCAGGGTGGACAGGTG

GGCCGCGAGCATGAGAAGACAGGTTTCGGTCATCCAGGGACTCAGGGTGGACAGGTGGGC

AGCGAGCAGGAGAAGAAGGGTATGATTGAGAAGATCAAGGAGAAGCTACCAGGTCACAAG

TAGGATGTGTGTCCACCATACACGTGGAGTCTGCATCTACGTGTCTTATGTTACTTATAA

TACGCATGGAGTGTGTCCGGAGTCTGTAATAATCTGCGCGTATCTGTTATGTC

>YNXC92

AGCCGTTGGATCTAGTTGAAAATGTCAGAGACGCGTGATGAGTATGGCAACCAGGTTCGC

CAAACCGACGAGTATGGGAACCCGATTCAGCATACTGGCACCGGGACAAAGCCCGGTTCG

GGCATACATGGTGGGGGCCATGGGATTGGCACAGGTGGTGGTGGTGGACAAGGCAAGCTC

CACCGCCCAGGCTCCGGCTCTTCCTCTGTAAGTATATTCGATGTTTCTCAAGGGCCATTT

CACTTGGTATATCGTGTTGGGATGACTAGATTAGTTGATTAACGGTTGTGATTGTAAATC

AGTGATATGGGCTTCTGTGTACCTTATTTCACAATGATAATCAATTGATACGCTGGATCG

TGATGAGCGGACCGGATGAGCCGTCCATCTTACAGGCTTTTTAAAATAGAATTATAGGGT

GGGTGGTATGAGGTCTACCAAGCCTGTAACATGGAATTACAATCATAGCCGTTAATCGGT

TCATCTCATCCGCTCATCGTGATCCAGCATGTCAAACAGAGATGGGTTTATGGAGAAATG

GATGTATGTAAGCCAACAAATCACAAAAAAAATCTGACATAATGATGGAGAGAATGCAAG

GAGTGTAAGTGAATCTACGCCGTTAAATGGATTTCATTGTAATTAATTTCGTTTTGTCAA

ATATTTTGTAGGACGAGGATGATGGACAAGGTGGGCGTAGGAAGAAGGGTTTGACGGAGA

AGATCAAAGAGAAGCTGCCAGGTGGAAACAAGACGACAGGTGCCTGTCATCCAGGGACTC

AGGGAGTACAGGGGGGTCGCGAGCATGAGAAGCCAGGTTGCGGTCAGGGTGGACAGGTGG

GCCGCGAGCATGAGAAGACAGGTTTCGGTCATCCAGGGACTCAGGGTGGACAGGTGGGCA

GCGAGCAGGAGAAGAAGGGTATGATTGAGAAGATCAAGGAGAAGCTGCCAGGTCACAAGT

AGGATGTGTGTCCACCATACACGTGGAGTCTACACCTACGTGTCTTATGTTACTTATAAT

ACGCATGGAGTGTGTCCGGAGTCTGTAATAATCTGCGCGTATCTGTTATGTC

>YNXC95

AGCCGTTGGATCTAGTTGAAAATGTCAGAGACGCGTGATGAGTATGGCAACCAGGTTCGC

CAAACCGACGAGTATGGGAACCCGATTCAGCATACTGGCACCGGGACAAAGCCCGGTTCG

GGCATACATGGTGGGGGCCATGGGATTGGCACAGGTGGTGGTGGTGGACAAGGCAAGCTC

CACCGCCCAGGCTCCGGCTCTTCCTCTGTAAGTATATTCGATGTTTCTCAAGGGCCATTT

CACTTGGTATATCGTGTTGGGATGACTAGATTAGTTGATTAACGGTTGTGATTGTAAATC

AGTGATATGGGCTTCTGTGTACCTTATTTCACAATGATAATCAATTGATACGCTGGATCG

TGATGAGCGGACCGGATGAGCCGTCCATCTTACAGGCTTTTTAAAATAGAATTATAGGGT

GGGTGGTATGAGGTCTACCAAGCCTGTAACATGGAATTACAATCATAGCCGTTAATCGGT

TCATCTCATCCGCTCATCGTGATCCAGCATGTCAAACAGAGATGGGTTTATGGAGAAATG

GATGTATGTAAGCCAACAAATCACAAAAAAAATCTGACATAATGATGGAGAGAATGCAAG

GAGTGTAAGTGAATCTACGCCGTTAAATGGATTTCATTGTAATTAATTTCGTTTTGTCAA

ATATTTTGTAGGACGAGGATGATGGACAAGGTGGGCGTAGGAAGAAGGGTTTGACGGAGA

AGATCAAAGAGAAGCTGCCAGGTGGAAACAAGACGACAGGTGCCTGTCATCCAGGGACTC

AGGGAGTACAGGGGGGTCGCGAGCATGAGAAGCCAGGTTGCGGTCAGGGTGGACAGGTGG

GCCGCGAGCATGAGAAGACAGGTTTCGGTCATCCAGGGACTCAGGGTGGACAGGTGGGCA

GCGAGCAGGAGAAGAAGGGTATGATTGAGAAGATCAAGGAGAAGCTGCCAGGTCACAAGT

AGGATGTGTGTCCACCATACACGTGGAGTCTACACCTACGTGTCTTATGTTACTTATAAT

ACGCATGGAGTGTGTCCGGAGTCTGTAATAATCTGCGCGTATCTGTTATGTC

>YNXC715

AGCCGTTGGATCTAGTTGAAAATGTCAGAGACGCGTGATGAGTATGGCAACCAGGTTCGC

CAAACCGACGAGTATGGGAACCCGATTCAGCATACTGGCACCGGGACAAAGCCCGGTTCG

GGCATACATGGTGGGGGCCATGGGATTGGCACAGGTGGTGGTGGTGGACAAGGCAAGCTC

CACCGTCCAGGCTCCGGCTCTTCCTCTGTAAGTATATTCGATGTTTCTCAAGGGCCATTT

CACTTGGTATATCGTGTTGGGATGACTAGATTAGTTGATTAACGGTTGTGATTGTAAATC

AGTGATATGGGCTTCTGTGTACCTTATTTCACAATGATAATCAATTGATACGCTGGATCG

TGATGAGCGGACCGGATGAGCCGTCCATCTTACTGGCTTTTTAAAATAGAATTATAGGGT

GGGTGGTATGAGGTCTACCAAGCCTGTAACATGGAATTACAATCATAGCTGTTAATCGGT

TCATCTCATCCGCTCATCGTGATCCAGCATGTCAAACAGAGATGGGTTTCTGGAGAAATG

GATGTATGTAAGCCCACAAATCACAAAAAAAAATCTGACATAATGATGGAGAGAATGCAA

GGAGTGTAAGTGAATCTACGCCGTTAAATGGATTTCATTGTACTTAACTTCGTTTTGTCA

AATATTTTGTAGGACGAGGATGATGGACAAGGTGGGCGTAGGAAGAAGGGTTTGACGGAG

AAGATAAAAGAGAAGCTGCCAGGTGGAAACAAGACGACAGGTGTCTGTCATCCAGGGACT

CAGGGTGTACAGGGGGGTCGCGAGCATGAGAAGCCAGGTTGCGGTCAGGGTGGACAGGTG

GGCCGCGAGCATGAGAAGACAGGTTTCGGTCATCCAGGGACTCAGGGTGGACAGGTGGGC

AGCGAGCAGGAGAAGAAGGGTATGATTGAGAAGATCAAGGAGAAGCTACCAGGTCACAAG

TAGGATGTGTGTCCACCATACACGTGGAGTCTGCATCTACGTGTCTTATGTTACTTATAA

TACGCATGGAGTGTGTCCGGAGTCTGTAATAATCTGCGCGTATCTGTTATGTC

>YNXC7113

AGCCGTTGGATCTAGTTGAAAATGTCAGAGACGCGTGATGAGTATGGCAACCAGGTTCGC

CAAACCGACGAGTATGGGAACCCGATTCAGCATACTGGCACCGGGACAAAGCCCGGTTCG

GGCATACATGGTGGGGGCCATGGGATTGGCACAGGTGGTGGTGGTGGACAAGGCAAGCTC

CACCGTCCAGGCTCCGGCTCTTCCTCTGTAAGTATATTCGATGTTTCTCAAGGGCCATTT

CACTTGGTATATCGTGTTGGGATGACTAGATTAGTTGATTAACGGTTGTGATTGTAAATC

AGTGATATGGGCTTCTGTGTACCTTATTTCACAATGATAATCAATTGATACGCTGGATCG

TGATGAGCGGACCGGATGAGCCGTCCATCTTACTGGCTTTTTAAAATAGAATTATAGGGT

GGGTGGTATGAGGTCTACCAAGCCTGTAACATGGAATTACAATCATAGCTGTTAATCGGT

TCATCTCATCCGCTCATCGTGATCCAGCATGTCAAACAGAGATGGGTTTCTGGAGAAATG

GATGTATGTAAGCCCACAAATCACAAAAAAAAATCTGACATAATGATGGAGAGAATGCAA

GGAGTGTAAGTGAATCTACGCCGTTAAATGGATTTCATTGTACTTAACTTCGTTTTGTCA

AATATTTTGTAGGACGAGGATGATGGACAAGGTGGGCGTAGGAAGAAGGGTTTGACGGAG

AAGATAAAAGAGAAGCTGCCAGGTGGAAACAAGACGACAGGTGTCTGTCATCCAGGGACT

CAGGGTGTACAGGGGGGTCGCGAGCATGAGAAGCCAGGTTGCGGTCAGGGTGGACAGGTG

GGCCGCGAGCATGAGAAGACAGGTTTCGGTCATCCAGGGACTCAGGGTGGACAGGTGGGC

AGCGAGCAGGAGAAGAAGGGTATGATTGAGAAGATCAAGGAGAAGCTACCAGGTCACAAG

TAGGATGTGTGTCCACCATACACGTGGAGTCTGCATCTACGTGTCTTATGTTACTTATAA

TACGCATGGAGTGTGTCCGGAGTCTGTAATAATCTGCGCGTATCTGTTATGTC

>YNXC7112

AGCCGTTGGATCTAGTTGAAAATGTCAGAGACGCGTGATGAGTATGGCAACCAGGTTCGC

CAAACCGACGAGTATGGGAACCCGATTCAGCATACTGGCACCGGGACAAAGCCCGGTTCG

GGCATACATGGTGGGGGCCATGGGATTGGCACAGGTGGTGGTGGTGGACAAGGCAAGCTC

CACCGTCCAGGCTCCGGCTCTTCCTCTGTAAGTATATTCGATGTTTCTCAAGGGCCATTT

CACTTGGTATATCGTGTTGGGATGACTAGATTAGTTGATTAACGGTTGTGATTGTAAATC

AGTGATATGGGCTTCTGTGTACCTTATTTCACAATGATAATCAATTGATACGATGGATCG

TGATGAGCGGACCGGATGAGCCGTCCATCTTACTGGCTTTTTAAAATAGAATTATAGGGT

GGGTGGTATGAGGTCCACCAAGCCTGTAACATGGAATTACAATCATAGCCGTTAATCGGT

TCATCTCATCCGCTCATCGTGATCCAGCATGTCAAACAGAGATGGGTTTCTGGAGAAATG

GATGTATGTAAGCCCACAAATCACAAAAAAAATCTGACATAATGATGGAGAGAATGCAAG

GAGTGTAAGTGAATCTACGCCGTTAAATGGATTTCATTGTACTTAATTTCAATTTCGTTT

TGTCAAATATTTTGTAGGACGAGGATGATGGACAAGGTGGGCGGAGGAAGAAGGGTTTGA

CGGAGAAGATCAAAGAGAAGCTGCCAGGTGGAAACAAGACGACAGGTGTCTGTCATCCAG

GGACTCAGGGTGTACAGGGGGGTCGCGAGCATGAGAAGCCAGGTTGCGGTCAGGGTGGAC

AGGTGGGCCGCGAGCATGAGAAGACAGGTTTCGGTCATCCAGGGACTCAGGGTGGACAGG

TGGGCAGCGAGCAGGAGAAGAAGGGTATGATTGAGAAGATAAAGGAGAAGCTGCCAGGTC

ACAAGTAGGATGTGTGTCCACCATACACGTGGAGTCTACACCTACGTGTCTTATGTTACT

TATAATACGCATGGAGTGTGTCCGGAGTCTGTAATAATCTGCGCGTATCTGTTATGTC

>YNXC1611

AGCCGTTGGATCTAGTTGAAAATGTCAGAGACGCGTGATGAGTATGGCAACCAGGTTCGC

CAAACCGACGAGTATGGGAACCCGATTCAGCATAGTGGCACCGGGACAAAGCCCGGTTCG

GGCATACATGGTGGGGGCCATGGGATTGGCACAGGTGGTGGTGGTGGACAAGGCAAGCTC

CACCGTCCAGGCTCCGGCTCTTCCTCTGTAAGTATATTCGATGTTTCTCAAGGGTCATTT

CACTTGGTATATCGTGTTGGGATGACTAGATTAGTTGATTAACGGTTGTGATTGTAAATC

AGTGATATGGGCTTCTGTGTACCTTATTTCACAATGATAATCAATTGATACGCTGGATCG

TGATGAGCGGACCGGATGAGCCGTCCATCTTACAGGCTTTTTAAAATAGAATTATAGGGT

GGGTGGTATGAGGTCCACCAAGCCTGTAACATGGAATTACAATCATAGCCGTTAATCGGT

TCATCTCATCCGCTCATCGTGATCCAGCATGTCAAACAGAGATGGGTTTCTGGAGAAATG

GATGTATGTAAGCCCACAAATCACAAAAAAAATCTGACATAATGATGGAGAGAATGCAAG

GAGTGTAAGTGAATCTACGCCGTTAAATGGATTTCATTGTACTTAATTTCAATTTCGTTT

TGTCAAATATTTTGTAGGACGAGGATGATGGACAAGGTGGGCGGAGGAAGAAGGGTTTGA

CGGAGAAGATCAAAGAGAAGCTGCCAGGTGGAAACAAGACGACAGGTGTCTGTCATCCAG

GGACTCAGGGTGTACAGGGGGGTCGCGAGCATGAGAAGCCAGGTTGCGGTCAGGGTGGAC

AGGTGGGCCGCGAGCATGAGAAGACAGGTTTCGGTCATCCAGGGACTCAGGGTGGACAGG

TGGGCAGCGAGCAGGAGAAGAAGGGTATGATTGAGAAGATAAAGGAGAAGCTGCCAGGTC

ACAAGTAGGATGTGTGTCCACCATACACGTGGAGTCTACACCTACGTGTCTTATGTTACT

TATAATACGCATGGAGTGTGTCCGGAGTCTGTAATAATCTGCGCGTATCTGTTATGTC

>YNXC1616

AGCCGTTGGATCTAGTTGAAAATGTCAGAGACGCGTGATGAGTATGGCAACCAGGTTCGC

CAAACCGACGAGTATGGGAACCCGATTCAGCATAGTGGCACCGGGACAAAGCCCGGTTCG

GGCATACATGGTGGGGGCCATGGGATTGGCACAGGTGGTGGTGGTGGACAAGGCAAGCTC

CACCGTCCAGGCTCCGGCTCTTCCTCTGTAAGTATATTCGATGTTTCTCAAGGGTCATTT

CACTTGGTATATCGTGTTGGGATGACTAGATTAGTTGATTAACGGTTGTGATTGTAAATC

AGTGATATGGGCTTCTGTGTACCTTATTTCACAATGATAATCAATTGATACGCTGGATCG

TGATGAGCGGACCGGATGAGCCGTCCATCTTACAGGCTTTTTAAAATAGAATTATAGGGT

GGGTGGTATGAGGTCCACCAAGCCTGTAACATGGAATTACAATCATAGCCGTTAATCGGT

TCATCTCATCCGCTCATCGTGATCCAGCATGTCAAACAGAGATGGGTTTCTGGAGAAATG

GATGTATGTAAGCCCACAAATCACAAAAAAAATCTGACATAATGATGGAGAGAATGCAAG

GAGTGTAAGTGAATCTACGCCGTTAAATGGATTTCATTGTACTTAATTTCAATTTCGTTT

TGTCAAATATTTTGTAGGACGAGGATGATGGACAAGGTGGGCGGAGGAAGAAGGGTTTGA

CGGAGAAGATCAAAGAGAAGCTGCCAGGTGGAAACAAGACGACAGGTGTCTGTCATCCAG

GGACTCAGGGTGTACAGGGGGGTCGCGAGCATGAGAAGCCAGGTTGCGGTCAGGGTGGAC

AGGTGGGCCGCGAGCATGAGAAGACAGGTTTCGGTCATCCAGGGACTCAGGGTGGACAGG

TGGGCAGCGAGCAGGAGAAGAAGGGTATGATTGAGAAGATAAAGGAGAAGCTGCCAGGTC

ACAAGTAGGATGTGTGTCCACCATACACGTGGAGTCTACACCTACGTGTCTTATGTTACT

TATAATACGCATGGAGTGTGTCCGGAGTCTGTAATAATCTGCGCGTATCTGTTATGTC

>YNXC1619

AGCCGTTGGATCTAGTTGAAAATGTCAGAGACGCGTGATGAGTATGGCAACCAGGTTCGC

CAAACCGACGAGTATGGGAACCCGATTCAGCATAGTGGCACCGGGACAAAGCCCGGTTCG

GGCATACATGGTGGGGGCCATGGGATTGGCACAGGTGGTGGTGGTGGACAAGGCAAGCTC

CACCGTCCAGGCTCCGGCTCTTCCTCTGTAAGTATATTCGATGTTTCTCAAGGGTCATTT

CACTTGGTATATCGTGTTGGGATGACTAGATTAGTTGATTAACGGTTGTGATTGTAAATC

AGTGATATGGGCTTCTGTGTACCTTATTTCACAATGATAATCAATTGATACGCTGGATCG

TGATGAGCGGACCGGATGAGCCGTCCATCTTACAGGCTTTTTAAAATAGAATTATAGGGT

GGGTGGTATGAGGTCCACCAAGCCTGTAACATGGAATTACAATCATAGCCGTTAATCGGT

TCATCTCATCCGCTCATCGTGATCCAGCATGTCAAACAGAGATGGGTTTCTGGAGAAATG

GATGTATGTAAGCCCACAAATCACAAAAAAAATCTGACATAATGATGGAGAGAATGCAAG

GAGTGTAAGTGAATCTACGCCGTTAAATGGATTTCATTGTACTTAATTTCAATTTCGTTT

TGTCAAATATTTTGTAGGACGAGGATGATGGACAAGGTGGGCGGAGGAAGAAGGGTTTGA

CGGAGAAGATCAAAGAGAAGCTGCCAGGTGGAAACAAGACGACAGGTGTCTGTCATCCAG

GGACTCAGGGTGTACAGGGGGGTCGCGAGCATGAGAAGCCAGGTTGCGGTCAGGGTGGAC

AGGTGGGCCGCGAGCATGAGAAGACAGGTTTCGGTCAGGGTGGACAGGTGGGCCGCGAGC

AGGAGAAGAAGGGTATGATTGAGAAGATAAAGGAGAAGCTGCCAGGTCACAAGTAGGATG

TGTGTCCACCATACACGTGGAGTCTACACCTACGTGTCTTATGTTACTTATAATACGCAT

GGAGTGTGTCCGGAGTCTGTAATAATCTGCGCGTATCTGTTATGTC

>YNMG166

AGCCGTTGGATCTAGTTGAAAATGTCAGAGACGCGTGATGAGTATGGCAACCAGGTTCGC

CAAACCGACGAGTATGGGAACCCGATTCAGCATACTGGCACCGGGACAAAGCCCGGTTCG

GGCATACATGGTGGGGGCCATGGGATTGGCACAGGTGGTGGTGGTGGACAAGGCAAGCTC

CACCGCCCAGGCTCCGGCTCTTCCTCTGTAAGTATATTCGATGTTTCTCAAGGGCCATTT

CACTTGGTATATCGTGTTGGGATGACTAGATTAGTTGATTAACGGTTGTGATTGTAAATC

AGTGATATGGGCTTCTGTGTACCTTATTTCACAATGATAATCAATTGATACGCTGGATCG

TGATGAGCGGACCGGATGAGCCGTCCATCTTACAGGCTTTTTAAAATAGAATTATAGGGT

GGGTGGTATGAGGTCTACCAAGCCTGTAACATGGAATTACAATCATAGCCGTTAATCGGT

TCATCTCATCCGCTCATCGTGATCCAGCATGTCAAACAGAGATGGGTTTATGGAGAAATG

GATGTATGTAAGCCAACAAATCACAAAAAAAATCTGACATAATGATGGAGAGAATGCAAG

GAGTGTAAGTGAATCTACGCCGTTAAATGGATTTCATTGTAATTAATTTCGTTTTGTCAA

ATATTTTGTAGGACGAGGATGATGGACAAGGTGGGCGTAGGAAGAAGGGTTTGACGGAGA

AGATCAAAGAGAAGCTGCCAGGTGGAAACAAGACGACAGGTGCCTGTCATCCAGGGACTC

AGGGAGTACAGGGGGGTCGCGAGCATGAGAAGCCAGGTTGCGGTCAGGGTGGACAGGTGG

GCCGCGAGCATGAGAAGACAGGTTTCGGTCATCCAGGGACTCAGGGTGGACAGGTGGGCA

GCGAGCAGGAGAAGAAGGGTATGATTGAGAAGATCAAGGAGAAGCTGCCAGGTCACAAGT

AGGATGTGTGTCCACCATACACGTGGAGTCTACACCTACGTGTCTTATGTTACTTATAAT

ACGCATGGAGTGTGTCCGGAGTCTGTAATAATCTGCGCGTATCTGTTATGTC

>YNMG167

AGCCGTTGGATCTAGTTGAAAATGTCAGAGACGCGTGATGAGTATGGCAACCAGGTTCGC

CAAACCGACGAGTATGGGAACCCGATTCAGCATACTGGCACCGGGACAAAGCCCGGTTCG

GGCATACATGGTGGGGGCCATGGGATTGGCACAGGTGGTGGTGGTGGACAAGGCAAGCTC

CACCGCCCAGGCTCCGGCTCTTCCTCTGTAAGTATATTCGATGTTTCTCAAGGGCCATTT

CACTTGGTATATCGTGTTGGGATGACTAGATTAGTTGATTAACGGTTGTGATTGTAAATC

AGTGATATGGGCTTCTGTGTACCTTATTTCACAATGATAATCAATTGATACGCTGGATCG

TGATGAGCGGACCGGATGAGCCGTCCATCTTACAGGCTTTTTAAAATAGAATTATAGGGT

GGGTGGTATGAGGTCTACCAAGCCTGTAACATGGAATTACAATCATAGCCGTTAATCGGT

TCATCTCATCCGCTCATCGTGATCCAGCATGTCAAACAGAGATGGGTTTATGGAGAAATG

GATGTATGTAAGCCAACAAATCACAAAAAAAATCTGACATAATGATGGAGAGAATGCAAG

GAGTGTAAGTGAATCTACGCCGTTAAATGGATTTCATTGTAATTAATTTCGTTTTGTCAA

ATATTTTGTAGGACGAGGATGATGGACAAGGTGGGCGTAGGAAGAAGGGTTTGACGGAGA

AGATCAAAGAGAAGCTGCCAGGTGGAAACAAGACGACAGGTGCCTGTCATCCAGGGACTC

AGGGAGTACAGGGGGGTCGCGAGCATGAGAAGCCAGGTTGCGGTCAGGGTGGACAGGTGG

GCCGCGAGCATGAGAAGACAGGTTTCGGTCATCCAGGGACTCAGGGTGGACAGGTGGGCA

GCGAGCAGGAGAAGAAGGGTATGATTGAGAAGATCAAGGAGAAGCTGCCAGGTCACAAGT

AGGATGTGTGTCCACCATACACGTGGAGTCTACACCTACGTGTCTTATGTTACTTATAAT

ACGCATGGAGTGTGTCCGGAGTCTGTAATAATCTGCGCGTATCTGTTATGTC

>YNMG631

AGCCGTTGGATCTAGTTGAAAATGTCAGAGACGCGTGATGAGTATGGCAACCAGGTTCGC

CAAACCGACGAGTATGGGAACCCGATTCAGCATACTGGCACCGGGACAAAGCCCGGTTCG

GGCATACATGGTGGGGGCCATGGGATTGGCACAGGTGGTGGTGGTGGACAAGGCAAGCTC

CACCGCCCAGGCTCCGGCTCTTCCTCTGTAAGTATATTCGATGTTTCTCAAGGGCCATTT

CACTTGGTATATCGTGTTGGGATGACTAGATTAGTTGATTAACGGTTGTGATTGTAAATC

AGTGATATGGGCTTCTGTGTACCTTATTTCACAATGATAATCAATTGATACGCTGGATCG

TGATGAGCGGACCGGATGAGCCGTCCATCTTACAGGCTTTTTAAAATAGAATTATAGGGT

GGGTGGTATGAGGTCTACCAAGCCTGTAACATGGAATTACAATCATAGCCGTTAATCGGT

TCATCTCATCCGCTCATCGTGATCCAGCATGTCAAACAGAGATGGGTTTATGGAGAAATG

GATGTATGTAAGCCAACAAATCACAAAAAAAATCTGACATAATGATGGAGAGAATGCAAG

GAGTGTAAGTGAATCTACGCCGTTAAATGGATTTCATTGTAATTAATTTCGTTTTGTCAA

ATATTTTGTAGGACGAGGATGATGGACAAGGTGGGCGTAGGAAGAAGGGTTTGACGGAGA

AGATCAAAGAGAAGCTGCCAGGTGGAAACAAGACGACAGGTGCCTGTCATCCAGGGACTC

AGGGAGTACAGGGGGGTCGCGAGCATGAGAAGCCAGGTTGCGGTCAGGGTGGACAGGTGG

GCCGCGAGCATGAGAAGACAGGTTTCGGTCATCCAGGGACTCAGGGTGGACAGGTGGGCA

GCGAGCAGGAGAAGAAGGGTATGATTGAGAAGATCAAGGAGAAGCTGCCAGGTCACAAGT

AGGATGTGTGTCCACCATACACGTGGAGTCTACACCTACGTGTCTTATGTTACTTATAAT

ACGCATGGAGTGTGTCCGGAGTCTGTAATAATCTGCGCGTATCTGTTATGTC

>YNMG41

AGCCGTTGGATCTAGTTGAAAATGTCAGAGACGCGTGATGAGTATGGCAACCAGGTTCGC

CAAACCGACGAGTATGGGAACCCGATTCAGCATACTGGCACCGGGACAAAGCCCGGTTCG

GGCATACATGGTGGGGGCCATGGGATTGGCACAGGTGGTGGTGGTGGACAAGGCAAGCTC

CACCGCCCAGGCTCCGGCTCTTCCTCTGTAAGTATATTCGATGTTTCTCAAGGGCCATTT

CACTTGGTATATCGTGTTGGGATGACTAGATTAGTTGATTAACGGTTGTGATTGTAAATC

AGTGATATGGGCTTCTGTGTACCTTATTTCACAATGATAATCAATTGATACGCTGGATCG

TGATGAGCGGACCGGATGAGCCGTCCATCTTACAGGCTTTTTAAAATAGAATTATAGGGT

GGGTGGTATGAGGTCTACCAAGCCTGTAACATGGAATTACAATCATAGCCGTTAATCGGT

TCATCTCATCCGCTCATCGTGATCCAGCATGTCAAACAGAGATGGGTTTATGGAGAAATG

GATGTATGTAAGCCAACAAATCACAAAAAAAATCTGACATAATGATGGAGAGAATGCAAG

GAGTGTAAGTGAATCTACGCCGTTAAATGGATTTCATTGTAATTAATTTCGTTTTGTCAA

ATATTTTGTAGGACGAGGATGATGGACAAGGTGGGCGTAGGAAGAAGGGTTTGACGGAGA

AGATCAAAGAGAAGCTGCCAGGTGGAAACAAGACGACAGGTGCCTGTCATCCAGGGACTC

AGGGAGTACAGGGGGGTCGCGAGCATGAGAAGCCAGGTTGCGGTCAGGGTGGACAGGTGG

GCCGCGAGCATGAGAAGACAGGTTTCGGTCATCCAGGGACTCAGGGTGGACAGGTGGGCA

GCGAGCAGGAGAAGAAGGGTATGATTGAGAAGATCAAGGAGAAGCTGCCAGGTCACAAGT

AGGATGTGTGTCCACCATACACGTGGAGTCTACACCTACGTGTCTTATGTTACTTATAAT

ACGCATGGAGTGTGTCCGGAGTCTGTAATAATCTGCGCGTATCTGTTATGTC

>YNMG43

AGCCGTTGGATCTAGTTGAAAATGTCAGAGACGCGTGATGAGTATGGCAACCAGGTTCGC

CAAACCGACGAGTATGGGAACCCGATTCAGCATACTGGCACCGGGACAAAGCCCGGTTCG

GGCATACATGGTGGGGGCCATGGGATTGGCACAGGTGGTGGTGGTGGACAAGGCAAGCTC

CACCGCCCAGGCTCCGGCTCTTCCTCTGTAAGTATATTCGATGTTTCTCAAGGGCCATTT

CACTTGGTATATCGTGTTGGGATGACTAGATTAGTTGATTAACGGTTGTGATTGTAAATC

AGTGATATGGGCTTCTGTGTACCTTATTTCACAATGATAATCAATTGATACGCTGGATCG

TGATGAGCGGACCGGATGAGCCGTCCATCTTACAGGCTTTTTAAAATAGAATTATAGGGT

GGGTGGTATGAGGTCTACCAAGCCTGTAACATGGAATTACAATCATAGCCGTTAATCGGT

TCATCTCATCCGCTCATCGTGATCCAGCATGTCAAACAGAGATGGGTTTATGGAGAAATG

GATGTATGTAAGCCAACAAATCACAAAAAAAATCTGACATAATGATGGAGAGAATGCAAG

GAGTGTAAGTGAATCTACGCCGTTAAATGGATTTCATTGTAATTAATTTCGTTTTGTCAA

ATATTTTGTAGGACGAGGATGATGGACAAGGTGGGCGTAGGAAGAAGGGTTTGACGGAGA

AGATCAAAGAGAAGCTGCCAGGTGGAAACAAGACGACAGGTGCCTGTCATCCAGGGACTC

AGGGAGTACAGGGGGGTCGCGAGCATGAGAAGCCAGGTTGCGGTCAGGGTGGACAGGTGG

GCCGCGAGCATGAGAAGACAGGTTTCGGTCATCCAGGGACTCAGGGTGGACAGGTGGGCA

GCGAGCAGGAGAAGAAGGGTATGATTGAGAAGATCAAGGAGAAGCTGCCAGGTCACAAGT

AGGATGTGTGTCCACCATACACGTGGAGTCTACACCTACGTGTCTTATGTTACTTATAAT

ACGCATGGAGTGTGTCCGGAGTCTGTAATAATCTGCGCGTATCTGTTATGTC

>YNMG38

AGCCGTTGGATCTAGTTGAAAATGTCAGAGACGCGTGATGAGTATGGCAACCAGGTTCGC

CAAACCGACGAGTATGGGAACCCGATTCAGCATACTGGCACCGGGACAAAGCCCGGTTCG

GGCATACATGGTGGGGGCCATGGGATTGGCACAGGTGGTGGTGGTGGACAAGGCAAGCTC

CACCGCCCAGGCTCCGGCTCTTCCTCTGTAAGTATATTCGATGTTTCTCAAGGGCCATTT

CACTTGGTATATCGTGTTGGGATGACTAGATTAGTTGATTAACGGTTGTGATTGTAAATC

AGTGATATGGGCTTCTGTGTACCTTATTTCACAATGATAATCAATTGATACGCTGGATCG

TGATGAGCGGACCGGATGAGCCGTCCATCTTACAGGCTTTTTAAAATAGAATTATAGGGT

GGGTGGTATGAGGTCTACCAAGCCTGTAACATGGAATTACAATCATAGCCGTTAATCGGT

TCATCTCATCCGCTCATCGTGATCCAGCATGTCAAACAGAGATGGGTTTATGGAGAAATG

GATGTATGTAAGCCAACAAATCACAAAAAAAATCTGACATAATGATGGAGAGAATGCAAG

GAGTGTAAGTGAATCTACGCCGTTAAATGGATTTCATTGTAATTAATTTCGTTTTGTCAA

ATATTTTGTAGGACGAGGATGATGGACAAGGTGGGCGTAGGAAGAAGGGTTTGACGGAGA

AGATCAAAGAGAAGCTGCCAGGTGGAAACAAGACGACAGGTGCCTGTCATCCAGGGACTC

AGGGAGTACAGGGGGGTCGCGAGCATGAGAAGCCAGGTTGCGGTCAGGGTGGACAGGTGG

GCCGCGAGCATGAGAAGACAGGTTTCGGTCATCCAGGGACTCAGGGTGGACAGGTGGGCA

GCGAGCAGGAGAAGAAGGGTATGATTGAGAAGATCAAGGAGAAGCTGCCAGGTCACAAGT

AGGATGTGTGTCCACCATACACGTGGAGTCTACACCTACGTGTCTTATGTTACTTATAAT

ACGCATGGAGTGTGTCCGGAGTCTGTAATAATCTGCGCGTATCTGTTATGTC

>YNMG39

AGCCGTTGGATCTAGTTGAAAATGTCAGAGACGCGTGATGAGTATGGCAACCAGGTTCGC

CAAACCGACGAGTATGGGAACCCGATTCAGCATACTGGCACCGGGACAAAGCCCGGTTCG

GGCATACATGGTGGGGGCCATGGGATTGGCACAGGTGGTGGTGGTGGACAAGGCAAGCTC

CACCGCCCAGGCTCCGGCTCTTCCTCTGTAAGTATATTCGATGTTTCTCAAGGGCCATTT

CACTTGGTATATCGTGTTGGGATGACTAGATTAGTTGATTAACGGTTGTGATTGTAAATC

AGTGATATGGGCTTCTGTGTACCTTATTTCACAATGATAATCAATTGATACGCTGGATCG

TGATGAGCGGACCGGATGAGCCGTCCATCTTACAGGCTTTTTAAAATAGAATTATAGGGT

GGGTGGTATGAGGTCTACCAAGCCTGTAACATGGAATTACAATCATAGCCGTTAATCGGT

TCATCTCATCCGCTCATCGTGATCCAGCATGTCAAACAGAGATGGGTTTATGGAGAAATG

GATGTATGTAAGCCAACAAATCACAAAAAAAATCTGACATAATGATGGAGAGAATGCAAG

GAGTGTAAGTGAATCTACGCCGTTAAATGGATTTCATTGTAATTAATTTCGTTTTGTCAA

ATATTTTGTAGGACGAGGATGATGGACAAGGTGGGCGTAGGAAGAAGGGTTTGACGGAGA

AGATCAAAGAGAAGCTGCCAGGTGGAAACAAGACGACAGGTGCCTGTCATCCAGGGACTC

AGGGAGTACAGGGGGGTCGCGAGCATGAGAAGCCAGGTTGCGGTCAGGGTGGACAGGTGG

GCCGCGAGCATGAGAAGACAGGTTTCGGTCATCCAGGGACTCAGGGTGGACAGGTGGGCA

GCGAGCAGGAGAAGAAGGGTATGATTGAGAAGATCAAGGAGAAGCTGCCAGGTCACAAGT

AGGATGTGTGTCCACCATACACGTGGAGTCTACACCTACGTGTCTTATGTTACTTATAAT

ACGCATGGAGTGTGTCCGGAGTCTGTAATAATCTGCGCGTATCTGTTATGTC

>YNMG310

AGCCGTTGGATCTAGTTGAAAATGTCAGAGACGCGTGATGAGTATGGCAACCAGGTTCGC

CAAACCGACGAGTATGGGAACCCGATTCAGCATACTGGCACCGGGACAAAGCCCGGTTCG

GGCATACATGGTGGGGGCCATGGGATTGGCACAGGTGGTGGTGGTGGACAAGGCAAGCTC

CACCGCCCAGGCTCCGGCTCTTCCTCTGTAAGTATATTCGATGTTTCTCAAGGGCCATTT

CACTTGGTATATCGTGTTGGGATGACTAGATTAGTTGATTAACGGTTGTGATTGTAAATC

AGTGATATGGGCTTCTGTGTACCTTATTTCACAATGATAATCAATTGATACGCTGGATCG

TGATGAGCGGACCGGATGAGCCGTCCATCTTACAGGCTTTTTAAAATAGAATTATAGGGT

GGGTGGTATGAGGTCTACCAAGCCTGTAACATGGAATTACAATCATAGCCGTTAATCGGT

TCATCTCATCCGCTCATCGTGATCCAGCATGTCAAACAGAGATGGGTTTATGGAGAAATG

GATGTATGTAAGCCAACAAATCACAAAAAAAATCTGACATAATGATGGAGAGAATGCAAG

GAGTGTAAGTGAATCTACGCCGTTAAATGGATTTCATTGTAATTAATTTCGTTTTGTCAA

ATATTTTGTAGGACGAGGATGATGGACAAGGTGGGCGTAGGAAGAAGGGTTTGACGGAGA

AGATCAAAGAGAAGCTGCCAGGTGGAAACAAGACGACAGGTGCCTGTCATCCAGGGACTC

AGGGAGTACAGGGGGGTCGCGAGCATGAGAAGCCAGGTTGCGGTCAGGGTGGACAGGTGG

GCCGCGAGCATGAGAAGACAGGTTTCGGTCATCCAGGGACTCAGGGTGGACAGGTGGGCA

GCGAGCAGGAGAAGAAGGGTATGATTGAGAAGATCAAGGAGAAGCTGCCAGGTCACAAGT

AGGATGTGTGTCCACCATACACGTGGAGTCTACACCTACGTGTCTTATGTTACTTATAAT

ACGCATGGAGTGTGTCCGGAGTCTGTAATAATCTGCGCGTATCTGTTATGTC

>YNJP827

AGCCGTTGGATCTAGTTGAAAATGTCAGAGACGCGTGATGAGTATGGCAACCAGGTTCGC

CAAACCGACGAGTATGGGAACCCGATTCAGCATACTGGCACCGGGACAAAGCCCGGTTCG

GGCATACATGGTGGGGGCCATGGGATTGGCACAGGTGGTGGTGGTGGACAAGGCAAGCTC

CACCGCCCAGGCTCCGGCTCTTCCTCTGTAAGTATATTCGATGTTTCTCAAGGGCCATTT

CACTTGGTATATCGTGTTGGGATGACTAGATTAGTTGATTAACGGTTGTGATTGTAAATC

AGTGATATGGGCTTCTGTGTACCTTATTTCACAATGATAATCAATTGATACGCTGGATCG

TGATGAGCGGACCGGATGAGCCGTCCATCTTACAGGCTTTTTAAAATAGAATTATAGGGT

GGGTGGTATGAGGTCTACCAAGCCTGTAACATGGAATTACAATCATAGCCGTTAATCGGT

TCATCTCATCCGCTCATCGTGATCCAGCATGTCAAACAGAGATGGGTTTATGGAGAAATG

GATGTATGTAAGCCAACAAATCACAAAAAAAATCTGACATAATGATGGAGAGAATGCAAG

GAGTGTAAGTGAATCTACGCCGTTAAATGGATTTCATTGTAATTAATTTCGTTTTGTCAA

ATATTTTGTAGGACGAGGATGATGGACAAGGTGGGCGTAGGAAGAAGGGTTTGACGGAGA

AGATCAAAGAGAAGCTGCCAGGTGGAAACAAGACGACAGGTGCCTGTCATCCAGGGACTC

AGGGTGTACAGGGGGGTCGCGAGCATGAGAAGCCAGGTTGCGGTCAGGGTGGACAGGTGG

GCCGCGAGCATGAGAAGACAGGTTTCGGTCATCCAGGGACTCAGGGTGGACAGGTGGGCA

GCGAGCAGGAGAAGAAGGGTATGATTGAGAAGATCAAGGAGAAGCTGCCAGGTCACAAGT

AGGATGTGTGTCCACCATACACGTGGAGTCTACACCTACGTGTCTTATGTTACTTATAAT

ACGCATGGAGTGTGTCCGGAGTCTGTAATAATCTGCGCGTATCTGTTATGTC

>YNJP828

AGCCGTTGGATCTAGTTGAAAATGTCAGAGACGCGTGATGAGTATGGCAACCAGGTTCGC

CAAACCGACGAGTATGGGAACCCGATTCAGCATACTGGCACCGGGACAAAGCCCGGTTCG

GGCATACATGGTGGGGGCAATGGGATTGGCACAGGTGGTGGTGGTGGACAAGGCAAGCTC

CACCGCCCAGGCTCCGGCTCTTCCTCTGTAAGTATATTCGATGTTTCTCAAGGGCCATTT

CACTTGGTATATCGTGTTGGGATGACTAGATTAGTTGATTAACGGTTGTGATTGTAAATC

AGTGATATGGGCTTCTGTGTACCTTATTTCACAATGATAATCAATTGATACGCTGGATCG

TGATGAGCGGACCGGATGAGCCGTCCATCTTACAGGCTTTTTAAAATAGAATTATAGGGT

GGGTGGTATGAGGTCTACCAAGCCTGTAACATGGAATTACAATCATAGCCGTTAATCGGT

TCATCTCATCCGCTCATCGTGATCCAGCATGTCAAACAGAGATGGGTTTATGGAGAAATG

GATGTATGTAAGCCAACAAATCACAAAAAAAATCTGACATAATGATGGAGAGAATGCAAG

GAGTGTAAGTGAATCTACGCCGTTAAATGGATTTCATTGTAATTAATTTCGTTTTGTCAA

ATATTTTGTAGGACGAGGATGATGGACAAGGTGGGCGTAGGAAGAAGGGTTTGACGGAGA

AGATCAAAGAGAAGCTGCCAGGTGGAAACAAGACGACAGGTGCCTGTCATCCAGGGACTC

AGGGTGTACAGGGGGGTCGCGAGCATGAGAAGCCAGGTTGCGGTCAGGGTGGACAGGTGG

GCCGCGAGCATGAGAAGACAGGTTTCGGTCATCCAGGGACTCAGGGTGGACAGGTGGGCA

GCGAGCAGGAGAAGAAGGGTATGATTGAGAAGATCAAGGAGAAGCTGCCAGGTCACAAGT

AGGATGTGTGTCCACCATACACGTGGAGTCTACACCTACGTGTCTTATGTTACTTATAAT

ACGCATGGAGTGTGTCCGGAGTCTGTAATAATCTGCGCGTATCTGTTATGTC

>YNJP1014

AGCCGTTGGATCTAGTTGAAAATGTCAGAGACGCGTGATGAGTATGGCAACCAGGTTCGC

CAAACCGACGAGTATGGGAACCCGATTCAGCATACTGGCACCGGGACAAAGCCCGGTTCG

GGCATACATGGTGGGGGCCATGGGATTGGCACAGGTGGTGGTGGTGGACAAGGCAAGCTC

CACCGCCCAGGCTCCGGCTCTTCCTCTGTAAGTATATTCGATGTTTCTCAAGGGCCATTT

CACTTGGTATATCGTGTTGGGATGACTAGATTAGTTGATTAACGGTTGTGATTGTAAATC

AGTGATATGGGCTTCTGTGTACCTTATTTCACAATGATAATCAATTGATACGCTGGATCG

TGATGAGCGGACCGGATGAGCCGTCCATCTTACAGGCTTTTTAAAATAGAATTATAGGGT

GGGTGGTATGAGGTCTACCAAGCCTGTAACATGGAATTACAATCATAGCCGTTAATCGGT

TCATCTCATCCGCTCATCGTGATCCAGCATGTCAAACAGAGATGGGTTTATGGAGAAATG

GATGTATGTAAGCCAACAAATCACAAAAAAAATCTGACATAATGATGGAGAGAATGCAAG

GAGTGTAAGTGAATCTACGCCGTTAAATGGATTTCATTGTAATTAATTTCGTTTTGTCAA

ATATTTTGTAGGACGAGGATGATGGACAAGGTGGGCGTAGGAAGAAGGGTTTGACGGAGA

AGATCAAAGAGAAGCTGCCAGGTGGAAACAAGACGACAGGTGCCTGTCATCCAGGGACTC

AGGGTGTACAGGGGGGTCGCGAGCATGAGAAGCCAGGTTGCGGTCAGGGTGGACAGGTGG

GCCGCGAGCATGAGAAGACAGGTTTCGGTCATCCAGGGACTCAGGGTGGACAGGTGGGCA

GCGAGCAGGAGAAGAAGGGTATGATTGAGAAGATCAAGGAGAAGCTGCCAGGTCACAAGT

AGGATGTGTGTCCACCATACACGTGGAGTCTACACCTACGTGTCTTATGTTACTTATAAT

ACGCATGGAGTGTGTCCGGAGTCTGTAATAATCTGCGCGTATCTGTTATGTC

>YNJP101

AGCCGTTGGATCTAGTTGAAAATGTCAGAGACGCGTGATGAGTATGGCAACCAGGTTCGC

CAAACCGACGAGTATGGGAACCCGATTCAGCATACTGGCACCGGGACAAAGCCCGGTTCG

GGCATACATGGTGGGGGCCATGGGATTGGCACAGGTGGTGGTGGTGGACAAGGCAAGCTC

CACCGCCCAGGCTCCGGCTCTTCCTCTGTAAGTATATTCGATGTTTCTCAAGGGCCATTT

CACTTGGTATATCGTGTTGGGATGACTAGATTAGTTGATTAACGGTTGTGATTGTAAATC

AGTGATATGGGCTTCTGTGTACCTTATTTCACAATGATAATCAATTGATACGCTGGATCG

TGATGAGCGGACCGGATGAGCCGTCCATCTTACAGGCTTTTTAAAATAGAATTATAGGGT

GGGTGGTATGAGGTCTACCAAGCCTGTAACATGGAATTACAATCATAGCCGTTAATCGGT

TCATCTCATCCGCTCATCGTGATCCAGCATGTCAAACAGAGATGGGTTTATGGAGAAATG

GATGTATGTAAGCCAACAAATCACAAAAAAAATCTGACATAATGATGGAGAGAATGCAAG

GAGTGTAAGTGAATCTACGCCGTTAAATGGATTTCATTGTAATTAATTTCGTTTTGTCAA

ATATTTTGTAGGACGAGGATGATGGACAAGGTGGGCGTAGGAAGAAGGGTTTGACGGAGA

AGATCAAAGAGAAGCTGCCAGGTGGAAACAAGACGACAGGTGCCTGTCATCCAGGGACTC

AGGGTGTACAGGGGGGTCGCGAGCATGAGAAGCCAGGTTGCGGTCAGGGTGGACAGGTGG

GCCGCGAGCATGAGAAGACAGGTTTCGGTCATCCAGGGACTCAGGGTGGACAGGTGGGCA

GCGAGCAGGAGAAGAAGGGTATGATTGAGAAGATCAAGGAGAAGCTGCCAGGTCACAAGT

AGGATGTGTGTCCACCATACACGTGGAGTCTACACCTACGTGTCTTATGTTACTTATAAT

ACGCATGGAGTGTGTCCGGAGTCTGTAATAATCTGCGCGTATCTGTTATGTC

>YNJP1017

AGCCGTTGGATCTAGTTGAAAATGTCAGAGACGCGTGATGAGTATGGCAACCAGGTTCGC

CAAACCGACGAGTATGGGAACCCGATTCAGCATACTGGCACCGGGACAAAGCCCGGTTCG

GGCATACATGGTGGGGGCCATGGGATTGGCACAGGTGGTGGTGGTGGACAAGGCAAGCTC

CACCGCCCAGGCTCCGGCTCTTCCTCTGTAAGTATATTCGATGTTTCTCAAGGGCCATTT

CACTTGGTATATCGTGTTGGGATGACTAGATTAGTTGATTAACGGTTGTGATTGTAAATC

AGTGATATGGGCTTCTGTGTACCTTATTTCACAATGATAATCAATTGATACGCTGGATCG

TGATGAGCGGACCGGATGAGCCGTCCATCTTACAGGCTTTTTAAAATAGAATTATAGGGT

GGGTGGTATGAGGTCTACCAAGCCTGTAACATGGAATTACAATCATAGCCGTTAATCGGT

TCATCTCATCCGCTCATCGTGATCCAGCATGTCAAACAGAGATGGGTTTATGGAGAAATG

GATGTATGTAAGCCAACAAATCACAAAAAAAATCTGACATAATGATGGAGAGAATGCAAG

GAGTGTAAGTGAATCTACGCCGTTAAATGGATTTCATTGTAATTAATTTCGTTTTGTCAA

ATATTTTGTAGGACGAGGATGATGGACAAGGTGGGCGTAGGAAGAAGGGTTTGACGGAGA

AGATCAAAGAGAAGCTGCCAGGTGGAAACAAGACGACAGGTGCCTGTCATCCAGGGACTC

AGGGTGTACAGGGGGGTCGCGAGCATGAGAAGCCAGGTTGCGGTCAGGGTGGACAGGTGG

GCCGCGAGCATGAGAAGACAGGTTTCGGTCATCCAGGGACTCAGGGTGGACAGGTGGGCA

GCGAGCAGGAGAAGAAGGGTATGATTGAGAAGATCAAGGAGAAGCTGCCAGGTCACAAGT

AGGATGTGTGTCCACCATACACGTGGAGTCTACACCTACGTGTCTTATGTTACTTATAAT

ACGCATGGAGTGTGTCCGGAGTCTGTAATAATCTGCGCGTATCTGTTATGTC

>YNJP54

AGCCGTTGGATCTAGTTGAAAATGTCAGAGACGCGTGATGAGTATGGCAACCAGGTTCGC

CAAACCGACGAGTATGGGAACCCGATTCAGCATACTGGCACCGGGACAAAGCCCGGTTCG

GGCATACATGGTGGGGGCCATGGGATTGGCACAGGTGGTGGTGGTGGACAAGGCAAGCTC

CACCGCCCAGGCTCCGGCTCTTCCTCTGTAAGTATATTCGATGTTTCTCAAGGGCCATTT

CACTTGGTATATCGTGTTGGGATGACTAGATTAGTTGATTAACGGTTGTGATTGTAAATC

AGTGATATGGGCTTCTGTGTACCTTATTTCACAATGATAATCAATTGATACGCTGGATCG

TGATGAGCGGACCGGATGAGCCGTCCATCTTACAGGCTTTTTAAAATAGAATTATAGGGT

GGGTGGTATGAGGTCTACCAAGCCTGTAACATGGAATTACAATCATAGCCGTTAATCGGT

TCATCTCATCCGCTCATCGTGATCCAGCATGTCAAACAGAGATGGGTTTATGGAGAAATG

GATGTATGTAAGCCAACAAATCACAAAAAAAATCTGACATAATGATGGAGAGAATGCAAG

GAGTGTAAGTGAATCTACGCCGTTAAATGGATTTCATTGTAATTAATTTCGTTTTGTCAA

ATATTTTGTAGGACGAGGATGATGGACAAGGTGGGCGTAGGAAGAAGGGTTTGACGGAGA

AGATCAAAGAGAAGCTGCCAGGTGGAAACAAGACGACAGGTGCCTGTCATCCAGGGACTC

AGGGTGTACAGGGGGGTCGCGAGCATGAGAAGCCAGGTTGCGGTCAGGGTGGACAGGTGG

GCCGCGAGCATGAGAAGACAGGTTTCGGTCATCCAGGGACTCAGGGTGGACAGGTGGGCA

GCGAGCAGGAGAAGAAGGGTATGATTGAGAAGATCAAGGAGAAGCTGCCAGGTCACAAGT

AGGATGTGTGTCCACCATACACGTGGAGTCTACACCTACGTGTCTTATGTTACTTATAAT

ACGCATGGAGTGTGTCCGGAGTCTGTAATAATCTGCGCGTATCTGTTATGTC

>YNJP55

AGCCGTTGGATCTAGTTGAAAATGTCAGAGACGCGTGATGAGTATGGCAACCAGGTTCGC

CAAACCGACGAGTATGGGAACCCGATTCAGCATACTGGCACCGGGACAAAGCCCGGTTCG

GGCATACATGGTGGGGGCCATGGGATTGGCACAGGTGGTGGTGGTGGACAAGGCAAGCTC

CACCGCCCAGGCTCCGGCTCTTCCTCTGTAAGTATATTCGATGTTTCTCAAGGGCCATTT

CACTTGGTATATCGTGTTGGGATGACTAGATTAGTTGATTAACGGTTGTGATTGTAAATC

AGTGATATGGGCTTCTGTGTACCTTATTTCACAATGATAATCAATTGATACGCTGGATCG

TGATGAGCGGACCGGATGAGCCGTCCATCTTACAGGCTTTTTAAAATAGAATTATAGGGT

GGGTGGTATGAGGTCTACCAAGCCTGTAACATGGAATTACAATCATAGCCGTTAATCGGT

TCATCTCATCCGCTCATCGTGATCCAGCATGTCAAACAGAGATGGGTTTATGGAGAAATG

GATGTATGTAAGCCAACAAATCACAAAAAAAATCTGACATAATGATGGAGAGAATGCAAG

GAGTGTAAGTGAATCTACGCCGTTAAATGGATTTCATTGTAATTAATTTCGTTTTGTCAA

ATATTTTGTAGGACGAGGATGATGGACAAGGTGGGCGTAGGAAGAAGGGTTTGACGGAGA

AGATCAAAGAGAAGCTGCCAGGTGGAAACAAGACGACAGGTGCCTGTCATCCAGGGACTC

AGGGTGTACAGGGGGGTCGCGAGCATGAGAAGCCAGGTTGCGGTCAGGGTGGACAGGTGG

GCCGCGAGCATGAGAAGACAGGTTTCGGTCATCCAGGGACTCAGGGTGGACAGGTGGGCA

GCGAGCAGGAGAAGAAGGGTATGATTGAGAAGATCAAGGAGAAGCTGCCAGGTCACAAGT

AGGATGTGTGTCCACCATACACGTGGAGTCTACACCTACGTGTCTTATGTTACTTATAAT

ACGCATGGAGTGTGTCCGGAGTCTGTAATAATCTGCGCGTATCTGTTATGTC

>YNJP5915

AGCCGTTGGATCTAGTTGAAAATGTCAGAGACGCGTGATGAGTATGGCAACCAGGTTCGC

CAAACCGACGAGTATGGGAACCCGATTCAGCATACTGGCACCGGGACAAAGCCCGGTTCG

GGCATACATGGTGGGGGCCATGGGATTGGCACAGGTGGTGGTGGTGGACAAGGCAAGCTC

CACCGCCCAGGCTCCGGCTCTTCCTCTGTAAGTATATTCGATGTTTCTCAAGGGCCATTT

CACTTGGTATATCGTGTTGGGATGACTAGATTAGTTGATTAACGGTTGTGATTGTAAATC

AGTGATATGGGCTTCTGTGTACCTTATTTCACAATGATAATCAATTGATACGCTGGATCG

TGATGAGCGGACCGGATGAGCCGTCCATCTTACAGGCTTTTTAAAATAGAATTATAGGGT

GGGTGGTATGAGGTCTACCAAGCCTGTAACATGGAATTACAATCATAGCCGTTAATCGGT

TCATCTCATCCGCTCATCGTGATCCAGCATGTCAAACAGAGATGGGTTTATGGAGAAATG

GATGTATGTAAGCCAACAAATCACAAAAAAAATCTGACATAATGATGGAGAGAATGCAAG

GAGTGTAAGTGAATCTACGCCGTTAAATGGATTTCATTGTAATTAATTTCGTTTTGTCAA

ATATTTTGTAGGACGAGGATGATGGACAAGGTGGGCGTAGGAAGAAGGGTTTGACGGAGA

AGATCAAAGAGAAGCTGCCAGGTGGAAACAAGACGACAGGTGCCTGTCATCCAGGGACTC

AGGGTGTACAGGGGGGTCGCGAGCATGAGAAGCCAGGTTGCGGTCAGGGTGGACAGGTGG

GCCGCGAGCATGAGAAGACAGGTTTCGGTCATCCAGGGACTCAGGGTGGACAGGTGGGCA

GCGAGCAGGAGAAGAAGGGTATGATTGAGAAGATCAAGGAGAAGCTGCCAGGTCACAAGT

AGGATGTGTGTCCACCATACACGTGGAGTCTACACCTACGTGTCTTATGTTACTTATAAT

ACGCATGGAGTGTGTCCGGAGTCTGTAATAATCTGCGCGTATCTGTTATGTC

>Hershey152

AGCCGTTGGATCTAGTTGAAAATGTCAGTGACGCGTGATGAGTATGGCAACCAGGTTCGC

CAGACCGACGAGTACGGGAACCCGATTCAGCATACTGGCACCGGGACAAAGGCCGGTTCG

GGCATACATGGTGGGGGCCATGGGATTGGCACAGGTGGTGGTGGTGGTGGACAAGGCAAG

CTCCACCGTTCAGGCTCTGGCTCCTCCTCTGTAAGTACATTCGATGTTTCTCAAGGGTCA

TTTCACTTGGTATATCGTGTTGGGATGATTAGATTAGTTGATTAACGGTTGTGATTGTAA

ATCAGTGATATTGGCTTCTGTGTACCTTAGTTCACGATGATAATCAATTGATACGCTGGA

TCGTGATGAGCGGACCGGATGAGCCGTCCATCTTACAGGCTTTTTAAAATAGAATTATAG

GGTGGGTGGTATGAGGTCCACCAAAGCCTGTAGCATGGAATTACAATCATAACCGTTAAT

CGGTTCATCTCATCCGCTCATCGTGATCTAGCATGTCAAACGGAGACGGGTTTCTGGAGA

AATGGATGTATGTAAGCCCACAAATCACAAAAATCTGACATTATGATGGAGAGAATGCAA

GGAGTGTAAGCGAATTTACGCCGTTAGATGGATTTCATTGTACTTTAACTTCGTTTTGTC

AAATATTTTGTAGGATGAGGATGATGGACAAGGTGGGCGTAGGAAGAAGGGTTTGACGGA

GAAGATCAAAGAGAAGCTGCCAGGTGGAAACAAGACGACAGGTGTCTGTCATCCAGGGAC

TCAGGGTGTACAGGGGGGCCGCGAGCATGAGAAGACAGGTTGCGGTCAGGGTGGTCAGGG

TGGACAGGTGGGCCGCGAGCATGAGAAGACAGGTTTCGGCCATCCAGTGACTCAGGGTGG

ACAGGTGGGCAACGAGCAGGAGAAGAAGGGTATGATTGAGAAGATCCAGGAGAAGCTGCC

AGGTCACAAGTAGGATGTGTGCCCACCATACACGTGGAGTCTACATCTACGTGCCTATTA

TGTTACTATAATACGCATGGAGTGTGTCCGGAGTCTGTAATAATCTGCGCGTATCTGTTA

TGTC

>Hershey155

AGCCGTTGGATCTAGTTGAAAATGTCACAGACGCGTGATGAGTATGGCAGCCAGGTTCGC

CAAACCGACGAGTATGGGAACCCGATTCAGCATACCGGCCCTGGGACAAAGGCCGGTTCA

GGCATACATGGTGGGGGCCATGGGATTGGCACAGGCGGTGGTGGTGGTGGACAAGGTAAG

CTCCACCGTTCAGGCTCCGGCTCTTCCTCTTCCTCTGTAAGTACATTCGATGTTTCTCAA

GGGTCATTTCACTTGGTATATCGTGTTGGGACGATTAGATTAGTTGATTAACGGTTGTGA

TTGTAAATCAGTGACATGGGCTTCTGTGTACCTTATTTCACAATGATAATCAATTGATAC

GCTGGATCGTGATGAGCGGACCGGATGAGCCGTCCATCTTACAGGCTTTTTAAAATAGAA

TTATAGGGTGGGTGGTATGAGGTCCACCAAAGCCTGTAACATGGAATTACAATCATAGCC

GCTAATTGGTTCATCTCATCCGCTCATCGTGATCTAGCATGTCAAACAGAGATGGGTTTC

TGGAGAAATGGATGTACGTAAGCCCACAAATCGCAAAAATCTGACATTTTGCTGGAGAGA

ATGCAAGGAGTGTAAGCGAATCTACGCCGTTAGATGGATTTCATTGTACTTTAACTTCGT

TTCCTCAAATATTTTGTAGGATGAGGATGATGGACAAGGTGGGCGTAGGAACAGGGGTTT

GACGGAGAAGATCGAAGAGAAGCTGCCAGGTGGAAACAAGACGACAGGTGTCTGTCATCC

AGGGACTCAGGGTGTACAGGGGGGCCGCGAGCATGAGAAGACAGGTTGCGGTCAGGGTGG

ACAGGTGGGCCGCGAGCATGAGAAGACAGGTTTCGGCCATCCAGGGACTCAGGGTGGACA

AGTGGGCAACGAGCAGGAGAAGAAGGGTATGATTGAGAAGATCCAGGAGAAGCTGCCAGG

TCACAAGTAGGATGTGTGCCCACCATACACGTGGAGTCTACATCTACGTGCCTATTATGT

TACTATAATACGCATGGAGTGTGTCCGGAGTCTGTAATAATCTGCGCGTATCTGTTATGT

C

>Hershey158

AGCCGTTGGATCTAGTTGAAAATGTCTGAGACGCGTGATGAGTATGGCAACCAGGTTCGC

CAAACCGACGAGTATGGGAACCCGATTCAGCATACTGGCACCGGCACAAAGGCCGGTGCG

GGCATACATGCTGGGGGCCATGGGATTGGCACAGGTGGTGGTGGTGGACAAGGCAAGCTC

CACCGTTCAGGCTCCGGGTCTTCCTCTGTAAGTATATTCGATGTTTCTCAAGGGTCATTT

CACTTGGTAGATCGTGTTGTGATGACTAGATTAGTTGATTAACCGTTGTGATTGTAAATC

AGTGATATGGGCTTCTGTGTTCCTTATTTCACAATGATAATCAATTGATACGGTGGATCG

TGATGAGCGGACCGAATGAGCCGTCCATCTTACAGGCTTTTTAAAATAGAATTATAGGGT

GGGTGGTATGAGGTCCACCAAAGCCTGTAACATGGAATTACAATTATAGCCGTTAATCGG

TTCATCTCATCCGCTCATCGTGATCCAGCATGTCAAACAGAGATGGGTTTCTGGCGAAAT

GGATGTATGTAAGCCCACAAATCACAAAAATCTGACATTATGATGGAGAGAATTGCAAGG

AGTGTAAGTGAATCTACGCCGTTAGATGGATACTTTTTAACTTCGTTTTGTCAAATATTC

TGTAGGATGAGGATGATGGACAAGGTGGGCGTAGGAAGAAGGGTTTGACGGAGAAGATCA

AAGAGAAGCTGCCAGGTGGGCAGAAGACGACAGGTGTCGGTTATCCAGGGAATCAGGGTG

TACAGGGGGGCCGCGGGCATGAGAAGACAGGTGTCTGTCATCCAGGGACTCAGGGTGTAC

AGGGGGGCCGCGAGCATGAGAAGACAGGTTTCGGTCATCCAGGGACTCAGGGTGGACACG

AGCATGAGAAGACAGGTGTCGGTCATCCAGGGACTCAGGGTGAGCAGGAGAAGAAGGGTA

TGATTGAGAAGATCAAGGAGAAGCTGCCAGGTCACAAGTAGGATGTGTGCCCACCATACA

CGTGGAGTCTACATCTATGTTTCTATGTTACTATAATACGCATGGAGTGTGTACGGATTC

TGTAATAATCTGCGCGTATCTGTTATGTC

>Hershey283

AGCCGTTGGATCTAGTTGAAAATGTCTGAGACGCGTGATGAGTATGGCAACCAGGTTCGC

CAAACCGACGAGTATGGGAACCCGATTCAGCATACTGGCACCGGCACAAAGGCCGGTGCG

GGCATACATGCTGGGGGCCATGGGATTGGCACAGGTGGTGGTGGTGGACAAGGCAAGCTC

CACCGTTCAGGCTCCGGGTCTTCCTCTGTAAGTATATTCGATGTTTCTCAAGGGTCATTT

CACTTGGTAGATCGTGTTGTGATGACTAGATTAGTTGATTAACCGTTGTGATTGTAAATC

AGTGATATGGGCTTCTGTGTTCCTTATTTCACAATGATAATCAATTGATACGGTGGATCG

TGATGAGCGGACCGAATGAGCCGTCCATCTTACAGGCTTTTTAAAATAGAATTATAGGGT

GGGTGGTATGAGGTCCACCAAAGCCTGTAACATGGAATTACAATTATAGCCGTTAATCGG

TTCATCTCATCCGCTCATCGTGATCCAGCATGTCAAACAGAGATGGGTTTCTGGCGAAAT

GGATGTATGTAAGCCCACAAATCACAAAAATCTGACATTATGATGGAGAGAATTGCAAGG

AGTGTAAGTGAATCTACGCCGTTAGATGGATACTTTTTAACTTCGTTTTGTCAAATATTC

TGTAGGATGAGGATGATGGACAAGGTGGGCGTAGGAAGAAGGGTTTGACGGAGAAGATCA

AAGAGAAGCTGCCAGGTGGGCAGAAGACGACAGGTGTCGGTTATCCAGGGAATCAGGGTG

TACAGGGGGGCCGCGAGCATGAGAAGACAGGTGTCTGTCATCCAGGGACTCAGGGTGTAC

AGGGGGGCCGCGAGCATGAGAAGACAGGTTTCGGTCATCCAGGGACTCAGGGTGGACACG

AGCATGAGAAGACAGGTGTCGGTCATCCAGGGACTCAGGGTGAGCAGGAGAAGAAGGGTA

TGATTGAGAAGATCAAGGAGAAGCTGCCAGGTCACAAGTAGGATGTGTGCCCACCATACA

CGTGGAGTCTACATCTATGTTTCTATGTTACTATAATACGCATGGAGTGTGTACGGATTC

TGTAATAATCTGCGCGTATCTGTTATGTC

>Hershey286

AGCCGTTGGATCTAGTTGAAAATGTCTGAGACGCGTGATGAGTATGGCAACCAGGTTCGC

CAAACCGACGAGTATGGGAACCCGATTCAGCATACTGGCACCGGCACAAAGGCCGGTGCG

GGCATACATGCTGGGGGCCATGGGATTGGCACAGGTGGTGGTGGTGGACAAGGCAAGCTC

CACCGTTCAGGCTCCGGGTCTTCCTCTGTAAGTATATTCGATGTTTCTCAAGGGTCATTT

CACTTGGTAGATCGTGTTGTGATGACTAGATTAGTTGATTAACCGTTGTGATTGTAAATC

AGTGATATGGGCTTCTGTGTTCCTTATTTCACAATGATAATCAATTGATACGGTGGATCG

TGATGAGCGGACCGAATGAGCCGTCCATCTTACAGGCTTTTTAAAATAGAATTATAGGGT

GGGTGGTATGAGGTCCACCAAAGCCTGTAACATGGAATTACAATTATAGCCGTTAATCGG

TTCATCTCATCCGCTCATCGTGATCCAGCATGTCAAACAGAGATGGGTTTCTGGCGAAAT

GGATGTATGTAAGCCCACAAATCACAAAAATCTGACATTATGATGGAGAGAATTGCAAGG

AGTGTAAGTGAATCTACGCCGTTAGATGGATACTTTTTAACTTCGTTTTGTCAAATATTC

TGTAGGATGAGGATGATGGACAAGGTGGGCGTAGGAAGAAGGGTTTGACGGAGAAGATCA

AAGAGAAGCTGCCAGGTGGGCAGAAGACGACAGGTGTCGGTTATCCAGGGAATCAGGGTG

TACAGGGGGGCCGCGAGCATGAGAAGACAGGTGTCTGTCATCCAGGGACTCAGGGTGTAC

AGGGGGGCCGCGAGCATGAGAAGACAGGTTTCGGCCATGCAGTGACTCAGGGTGGACAGG

TGGGCAACGAGCAGGAGAAGAAGGGTATGATTGAGAAGATCCAGGAGAAGCTGCCAGGTC

ACAAGTAGGATGTGTGCCCACCATACACGTGGAGTCTACATCTACGTGCCTATTATGTTA

CTATAATACGCATGGAGTGTGTCCGGAGTCTGTAATAATCTGCGCGTATCTGTTATGTC

>Hershey2824

AGCCGTTGGATCTAGTTGAAAATGTCAGTGACGCGTGATGAGTATGGCAACCAGGTTCGC

CAGACCGACGAGTATGGGAACCCGATTCAGCATACTGGCACCGGGACAAAGGCCGGTTCG

GGCATACATGGTGGGGGCCATGGGATTGGCACAGGTGGTGGTGGTGGTGGACAAGGCAAG

CTCCACCGTTCAGGCTCTGGCTCTTCCTCTGTAAGTACATTCGATGTTTCTCAAGGGTCA

TTTCACTTGGTATATCGTGTTGGGATGATTAGATTAGTTGATTAACGGTTGTGATTGTAA

ATCAGTGATATGGGCATCTGTGTACCTTAGTTCACGATGATAATCAATTGATACGCTGGA

TCGTGATGAGCGGACCGGATGAGCCGTCCATCTTACAGGCTTTTTAAAATAGAATTATAG

GGTGGGTGGTATGAGGTCCACCAAAGCCTGTAACATGGAATTACAATCATAGCCGTTAAT

CGGTTCATCTCATCCGCTCATCGTGATCTAGCATGTCAAACAGAGATGGGTTTCTGGAGA

AATGGATGTATGTAAGCCCACAAATCACAAAAATCTGACATTATGATGGAGAGAATGCAA

GGAGTGTAAGCGAATTTACGCCGTTAGATGGATTTCATTGTACTTTTAACTTTGTTTTGT

CGAATATTTTGTAGGATGAGGATGATGGACAAGGTGGGCGTAGGAAGAAGGGTTTGACGG

AGAAGATCAAAGAGAAGCTGCCAGGTGGAAACAAGACGACAGGTGTCTGTCATCCAGGGA

CTCAGGGTGTACAGGGGGGCCGCGAGCATGAGAAGACAGGTTGCGGTCAGGGTGGTCAGG

GTGGACAGGTGGGCCGCGAGCATGAGAAGACAGGTTTCGGCCATGCAGTGACTCAGGGTG

GACAGGTGGGCAACGAGCAGGAGAAGAAGGGTATGATTGAGAAGATCCAGGAGAAGCTGC

CAGGTCACAAGTAGGATGTGTGCCCACCATACACGTGGAGTCTACATCTACGTGCCTATT

ATGTTACTATAATACGCATGGAGTGTGTCCGGAGTCTGTAATAATCTGCGCGTATCTGTT

ATGTC

>Hershey312

AGCCGTTGGATCTAGTTGAAAATGTCACAGGCGCGTGATGAGTATGGCAGCCAGGTTCGC

CAAACCGACGAGTATGGGAACCCGATTCAGCATACTGGCACGGGGACAACGGCCGGTACG

GGCATACATGGTGGGGGCCATGGGATTGGCACAGGTGGTGGTGGTGGTGGACAAGGCAAG

CTCCACCGTTCAGGCTCTGGCTCTTCCTCTGTAAGTACATTCGATGTTTCTCAAGGGTTA

TTTCACTTGGTATATCGTGTTGGGATGATTAGATTAGTTGATTAACGGTTGTGAATGTAA

ATCAGTGATATGGCTTCTGTGTACCTTATTTCACAATGATAATCAATTGATACGCTGGAT

CGTGATGAGCGGACCGGATGAGCCGTCCAGCTTACAGGCTTTTTAAAATAGAATTATAGG

TGGGTGGTATGAGGTCCACCAAAGCCTGTAACATGGAATTACAATCATAGCCGTTAATTG

GCTCATCTCATCCGCTCATCGTGATCTAGCATGTCAAACAAAGATGGGTTTCTGGAGAAA

TGGATGTATGTAAGCCCACAAATCGCAAAAATCTGACATTATGATGGAGAGAATGCAAGG

AGTGTAAGCGAATCTACGCCGTTAGATGGATTTCATTGCACTTTAACTTCGTTTCCTCAA

ATATTTTGTAGGATGAGGATGATGGACAAGGTGGGCGTAGGAACAGGGGTTTGACGGAGA

AGATCAAAGAGAAGCTGCCAGGTGGAAACAAGACGACAGGTGTCTGTCATCCAGGGACTC

AGGGTGTACAGGGGGGCCGCGAGCATGAGAAGACAGGTTGCGGTCAGGGTGGACAGGTGG

GCCGCGAGCATGAGAAGACAGGTTTCGGCCATCCAGGGACTCAGGGTGGACAGGTGGGCA

ACGAGCAGGAGAAGAAGGGTATGATTGAGAAGATCCAGGAGAAGCTGCCAGGTCACAAGT

AGGATGTGTGCCCACCATACACGTGGAGTCTACATCTACGTGCCTATTATGTTACTATAA

TACGCATGGAGTGTGTCCGGAGTCTGTAATAATCTGCGCGTATCTGTTATGTC

>Hershey313

AGCCGTTGGATCTAGTTGAAAATGTCAGTGACGCGTGATGAGTATGGCAACCAGGTTCGG

CAGACCGACGAGTATGGGAACCCGATTCAGCATACTGGCACCGGGACAAAGGCCGGTTCG

GGCATACATGGTGGGGGCCATGGGATTGGCACAGGTGGTGGTGGTGGTGGACAAGGCAAG

CTCCACCGTTCAGGCTCTGGCTCTTCCTCTGTAAGTACATTCGATGTTTCTCAAGGGTCA

TTTCACTTGGTATATCGTGTTGGGATGATTAGATTAGTTGATTAACGGTTGTGATTGTAA

ATCAGTGATATGGGCTTCTGTGTACCTTAGTTCACGATGATAATCAATTGATACGCTGGA

TCGTGATGAGCGGACCGGATGAGCCGTCCATCTTACAGGCTTTTTAAAATAGAATTATAG

GGTGGGTGGTATGAGGTCCACCAAAGCCTGTAACATGGAATTACAATCATAGCCGTTAAT

CGGTTCATCTCATCCGCTCATCGTGATCTAGCATGTAAAACAGAGATGGGTTTCTGGAGA

AATGGATGCATGTAAGCCCACAAATCACAAAAATCTGACATTATGATGGAGAGAATGCAA

GGAGTGTAAGCGAATTTACGCCGTTAGATGGATTTCATTGTACTTTTAACTTTGTTTTGT

CAAATATTTTGTAGGATGAGGATGATGGACAAGGTGGGCGTAGGAAGAAAGGTTTGACGG

AGAAGATCAAAGAGAAGCTGCCAGGTGGAAACAAGACGACAGGTGTCTGTCATCCAGGGA

CTCAGGGTGTACAGGGGGGCCGCGAGCATGAGAAGACAGGTTGCGGTCAGGGTGGTCAGG

GTGGACAGGTGGGCCGCGAGCATGAGAAGACAGGTTTCGGCCATCCAGTGACTCAGGGTG

GACAGGTGGGCAACGAGCAGGAGAAGAAGGGTATGATTGAGAAGATCCAGGAGAAGCTGC

CAGGTCACAAGTAGGATGTGTGCCCACCATACACGTGGAGTCTACATCTACGTGTCTATT

ATGTTACTATAATACGCATGGAATGTGTCCGGAGTCTGTAATAATCTGCGCGTATCTGTT

ATGTC

>Hershey314

AGCCGTTGGATCTAGTTGAAAATGTCAGTGACGCGTGATGAGTATGGCAACCAGGTTCGC

CAGACCGACGAGTATGGGAACCCGATTCAGCATACTGGCACCGGGACAAAGGCCGGTTCG

GGCATACATGGTGGGGGCCATGGGATTGGCACAGGTGGTGGTGGTGGTGGACAAGGCAAG

CTCCACCGTTCAGGCTCTGGCTCTTCCTCTGTAAGTACATTCGATGTTTCTCAAGGGTCA

TTTCACTTGGTATATCGTGTTGGGATGATTAGATTAGTTGATTAACGGTTGTGATTGTAA

ATCAGTGATATGGGCATCTGTGTACCTTAGTTCACGATGATAATCAATTGATACGCTGGA

TCGTGATGAGCGGACCGGATGAGCCGTCCATCTTACAGGCTTTTTAAAATAGAATTATAG

GGTGGGTGGTATGAGGTCCACCAAAGCCTGTAACATGGAATTACAATCATAGCCGTTAAT

CGGTTCATCTCATCCGCTCATCGTGATCTAGCATGTCAAACAGAGATGGGTTTCTGGAGA

AATGGATGTATGTAAGCCCACAAATCACAAAAATCTGACATTATGATGGAGAGAATGCAA

GGAGTGTAAGCGAATTTACGCCGTTAGATGGATTTCATTGTACTTTTAACTTTGTTTTGT

CGAATATTTTGTAGGATGAGGATGATGGACAAGGTGGGCGTAGGAAGAAGGGTTTGACGG

AGAAGATCAAAGAGAAGCTGCCAGGTGGAAACAAGACGACAGGTGTCTGTCATCCAGGGA

CTCAGGGTGTACAGGGGGGCCGCGAGCATGAGAAGACAGGTTGCGGTCAGGGTGGTCAGG

GTGGACAGGTGGGCCGCGAGCATGAGAAGACAGGTTTCGGCCATGCAGTGACTCAGGGTG

GACAGGTGGGCAACGAGCAGGAGAAGAAGGGTATGATTGAGAAGATCCAGGAGAAGCTGC

CAGGTCACAAGTAGGATGTGTGCCCACCATACACGTGGAGTCTACATCTACGTGCCTATT

ATGTTACTATAATACGCATGGAGTGTGTCCGGAGTCTGTAATAATCTGCGCGTATCTGTT

ATGTC

>Hershey61

AGCCGTTGGATCTAGTTGAAAATGTCTGAGACGCGTGATGAGTATGGCAACCAGGTTCGC

CAAACCGACGAGTATGGGAACCCGATTCAGCATACTGGCACCGGCACAAAGGCCGGTGCG

GGCATACATGCTGGGGGCCATGGGATTGGCACAGGTGGTGGTGGTGGACAAGGCAAGCTC

CACCGTTCAGGCTCCGGGTCTTCCTCTGTAAGTATATTCGATGTTTCTCAAGGGTCATTT

CACTTGGTAGATCGTGTTGTGATGACTAGATTAGTTGATTAACCGTTGTGATTGTAAATC

AGTGATATGGGCTTCTGTGTTCCTTATTTCACAATGATAATCAATTGATACGGTGGATCG

TGATGAGCGGACCGAATGAGCCGTCCATCTTACAGGCTTTTTAAAATAGAATTATAGGGT

GGGTGGTATGAGGTCCACCAAAGCCTGTAACATGGAATTACAATTATAGCCGTTAATCGG

TTCATCTCATCCGCTCATCGTGATCCAGCATGTCAAACAGAGATGGGTTTCTGGCGAAAT

GGATGTATGTAAGCCCACAAATCACAAAAATCTGACATTATGATGGAGAGAATTGCAAGG

AGTGTAAGTGAATCTACGCCGTTAGATGGATACTTTTTAACTTCGTTTTGTCAAATATTC

TGTAGGATGAGGATGATGGACAAGGTGGGCGTAGGAAGAAGGGTTTGACGGAGAAGATCA

AAGAGAAGCTGCCAGGTGGGCAGAAGACGACAGGTGTCGGTTATCCAGGGAATCAGGGTG

TACAGGGGGGCCGCGAGCATGAGAAGACAGGTGTCTGTCATCCAGGGACTCAGGGTGTAC

AGGGGGGCCGCGAGCATGAGAAGACAGGTTTCGGTCATCCAGGGACTCAGGGTGGACACG

AGCATGAGAAGACAGGTGTCGGTCATCCAGGGACTCAGGGTGAGCAGGAGAAGAAGGGTA

TGATTGAGAAGATCAAGGAGAAGCTGCCAGGTCACAAGTAGGATGTGTGCCCACCATACA

CGTGGAGTCTACATCTATGTTTCTATGTTACTATAATACGCATGGAGTGTGTACGGATTC

TGTAATAATCTGCGCGTATCTGTTATGTC

>Hershey64

AGCCGTTGGATCTAGTTGAAAATGTCTGAGACGCGTGATGAGTATGGCAACCAGGTTCGC

CAAACCGACGAGTATGGGAACCCGATTCAGCATACTGGCACCGGCACAAAGGCCGGTGCG

GGCATACATGCTGGGGGCCATGGGATTGGCACAGGTGGTGGTGGTGGACAAGGCAAGCTC

CACCGTTCAGGCTCCGGGTCTTCCTCTGTAAGTATATTCGATGTTTCTCAAGGGTCATTT

CACTTGGTAGATCGTGTTGTGATGACTAGATTAGTTGATTAACCGTTGTGATTGTAAATC

AGTGATATGGGCTTCTGTGTTCCTTATTTCACAATGATAATCAATTGATACGGTGGATCG

TGATGAGCGGACCGAATGAGCCGTCCATCTTACAGGCTTTTTAAAATAGAATTATAGGGT

GGGTGGTATGAGGTCCACCAAAGCCTGTAACATGGAATTACAATTATAGCCGTTAATCGG

TTCATCTCATCCGCTCATCGTGATCCAGCATGTCAAACAGAGATGGGTTTCTGGCGAAAT

GGATGTATGTAAGCCCACAAATCACAAAAATCTGACATTATGATGGAGAGAATTGCAAGG

AGTGTAAGTGAATCTACGCCGTTAGATGGATACTTTTTAACTTCGTTTTGTCAAATATTC

TGTAGGATGAGGATGATGGACAAGGTGGGCGTAGGAAGAAGGGTTTGACGGAGAAGATCA

AAGAGAAGCTGCCAGGTGGGCAGAAGACGACAGGTGTCGGTTATCCAGGGAATCAGGGTG

TACAGGGGGGCCGCGAGCATGAGAAGACAGGTGTCTGTCATCCAGGGACTCAGGGTGTAC

AGGGGGGCCGCGAGCATGAGAAGACAGGTTTCGGTCATCCAGGGACTCAGGGTGGACACG

AGCATGAGAAGACAGGTGTCGGTCATCCAGGGACTCAGGGTGAGCAGGAGAAGAAGGGTA

TGATTGAGAAGATCAAGGAGAAGCTGCCAGGTCACAAGTAGGATGTGTGCCCACCATACA

CGTGGAGTCTACATCTATGTTTCTATGTTACTATAATACGCATGGAGTGTGTACGGATTC

TGTAATAATCTGCGCGTATCTGTTATGTC

>Hershey68

AGCCGTTGGATCTAGTTGAAAATGTCTGAGACGCGTGATGAGTATGGCAACCAGGTTCGC

CAAACCGACGAGTATGGGAACCCGATTCAGCATACTGGCACCGGCACAAAGGCCGGTGCG

GGCATACATGCTGGGGGCCATGGGATTGGCACAGGTGGTGGTGGTGGACAAGGCAAGCTC

CACCGTTCAGGCTCCGGGTCTTCCTCTGTAAGTATATTCGATGTTTCTCAAGGGTCATTT

CACTTGGTAGATCGTGTTGTGATGACTAGATTAGTTGATTAACCGTTGTGATTGTAAATC

AGTGATATGGGCTTCTGTGTTCCTTATTTCACGATGATAATCAATTGATACGGTGGATCG

TGATGAGCGGACCGGATGAGCCGTCCATCTTACAGGCTTTTTAAAATAGAATTATAGGGT

GGGTGGTATGAGGTCCACCAAAGCCTGTAACATGGAATTACAATCATAGCCGTTAATCGG

TTCATCTCATCCGCTCATCGTGATCCAGCATGTCAAACAGAGATGGGTTTCTGGCGAAAT

GGATGTATGTAAGCCCACAAATCACAAAAATCTGACATTATGATGGAGAGAATTGCAAGG

AGTGTAAGTGAATCTACGCCGTTAGATGGATACTTCAAGGAGTGTAAGTGAATCTACGCC

GTTAGATGGATACTTTTTAACTTCGTTTTGTCAAATATTCTGTAGGATGAGGATGATGGA

CAAGGTGGGCGTAGGAAGAAGGGTTTGACGGAGAAGATCAAAGAGAAGCTGCCAGGTGGG

CAGAAGACGACGACAGGTGTGGGTTATCCAGGGAATCAGGGTGTACAGGGGGGCCGCGAG

CATGAGAAGACAGGTGTCTGTCATCCAGGGACTCAGGGTGTACAGGGGGGCCGCGAGCAT

GAGAAGACAGGTTTCGGTCATCCAGGGACTCAGGGTGGACACGAGCATGAGAAGACAGGT

GTCGGTCATCCAGGGACTCAGGGTGAGCAGGAGAAGAAGGGTATGATTGAGAAGATCAAG

GAGAAGCTGCCAGGTCACAAGTAGGATGTGTGCCCACCATACACGTGGAGTCTACATCTA

TGTTTCTATGTTACTATAATACGCATGGAGTGTGTACGGATTCTGTAATAATATGCGCGT

ATCTGTTATGTC

>Hershey71

AGCCGTTGGATCTAGTTGAAAATGTCAGTGACGCGTGATGAGTATGGCAACCAGGTTCGC

CAGACCGACGAGTATGGGAACCCGATTCAGCATACTGGCACCGGGACAAAGGCCGGTTCG

GGCATACATGGTGGGGGCCATGGGATTGGCACAGGTGGTGGTGGTGGTGGACAAGGCAAG

CTCCACCGTTCAGGCTCTGGCTCTTCCTCTGTAAGTACATTCGATGTTTCTCAAGGGTCA

TTTCACTTGGTATATCGTGTTGGGATGATTAGATTAGTTGATTAACGGTTGTGATTGTAA

ATCAGTGATATGGGCATCTGTGTACCTTAGTTCACGATGATAATCAATTGATACGCTGGA

TCGTGATGAGCGGACCGGATGAGCCGTCCATCTTACAGGCTTTTTAAAATAGAATTATAG

GGTGGGTGGTATGAGGTCCACCAAAGCCTGTAACATGGAATTACAATCATAGCCGTTAAT

CGGTTCATCTCATCCGCTCATCGTGATCTAGCATGTCAAACAGAGATGGGTTTCTGGAGA

AATGGATGTATGTAAGCCCACAAATCACAAAAATCTGACATTATGATGGAGAGAATGCAA

GGAGTGTAAGCGAATTTACGCCGTTAGATGGATTTCATTGTACTTTTAACTTTGTTTTGT

CGAATATTTTGTAGGATGAGGATGATGGACAAGGTGGGCGTAGGAAGAAGGGTTTGACGG

AGAAGATCAAAGAGAAGCTGCCAGGTGGAAACAAGACGACAGGTGTCTGTCATCCAGGGA

CTCAGGGTGTACAGGGGGGCCGCGAGCATGAGAAGACAGGTTGCGGTCAGGGTGGTCAGG

GTGGACAGGTGGGCCGCGAGCATGAGAAGACAGGTTTCGGCCATGCAGTGACTCAGGGTG

GACAGGTGGGCAACGAGCAGGAGAAGAAGGGTATGATTGAGAAGATCAAGGAGAAGCTGC

CAGGTCACAAGTAGGATGTGTGCCCACCATACACGTGGAGTCTACATCTATGTTTCTATG

TTACTATAATACGCATGGAGTGTGTACGGATTATGTAATAATCTGCGCGTATCTGTTATG

TC

>Hershey72

AGCCGTTGGATCTAGTTGAAAATGTCTGAGACGCGTGATGAGTATGGCAACCAGGTTCGC

CAAACCGACGAGTATGGGAACCCGATTCAGCATACTGGCACCGGGACAAAGGGCGGTGCG

GGAATACATGGTGGGGGTCATGGGATTGGCACAGGTGGTGGTGGTGGACAAGGCAAGCTC

CACCGTTCAGGCTCCGGCTCTTCCTCTGTAAGTATATTAGATGTTTCTCAAGAGTCATTT

CACTTGGTAGATTGTGTTGGGATGACTAGATTAGTTGATTAACGGTTGCGATTGTAAATC

AGTGATATGGGCTTCTGTCTACCTTATTTCATGATGATAATCAATTGATACGGTGGATCG

TGATGAGCGGATCGGATGAGCCGTACATCTTACAGGCTTTTTAAAATAGAATTATAGGGT

GGGTGGTATGAGGTCCACCAAAGCCTGTAACATGGAATTACAATCATAGCCGTTAATAGG

TTCATCTCATCCGCTCATCGTGATCCAGCATGTCAAACAGAGATGGGTTTCTGGCGAAAT

GGATGTATGTAAGCCCACAAATCACAAAAATCTGACATTATGATGGAGAGAATTGCAAGG

AGTGCAAGTGAATCTACGCCGTTAGATGGATACTTTTTAACTTCGTTTTGTCAAATATTC

CGTAGGATGAGGATGATGGACAAGGTGGGCGTAGGAAGAAGGGTTTGACGGAGAAGATCA

AAGAGAAGCTGCCAGGTGGAAACAAGACGACAGGTGTCTGTCATCCAGGGACTCAGGGTG

TACAGGGGGGCCGCGAGCATGAGAAGACAGGTTGCGGTCAGGGTGGTCAGGGTGGACAGG

TGGGCCGCGAGCATGAGAAGACAGGTTTCGGCCATGCAGTGACTCAGGGTGGACAGGTGG

GCAACGAGCAGGAGAAGAAGGGTATGATTGAGAAGATCCAGGAGAAGCTGCCAGGTCACA

AGTAGGATGTGTGCCCACCATACACGTGGAGTCTACATCTACGTGCCTATTATGTTACTA

TAATACGCATGGAGTGTGTCCGGAGTCTGTAATAATCTGCGCGTATCTGTTATGTC

>BK12

AGCCGTTGGATCTAGTTGAAAATGTCTGAGACGCGTGATGAGTATGGCAACGAGGTTCGC

CAAACCGACGAGTATGGGAACCCGATTCAGCATACTGGCACCGGGACAAAGGGCGGTGCG

GGAATACATGGTGGGGGTCATGGGATTGGCACAGGTGGTGGTGGTGGACAAGGCAAGCTC

CACCGTTCAGGCTCCGGCTCTTCCTCTGTAAGTATATTAGATGTTTCTCAAGAGTCATTT

CACTTGGTAGATTGTGTTGGGATGACTAGATTAGTTGATTAACGGTTGCGATTGTAAATC

AGTGATATGGGCTTCTGTAGACCTTATTTCACGATGATAATCAATTGATACGGTGGATCG

TGATGAGCGGATCGGATGAGCCGTACATCTTACAGGCTTTTTAAAATAGAATTATAGGGT

GGGTGGTATGAGGTCCACCAAAGCCTGTAACATGGAATTACAATCATAGCCGTTAATCGG

TTCATCTCATCCGCTCATCGTGATCCAGCATGTCAAACGGAGATGGGTTACTGGCGAAAT

GGATGTATGTAAGCCCACAAATCACAAAAATCTGACATTATGATGGAGAGAATTGCAAGG

AGTGTAAGTGAATCTACGCCGTTAGATGGATACTTTTTAACTTCGTTTTGTCAAATATTC

TGTAGGATGAGGATGATGGACAAGGTGGGCGTAGGAAGAAGGGTTTGACGGAGAAGATCA

AAGAGAAGCTGCCAGGTGGGCAGAAGACGACGACAGGTGTGGGTTATCCAGGGAATCAGG

GTGTACAGGGGGGCCGCGAGCATGAGAAGACAGGTGTCTGTCATCCAGGGACTCAGGGTG

TACAGGGGGGCCGCGAGCATGAGAAGACAGGTTTCGGTCATCCAGGGACTCAGGGTGGAC

ACGAGCATGAGAAGACAGGTGTCGGTCATCCAGGGACTCAGGGTGAGCAGGAGAAGAAGG

GTATGATTGAGAAGATCAAGGAGAAGCTGCCAGGTCACAAGTAGGATGTGTGCCCACCAT

ACACGTGGAGTCTACATCTATGTTTCTATGTTACTATAATACGCATGGAGTGTGTACGGA

TTCTGTAATAATCTGCGCGTATCTGTTATGTC

>BK14

AGCCGTTGGATCTAGTTGAAAATGTCTGAGACGCGTGATGAGTATGGCAACGAGGTTCGC

CAAACCGACGAGTATGGGAACCCGATTCAGCATACTGGCACCGGGACAAAGGGCGGTGCG

GGAATACATGGTGGGGGTCATGGGATTGGCACAGGTGGTGGTGGTGGACAAGGCAAGCTC

CACCGTTCAGGCTCCGGCTCTTCCTCTGTAAGTATATTAGATGTTTCTCAAGAGTCATTT

CACTTGGTAGATTGTGTTGGGATGACTAGATTAGTTGATTAACGGTTGCGATTGTAAATC

AGTGATATGGGCTTCTGTAGACCTTATTTCACGATGATAATCAATTGATACGGTGGATCG

TGATGAGCGGATCGGATGAGCCGTACATCTTACAGGCTTTTTAAAATAGAATTATAGGGT

GGGTGGTATGAGGTCCACCAAAGCCTGTAACATGGAATTACAATCATAGCCGTTAATCGG

TTCATCTCATCCGCTCATCGTGATCCAGCATGTCAAACGGAGATGGGTTACTGGCGAAAT

GGATGTATGTAAGCCCACAAATCACAAAAATCTGACATTATGATGGAGAGAATTGCAAGG

AGTGTAAGTGAATCTACGCCGTTAGATGGATACTTTTTAACTTCGTTTTGTCAAATATTC

TGTAGGATGAGGATGATGGACAAGGTGGGCGTAGGAAGAAGGGTTTGACGGAGAAGATCA

AAGAGAAGCTGCCAGGTGGGCAGAAGACGACGACAGGTGTGGGTTATCCAGGGAATCAGG

GTGTACAGGGGGGCCGCGAGCATGAGAAGACAGGTGTCTGTCATCCAGGGACTCAGGGTG

TACAGGGGGGCCGCGAGCATGAGAAGACAGGTTTCGGTCATCCAGGGACTCAGGGTGGAC

ACGAGCATGAGAAGACAGGTGTCGGTCATCCAGGGACTCAGGGTGAGCAGGAGAAGAAGG

GTATGATTGAGAAGATCAAGGAGAAGCTGCCAGGTCACAAGTAGGATGTGTGCCCACCAT

ACACGTGGAGTCTACATCTATGTTTCTATGTTACTATAATACGCATGGAGTGTGTACGGA

TTCTGTAATAATCTGCGCGTATCTGTTATGTC

>BK23

AGCCGTTGGATCTAGTTGAAAATGTCAGTGACGCGTGATGAGTATGGCAACCAGGTTCGG

CAGACCGACGAGTATGGGAACCCGATTCAGCATACTGGCACCGGGACAAAGGCCGGTTCG

GGCATACATGGTGGGGGCCATGGGATTGGCACAGGTGGTGGTGGTGGTGGACAAGGCAAG

CTCCACCGTTCAGGCTCTGGCTCTTCCTCTGTAAGTACATTCGATGTTTCTCAAGGGTCA

TTTCACTTGGTATATCGTGTTGGGATGATTAGATTAGTTGATTAACGGTTGTGATTGTAA

ATCAGTGATATGGGCTTCTGTGTACCTTAGTTCACGATGATAATCAATTGATACGCTGGA

TCGTGATGAGCGGACCGGATGAGCCGTCCATCTTACAGGCTTTTTAAAATAGAATTATAG

GGTGGGTGGTATGAGGTCCACCAAAGCCTGTAACATGGAATTACAATCATAGCCGTTAAT

CGGTTCATCTCATCCGCTCATCGTGATCTAGCATGTAAAACAGAGATGGGTTTCTGGAGA

AATGGATGCATGTAAGCCCACAAATCACAAAAATCTGACATTATGATGGAGAGAATGCAA

GGAGTGTAAGCGAATTTACGCCGTTAGATGGATTTCATTGTACTTTTAACTTTGTTTTGT

CAAATATTTTGTAGGATGAGGATGATGGACAAGGTGGGCGTAGGAAGAAAGGTTTGACGG

AGAAGATCAAAGAGAAGCTGCCAGGTGGAAACAAGACGACAGGTGTCTGTCATCCAGGGA

CTCAGGGTGTACAGGGGGGCCGCGAGCATGAGAAGACAGGTTGCGGTCAGGGTGGTCAGG

GTGGACAGGTGGGCCGCGAGCATGAGAAGACAGGTTTCGGCCATCCAGTGACTCAGGGTG

GACAGGTGGGCAACGAGCAGGAGAAGAAGGGTATGATTGAGAAGATCCAGGAGAAGCTGC

CAGGTCACAAGTAGGATGTGTGCCCACCATACACGTGGAGTCTACATCTACGTGTCTATT

ATGTTACTATAATACGCATGGAATGTGTCCGGAGTCTGTAATAATCTGCGCGTATCTGTT

ATGTC

>BK261

AGCCGTTGGATCTAGTTGAAAATGTCAGTGACGCGTGATGAGTATGGCAACCAGGTTCGG

CAGACCGACGAGTATGGGAACCCGATTCAGCATACTGGCACCGGGACAAAGGCCGGTTCG

GGCATACATGGTGGGGGCCATGGGATTGGCACAGGTGGTGGTGGTGGTGGACAAGGCAAG

CTCCACCGTTCAGGCTCTGGCTCTTCCTCTGTAAGTACATTCGATGTTTCTCAAGGGTCA

TTTCACTTGGTATATCGTGTTGGGATGATTAGATTAGTTGATTAACGGTTGTGATTGTAA

ATCAGTGATATGGGCTTCTGTGTACCTTAGTTCACGATGATAATCAATTGATACGCTGGA

TCGTGATGAGCGGACCGGATGAGCCGTCCATCTTACAGGCTTTTTAAAATAGAATTATAG

GGTGGGTGGTATGAGGTCCACCAAAGCCTGTAACATGGAATTACAATCATAGCCGTTAAT

CGGTTCATCTCATCCGCTCATCGTGATCTAGCATGTAAAACAGAGATGGGTTTCTGGAGA

AATGGATGCATGTAAGCCCACAAATCACAAAAATCTGACATTATGATGGAGAGAATGCAA

GGAGTGTAAGCGAATTTACGCCGTTAGATGGATTTCATTGTACTTTTAACTTTGTTTTGT

CAAATATTTTGTAGGATGAGGATGATGGACAAGGTGGGCGTAGGAAGAAAGGTTTGACGG

AGAAGATCAAAGAGAAGCTGCCAGGTGGAAACAAGACGACAGGTGTCTGTCATCCAGGGA

CTCAGGGTGTACAGGGGGGCCGCGAGCATGAGAAGACAGGTTGCGGTCAGGGTGGTCAGG

GTGGACAGGTGGGCCGCGAGCATGAGAAGACAGGTTTCGGCCATCCAGTGACTCAGGGTG

GACAGGTGGGCAACGAGCAGGAGAAGAAGGGTATGATTGAGAAGATCCAGGAGAAGCTGC

CAGGTCACAAGTAGGATGTGTGCCCACCATACACGTGGAGTCTACATCTACGTGTCTATT

ATGTTACTATAATACGCATGGAATGTGTCCGGAGTCTGTAATAATCTGCGCGTATCTGTT

ATGTC

>BK27

AGCCGTTGGATCTAGTTGAAAATGTCAGTGACGCGTGATGAGTATGGCAACCAGGTTCGG

CAGACCGACGAGTATGGGAACCCGATTCAGCATACTGGCACCGGGACAAAGGCCGGTTCG

GGCATACATGGTGGGGGCCATGGGATTGGCACAGGTGGTGGTGGTGGTGGACAAGGCAAG

CTCCACCGTTCAGGCTCTGGCTCTTCCTCTGTAAGTACATTCGATGTTTCTCAAGGGTCA

TTTCACTTGGTATATCGTGTTGGGATGATTAGATTAGTTGATTAACGGTTGTGATTGTAA

ATCAGTGATATGGGCTTCTGTGTACCTTAGTTCACGATGATAATCAATTGATACGCTGGA

TCGTGATGAGCGGACCGGATGAGCCGTCCATCTTACAGGCTTTTTAAAATAGAATTATAG

GGTGGGTGGTATGAGGTCCACCAAAGCCTGTAACATGGAATTACAATCATAGCCGTTAAT

CGGTTCATCTCATCCGCTCATCGTGATCTAGCATGTAAAACAGAGATGGGTTTCTGGAGA

AATGGATGCATGTAAGCCCACAAATCACAAAAATCTGACATTATGATGGAGAGAATGCAA

GGAGTGTAAGCGAATTTACGCCGTTAGATGGATTTCATTGTACTTTTAACTTTGTTTTGT

CAAATATTTTGTAGGATGAGGATGATGGACAAGGTGGGCGTAGGAAGAAAGGTTTGACGG

AGAAGATCAAAGAGAAGCTGCCAGGTGGAAACAAGACGACAGGTGTCTGTCATCCAGGGA

CTCAGGGTGTACAGGGGGGCCGCGAGCATGAGAAGACAGGTTGCGGTCAGGGTGGTCAGG

GTGGACAGGTGGGCCGCGAGCATGAGAAGACAGGTTTCGGCCATCCAGTGACTCAGGGTG

GACAGGTGGGCAACGAGCAGGAGAAGAAGGGTATGATTGAGAAGATCCAGGAGAAGCTGC

CAGGTCACAAGTAGGATGTGTGCCCACCATACACGTGGAGTCTACATCTACGTGTCTATT

ATGTTACTATAATACGCATGGAATGTGTCCGGAGTCTGTAATAATCTGCGCGTATCTGTT

ATGTC

>BK31

AGCCGTTGGATCTAGTTGAAAATGTCTGAGACGCGTGATGAGTATGGCAACCAGGTTCGC

CAAACCGACGAGTATGGGAACCCGATTCAGCATACTGGCACCGGGACAAAGGGCGGTGCG

GGAATACATGGTGGGGGTCATGGGATTGGCACAGGTGGTGGTGGTGGACAAGGCAAGCTC

CACCGTTCAGGCTCCGGCTCTTCCTCTGTAAGTACATTCGATGTTTCTCAAGGGTCATTT

CACTTGGTATATCGTGTTGGGATGATTAGATTAGTTGATTAACGGTTGTGATTGTAAATC

AGTGATATGGGCTTCTGTGTACCTTAGTTCACGATGATAATCAATTGATACGCTGGATCG

TGATGAGCGGACCGGATGAGCCGTCCATCTTACAGGCTTTTTAAAATAGAATTATAGGGT

GGGTGGTATGAGGTCCACCAAAGCCTGTAACATGGAATTACAATCATAGCCGTTAATCGG

TTCATCTCATCCGCTCATCGTGATCTAGCATGTAAAACAGAGATGGGTTTCTGGAGAAAT

GGATGCATGTAAGCCCACAAATCACAAAAATCTGACATTATGATGGAGAGAATGCAAGGA

GTGTAAGCGAATTTACGCCGTTAGATGGATTTCATTGTACTTTTAACTTTGTTTTGTCAA

ATATTTTGTAGGATGAGGATGATGGACAAGGTGGGCGTAGGAAGAAAGGTTTGACGGAGA

AGATCAAAGAGAAGCTGCCAGGTGGAAACAAGACGACAGGTGTCTGTCATCCAGGGACTC

AGGGTGTACAGGGGGGCCGCGAGCATGAGAAGACAGGTTGCGGTCAGGGTGGTCAGGGTG

GACAGGTGGGCCGCGAGCATGAGAAGACAGGTTTCGGCCATCCAGTGACTCAGGGTGGAC

AGGTGGGCAACGAGCAGGAGAAGAAGGGTATGATTGAGAAGATCCAGGAGAAGCTGCCAG

GTCACAAGTAGGATGTGTGCCCACCATACACGTGGAGTCTACATCTACGTGTCTATTATG

TTACTATAATACGCATGGAATGTGTCCGGAGTCTGTAATAATCTGCGCGTATCTGTTATG

TC

>BK38

AGCCGTTGGATCTAGTTGAAAATGTCTGAGACGCGTGATGAGTATGGCAACCAGGTTCGC

CAAACCGACGAGTATGGGAACCCGATTCAGCATACTGGCACCGGGACAAAGGGCGGTGCG

GGAATACATGGTGGGGGTCATGGGATTGGCACAGGTGGTGGTGGTGGACAAGGCAAGCTC

CACCGTTCAGGCTCCGGCTCTTCCTCTGTAAGTACATTCGATGTTTCTCAAGGGTCATTT

CACTTGGTATATCGTGTTGGGATGATTAGATTAGTTGATTAACGGTTGTGATTGTAAATC

AGTGATATGGGCTTCTGTGTACCTTAGTTCACGATGATAATCAATTGATACGCTGGATCG

TGATGAGCGGACCGGATGAGCCGTCCATCTTACAGGCTTTTTAAAATAGAATTATAGGGT

GGGTGGTATGAGGTCCACCAAAGCCTGTAACATGGAATTACAATCATAGCCGTTAATCGG

TTCATCTCATCCGCTCATCGTGATCTAGCATGTAAAACAGAGATGGGTTTCTGGAGAAAT

GGATGCATGTAAGCCCACAAATCACAAAAATCTGACATTATGATGGAGAGAATGCAAGGA

GTGTAAGCGAATTTACGCCGTTAGATGGATTTCATTGTACTTTTAACTTTGTTTTGTCAA

ATATTTTGTAGGATGAGGATGATGGACAAGGTGGGCGTAGGAAGAAAGGTTTGACGGAGA

AGATCAAAGAGAAGCTGCCAGGTGGAAACAAGACGACAGGTGTCTGTCATCCAGGGACTC

AGGGTGTACAGGGGGGCCGCGAGCATGAGAAGACAGGTTGCGGTCAGGGTGGTCAGGGTG

GACAGGTGGGCCGCGAGCATGAGAAGACAGGTTTCGGCCATCCAGTGACTCAGGGTGGAC

AGGTGGGCAACGAGCAGGAGAAGAAGGGTATGATTGAGAAGATCCAGGAGAAGCTGCCAG

GTCACAAGTAGGATGTGTGCCCACCATACACGTGGAGTCTACATCTACGTGTCTATTATG

TTACTATAATACGCATGGAATGTGTCCGGAGTCTGTAATAATCTGCGCGTATCTGTTATG

TC

>BK381

AGCCGTTGGATCTAGTTGAAAATGTCTGAGACGCGTGATGAGTATGGCAACGAGGTTCGC

CAAACCGACGAGTATGGGAACCCGATTCAGCATACTGGCACCGGGACAAAGGGCGGTGCG

GGAATACATGGTGGGGGTCATGGGATTGGCACAGGTGGTGGTGGTGGACAAGGCAAGCTC

CACCGTTCAGGCTCCGGCTCTTCCTCTGTAAGTATATTAGATGTTTCTCAAGAGTCATTT

CACTTGGTAGATTGTGTTGGGATGACTAGATTAGTTGATTAACGGTTGCGATTGTAAATC

AGTGATATGGGCTTCTGTAGACCTTATTTCACGATGATAATCAATTGATACGGTGGATCG

TGATGAGCGGATCGGATGAGCCGTACATCTTACAGGCTTTTTAAAATAGAATTATAGGGT

GGGTGGTATGAGGTCCACCAAAGCCTGTAACATGGAATTACAATCATAGCCGTTAATCGG

TTCATCTCATCCGCTCATCGTGATCCAGCATGTCAAACGGAGATGGGTTACTGGCGAAAT

GGATGTATGTAAGCCCACAAATCACAAAAATCTGACATTATGATGGAGAGAATTGCAAGG

AGTGTAAGTGAATCTACGCCGTTAGATGGATACTTTTTAACTTCGTTTTGTCAAATATTC

TGTAGGATGAGGATGATGGACAAGGTGGGCGTAGGAAGAAGGGTTTGACGGAGAAGATCA

AAGAGAAGCTGCCAGGTGGGCAGAAGACGACGACAGGTGTGGGTTATCCAGGGAATCAGG

GTGTACAGGGGGGCCGCGAGCATGAGAAGACAGGTGTCTGTCATCCAGGGACTCAGGGTG

TACAGGGGGGCCGCGAGCATGAGAAGACAGGTTTCGGTCATCCAGGGACTCAGGGTGGAC

ACGAGCATGAGAAGACAGGTGTCGGTCATCCAGGGACTCAGGGTGAGCAGGAGAAGAAGG

GTATGATTGAGAAGATCAAGGAGAAGCTGCCAGGTCACAAGTAGGATGTGTGCCCACCAT

ACACGTGGAGTCTACATCTATGTTTCTATGTTACTATAATACGCATGGAGTGTGTACGGA

TTCTGTAATAATCTGCGCGTATCTGTTATGTC

>BK53

AGCCGTTGGATCTAGTTGAAAATGTCTGAGACGCGTGATGAGTATGGCAACGAGGTTCGC

CAAACCGACGAGTATGGGAACCCGATTCAGCATACTGGCACCGGGACAAAGGGCGGTGCG

GGAATACATGGTGGGGGTCATGGGATTGGCACAGGTGGTGGTGGTGGACAAGGCAAGCTC

CACCGTTCAGGCTCCGGCTCTTCCTCTGTAAGTATATTAGATGTTTCTCAAGAGTCATTT

CACTTGGTAGATTGTGTTGGGATGACTAGATTAGTTGATTAACGGTTGCGATTGTAAATC

AGTGATATGGGCTTCTGTAGACCTTATTTCACGATGATAATCAATTGATACGGTGGATCG

TGATGAGCGGATCGGATGAGCCGTACATCTTACAGGCTTTTTAAAATAGAATTATAGGGT

GGGTGGTATGAGGTCCACCAAAGCCTGTAACATGGAATTACAATCATAGCCGTTAATCGG

TTCATCTCATCCGCTCATCGTGATCCAGCATGTCAAACGGAGATGGGTTACTGGCGAAAT

GGATGTATGTAAGCCCACAAATCACAAAAATCTGACATTATGATGGAGAGAATTGCAAGG

AGTGTAAGTGAATCTACGCCGTTAGATGGATACTTTTTAACTTCGTTTTGTCAAATATTC

TGTAGGATGAGGATGATGGACAAGGTGGGCGTAGGAAGAAGGGTTTGACGGAGAAGATCA

AAGAGAAGCTGCCAGGTGGGCAGAAAACGACAGGTGTCGGTTATCCAGGGAATCAGGGTG

TACAGGGGGGCCGCGAGCATGAGAAGACAGGTGTCTGTCATCCAGGGAATCAGGGTGTAC

AGGGGGGCCGCGAGCATGAGAAGACAGGTTTCGGTCATCCAGGGACTCAGGGTGGACACG

AGCATGAGAAGACAGGTGTCGGTCATCCAGGGACTCAGGGTGAGCAGGAGAAGAAGGGTA

TGATTGAGAAGATCAAGGAGAAGCTGCCAGGTCACAAGTAGGATGTGTGCCCACCATACA

CGTGGAGTCTACATCTATGTTTCTATGTTACTATAATACGCATGGAGTGTGTACGGATTC

TGTAATAATCTGCGCGTATCTGTTATGTC

>BK511

AGCCGTTGGATCTAGTTGAAAATGTCTGAGACGCGTGATGAGTATGGCAACGAGGTTCGC

CAAACCGACGAGTATGGGAACCCGATTCAGCATACTGGCACCGGGACAAAGGGCGGTGCG

GGAATACATGGTGGGGGTCATGGGATTGGCACAGGTGGTGGTGGTGGACAAGGCAAGCTC

CACCGTTCAGGCTCCGGCTCTTCCTCTGTAAGTATATTAGATGTTTCTCAAGAGTCATTT

CACTTGGTAGATTGTGTTGGGATGACTAGATTAGTTGATTAACGGTTGCGATTGTAAATC

AGTGATATGGGCTTCTGTAGACCTTATTTCACGATGATAATCAATTGATACGGTGGATCG

TGATGAGCGGATCGGATGAGCCGTACATCTTACAGGCTTTTTAAAATAGAATTATAGGGT

GGGTGGTATGAGGTCCACCAAAGCCTGTAACATGGAATTACAATCATAGCCGTTAATCGG

TTCATCTCATCCGCTCATCGTGATCCAGCATGTCAAACGGAGATGGGTTACTGGCGAAAT

GGATGTATGTAAGCCCACAAATCACAAAAATCTGACATTATGATGGAGAGAATTGCAAGG

AGTGTAAGTGAATCTACGCCGTTAGATGGATACTTTTTAACTTCGTTTTGTCAAATATTC

TGTAGGATGAGGATGATGGACAAGGTGGGCGTAGGAAGAAGGGTTTGACGGAGAAGATCA

AAGAGAAGCTGCCAGGTGGGCAGAAAACGACAGGTGTCGGTTATCCAGGGAATCAGGGTG

TACAGGGGGGCCGCGAGCATGAGAAGACAGGTGTCTGTCATCCAGGGAATCAGGGTGTAC

AGGGGGGCCGCGAGCATGAGAAGACAGGTTTCGGTCATCCAGGGACTCAGGGTGGACACG

AGCATGAGAAGACAGGTGTCGGTCATCCAGGGACTCAGGGTGAGCAGGAGAAGAAGGGTA

TGATTGAGAAGATCAAGGAGAAGCTGCCAGGTCACAAGTAGGATGTGTGCCCACCATACA

CGTGGAGTCTACATCTATGTTTCTATGTTACTATAATACGCATGGAGTGTGTACGGATTC

TGTAATAATCTGCGCGTATCTGTTATGTC

>BK513

AGCCGTTGGATCTAGTTGAAAATGTCTGAGACGCGTGATGAGTATGGCAACGAGGTTCGC

CAAACCGACGAGTATGGGAACCCGATTCAGCATACTGGCACCGGGACAAAGGGCGGTGCG

GGAATACATGGTGGGGGTCATGGGATTGGCACAGGTGGTGGTGGTGGACAAGGCAAGCTC

CACCGTTCAGGCTCCGGCTCTTCCTCTGTAAGTATATTAGATGTTTCTCAAGAGTCATTT

CACTTGGTAGATTGTGTTGGGATGACTAGATTAGTTGATTAACGGTTGCGATTGTAAATC

AGTGATATGGGCTTCTGTAGACCTTATTTCACGATGATAATCAATTGATACGGTGGATCG

TGATGAGCGGATCGGATGAGCCGTACATCTTACAGGCTTTTTAAAATAGAATTATAGGGT

GGGTGGTATGAGGTCCACCAAAGCCTGTAACATGGAATTACAATCATAGCCGTTAATCGG

TTCATCTCATCCGCTCATCGTGATCCAGCATGTCAAACGGAGATGGGTTACTGGCGAAAT

GGATGTATGTAAGCCCACAAATCACAAAAATCTGACATTATGATGGAGAGAATTGCAAGG

AGTGTAAGTGAATCTACGCCGTTAGATGGATACTTTTTAACTTCGTTTTGTCAAATATTC

TGTAGGATGAGGATGATGGACAAGGTGGGCGTAGGAAGAAGGGTTTGACGGAGAAGATCA

AAGAGAAGCTGCCAGGTGGGCAGAAAACGACAGGTGTCGGTTATCCAGGGAATCAGGGTG

TACAGGGGGGCCGCGAGCATGAGAAGACAGGTGTCTGTCATCCAGGGAATCAGGGTGTAC

AGGGGGGCCGCGAGCATGAGAAGACAGGTTTCGGTCATCCAGGGACTCAGGGTGGACACG

AGCATGAGAAGACAGGTGTCGGTCATCCAGGGACTCAGGGTGAGCAGGAGAAGAAGGGTA

TGATTGAGAAGATCAAGGAGAAGCTGCCAGGTCACAAGTAGGATGTGTGCCCACCATACA

CGTGGAGTCTACATCTATGTTTCTATGTTACTATAATACGCATGGAGTGTGTACGGATTC

TGTAATAATCTGCGCGTATCTGTTATGTC

>BK61

AGCCGTTGGATCTAGTTGAAAATGTCAGTGACGCGTGATGAGTATGGCAACCAGGTTCGC

CAGACCGACGAGTATGGGAACCCGATTCAGCATACTGGCACCGGGACAAAGGCCGGTTCG

GGCATACATGGTGGGGGCCATGGGATTGGCACAGGTGGTGGTGGTGGTGGACAAGGCAAG

CTCCACCGTTCAGGCTCTGGCTCTTCCTCTGTAAGTACATTCGATGTTTCTCAAGGGTCA

TTTCACTTGGTATATCGTGTTGGGATGATTAGATTAGTTGATTAACGGTTGTGATTGTAA

ATCAGTGATATGGGCATCTGTGTACCTTAGTTCACGATGATAATCAATTGATACGCTGGA

TCGTGATGAGCGGACCGGATGAGCCGTACATCTTACAGGCTTTTTAAAATAGAATTATAG

GGTGGGTGGTATGAGGTCCACCAAAGCCTGTAACATGGAATTACAATCATAGCCGTTAAT

CGGTTCATCTCATCCGCTCATCGTGATCCAGCATGTCAAACGGAGATGGGTTACTGGCGA

AATGGATGTATGTAAGCCCACAAATCACAAAAATCTGACATTATGATGGAGAGAATTGCA

AGGAGTGTAAGTGAATTTACGCCGTTAGATGGATACTTTTTAACTTCGTTTTGTCAAATA

TTCTGTAGGATGAGGATGATGGACAAGGTGGGCGTAGGAAGAAGGGTTTGACGGAGAAGA

TCAAAGAGAAGCTGCCAGGTGGGCAGAAGACGACAGGTGTCGGTTATCCAGGGAATCAGG

GTGTACAGGGGGGCCGCGAGCATGAGAAGACAGGTGTCTGTCATCCAGGGACTCAGGGTG

TACAGGGGGGCCGCGAGCATGAGAAGACAGGTTTCGGTCATCCAGGGACTCAGGGTGGAC

ACGAGCATGAGAAGACAGGTGTCGGTCATCCAGGGACTCAGGGTGAGCAGGAGAAGAAGG

GTATGATTGAGAAGATCAAGGAGAAGCTGCCAGGTCACAAGTAGGATGTGTGCCCACCAT

ACACGTGGAGTCTACATCTATGTTTCTATGTTACTATAATACGCATGGAGTGTGTACGGA

TTCTGTAATAATCTGCGCGTATCTGTTATGTC

>BK610

AGCCGTTGGATCTAGTTGAAAATGTCAGTGACGCGTGATGAGTATGGCAACCAGGTTCGC

CAGACCGACGAGTATGGGAACCCGATTCAGCATACTGGCACCGGGACAAAGGCCGGTTCG

GGCATACATGGTGGGGGCCATGGGATTGGCACAGGTGGTGGTGGTGGTGGACAAGGCAAG

CTCCACCGTTCAGGCTCTGGCTCTTCCTCTGTAAGTACATTCGATGTTTCTCAAGGGTCA

TTTCACTTGGTATATCGTGTTGGGATGATTAGATTAGTTGATTAACGGTTGTGATTGTAA

ATCAGTGATATGGGCATCTGTGTACCTTAGTTCACGATGATAATCAATTGATACGCTGGA

TCGTGATGAGCGGACCGGATGAGCCGTCCATCTTACAGGCTTTTTAAAATAGAATTATAG

GGTGGGTGGTATGAGGTCCACCAAAGCCTGTAACATGGAATTACAATCATAGCCGTTAAT

CGGTTCATCTCATCCGCTCATCGTGATCTAGCATGTCAAACAGAGATGGGTTTCTGGAGA

AATGGATGTATGTAAGCCCACAAATCACAAAAATCTGACATTATGATGGAGAGAATGCAA

GGAGTGTAAGCGAATTTACGCCGTTAGATGGATTTCATTGTACTTTTAACTTTGTTTTGT

CGAATATTTTGTAGGATGAGGATGATGGACAAGGTGGGCGTAGGAAGAAGGGTTTGACGG

AGAAGATCAAAGAGAAGCTGCCAGGTGGAAACAAGACGACAGGTGTCTGTCATCCAGGGA

CTCAGGGTGTACAGGGGGGCCGCGAGCATGAGAAGACAGGTTGCGGTCAGGGTGGTCAGG

GTGGACAGGTGGGCCGCGAGCATGAGAAGACAGGTTTCGGCCATGCAGTGACTCAGGGTG

GACAGGTGGGCAACGAGCAGGAGAAGAAGGGTATGATTGAGAAGATCCAGGAGAAGCTGC

CAGGTCACAAGTAGGATGTGTGCCCACCATACACGTGGAGTCTACATCTACGTGCCTATT

ATGTTACTATAATACGCATGGAGTGTGTCCGGAGTCTGTAATAATCTGCGCGTATCTGTT

ATGTC

>BK66

AGCCGTTGGATCTAGTTGAAAATGTCAGTGACGCGTGATGAGTATGGCAACCAGGTTCGC

CAGACCGACGAGTATGGGAACCCGATTCAGCATACTGGCACCGGGACAAAGGCCGGTTCG

GGCATACATGGTGGGGGCCATGGGATTGGCACAGGTGGTGGTGGTGGTGGACAAGGCAAG

CTCCACCGTTCAGGCTCTGGCTCTTCCTCTGTAAGTACATTCGATGTTTCTCAAGGGTCA

TTTCACTTGGTATATCGTGTTGGGATGATTAGATTAGTTGATTAACGGTTGTGATTGTAA

ATCAGTGATATGGGCATCTGTGTACCTTAGTTCACGATGATAATCAATTGATACGCTGGA

TCGTGATGAGCGGACCGGATGAGCCGTCCATCTTACAGGCTTTTTAAAATAGAATTATAG

GGTGGGTGGTATGAGGTCCACCAAAGCCTGTAACATGGAATTACAATCATAGCCGTTAAT

CGGTTCATCTCATCCGCTCATCGTGATCTAGCATGTCAAACAGAGATGGGTTTCTGGAGA

AATGGATGTATGTAAGCCCACAAATCACAAAAATCTGACATTATGATGGAGAGAATGCAA

GGAGTGTAAGCGAATTTACGCCGTTAGATGGATTTCATTGTACTTTTAACTTTGTTTTGT

CGAATATTTTGTAGGATGAGGATGATGGACAAGGTGGGCGTAGGAAGAAGGGTTTGACGG

AGAAGATCAAAGAGAAGCTGCCAGGTGGAAACAAGACGACAGGTGTCTGTCATCCAGGGA

CTCAGGGTGTACAGGGGGGCCGCGAGCATGAGAAGACAGGTTGCGGTCAGGGTGGTCAGG

GTGGACAGGTGGGCCGCGAGCATGAGAAGACAGGTTTCGGCCATGCAGTGACTCAGGGTG

GACAGGTGGGCAACGAGCAGGAGAAGAAGGGTATGATTGAGAAGATCCAGGAGAAGCTGC

CAGGTCACAAGTAGGATGTGTGCCCACCATACACGTGGAGTCTACATCTACGTGCCTATT

ATGTTACTATAATACGCATGGAGTGTGTCCGGAGTCTGTAATAATCTGCGCGTATCTGTT

ATGTC

>BK82

AGCCGTTGGATCTAGTTGAAAATGTCTGAGACGCGTGATGAGTATGGCAACGAGGTTCGC

CAAACCGACGAGTATGGGAACCCGATTCAGCATACTGGCACCGGGACAAAGGGCGGTGCG

GGAATACATGGTGGGGGTCATGGGATTGGCACAGGTGGTGGTGGTGGACAAGGCAAGCTC

CACCGTTCAGGCTCCGGCTCTTCCTCTGTAAGTATATTAGATGTTTCTCAAGAGTCATTT

CACTTGGTAGATTGTGTTGGGATGACTAGATTAGTTGATTAACGGTTGCGATTGTAAATC

AGTGATATGGGCTTCTGTAGACCTTATTTCACGATGATAATCAATTGATACGGTGGATCG

TGATGAGCGGATCGGATGAGCCGTACATCTTACAGGCTTTTTAAAATAGAATTATAGGGT

GGGTGGTATGAGGTCCACCAAAGCCTGTAACATGGAATTACAATCATAGCCGTTAATCGG

TTCATCTCATCCGCTCATCGTGATCCAGCATGTCAAACGGAGATGGGTTACTGGCGAAAT

GGATGTATGTAAGCCCACAAATCACAAAAATCTGACATTATGATGGAGAGAATTGCAAGG

AGTGTAAGTGAATCTACGCCGTTAGATGGATACTTTTTAACTTCGTTTTGTCAAATATTC

TGTAGGATGAGGATGATGGACAAGGTGGGCGTAGGAAGAAGGGTTTGACGGAGAAGATCA

AAGAGAAGCTGCCAGGTGGGCAGAAAACGACAGGTGTCGGTTATCCAGGGAATCAGGGTG

TACAGGGGGGCCGCGAGCATGAGAAGACAGGTGTCTGTCATCCAGGGAATCAGGGTGTAC

AGGGGGGCCGCGAGCATGAGAAGACAGGTTTCGGTCATCCAGGGACTCAGGGTGGACACG

AGCATGAGAAGACAGGTGTCGGTCATCCAGGGACTCAGGGTGAGCAGGAGAAGAAGGGTA

TGATTGAGAAGATCAAGGAGAAGCTGCCAGGTCACAAGTAGGATGTGTGCCCACCATACA

CGTGGAGTCTACATCTATGTTTCTATGTTACTATAATACGCATGGAGTGTGTACGGATTC

TGTAATAATCTGCGCGTATCTGTTATGTC

>BK84

AGCCGTTGGATCTAGTTGAAAATGTCTGAGACGCGTGATGAGTATGGCAACGAGGTTCGC

CAAACCGACGAGTATGGGAACCCGATTCAGCATACTGGCACCGGGACAAAGGGCGGTGCG

GGAATACATGGTGGGGGTCATGGGATTGGCACAGGTGGTGGTGGTGGACAAGGCAAGCTC

CACCGTTCAGGCTCCGGCTCTTCCTCTGTAAGTATATTAGATGTTTCTCAAGAGTCATTT

CACTTGGTAGATTGTGTTGGGATGACTAGATTAGTTGATTAACGGTTGCGATTGTAAATC

AGTGATATGGGCTTCTGTAGACCTTATTTCACGATGATAATCAATTGATACGGTGGATCG

TGATGAGCGGATCGGATGAGCCGTACATCTTACAGGCTTTTTAAAATAGAATTATAGGGT

GGGTGGTATGAGGTCCACCAAAGCCTGTAACATGGAATTACAATCATAGCCGTTAATCGG

TTCATCTCATCCGCTCATCGTGATCCAGCATGTCAAACGGAGATGGGTTACTGGCGAAAT

GGATGTATGTAAGCCCACAAATCACAAAAATCTGACATTATGATGGAGAGAATTGCAAGG

AGTGTAAGTGAATCTACGCCGTTAGATGGATACTTTTTAACTTCGTTTTGTCAAATATTC

TGTAGGATGAGGATGATGGACAAGGTGGGCGTAGGAAGAAGGGTTTGACGGAGAAGATCA

AAGAGAAGCTGCCAGGTGGGCAGAAAACGACAGGTGTCGGTTATCCAGGGAATCAGGGTG

TACAGGGGGGCCGCGAGCATGAGAAGACAGGTGTCTGTCATCCAGGGAATCAGGGTGTAC

AGGGGGGCCGCGAGCATGAGAAGACAGGTTTCGGTCATCCAGGGACTCAGGGTGGACACG

AGCATGAGAAGACAGGTGTCGGTCATCCAGGGACTCAGGGTGAGCAGGAGAAGAAGGGTA

TGATTGAGAAGATCAAGGAGAAGCTGCCAGGTCACAAGTAGGATGTGTGCCCACCATACA

CGTGGAGTCTACATCTATGTTTCTATGTTACTATAATACGCATGGAGTGTGTACGGATTC

TGTAATAATCTGCGCGTATCTGTTATGTC

>BK85

AGCCGTTGGATCTAGTTGAAAATGTCTGAGACGCGTGATGAGTATGGCAACGAGGTTCGC

CAAACCGACGAGTATGGGAACCCGATTCAGCATACTGGCACCGGGACAAAGGGCGGTGCG

GGAATACATGGTGGGGGTCATGGGATTGGCACAGGTGGTGGTGGTGGACAAGGCAAGCTC

CACCGTTCAGGCTCCGGCTCTTCCTCTGTAAGTATATTAGATGTTTCTCAAGAGTCATTT

CACTTGGTAGATTGTGTTGGGATGACTAGATTAGTTGATTAACGGTTGCGATTGTAAATC

AGTGATATGGGCTTCTGTAGACCTTATTTCACGATGATAATCAATTGATACGGTGGATCG

TGATGAGCGGATCGGATGAGCCGTACATCTTACAGGCTTTTTAAAATAGAATTATAGGGT

GGGTGGTATGAGGTCCACCAAAGCCTGTAACATGGAATTACAATCATAGCCGTTAATCGG

TTCATCTCATCCGCTCATCGTGATCCAGCATGTCAAACGGAGATGGGTTACTGGCGAAAT

GGATGTATGTAAGCCCACAAATCACAAAAATCTGACATTATGATGGAGAGAATTGCAAGG

AGTGTAAGTGAATCTACGCCGTTAGATGGATACTTTTTAACTTCGTTTTGTCAAATATTC

TGTAGGATGAGGATGATGGACAAGGTGGGCGTAGGAAGAAGGGTTTGACGGAGAAGATCA

AAGAGAAGCTGCCAGGTGGGCAGAAAACGACAGGTGTCGGTTATCCAGGGAATCAGGGTG

TACAGGGGGGCCGCGAGCATGAGAAGACAGGTGTCTGTCATCCAGGGAATCAGGGTGTAC

AGGGGGGCCGCGAGCATGAGAAGACAGGTTTCGGTCATCCAGGGACTCAGGGTGGACACG

AGCATGAGAAGACAGGTGTCGGTCATCCAGGGACTCAGGGTGAGCAGGAGAAGAAGGGTA

TGATTGAGAAGATCAAGGAGAAGCTGCCAGGTCACAAGTAGGATGTGTGCCCACCATACA

CGTGGAGTCTACATCTATGTTTCTATGTTACTATAATACGCATGGAGTGTGTACGGATTC

TGTAATAATCTGCGCGTATCTGTTATGTC

>MSL114

AGCCGTTGGATCTAGTTGAAAATGTCAGTGACGCGTGATGAGTATGGCAACCAGGTTCGC

CAGACCGACGAGTATGGGAACCCGATTCAGCATACTGGCACCGGGACAAAGGCCGGTTTG

GGCATACATGGTGGGGGCCATGGGATTGGCACAGGTGGTGGTGGTGGTGGACAAGGCAAG

CTCCACCGTTCAGGCTCTGGCTCTTCCTCTGTAAGTACATTCGATGTTTCTCAAGGGTCA

TTTCACTTGGTATATCGTGTTGGGATGATTAGATTAGTTGATTAACGGTTGTGATTGTAA

ATCAGTGATATGGGCTTCTGTGTACCTTAGTTCACGATGATATTCAATTGATACGCTGGA

TCGTGATGAGCGGACCGGATGAGCCGTCCATCTTACAGGCTTTTTAAAATAGAATTATAG

GGTGGGTGGTATGAGGTCCACCAAAGCCTGTAACATGGAATTACAATCATAGCCGTTAAT

CGGTTCATCTCATCCGCTCATCGTGATCTAGCATGTCAAACAGAGATGGGTTTCTGGAGA

AATGGATGTATGTAAGCCCACAAATCACAAAAATCTGACATTATGATGGAGAGAATGCAA

GGAGTGTAAGCGAATTTACGCCGTTAGATGGATTTCATTGTACTTTTAACTTTGTTTTGT

CAAATATTTTGTAGGATGAGGATGATGGACAAGGTGGGCGTAGGAAGAAGGGTTTGATGG

AGAAGATCAAAGAGAAGCTGCCAGGTGGAAACAAGACGACAGGTGTCTGTCATCCAGGGA

CTCAGGGTGTACAGGGGGGCCGCGAGCATGAGAAGACAGGTTGCGGTCAGGGTGGACAGG

TGCGCCGCGAGCATGAGAAGACAGGTTTCGGCCACCCAGGGACTCAGGGTGGCCAGGTGG

GCAACGAGCAGGAGAAGAAGGGTATGATTGAGAAGATCCAGGAGAACCTGCCAGGTCACA

AGTAGGATGTGTGCCCACCATACACGTGGAGTCTACATCTACGTGTCTATTATGTTACTA

TAATACGCATGGAGTGTGTCCGGAGTCTGTAATAATCTGCGCGTATCTGTTATGTC

>MSL115

AGCCGTTGGATCTAGTTGAAAATGTCAGTGACGCGTGATGAGTATGGCAACCAGGTTCGC

CAGACCGACGAGTATGGGAACCCGATTCAGCATACTGGCACCGGGACAAAGGCCGGTTTG

GGCATACATGGTGGGGGCCATGGGATTGGCACAGGTGGTGGTGGTGGTGGACAAGGCAAG

CTCCACCGTTCAGGCTCTGGCTCTTCCTCTGTAAGTACATTCGATGTTTCTCAAGGGTCA

TTTCACTTGGTATATCGTGTTGGGATGATTAGATTAGTTGATTAACGGTTGTGATTGTAA

ATCAGTGATATGGGCTTCTGTGTACCTTAGTTCACGATGATATTCAATTGATACGCTGGA

TCGTGATGAGCGGACCGGATGAGCCGTCCATCTTACAGGCTTTTTAAAATAGAATTATAG

GGTGGGTGGTATGAGGTCCACCAAAGCCTGTAACATGGAATTACAATCATAGCCGTTAAT

CGGTTCATCTCATCCGCTCATCGTGATCTAGCATGTCAAACAGAGATGGGTTTCTGGAGA

AATGGATGTATGTAAGCCCACAAATCACAAAAATCTGACATTATGATGGAGAGAATGCAA

GGAGTGTAAGCGAATTTACGCCGTTAGATGGATTTCATTGTACTTTTAACTTTGTTTTGT

CAAATATTTTGTAGGATGAGGATGATGGACAAGGTGGGCGTAGGAAGAAGGGTTTGATGG

AGAAGATCAAAGAGAAGCTGCCAGGTGGAAACAAGACGACAGGTGTCTGTCATCCAGGGA

CTCAGGGTGTACAGGGGGGCCGCGAGCATGAGAAGACAGGTTGCGGTCAGGGTGGACAGG

TGCGCCGCGAGCATGAGAAGACAGGTTTCGGCCACCCAGGGACTCAGGGTGGCCAGGTGG

GCAACGAGCAGGAGAAGAAGGGTATGATTGAGAAGATCCAGGAGAACCTGCCAGGTCACA

AGTAGGATGTGTGCCCACCATACACGTGGAGTCTACATCTACGTGTCTATTATGTTACTA

TAATACGCATGGAGTGTGTCCGGAGTCTGTAATAATCTGCGCGTATCTGTTATGTC

>MSL121

AGCCGTTGGATCTAGTTGAAAATGTCTGAGACGCGTGATGAGTATGGCAACCAGGTTCGC

CAAACCGACGAGTATGGGAACCCGATTCAGCATACTGGCACCGGGACAAAGGGCGGTGCG

GGAATACATGGTGGGGGTCATGGGATTGGCACAGGTGGTGGTGGTGGACAAGGCAAGCTC

CACCGTTCAGGCTCCGGCTCTTCCTCTGTAAGTATATTAGATGTTTCTCAAGAGTCATTT

CACTTGGTAGATTGTGTTGGATGACTAGATTAGTTGATTAACGGTTGCGATTGTAAATCA

GTGATATGGGCTTCTGTCTACCTTATTTCATGATGATAATCAATTGATACGGTGGATCGT

GATGAGCGGATCGGATGAGCCGTACATCTTACAGGCTTTTTAAAATAGAATTATAGGGTG

GGTGGTATGAGGTCCACCAAAGCCTGTAACATGGAATTACAATCATAGCCGTTAATCGGT

TCATCTCATCCGCTCATCGTGATCCAGCATGTCAAACGGAGATGGGTTACTGGCGAAATG

GATGTATGTAAGCCCACAAATCACAAAAATCTGACATTATGATGGAGAGAATTGCAAGGA

GTGTAAGTGAATCTACGCCGTTAGATGGATACTTTTTAACTTCGTTTTGTCAAATATTCT

GTAGGATGAGGATGATGGACAAGGTGGGCGTAGGAAGAAGGGTTTGACGGAGAAGATCAA

AGAGAAGCTGCCAGGTGGGCAGAAGACGACAGGTGTCGGTTATCCAGGGAATCAGGGTGT

ACAGGGGGGCCGCGAGCATGAGAAGACAGGTGTCTGTCATCCAGGGACTCAGGGTGTACA

GGGGGGCCGCGAGCATGAGAAGACAGGTTTCGGTCATCCAGGGACTCAGGGTGGACACGA

GCATGAGAAGACAGGTGTCGGTCATCCAGGGACTCAGGGTGAGCAGGAGAAGAAGGGTAT

GATTGAGAAGATCAAGGAGAAGCTGCCAGGTCACAAGTAGGATGTGTGCCCACCATACAC

GTGGAGTCTACATCTATGTTTCTATGTTACTATAATACGCATGGAGTGTGTACGGATTCT

GTAATAATCTGCGCGTATCTGTTATGTC

>MSL21

AGCCGTTGGATCTAGTTGAAAATGTCTGAGACGCGTGATGAGTATGGCAACCAGGTTAGC

CAAACCGACGAGTATGGGAACCCGATTCAGCATACTGGCACCGGGACAAAGGGCGGTGCG

GGAATACATGGTGGGGGTCATGGGATTGGCACAGGTGGTGGTGGTGGACAAGGCAAGCTC

CACCGTTCAGGCTCCGGCTCTTCCTCTGTAAGTATATTAGATGTTTCTCAAGAGTCATTT

CACTTGGTAGATTGTGTTGGGATGACTAGATTAGTTGATTAACGGTTGCGATTGTAAATC

AGTGATATGGGCTTCTGTCTACCTTATTTCACGATGATAATCAATTGATACGGTGGATCG

TGATGAGCGGATCGGATGAGCCGTACATCTTACAGGCTTTTTAAAATAGAATTATAGGGT

GGGTGGTATGAGGTCCACCAAAGCCTGTAACATGGAATTACAATCATAGCCGTTAATCGG

TTCATCTCATCCGCTCATCGTGATCTAGCATGTAAAACAGAGATGGGTTTCTGGAGAAAT

GGATGCATGTAAGCCCACAAATCACAAAAATCTGACATTATGATGGAGAGAATGCAAGGA

GTGTAAGCGAATTTACGCCGTTAGATGGATTTCATTGTACTTTTAACTTTGTTTTGTCAA

ATATTTTGTAGGATGAGGATGATGGACAAGGTGGGCGTAGGAAGAAAGGTTTGACGGAGA

AGATCAAAGAGAAGCTGCCAGGTGGAAACAAGACGACAGGTGTCTGTCATCCAGGGACTC

AGGGTGTACAGGGGGGCCGCGAGCATGAGAAGACAGGTTGCGGTCAGGGTGGTCAGGGTG

GACAGGTGGGCCGCGAGCATGAGAAGACAGGTTTCGGCCATCCAGTGACTCAGGGTGGAC

AGGTGGGCAACGAGCAGGAGAAGAAGGGTATGATCGAGAAGATCAAGGAGAAGCTGCCAG

GTCACAAGTAGGATGTGTGCCCACCATACACGTGGAGTCTACATCTATGTTTCTATGTTA

CTATAATACGCATGGAGTGTGTACGGATTCTGTAATAATCTGCGCGTATCTGTTATGTC

>MSL29

AGCCGTTGGATCTAGTTGAAAATGTCAGTGACGCGTGATGAGTATGGCAACCAGGTTCGG

CAGACCGACGAGTATGGGAACCCGATTCAGCATACTGGCACCGGGACAAAGGCCGGTTCG

GGCATACATGGTGGGGGCCATGGGATTGGCACAGGTGGTGGTGGTGGTGGACAAGGCAAG

CTCCACCGTTCAGGCTCTGGCTCTTCCTCTGTAAGTACATTCGATGTTTCTCAAGGGTCA

TTTCACTTGGTATATCGTGTTGGGATGATTAGATTAGTTGATTAACGGTTGTGATTGTAA

ATCAGTGATATGGGCTTCTGTGTACCTTAGTTCACGATGATAATCAATTGATACGCTGGA

TCGTGATGAGCGGACCGGATGAGCCGTCCATCTTACAGGCTTTTTAAAATAGAATTATAG

GGTGGGTGGTATGAGGTCCACCAAAGCCTGTAACATGGAATTACAATCATAGCCGTTAAT

CGGTTCATCTCATCCGCTCATCGTGATCTAGCATGTAAAACAGAGATGGGTTTCTGGAGA

AATGGATGCATGTAAGCCCACAAATCACAAAAATCTGACATTATGATGGAGAGAATGCAA

GGAGTGTAAGCGAATTTACGCCGTTAGATGGATTTCATTGTACTTTTAACTTTGTTTTGT

CAAATATTTTGTAGGATGAGGATGATGGACAAGGTGGGCGTAGGAAGAAAGGTTTGACGG

AGAAGATCAAAGAGAAGCTGCCAGGTGGAAACAAGACGACAGGTGTCTGTCATCCAGGGA

CTCAGGGTGTACAGGGGGGCCGCGAGCATGAGAAGACAGGTTGCGGTCAGGGTGGTCAGG

GTGGACAGGTGGGCCGCGAGCATGAGAAGACAGGTTTCGGCCATCCAGTGACTCAGGGTG

GACAGGTGGGCAACGAGCAGGAGAAGAAGGGTATGATTGAGAAGATCCAGGAGAAGCTGC

CAGGTCACAAGTAGGATGTGTGCCCACCATACACGTGGAGTCTACATCTACGTGTCTATT

ATGTTACTATAATACGCATGGAATGTGTCCGGAGTCTGTAATAATCTGCGCGTATCTGTT

ATGTC

>MSL210

AGCCGTTGGATCTAGTTGAAAATGTCAGTGACGCGTGATGAGTATGGCAACCAGGTTCGC

CAGACCGACGAGTATGGGAACCCGATTCAGCATACTGGCACCGGGACAAAGGCCGGTTTG

GGCATACATGGTGGGGGCCATGGGATTGGCACAGGTGGTGGTGGTGGTGGACAAGGCAAG

CTCCACCGTTCAGGCTCTGGCTCTTCCTCTGTAAGTACATTCGATGTTTCTCAAGGGTCA

TTTCACTTGGTATATCGTGTTGGGATGATTAGATTAGTTGATTAACGGTTGTGATTGTAA

ATCAGTGATATGGGCTTCTGTGTACCTTAGTTCACGATGATATTCAATTGATACGCTGGA

TCGTGATGAGCGGACCGGATGAGCCGTCCATCTTACAGGCTTTTTAAAATAGAATTATAG

GGTGGGTGGTATGAGGTCCACCAAAGCCTGTAACATGGAATTACAATCATAGCCGTTAAT

CGGTTCATCTCATCCGCTCATCGTGATCTAGCATGTCAAACAGAGATGGGTTTCTGGAGA

AATGGATGTATGTAAGCCCACAAATCACAAAAATCTGACATTATGATGGAGAGAATGCAA

GGAGTGTAAGCGAATTTACGCCGTTAGATGGATTTCATTGTACTTTTAACTTTGTTTTGT

CAAATATTTTGTAGGATGAGGATGATGGACAAGGTGGGCGTAGGAAGAAGGGTTTGATGG

AGAAGATCAAAGAGAAGCTGCCAGGTGGAAACAAGACGACAGGTGTCTGTCATCCAGGGA

CTCAGGGTGTACAGGGGGGCCGCGAGCATGAGAAGACAGGTTGCGGTCAGGGTGGACAGG

TGCGCCGCGAGCATGAGAAGACAGGTTTCGGCCACCCAGGGACTCAGGGTGGCCAGGTGG

GCAACGAGCAGGAGAAGAAGGGTATGATTGAGAAGATCCAGGAGAACCTGCCAGGTCACA

AGTAGGATGTGTGCCCACCATACACGTGGAGTCTACATCTACGTGTCTATTATGTTACTA

TAATACGCATGGAGTGTGTCCGGAGTCTGTAATAATCTGCGCGTATCTGTTATGTC

>MSL223

AGCCGTTGGATCTAGTTGAAAATGTCAGTGACGCGTGATGAGTATGGCAACCAGGTTCGC

CAGACCGACGAGTATGGGAACCCGATTCAGCATACTGGCACCGGGACAAAGGCCGGTTTG

GGCATACATGGTGGGGGCCATGGGATTGGCACAGGTGGTGGTGGTGGTGGACAAGGCAAG

CTCCACCGTTCAGGCTCTGGCTCTTCCTCTGTAAGTACATTCGATGTTTCTCAAGGGTCA

TTTCACTTGGTATATCGTGTTGGGATGATTAGATTAGTTGATTAACGGTTGTGATTGTAA

ATCAGTGATATGGGCTTCTGTGTACCTTAGTTCACGATGATATTCAATTGATACGCTGGA

TCGTGATGAGCGGACCGGATGAGCCGTCCATCTTACAGGCTTTTTAAAATAGAATTATAG

GGTGGGTGGTATGAGGTCCACCAAAGCCTGTAACATGGAATTACAATCATAGCCGTTAAT

CGGTTCATCTCATCCGCTCATCGTGATCTAGCATGTCAAACAGAGATGGGTTTCTGGAGA

AATGGATGTATGTAAGCCCACAAATCACAAAAATCTGACATTATGATGGAGAGAATGCAA

GGAGTGTAAGCGAATTTACGCCGTTAGATGGATTTCATTGTACTTTTAACTTTGTTTTGT

CAAATATTTTGTAGGATGAGGATGATGGACAAGGTGGGCGTAGGAAGAAAGGTTTGACGG

AGAAGATCAAAGAGAAGCTGCCAGGTGGAAACAAGACGACAGGTGTCTGTCATCCAGGGA

CTCAGGGTGTACAGGGGGGCCGCGAGCATGAGAAGACAGGTTGCGGTCAGGGTGGTCAGG

GTGGACAGGTGGGCCGCGAGCATGAGAAGACAGGTTTCGGCCATCCAGTGACTCAGGGTG

GACAGGTGGGCAACGAGCAGGAGAAGAAGGGTATGATTGAGAAGATCCAGGAGAAGCTGC

CAGGTCACAAGTAGGATGTGTGCCCACCATACACGTGGAGTCTACATCTACGTGTCTATT

ATGTTACTATAATACGCATGGAATGTGTCCGGAGTCTGTAATAATCTGCGCGTATCTGTT

ATGTC

>MSL37

AGCCGTTGGATCTAGTTGAAAATGTCAGTGACGCGTGATGAGTATGGCAACCAGGTTCGC

CAGACCGACGAGTATGGGAACCCGATTCAGCATACTGGCACCGGGACAAAGGCCGGTTTG

GGCATACATGGTGGGGGCCATGGGATTGGCACAGGTGGTGGTGGTGGTGGACAAGGCAAG

CTCCACCGTTCAGGCTCTGGCTCTTCCTCTGTAAGTACATTCGATGTTTCTCAAGGGTCA

TTTCACTTGGTATATCGTGTTGGGATGATTAGATTAGTTGATTAACGGTTGTGATTGTAA

ATCAGTGATATGGGCTTCTGTGTACCTTAGTTCACGATGATATTCAATTGATACGCTGGA

TCGTGATGAGCGGACCGGATGAGCCGTCCATCTTACAGGCTTTTTAAAATAGAATTATAG

GGTGGGTGGTATGAGGTCCACCAAAGCCTGTAACATGGAATTACAATCATAGCCGTTAAT

CGGTTCATCTCATCCGCTCATCGTGATCTAGCATGTCAAACAGAGATGGGTTTCTGGAGA

AATGGATGTATGTAAGCCCACAAATCACAAAAATCTGACATTATGATGGAGAGAATGCAA

GGAGTGTAAGCGAATTTACGCCGTTAGATGGATTTCATTGTACTTTTAACTTTGTTTTGT

CAAATATTTTGTAGGATGAGGATGATGGACAAGGTGGGCGTAGGAAGAAGGGTTTGATGG

AGAAGATCAAAGAGAAGCTGCCAGGTGGAAACAAGACGACAGGTGTCTGTCATCCAGGGA

CTCAGGGTGTACAGGGGGGCCGCGAGCATGAGAAGACAGGTTGCGGTCAGGGTGGACAGG

TGCGCCGCGAGCATGAGAAGACAGGTTTCGGCCACCCAGGGACTCAGGGTGGCCAGGTGG

GCAACGAGCAGGAGAAGAAGGGTATGATTGAGAAGATCCAGGAGAACCTGCCAGGTCACA

AGTAGGATGTGTGCCCACCATACACGTGGAGTCTACATCTACGTGTCTATTATGTTACTA

TAATACGCATGGAGTGTGTCCGGAGTCTGTAATAATCTGCGCGTATCTGTTATGTC

>MSL315

AGCCGTTGGATCTAGTTGAAAATGTCAGTGACGCGTGATGAGTATGGCAACCAGGTTCGC

CAGACCGACGAGTATGGGAACCCGATTCAGCATACTGGCACCGGGACAAAGGCCGGTTTG

GGCATACATGGTGGGGGCCATGGGATTGGCACAGGTGGTGGTGGTGGTGGACAAGGCAAG

CTCCACCGTTCAGGCTCTGGCTCTTCCTCTGTAAGTACATTCGATGTTTCTCAAGGGTCA

TTTCACTTGGTATATCGTGTTGGGATGATTAGATTAGTTGATTAACGGTTGTGATTGTAA

ATCAGTGATATGGGCTTCTGTGTACCTTAGTTCACGATGATATTCAATTGATACGCTGGA

TCGTGATGAGCGGACCGGATGAGCCGTCCATCTTACAGGCTTTTTAAAATAGAATTATAG

GGTGGGTGGTATGAGGTCCACCAAAGCCTGTAACATGGAATTACAATCATAGCCGTTAAT

CGGTTCATCTCATCCGCTCATCGTGATCTAGCATGTCAAACAGAGATGGGTTTCTGGAGA

AATGGATGTATGTAAGCCCACAAATCACAAAAATCTGACATTATGATGGAGAGAATGCAA

GGAGTGTAAGCGAATTTACGCCGTTAGATGGATTTCATTGTACTTTTAACTTTGTTTTGT

CAAATATTTTGTAGGATGAGGATGATGGACAAGGTGGGCGTAGGAAGAAGGGTTTGATGG

AGAAGATCAAAGAGAAGCTGCCAGGTGGAAACAAGACGACAGGTGTCTGTCATCCAGGGA

CTCAGGGTGTACAGGGGGGCCGCGAGCATGAGAAGACAGGTTGCGGTCAGGGTGGACAGG

TGCGCCGCGAGCATGAGAAGACAGGTTTCGGCCACCCAGGGACTCAGGGTGGCCAGGTGG

GCAACGAGCAGGAGAAGAAGGATATGATTGAGAAGATCCAGGAGAACCTGCCAGGTCACA

AGTAGGATGTGTGCCCACCATACACGTGGAGTCTACATCTACGTGTCTATTATGTTACTA

TAATACGCATGGAGTGTGTCCGGAGTCTGTAATAATCTGCGCGTATCTGTTATGTC

>MSL316

AGCCGTTGGATCTAGTTGAAAATGTCTGAGACGCGTGATGAGTATGGCAACCAGGTTAGC

CAAACCGACGAGTATGGGAACCCGATTCAGCATACTGGCACCGGGACAAAGGGCGGTGCG

GGAATACATGGTGGGGGTCATGGGATTGGCACAGGTGGTGGTGGTGGACAAGGCAAGCTC

CACCGTTCAGGCTCCGGCTCTTCCTCTGTAAGTATATTAGATGTTTCTCAAGAGTCATTT

CACTTGGTAGATTGTGTTGGGATGACTAGATTAGTTGATTAACGGTTGCGATTGTAAATC

AGTGATATGGGCTTCTGTCTACCTTATTTCACGATGATAATCAATTGATACGGTGGATCG

TGATGAGCGGATCGGATGAGCCGTACATCTTACAGGCTTTTTAAAATAGAATTATAGGGT

TGGTGGTATGAGGTCCACCAAAGCCTGTAACATGGAATTACAATCATAGCCGTTAATCGG

TTCATCTCATCCGCTCATCGTGATCCAGCATGTCAAACGGAGATGGGTTACTGGCGAAAT

GGATGTATGTAAGCCCACAAATCACAAAAATCTGACATTATGATGGAGAGAATTGCAAGG

AGTGTAAGTGAATCTACGCCGTTAGATGGATACTTTTTAACTTCGTTTTGTCAAATATTC

TGTAGGATGAGGATGATGGACAAGGTGGGCGTAGGAAGAAGGGTTTGACGGAGAAGATCA

AAGAGAAGCTGCCAGGTGGGCAGAAGACGACAGGTGTCGGTTATCCAGGGAATCAGGGTG

TACAGGGGGGCCGCGAGCATGAGAAGACAGGTGTCTGTCATCCAGGGACTCAGGGTGTAC

AGGGGGGCCGCGAGCATGAGAAGACCGGTTTCGGTCATCCAGGGACTCAGGGTGGACACG

AGCATGAGAAGACAGGTGTCGGTCATCCAGGGACTCAGGGTGAGCAGGAGAAGAAGGGTA

TGATTGAGAAGATCAAGGAGAAGCTGCCAGGTCACAAGTAGGATGTGTGCCCACCATACA

CGTGGAGTCTACATCTATGTTTCTATGTTACTATAATACGCATGGAGTGTGTACGGATTC

TGTAATAATCTGCGCGTATCTGTTATGTC

>MSL43

AGCCGTTGGATCTAGTTGAAAATGTCAGTGACGCGTGATGAGTATGGCAACCAGGTTCGG

CAGACCGACGAGTATGGGAACCCGATTCAGCATACTGGCACCGGGACAAAGGGCGGTGCG

GGAATACATGGTGGGGGTCATGGGATTGGCACAGGTGGTGGTGGTGGACAAGGCAAGCTC

CACCGTTCAGGCTCCGGCTCTTCCTCTGTAAGTATATTAGATGTTTCTCAAGAGTCATTT

CACTTGGTAGATTGTGTTGGGATGACTAGATTAGTTGATTAACGGTTGCGATTGTAAATC

AGTGATATGGGCTTCTGTCTACCTTATTTCACGATGATAATCAATTGATACGGTGGATCG

TGATGAGCGGATCGGATGAGCCGTACATCTACAGGCTTTTTAAAATAGAATTATAGGGTG

GGTGGTATGAGGTCCACCAAAGCCTGTAACATGGAATTACAATCATAGCCGTTAATCGGT

TCATCTCATCCGCTCATCGTGATCCAGCATGTCAAACAGAGATGGGTTTCTGGCGAAATG

GATGTATGTAAGCCCACAAATCACAAAAATCTGATATTATGATGGAGAGAATTGCAAGGA

GTGTAAGTGAATCTACGCCGTTAGATGGATACTTTTTAACTTCGTTTTGTCAAATATTCT

GTAGGATGAGGATGATGGACAAGGTGGGCGTAGGAAGAAGGGTTTGACGGAGAAGATCAA

AGAGAAGCTGCCAGGTGGGCAGAAGACGACAGGTGTCGGTTATCCAGGGAATCAGGGTGT

ACAGGGGGGCCGCGAGCATGAGAAGACAGGTGTCTGTCATCCAGGGACTCAGGGTGTACA

GGGGGGCCGCGAGCATGAGAAGACCGGTTTCGGTCATCCAGGGACTCAGGGTGGACACGA

GCATGAGAAGACAGGTGTCGGTCATCCAGGGACTCAGGGTGAGCAGGAGAAGAAGGGTAT

GATTGAGAAGATCAAGGAGAAGCTGCCAGGTCACAAGTAGGATGTGTGCCCACCATACAC

GTGGAGTCTACATCTATGTTTCTATGTTACTATAATACGCATGGAGTGTGTACGGATTCT

GTAATAATCTGCGCGTATCTGTTATGTC

>MSL45

AGCCGTTGGATCTAGTTGAAAATGTCAGTGACGCGTGATGAGTATGGCAACCAGGTTCGG

CAGACCGACGAGTATGGGAACCCGATTCAGCATACTGGCACCGGGACAAAGGCCGGTTCG

GGCATACATGGTGGGGGCCATGGGATTGGCACAGGTGGTGGTGGTGGTGGACAAGGCAAG

CTCCACCGTTCAGGCTCTGGCTCTTCCTCTGTAAGTACATTCGATGTTTCTCAAGGGTCA

TTTCACTTGGTATATCGTGTTGGGATGATTAGATTAGTTGATTAACGGTTGTGATTGTAA

ATCAGTGATATGGGCTTCTGTGTACCTTAGTTCACGATGATAATCAATTGATACGCTGGA

TCGTGATGAGCGGACCGGATGAGCCGTCCATCTTACAGGCTTTTTAAAATAGAATTATAG

GGTGGGTGGTATGAGGTCCACCAAAGCCTGTAACATGGAATTACAATCATAGCCGTTAAT

CGGTTCATCTCATCCGCTCATCGTGATCTAGCATGTAAAACAGAGATGGGTTTCTGGAGA

AATGGATGCATGTAAGCCCACAAATCACAAAAATCTGACATTATGATGGAGAGAATTGCA

AGGAGTGTAAGTGAATCTACGCCGTTAGATGGATACTTTTTAACTTCGTTTTGTCAAATA

TTCTGTAGGATGAGGATGATGGACAAGGTGGGCGTAGGAAGAAGGGTTTGACGGAGAAGA

TCAAAGAGAAGCTGCCAGGTGGGCAGAAGACGACGACAGGTGTGGGTTATCCAGGGAATC

AGGGTGTACAGGGGGGCCGCGAGCATGAGAAGACAGGTGTCTGTCATCCAGGGACTCAGG

GTGTACAGGGGGGCCGCGAGCATGAGAAGACAGGTTTCGGTCATCCAGGGACTCAGGGTG

GACACGAGCATGAGAAGACAGGTGTCGGTCATCCAGGGACTCAGGGTGAGCAGGAGAAGA

AGGGTATGATTGAGAAGATCAAGGAGAAGCTGCCAGGTCACAAGTAGGATGTGTGCCCAC

CATACACGTGGAGTCTACATCTATGTTTCTATGTTACTATAATACGCATGGAGTGTGTAC

GGATTCTGTAATAATCTGCGCGTATCTGTTATGTC

>MSL48

AGCCGTTGGATCTAGTTGAAAATGTCTGAGACGCGTGATGAGTATGGCAACCAGGTTCGC

CAAACCGACGAGTATGGGAACCCGATTCAGCATACTGGCACCGGGACAAAGGGCGGTGCG

GGAATACATGGTGGGGGTCATGGGATTGGCACAGGTGGTGGTGGTGGACAAGGCAAGCTC

CACCGTTCAGGCTCCGGCTCTTCCTCTGTAAGTATATTAGATGTTTCTCAAGAGTCATTT

CACTTGGTAGATTGTGTTGGGATGACTAGATTAGTTGATTAACGGTTGCGATTGTAAATC

AGTGATATGGGCTTCTGTAGACCTTATTTCACGATGATAATCAATTGATACGGTGGATCG

TGATGAGCGGATCGGATGAGCCGTACATCTTACAGGCTTTTTAAAATAGAATTATAGGGT

GGGTGGTATGAGGTCCACCAAAGCCTGTAACATGGAATTACAATCATAGCCGTTAATCGG

TTCATCTCATCCGCTCATCGTGATCTAGCATGTAAAACAGAGATGGGTTTCTGGAGAAAT

GGATGCATGTAAGCCCACAAATCACAAAAATCTGACATTATGATGGAGAGAATGCAAGGA

GTGTAAGCGAATTTACGCCGTTAGATGGATTTCATTGTACTTTTAACTTTGTTTTGTCAA

ATATTTTGTAGGATGAGGATGATGGACAAGGTGGGCGTAGGAAGAAAGGTTTGACGGAGA

AGATCAAAGAGAAGCTGCCAGGTGGAAACAAGACGACAGGTGTCTGTCATCCAGGGACTC

AGGGTGTACAGGGGGGCCGCGAGCATGAGAAGACAGGTTGCGGTCAGGGTGGTCAGGGTG

GACAGGTGGGCCGCGAGCATGAGAAGACAGGTTTCGGCCATCCAGTGACTCAGGGTGGAC

AGGTGGGCAACGAGCAGGAGAAGAAGGGTATGATTGAGAAGATCCAGGAGAAGCTGCCAG

GTCACAAGTAGGATGTGTGCCCACCATACACGTGGAGTCTACATCTACGTGTCTATTATG

TTACTATAATACGCATGGAATGTGTCCGGAGTCTGTAATAATCTGCGCGTATCTGTTATG

TC

>MSL53

AGCCGTTGGATCTAGTTGAAAATGTCTGAGACGCGTGATGAGTATGGCAACCAGGTTCGC

CAAACCGACGAGTATGGGAACCCGATTCAGCATACTGGCACCGGGACAAAGGCCGGTTTG

GGCATACATGGTGGGGGCCATGGGATTGGCACAGGTGGTGGTGGTGGTGGACAAGGCAAG

CTCCACCGTTCAGGCTCTGGCTCTTCCTCTGTAAGTACATTCGATGTTTCTCAAGGGTCA

TTTCACTTGGTATATCGTGTTGGGATGATTAGATTAGTTGATTAACGGTTGTGATTGTAA

ATCAGTGATATGGGCTTCTGTGTACCTTAGTTCACGATGATAATCAATTGATACGCTGGA

TCGTGATGAGCGGACCGGATGAGCCGTCCATCTTACAGGCTTTTTAAAATAGAATTATAG

GGTGGGTGGTATGAGGTCCACCAAAGCCTGTAACATGGAATTACAATCATAGCCGTTAAT

CGGTTCATCTCATCCGCTCATCGTGATCCAGCATGTGAAACAGAGATGGGTTTCTGGCGA

AATGGATGTATGTAAGCCCACAAATCACAAAAATCTGACATTATGATGGAGAGAATTGCA

AGGAGTGTAAGTGGATCTACGCCGTTGGATGGATACTTTTTAACTTCGTTTTGTCAAATA

TTCTGTAGGATGAGGATGATGGACAAGGTGGGCGTAGGAAGAAGGGTTTGACGGAGAAGA

TCAAAGAGAAGCTGCCAGGTGGGCAGAAGACGACAGGTGTCTGTCATCCAGGGACTCAGG

GTGTACAGGGGGGCCGCGAGCATGAGAAGACAGGTTGCGGTCAGGGTGGTCAGGGTGGAC

AGGTGGGCCGCGAGCATGAGAAGACAGGTTTCGGCCATCCAGTGACTCAGGGTGGACAGG

TGGGCAACGAGCAGGAGAAGAAGGGTATGATTGAGAAGATCCAGGAGAAGCTGCCAGGTC

ACAAGTAGGATGTGTGCCCACCATACACGTGGAGTCTACATCTACGTGTCTATTATGTTA

CTATAATACGCATGGAATGTGTCCGGAGTCTGTAATAATCTGCGCGTATCTGTTATGTC

>MSL56

AGCCGTTGGATCTAGTTGAAAATGTCAGTGACGCGTGATGAGTATGGCAACCAGGTTCGG

CAGACCGACGAGTATGGGAACCCGATTCAGCATACTGGCACCGGGACAAAGGCCGGTTCG

GGCATACATGGTGGGGGCCATGGGATTGGCACAGGTGGTGGTGGTGGTGGACAAGGCAAG

CTCCACCGTTCAGGCTCTGGCTCTTCCTCTGTAAGTACATTCGATGTTTCTCAAGGGTCA

TTTCACTTGGTATATCGTGTTGGGATGATTAGATTAGTTGATTAACGGTTGTGATTGTAA

ATCAGTGATATGGGCTTCTGTGTACCTTAGTTCACGATGATAATCAATTGATACGCTGGA

TCGTGATGAGCGGACCGGATGAGCCGTCCATCTTACAGGCTTTTTAAAATAGAATTATAG

GGTGGGTGGTATGAGGTCCACCAAAGCCTGTAACATGGAATTACAATCATAGCCGTTAAT

CGGTTCATCTCATCCGCTCATCGTGATCTAGCATGTAAAACAGAGATGGGTTTCTGGAGA

AATGGATGCATGTAAGCCCACAAATCACAAAAATCTGACATTATGATGGAGAGAATGCAA

GGAGTGTAAGCGAATTTACGCCGTTAGATGGATTTCATTGTACTTTTAACTTTGTTTTGT

CAAATATTTTGTAGGATGAGGATGATGGACAAGGTGGGCGTAGGAAGAAAGGTTTGACGG

AGAAGATCAAAGAGAAGCTGCCAGGTGGAAACAAGACGACAGGTGTCTGTCATCCAGGGA

CTCAGGGTGTACAGGGGGGCCGCGAGCATGAGAAGACAGGTTGCGGTCAGGGTGGTCAGG

GTGGACAGGTGGGCCGCGAGCATGAGAAGACAGGTTTCGGCCATCCAGTGACTCAGGGTG

GACAGGTGGGCAACGAGCAGGAGAAGAAGGGTATGATTGAGAAGATCCAGGAGAAGCTGC

CAGGTCACAAGTAGGATGTGTGCCCACCATACACGTGGAGTCTACATCTACGTGTCTATT

ATGTTACTATAATACGCATGGAATGTGTCCGGAGTCTGTAATAATCTGCGCGTATCTGTT

ATGTC

>MSL58

AGCCGTTGGATCTAGTTGAAAATGTCAGTGACGCGTGATGAGTATGGCAACCAGGTTCGG

CAGACCGACGAGTATGGGAACCCGATTCAGCATACTGGCACCGGGACAAAGGCCGGTTCG

GGCATACATGGTGGGGGCCATGGGATTGGCACAGGTGGTGGTGGTGGTGGACAAGGCAAG

CTCCACCGTTCAGGCTCTGGCTCTTCCTCTGTAAGTACATTCGATGTTTCTCAAGGGTCA

TTTCACTTGGTATATCGTGTTGGGATGATTAGATTAGTTGATTAACGGTTGTGATTGTAA

ATCAGTGATATGGGCTTCTGTGTACCTTAGTTCACGATGATAATCAATTGATACGCTGGA

TCGTGATGAGCGGACCGGATGAGCCGTCCATCTTACAGGCTTTTTAAAATAGAATTATAG

GGTGGGTGGTATGAGGTCCACCAAAGCCTGTAACATGGAATTACAATCATAGCCGTTAAT

CGGTTCATCTCATCCGCTCATCGTGATCTAGCATGTAAAACAGAGATGGGTTTCTGGAGA

AATGGATGCATGTAAGCCCACAAATCACAAAAATCTGACATTATGATGGAGAGAATGCAA

GGAGTGTAAGCGAATTTACGCCGTTAGATGGATTTCATTGTACTTTTAACTTTGTTTTGT

CAAATATTTTGTAGGATGAGGATGATGGACAAGGTGGGCGTAGGAAGAAAGGTTTGACGG

AGAAGATCAAAGAGAAGCTGCCAGGTGGAAACAAGACGACAGGTGTCTGTCATCCAGGGA

CTCAGGGTGTACAGGGGGGCCGCGAGCATGAGAAGACAGGTTGCGGTCAGGGTGGTCAGG

GTGGACAGGTGGGCCGCGAGCATGAGAAGACAGGTTTCGGCCATCCAGTGACTCAGGGTG

GACAGGTGGGCAACGAGCAGGAGAAGAAGGGTATGATTGAGAAGATCCAGGAGAAGCTGC

CAGGTCACAAGTAGGATGTGTGCCCACCATACACGTGGAGTCTACATCTACGTGTCTATT

ATGTTACTATAATACGCATGGAATGTGTCCGGAGTCTGTAATAATCTGCGCGTATCTGTT

ATGTC

>MSL510

AGCCGTTGGATCTAGTTGAAAATGTCTGAGACGCGTGATGAGTATGGCAACCAGGTTAGC

CAAACCGACGAGTATGGGAACCCGATTCAGCATACTGGCACCGGGACAAAGGGCGGTGCG

GGAATACATGGTGGGGGTCATGGGATTGGCACAGGTGGTGGTGGTGGACAAGGCAAGCTC

CACCGTTCAGGCTCTGGCTCTTCCTCTGTAAGTACATTCGATGTTTCTCAAGGGTCATTT

CACTTGGTATATCGTGTTGGGATGATTAGATTAGTTGATTAACGGTTGTGATTGTAAATC

AGTGATATGGGCTTCTGTGTACCTTAGTTCACGATGATATTCAATTGATACGCTGGATCG

TGATGAGCGGACCGGATGAGCCGTCCATCTTACAGGCTTTTTAAAATAGAATTATAGGGT

GGGTGGTATGAGGTCCACCAAAGCCTGTAACATGGAATTACAATCATAGCCGTTAATCGG

TTCATCTCATCCGCTCATCGTGATCTAGCATGTAAAACAGAGATGGGTTTCTGGAGAAAT

GGATGCATGTAAGCCCACAAATCACAAAAATCTGACATTATGATGGAGAGAATGCAAGGA

GTGTAAGCGAATTTACGCCGTTAGATGGATTTCATTGTACTTTTAACTTTGTTTTGTCAA

ATATTTTGTAGGATGAGGATGATGGACAAGGTGGGCGTAGGAAGAAAGGTTTGACGGAGA

AGATCAAAGAGAAGCTGCCAGGTGGAAACAAGACGACAGGTGTCTGTCATCCAGGGACTC

AGGGTGTACAGGGGGGCCGCGAGCATGAGAAGACAGGTTGCGGTCAGGGTGGTCAGGGTG

GACAGGTGGGCCGCGAGCATGAGAAGACAGGTTTCGGCCATCCAGTGACTCAGGGTGGAC

AGGTGGGCAACGAGCAGGAGAAGAAGGGTATGATTGAGAAGATCCAGGAGAAGCTGCCAG

GTCACAAGTAGGATGTGTGCCCACCATACACGTGGAGTCTACATCTACGTGTCTATTATG

TTACTATAATACGCATGGAATGTGTCCGGAGTCTGTAATAATCTGCGCGTATCTGTTATG

TC

>MSL73

AGCCGTTGGATCTAGTTGAAAATGTCAGTGACGCGTGATGAGTATGGCAACCAGGTTCGC

CAGACCGACGAGTATGGGAACCCGATTCAGCATACTGGCACCGGGACAAAGGCCGGTTTG

GGCATACATGGTGGGGGCCATGGGATTGGCACAGGTGGTGGTGGTGGTGGACAAGGCAAG

CTCCACCGTTCAGGCTCTGGCTCTTCCTCTGTAAGTACATTCGATGTTTCTCAAGGGTCA

TTTCACTTGGTATATCGTGTTGGGATGATTAGATTAGTTGATTAACGGTTGTGATTGTAA

ATCAGTGATATGGGCTTCTGTGTACCTTAGTTCACGATGATATTCAATTGATACGCTGGA

TCGTGATGAGCGGACCGGATGAGCCGTCCATCTTACAGGCTTTTTAAAATAGAATTATAG

GGTGGGTGGTATGAGGTCCACCAAAGCCTGTAACATGGAATTACAATCATAGCCGTTAAT

CGGTTCATCTCATCCGCTCATCGTGATCTAGCATGTCAAACAGAGATGGGTTTCTGGAGA

AATGGATGTATGTAAGCCCACAAATCACAAAAATCTGACATTATGATGGAGAGAATGCAA

GGAGTGTAAGCGAATTTACGCCGTTAGATGGATTTCATTGTACTTTTAACTTTGTTTTGT

CAAATATTTTGTAGGATGAGGATGATGGACAAGGTGGGCGTAGGAAGAAGGGTTTGATGG

AGAAGATCAAAGAGAAGCTGCCAGGTGGAAACAAGACGACAGGTGTCTGTCATCCAGGGA

CTCAGGGTGTACAGGGGGGCCGCGAGCATGAGAAGACAGGTTGCGGTCAGGGTGGACAGG

TGCGCCGCGAGCATGAGAAGACAGGTTTCGGCCATCCAGGGACTCAGGGTGGCCAGGTGG

GCAACGAGCAGGAGAAGAAGGGTATGATTGAGAAGATCCAGGAGAACCTGCCAGGTCACA

AGTAGGATGTGTGCCCACCATACACGTGGAGTCTACATCTACGTGTCTATTATGTTACTA

TAATACGCATGGAGTGTGTCCGGAGTCTGTAATAATCTGCGCGTATCTGTTATGTC

>MSL77

AGCCGTTGGATCTAGTTGAAAATGTCTGAGACGCGTGATGAGTATGGCAACCAGGTTAGC

CAAACCGACGAGTATGGGAACCCGATTCAGCATACTGGCACCGGGACAAAGGGCGGTGCG

GGAATACATGGTGGGGGTCATGGGATTGGCACAGGTGGTGGTGGTGGACAAGGCAAGCTC

CACCGTTCAGGCTCCGGCTCTTCCTCTGTAAGTATATTAGATGTTTCTCAAGAGTCATTT

CACTTGGTAGATTGTGTTGGGATGACTAGATTAGTTGATTAACGGTTGCGATTGTAAATC

AGTGATATGGGCTTCTGTCTACCTTATTTCACGATGATAATCAATTGATACGGTGGATCG

TGATGAGCGGATCGGATGAGCCGTACATCTTACAGGCTTTTTAAAATAGAATTATAGGGT

TGGTGGTATGAGGTCCACCAAAGCCTGTAACATGGAATTACAATCATAGCCGTTAATCGG

TTCATCTCATCCGCTCATCGTGATCCAGCATGTCAAACGGAGATGGGTTACTGGCGAAAT

GGATGTATGTAAGCCCACAAATCACAAAAATCTGACATTATGATGGAGAGAATTGCAAGG

AGTGTAAGTGAATCTACGCCGTTAGATGGATACTTTTTAACTTCGTTTTGTCAAATATTC

TGTAGGATGAGGATGATGGACAAGGTGGGCGTAGGAAGAAGGGTTTGACGGAGAAGATCA

AAGAGAAGCTGCCAGGTGGGCAGAAGACGACAGGTGTCGGTTATCCAGGGAATCAGGGTG

TACAGGGGGGCCGCGAGCATGAGAAGACAGGTGTCTGTCATCCAGGGACTCAGGGTGTAC

AGGGGGGCCGCGAGCATGAGAAGACCGGTTTCGGTCATCCAGGGACTCAGGGTGGACACG

AGCATGAGAAGACAGGTGTCGGTCATCCAGGGACTCAGGGTGAGCAGGAGAAGAAGGGTA

TGATTGAGAAGATCAAGGAGAAGCTGCCAGGTCACAAGTAGGATGTGTGCCCACCATACA

CGTGGAGTCTACATCTATGTTTCTATGTTACTATAATACGCATGGAGTGTGTACGGATTC

TGTAATAATCTGCGCGTATCTGTTATGTC

>MSL78

AGCCGTTGGATCTAGTTGAAAATGTCAGTGACGCGTGATGAGTATGGCAACCAGGTTCGC

CAGACCGACGAGTATGGGAACCCGATTCAGCATACTGGCACCGGGACAAAGGCCGGTTCG

GGCATACATGGTGGGGGCCATGGGATTGGCACAGGTGGTGGTGGTGGTGGACAAGGCAAG

CTCCACCGTTCAGGCTCTGGCTCTTCCTCTGTAAGTACATTCGATGTTTCTCAAGGGTCA

TTTCACTTGGTATATCGTGTTGGGATGATTAGATTAGTTGATTAACGGTTGTGATTGTAA

ATCAGTGATATGGGCTTCTGTGTACCTTAGTTCACGATGATAATCAATTGATACGCTGGA

TCGTGATGAGCGGACCGGATGAGCCGTCCATCTTACAGGCTTTTTAAAATAGAATTATAG

GGTGGGTGGTATGAGGTCCACCAAAGCCTGTAACATGGAATTACAATCATAGCCGTTAAT

CGGTTCATCTCATCCGCTCATCGTGATCTAGCATGTAAAACAGAGATGGGTTTCTGGAGA

AATGGATGCATGTAAGCCCACAAATCACAAAAATCTGACATTATGATGGAGAGAATGCAA

GGAGTGTAAGCGAATTTACGCCGTTAGATGGATTTCATTGTACTTTTAACTTTGTTTTGT

CAAATATTTTGTAGGATGAGGATGATGGACAAGGTGGGCGTAGGAAGAAAGGTTTGACGG

AGAAGATCAAAGAGAAGCTGCCAGGTGGAAACAAGACGACAGGTGTCTGTCATCCAGGGA

CTCAGGGTGTACAGGGGGGCCGCGAGCATGAGAAGACAGGTTGCGGTCAGGGTGGACAGG

TGCGCCGCGAGCATGAGAAGACAGGTTTCGGCCATCCAGGGACTCAGGGTGGCCAGGTGG

GCAACGAGCAGGAGAAGAAGGGTATGATTGAGAAGATCCAGGAGAACCTGCCAGGTCACA

AGTAGGATGTGTGCCCACCATACACGTGGAGTCTACATCTACGTGTCTATTATGTTACTA

TAATACGCATGGAGTGTGTCCGGAGTCTGTAATAATCTGCGCGTATCTGTTATGTC

>ZZY25

AGCCGTTGGATCTAGTTGAAAATGTCTGAGACGCGTGATGAGTATGGCAACGAGGTTCGC

CAAACCGACGAGTATGGGAACCCGATTCAGCATACTGGCACCGGGACAAAGGGCGGTGCG

GGAATACATGGTGGGGGTCATGGGATTGGCACAGGTGGTGGTGGTGGACAAGGCAAGCTC

CACCGTTCAGGCTCCGGCTCTTCCTCTGTAAGTATATTAGATGTTTCTCAAGAGTCATTT

CACTTGGTAGATTGTGTTGGGATGACTAGATTAGTTGATTAACGGTTGCGATTGTAAATC

AGTGATATGGGCTTCTGTAGACCTTATTTCACGATGATAATCAATTGATACGGTGGATCG

TGATGAGCGGATCGGATGAGCCGTACATCTTACAGGCTTTTTAAAATAGAATTATAGGGT

GGGTGGTATGAGGTCCACCAAAGCCTGTAACATGGAATTACAATCATAGCCGTTAATCGG

TTCATCTCATCCGCTCATCGTGATCCAGCATGTCAAACGGAGATGGGTTACTGGCGAAAT

GGATGTATGTAAGCCCACAAATCACAAAAATCTGACATTATGATGGAGAGAATTGCAAGG

AGTGTAAGTGAATCTACGCCGTTAGATGGATACTTTTTAACTTCGTTTTGTCAAATATTC

TGTAGGATGAGGATGATGGACAAGGTGGGCGTAGGAAGAAGGGTTTGACGGAGAAGATCA

AAGAGAAGCTGCCAGGTGGGCAGAAAACGACAGGTGTCGGTTATCCAGGGAATCAGGGTG

TACAGGGGGGCCGCGAGCATGAGAAGACAGGTGTCTGTCATCCAGGGAATCAGGGTGTAC

AGGGGGGCCGCGAGCATGAGAAGACAGGTTTCGGTCATCCAGGGACTCAGGGTGGACACG

AGCATGAGAAGACAGGTGTCGGTCATCCAGGGACTCAGGGTGAGCAGGAGAAGAAGGGTA

TGATTGAGAAGATCAAGGAGAAGCTGCCAGGTCACAAGTAGGATGTGTGCCCACCATACA

CGTGGAGTCTACATCTATGTTTCTATGTTACTATAATACGCATGGAGTGTGTACGGATTC

TGTAATAATCTGCGCGTATCTGTTATGTC

>ZZY26

AGCCGTTGGATCTAGTTGAAAATGTCAGTGACGCGTGATGAGTATGGCAACCAGGTTCGC

CAGACCGACGAGTATGGGAACCCGATTCAGCATACTGGCACCGGGACAAAGGCCGGTTCG

GGCATACATGGTGGGGGCCATGGGATTGGCACAGGTGGTGGTGGTGGTGGACAAGGCAAG

CTCCACCGTTCAGGCTCTGGCTCTTCCTCTGTAAGTACATTCGATGTTTCTCAAGGGTCA

TTTCACTTGGTATATCGTGTTGGGATGATTAGATTAGTTGATTAACGGTTGTGATTGTAA

ATCAGTGATATGGGCATCTGTGTACCTTAGTTCACGATGATAATCAATTGATACGCTGGA

TCGTGATGAGCGGACCGGATGAGCCGTCCATCTTACAGGCTTTTTAAAATAGAATTATAG

GGTGGGTGGTATGAGGTCCACCAAAGCCTGTAACATGGAATTACAATCATAGCCGTTAAT

CGGTTCATCTCATCCGCTCATCGTGATCTAGCATGTCAAACAGAGATGGGTTTCTGGAGA

AATGGATGTATGTAAGCCCACAAATCACAAAAATCTGACATTATGATGGAGAGAATGCAA

GGAGTGTAAGCGAATTTACGCCGTTAGATGGATTTCATTGTACTTTTAACTTTGTTTTGT

CGAATATTTTGTAGGATGAGGATGATGGACAAGGTGGGCGTAGGAAGAAGGGTTTGACGG

AGAAGATCAAAGAGAAGCTGCCAGGTGGAAACAAGACGACAGGTGTCTGTCATCCAGGGA

CTCAGGGTGTACAGGGGGGCCGCGAGCATGAGAAGACAGGTTGCGGTCAGGGTGGTCAGG

GTGGACAGGTGGGCCGCGAGCATGAGAAGACAGGTTTCGGCCATGCAGTGACTCAGGGTG

GACAGGTGGGCAACGAGCAGGAGAAGAAGGGTATGATTGAGAAGATCCAGGAGAAGCTGC

CAGGTCACAAGTAGGATGTGTGCCCACCATACACGTGGAGTCTACATCTACGTGCCTATT

ATGTTACTATAATACGCATGGAGTGTGTCCGGAGTCTGTAATAATCTGCGCGTATCTGTT

ATGTC

>ZZY212

AGCCGTTGGATCTAGTTGAAAATGTCTGAGACGCGTGATGAGTATGGCAACGTGGTTCGC

CAAACCGACGAGTATGGGAACCCGATTCAGCATACTGGCACCGGGACAAAGGGCGGTGCG

GGAATACATGGTGGGGGTCATGGGATTGGCACAGGTGGTGGTGGTGGACAAGGCAAGCTC

CACCGTTCAGGCTCCGGCTCTTCCTCTGTAAGTATATTAGATGTTTCTCAAGAGTCATTT

CACTTGGTAGATTGTGTTGGGATGACTAGATTAGTTGATTAACGGTTGCGATTGTAAATC

AGTGATATGGGCTTCTGTAGACCTTATTTCACGATGATAATCAATTGATACGGTGGATCG

TGATGAGCGGATCGGATGAGCCGTACATCTTACAGGCTTTTTAAAATAGAATTATAGGGT

GGGTGGTATGAGGTCCACCAAAGCCTGTAACATGGAATTACAATCATAGCCGTTAATCGG

TTCATCTCATCCGCTCATCGTGATCCAGCATGTCAAACGGAGATGGGTTACTGGCGAAAT

GGATGTATGTAAGCCCACAAATCACAAAAATGTGACATTATGATGGAGAGAATTGCAAGG

AGTGTAAGTGAATCTACGCCGTTAGATGGATACTTTTTAACTTCGTTTTGTCAAATATTC

TGTAGGATGAGGATGATGGACAAGGTGGGCGTAGGAAGAAGGGTTTGACGGAGAAGATCA

AAGAGAAGCTGCCAGGTGGGCAGAAGACGACGACAGGTGTGGGTTATCCAGGGACTCAGG

GTGTACAGGGGGGCCGCGAGCATGAGAAGACAGGTGTCTGTCATCCAGGGACTCAGGGTG

TACAGGGGGGCCGCGAGCATGAGAAGACAGGTTTCGGTCATCCAGGGACTCAGGGTGGAC

ACGAGCATGAGAAGACAGGTGTCGGTCATCCAGGGACTCAGGGTGAGCAGGAGAAGAAGG

GTATGATTGAGAAGATCAAGGAGAAGCTGCCTGGTCACAAGTAGGATGTGTGCCCACCAT

ACACGTGGAGTCTACATCTATGTTTCTATGTTACTATAATACGCATGGAGTGTGTACGGA

TTCTGTAATAATCTGCGCGTATCTGTTATGTC

>ZZY34

AGCCGTTGGATCTAGTTGAAAATGTCTGAGACGCGTGATGAGTATGGCAACGAGGTTCGC

CAAACCGACGAGTATGGGAACCCGATTCAGCATACTGGCACCGGGACAAAGGGCGGTGCG

GGAATACATGGTGGGGGTCATGGGATTGGCACAGGTGGTGGTGGTGGACAAGGCAAGCTC

CACCGTTCAGGCTCCGGCTCTTCCTCTGTAAGTATATTAGATGTTTCTCAAGAGTCATTT

CACTTGGTAGATTGTGTTGGGATGACTAGATTAGTTGATTAACGGTTGCGATTGTAAATC

AGTGATATGGGCTTCTGTAGACCTTATTTCACGATGATAATCAATTGATACGGTGGATCG

TGATGAGCGGATCGGATGAGCCGTACATCTTACAGGCTTTTTAAAATAGAATTATAGGGT

GGGTGGTATGAGGTCCACCAAAGCCTGTAACATGGAATTACAATCATAGCCGTTAATCGG

TTCATCTCATCCGCTCATCGTGATCCAGCATGTCAAACGGAGATGGGTTACTGGCGAAAT

GGATGTATGTAAGCCCACAAATCACAAAAATCTGACATTATGATGGAGAGAATTGCAAGG

AGTGTAAGTGAATCTACGCCGTTAGATGGATACTTTTTAACTTCGTTTTGTCAAATATTC

TGTAGGATGAGGATGATGGACAAGGTGGGCGTAGGAAGAAGGGTTTGACGGAGAAGATCA

AAGAGAAGCTGCCAGGTGGGCAGAAAACGACAGGTGTCGGTTATCCAGGGAATCAGGGTG

TACAGGGGGGCCGCGAGCATGAGAAGACAGGTGTCTGTCATCCAGGGAATCAGGGTGTAC

AGGGGGGCCGCGAGCATGAGAAGACAGGTTTCGGTCATCCAGGGACTCAGGGTGGACACG

AGCATGAGAAGACAGGTGTCGGTCATCCAGGGACTCAGGGTGAGCAGGAGAAGAAGGGTA

TGATTGAGAAGATCAAGGAGAAGCTGCCAGGTCACAAGTAGGATGTGTGCCCACCATACA

CGTGGAGTCTACATCTATGTTTCTATGTTACTATAATACGCATGGAGTGTGTACGGATTC

TGTAATAATCTGCGCGTATCTGTTATGTC

>ZZY35

AGCCGTTGGATCTAGTTGAAAATGTCTGAGACGCGTGATGAGTATGGCAACGAGGTTCGC

CAAACCGACGAGTATGGGAACCCGATTCAGCATACTGGCACCGGGACAAAGGGCGGTGCG

GGAATACATGGTGGGGGTCATGGGATTGGCACAGGTGGTGGTGGTGGACAAGGCAAGCTC

CACCGTTCAGGCTCCGGCTCTTCCTCTGTAAGTATATTAGATGTTTCTCAAGAGTCATTT

CACTTGGTAGATTGTGTTGGGATGACTAGATTAGTTGATTAACGGTTGCGATTGTAAATC

AGTGATATGGGCTTCTGTAGACCTTATTTCACGATGATAATCAATTGATACGGTGGATCG

TGATGAGCGGATCGGATGAGCCGTACATCTTACAGGCTTTTTAAAATAGAATTATAGGGT

GGGTGGTATGAGGTCCACCAAAGCCTGTAACATGGAATTACAATCATAGCCGTTAATCGG

TTCATCTCATCCGCTCATCGTGATCCAGCATGTCAAACGGAGATGGGTTACTGGCGAAAT

GGATGTATGTAAGCCCACAAATCACAAAAATCTGACATTATGATGGAGAGAATTGCAAGG

AGTGTAAGTGAATCTACGCCGTTAGATGGATACTTTTTAACTTCGTTTTGTCAAATATTC

TGTAGGATGAGGATGATGGACAAGGTGGGCGTAGGAAGAAGGGTTTGACGGAGAAGATCA

AAGAGAAGCTGCCAGGTGGGCAGAAAACGACAGGTGTCGGTTATCCAGGGAATCAGGGTG

TACAGGGGGGCCGCGAGCATGAGAAGACAGGTGTCTGTCATCCAGGGAATCAGGGTGTAC

AGGGGGGCCGCGAGCATGAGAAGACAGGTTTCGGTCATCCAGGGACTCAGGGTGGACACG

AGCATGAGAAGACAGGTGTCGGTCATCCAGGGACTCAGGGTGAGCAGGAGAAGAAGGGTA

TGATTGAGAAGATCAAGGAGAAGCTGCCAGGTCACAAGTAGGATGTGTGCCCACCATACA

CGTGGAGTCTACATCTATGTTTCTATGTTACTATAATACGCATGGAGTGTGTACGGATTC

TGTAATAATCTGCGCGTATCTGTTATGTC

>ZZY315

AGCCGTTGGATCTAGTTGAAAATGTCTGAGACGCGTGATGAGTATGGCAACGAGGTTCGC

CAAACCGACGAGTATGGGAACCCGATTCAGCATACTGGCACCGGGACAAAGGGCGGTGCG

GGAATACATGGTGGGGGTCATGGGATTGGCACAGGTGGTGGTGGTGGACAAGGCAAGCTC

CACCGTTCAGGCTCCGGCTCTTCCTCTGTAAGTATATTAGATGTTTCTCAAGAGTCATTT

CACTTGGTAGATTGTGTTGGGATGACTAGATTAGTTGATTAACGGTTGCGATTGTAAATC

AGTGATATGGGCTTCTGTAGACCTTATTTCACGATGATAATCAATTGATACGGTGGATCG

TGATGAGCGGATCGGATGAGCCGTACATCTTACAGGCTTTTTAAAATAGAATTATAGGGT

GGGTGGTATGAGGTCCACCAAAGCCTGTAACATGGAATTACAATCATAGCCGTTAATCGG

TTCATCTCATCCGCTCATCGTGATCCAGCATGTCAAACGGAGATGGGTTACTGGCGAAAT

GGATGTATGTAAGCCCACAAATCACAAAAATCTGACATTATGATGGAGAGAATTGCAAGG

AGTGTAAGTGAATCTACGCCGTTAGATGGATACTTTTTAACTTCGTTTTGTCAAATATTC

TGTAGGATGAGGATGATGGACAAGGTGGGCGTAGGAAGAAGGGTTTGACGGAGAAGATCA

AAGAGAAGCTGCCAGGTGGGCAGAAAACGACAGGTGTCGGTTATCCAGGGAATCAGGGTG

TACAGGGGGGCCGCGAGCATGAGAAGACAGGTGTCTGTCATCCAGGGAATCAGGGTGTAC

AGGGGGGCCGCGAGCATGAGAAGACAGGTTTCGGTCATCCAGGGACTCAGGGTGGACACG

AGCATGAGAAGACAGGTGTCGGTCATCCAGGGACTCAGGGTGAGCAGGAGAAGAAGGGTA

TGATTGAGAAGATCAAGGAGAAGCTGCCAGGTCACAAGTAGGATGTGTGCCCACCATACA

CGTGGAGTCTACATCTATGTTTCTATGTTACTATAATACGCATGGAGTGTGTACGGATTC

TGTAATAATCTGCGCGTATCTGTTATGTC

>ZZY45

AGCCGTTGGATCTAGTTGAAAATGTCTGAGACGCGTGATGAGTATGGCAACCAGGTTCGC

CAAACCGACGAGTATGGGAACCCGATTCAGCATACTGGCACCGGCACAAAGGCCGGTGCG

GGCATACATGCTGGGGGCCATGGGATTGGCACAGGTGGTGGTGGTGGACAAGGCAAGCTC

CACCGTTCAGGCTCCGGGTCTTCCTCTGTAAGTATATTCGATGTTTCTCAAGGGTCATTT

CACTTGGTAGATCGTGTTGTGATGACTAGATTAGTTGATTAACCGTTGTGATTGTAAATC

AGTGATATGGGCTTCTGTGTTCCTTATTTCACAATGATAATCAATTGATACGGTGGATCG

TGATGAGCGGACCGAATGAGCCGTCCATCTTACAGGCTTTTTAAAATAGAATTATAGGGT

GGGTGGTATGAGGTCCACCAAAGCCTGTAACATGGAATTACAATTATAGCCGTTAATCGG

TTCATCTCATCCGCTCATCGTGATCCAGCATGTCAAACAGAGATGGGTTTCTGGCGAAAT

GGATGTATGTAAGCCCACAAATCACAAAAATCTGACATTATGATGGAGAGAATTGCAAGG

AGTGTAAGTGAATCTACGCCGTTAGATGGATACTTTTTAACTTCGTTTTGTCAAATATTC

TGTAGGATGAGGATGATGGACAAGGTGGGCGTAGGAAGAAGGGTTTGACGGAGAAGATCA

AAGAGAAGCTGCCAGGTGGGCAGAAGACGACAGGTGTCGGTTATCCAGGGAATCAGGGTG

TACAGGGGGGCCGCGAGCATGAGAAGACAGGTGTCTGTCATCCAGGGACTCAGGGTGTAC

AGGGGGGCCGCGAGCATGAGAAGACAGGTTTCGGTCATCCAGGGACTCAGGGTGGACACG

AGCATGAGAAGACAGGTGTCGGTCATCCAGGGACTCAGGGTGAGCAGGAGAAGAAGGGTA

TGATTGAGAAGATCAAGGAGAAGCTGCCAGGTCACAAGTAGGATGTGTGCCCACCATACA

CGTGGAGTCTACATCTATGTTTCTATGTTACTATAATACGCATGGAGTGTGTACGGATTC

TGTAATAATCTGCGCGTATCTGTTATGTC

>ZZY46

AGCCGTTGGATCTAGTTGAAAATGTCAGTGACGCGTGATGAGTATGGCAACCAGGTTCGC

CAGACCGACGAGTATGGGAACCCGATTCAGCATACTGGCACCGGGACAAAGGCCGGTTCG

GGCATACATGGTGGGGGCCATGGGATTGGCACAGGTGGTGGTGGTGGTGGACAAGGCAAG

CTCCACCGTTCAGGCTCTGGCTCTTCCTCTGTAAGTACATTCGATGTTTCTCAAGGGTCA

TTTCACTTGGTATATCGTGTTGGGATGATTAGATTAGTTGATTAACGGTTGTGATTGTAA

ATCAGTGATATGGGCATCTGTGTACCTTAGTTCACGATGATAATCAATTGATACGCTGGA

TCGTGATGAGCGGACCGGATGAGCCGTCCATCTTACAGGCTTTTTAAAATAGAATTATAG

GGTGGGTGGTATGAGGTCCACCAAAGCCTGTAACATGGAATTACAATCATAGCCGTTAAT

CGGTTCATCTCATCCGCTCATCGTGATCTAGCATGTCAAACAGAGATGGGTTTCTGGAGA

AATGGATGTATGTAAGCCCACAAATCACAAAAATCTGACATTATGATGGAGAGAATGCAA

GGAGTGTAAGCGAATTTACGCCGTTAGATGGATTTCATTGTACTTTTAACTTTGTTTTGT

CGAATATTTTGTAGGATGAGGATGATGGACAAGGTGGGCGTAGGAAGAAGGGTTTGACGG

AGAAGATCAAAGAGAAGCTGCCAGGTGGGCAGAAGACGACAGGTGTCGGTTATCCAGGGA

ATCAGGGTGTACAGGGGGGCCGCGAGCATGAGAAGACAGGTGTCTGTCATCCAGGGACTC

AGGGTGTACAGGGGGGCCGCGAGCATGAGAAGACAGGTTTCGGTCATCCAGGGACTCAGG

GTGGACACGAGCATGAGAAGACAGGTGTCGGTCATCCAGGGACTCAGGGTGAGCAGGAGA

AGAAGGGTATGATTGAGAAGATCAAGGAGAAGCTGCCAGGTCACAAGTAGGATGTGTGCC

CACCATACACGTGGAGTCTACATCTATGTTTCTATGTTACTATAATACGCATGGAGTGTG

TACGGATTCTGTAATAATCTGCGCGTATCTGTTATGTC

>ZZY412

AGCCGTTGGATCTAGTTGAAAATGTCAGTGACGCGTGATGAGTATGGCAACCAGGTTCGG

CAGACCGACGAGTATGGGAACCCGATTCAGCATACTGGCACCGGGACAAAGGCCGGTTCG

GGCATACATGGTGGGGGCCATGGGATTGGCACAGGTGGTGGTGGTGGTGGACAAGGCAAG

CTCCACCGTTCAGGCTCTGGCTCTTCCTCTGTAAGTACATTCGATGTTTCTCAAGGGTCA

TTTCACTTGGTATATCGTGTTGGGATGATTAGATTAGTTGATTAACGGTTGTGATTGTAA

ATCAGTGATATGGGCTTCTGTGTACCTTAGTTCACGATGATAATCAATTGATACGCTGGA

TCGTGATGAGCGGACCGGATGAGCCGTCCATCTTACAGGCTTTTTAAAATAGAATTATAG

GGTGGGTGGTATGAGGTCCACCAAAGCCTGTAACATGGAATTACAATCATAGCCGTTAAT

CGGTTCATCTCATCCGCTCATCGTGATCTAGCATGTAAAACAGAGATGGGTTTCTGGAGA

AATGGATGCATGTAAGCCCACAAATCACAAAAATCTGACATTATGATGGAGAGAATGCAA

GGAGTGTAAGCGAATTTACGCCGTTAGATGGATTTCATTGTACTTTTAACTTTGTTTTGT

CGAATATTTTGTAGGATGAGGATGATGGACAAGGTGGGCGTAGGAAGAAGGGTTTGACGG

AGAAGATCAAAGAGAAGCTGCCAGGTGGAAACAAGACGACAGGTGTCTGTCATCCAGGGA

CTCAGGGTGTACAGGGGGGCCGCGAGCATGAGAAGACAGGTTGCGGTCAGGGTGGTCAGG

GTGGACAGGTGGGCCGCGAGCATGAGAAGACAGGTTTCGGCCATGCAGTGACTCAGGGTG

GACAGGTGGGCAACGAGCAGGAGAAGAAGGGTATGATTGAGAAGATCAAGGAGAAGCTGC

CAGGTCACAAGTAGGATGTGTGCCCACCATACACGTGGAGTCTACATCTACGTGCCTATT

ATGTTACTATAATACGCATGGAGTGTGTCCGGAGTCTGTAATAATCTGCGCGTATCTGTT

ATGTC

>ZZY53

AGCCGTTGGATCTAGTTGAAAATGTCTGAGACGCGTGATGAGTATGGCAACGAGGTTCGC

CAAACCGACGAGTATGGGAACCCGATTCAGCATACTGGCACCGGGACAAAGGGCGGTGCG

GGAATACATGGTGGGGGTCATGGGATTGGCACAGGTGGTGGTGGTGGACAAGGCAAGCTC

CACCGTTCAGGCTCCGGCTCTTCCTCTGTAAGTATATTAGATGTTTCTCAAGAGTCATTT

CACTTGGTAGATTGTGTTGGGATGACTAGATTAGTTGATTAACGGTTGCGATTGTAAATC

AGTGATATGGGCTTCTGTAGACCTTATTTCACGATGATAATCAATTGATACGGTGGATCG

TGATGAGCGGATCGGATGAGCCGTACATCTTACAGGCTTTTTAAAATAGAATTATAGGGT

GGGTGGTATGAGGTCCACCAAAGCCTGTAACATGGAATTACAATCATAGCCGTTAATCGG

TTCATCTCATCCGCTCATCGTGATCCAGCATGTCAAACGGAGATGGGTTACTGGCGAAAT

GGATGTATGTAAGCCCACAAATCACAAAAATCTGACATTATGATGGAGAGAATTGCAAGG

AGTGTAAGTGAATCTACGCCGTTAGATGGATACTTTTTAACTTCGTTTTGTCAAATATTC

TGTAGGATGAGGATGATGGACAAGGTGGGCGTAGGAAGAAGGGTTTGACGGAGAAGATCA

AAGAGAAGCTGCCAGGTGGGCAGAAAACGACAGGTGTCGGTTATCCAGGGAATCAGGGTG

TACAGGGGGGCCGCGAGCATGAGAAGACAGGTGTCTGTCATCCAGGGAATCAGGGTGTAC

AGGGGGGCCGCGAGCATGAGAAGACAGGTTTCGGTCATCCAGGGACTCAGGGTGGACACG

AGCATGAGAAGACAGGTGTCGGTCATCCAGGGACTCAGGGTGAGCAGGAGAAGAAGGGTA

TGATTGAGAAGATCAAGGAGAAGCTGCCAGGTCACAAGTAGGATGTGTGCCCACCATACA

CGTGGAGTCTACATCTATGTTTCTATGTTACTATAATACGCATGGAGTGTGTACGGATTC

TGTAATAATCTGCGCGTATCTGTTATGTC

>ZZY55

AGCCGTTGGATCTAGTTGAAAATGTCTGAGACGCGTGATGAGTATGGCAACCAGGTTCGC

CAAACCGACGAGTATGGGAACCCGATTCAGCATACTGGCACCGGCACAAAGGCCGGTGCG

GGCATACATGCTGGGGGCCATGGGATTGGCACAGGTGGTGGTGGTGGACAAGGCAAGCTC

CACCGTTCAGGCTCCGGGTCTTCCTCTGTAAGTATATTCGATGTTTCTCAAGGGTCATTT

CACTTGGTAGATCGTGTTGTGATGACTAGATTAGTTGATTAACTGTTGTGATTGTAAATC

AGTGATATGGGCTTCTGTGTTCCTTATTTCACAATGATAATCAATTGATACGGTGGATCG

TGATGAGCGGACCGAATGAGCCGTCCATCTTACAGGCTTTTTAAAATAGAATTATAGGGT

GGGTGGTATGAGGTCCACCAAAGCCTGTAACATGGAATTACAATTATAGCCGTTAATCGG

TTCATCTCATCCGCTCATCGTGATCCAGCATGTCAAACAGAGATGGGTTTCTGGCGAAAT

GGATGTATGTAAGCCCACAAATCACAAAAATCTGACATTATGATGGAGAGAATTGCCAGG

AGTGTAAGTGAATCTACGCCGTTAGATGGATACTTTTTAACTTCGTTTTGTCAAATATTC

TGTAGGATGAGGATGATGGACAAGGTGGGCGTAGGAAGAAGGGTTTGACGGAGAAGATCA

AAGAGAAGCTGCCAGGTGGGCAGAAGACGACAGGTGTCGGTTATCCAGGGAATCAGGGTG

TACAGGGGGGCCGCGAGCATGAGAAGACAGGTGTCTGTCATCCAGGGACTCAGGGTGTAC

AGGGGGGCCGCGAGCATGAGAAGACAGGTTTCGGTCATCCAGGGACTCAGGGTGGACACG

AGCATGAGAAGACAGGTGTCGGTCATCCAGGGACTCAGGGTGAGCAGGAGAAGAAGGGTA

TGATTGAGAAGATCAAGGAGAAGCTGCCAGGTCACAAGTAGGATGTGTGCCCACCATACA

CGTGGAGTCTACATCTATGTTTCTATGTTACTATAATACGCATGGAGTGTGTACGGATTC

TGTAATAATCTGCGCGTATCTGTTATGTC

>ZZY511

AGCCGTTGGATCTAGTTGAAAATGTCTGAGACGCGTGATGAGTATGGCAACGAGGTTCGC

CAAACCGACGAGTATGGGAACCCGATTCAGCATACTGGCACCGGGACAAAGGGCGGTGCG

GGAATACATGGTGGGGGTCATGGGATTGGCACAGGTGGTGGTGGTGGACAAGGCAAGCTC

CACCGTTCAGGCTCCGGCTCTTCCTCTGTAAGTATATTCGATGTTTCTCAAGGGTCATTT

CACTTGGTAGATCGTGTTGTGATGACTAGATTAGTTGATTAACTGTTGTGATTGTAAATC

AGTGATATGGGCTTCTGTGTTCCTTATTTCACAATGATAATCAATTGATACGGTGGATCG

TGATGAGCGGACCGAATGAGCCGTCCATCTTACAGGCTTTTTAAAATAGAATTATAGGGT

GGGTGGTATGAGGTCCACCAAAGCCTGTAACATGGAATTACAATTATAGCCGTTAATCGG

TTCATCTCATCCGCTCATCGTGATCCAGCATGTCAAACAGAGATGGGTTTCTGGCGAAAT

GGATGTATGTAAGCCCACAAATCACAAAAATCTGACATTATGATGGAGAGAATTGCCAGG

AGTGTAAGTGAATCTACGCCGTTAGATGGATACTTTTTAACTTCGTTTTGTCAAATATTC

TGTAGGATGAGGATGATGGACAAGGTGGGCGTAGGAAGAAGGGTTTGACGGAGAAGATCA

AAGAGAAGCTGCCAGGTGGGCAGAAGACGACAGGTGTCGGTTATCCAGGGAATCAGGGTG

TACAGGGGGGCCGCGAGCATGAGAAGACAGGTGTCTGTCATCCAGGGACTCAGGGTGTAC

AGGGGGGCCGCGAGCATGAGAAGACAGGTTTCGGTCATCCAGGGACTCAGGGTGGACACG

AGCATGAGAAGACAGGTGTCGGTCATCCAGGGACTCAGGGTGAGCAGGAGAAGAAGGGTA

TGATTGAGAAGATCAAGGAGAAGCTGCCAGGTCACAAGTAGGATGTGTGCCCACCATACA

CGTGGAGTCTACATCTATGTTTCTATGTTACTATAATACGCATGGAGTGTGTACGGATTC

TGTAATAATCTGCGCGTATCTGTTATGTC

>ZZY62

AGCCGTTGGATCTAGTTGAAAATGTCTGAGACGCGTGATGAGTATGGCAACGAGGTTCGC

CAAACCGACGAGTATGGGAACCCGATTCAGCATACTGGCACCGGGACAAAGGGCGGTGCG

GGAATACATGGTGGGGGTCATGGGATTGGCACAGGTGGTGGTGGTGGACAAGGCAAGCTC

CACCGTTCAGGCTCCGGCTCTTCCTCTGTAAGTATATTAGATGTTTCTCAAGAGTCATTT

CACTTGGTAGATTGTGTTGGGATGACTAGATTAGTTGATTAACGGTTGCGATTGTAAATC

AGTGATATGGGCTTCTGTAGACCTTATTTCACGATGATAATCAATTGATACGGTGGATCG

TGATGAGCGGATCGGATGAGCCGTACATCTTACAGGCTTTTTAAAATAGAATTATAGGGT

GGGTGGTATGAGGTCCACCAAAGCCTGTAACATGGAATTACAATCATAGCCGTTAATCGG

TTCATCTCATCCGCTCATCGTGATCCAGCATGTCAAACGGAGATGGGTTACTGGCGAAAT

GGATGTATGTAAGCCCACAAATCACAAAAATCTGACATTATGATGGAGAGAATTGCAAGG

AGTGTAAGTGAATCTACGCCGTTAGATGGATACTTTTTAACTTCGTTTTGTCAAATATTC

TGTAGGATGAGGATGATGGACAAGGTGGGCGTAGGAAGAAGGGTTTGACGGAGAAGATCA

AAGAGAAGCTGCCAGGTGGGCAGAAAACGACAGGTGTCGGTTATCCAGGGAATCAGGGTG

TACAGGGGGGCCGCGAGCATGAGAAGACAGGTGTCTGTCATCCAGGGAATCAGGGTGTAC

AGGGGGGCCGCGAGCATGAGAAGACAGGTTTCGGTCATCCAGGGACTCAGGGTGGACACG

AGCATGAGAAGACAGGTGTCGGTCATCCAGGGACTCAGGGTGAGCAGGAGAAGAAGGGTA

TGATTGAGAAGATCAAGGAGAAGCTGCCAGGTCACAAGTAGGATGTGTGCCCACCATACA

CGTGGAGTCTACATCTATGTTTCTATGTTACTATAATACGCATGGAGTGTGTACGGATTC

TGTAATAATCTGCGCGTATCTGTTATGTC

>ZZY615

AGCCGTTGGATCTAGTTGAAAATGTCAGTGACGCGTGATGAGTATGGCAACCAGGTTCGC

CAGACCGACGAGTATGGGAACCCGATTCAGCATACTGGCACCGGGACAAAGGCCGGTTCG

GGCATACATGGTGGGGGCCATGGGATTGGCACAGGTGGTGGTGGTGGTGGACAAGGCAAG

CTCCACCGTTCAGGCTCTGGCTCTTCCTCTGTAAGTACATTCGATGTTTCTCAAGGGTCA

TTTCACTTGGTATATCGTGTTGGGATGATTAGATTAGTTGATTAACGGTTGTGATTGTAA

ATCAGTGATATGGGCATCTGTGTACCTTAGTTCACGATGATAATCAATTGATACGCTGGA

TCGTGATGAGCGGACCGGATGAGCCGTCCATCTTACAGGCTTTTTAAAATAGAATTATAG

GGTGGGTGGTATGAGGTCCACCAAAGCCTGTAACATGGAATTACAATCATAGCCGTTAAT

CGGTTCATCTCATCCGCTCATCGTGATCTAGCATGTCAAACAGAGATGGGTTTCTGGAGA

AATGGATGTATGTAAGCCCACAAATCACAAAAATCTGACATTATGATGGAGAGAATGCAA

GGAGTGTAAGCGAATTTACGCCGTTAGATGGATTTCATTGTACTTTTAACTTTGTTTTGT

CGAATATTTTGTAGGATGAGGATGATGGACAAGGTGGGCGTAGGAAGAAGGGTTTGACGG

AGAAGATCAAAGAGAAGCTGCCAGGTGGAAACAAGACGACAGGTGTCTGTCATCCAGGGA

CTCAGGGTGTACAGGGGGGCCGCGAGCATGAGAAGACAGGTTGCGGTCAGGGTGGTCAGG

GTGGACAGGTGGGCCGCGAGCATGAGAAGACAGGTTTCGGCCATGCAGTGACTCAGGGTG

GACAGGTGGGCAACGAGCAGGAGAAGAAGGGTATGATTGAGAAGATCCAGGAGAAGCTGC

CAGGTCACAAGTAGGATGTGTGCCCACCATACACGTGGAGTCTACATCTACGTGCCTATT

ATGTTACTATAATACGCATGGAGTGTGTCCGGAGTCTGTAATAATCTGCGCGTATCTGTT

ATGTC

>ZZY632

AGCCGTTGGATCTAGTTGAAAATGTCTGAGACGCGTGATGAGTATGGCAACGTGGTTCGC

CAAACCGACGAGTATGGGAACCCGATTCAGCATACTGGCACCGGGACAAAGGGCGGTGCG

GGAATACATGGTGGGGGTCATGGGATTGGCACAGGTGGTGGTGGTGGACAAGGCAAGCTC

CACCGTTCAGGCTCCGGCTCTTCCTCTGTAAGTATATTAGATGTTTCTCAAGAGTCATTT

CACTTGGTAGATTGTGTTGGGATGACTAGATTAGTTGATTAACGGTTGCGATTGTAAATC

AGTGATATGGGCTTCTGTAGACCTTATTTCACGATGATAATCAATTGATACGGTGGATCG

TGATGAGCGGATCGGATGAGCCGTACATCTTACAGGCTTTTTAAAATAGAATTATAGGGT

GGGTGGTATGAGGTCCACCAAAGCCTGTAACATGGAATTACAATCATAGCCGTTAATCGG

TTCATCTCATCCGCTCATCGTGATCCAGCATGTCAAACGGAGATGGGTTACTGGCGAAAT

GGATGTATGTAAGCCCACAAATCACAAAAATGTGACATTATGATGGAGAGAATTGCAAGG

AGTGTAAGTGAATCTACGCCGTTAGATGGATACTTTTTAACTTCGTTTTGTCAAATATTC

TGTAGGATGAGGATGATGGACAAGGTGGGCGTAGGAAGAAGGGTTTGACGGAGAAGATCA

AAGAGAAGCTGCCAGGTGGGCAGAAGACGACGACAGGTGTGGGTTATCCAGGGACTCAGG

GTGTACAGGGGGGCCGCGAGCATGAGAAGACAGGTGTCTGTCATCCAGGGACTCAGGGTG

TACAGGGGGGCCGCGAGCATGAGAAGACAGGTTTCGGTCATCCAGGGACTCAGGGTGGAC

ACGAGCATGAGAAGACAGGTGTCGGTCATCCAGGGACTCAGGGTGAGCAGGAGAAGAAGG

GTATGATTGAGAAGATCAAGGAGAAGCTGCCTGGTCACAAGTAGGATGTGTGCCCACCAT

ACACGTGGAGTCTACATCTATGTTTCTATGTTACTATAATACGCATGGAGTGTGTACGGA

TTCTGTAATAATCTGCGCGTATCTGTTATGTC

>NK14

AGCCGTTGGATCTAGTTGAAAATGTCTGAGACGCGTGATGAGTATGGCAACCAGGTTCGC

CAAACCGACGAGTATGGGAACCCGATTCAGCATACTGGCACCGGCACAAAGGCCGGTGCG

GGCATACATGCTGGGGGCCATGGGATTGGCACAGGTGGTGGTGGTGGACAAGGCAAGCTC

CACCGTTCAGGCTCCGGGTCTTCCTCTGTAAGTATATTCGATGTTTCTCAAGGGTCATTT

CACTTGGTAGATCGTGTTGTGATGACTAGATTAGTTGATTAACTGTTGTGATTGTAAATC

AGTGATATGGGCTTCTGTGTTCCTTATTTCACAATGATAATCAATTGATACGGTGGATCG

TGATGAGCGGACCGAATGAGCCGTCCATCTTACAGGCTTTTTAAAATAGAATTATAGGGT

GGGTGGTATGAGGTCCACCAAAGCCTGTAACATGGAATTACAATTATAGCCGTTAATCGG

TTCATCTCATCCGCTCATCGTGATCCAGCATGTCAAACAGAGATGGGTTTCTGGCGAAAT

GGATGTATGTAAGCCCACAAATCACAAAAATCTGACATTATGATGGAGAGAATTGCCAGG

AGTGTAAGTGAATCTACGCCGTTAGATGGATACTTTTTAACTTCGTTTTGTCAAATATTC

TGTAGGATGAGGATGATGGACAAGGTGGGCGTAGGAAGAAGGGTTTGACGGAGAAGATCA

AAGAGAAGCTGCCAGGTGGGCAGAAGACGACAGGTGTCGGTTATCCAGGGAATCAGGGTG

TACAGGGGGGCCGCGAGCATGAGAAGACAGGTGTCTGTCATCCAGGGACTCAGGGTGTAC

AGGGGGGCCGCGAGCATGAGAAGACAGGTTTCGGTCATCCAGGGACTCAGGGTGGACACG

AGCATGAGAAGACAGGTGTCGGTCATCCAGGGACTCAGGGTGAGCAGGAGAAGAAGGGTA

TGATTGAGAAGATCAAGGAGAAGCTGCCAGGTCACAAGTAGGATGTGTGCCCACCATACA

CGTGGAGTCTACATCTATGTTTCTATGTTACTATAATACGCATGGAGTGTGTACGGATTC

TGTAATAATCTGCGCGTATCTGTTATGTC

>NK15

AGCCGTTGGATCTAGTTGAAAATGTCAGTGACGCGTGATGAGTATGGCAACCAGGTTCGC

CAGACCGACGAGTATGGGAACCCGATTCAGCATACTGGCACCGGGACAAAGGCCGGTTCG

GGCATACATGGTGGGGGCCATGGGATTGGCACAGGTGGTGGTGGTGGTGGACAAGGCAAG

CTCCACCGTTCAGGCTCTGGCTCTTCCTCTGTAAGTACATTCGATGTTTCTCAAGGGTCA

TTTCACTTGGTATATCGTGTTGTGATGACTAGATTAGTTGATTAACTGTTGTGATTGTAA

ATCAGTGATATGGGCTTCTGTGTTCCTTATTTCACAATGATAATCAATTGATACGGTGGA

TCGTGATGAGCGGACCGAATGAGCCGTCCATCTTACAGGCTTTTTAAAATAGAATTATAG

GGTGGGTGGTATGAGGTCCACCAAAGCCTGTAACATGGAATTACAATCATAGCCGTTAAT

CGGTTCATCTCATCCGCTCATCGTGATCTAGCATGTCAAACAGAGATGGGTTTCTGGAGA

AATGGATGTATGTAAGCCCACAAATCACAAAAATCTGACATTATGATGGAGAGAATGCAA

GGAGTGTAAGCGAATTTACGCCGTTAGATGGATTTCATTGTACTTTTAACTTTGTTTTGT

CGAATATTTTGTAGGATGAGGATGATGGACAAGGTGGGCGTAGGAAGAAGGGTTTGACGG

AGAAGATCAAAGAGAAGCTGCCAGGTGGAAACAAGACGACAGGTGTCTGTCATCCAGGGA

CTCAGGGTGTACAGGGGGGCCGCGAGCATGAGAAGACAGGTTGCGGTCAGGGTGGTCAGG

GTGGACAGGTGGGCCGCGAGCATGAGAAGACAGGTTTCGGCCATGCAGTGACTCAGGGTG

GACAGGTGGGCAACGAGCAGGAGAAGAAGGGTATGATTGAGAAGATCCAGGAGAAGCTGC

CAGGTCACAAGTAGGATGTGTGCCCACCATACACGTGGAGTCTACATCTACGTGCCTATT

ATGTTACTATAATACGCATGGAGTGTGTCCGGAGTCTGTAATAATCTGCGCGTATCTGTT

ATGTC

>NK112

AGCCGTTGGATCTAGTTGAAAATGTCTGAGACGCGTGATGAGTATGGCAACCAGGTTCGC

CAAACCGACGAGTATGGGAACCCGATTCAGCATACTGGCACCGGGACAAAGGGCGGTGCG

GGAATACATGGTGGGGGTCATGGGATTGGCACAGGTGGTGGTGGTGGACAAGGCAAGCTC

CACCGTTCAGGCTCCGGCTCTTCCTCTGTAAGTATATTAGATGTTTCTCAAGAGTCATTT

CACTTGGTAGATTGTGTTGGGATGACTAGATTAGTTGATTAACGGTTGCGATTGTAAATC

AGTGATATGGGCTTCTGTCTACCTTATTTCATGATGATAATCAATTGATACGGTGGATCG

TGATGAGCGGATCGGATGAGCCGTACATCTTACAGGCTTTTTAAAATAGAATTATAGGGT

GGGTGGTATGAGGTCCACCAAAGCCTGTAACATGGAATTACAATCATAGCCGTTAATAGG

TTCATCTCATCCGCTCATCGTGATCCAGCATGTCAAACAGAGATGGGTTTCTGGCGAAAT

GGATGTATGTAAGCCCACAAATCACAAAAATCTGACATTATGATGGAGAGAATGCAAGGA

GTGTAAGCGAATTTACGCCGTTAGATGGATTTCATTGTACTTTTAACTTTGTTTTGTCGA

ATATTTTGTAGGATGAGGATGATGGACAAGGTGGGCGTAGGAAGAAGGGTTTGACGGAGA

AGATCAAAGAGAAGCTGCCAGGTGGAAACAAGACGACAGGTGTCTGTCATCCAGGGACTC

AGGGTGTACAGGGGGGCCGCGAGCATGAGAAGACAGGTTGCGGTCAGGGTGGTCAGGGTG

GACAGGTGGGCCGCGAGCATGAGAAGACAGGTTTCGGCCATGCAGTGACTCAGGGTGGAC

AGGTGGGCAACGAGCAGGAGAAGAAGGGTATGATTGAGAAGATCCAGGAGAAGCTGCCAG

GTCACAAGTAGGATGTGTGCCCACCATACACGTGGAGTCTACATCTACGTGCCTATTATG

TTACTATAATACGCATGGAGTGTGTCCGGAGTCTGTAATAATCTGCGCGTATCTGTTATG

TC

>NK23

AGCCGTTGGATCTAGTTGAAAATGTCTGAGACGCGTGATGAGTATGGCAACGAGGTTCGC

CAAACCGACGAGTATGGGAACCCGATTCAGCATACTGGCACCGGGACAAAGGGCGGTGCG

GGAATACATGGTGGGGGTCATGGGATTGGCACAGGTGGTGGTGGTGGACAAGGCAAGCTC

CACCGTTCAGGCTCCGGCTCTTCCTCTGTAAGTATATTAGATGTTTCTCAAGAGTCATTT

CACTTGGTAGATTGTGTTGGGATGACTAGATTAGTTGATTAACGGTTGCGATTGTAAATC

AGTGATATGGGCTTCTGTAGACCTTATTTCACGATGATAATCAATTGATACGGTGGATCG

TGATGAGCGGATCGGATGAGCCGTACATCTTACAGGCTTTTTAAAATAGAATTATAGGGT

GGGTGGTATGAGGTCCACCAAAGCCTGTAACATGGAATTACAATCATAGCCGTTAATCGG

TTCATCTCATCCGCTCATCGTGATCCAGCATGTCAAACGGAGATGGGTTACTGGCGAAAT

GGATGTATGTAAGCCCACAAATCACAAAAATCTGACATTATGATGGAGAGAATTGCAAGG

AGTGTAAGTGAATCTACGCCGTTAGATGGATACTTTTTAACTTCGTTTTGTCAAATATTC

TGTAGGATGAGGATGATGGACAAGGTGGGCGTAGGAAGAAGGGTTTGACGGAGAAGATCA

AAGAGAAGCTGCCAGGTGGGCAGAAAACGACAGGTGTCGGTTATCCAGGGAATCAGGGTG

TACAGGGGGGCCGCGAGCATGAGAAGACAGGTGTCTGTCATCCAGGGACTCAGGGTGTAC

AGGGGGGCCGCGAGCATGAGAAGACAGGTTTCGGTCATCCAGGGACTCAGGGTGGACACG

AGCATGAGAAGACAGGTGTCGGTCATCCAGGGACTCAGGGTGAGCAGGAGAAGAAGGGTA

TGATTGAGAAGATCAAGGAGAAGCTGCCAGGTCACAAGTAGGATGTGTGCCCACCATACA

CGTGGAGTCTACATCTATGTTTCTATGTTACTATAATACGCATGGAGTGTGTACGGATTC

TGTAATAATCTGCGCGTATCTGTTATGTC

>NK27

AGCCGTTGGATCTAGTTGAAAATGTCTGAGACGCGTGATGAGTATGGCAACCAGGTTCGC

CAAACCGACGAGTATGGGAACCCGATTCAGCATACTGGCACCGGGACAAAGGCCGGTGCG

GGCATACATGGTGGGGGCCATGGGATTGGCACAGGTGGTGGTGGTGGACAAGGCAAGCTC

CACCGTTCAGGCTCCGGGTCTTCCTCTGTAAGTATATTCGATGTTTCTCAAGGGTCATTT

CACTTGGTAGATCGTGTTGTGATGACTAGATTAGTTGATTAACCGTTGTGATTGTAAATC

AGTGATATGGGCTTCTGTGTACCTTATTTCACGATGATAATCAATTGATACGGTGGATCG

TGATGAGCGGACCGGATGAGCCGTCCATCTTACAGGCTTTTTAAAATAGAATTATAGGGT

GGGTGGTATGAGGTCCACCAAAGCCTGTAACATGGAATTACAATCATAGCCGTTAATCGG

TTCATCTCATCCGCTCATCGTGATCCAGCATGTCAAACAGAGATGGGTTTCTGGCGAAAT

GGATGTATGTAAGCCCACAAATCACAAAAATCTGACATTATGATGGAGAGAATTGCAAGG

AGTGTAAGTGAATCTACGCCGTTAGATGGATACTTCAAGGAGTGTAAGTGAATCTACGCC

GTTAGATGGATACTTTTTAACTTCGTTTTGTCAAATATTCTGTAGGATGAGGATGATGGA

CAAGGTGGGCGTAGGAAGAAGGGTTTGACGGAGAAGATCAAAGAGAAGCTGCCAGGTGGG

CAGAAAACGACAGGTGTCGGTTATCCAGGGAATCAGGGTGTACAGGGGGGCCGCGAGCAT

GAGAAGACAGGTGTCTGTCATCCAGGGAATCAGGGTGTACAGGGGGGCCGCGAGCATGAG

AAGACAGGTTTCGGTCATCCAGGGACTCAGGGTGGACACGAGCATGAGAAGACAGGTGTC

GGTCATCCAGGGACTCAGGGTGAGCAGGAGAAGAAGGGTATGATTGAGAAGATCAAGGAG

AAGCTGCCAGGTCACAAGTAGGATGTGTGCCCACCATACACGTGGAGTCTACATCTATGT

TTCTATGTTACTATAATACGCATGGAGTGTGTACGGATTCTGTAATAATCTGCGCGTATC

TGTTATGTC

>NK216

AGCCGTTGGATCTAGTTGAAAATGTCTGAGACGCGTGATGAGTATGGCAACGAGGTTCGC

CAAACCGACGAGTATGGGAACCCGATTCAGCATACTGGCACCGGGACAAAGGGCGGTGCG

GGAATACATGGTGGGGGTCATGGGATTGGCACAGGTGGTGGTGGTGGACAAGGCAAGCTC

CACCGTTCAGGCTCCGGCTCTTCCTCTGTAAGTATATTAGATGTTTCTCAAGAGTCATTT

CACTTGGTAGATTGTGTTGGGATGACTAGATTAGTTGATTAACGGTTGCGATTGTAAATC

AGTGATATGGGCTTCTGTAGACCTTATTTCACGATGATAATCAATTGATACGGTGGATCG

TGATGAGCGGATCGGATGAGCCGTACATCTTACAGGCTTTTTAAAATAGAATTATAGGGT

GGGTGGTATGAGGTCCACCAAAGCCTGTAACATGGAATTACAATCATAGCCGTTAATCGG

TTCATCTCATCCGCTCATCGTGATCCAGCATGTCAAACGGAGATGGGTTACTGGCGAAAT

GGATGTATGTAAGCCCACAAATCACAAAAATCTGACATTATGATGGAGAGAATTGCAAGG

AGTGTAAGTGAATCTACGCCGTTAGATGGATACTTTTTAACTTCGTTTTGTCAAATATTC

TGTAGGATGAGGATGATGGACAAGGTGGGCGTAGGAAGAAGGGTTTGACGGAGAAGATCA

AAGAGAAGCTGCCAGGTGGGCAGAAAACGACAGGTGTCGGTTATCCAGGGAATCAGGGTG

TACAGGGGGGCCGCGAGCATGAGAAGACAGGTGTCTGTCATCCAGGGAATCAGGGTGTAC

AGGGGGGCCGCGAGCATGAGAAGACAGGTTTCGGTCATCCAGGGACTCAGGGTGGACACG

AGCATGAGAAGACAGGTGTCGGTCATCCAGGGACTCAGGGTGAGCAGGAGAAGAAGGGTA

TGATTGAGAAGATCAAGGAGAAGCTGCCAGGTCACAAGTAGGATGTGTGCCCACCATACA

CGTGGAGTCTACATCTATGTTTCTATGTTACTATAATACGCATGGAGTGTGTACGGATTC

TGTAATAATCTGCGCGTATCTGTTATGTC

>NK33

AGCCGTTGGATCTAGTTGAAAATGTCTGAGACGCGTGATGAGTATGGCAACCAGGTTCGC

CAAACCGACGAGTATGGGAACCCGATTCAGCATACTGGCACCGGGACAAAGGCCGGTGCG

GGCATACATGGTGGGGGCCATGGGATTGGCACAGGTGGTGGTGGTGGACAAGGCAAGCTC

CACCGTTCAGGCTCCGGGTCTTCCTCTGTAAGTATATTCGATGTTTCTCAAGGGTCATTT

CACTTGGTAGATCGTGTTGTGATGACTAGATTAGTTGATTAACCGTTGTGATTGTAAATC

AGTGATATGGGCTTCTGTGTACCTTATTTCACGATGATAATCAATTGATACGGTGGATCG

TGATGAGCGGACCGGATGAGCCGTCCATCTTACAGGCTTTTTAAAATAGAATTATAGGGT

GGGTGGTATGAGGTCCACCAAAGCCTGTAACATGGAATTACAATCATAGCCGTTAATCGG

TTCATCTCATCCGCTCATCGTGATCCAGCATGTCAAACAGAGATGGGTTTCTGGCGAAAT

GGATGTATGTAAGCCCACAAATCACAAAAATCTGACATTATGATGGAGAGAATTGCAAGG

AGTGTAAGTGAATCTACGCCGTTAGATGGATACTTCAAGGAGTGTAAGTGAATCTACGCC

GTTAGATGGATACTTTTTAACTTCGTTTTGTCAAATATTCTGTAGGATGAGGATGATGGA

CAAGGTGGGCGTAGGAAGAAGGGTTTGACGGAGAAGATCAAAGAGAAGCTGCCAGGTGGG

CAGAAGACGACGACAGGTGTGGGTTATCCAGGGAATCAGGGTGTACAGGGGGGCCGCGAG

CATGAGAAGACAGGTGTCTGTCATCCAGGGACTCAGGGTGTACAGGGGGGCCGCGAGCAT

GAGAAGACAGGTTTCGGTCATCCAGGGACTCAGGGTGGACACGAGCATGAGAAGACAGGT

GTCGGTCATCCAGGGACTCAGGGTGAGCAGGAGAAGAAGGGTATGATTGAGAAGATCAAG

GAGAAGCTGCCAGGTCACAAGTAGGATGTGTGCCCACCATACACGTGGAGTCTACATCTA

TGTTTCTATGTTACTATAATACGCATGGAGTGTGTACGGATTCTGTAATAATATGCGCGT

ATCTGTTATGTC

>NK35

AGCCGTTGGATCTAGTTGAAAATGTCTGAGACGCGTGATGAGTATGGCAACGAGGTTCGC

CAAACCGACGAGTATGGGAACCCGATTCAGCATACTGGCACCGGGACAAAGGGCGGTGCG

GGAATACATGGTGGGGGTCATGGGATTGGCACAGGTGGTGGTGGTGGACAAGGCAAGCTC

CACCGTTCAGGCTCCGGCTCTTCCTCTGTAAGTATATTAGATGTTTCTCAAGAGTCATTT

CACTTGGTAGATTGTGTTGGGATGACTAGATTAGTTGATTAACGGTTGCGATTGTAAATC

AGTGATATGGGCTTCTGTAGACCTTATTTCACGATGATAATCAATTGATACGGTGGATCG

TGATGAGCGGATCGGATGAGCCGTACATCTTACAGGCTTTTTAAAATAGAATTATAGGGT

GGGTGGTATGAGGTCCACCAAAGCCTGTAACATGGAATTACAATCATAGCCGTTAATCGG

TTCATCTCATCCGCTCATCGTGATCCAGCATGTCAAACGGAGATGGGTTACTGGCGAAAT

GGATGTATGTAAGCCCACAAATCACAAAAATCTGACATTATGATGGAGAGAATTGCAAGG

AGTGTAAGTGAATCTACGCCGTTAGATGGATACTTTTTAACTTCGTTTTGTCAAATATTC

TGTAGGATGAGGATGATGGACAAGGTGGGCGTAGGAAGAAGGGTTTGACGGAGAAGATCA

AAGAGAAGCTGCCAGGTGGGCAGAAGACGACGACAGGTGTGGGTTATCCAGGGAATCAGG

GTGTACAGGGGGGCCGCGAGCATGAGAAGACAGGTGTCTGTCATCCAGGGACTCAGGGTG

TACAGGGGGGCCGCGAGCATGAGAAGACAGGTTTCGGTCATCCAGGGACTCAGGGTGGAC

ACGAGCATGAGAAGACAGGTGTCGGTCATCCAGGGACTCAGGGTGAGCAGGAGAAGAAGG

GTATGATTGAGAAGATCAAGGAGAAGCTGCCAGGTCACAAGTAGGATGTGTGCCCACCAT

ACACGTGGAGTCTACATCTATGTTTCTATGTTACTATAATACGCATGGAGTGTGTACGGA

TTCTGTAATAATCTGCGCGTATCTGTTATGTC

>NK37

AGCCGTTGGATCTAGTTGAAAATGTCTGAGACGCGTGATGAGTATGGCAACGAGGTTCGC

CAAACCGACGAGTATGGGAACCCGATTCAGCATACTGGCACCGGGACAAAGGGCGGTGCG

GGAATACATGGTGGGGGTCATGGGATTGGCACAGGTGGTGGTGGTGGACAAGGCAAGCTC

CACCGTTCAGGCTCCGGCTCTTCCTCTGTAAGTATATTAGATGTTTCTCAAGAGTCATTT

CACTTGGTAGATTGTGTTGGGATGACTAGATTAGTTGATTAACGGTTGCGATTGTAAATC

AGTGATATGGGCTTCTGTAGACCTTATTTCACGATGATAATCAATTGATACGGTGGATCG

TGATGAGCGGATCGGATGAGCCGTACATCTTACAGGCTTTTTAAAATAGAATTATAGGGT

GGGTGGTATGAGGTCCACCAAAGCCTGTAACATGGAATTACAATCATAGCCGTTAATCGG

TTCATCTCATCCGCTCATCGTGATCCAGCATGTCAAACGGAGATGGGTTACTGGCGAAAT

GGATGTATGTAAGCCCACAAATCACAAAAATCTGACATTATGATGGAGAGAATTGCAAGG

AGTGTAAGTGAATCTACGCCGTTAGATGGATACTTTTTAACTTCGTTTTGTCAAATATTC

TGTAGGATGAGGATGATGGACAAGGTGGGCGTAGGAAGAAGGGTTTGACGGAGAAGATCA

AAGAGAAGCTGCCAGGTGGGCAGAAGACGACGACAGGTGTGGGTTATCCAGGGAATCAGG

GTGTACAGGGGGGCCGCGAGCATGAGAAGACAGGTGTCTGTCATCCAGGGACTCAGGGTG

TACAGGGGGGCCGCGAGCATGAGAAGACAGGTTTCGGTCATCCAGGGACTCAGGGTGGAC

ACGAGCATGAGAAGACAGGTGTCGGTCATCCAGGGACTCAGGGTGAGCAGGAGAAGAAGG

GTATGATTGAGAAGATCAAGGAGAAGCTGCCAGGTCACAAGTAGGATGTGTGCCCACCAT

ACACGTGGAGTCTACATCTATGTTTCTATGTTACTATAATACGCATGGAGTGTGTACGGA

TTCTGTAATAATCTGCGCGTATCTGTTATGTC

>NK45

AGCCGTTGGATCTAGTTGAAAATGTCTGAGACGCGTGATGAGTATGGCAACCAGGTTCGC

CAAACCGACGAGTATGGGAACCCGATTCAGCATACTGGCACCGGCACAAAGGCCGGTGCG

GGCATACATGCTGGGGGCCATGGGATTGGCACAGGTGGTGGTGGTGGACAAGGCAAGCTC

CACCGTTCAGGCTCCGGGTCTTCCTCTGTAAGTATATTCGATGTTTCTCAAGGGTCATTT

CACTTGGTAGATCGTGTTGTGATGACTAGATTAGTTGATTAACCGTTGTGATTGTAAATC

AGTGATATGGGCTTCTGTGTTCCTTATTTCACAATGATAATCAATTGATACGGTGGATCG

TGATGAGCGGACCGAATGAGCCGTCCATCTTACAGGCTTTTTAAAATAGAATTATAGGGT

GGGTGGTATGAGGTCCACCAAAGCCTGTAACATGGAATTACAATTATAGCCGTTAATCGG

TTCATCTCATCCGCTCATCGTGATCCAGCATGTCAAACAGAGATGGGTTTCTGGCGAAAT

GGATGTATGTAAGCCCACAAATCACAAAAATCTGACATTATGATGGAGAGAATTGCAAGG

AGTGTAAGTGAATCTACGCCGTTAGATGGATACTTTTTAACTTCGTTTTGTCAAATATTC

TGTAGGATGAGGATGATGGACAAGGTGGGCGTAGGAAGAAGGGTTTGACGGAGAAGATCA

AAGAGAAGCTGCCAGGTGGGCAGAAGACGACAGGTGTCGGTTATCCAGGGAATCAGGGTG

TACAGGGGGGCCGCGAGCATGAGAAGACAGGTGTCTGTCATCCAGGGACTCAGGGTGTAC

AGGGGGGCCGCGAGCATGAGAAGACAGGTTTCGGTCATCCAGGGACTCAGGGTGGACACG

AGCATGAGAAGACAGGTGTCGGTCATCCAGGGACTCAGGGTGAGCAGGAGAAGAAGGGTA

TGATTGAGAAGATCAAGGAGAAGCTGCCAGGTCACAAGTAGGATGTGTGCCCACCATACA

CGTGGAGTCTACATCTATGTTTCTATGTTACTATAATACGCATGGAGTGTGTACGGATTC

TGTAATAATCTGCGCGTATCTGTTATGTC

>NK43

AGCCGTTGGATCTAGTTGAAAATGTCTGAGACGCGTGATGAGTATGGCAACCAGGTTCGC

CAAACCGACGAGTATGGGAACCCGATTCAGCATACTGGCACCGGGACAAAGGGCGGTGCG

GGAATACATGGTGGGGGTCATGGGATTGGCACAGGTGGTGGTGGTGGACAAGGCAAGCTC

CACCGTTCAGGCTCCGGCTCTTCCTCTGTAAGTATATTAGATGTTTCTCAAGAGTCATTT

CACTTGGTAGATTGTGTTGGGATGACTAGATTAGTTGATTAACGGTTGCGATTGTAAATC

AGTGATATGGGCTTCTGTCTACCTTATTTCATGATGATAATCAATTGATACGGTGGATCG

TGATGAGCGGATCGGATGAGCCGTACATCTTACAGGCTTTTTAAAATAGAATTATAGGGT

GGGTGGTATGAGGTCCACCAAAGCCTGTAACATGGAATTACAATCATAGCCGTTAATAGG

TTCATCTCATCCGCTCATCGTGATCCAGCATGTCAAACAGAGATGGGTTTCTGGCGAAAT

GGATGTATGTAAGCCCACAAATCACAAAAATCTGACATTATGATGGAGAGAATTGCAAGG

AGTGCAAGTGAATCTACGCCGTTAGATGGATACTTTTTAACTTCGTTTTGTCAAATATTC

CGTAGGATGAGGATGATGGACAAGGTGGGCGTAGGAAGAAGGGTTTGACGGAGAAGATCA

AAGAGAAGCTGCCAGGTGGGCAGAAGACGACAGGTGTCGGTTATCCAGGGAATCAGGGTG

TACAGGGGGGCCGCGAGCATGAGAAGACAGGTGTCTGTCATCCAGGGACTCAGGGTGTAC

AGGGGGGCCGCGAGCATGAGAAGACAGGTTTCGGTCATCCAGGGACTCAGGGTGGACACG

AGCATGAGAAGATAGGTGTCGGTCATCCAGGGACTCAGGGTGAGCAGGAGAAGAAGGGTA

TGATTGAGAAGATCAAGGAGAAGCTGCCAGGTCACAAGTAGGATGTGTGCCCACCATACA

CGTGGAGTCTACATCTATGTTTCTATGTTACTATAATACGCATGGAGTGTGTACGGATTA

TGTAATAATCTGCGCGTATCTGTTATGTC

>NK46

AGCCGTTGGATCTAGTTGAAAATGTCTGAGACGCGTGATGAGTATGGCAACCAGGTTCGC

CAAACCGACGAGTATGGGAACCCGATTCAGCATACTGGCACCGGGACAAAGGGCGGTGCG

GGAATACATGGTGGGGGTCATGGGATTGGCACAGGTGGTGGTGGTGGACAAGGCAAGCTC

CACCGTTCAGGCTCCGGCTCTTCCTCTGTAAGTATATTAGATGTTTCTCAAGAGTCATTT

CACTTGGTAGATTGTGTTGGGATGACTAGATTAGTTGATTAACGGTTGCGATTGTAAATC

AGTGATATGGGCTTCTGTCTACCTTATTTCATGATGATAATCAATTGATACGGTGGATCG

TGATGAGCGGATCGGATGAGCCGTACATCTTACAGGCTTTTTAAAATAGAATTATAGGGT

GGGTGGTATGAGGTCCACCAAAGCCTGTAACATGGAATTACAATCATAGCCGTTAATAGG

TTCATCTCATCCGCTCATCGTGATCCAGCATGTCAAACAGAGATGGGTTTCTGGCGAAAT

GGATGTATGTAAGCCCACAAATCACAAAAATCTGACATTATGATGGAGAGAATTGCAAGG

AGTGCAAGTGAATCTACGCCGTTAGATGGATACTTTTTAACTTCGTTTTGTCAAATATTC

CGTAGGATGAGGATGATGGACAAGGTGGGCGTAGGAAGAAGGGTTTGACGGAGAAGATCA

AAGAGAAGCTGCCAGGTGGGCAGAAGACGACAGGTGTCGGTTATCCAGGGAATCAGGGTG

TACAGGGGGGCCGCGAGCATGAGAAGACAGGTGTCTGTCATCCAGGGACTCAGGGTGTAC

AGGGGGGCCGCGAGCATGAGAAGACAGGTTTCGGTCATCCAGGGACTCAGGGTGGACACG

AGCATGAGAAGATAGGTGTCGGTCATCCAGGGACTCAGGGTGAGCAGGAGAAGAAGGGTA

TGATTGAGAAGATCAAGGAGAAGCTGCCAGGTCACAAGTAGGATGTGTGCCCACCATACA

CGTGGAGTCTACATCTATGTTTCTATGTTACTATAATACGCATGGAGTGTGTACGGATTA

TGTAATAATCTGCGCGTATCTGTTATGTC

>NK412

AGCCGTTGGATCTAGTTGAAAATGTCAGTGACGCGTGATGAGTATGGCAACCAGGTTCGG

CAGACCGACGAGTATGGGAACCCGATTCAGCATACTGGCACCGGGACAAAGGCCGGTTCG

GGCATACATGGTGGGGGCCATGGGATTGGCACAGGTGGTGGTGGTGGTGGACAAGGCAAG

CTCCACCGTTCAGGCTCTGGCTCTTCCTCTGTAAGTACATTCGATGTTTCTCAAGGGTCA

TTTCACTTGGTATATCGTGTTGGGATGATTAGATTAGTTGATTAACGGTTGTGATTGTAA

ATCAGTGATATGGGCTTCTGTGTACCTTAGTTCACGATGATAATCAATTGATACGCTGGA

TCGTGATGAGCGGACCGGATGAGCCGTCCATCTTACAGGCTTTTTAAAATAGAATTATAG

GGTGGGTGGTATGAGGTCCACCAAAGCCTGTAACATGGAATTACAATCATAGCCGTTAAT

CGGTTCATCTCATCCGCTCATCGTGATCTAGCATGTAAAACAGAGATGGGTTTCTGGAGA

AATGGATGCATGTAAGCCCACAAATCACAAAAATCTGACATTATGATGGAGAGAATGCAA

GGAGTGTAAGCGAATTTACGCCGTTAGATGGATTTCATTGTACTTTTAACTTTGTTTTGT

CGAATATTTTGTAGGATGAGGATGATGGACAAGGTGGGCGTAGGAAGAAGGGTTTGACGG

AGAAGATCAAAGAGAAGCTGCCAGGTGGAAACAAGACGACAGGTGTCTGTCATCCAGGGA

CTCAGGGTGTACAGGGGGGCCGCGAGCATGAGAAGACAGGTTGCGGTCAGGGTGGTCAGG

GTGGACAGGTGGGCCGCGAGCATGAGAAGACAGGTTTCGGCCATGCAGTGACTCAGGGTG

GACAGGTGGGCAACGAGCAGGAGAAGAAGGGTATGATTGAGAAGATCAAGGAGAAGCTGC

CAGGTCACAAGTAGGATGTGTGCCCACCATACACGTGGAGTCTACATCTACGTGCCTATT

ATGTTACTATAATACGCATGGAGTGTGTCCGGAGTCTGTAATAATCTGCGCGTATCTGTT

ATGTC

>NK451

AGCCGTTGGATCTAGTTGAAAATGTCTGAGACGCGTGATGAGTATGGCAACCAGGTTCGC

CAAACCGACGAGTATGGGAACCCGATTCAGCATACTGGCACCGGGACAAAGGGCGGTGCG

GGAATACATGGTGGGGGTCATGGGATTGGCACAGGTGGTGGTGGTGGACAAGGCAAGCTC

CACCGTTCAGGCTCCGGCTCTTCCTCTGTAAGTATATTAGATGTTTCTCAAGAGTCATTT

CACTTGGTAGATTGTGTTGGGATGACTAGATTAGTTGATTAACGGTTGCGATTGTAAATC

AGTGATATGGGCTTCTGTCTACCTTATTTCATGATGATAATCAATTGATACGGTGGATCG

TGATGAGCGGATCGGATGAGCCGTACATCTTACAGGCTTTTTAAAATAGAATTATAGGGT

GGGTGGTATGAGGTCCACCAAAGCCTGTAACATGGAATTACAATCATAGCCGTTAATAGG

TTCATCTCATCCGCTCATCGTGATCCAGCATGTCAAACAGAGATGGGTTTCTGGCGAAAT

GGATGTATGTAAGCCCACAAATCACAAAAATCTGACATTATGATGGAGAGAATTGCAAGG

AGTGCAAGTGAATCTACGCCGTTAGATGGATACTTTTTAACTTCGTTTTGTCAAATATTC

CGTAGGATGAGGATGATGGACAAGGTGGGCGTAGGAAGAAGGGTTTGACGGAGAAGATCA

AAGAGAAGCTGCCAGGTGGGCAGAAGACGACAGGTGTCGGTTATCCAGGGAATCAGGGTG

TACAGGGGGGCCGCGAGCATGAGAAGACAGGTGTCTGTCATCCAGGGACTCAGGGTGTAC

AGGGGGGCCGCGAGCATGAGAAGACAGGTTTCGGTCATCCAGGGACTCAGGGTGGACACG

AGCATGAGAAGATAGGTGTCGGTCATCCAGGGACTCAGGGTGAGCAGGAGAAGAAGGGTA

TGATTGAGAAGATCAAGGAGAAGCTGCCAGGTCACAAGTAGGATGTGTGCCCACCATACA

CGTGGAGTCTACATCTATGTTTCTATGTTACTATAATACGCATGGAGTGTGTACGGATTA

TGTAATAATCTGCGCGTATCTGTTATGTC

>NK55

AGCCGTTGGATCTAGTTGAAAATGTCTGAGACGCGTGATGAGTATGGCAACCAGGTTCGC

CAAACCGACGAGTATGGGAACCCGATTCAGCATACTGGCACCGGGACAAAGGGCGGTGCG

GGAATACATGGTGGGGGTCATGGGATTGGCACAGGTGGTGGTGGTGGACAAGGCAAGCTC

CACCGTTCAGGCTCCGGCTCTTCCTCTGTAAGTATATTAGATGTTTCTCAAGAGTCATTT

CACTTGGTAGATTGTGTTGGGATGACTAGATTAGTTGATTAACGGTTGCGATTGTAAATC

AGTGATATGGGCTTCTGTCTACCTTATTTCATGATGATAATCAATTGATACGGTGGATCG

TGATGAGCGGATCGGATGAGCCGTACATCTTACAGGCTTTTTAAAATAGAATTATAGGGT

GGGTGGTATGAGGTCCACCAAAGCCTGTAACATGGAATTACAATCATAGCCGTTAATAGG

TTCATCTCATCCGCTCATCGTGATCCAGCATGTCAAACAGAGATGGGTTTCTGGCGAAAT

GGATGTATGTAAGCCCACAAATCACAAAAATCTGACATTATGATGGAGAGAATTGCAAGG

AGTGCAAGTGAATCTACGCCGTTAGATGGATACTTTTTAACTTCGTTTTGTCAAATATTC

CGTAGGATGAGGATGATGGACAAGGTGGGCGTAGGAAGAAGGGTTTGACGGAGAAGATCA

AAGAGAAGCTGCCAGGTGGGCAGAAGACGACAGGTGTCGGTTATCCAGGGAATCAGGGTG

TACAGGGGGGCCGCGAGCATGAGAAGACAGGTGTCTGTCATCCAGGGACTCAGGGTGTAC

AGGGGGGCCGCGAGCATGAGAAGACAGGTTTCGGTCATCCAGGGACTCAGGGTGGACACG

AGCATGAGAAGATAGGTGTCGGTCATCCAGGGACTCAGGGTGAGCAGGAGAAGAAGGGTA

TGATTGAGAAGATCAAGGAGAAGCTGCCAGGTCACAAGTAGGATGTGTGCCCACCATACA

CGTGGAGTCTACATCTATGTTTCTATGTTACTATAATACGCATGGAGTGTGTACGGATTC

TGTAATAATCTGCGCGTATCTGTTATGTC

>NK52

AGCCGTTGGATCTAGTTGAAAATGTCTGAGACGCGTGATGAGTATGGCAACCAGGTTCGC

CAAACCGACGAGTATGGGAACCCGATTCAGCATACTGGCACCGGGACAAAGGGCGGTGCG

GGAATACATGGTGGGGGTCATGGGATTGGCACAGGTGGTGGTGGTGGACAAGGCAAGCTC

CACCGTTCAGGCTCCGGCTCTTCCTCTGTAAGTATATTAGATGTTTCTCAAGAGTCATTT

CACTTGGTAGATTGTGTTGGGATGACTAGATTAGTTGATTAACGGTTGCGATTGTAAATC

AGTGATATGGGCTTCTGTCTACCTTATTTCACGATGATAATCAATTGATACGGTGGATCG

TGATGAGCGGATCGGATGAGCCGTACATCTTACAGGCTTTTTAAAATAGAATTATAGGGT

TGGTGGTATGAGGTCCACCAAAGCCTGTAACATGGAATTACAATCATAGCCGTTAATCGG

TTCATCTCATCCGCTCATCGTGATCCAGCATGTCAAACGGAGATGGGTTACTGGCGAAAT

GGATGTATGTAAGCCCACAAATCACAAAAATCTGACATTATGATGGAGAGAATTGCAAGG

AGTGTAAGTGAATCTACGCCGTTAGATGGATACTTTTTAACTTCGTTTTGTCAAATATTC

TGTAGGATGAGGATGATGGACAAGGTGGGCGTAGGAAGAAGGGTTTGACGGAGAAGATCA

AAGAGAAGCTGCCAGGTGGGCAGAAGACGACAGGTGTCGGTTATCCAGGGAATCAGGGTG

TACAGGGGGGCCGCGAGCATGAGAAGACAGGTGTCTGTCATCCAGGGACTCAGGGCGTAC

AGGGGGGCCGCGAGCATGAGAAGACAGGTTTCGGTCATCCAGGGACTCAGGGTGGACACG

AGCATGAGAAGACAGGTGTCGGTCATCCAGGGACTCAGGGTGAGCAGGAGAAGAAGGGTA

TGATTGAGAAGATCAAGGAGAAGCTGCCAGGTCACAAGTAGGATGTGTGCCCACCATACA

CGTGGAGTCTACATCTATGTTTCTATGTTACTATAATACGCATGGAGTGTGTACGGATTC

TGTAATAATCTGCGCGTATCTGTTATGTC

>NK58

AGCCGTTGGATCTAGTTGAAAATGTCTGAGACGCGTGATGAGTATGGCAACCAGGTTCGC

CAAACCGACGAGTATGGGAACCCGATTCAGCATACTGGCACCGGGACAAAGGGCGGTGCG

GGAATACATGGTGGGGGTCATGGGATTGGCACAGGTGGTGGTGGTGGACAAGGCAAGCTC

CACCGTTCAGGCTCCGGCTCTTCCTCTGTAAGTATATTAGATGTTTCTCAAGAGTCATTT

CACTTGGTAGATTGTGTTGGGATGACTAGATTAGTTGATTAACGGTTGCGATTGTAAATC

AGTGATATGGGCTTCTGTCTACCTTATTTCATGATGATAATCAATTGATACGGTGGATCG

TGATGAGCGGATCGGATGAGCCGTACATCTTACAGGCTTTTTAAAATAGAATTATAGGGT

GGGTGGTATGAGGTCCACCAAAGCCTGTAACATGGAATTACAATCATAGCCGTTAATAGG

TTCATCTCATCCGCTCATCGTGATCCAGCATGTCAAACAGAGATGGGTTTCTGGCGAAAT

GGATGTATGTAAGCCCACAAATCACAAAAATCTGACATTATGATGGAGAGAATTGCAAGG

AGTGCAAGTGAATCTACGCCGTTAGATGGATACTTTTTAACTTCGTTTTGTCAAATATTC

CGTAGGATGAGGATGATGGACAAGGTGGGCGTAGGAAGAAGGGTTTGACGGAGAAGATCA

AAGAGAAGCTGCCAGGTGGGCAGAAGACGACAGGTGTCGGTTATCCAGGGAATCAGGGTG

TACAGGGGGGCCGCGAGCATGAGAAGACAGGTGTCTGTCATCCAGGGACTCAGGGTGTAC

AGGGGGGCCGCGAGCATGAGAAGACAGGTTTCGGTCATCCAGGGACTCAGGGTGGACACG

AGCATGAGAAGATAGGTGTCGGTCATCCAGGGACTCAGGGTGAGCAGGAGAAGAAGGGTA

TGATTGAGAAGATCAAGGAGAAGCTGCCAGGTCACAAGTAGGATGTGTGCCCACCATACA

CGTGGAGTCTACATCTATGTTTCTATGTTACTATAATACGCATGGAGTGTGTACGGATTA

TGTAATAATCTGCGCGTATCTGTTATGTC

>NK59

AGCCGTTGGATCTAGTTGAAAATGTCTGAGACGCGTGATGAGTATGGCAACCAGGTTCGC

CAAACCGACGAGTATGGGAACCCGATTCAGCATACTGGCACCGGGACAAAGGGCGGTGCG

GGAATACATGGTGGGGGTCATGGGATTGGCACAGGTGGTGGTGGTGGACAAGGCAAGCTC

CACCGTTCAGGCTCCGGCTCTTCCTCTGTAAGTATATTAGATGTTTCTCAAGAGTCATTT

CACTTGGTAGATTGTGTTGGGATGACTAGATTAGTTGATTAACGGTTGCGATTGTAAATC

AGTGATATGGGCTTCTGTCTACCTTATTTCACGATGATAATCAATTGATACGGTGGATCG

TGATGAGCGGATCGGATGAGCCGTACATCTTACAGGCTTTTTAAAATAGAATTATAGGGT

TGGTGGTATGAGGTCCACCAAAGCCTGTAACATGGAATTACAATCATAGCCGTTAATCGG

TTCATCTCATCCGCTCATCGTGATCCAGCATGTCAAACGGAGATGGGTTACTGGCGAAAT

GGATGTATGTAAGCCCACAAATCACAAAAATCTGACATTATGATGGAGAGAATTGCAAGG

AGTGTAAGTGAATCTACGCCGTTAGATGGATACTTTTTAACTTCGTTTTGTCAAATATTC

TGTAGGATGAGGATGATGGACAAGGTGGGCGTAGGAAGAAGGGTTTGACGGAGAAGATCA

AAGAGAAGCTGCCAGGTGGGCAGAAGACGACAGGTGTCGGTTATCCAGGGAATCAGGGTG

TACAGGGGGGCCGCGAGCATGAGAAGACAGGTGTCTGTCATCCAGGGACTCAGGGCGTAC

AGGGGGGCCGCGAGCATGAGAAGACAGGTTTCGGTCATCCAGGGACTCAGGGTGGACACG

AGCATGAGAAGACAGGTGTCGGTCATCCAGGGACTCAGGGTGAGCAGGAGAAGAAGGGTA

TGATTGAGAAGATCAAGGAGAAGCTGCCAGGTCACAAGTAGGATGTGTGCCCACCATACA

CGTGGAGTCTACATCTATGTTTCTATGTTACTATAATACGCATGGAGTGTGTACGGATTC

TGTAATAATCTGCGCGTATCTGTTATGTC

>NK66

AGCCGTTGGATCTAGTTGAAAATGTCTGAGACGCGTGATGAGTATGGCAACGTGGTTCGC

CAAACCGACGAGTATGGGAACCCGATTCAGCATACTGGCACCGGGACAAAGGGCGGTGCG

GGAATACATGGTGGGGGTCATGGGATTGGCACAGGTGGTGGTGGTGGACAAGGCAAGCTC

CACCGTTCAGGCTCCGGCTCTTCCTCTGTAAGTATATTAGATGTTTCTCAAGAGTCATTT

CACTTGGTAGATTGTGTTGGGATGACTAGATTAGTTGATTAACGGTTGCGATTGTAAATC

AGTGATATGGGCTTCTGTAGACCTTATTTCACGATGATAATCAATTGATACGGTGGATCG

TGATGAGCGGATCGGATGAGCCGTACATCTTACAGGCTTTTTAAAATAGAATTATAGGGT

GGGTGGTATGAGGTCCACCAAAGCCTGTAACATGGAATTACAATCATAGCCGTTAATCGG

TTCATCTCATCCGCTCATCGTGATCCAGCATGTCAAACGGAGATGGGTTACTGGCGAAAT

GGATGTATGTAAGCCCACAAATCACAAAAATGTGACATTATGATGGAGAGAATTGCAAGG

AGTGTAAGTGAATCTACGCCGTTAGATGGATACTTTTTAACTTCGTTTTGTCAAATATTC

TGTAGGATGAGGATGATGGACAAGGTGGGCGTAGGAAGAAGGGTTTGACGGAGAAGATCA

AAGAGAAGCTGCCAGGTGGGCAGAAGACGACGACAGGTGTGGGTTATCCAGGGACTCAGG

GTGTACAGGGGGGCCGCGAGCATGAGAAGACAGGTGTCTGTCATCCAGGGACTCAGGGTG

TACAGGGGGGCCGCGAGCATGAGAAGACAGGTTTCGGCCATGCAGTGACTCAGGGTGGAC

AGGTGGGCAACGAGCAGGAGAAGAAGGGTATGATTGAGAAGATCCAGGAGAAGCTGCCAG

GTCACAAGTAGGATGTGTGCCCACCATACACGTGGAGTCTACATCTACGTGCCTATTATG

TTACTATAATACGCATGGAGTGTGTCCGGAGTCTGTAATAATCTGCGCGTATCTGTTATG

TC

>NK613

AGCCGTTGGATCTAGTTGAAAATGTCAGTGACGCGTGATGAGTATGGCAACCAGGTTCGC

CAGACCGACGAGTATGGGAACCCGATTCAGCATACTGGCACCGGGACAAAGGCCGGTTCG

GGCATACATGGTGGGGGCCATGGGATTGGCACAGGTGGTGGTGGTGGTGGACAAGGCAAG

CTCCACCGTTCAGGCTCTGGCTCTTCCTCTGTAAGTACATTCGATGTTTCTCAAGGGTCA

TTTCACTTGGTATATCGTGTTGGGATGATTAGATTAGTTGATTAACGGTTGTGATTGTAA

ATCAGTGATATGGGCATCTGTGTACCTTAGTTCACGATGATAATCAATTGATACGCTGGA

TCGTGATGAGCGGACCGGATGAGCCGTCCATCTTACAGGCTTTTTAAAATAGAATTATAG

GGTGGGTGGTATGAGGTCCACCAAAGCCTGTAACATGGAATTACAATCATAGCCGTTAAT

CGGTTCATCTCATCCGCTCATCGTGATCTAGCATGTCAAACAGAGATGGGTTTCTGGAGA

AATGGATGTATGTAAGCCCACAAATCACAAAAATCTGACATTATGATGGAGAGAATGCAA

GGAGTGTAAGCGAATTTACGCCGTTAGATGGATTTCATTGTACTTTTAACTTTGTTTTGT

CGAATATTTTGTAGGATGAGGATGATGGACAAGGTGGGCGTAGGAAGAAGGGTTTGACGG

AGAAGATCAAAGAGAAGCTGCCAGGTGGAAACAAGACGACAGGTGTCTGTCATCCAGGGA

CTCAGGGTGTACAGGGGGGCCGCGAGCATGAGAAGACAGGTTGCGGTCAGGGTGGTCAGG

GTGGACAGGTGGGCCGCGAGCATGAGAAGACAGGTTTCGGCCATGCAGTGACTCAGGGTG

GACAGGTGGGCAACGAGCAGGAGAAGAAGGGTATGATTGAGAAGATCCAGGAGAAGCTGC

CAGGTCACAAGTAGGATGTGTGCCCACCATACACGTGGAGTCTACATCTACGTGCCTATT

ATGTTACTATAATACGCATGGAGTGTGTCCGGAGTCTGTAATAATCTGCGCGTATCTGTT

ATGTC

>NK615

AGCCGTTGGATCTAGTTGAAAATGTCTGAGACGCGTGATGAGTATGGCAACCAGGTTAGC

CAAACCGACGAGTATGGGAACCCGATTCAGCATACTGGCACCGGGACAAAGGGCGGTGCG

GGAATACATGGTGGGGGTCATGGGATTGGCACAGGTGGTGGTGGTGGACAAGGCAAGCTC

CACCGTTCAGGCTCCGGCTCTTCCTCTGTAAGTATATTAGATGTTTCTCAAGAGTCATTT

CACTTGGTAGATTGTGTTGGGATGACTAGATTAGTTGATTAACGGTTGCGATTGTAAATC

AGTGATATGGGCTTCTGTAGACCTTATTTCACGATGATAATCAATTGATACGGTGGATCG

TGATGAGCGGATCGGATGAGCCGTACATCTTACAGGCTTTTTAAAATAGAATTATAGGGT

GGGTGGTATGAGGTCCACCAAAGCCTGTAACATGGAATTACAATCATAGCCGTTAATCGG

TTCATCTCATCCGCTCATCGTGATCCAGCATGTCAAACGGAGATGGGTTACTGGCGAAAT

GGATGTATGTAAGCCCACAAATCACAAAAATGTGACATTATGATGGAGAGAATTGCAAGG

AGTGTAAGTGAATCTACGCCGTTAGATGGATACTTTTTAACTTCGTTTTGTCAAATATTC

TGTAGGATGAGGATGATGGACAAGGTGGGCGTAGGAAGAAGGGTTTGACGGAGAAGATCA

AAGAGAAGCTGCCAGGTGGGCAGAAGACGACGACAGGTGTGGGTTATCCAGGGACTCAGG

GTGTACAGGGGGGCCGCGAGCATGAGAAGACAGGTGTCTGTCATCCAGGGACTCAGGGTG

TACAGGGGGGCCGCGAGCATGAGAAGACAGGTTTCGGTCATCCAGGGACTCAGGGTGGAC

ACGAGCATGAGAAGACAGGTGTCGGTCATCCAGGGACTCAGGGTGAGCAGGAGAAGAAGG

GTATGATTGAGAAGATCAAGGAGAAGCTGCCTGGTCACAAGTAGGATGTGTGCCCACCAT

ACACGTGGAGTCTACATCTATGTTTCTATGTTACTATAATACGCATGGAGTGTGTACGGA

TTCTGTAATAATCTGCGCGTATCTGTTATGTC

>LYS11

AGCCGTTGGATCTAGTTGAAAATGTCAGTGACGCGTGATGAGTATGGCAACCAGGTTCGC

CAGACCGACGAGTATGGGAACCCGATTCAGCATACTGGCACCGGGACAAAGGCCGGTTCG

GGCATACATGGTGGGAGCCATGGGATTGGCACAGGTGGTGGTGGTGGTGGTGGTGGACAA

GGCAAGCTCCACCGTTCAGGCTCTGGCTCTTCCTCTGTAAGTACATTCGATGTTTCTCAA

GGGTCATTTCACTTGGTATATCGTGTTGGGATGATTAGATTAGTTGATTAACGGTTGTGA

TTGTAAATCAGTGATATGGGCTTCTGTGTACCTTAGTTCACGATGATATTCAATTGATAC

GCTGGATCGTGATGAGCGGACCGGATGAGCCGTCCATCTTACAGGCTTTTTAAAATAGAA

TTATAGGGTGGGTGGTATGAGGTCCACCAAAGCCTGTAACATGGAATTACAATCATAGCC

GTTAATCGGTTCATCTCATCCGCTCATCGTGATCTAGCATGTCAAACAGAGATGGGTTTC

TGGAGAAATGGATGTATGTAAGCCCACAAATCACAAAAATCTGACATTATGATGGAGAGA

ATGCAAGGAGTGTAAGCGAATTTACGCCGTTAGATGGATTTCATTGTACTTGAACTTTGT

TTTGTCAAATATTTTGTAGGATGAGGATGATGGACAAGGTGGGCGTAGGAAGAAGGGTTT

GACGGAGAAGATCAAAGAGAAGCTGCCAGGTGGAAACAAGACGACAGGTGTCTGTCATCC

AGGGACTCAGGGTGTACAGGGGGGCCGCGAGCATGAGAAGACAGGTTGCGGTCAGGGTGG

TCAGGGTGGACAGGTGGGCCGCGAGCATGAGAAGACAGGTTTCGGCCATCCAGTGACTCA

GGGTGGACAGGTGGGCAACGAGCAGGAGAAGAAGGGTATGATTGAGAAGATCCAGGAGAA

GCTGCCAGGTCACAAGTAGGATGTGTGCCCACCATACACGTGGAGTCTACATCTACGTGC

ATATTATGTTACTATAATACGCATGGAGTGTGTCCGGAGTCTGTAATAATCTGCGCGTAT

CTGTTATGTC

>LYS12

AGCCGTTGGATCTAGTTGAAAATGTCTGAGACGCGTGATGAGTATGGCAACCAGGTTAGC

CAAACCGACGAGTATGGGAACCCGATTCAGCATACTGGCACCGGGACAAAGGGCGGTGCG

GGAATACATGGTGGGGGTCATGGGATTGGCACAGGTGGTGGTGGTGGACAAGGCAAGCTC

CACCGTTCAGGCTCCGGCTCTTCCTCTGTAAGTATATTAGATGTTTCTCAAGAGTCATTT

CACTTGGTAGATTGTGTTGGGATGACTAGATTAGTTGATTAACGGTTGCGATTGTAAATC

AGTGATATGGGCTTCTGTCTACCTTATTTCACGATGATAATCAATTGATACGGTGGATCG

TGATGAGCGGATCGGATGAGCCGTACATCTTACAGGCTTTTTAAAATAGAATTATAGGGT

TGGTGGTATGAGGTCCACCAAAGCCTGTAACATGGAATTACAATCATAGCCGTTAATCGG

TTCATCTCATCCGCTCATCGTGATCCAGCATGTCAAACGGAGATGGGTTACTGGCGAAAT

GGATGTATGTAAGCCCACAAATCACAAAAATCTGACATTATGATGGAGAGAATTGCAAGG

AGTGTAAGTGAATCTACGCCGTTAGATGGATACTTTTTAACTTCGTTTTGTCAAATATTC

TGTAGGATGAGGATGATGGACAAGGTGGGCGTAGGAAGAAGGGTTTGACGGAGAAGATCA

AAGAGAAGCTGCCAGGTGGGCAGAAGACGACAGGTGTCGGTTATCCAGGGAATCAGGGTG

TACAGGGGGGCCGCGAGCATGAGAAGACAGGTGTCTGTCATCCAGGGACTCAGGGCGTAC

AGGGGGGCCGCGAGCATGAGAAGACAGGTTTCGGTCATCCAGGGACTCAGGGTGGACACG

AGCATGAGAAGACAGGTGTCGGTCATCCAGGGACTCAGGGTGAGCAGGAGAAGAAGGGTA

TGATTGAGAAGATCAAGGAGAAGCTGCCAGGTCACAAGTAGGATGTGTGCCCACCATACA

CGTGGAGTCTACATCTATGTTTCTATGTTACTATAATACGCATGGAGTGTGTACGGATTC

TGTAATAATCTGCGCGTATCTGTTATGTC

>LYS16

AGCCGTTGGATCTAGTTGAAAATGTCAGTGACGCGTGATGAGTATGGCAACCAGGTTCGC

CAGACCGACGAGTATGGGAACCCGATTCAGCATACTGGCACCGGGACAAAGGCCGGTTCG

GGCATACATGGTGGGGGCCATGGGATTGGCACAGGTGGTGGTGGTGGTGGACAAGGCAAG

CTCCACCGTTCAGGCTCTGGCTCTTCCTCTGTAAGTACATTCGATGTTTCTCAAGGGTCA

TTTCACTTGGTATATCGTGTTGGGATGATTAGATTAGTTGATTAACGGTTGTGATTGTAA

ATCAGTGATATGGGCTTCTGTGTACCTTAGTTCACGATGATAATCAATTGATACGCTGGA

TCGTGATGAGCGGACCGGATGAGCCGTCCATCTTACAGGCTTTTTAAAATAGAATTATAG

GGTGGGTGGTATGAGGTCCACCAAAGCCTGTAACATGGAATTACAATCATAGCCGTTAAT

CGGTTCATCTCATCCGCTCATCGTGATCTAGCATGTCAAACAGAGATGGGTTTCTGGAGA

AATGGATGTATGTAAGCCCACAAATCACAAAAATCTGACATTATGATGGAGAGAATGCAA

GGAGTGTAAGCGAATTTACGCCGTTAGATGGATTTCATTGTACTTTTAACTTTGTTTTGT

CGAATATTTTGTAGGATGAGGATGATGGACAAGGTGGGCGTAGGAAGAAGGGTTTGACGG

AGAAGATCAAAGAGAAGCTGCCAGGTGGAAACAAGACGACAGGTGTCTGTCATCCAGGGA

CTCAGGGTGTACAGGGGGGCCGCGAGCATGAGAAGACAGGTTGCGGTCAGGGTGGTCAGG

GTGGACAGGTGGGCCGCGAGCATGAGAAGACAGGTTTCGGCCATGCAGTGACTCAGGGTG

GACAGGTGGGCAACGAGCAGGAGAAGAAGGGTATGATTGAGAAGATCCAGGAGAAGCTGC

CAGGTCACAAGTAGGATGTGTGCCCACCATACACGTGGAGTCTACATCTACGTGCCTATT

ATGTTACTATAATACGCATGGAGTGTGTCCGGAGTCTGTAATAATCTGCGCGTATCTGTT

ATGTC

>LYS161

AGCCGTTGGATCTAGTTGAAAATGTCTGAGACGCGTGATGAGTATGGCAACCAGGTTAGC

CAAACCGACGAGTATGGGAACCCGATTCAGCATACTGGCACCGGCACAAAGGCCGGTGCG

GGCATACATGCTGGGGGCCATGGGATTGGCACAGGTGGTGGTGGTGGACAAGGCAAGCTC

CACCGTTCAGGCTCCGGGTCTTCCTCTGTAAGTATATTCGATGTTTCTCAAGGGTCATTT

CACTTGGTAGATCGTGTTGTGATGACTAGATTAGTTGATTAACTGTTGTGATTGTAAATC

AGTGATATGGGCTTCTGTGTTCCTTATTTCACAATGATAATCAATTGATACGGTGGATCG

TGATGAGCGGACCGAATGAGCCGTCCATCTTACAGGCTTTTTAAAATAGAATTATAGGGT

GGGTGGTATGAGGTCCACCAAAGCCTGTAACATGGAATTACAATTATAGCCGTTAATCGG

TTCATCTCATCCGCTCATCGTGATCCAGCATGTCAAACAGAGATGGGTTTCTGGCGAAAT

GGATGTATGTAAGCCCACAAATCACAAAAATCTGACATTATGATGGAGAGAATTGCCAGG

AGTGTAAGTGAATCTACGCCGTTAGATGGATACTTTTTAACTTCGTTTTGTCAAATATTC

TGTAGGATGAGGATGATGGACAAGGTGGGCGTAGGAAGAAGGGTTTGACGGAGAAGATCA

AAGAGAAGCTGCCAGGTGGGCAGAAGACGACAGGTGTCGGTTATCCAGGGAATCAGGGTG

TACAGGGGGGCCGCGAGCATGAGAAGACAGGTGTCTGTCATCCAGGGACTCAGGGTGTAC

AGGGGGGCCGCGAGCATGAGAAGACAGGTTTCGGTCATCCAGGGACTCAGGGTGGACACG

AGCATGAGAAGACAGGTGTCGGTCATCCAGGGACTCAGGGTGAGCAGGAGAAGAAGGGTA

TGATTGAGAAGATCAAGGAGAAGCTGCCAGGTCACAAGTAGGATGTGTGCCCACCATACA

CGTGGAGTCTACATCTATGTTTCTATGTTACTATAATACGCATGGAGTGTGTACGGATTC

TGTAATAATCTGCGCGTATCTGTTATGTC

>LYS24

AGCCGTTGGATCTAGTTGAAAATGTCTGAGACGCGTGATGAGTATGGCAACGTGGTTCGC

CAAACCGACGAGTATGGGAACCCGATTCAGCATACTGGCACCGGGACAAAGGGCGGTGCG

GGAATACATGGTGGGGGTCATGGGATTGGCACAGGTGGTGGTGGTGGACAAGGCAAGCTC

CACCGTTCAGGCTCCGGCTCTTCCTCTGTAAGTATATTAGATGTTTCTCAAGAGTCATTT

CACTTGGTAGATTGTGTTGGGATGACTAGATTAGTTGATTAACGGTTGCGATTGTAAATC

AGTGATATGGGCTTCTGTAGACCTTATTTCACGATGATAATCAATTGATACGGTGGATCG

TGATGAGCGGATCGGATGAGCCGTACATCTTACAGGCTTTTTAAAATAGAATTATAGGGT

GGGTGGTATGAGGTCCACCAAAGCCTGTAACATGGAATTACAATCATAGCCGTTAATCGG

TTCATCTCATCCGCTCATCGTGATCCAGCATGTCAAACAGAGATGGGTTTCTGGCGAAAT

GGATGTATGTAAGCCCACAAATCACAAAAATCTGACATTATGATGGAGAGAATTGCAAGG

AGTGTAAGTGAATCTACGCCGTTAGATGGATACTTCAAGGAGTGTAAGTGAATCTACGCC

GTTAGATGGATACTTTTTAACTTCGTTTTGTCAAATATTCTGTAGGATGAGGATGATGGA

CAAGGTGGGCGTAGGAAGAAGGGTTTGACGGAGAAGATCAAAGAGAAGCTGCCAGGTGGG

CAGAAGACGACGACAGGTGTGGGTTATCCAGGGAATCAGGGTGTACAGGGGGGCCGCGAG

CATGAGAAGACAGGTGTCTGTCATCCAGGGACTCAGGGTGTACAGGGGGGCCGCGAGCAT

GAGAAGACAGGTTTCGGTCATCCAGGGACTCAGGGTGGACACGAGCATGAGAAGACAGGT

GTCGGTCATCCAGGGACTCAGGGTGAGCAGGAGAAGAAGGGTATGATTGAGAAGATCAAG

GAGAAGCTGCCAGGTCACAAGTAGGATGTGTGCCCACCATACACGTGGAGTCTACATCTA

TGTTTCTATGTTACTATAATACGCATGGAGTGTGTACGGATTCTGTAATAATCTGCGCGT

ATCTGTTATGTC

>LYS27

AGCCGTTGGATCTAGTTGAAAATGTCTGAGACGCGTGATGAGTATGGCAACCAGGTTCGC

CAAACCGACGAGTATGGGAACCCGATTCAGCATACTGGCACCGGGACAAAGGCCGGTGCG

GGCATACATGGTGGAGGCCATGGGATTGGCACAGGTGGTGGTGGTGGACAAGGCAAGCTC

CACCGTTCAGGCTCCGGGTCTTCCTCTGTAAGTATATTCGATGTTTCTCAAGGGTCATTT

CACTTGGTAGATCGTGTTGTGATGACTAGATTAGTTGATTAACCGTTGTGATTGTAAATC

AGTGATATGGGCTTCTGTGTACCTTATTTCACGATGATAATCAATTGATACGGTGGATCG

TGATGAGCGGACCGGATGAGCCGTCCATCTTACAGGCTTTTTAAAATAGAATTATAGGGT

GGGTGGTATGAGGTCCACCAAAGCCTGTAACATGGAATTACAATCATAGCCGTTAATCGG

TTCATCTCATCCGCTCATCGTGATCCAGCATGTCAAACAGAGATGGGTTTCTGGCGAAAT

GGATGTATGTAAGCCCACAAATCACAAAAATCTGACATTATGATGGAGAGAATTGCAAGG

AGTGTAAGTGAATCTACGCCGTTAGATGGATACTTCAAGGAGTGTAAGTGAATCTACGCC

GTTAGATGGATACTTTTTAACTTCGTTTTGTCAAATATTCTGTAGGATGAGGATGATGGA

CAAGGTGGGCGTAGGAAGAAGGGTTTGACGGAGAAGATCAAAGAGAAGCTGCCAGGTGGG

CAGAAGACGACGACAGGTGTGGGTTATCCAGGGAATCAGGGTGTACAGGGGGGCCGCGAG

CATGAGAAGACAGGTGTCTGTCATCCAGGGACTCAGGGTGTACAGGGGGGCCGCGAGCAT

GAGAAGACAGGTTTCGGTCATCCAGGGACTCAGGGTGGACACGAGCATGAGAAGACAGGT

GTCGGTCATCCAGGGACTCAGGGTGAGCAGGAGAAGAAGGGTATGATTGAGAAGATCAAG

GAGAAGCTGCCAGGTCACAAGTAGGATGTGTGCCCACCATACACGTGGAGTCTACATCTA

TGTTTCTATGTTACTATAATACGCATGGAGTGTGTACGGATTCTGTAATAATCTGCGCGT

ATCTGTTATGTC

>LYS28

AGCCGTTGGATCTAGTTGAAAATGTCACAGACGCGTGATGAGTATGGCAGCCAGGTTCGC

CAAACCGACGAGTATGGGAACCCGATTCAGCATACTGGCACCGGGACAAAGGCCGGTGCG

GGCATACATGGTGGGGGCCATGGGATTGGCACAGGTGGTGGTGGTGGTGGACAAGGCAAG

CTCCACCGTTCAGGCTCCGGCTCTTCCTCTGTAAGTATATTTGATGTTTCTCAAGGGTCA

TTTCACTTGGTATATGGTGTTGGGATGATTAGATTAGTTGATTAACGGTTGTGATTGTAA

ATCAGTGATATGGGCTTCTGTGTACCTTATTTCACAATAATAATCAATTGATATGCTGGA

TCGTGTTGAGCGGACCGGATGAGCCGTCCATCTTGCAGGCTTTTTAAAATAGAATTATAG

GGTGGGTGGTATGAGGTCCACCAAAGCCTGTAACATGGAATTACAATCATAGCCGTTAAT

CGGTTCATCTCATCCGCTCATTGTGATCTAGCATGTCAAACAGAGATGGGTTTCTGGAGA

AATGGATGTATGTAAGCCCACAAATCAGAAAAATCTAACATTATGATGGAGAGAATGCAA

GGAGTGTTAAGTGAATCTACGCCGTTTGATGGATTTCATTGTACTTTTAACTTCGTTTTG

TCAAATATTTTGTAGGATGAGGATGATGGACAAGGTGGGCGTAGGAAGAAGGGTTTGACG

GAGAAGATCAAAGAGAAATTGCCAGGTGGAAACAAGACGACAGGTGTCTGTCATCCAGGG

ACTCAGGGTGTACAGGGGGGCCGCGAGCATGAGAAGACAGGTTGCGGTCAGGGTGGACAG

GTGGGCCGCGAGCATGAGAAGACAGGTTTCGGTCATCCAGGGACTCATGGTGGACAGGTG

GGTAATGAGCAGGAGAAGGGTATGATTGAGAAGATCCAGGAGAAGCTGCCAGGTCACAAG

TAGGATGTGTGCCCACCATACACGTGGAGTCTACATCTACGTGTCTATTATGTTACTATA

ATACGCATGGAGTGTGTCCGGAGTCTGTAATAATCTGCGCGTATCTGTTATGTC

>LYS43

AGCCGTTGGATCTAGTTGAAAATGTCTGAGACGCGTGATGAGTATGGCAACCAGGTTCGC

CAAACCGACGAGTATGGGAACCCGATTCAGCATACTGGCACCGGGACAAAGGGCGGTGCG

GGAATACATGGTGGGGGTCATGGGATTGGCACAGGTGGTGGTGGTGGACAAGGCAAGCTC

CACCGTTCAGGCTCCGGCTCTTCCTCTGTAAGTATATTAGATGTTTCTCAAGAGTCATTT

CACTTGGTAGATTGTGTTGGATGACTAGATTAGTTGATTAACGGTTGTGATTGTAAATCA

GTGATATGGGCTTATGTCTACCTTATTTCATGATGATAATCAATTGATACGGTGGATCGT

GATGAGCGGATCGGATGAGCCGTACATCTTACAGGCTTTTTAAAATAGAATTATAGGGTG

GGTGGTATGAGGTCCACCAAAGCCTGTAACATGGAATTACAATCATAGCCGTTAATAGGT

TCATCTCATCCGCTCATCGTGATCCAGCATGTCAAACAGAGATGGGTTTCTGGCGAAATG

GATGTATGTAAGCCCACAAATCACAAAAATCTGACATTATGATGGAGAGAATTGCAAGGA

GTGCAAGTGAATCTACGCCGTTAGATGGATACTTTTTAACTTCGTTTTGTCAAATATTCC

GTAGGATGAGGATGATGGACAAGGTGGGCGTAGGAAGAAGGGTTTGACGGAGAAGATCAA

AGAGAAGCTGCCAGGTGGGCAGAAGACGACAGGTGTCGGTTATCCAGGGAATCAGGGTGT

ACAGGGGGGCCGCGAGCATGAGAAGACAGGTGTCTGTCATCCAGGGACTCAGGGTGTACA

GGGGGGCCGCGAGCATGAGAAGACAGGTTTCGGTCATCCAGGGACTCAGGGTGGACACGA

GCATGAGAAGATAGGTGTCGGTCATCCAGGGACTCAGGGTGAGCAGGAGAAGAAGGGTAT

GATTGAGAAGATCAAGGAGAAGCTGCCAGGTCACAAGTAGGATGTGTGCCCACCATACAC

GTGGAGTCTACATCTATGTTTCTATGTTACTATAATACGCATGGAGTGTGTACGGATTCT

ATAATAATCTGCGCGTATCTGTTATGTC

>LYS4141

AGCCGTTGGATCTAGTTGAAAATGTCTGAGACGCGTGATGAGTATGGCAACCAGGTTCGC

CAAACCGACGAGTATGGGAACCCGATTCAGCATACTGGCACCGGGACAAAGGGCGGTGCG

GGAATACATGGTGGGGGTCATGGGATTGGCACAGGTGGTGGTGGTGGACAAGGCAAGCTC

CACCGTTCAGGCTCCGGCTCTTCCTCTGTAAGTATATTAGATGTTTCTCAAGAGTCATTT

CACTTGGTAGATTGTGTTGGATGACTAGATTAGTTGATTAACGGTTGTGATTGTAAATCA

GTGATATGGGCTTATGTCTACCTTATTTCATGATGATAATCAATTGATACGGTGGATCGT

GATGAGCGGATCGGATGAGCCGTACATCTTACAGGCTTTTTAAAATAGAATTATAGGGTG

GGTGGTATGAGGTCCACCAAAGCCTGTAACATGGAATTACAATCATAGCCGTTAATCGGT

TCATCTCATCCGCTCATCGTGATCTAGCATGTAAAACAGAGATGGGTTTCTGGAGAAATG

GATGCATGTAAGCCCACAAATCACAAAAATCTGACATTATGATGGAGAGAATGCAAGGAG

TGTAAGCGAATTTACGCCGTTAGATGGATTTCATTGTACTTTTAACTTTGTTTTGTCAAA

TATTTTGTAGGATGAGGATGATGGACAAGGTGGGCGTAGGAAGAAAGGTTTGACGGAGAA

GATCAAAGAGAAGCTGCCAGGTGGAAACAAGACGACAGGTGTCTGTCATCCAGGGACTCA

GGGTGTTAAGGGGGGCCGCGAGCATGAGAAGACAGGTTGCGGTCAGGGTGGTCAGGGTGG

ACAGGTGGGCCGCGAGCATGAGAAGACAGGTTTCGGCCATCCAGTGACTCAGGGTGGACA

GGTGGGCAACGAGCAGGAGAAGAAGGGTATGATTGAGAAGATCCAGGAGAAGCTGCCAGG

TCACAAGTAGGATGTGTGCCCACCATACACGTGGAGTCTACATCTACGTGTCTATTATGT

TACTATAATACGCATGGAATGTGTCCGGAGTCTGTAATAATCTGCGCGTATCTGTTATGT

C

>LYS414

AGCCGTTGGATCTAGTTGAAAATGTCTGAGACGCGTGATGAGTATGGCAACCAGGTTCGC

CAAACCGACGAGTATGGGAACCCGATTCAGCATACTGGCACCGGGACAAAGGGCGGTGCG

GGAATACATGGTGGGGGTCATGGGATTGGCACAGGTGGTGGTGGTGGACAAGGCAAGCTC

CACCGTTCAGGCTCCGGCTCTTCCTCTGTAAGTATATTAGATGTTTCTCAAGAGTCATTT

CACTTGGTAGATTGTGTTGGATGACTAGATTAGTTGATTAACGGTTGTGATTGTAAATCA

GTGATATGGGCTTATGTCTACCTTATTTCATGATGATAATCAATTGATACGGTGGATCGT

GATGAGCGGATCGGATGAGCCGTACATCTTACAGGCTTTTTAAAATAGAATTATAGGGTG

GGTGGTATGAGGTCCACCAAAGCCTGTAACATGGAATTACAATCATAGCCGTTAATAGGT

TCATCTCATCCGCTCATCGTGATCCAGCATGTCAAACAGAGATGGGTTTCTGGCGAAATG

GATGTATGTAAGCCCACAAATCACAAAAATCTGACATTATGATGGAGAGAATTGCAAGGA

GTGCAAGTGAATCTACGCCGTTAGATGGATACTTTTTAACTTCGTTTTGTCAAATATTCC

GTAGGATGAGGATGATGGACAAGGTGGGCGTAGGAAGAAGGGTTTGACGGAGAAGATCAA

AGAGAAGCTGCCAGGTGGGCAGAAGACGACAGGTGTCGGTTATCCAGGGAATCAGGGTGT

ACAGGGGGGCCGCGAGCATGAGAAGACAGGTGTCTGTCATCCAGGGACTCAGGGTGTACA

GGGGGGCCGCGAGCATGAGAAGACAGGTTTCGGTCATCCAGGGACTCAGGGTGGACACGA

GCATGAGAAGATAGGTGTCGGTCATCCAGGGACTCAGGGTGAGCAGGAGAAGAAGGGTAT

GATTGAGAAGATCAAGGAGAAGCTGCCAGGTCACAAGTAGGATGTGTGCCCACCATACAC

GTGGAGTCTACATCTATGTTTCTATGTTACTATAATACGCATGGAGTGTGTACGGATTCT

ATAATAATCTGCGCGTATCTGTTATGTC

>LYS51

AGCCGTTGGATCTAGTTGAAAATGTCAGTGACGCGTGATGAGTATGGCAACCAGGTTCGC

CAGACCGACGAGTATGGGAACCCGATTCAGCATACTGGCACCGGGACAAAGGCCGGTTCG

GGCATACATGGTGGGGGCCATGGGATTGGCACAGGTGGTGGTGGTGGTGGACAAGGCAAG

CTCCACCGTTCAGGCTCTGGCTCCTCCTCTGTAAGTACATTCGATGTTTCTCAAGGGTCA

TTTCACTTGGTATATCGTGTTGGGATGATTAGATTAGTTGATTAACGGTTGTGATTGTAA

ATCAGTGATATTGGCTTCTGTGTACCTTAGTTCACGATGATAATCAATTGATACGCTGGA

TCGTGATGAGCGGACCGGATGAGCCGTCCATCTTACAGGCCTTTTAAAATAGAATTATAG

GGTGGGTGGTATGAGGTCCACCAAAGCCTGTAACATGGAATTACAATCATAACCGTTAAT

CGGTTCATCTCATCCGCTCATCGTGATCTAGCATGTCAAACGGAGACGGGTTTATGGAGA

AATGGATGTATGTAAGCCCACAAATCACAAAAATCTGACATTATGATGGAGAGAATGCAA

GGAGTGTAAGCGAATTTACGCCGTTAGATGGATTTCATTGTACTTTAACTTCGTTTTGTC

AAATATTTTTTAGGATGAGGATGATGGACAAGGTGGGCGTAGGAAGAAGGGTTTGACGGA

GAAGATCAAAGAGAAGCTGCCAGGTGGAAACAAGACGACAGGTGTCTGTCATCCAGGGAC

TCAGGGTGTACAGGGGGGCCGCGAGCATGAGAAGACAGGTTGCGGTCAGGGTGGTCAGGG

TGGACAGGTGGGCCGCGAGCATGAGAAGACAGGTTTCGGCCATCCAGTGACTCAGGGTGG

ACAGGTGGGCAACGAGCAGGAGAAGAAGGGTATGATTGAGAAGATCCAGGAGAAGCTGCC

AGGTCACAAGTAGGATGTGTGCCCACCATACACGTGGAGTCTACATCTACGTGTCTATTA

TGTTACTATAATACGCATGGAATGTGTCCGGAGTCTGTAATAATCTGCGCGTATCTGTTA

TGTC

>LYS53

AGCCGTTGGATCTAGTTGAAAATGTCAGTGACGCGTGATGAGTATGGCAACCAGGTTCGG

CAGACCGACGAGTATGGGAACCCGATTCAGCATACTGGCACCGGGACAAAGGCCGGTTCG

GGCATACATGGTGGGGGCCATGGGATTGGCACAGGTGGTGGTGGTGGTGGACAAGGCAAG

CTCCACCGTTCAGGCTCTGGCTCTTCCTCTGTAAGTACATTCGATGTTTCTCAAGGGTCA

TTTCACTTGGTATATCGTGTTGGGATGATTAGATTAGTTGATTAACGGTTGTGATTGTAA

ATCAGTGATATGGGCTTCTGTGTACCTTAGTTCACGATGATAATCAATTGATACGCTGGA

TCGTGATGAGCGGACCGGATGAGCCGTCCATCTTACAGGCTTTTTAAAATAGAATTATAG

GGTGGGTGGTATGAGGTCCACCAAAGCCTGTAACATGGAATTACAATCATAGCCGTTAAT

CGGTTCATCTCATCCGCTCATCGTGATCTAGCATGTAAAACAGAGATGGGTTTCTGGAGA

AATGGATGCATGTAAGCCCACAAATCACAAAAATCTGACATTATGATGGAGAGAATGCAA

GGAGTGTAAGCGAATTTACGCCGTTAGATGGATTTCATTGTACTTTTAACTTTGTTTTGT

CAAATATTTTGTAGGATGAGGATGATGGACAAGGTGGGCGTAGGAAGAAAGGTTTGACGG

AGAAGATCAAAGAGAAGCTGCCAGGTGGAAACAAGACGACAGGTGTCTGTCATCCAGGGA

CTCAGGGTGTACAGGGGGGCCGCGAGCATGAGAAGACAGGTTGCGGTCAGGGTGGTCAGG

GTGGACAGGTGGGCCGCGAGCATGAGAAGACAGGTTTCGGCCATCCAGTGACTCAGGGTG

GACAGGTGGGCAACGAGCAGGAGAAGAAGGGTATGATTGAGAAGATCCAGGAGAAGCTGC

CAGGTCACAAGTAGGATGTGTGCCCACCATACACGTGGAGTCTACATCTACGTGTCTATT

ATGTTACTATAATACGCATGGAATGTGTCCGGAGTCTGTAATAATCTGCGCGTATCTGTT

ATGTC

>LYS58

AGCCGTTGGATCTAGTTGAAAATGTCAGTGACGCGTGATGAGTATGGCAACCAGGTTCGC

CAGACCGACGAGTATGGGAACCCGATTCAGCATACTGGCACCGGGACAAAGGCCGGTTCG

GGCATACATGGTGGGGGCCATGGGATTGGCACAGGTGGTGGTGGTGGTGGACAAGGCAAG

CTCCACCGTTCAGGCTCTGGCTCCTCCTCTGTAAGTACATTCGATGTTTCTCAAGGGTCA

TTTCACTTGGTATATCGTGTTGGGATGATTAGATTAGTTGATTAACGGTTGTGATTGTAA

ATCAGTGATATTGGCTTCTGTGTACCTTAGTTCACGATGATAATCAATTGATACGCTGGA

TCGTGATGAGCGGACCGGATGAGCCGTCCATCTTACAGGCCTTTTAAAATAGAATTATAG

GGTGGGTGGTATGAGGTCCACCAAAGCCTGTAACATGGAATTACAATCATAACCGTTAAT

CGGTTCATCTCATCCGCTCATCGTGATCTAGCATGTCAAACGGAGACGGGTTTATGGAGA

AATGGATGTATGTAAGCCCACAAATCACAAAAATCTGACATTATGATGGAGAGAATGCAA

GGAGTGTAAGCGAATTTACGCCGTTAGATGGATTTCATTGTACTTTAACTTCGTTTTGTC

AAATATTTTTTAGGATGAGGATGATGGACAAGGTGGGCGTAGGAAGAAGGGTTTGACGGA

GAAGATCAAAGAGAAGCTGCCAGGTGGAAACAAGACGACAGGTGTCTGTCATCCAGGGAC

TCAGGGTGTACAGGGGGGCCGCGAGCATGAGAAGACAGGTTGCGGTCAGGGTGGTCAGGG

TGGACAGGTGGGCCGCGAGCATGAGAAGACAGGTTTCGGCCATCCAGTGACTCAGGGTGG

ACAGGTGGGCAACGAGCAGGAGAAGAAGGGTATGATTGAGAAGATCCAGGAGAAGCTGCC

AGGTCACAAGTAGGATGTGTGCCCACCATACACGTGGAGTCTACATCTACGTGTCTATTA

TGTTACTATAATACGCATGGAATGTGTCCGGAGTCTGTAATAATCTGCGCGTATCTGTTA

TGTC

>LYS62

AGCCGTTGGATCTAGTTGAAAATGTCTGAGACGCGTGATGAGTATGGCAACGAGGTTCGC

CAAACCGACGAGTATGGGAACCCGATTCAGCATACTGGCACCGGGACAAAGGGCGGTGCG

GGAATACATGGTGGGGGTCATGGGATTGGCACAGGTGGTGGTGGTGGACAAGGCAAGCTC

CACCGTTCAGGCTCCGGCTCTTCCTCTGTAAGTATATTAGATGTTTCTCAAGAGTCATTT

CACTTGGTAGATTGTGTTGGGATGACTAGATTAGTTGATTAACGGTTGCGATTGTAAATC

AGTGATATGGGCTTCTGTAGACCTTATTTCACGATGATAATCAATTGATACGGTGGATCG

TGATGAGCGGATCGGATGAGCCGTACATCTTACAGGCTTTTTAAAATAGAATTATAGGGT

GGGTGGTATGAGGTCCACCAAAGCCTGTAACATGGAATTACAATCATAGCCGTTAATCGG

TTCATCTCATCCGCTCATCGTGATCCAGCATGTCAAACGGAGATGGGTTACTGGCGAAAT

GGATGTATGTAAGCCCACAAATCACAAAAATCTGACATTATGATGGAGAGAATTGCAAGG

AGTGTAAGTGAATCTACGCCGTTAGATGGATACTTTTTAACTTCGTTTTGTCAAATATTC

TGTAGGATGAGGATGATGGACAAGGTGGGCGTAGGAAGAAGGGTTTGACGGAGAAGATCA

AAGAGAAGCTGCCAGGTGGGCAGAAGACGACAGGTGTCGGTTATCCAGGGAATCAGGGTG

TACAGGGGGGCCGCGAGCATGAGAAGACAGGTGTCTGTCATCCAGGGACTCAGGGTGTAC

AGGGGGGCCGCGAGCATGAGAAGACAGGTTTCGGTCATCCAGGGACTCAGGGTGGACACG

AGCATGAGAAGACAGGTGTCGGTCATCCAGGGACTCAGGGTGAGCAGGAGAAGAAGGGTA

TGATTGAGAAGATCAAGGAGAAGCTGCCAGGTCACAAGTAGGATGTGTGCCCACCATACA

CGTGGAGTCTACATCTATGTTTCTATGTTACTATAATACGCATGGAGTGTGTACGGATTC

TATAATAATCTGCGCGTATCTGTTATGTC

>LYS67

AGCCGTTGGATCTAGTTGAAAATGTCTGAGACGCGTGATGAGTATGGCAACGAGGTTCGC

CAAACCGACGAGTATGGGAACCCGATTCAGCATACTGGCACCGGGACAAAGGGCGGTGCG

GGAATACATGGTGGGGGTCATGGGATTGGCACAGGTGGTGGTGGTGGACAAGGCAAGCTC

CACCGTTCAGGCTCCGGCTCTTCCTCTGTAAGTATATTAGATGTTTCTCAAGAGTCATTT

CACTTGGTAGATTGTGTTGGGATGACTAGATTAGTTGATTAACGGTTGCGATTGTAAATC

AGTGATATGGGCTTCTGTAGACCTTATTTCACGATGATAATCAATTGATACGGTGGATCG

TGATGAGCGGATCGGATGAGCCGTACATCTTACAGGCTTTTTAAAATAGAATTATAGGGT

GGGTGGTATGAGGTCCACCAAAGCCTGTAACATGGAATTACAATCATAGCCGTTAATCGG

TTCATCTCATCCGCTCATCGTGATCCAGCATGTCAAACGGAGATGGGTTACTGGCGAAAT

GGATGTATGTAAGCCCACAAATCACAAAAATCTGACATTATGATGGAGAGAATTGCAAGG

AGTGTAAGTGAATCTACGCCGTTAGATGGATACTTTTTAACTTCGTTTTGTCAAATATTC

TGTAGGATGAGGATGATGGACAAGGTGGGCGTAGGAAGAAGGGTTTGACGGAGAAGATCA

AAGAGAAGCTGCCAGGTGGGCAGAAGACGACAGGTGTCGGTTATCCAGGGAATCAGGGTG

TACAGGGGGGCCGCGAGCATGAGAAGACAGGTGTCTGTCATCCAGGGACTCAGGGTGTAC

AGGGGGGCCGCGAGCATGAGAAGACAGGTTTCGGTCATCCAGGGACTCAGGGTGGACACG

AGCATGAGAAGACAGGTGTCGGTCATCCAGGGACTCAGGGTGAGCAGGAGAAGAAGGGTA

TGATTGAGAAGATCAAGGAGAAGCTGCCAGGTCACAAGTAGGATGTGTGCCCACCATACA

CGTGGAGTCTACATCTATGTTTCTATGTTACTATAATACGCATGGAGTGTGTACGGATTC

TATAATAATCTGCGCGTATCTGTTATGTC

>LYS68

AGCCGTTGGATCTAGTTGAAAATGTCTGAGACGCGTGATGAGTATGGCAACGAGGTTCGC

CAAACCGACGAGTATGGGAACCCGATTCAGCATACTGGCACCGGGACAAAGGGCGGTGCG

GGAATACATGGTGGGGGTCATGGGATTGGCACAGGTGGTGGTGGTGGACAAGGCAAGCTC

CACCGTTCAGGCTCCGGCTCTTCCTCTGTAAGTATATTAGATGTTTCTCAAGAGTCATTT

CACTTGGTAGATTGTGTTGGGATGACTAGATTAGTTGATTAACGGTTGCGATTGTAAATC

AGTGATATGGGCTTCTGTAGACCTTATTTCACGATGATAATCAATTGATACGGTGGATCG

TGATGAGCGGATCGGATGAGCCGTACATCTTACAGGCTTTTTAAAATAGAATTATAGGGT

GGGTGGTATGAGGTCCACCAAAGCCTGTAACATGGAATTACAATCATAGCCGTTAATCGG

TTCATCTCATCCGCTCATCGTGATCCAGCATGTCAAACGGAGATGGGTTACTGGCGAAAT

GGATGTATGTAAGCCCACAAATCACAAAAATCTGACATTATGATGGAGAGAATTGCAAGG

AGTGTAAGTGAATCTACGCCGTTAGATGGATACTTTTTAACTTCGTTTTGTCAAATATTC

TGTAGGATGAGGATGATGGACAAGGTGGGCGTAGGAAGAAGGGTTTGACGGAGAAGATCA

AAGAGAAGCTGCCAGGTGGGCAGAAGACGACAGGTGTCGGTTATCCAGGGAATCAGGGTG

TACAGGGGGGCCGCGAGCATGAGAAGACAGGTGTCTGTCATCCAGGGACTCAGGGTGTAC

AGGGGGGCCGCGAGCATGAGAAGACAGGTTTCGGTCATCCAGGGACTCAGGGTGGACACG

AGCATGAGAAGACAGGTGTCGGTCATCCAGGGACTCAGGGTGAGCAGGAGAAGAAGGGTA

TGATTGAGAAGATCAAGGAGAAGCTGCCAGGTCACAAGTAGGATGTGTGCCCACCATACA

CGTGGAGTCTACATCTATGTTTCTATGTTACTATAATACGCATGGAGTGTGTACGGATTC

TATAATAATCTGCGCGTATCTGTTATGTC

>LYS74

AGCCGTTGGATCTAGTTGAAAATGTCTGAGACGCGTGACGAGTATGGCAACCAGGTTCGC

CAAACCGACGAGTATGGGAACCCGATTCAGCATACTGGCACCGGGACAAAGGGCGGTGCG

GGAATACATGGTGGGGGTCATGGGATTGGCACAGGTGGTGGTGGTGGACAAGGCAAGCTC

CACCGTTCAGGCTCCGGCTCTTCCTCTGTAAGTATATTAGATGTTTCTCAAGAGTCATTT

CACTTGGTAGATTGTGTTGGGATGACTAGATTAGTTGATTAACGGTTGCGATTGTAAATC

AGTGATATGGGCTTCTGTCTACCTTATTTCATGATGATAATCAATTGATACGGTGGATCG

TGATGAGCGGATCGGATGAGCCGTACATCTTACAGGCTTTTTAAAATAGAATTATAGGGT

GGGTGGTATGAGGTCCACCAAAGCCTGTAACATGGAATTACAATCATAGCCGTTAATCGG

TTCATCTCATCCGCTCATCGTGATCCAGCATGTCAAACGGAGATGGGTTACTGGCGAAAT

GGATGTATGTAAGCCCACAAATCACAAAAATCTGACATTATGATGGAGAGAATTGCAAGG

AGTGTAAGTGAATCTACGCCGTTAGATGGATACTTTTTAACTTCGTTTTGTCAAATATTC

TGTAGGATGAGGATGATGGACAAGGTGGGCGTAGGAAGAAGGGTTTGACGGAGAAGATCA

AAGAGAAGCTGCCAGGTGGGCAGAAGACGACGACAGGTGTGGGTTATCCAGGGAATCAGG

GTGTACAGGGGGGCCGCGAGCATGAGAAGACAGGTGTCTGTCATCCAGGGACTCAGGGTG

TACAGGGGGGCCGCGAGCATGAGAAGACAGGTTTCGGTCATCCAGGGACTCAGGGTGGAC

ACGAGCATGAGAAGACAGGTGTCGGTCATCCAGGGACTCAGGGTGAGCAGGAGAAGAAGG

GTATGATTGAGAAGATCAAGGAGAAGCTGCCAGGTCACAAGTAGGATGTGTGCCCACCAT

ACACGTGGAGTCTACATCTATGTTTCTATGTTACTATAATACGCATGGAGTGTGTACGGA

TTCTGTAATAATCTGCGCGTATCTGTTATGTC

>LYS73

AGCCGTTGGATCTAGTTGAAAATGTCTGAGACGCGTGATGAGTATGGCAACGAGGTTCGC

CAAACCGACGAGTATGGGAACCCGATTCAGCATACTGGCACCGGGACAAAGGGCGGTGCG

GGAATACATGGTGGGGGTCATGGGATTGGCACAGGTGGTGGTGGTGGACAAGGCAAGCTC

CACCGTTCAGGCTCCGGCTCTTCCTCTGTAAGTATATTAGATGTTTCTCAAGAGTCATTT

CACTTGGTAGATTGTGTTGGGATGACTAGATTAGTTGATTAACGGTTGCGATTGTAAATC

AGTGATATGGGCTTCTGTAGACCTTATTTCACGATGATAATCAATTGATACGGTGGATCG

TGATGAGCGGATCGGATGAGCCGTACATCTTACAGGCTTTTTAAAATAGAATTATAGGGT

GGGTGGTATGAGGTCCACCAAAGCCTGTAACATGGAATTACAATCATAGCCGTTAATCGG

TTCATCTCATCCGCTCATCGTGATCCAGCATGTCAAACGGAGATGGGTTACTGGCGAAAT

GGATGTATGTAAGCCCACAAATCACAAAAATCTGACATTATGATGGAGAGAATTGCAAGG

AGTGTAAGTGAATCTACGCCGTTAGATGGATACTTTTTAACTTCGTTTTGTCAAATATTC

TGTAGGATGAGGATGATGGACAAGGTGGGCGTAGGAAGAAGGGTTTGACGGAGAAGATCA

AAGAGAAGCTGCCAGGTGGGCAGAAGACGACAGGTGTCGGTTATCCAGGGAATCAGGGTG

TACAGGGGGGCCGCGAGCATGAGAAGACAGGTGTCTGTCATCCAGGGACTCAGGGTGTAC

AGGGGGGCCGCGAGCATGAGAAGACAGGTTTCGGTCATCCAGGGACTCAGGGTGGACACG

AGCATGAGAAGATAGGTGTCGGTCATCCAGGGACTCAGGGTGAGCAGGAGAAGAAGGGTA

TGATTGAGAAGATCAAGGAGAAGCTGCCAGGTCACAAGTAGGATGTGTGCCCACCATACA

CGTGGAGTCTACATCTATGTTTCTATGTTACTATAATACGCATGGAGTGTGTACGGATTA

TGTAATAATCTGCGCGTATCTGTTATGTC

>LYS78

AGCCGTTGGATCTAGTTGAAAATGTCTGAGACGCGTGATGAGTATGGCAACGAGGTTCGC

CAAACCGACGAGTATGGGAACCCGATTCAGCATACTGGCACCGGGACAAAGGGCGGTGCG

GGAATACATGGTGGGGGTCATGGGATTGGCACAGGTGGTGGTGGTGGACAAGGCAAGCTC

CACCGTTCAGGCTCCGGCTCTTCCTCTGTAAGTATATTAGATGTTTCTCAAGAGTCATTT

CACTTGGTAGATTGTGTTGGGATGACTAGATTAGTTGATTAACGGTTGCGATTGTAAATC

AGTGATATGGGCTTCTGTAGACCTTATTTCACGATGATAATCAATTGATACGGTGGATCG

TGATGAGCGGATCGGATGAGCCGTACATCTTACAGGCTTTTTAAAATAGAATTATAGGGT

GGGTGGTATGAGGTCCACCAAAGCCTGTAACATGGAATTACAATCATAGCCGTTAATCGG

TTCATCTCATCCGCTCATCGTGATCCAGCATGTCAAACGGAGATGGGTTACTGGCGAAAT

GGATGTATGTAAGCCCACAAATCACAAAAATCTGACATTATGATGGAGAGAATTGCAAGG

AGTGTAAGTGAATCTACGCCGTTAGATGGATACCTTTTAACTTCGTTTTGTCAAATATTC

CGTAGGATGAGGATGATGGACAAGGTGGGCGTAGGAAGAAGGGTTTGACGGAGAAGATCA

AAGAGAAGCTGCCAGGTGGGCAGAAGACGACAGGTGTCGGTTATCCAGGGAATCAGGGTG

TACAGGGGGGCCGCGAGCATGAGAAGACAGGTGTCTGTCATCCAGGGACTCAGGGTGTAC

AGGGGGGCCGCGAGCATGAGAAGACAGGTTTCGGTCATCCAGGGACTCAGGGTGGACACG

AGCATGAGAAGACAGGTGTCGGTCATCCAGGGACTCAGGGTGAGCAGGAGAAGAAGGGTA

TGATTGAGAAGATCAAGGAGAAGCTGCCAGGTCACAAGTAGGATGTGTGCCCACCATACA

CGTGGAGTCTACATCTATGTTTCTATGTTACTATAATACGCATGGAGTGTGTACGGATTA

TGTAATAATCTGCGCGTATCTGTTATGTC

>LYS82

AGCCGTTGGATCTAGTTGAAAATGTCTGAGACGCGTGATGAGTATGGCAACGAGGTTCGC

CAAACCGACGAGTATGGGAACCCGATTCAGCATACTGGCACCGGGACAAAGGGCGGTGCG

GGAATACATGGTGGGGGTCATGGGATTGGCACAGGTGGTGGTGGTGGACAAGGCAAGCTC

CACCGTTCAGGCTCCGGCTCTTCCTCTGTAAGTATATTAGATGTTTCTCAAGAGTCATTT

CACTTGGTAGATTGTGTTGGGATGACTAGATTAGTTGATTAACGGTTGCGATTGTAAATC

AGTGATATGGGCTTCTGTAGACCTTATTTCACGATGATAATCAATTGATACGGTGGATCG

TGATGAGCGGATCGGATGAGCCGTACATCTTACAGGCTTTTTAAAATAGAATTATAGGGT

GGGTGGTATGAGGTCCACCAAAGCCTGTAACATGGAATTACAATCATAGCCGTTAATCGG

TTCATCTCATCCGCTCATCGTGATCCAGCATGTCAAACGGAGATGGGTTACTGGCGAAAT

GGATGTATGTAAGCCCACAAATCACAAAAATCTGACATTATGATGGAGAGAATTGCAAGG

AGTGTAAGTGAATCTACGCCGTTAGATGGATACTTTTTAACTTCGTTTTGTCAAATATTC

TGTAGGATGAGGATGATGGACAAGGTGGGCGTAGGAAGAAGGGTTTGACGGAGAAGATCA

AAGAGAAGCTGCCAGGTGGGCAGAAAACGACAGGTGTCGGTTATCCAGGGAATCAGGGTG

TACAGGGGGGCCGCGAGCATGAGAAGACAGGTGTCTGTCATCCAGGGAATCAGGGTGTAC

AGGGGGGCCGCGAGCATGAGAAGACAGGTTTCGGTCATCCAGGGACTCAGGGTGGACACG

AGCATGAGAAGACAGGTGTCGGTCATCCAGGGACTCAGGGTGAGCAGGAGAAGAAGGGTA

TGATTGAGAAGATCAAGGAGAAGCTGCCAGGTCACAAGTAGGATGTGTGCCCACCATACA

CGTGGAGTCTACATCTATGTTTCTATGTTACTATAATACGCATGGAGTGTGTACGGATTC

TGTAATAATCTGCGCGTATCTGTTATGTC

>LYS87

AGCCGTTGGATCTAGTTGAAAATGTCAGTGACGCGTGATGAGTATGGCAACCAGGTTCGC

CAGACCGACGAGTATGGGAACCCGATTCAGCATACTGGCACCGGGACACAGGCCGGTTCG

GGCATACATGGTGGGGGCCATGGGATTGACACAGGTGGTGGACAAGGCAAGCTCCACCGT

TCAGGCTCTGGCTCTTCCTCTGTAAGTATATTAGATGTTTCTCAAGAGTCATTTCACTTG

GTAGATTGTGTTGGGATGACTAGATTAGTTGATTAACGGTTGCGATTGTAAATCAGTGAT

ATGGGCTTCTGTAGACCTTATTTCACGATGATAATCAATTGATACGGTGGATCGTGATGA

GCGGATCGGATGAGCCGTACATCTTACAGGCTTTTTAAAATAGAATTATAGGGTGGGTGG

TATGAGGTCCACCAAAGCCTGTAACATGGAATTACAATCATAGCCGTTAATCGGTTCATC

TCATCCGCTCATCGTGATCCAGCATGTCAAACGGAGATGGGTTACTGGCGAAATGGATGT

ATGTAAGCCCACAAATCACAAAAATCTGACATTATGATGGAGAGAATTGCAAGGAGTGTA

AGTGAATCTACGCCGTTAGATGGATACTTTTTAACTTCGTTTTGTCAAATATTCTGTAGG

ATGAGGATGATGGACAAGGTGGGCGTAGGAAGAAGGGTTTGACGGAGAAGATCAAAGAGA

AGCTGCCAGGTGGGCAGAAAACGACAGGTGTCGGTTATCCAGGGAATCAGGGTGTACAGG

GGGGCCGCGAGCATGAGAAGACAGGTGTCTGTCATCCAGGGAATCAGGGTGTACAGGGGG

GCCGCGAGCATGAGAAGACAGGTTTCGGTCATCCAGGGACTCAGGGTGGACACGAGCATG

AGAAGACAGGTGTCGGTCATCCAGGGACTCAGGGTGAGCAGGAGAAGAAGGGTATGATTG

AGAAGATCAAGGAGAAGCTGCCAGGTCACAAGTAGGATGTGTGCCCACCATACACGTGGA

GTCTACATCTATGTTTCTATGTTACTATAATACGCATGGAGTGTGTACGGATTCTGTAAT

AATCTGCGCGTATCTGTTATGTC

>LYS88

AGCCGTTGGATCTAGTTGAAAATGTCAGTGACGCGTGATGAGTATGGCAACCAGGTTCGC

CAGACCGACGAGTATGGGAACCCGATTCAGCATACTGGCACCGGGACACAGGCCGGTTCG

GGCATACATGGTGGGGGCCATGGGATTGACACAGGTGGTGGACAAGGCAAGCTCCACCGT

TCAGGCTCTGGCTCTTCCTCTGTAAGTACATTCGATGTTTCTCAAGGGTCATTTCACTTG

GTATATCGTGTTGGGATGATTAGATTAGTTGATTAACGGTTGTGATTGTAAATCAGTGAT

ATGGGCTTCTGTGTACCTTAGTTCACGATGATAATCAATTGATACGCTGGATCGTGATGA

GCGGACCGGATGAGCCGTCCATCTTACAGGCTTTTTAAAATAGAATTATAGGGTGGGCGG

TATGAGGTCCACCAAAGCCTGTAACATGGAATTACAATCATAGCCGTTAATCGGTTCATC

TCATCCGCTCATAGTGATCTAGCATGTCAAACAGAGATGGGTTTCTGGAGAAATGGATGT

ATTTAAGCCCACAAATCACAAAAATCTGACATTATGATGGAGAGAATGCAAGGAGTGTTA

AGCGAATTTACGCCGTTAGATGGATTTCATTGTACTTTAACTTTGTTTTGTCAAATATTT

TGTAGGATGAGGATGATGGACAAGGTGGGCGTAGGAAGAAGGGTTTGACGGAGAAGATCA

AAGAGAAGCTGCCAGGTGGAAATAAGACGACAGGTGTCTGTCATCCAGGGACTCAGGGTG

TACAGGGGGGCCGCGAGCATGAGAAGACAGGTTGCGGTCAGGGTGGTCAGGGTGGACAGG

TGGGCCGCGAGCATGAGAAGACAGGTTTCGGCCATCCAGTGACTCAGGGTGGACAGGTGG

GCAACGAGCAGGAGAAGAAGGGTATGATTGAGAAGATCCAGGAGAAGCTGCCAGGTCACA

AGTAGGATGTGTGCCCACCATACACGTGGAGTCTACATCTACGTGTCTATTATGTTACTA

TAATACGCATGGAGTGTGTCCGGAGTCTGTAATAATCTGCGCGTATCTGTTATGTC
