# Supplementary material for: Interspecies evolutionary divergence in Liriodendron, evidence from the nucleotide variations of LcDHN-like gene
Source: BMC Evol Biol. 2018 Dec 19;18:195. doi: 10.1186/s12862-018-1318-7 (PMC6300021; doi:10.1186/s12862-018-1318-7)
Supplement: Supplementary file 4 — The list of 58 predicted proteins in Liriodendron. (DOCX 19 kb) [file 12862_2018_1318_MOESM4_ESM.docx]

| **Additional file 4: The list of 58 predicted proteins in *Liriodendron*** | |
| --- | --- |
| P1 | MSETRDEYGNQVRQTDEYGNPIQHTGTGTKPGSGIHGGGHGIGTGGGGGQGKLHRPGSGSSSDEDDGQGGRRKKGLTEKIKEKLPGGNKTTGVCHPGTQGVQGGREHEKPGCGQGGQVGREHEKTGFGHPGTQGGQVGSEQEKKGMIEKIKEKLPGHK |
| P2 | MSETRDEYGNQVRQTDEYGNPIQHTGTGTKPGSGIHGGGHGIGTGGGGGQGKLHRPGSGSSSDEDDGQGGRRKKGLTEKIKEKLPGGNKTTGVCHPGTQGVQGGREHEKPGFGQGGQVGREHEKTGFGHPGTQGGQVGSEQEKKGMIEKIKEKLPGHK |
| P3 | MSETREEYGNQVRQTDEYGNPIQHTGTGTKPGSGIHGGGHGIGTGGGGGQGKLHRPGSGSSSDEDDGQGGRRKKGLTEKIKEKLPGGNKTTGVCHPGTQGVQGGREHEKPGCGQGGQVGREHEKTGFGHPGTQGGQVGSEQEKKGMIEKIKEKLPGHK |
| P4 | MSETRDEYGNQVRQTDEYGNPIQHTGTGTKAGSGIHGGAHGIGTGGGGGQGKLHRPGSGSSSDEDDGQGGRRKKGLTEKIKEKLPGGNKTTGVCHPGTQGVQGGREHEKPGCGQGGQVGREHEKTGFGHPGTQGGQVGSEQEKKGMIEKIKEKLPGHK |
| P5 | MSETRDEYGNQVRQTDEYENPIQHTGTGTKPGSGIHGGGHGIGTGGGGGQGKLHRPGSGSSSDEDDGQGGRRKKGLTEKIKEKLPGGNKTTGVCHPGTQGVQGGREHEKPGCGQGGQVGREHEKTGFGHPGTQGGQVGSEQEKKGMIEKIKEKLPGHK |
| P6 | MSETRDEYGNQVRQTDEYGNPIQHSGTGTKPGSGIHGGGHGIGTGGGGGQGKLHRPGSGSSSDEDDGQGGRRKKGLTEKIKEKLPGGNKTTGVCHPGTQGVQGGREHEKPGCGQGGQVGREHEKTGFGHPGTQGGQVGSEQEKKGMIEKIKEKLPGHK |
| P7 | MSETRDEYGNQVRQTDEYGNPIQHSGTGTKPGSGIHGGGHGIGTGGGGGQGKLHRPGSGSSSDEDDGQGGRRKKGLTEKIKEKLPGGNKTTGVCHPGTQGVQGGREHEKPGCGQGGQVGREHEKTGFGQGGQVGREQEKKGMIEKIKEKLPGHK |
| P8 | MSETRDEYGNQVRQTDEYGNPIQHTGTGTKPGSGIHGGGHGIGTGGGGGQGKLHRPGSGSSSDEDDGQGGRRKKGLTEKIKEKLPGGNKTTGVCHPGTQGVQGGREHEKQGCGQGGQVGREHEKTGFGHPGTQGGQVGSEQEKKGMIEKIKEKLPGHK |
| P9 | MSETRDEYGNQVRQTDEYGNPIQHSGTGTKPGSGIHGGAHGIGTGGGGGQGKLHRPGSGSSSDEDDGQGGRRKKGLTEKIKEKLPGGNKTTGVCHPGTQGVQGGREHEKPGCGQGGQVGREHEKTGFGHPGTQGGQVGSEQEKKGMIEKIKEKLPGHK |
| P10 | MSETRDEYGNQVRQTDEYGNPIQHTGTGTKPGSGIHGGGHGIGTGGGGGQGKLHRSGSGSSSDEDDGQGGRRKKGLTEKIKEKLPGGNKTTGVCHPGTQGVQGGREHEKPGCGQGGQVGREHEKTGFGHPGTQGGQVGSEQEKKGMIEKIKEKLPGHK |
| P11 | MSETRDEYGNQVRQTDEYGNPIQHSGTGTKPGSGIHGGGHGIGTGGGGGQGKLHRPGSGSSSDEDDGQGGRRKKGLTEKIKEKLPGGNKTTGVCHQGTQGVQGGREHEKPGCGQGGQVGREHEKTGFGHPGTQGGQVGSEQEKKGMIEKIKEKLPGHK |
| P12 | MSETRDEYGNQVRQTDEYGNPIQHTGTGTKPGSGIHGGGHGIGTGGGGGQGKLHRPGSGSSSDEDDGQGGRRKKGLTEKIKEKLPGGNKTTGACHPGTQGVQGGREHEKPGCGQGGQVGREHEKTGFGHPGTQGGQVGSEQEKKGMIEKIKEKLPGHK |
| P13 | MSETRDEYGNQVRQTDEYGNPIQHTGTGTKPGSGIHGGGHGIGTGGGGGQGKLHRPGSGSSSDEDDGQGGRRKKGLTEKIKEKLPGGNKTTGVCHPGTKGVQGGREHEKPGCGQGGQVGREHEKTGLGHPGTQGGQVGSEQEKKGMIEKIKEKLPGHK |
| P14 | MSETRDEYGNQVRQTDEYGNPIQHTGTGTKPGSGIHGGGHGIGTGGGGGQGKLHRPGSGSSSDEDDGQGGRRKKGLTEKIKEKLPGGNKTTGVCHPGTQGVQGGREHEKPGCGQGGQVGREHEKTGLGHPGTQGGQVGSEQEKKGMIEKIKEKLPGHK |
| P15 | MSETRDEYGNQVRQTDEYGNPIQHTGTGTKPGSGIHGGGHGIGTGGGGGQGKLHRPGSGSSSDEDDGQGERRKKGLTEKIKEKLPGGNKTTGVCHPGTQGVQGGREHEKPGCGQGGQVGREHEKTGFGHPGTQGGQVGSEQEKKGMIEKIKEKLPGHK |
| P16 | MSETRDEYGNQVRQTDEYGNPIQHTGTGTKPGSGIHGGGHGIGTGGGGGQGKLHRPGSGSSSDEDDGQGGRRKKGLTEKIKEKLPGGNKTTGVCHPGTQGAQGGREHEKPGCGQGGQVGREHEKTGFGHPGTQGGQVGSEQEKKGMIEKIKEKLPGHK |
| P17 | MSETRDEYGNQVRQTDEYGNPIQHTGTGTKPGSGIHGGGNGIGTGGGGGQGKLHRPGSGSSSDEDDGQGGRRKKGLTEKIKEKLPGGNKTTGACHPGTQGVQGGREHEKPGCGQGGQVGREHEKTGFGHPGTQGGQVGSEQEKKGMIEKIKEKLPGHK |
| P18 | MSVTRDEYGNQVRQTDEYGNPIQHTGTGTKAGSGIHGGGHGIGTGGGGGGQGKLHRSGSGSSSDEDDGQGGRRKKGLTEKIKEKLPGGNKTTGVCHPGTQGVQGGREHEKTGCGQGGQGGQVGREHEKTGFGHPVTQGGQVGNEQEKKGMIEKIQEKLPGHK |
| P19 | MSVTRDEYGNQVRQTDEYGNPIQHTGTGTKAGSGIHGGGHGIGTGGGGGGQGKLHRSGSGSSSDEDDGQGGRRKKGLTEKIKEKLPGGNKTTGVCHPGTQGVQGGREHEKTGCGQGGQGGQVGREHEKTGFGHAVTQGGQVGNEQEKKGMIEKIQEKLPGHK |
| P20 | MSETRDEYGNQVRQTDEYGNPIQHTGTGTKAGAGIHAGGHGIGTGGGGGQGKLHRSGSGSSSDEDDGQGGRRKKGLTEKIKEKLPGGQKTTGVGYPGNQGVQGGREHEKTGVCHPGTQGVQGGREHEKTGFGHPGTQGGHEHEKTGVGHPGTQGEQEKKGMIEKIKEKLPGHK |
| P21 | MSETRDEYGNQVRQTDEYGNPIQHTGTGTKAGAGIHAGGHGIGTGGGGGQGKLHRSGSGSSSDEDDGQGGRRKKGLTEKIKEKLPGGQKTTTGVGYPGNQGVQGGREHEKTGVCHPGTQGVQGGREHEKTGFGHPGTQGGHEHEKTGVGHPGTQGEQEKKGMIEKIKEKLPGHK |
| P22 | MSETRDEYGNQVRQTDEYGNPIQHTGTGTKGGAGIHGGGHGIGTGGGGGQGKLHRSGSGSSSDEDDGQGGRRKKGLTEKIKEKLPGGNKTTGVCHPGTQGVQGGREHEKTGCGQGGQGGQVGREHEKTGFGHAVTQGGQVGNEQEKKGMIEKIQEKLPGHK |
| P23 | MSETRDEYGNQVRQTDEYGNPIQHTGTGTKGGAGIHGGGHGIGTGGGGGQGKLHRSGSGSSSDEDDGQGGRRKKGLTEKIKEKLPGGNKTTGVCHPGTQGVQGGREHEKTGCGQGGQGGQVGREHEKTGFGHPVTQGGQVGNEQEKKGMIEKIQEKLPGHK |
| P24 | MSVTRDEYGNQVRQTDEYGNPIQHTGTGTKAGLGIHGGGHGIGTGGGGGGQGKLHRSGSGSSSDEDDGQGGRRKKGLMEKIKEKLPGGNKTTGVCHPGTQGVQGGREHEKTGCGQGGQVRREHEKTGFGHPGTQGGQVGNEQEKKGMIEKIQENLPGHK |
| P25 | MSVTRDEYGNQVRQTDEYGNPIQHTGTGTKAGSGIHGGGHGIGTGGGGGGQGKLHRSGSGSSSDEDDGQGGRRKKGLTEKIKEKLPGGQKTTGVGYPGNQGVQGGREHEKTGVCHPGTQGVQGGREHEKTGFGHPGTQGGHEHEKTGVGHPGTQGEQEKKGMIEKIKEKLPGHK |
| P26 | MSVTRDEYGNQVRQTDEYGNPIQHTGTGTKAGSGIHGGGHGIGTGGGGGGQGKLHRSGSGSSSDEDDGQGGRRKKGLTEKIKEKLPGGQKTTTGVGYPGNQGVQGGREHEKTGVCHPGTQGVQGGREHEKTGFGHPGTQGGHEHEKTGVGHPGTQGEQEKKGMIEKIKEKLPGHK |
| P27 | MSETRDEYGNQVSQTDEYGNPIQHTGTGTKGGAGIHGGGHGIGTGGGGGQGKLHRSGSGSSSDEDDGQGGRRKKGLTEKIKEKLPGGQKTTGVGYPGNQGVQGGREHEKTGVCHPGTQGVQGGREHEKTGFGHPGTQGGHEHEKTGVGHPGTQGEQEKKGMIEKIKEKLPGHK |
| P28 | MSVTRDEYGNQVRQTDEYGNPIQHTGTGTKAGSGIHGGGHGIGTGGGGGGQGKLHRSGSGSSSDEDDGQGGRRKKGLTEKIKEKLPGGNKTTGVCHPGTQGVQGGREHEKTGCGQGGQGGQVGREHEKTGFGHAVTQGGQVGNEQEKKGMIEKIKEKLPGHK |
| P29 | MSETRDEYGNVVRQTDEYGNPIQHTGTGTKGGAGIHGGGHGIGTGGGGGQGKLHRSGSGSSSDEDDGQGGRRKKGLTEKIKEKLPGGQKTTTGVGYPGTQGVQGGREHEKTGVCHPGTQGVQGGREHEKTGFGHPGTQGGHEHEKTGVGHPGTQGEQEKKGMIEKIKEKLPGHK |
| P30 | MSETRDEYGNQVRQTDEYGNPIQHTGTGTKAGAGIHGGGHGIGTGGGGGQGKLHRSGSGSSSDEDDGQGGRRKKGLTEKIKEKLPGGQKTTTGVGYPGNQGVQGGREHEKTGVCHPGTQGVQGGREHEKTGFGHPGTQGGHEHEKTGVGHPGTQGEQEKKGMIEKIKEKLPGHK |
| P31 | MSETRDEYGNQVRQTDEYGNPIQHTGTGTKAGAGIHGGGHGIGTGGGGGQGKLHRSGSGSSSDEDDGQGGRRKKGLTEKIKEKLPGGQKTTGVGYPGNQGVQGGREHEKTGVCHPGNQGVQGGREHEKTGFGHPGTQGGHEHEKTGVGHPGTQGEQEKKGMIEKIKEKLPGHK |
| P32 | MSETRDEYGNQVRQTDEYGNPIQHTGTGTKGGAGIHGGGHGIGTGGGGGQGKLHRSGSGSSSDEDDGQGGRRKKGLTEKIKEKLPGGQKTTGVGYPGNQGVQGGREHEKTGVCHPGTQGVQGGREHEKTGFGHPGTQGGHEHEKTGVGHPGTQGEQEKKGMIEKIKEKLPGHK |
| P33 | MSETRDEYGNQVRQTDEYGNPIQHTGTGTKGGAGIHGGGHGIGTGGGGGQGKLHRSGSGSSSDEDDGQGGRRKKGLTEKIKEKLPGGQKTTGVGYPGNQGVQGGREHEKTGVCHPGTQGVQGGREHEKTGFGHPGTQGGHEHEKIGVGHPGTQGEQEKKGMIEKIKEKLPGHK |
| P34 | MSETRDEYGNQVRQTDEYGNPIQHTGTGTKGGAGIHGGGHGIGTGGGGGQGKLHRSGSGSSSDEDDGQGGRRKKGLTEKIKEKLPGGQKTTTGVGYPGNQGVQGGREHEKTGVCHPGTQGVQGGREHEKTGFGHPGTQGGHEHEKTGVGHPGTQGEQEKKGMIEKIKEKLPGHK |
| P35 | MSETRDEYGNEVRQTDEYGNPIQHTGTGTKGGAGIHGGGHGIGTGGGGGQGKLHRSGSGSSSDEDDGQGGRRKKGLTEKIKEKLPGGQKTTGVGYPGNQGVQGGREHEKTGVCHPGNQGVQGGREHEKTGFGHPGTQGGHEHEKTGVGHPGTQGEQEKKGMIEKIKEKLPGHK |
| P36 | MSETRDEYGNEVRQTDEYGNPIQHTGTGTKGGAGIHGGGHGIGTGGGGGQGKLHRSGSGSSSDEDDGQGGRRKKGLTEKIKEKLPGGQKTTGVGYPGNQGVQGGREHEKTGVCHPGTQGVQGGREHEKTGFGHPGTQGGHEHEKTGVGHPGTQGEQEKKGMIEKIKEKLPGHK |
| P37 | MSETRDEYGNEVRQTDEYGNPIQHTGTGTKGGAGIHGGGHGIGTGGGGGQGKLHRSGSGSSSDEDDGQGGRRKKGLTEKIKEKLPGGQKTTTGVGYPGNQGVQGGREHEKTGVCHPGTQGVQGGREHEKTGFGHPGTQGGHEHEKTGVGHPGTQGEQEKKGMIEKIKEKLPGHK |
| P38 | MSETRDEYGNEVRQTDEYGNPIQHTGTGTKGGAGIHGGGHGIGTGGGGGQGKLHRSGSGSSSDEDDGQGGRRKKGLTEKIKEKLPGGQKTTGVGYPGNQGVQGGREHEKTGVCHPGTQGVQGGREHEKTGFGHPGTQGGHEHEKIGVGHPGTQGEQEKKGMIEKIKEKLPGHK |
| P39 | MSQTRDEYGSQVRQTDEYGNPIQHTGPGTKAGSGIHGGGHGIGTGGGGGGQGKLHRSGSGSSSSSDEDDGQGGRRNRGLTEKIEEKLPGGNKTTGVCHPGTQGVQGGREHEKTGCGQGGQVGREHEKTGFGHPGTQGGQVGNEQEKKGMIEKIQEKLPGHK |
| P40 | MSETRDEYGNQVRQTDEYGNPIQHTGTGTKAGAGIHAGGHGIGTGGGGGQGKLHRSGSGSSSDEDDGQGGRRKKGLTEKIKEKLPGGQKTTGVGYPGNQGVQGGRGHEKTGVCHPGTQGVQGGREHEKTGFGHPGTQGGHEHEKTGVGHPGTQGEQEKKGMIEKIKEKLPGHK |
| P41 | MSETRDEYGNQVRQTDEYGNPIQHTGTGTKAGAGIHAGGHGIGTGGGGGQGKLHRSGSGSSSDEDDGQGGRRKKGLTEKIKEKLPGGQKTTGVGYPGNQGVQGGREHEKTGVCHPGTQGVQGGREHEKTGFGHAVTQGGQVGNEQEKKGMIEKIQEKLPGHK |
| P42 | MSQARDEYGSQVRQTDEYGNPIQHTGTGTTAGTGIHGGGHGIGTGGGGGGQGKLHRSGSGSSSDEDDGQGGRRNRGLTEKIKEKLPGGNKTTGVCHPGTQGVQGGREHEKTGCGQGGQVGREHEKTGFGHPGTQGGQVGNEQEKKGMIEKIQEKLPGHK |
| P43 | MSETRDEYGNQVSQTDEYGNPIQHTGTGTKGGAGIHGGGHGIGTGGGGGQGKLHRSGSGSSSDEDDGQGGRRKKGLTEKIKEKLPGGNKTTGVCHPGTQGVQGGREHEKTGCGQGGQGGQVGREHEKTGFGHPVTQGGQVGNEQEKKGMIEKIKEKLPGHK |
| P44 | MSVTRDEYGNQVRQTDEYGNPIQHTGTGTKAGLGIHGGGHGIGTGGGGGGQGKLHRSGSGSSSDEDDGQGGRRKKGLTEKIKEKLPGGNKTTGVCHPGTQGVQGGREHEKTGCGQGGQGGQVGREHEKTGFGHPVTQGGQVGNEQEKKGMIEKIQEKLPGHK |
| P45 | MSVTRDEYGNQVRQTDEYGNPIQHTGTGTKAGLGIHGGGHGIGTGGGGGGQGKLHRSGSGSSSDEDDGQGGRRKKGLMEKIKEKLPGGNKTTGVCHPGTQGVQGGREHEKTGCGQGGQVRREHEKTGFGHPGTQGGQVGNEQEKKDMIEKIQENLPGHK |
| P46 | MSVTRDEYGNQVRQTDEYGNPIQHTGTGTKGGAGIHGGGHGIGTGGGGGQGKLHRSGSGSSSDEDDGQGGRRKKGLTEKIKEKLPGGQKTTGVGYPGNQGVQGGREHEKTGVCHPGTQGVQGGREHEKTGFGHPGTQGGHEHEKTGVGHPGTQGEQEKKGMIEKIKEKLPGHK |
| P47 | MSETRDEYGNQVRQTDEYGNPIQHTGTGTKAGLGIHGGGHGIGTGGGGGGQGKLHRSGSGSSSDEDDGQGGRRKKGLTEKIKEKLPGGQKTTGVCHPGTQGVQGGREHEKTGCGQGGQGGQVGREHEKTGFGHPVTQGGQVGNEQEKKGMIEKIQEKLPGHK |
| P48 | MSETRDEYGNQVSQTDEYGNPIQHTGTGTKGGAGIHGGGHGIGTGGGGGQGKLHRSGSGSSSDEDDGQGGRRKKGLTEKIKEKLPGGNKTTGVCHPGTQGVQGGREHEKTGCGQGGQGGQVGREHEKTGFGHPVTQGGQVGNEQEKKGMIEKIQEKLPGHK |
| P49 | MSVTRDEYGNQVRQTDEYGNPIQHTGTGTKAGSGIHGGGHGIGTGGGGGGQGKLHRSGSGSSSDEDDGQGGRRKKGLTEKIKEKLPGGNKTTGVCHPGTQGVQGGREHEKTGCGQGGQVRREHEKTGFGHPGTQGGQVGNEQEKKGMIEKIQENLPGHK |
| P50 | MSETRDEYGNVVRQTDEYGNPIQHTGTGTKGGAGIHGGGHGIGTGGGGGQGKLHRSGSGSSSDEDDGQGGRRKKGLTEKIKEKLPGGQKTTTGVGYPGTQGVQGGREHEKTGVCHPGTQGVQGGREHEKTGFGHAVTQGGQVGNEQEKKGMIEKIQEKLPGHK |
| P51 | MSETRDEYGNQVSQTDEYGNPIQHTGTGTKGGAGIHGGGHGIGTGGGGGQGKLHRSGSGSSSDEDDGQGGRRKKGLTEKIKEKLPGGQKTTTGVGYPGTQGVQGGREHEKTGVCHPGTQGVQGGREHEKTGFGHPGTQGGHEHEKTGVGHPGTQGEQEKKGMIEKIKEKLPGHK |
| P52 | MSVTRDEYGNQVRQTDEYGNPIQHTGTGTKAGSGIHGGSHGIGTGGGGGGGGQGKLHRSGSGSSSDEDDGQGGRRKKGLTEKIKEKLPGGNKTTGVCHPGTQGVQGGREHEKTGCGQGGQGGQVGREHEKTGFGHPVTQGGQVGNEQEKKGMIEKIQEKLPGHK |
| P53 | MSETRDEYGNQVSQTDEYGNPIQHTGTGTKAGAGIHAGGHGIGTGGGGGQGKLHRSGSGSSSDEDDGQGGRRKKGLTEKIKEKLPGGQKTTGVGYPGNQGVQGGREHEKTGVCHPGTQGVQGGREHEKTGFGHPGTQGGHEHEKTGVGHPGTQGEQEKKGMIEKIKEKLPGHK |
| P54 | MSETRDEYGNVVRQTDEYGNPIQHTGTGTKGGAGIHGGGHGIGTGGGGGQGKLHRSGSGSSSDEDDGQGGRRKKGLTEKIKEKLPGGQKTTTGVGYPGNQGVQGGREHEKTGVCHPGTQGVQGGREHEKTGFGHPGTQGGHEHEKTGVGHPGTQGEQEKKGMIEKIKEKLPGHK |
| P55 | MSQTRDEYGSQVRQTDEYGNPIQHTGTGTKAGAGIHGGGHGIGTGGGGGGQGKLHRSGSGSSSDEDDGQGGRRKKGLTEKIKEKLPGGNKTTGVCHPGTQGVQGGREHEKTGCGQGGQVGREHEKTGFGHPGTHGGQVGNEQEKGMIEKIQEKLPGHK |
| P56 | MSETRDEYGNQVRQTDEYGNPIQHTGTGTKGGAGIHGGGHGIGTGGGGGQGKLHRSGSGSSSDEDDGQGGRRKKGLTEKIKEKLPGGNKTTGVCHPGTQGVKGGREHEKTGCGQGGQGGQVGREHEKTGFGHPVTQGGQVGNEQEKKGMIEKIQEKLPGHK |
| P57 | MSVTRDEYGNQVRQTDEYGNPIQHTGTGTQAGSGIHGGGHGIDTGGGQGKLHRSGSGSSSDEDDGQGGRRKKGLTEKIKEKLPGGQKTTGVGYPGNQGVQGGREHEKTGVCHPGNQGVQGGREHEKTGFGHPGTQGGHEHEKTGVGHPGTQGEQEKKGMIEKIKEKLPGHK |
| P58 | MSVTRDEYGNQVRQTDEYGNPIQHTGTGTQAGSGIHGGGHGIDTGGGQGKLHRSGSGSSSDEDDGQGGRRKKGLTEKIKEKLPGGNKTTGVCHPGTQGVQGGREHEKTGCGQGGQGGQVGREHEKTGFGHPVTQGGQVGNEQEKKGMIEKIQEKLPGHK |

P1:ZJAJ51 ZJAJ53 ZJAJ54 ZJAJ251 ZJAJ255 ZJAJ341 ZJAJ562 ZJSY42 ZJSY45 ZJSY49 ZJSY64 ZJSY613 ZJSY72 ZJSY73 ZJSY75 ZJSY914 ZJSY916 ZJSY917 JXLS55 JXLS510 JXLS513 FJWYS5123 FJWYS43 HXN29 HBXN31 HBXN329 HBXN62 HBEX818 HBEX83 HBEX84 HBEX15 HBEX22 HBEX24 HNSN54 HNSN55 HNSN56 GXLY3133 GXLY3136 GXLY111 GXLY112 GXLY19 GXMES2364 GXMES2366 GXMES2368 GXHP114 GXHP115 GXHP111 GZYJ17322 GZYJ17323 GZYJ17324 GZYJ4454 GZYJ4455 GZYJ53163 GZYJ53164 GZYJ53165 GZYJ325412 GZYJ325413 GZYJ325415 GZST64 GZST66 GZST624 GZST910 GZST911 GZST916 GZST138 GZST136 GZST139 SCYY91 SCYY95 SCYY911 SCYY518 SCYY528 SCYY531 YNXC475 YNXC93 YNXC715 YNXC7113 YNXC7112

P2:ZJAJ342 ZJAJ343 ZJAJ349 ZJAJ3411 ZJAJ3412 ZJAJ561 ZJAJ564 AHJXQ231 AHJXQ234 AHHS78 AHHS710 FJWYS212 FJWYS45 FJWYS47

P3:ZJAJ253 AHJXQ41 AHJXQ43 AHJX1215 AHJX125 AHJX126 AHJX1220

P4:HXN21 HXN24 HBEX12 HBEX14 HBEX27 HBEX66 HBEX69 HBEX610

P5:HBXN320 HBXN69 HBXN616 GXLY41 GXLY44 GXLY47 GXHP4104 GXHP41018 GXHP510 GXHP515

P6:ZJSY66 AHHS27 AHHS29 AHHS210 AHHS63 AHHS66 JXLS23 JXLS25 JXLS27 JXLS31 JXLS34 JXLS38 FJWYS24 FJWYS27 HBXN11 HBXN15 HBXN112 HBXN116 HNSN11 HNSN14 HNSN16 HNSN41 HNSN43 HNSN44 GZXS23 SCXY71 SCXY257 SCXY259 SCXY2512 SCXY272 SCXY276 SCYY81 SCYY83 SCYY86 YNXC1611 YNXC1616

P7:YNXC1619

P8:GXMES131 GXMES132 GXMES133 GXMES2491 GXMES2492 GXMES2496 GZLP241 GZLP242 GZLP244 GZLP261 GZLP262 YNXC478 YNXC4722

P9:GZXS24 GZXS22 GZXS523 SCXY72 SCXY73

P10:GZXS56 GZXS520 SCXY274

P11:GZLP264 GZLPD1014 GZLPD710 GZLPD714 GZLPD716

P12:YNXC92 YNXC95 YNMG166 YNMG167 YNMG631 YNMG41 YNMG43 YNMG38 YNMG39 YNMG310 YNJP827 YNJP1014 YNJP101 YNJP1017 YNJP54 YNJP55 YNJP5915

P13:FJWYS5122

P14:FJWYS5128

P15:GXLY3132

P16:GXHP513

P17:YNJP828

P18:Hershey152 Hershey313 BK23 BK261 BK27 MSL29 MSL56 MSL58 LYS51 LYS53 LYS58

P19:Hershey2824 Hershey314 BK610 BK66 ZZY26 ZZY615 NK15 NK613 LYS16

P20:Hershey283 Hershey61 Hershey64 ZZY45 ZZY55 NK14 NK45

P21:Hershey68

P22:Hershey72 NK112

P23:BK31 BK38 MSL48

P24:MSL114 MSL115 MSL73 MSL210 MSL37

P25:BK61 ZZY46

P26:MSL45

P27:MSL316 MSL77 LYS12

P28:Hershey71 ZZY412 NK412

P29:ZZY212 ZZY632

P30:NK33 LYS27

P31:NK27

P32:MSL121 NK52 NK59

P33:NK43 NK46 NK55 NK58 LYS43 LYS414 NK451

P34:2LYS74

P35:BK53 BK511 BK513 BK82 BK84 BK85 ZZY25 ZZY34 ZZY35 ZZY315 ZZY53 ZZY62 NK216 LYS82

P36:ZZY511 NK23 LYS62 LYS67 LYS68 LYS78

P37:BK12 BK14 BK381 NK35 NK37

P38:LYS73

P39:Hershey155

P40:Hershey158

P41:Hershey286

P42:Hershey312

P43:MSL21

P44:MSL223

P45:MSL315

P45:MSL43

P47:MSL53

P48:MSL510

P49:MSL78

P50:NK66

P51:NK615

P52:LYS11

P53:LYS161

P54:LYS24

P55:LYS28

P56:LYS4141

P57:LYS87

P58:LYS88
